# Biogeographical Regions and Climate Change: Lanternfishes Shed Light on the Role of Climatic Barriers in the Southern Ocean

**DOI:** 10.1111/gcb.70256

**Published:** 2025-06-16

**Authors:** Cam Ly Rintz, Philippe Koubbi, Berta Ramiro‐Sánchez, Clara Azarian, Jilda Alicia Caccavo, Cédric Cotté, Eric Goberville, Claire Godet, Percy Alexander Hulley, Rémy Le Goff, Fabien Leprieur, Marine Robuchon, Baptiste Serandour, Boris Leroy

**Affiliations:** ^1^ Laboratoire de Biologie Des Organismes et Des Écosystèmes Aquatiques‐BOREA Muséum National d'Histoire Naturelle (MNHN), SU, CNRS, IRD, UA Paris France; ^2^ Laboratoire D'océanographie et du Climat: Expérimentations et Approches Numériques (LOCEAN/IPSL) UPMC, CNRS, IRD, Muséum National D'histoire Naturelle, Sorbonne Université Paris France; ^3^ IFREMER Centre Manche Mer du Nord Unité Halieutique Manche‐Mer du Nord Boulogne‐sur‐Mer France; ^4^ Centro Oceanográfico de Santander Instituto Español de Oceanografía, CSIC Santander Spain; ^5^ Ecole Nationale Des Ponts et Chaussées (ENPC) Champs‐sur‐Marne France; ^6^ Laboratoire Des Sciences du Climat et de L'environnement (LSCE/IPSL), CEA, CNRS, UVSQ Université Paris‐Saclay Gif‐sur‐Yvette France; ^7^ Department of Marine Biology Iziko‐South African Museum Cape Town South Africa; ^8^ UMR MARBEC CNRS, IRD, IFREMER, Univ Montpellier Montpellier France; ^9^ European Commission Joint Research Centre Ispra VA Italy; ^10^ Department of Ecology, Environment and Plant Sciences Stockholm University, Universitetsvägen 10A Stockholm Sweden

**Keywords:** biogeographical regions, climate change, climatic barrier, community‐level modelling, distribution shift, mesopelagic, Myctophidae, Southern Ocean

## Abstract

To predict the spatial responses of biodiversity to climate change, studies typically rely on species‐specific approaches, such as species distribution models. In this study, we propose an alternative methodology that investigates the collective response of species groups by modelling biogeographical regions. Biogeographical regions are areas defined by homogeneous species compositions and separated by barriers to dispersal. When climate acts as such a barrier, species within the same region are expected to respond similar to changing climatic conditions, enabling the prediction of entire region shifts in response to future climate scenarios. We applied this approach to the Southern Ocean, which exhibits sharp climatic transitions known as oceanic fronts, focusing on the mesopelagic lanternfishes (family Myctophidae). We compiled occurrence data for 115 lanternfish species from 1950 onwards and employed a network‐based analysis to identify two major biogeographical regions: a southern and a subtropical region. These regions were found to be distinct, with minimal overlap in species distributions along the temperature gradient and a separation around 8°C, indicating that temperature likely acts as a climatic barrier. Using an ensemble modelling approach, we projected the response of these regions to future temperature changes under various climate scenarios. Our results suggest a circumpolar expansion of the subtropical region and a contraction of the southern region, with the Southern Ocean becoming a cul‐de‐sac for southern species. Ultimately, our results suggest that when support is found for the climatic barrier hypothesis, community‐level models from a ‘group first, then predict’ strategy may effectively predict future shifts in species assemblages.

## Graphical abstract

We developed a method to predict how groups of species may shift with climate change by modelling biogeographical regions—areas with similar species. Focusing on lanternfishes in the Southern Ocean, we found two main regions separated by temperature. Because climate acts as a barrier, species within each region may respond similarly to climate change. This allowed us to project that ocean warming may drive subtropical species southward, while southern species adapted to colder waters may lose habitat and retreat toward Antarctica.

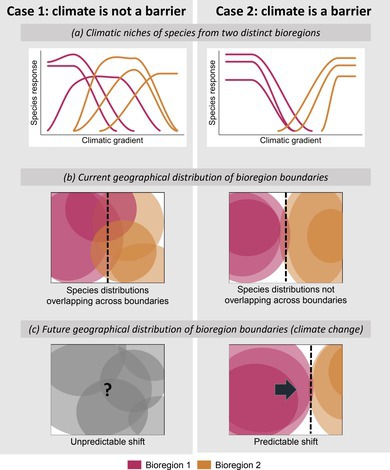

## RÉSUMÉ

Les études prédictives sur les réponses spatiales de la biodiversité au changement climatique reposent généralement sur des approches espèce par espèce, comme les modèles de distribution d’espèces. Dans cette étude, nous proposons une méthodologie alternative qui explore la réponse collective de groupes d’espèces en modélisant des régions biogéographiques. Les régions biogéographiques sont des zones définies par une composition spécifique homogène et séparées par des barrières à la dispersion. Lorsque le climat agit comme une barrière, on s’attend à ce que les espèces d’une même région répondent de manière similaire à des changements de conditions climatiques, permettant ainsi de prédire les déplacements de régions entières en réponse à des scénarios climatiques futurs. Nous avons appliqué cette approche à l’Océan Austral, qui présente d’abruptes transitions climatiques correspondant à des fronts océaniques, en nous concentrant sur les poissons‐lanternes mésopélagiques (famille Myctophidae). Nous avons compilé des données d’occurrence pour 115 espèces de poissons‐lanternes depuis 1950, et effectué une analyse basée sur les réseaux pour identifier deux régions biogéographiques majeures : une région australe et une région subtropicale. Celles‐ci se sont révélées distinctes, avec un chevauchement limité entre les distributions d’espèces le long du gradient de température et une séparation autour de 8°C, ce qui indique que la température pourrait agir comme une barrière climatique. En appliquant une approche de modélisation d’ensemble, nous avons projeté la réponse de ces régions en fonction de futurs changements de température selon divers scénarios climatiques. Nos résultats suggèrent une expansion circumpolaire de la région subtropicale et une contraction de la région australe, où l’Océan Austral formerait un cul‐de‐sac pour les espèces polaires. Enfin, nos résultats suggèrent que, lorsque l’hypothèse d’une barrière climatique est vérifiée, les modèles de communautés suivant la stratégie “assembler, puis prédire” pourraient effectivement prédire les potentiels déplacements d’assemblages d’espèces.

## 1 Introduction

A major response of species to climate change is the shifting of their distributions to track suitable abiotic conditions (Parmesan and Yohe 2003; Bellard et al. 2012), particularly in marine environments where organisms face fewer obstacles to their dispersion than on land (Poloczanska et al. 2014; Lenoir et al. 2020; Pinsky et al. 2020). Modelling future distribution shifts under climate change scenarios is a common approach to predict the impacts of climate change and derive future biodiversity distribution patterns. These predictions, however, are generally made on a single‐species basis (Guisan and Thuiller 2005; Elith and Leathwick 2009; Araújo et al. 2019), which introduces uncertainty. This uncertainty arises because biotic interactions are expected to change as species shift in space and encounter new environments and interact with other species, making it difficult to predict the future geographic distribution of biodiversity (Davis et al. 1998; Tylianakis et al. 2008).

To move beyond single‐species predictions, joint species distribution models (i.e., models accounting for both biotic and abiotic factors) are being developed to forecast biodiversity (Dormann et al. 2018; Gordó‐Vilaseca et al. 2024). Alternatively, exploring assemblage‐level responses rather than species‐level ones can be useful. This approach helps predict the potential impacts of climate change on ecosystems as a whole. For example, over large geographical areas, species are spatially clustered in distinct regions of homogeneous composition called biogeographical regions (also referred to as bioregions or ecoregions; Vilhena and Antonelli 2015; Lomolino et al. 2016; Leroy et al. 2019; Hill et al. 2020; Woolley et al. 2020). Biogeographical regions have a distinct composition because they are separated by barriers to species dispersal, which can be ‘hard’ barriers (e.g., continents separating oceans) or ‘soft’ (e.g., oceanic currents, climatic variations; Briggs and Bowen 2013; Ficetola et al. 2017; Kocsis et al. 2018). In particular, investigating climatic barriers may help forecast expected shifts of biogeographical regions under climate change.

**FIGURE 1:**
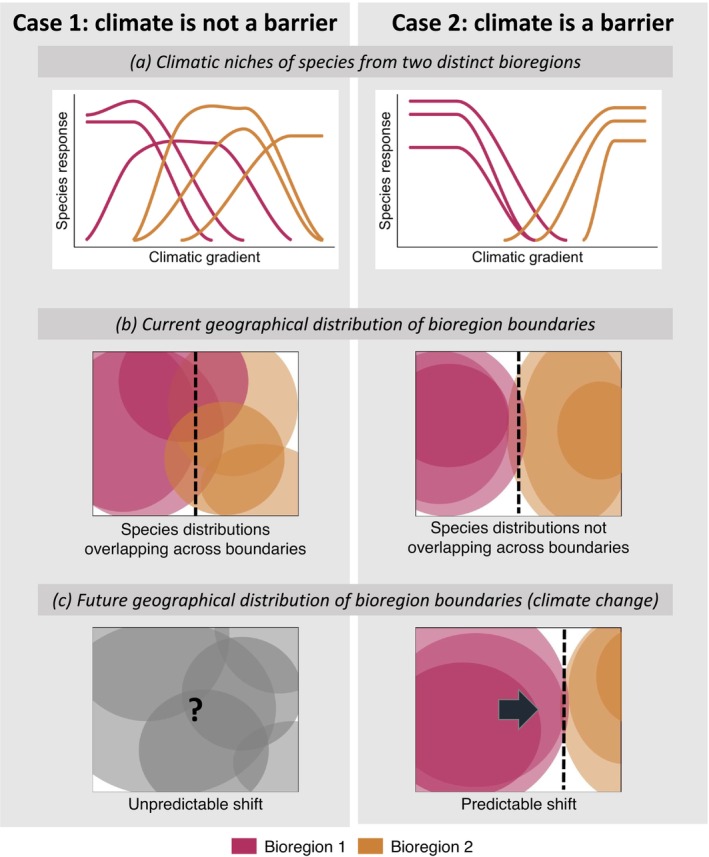
Conceptual diagram representing hypotheses of the effect of climate at the boundary between two biogeographical regions. The left column (case 1) represents a case where the climatic boundary between the two regions is not an impermeable barrier; the right column (case 2) represents a case where the climatic boundary acts as a barrier limiting dispersal between regions. (a) Responses of species to the climatic gradient at the boundary between regions. (b) Current geographical distributions of species at the boundary between regions. (c) Shifts in distributions of species at the boundary between regions, following climate change.

For such predictions to be realistic at the scale of biogeographical regions, these must be composed of species whose dispersal is restricted by a climatic boundary. However, climate boundaries are rarely impermeable barriers (Whittaker 1975), especially in oceans (Kocsis et al. 2018). When climate is not a barrier, we can generally expect species to have species‐specific responses to the climate gradient at the boundary between two regions (Figure 1a, left panel). Hence, their distributions are likely to extend across region boundaries (Figure 1b, left panel). In such a case, the individual responses of species to the climatic boundary will likely result in variable range shifts across species in response to climate change. These variable responses will alter biotic interactions, making it impossible to predict future region boundaries (Figure 1c, left panel). However, in a case where climate acts as a barrier between regions with similar responses for all species (Figure 1a, right panel), we can expect similar shifts for all species. If species distributions share the same shifts in space, then current biotic interactions are likely to remain stable in the future, resulting in predictable shifts in region boundaries (Figure 1b,c, right panel).

In this study, we hypothesise that a good candidate for a climatic barrier between biogeographical regions is the boundary between the Southern Ocean and other oceans. The Southern Ocean is not geographically limited by continents to its north but is bounded by large oceanographic features such as frontal zones, which exhibit strong variations in oceanographic conditions associated with the Antarctic circumpolar current (ACC) (Post et al. 2014). Oceanic fronts are separations between water masses with different physical and biogeochemical properties (Chapman et al. 2020). In the Southern Ocean, there are five successive annular fronts over the latitudinal gradient, which may act as climatic boundaries with other oceans (Orsi et al. 1995). These fronts typically display abrupt changes in water conditions, notably in water temperature, and are from north to south the Subantarctic Front (SAF), the Polar Front and the Southern ACC Front. The southern boundary delimits the extent of the ACC to the south, while the Subtropical Front (STF) marks its northern limit. These oceanic fronts and the strong eastward flow of the ACC could act as a soft barrier to the dispersal of pelagic marine species between different regions (Figure 1, right panel).

We explore the hypothesis of an oceanographic climatic barrier that delimits the biogeographical regions of a group of pelagic fishes, the lanternfishes (Myctophidae family). As ectothermic organisms, these fishes are sensitive to water temperature, which may limit their habitat occupancy. Previous observations have shown the influence of oceanic fronts on their distribution (Collins et al. 2012; Freer et al. 2019; Woods et al. 2023) and a separation between species assemblages in the Southern Ocean and the Subtropical zone at the regional scale, in the Indian sector (Koubbi et al. 2011, 2023). In terms of biomass, myctophids account for most of the mesopelagic fishes found at depths between 200 and 1000 m in the Southern Ocean (Gjøsaeter et al. 1980; Filin et al. 1990; Collins et al. 2008; Duhamel et al. 2014; Caccavo et al. 2021). These species are a key component of mesopelagic ecosystems, given their intermediate position in trophic networks: they channel energy from zooplankton, especially euphausiids, copepods and amphipods towards top predators such as large fishes, penguins, squids, seals and flying seabirds (Kozlov 1995; Cornejo‐Donoso and Antezana 2008; Cherel et al. 2010; Saunders et al. 2019). Like many mesopelagic fishes, they also play an important role in carbon sequestration in the ocean (Radchenko 2007; Davison et al. 2013), linked to their diel vertical migration between deep waters during the day and the epipelagic zone (10–100 m) at night (Duhamel et al. 2000, 2014).

Given their critical role in Southern Ocean ecosystems, changes in myctophid distribution would have significant ecological implications (Caccavo et al. 2021). Their sensitivity to water temperature raises concerns regarding future impacts on their distribution linked to climate change. The Southern Ocean plays a critical role in global climate, and its sensitivity to climate change is driven by numerous and complex physical mechanisms related to ocean circulation and ice shelf melting. Between 1970 and 2017, the upper 2000 m of the ocean south of 30° S—which represents 25% of global ocean area—accounted for 35%–43% of the global ocean heat uptake, with an increasing proportion in recent years (Good et al. 2013; Cheng et al. 2017; Ishii et al. 2017; Meredith et al. 2019). Polar species are particularly vulnerable to warming because of their narrow thermal tolerance and the long time required to acclimate (Peck et al. 2014). They also have limited available space for distribution shifts, due to the unique configuration of the Southern Ocean surrounding the Antarctic continent, making it a cul‐de‐sac for southward shifts. As a result, the warming of polar climates would lead to the shrinking of polar habitats and a range contraction of marine polar species (Parmesan 2006; Garcia et al. 2014; Constable et al. 2014; Meredith et al. 2019; Atkinson et al. 2019). This is corroborated by myctophid studies, as individual distribution models applied to several southern lanternfish species predict a poleward shift and highlight the importance of thermal tolerance and habitat availability (Freer et al. 2019; Liu et al. 2024).

Here, our aim is to characterise the distribution of all myctophids occurring south of latitude 30° S and to predict how these species would be impacted by projected climate change. We make the first delineation of myctophid biogeographical regions at the scale of the entire southern and subtropical area. Following our conceptual framework (Figure 1), we then explore the hypothesis of climatic barriers driving region boundaries. Finally, we use environmental predictors to model their current and future distribution under several climate change scenarios.

## 2 Materials and Methods

**FIGURE 2:**
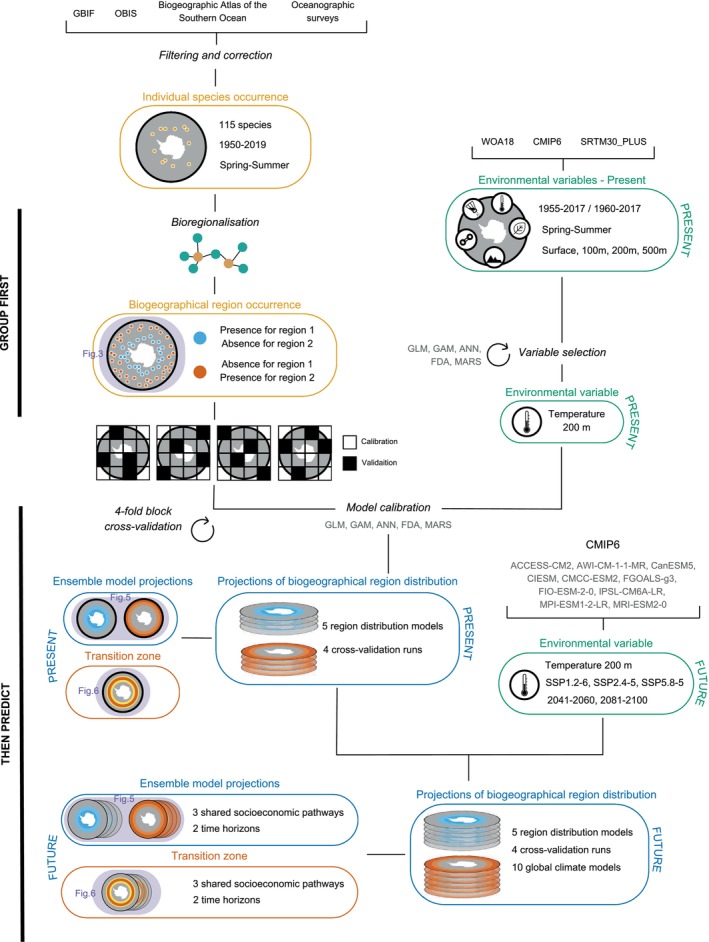
Methodological diagram. This diagram represents the multiple steps in the analysis, displaying the type of input data, the ‘group first, then predict’ processing and the outputs.

An overview of the methodology used in this study is provided below in the form of a schematic diagram (Figure 2), while the following sections describe each step in detail.

### 2.1 Species Occurrence Database

We compiled and consolidated an occurrence database of myctophids in the study area through a two‐step process. First, we compiled global distribution data for myctophids, and second, we filtered this global data set to include only species occurring in the study area (south of 30° S). We assembled the global database from multiple sources: the Ocean Biodiversity Information System (see Appendix S1), the Global Biodiversity Information Facility (see Appendix S1) and the Biogeographic Atlas of the Southern Ocean (Duhamel et al. 2014). Additionally, we incorporated supplementary data from recent oceanographic surveys conducted around Crozet, Kerguelen, St Paul and New Amsterdam from 2017 to 2019 (Koubbi et al. 2023).

The global database contained 260 species. We filtered this data set by applying a minimum cut‐off of 10 occurrences in the study area to exclude vagrant species. However, to avoid excluding rare species and species endemic to the study area, we implemented a second rule: we included all species with more than 50% of their occurrence records within the study area. We tested how different occurrence cut‐off thresholds affect the number of species selected for analysis and found that varying thresholds do not substantially change the number of species chosen, supporting our threshold choice (see Appendix S2). We retained species data recorded between 1950 and 2019, focusing on spring and summer (October to March), as these were the best‐sampled periods. We aggregated records onto a grid with 1° spatial resolution, considering multiple records of a species within the same grid cell as a single presence.

For the global filtered data set, we employed a two‐step procedure to check for errors in the occurrence data. First, we automatically checked for coordinate errors, such as equal longitude and latitude values, zero values and records on land, using the *CoordinateCleaner* R package (v2.0‐20; Zizka et al. 2019). Second, we manually reviewed the records for all species to remove dubious or uncertain occurrences based on our expert knowledge. The final data set comprised 115 species. We documented all corrections in Appendices S3 and S4.

### 2.2 Environmental Data

To predict myctophid regions, we pre‐selected several oceanic variables known to influence myctophid distributions (Koubbi et al. 2011; Duhamel et al. 2014; Freer et al. 2019): temperature, salinity, oxygen, chlorophyll a concentration and bathymetry. We extracted variables at 0, 100, 200 and 500 m depth, which represent depths at which lanternfishes spend time due to their nycthemeral vertical migrations.

To calibrate our distribution models with an environmental baseline, we downloaded values of temperature and salinity from the 2018 World Ocean Database (Boyer et al. 2018) for the 1955–2017 period and oxygen for the 1960–2017 period. Chlorophyll data, not available in this database, were extracted from the available outputs of the two global climate models CESM2‐WACCM and GFDL‐ESM4 from the Coupled Model Intercomparison Project 6 (CMIP6, Eyring et al. 2016) for the 1955–2017 period. For all variables (temperature, salinity, oxygen and chlorophyll), we averaged values from the spring and summer periods (October to March in the Southern Hemisphere) because the majority of myctophid samples were collected during these periods. For bathymetry, we used the SRTM30_PLUS data set (Becker et al. 2009). All variables were gridded at a 1° resolution within the study area, ranging from 30° S to 90° S. More details are given in Appendix S5.

To project the impacts of climate change on myctophid regions, we used 10 models (ACCESS‐CM2, AWI‐CM‐1‐1‐MR, CanESM5, CIESM, CMCC‐ESM2, FGOALS‐g3, FIO‐ESM‐2‐0, IPSL‐CM6A‐LR, MPI‐ESM1‐2‐LR and MRI‐ESM2‐0; see Appendix S6) from the CMIP6 (Eyring et al. 2016) and three emission scenarios, also known as Shared Socioeconomic Pathways (SSPs): SSP1‐2.6 (strong mitigation scenario), SSP2‐4.5 (modest mitigation) and SSP5‐8.5 (no mitigation; O'Neill et al. 2016). For each scenario, we computed the multi‐model mean of the environmental variables of interest over two time horizons based on the IPCC reference periods for the medium‐term (2041–2060) and the long‐term (2081–2100) (Ara Begum et al. 2022). We selected models based on the availability of outputs for our chosen variables, scenarios and time horizons as well as the performance of each model in simulating sea surface temperature and salinity (Liu et al. 2022). To maintain consistency with our established environmental baseline, we applied a bias correction procedure by adding the projected changes to the baseline instead of using the absolute model outputs. To achieve this, we calculated the anomalies projected by each model (i.e., the difference between future and historical values) per grid cell and applied them to our baseline values. These anomalies were calculated based on the ‘historical’ period of the CMIP6 models, which spans from 1950 to 2014. This differs slightly from the baseline period of observed temperature data (1955–2017), but we opted to maintain the standard historical period defined in CMIP6 to ensure consistency with other studies using these data sets. To ensure that this temporal mismatch would not significantly affect the final results, we compared the CMIP6 historical simulations and the observed baseline of temperature using a normalised Taylor diagram (see Appendix S7).

### 2.3 Bioregionalisation

To predict current and future distributions of myctophid regions, we applied a two‐step procedure referred to as ‘group first, then predict’ (Ferrier and Guisan 2006; Hill et al. 2020; Woolley et al. 2020). First, we identified biogeographical regions based on species occurrence records, then we modelled these regions with appropriate environmental predictors to make current and future predictions.

#### 2.3.1 Identifying Biogeographical Regions

To identify the biogeographical regions in our study zone, we followed the biogeographical network approach introduced by Vilhena and Antonelli (2015). This approach represents the distribution of species as a network composed of nodes corresponding to either species or sites (grid cells), with links between these two types of nodes. A species can be linked to multiple sites, and a site can include multiple species; however, species–species or site–site links are not allowed in this type of network. Biogeographical networks offer several advantages over other classification approaches, such as similarity‐based hierarchical clustering. Most importantly, they do not lose species identity during the region delineation process, which improves the robustness of regions and allows for analysis from the perspective of species (Vilhena and Antonelli 2015; Bloomfield et al. 2018; Leroy et al. 2019). Furthermore, they are robust to species richness imbalance and do not require data adjustments to balance sites (Leroy et al. 2019). This is particularly useful given that our database is a compilation of various sources with different protocols and sampling efforts, especially in the Southern Ocean where sampling is highly spatially heterogeneous, due to accessibility reasons (e.g., distance to the coast, shipping routes, research stations or protected areas) (Griffiths et al. 2014).

On one hand, we used the Gephi software (Bastian et al. 2009) to visualise the network using the spatialisation algorithm *Force Atlas 2*. This algorithm groups nodes that are strongly interconnected (i.e., grid cells sharing species) and spreads them away from all other nodes that are not interconnected (i.e., sites that have few or no species in common). On the other hand, to identify regions, we clustered species and sites by applying the community‐detection algorithm *Infomap* based on the *Map Equation* approach (Rosvall and Bergstrom 2008; Smiljanić et al. 2023), which has been recommended for biogeographical studies (Vilhena and Antonelli 2015; Costello et al. 2017; Bloomfield et al. 2018; Leroy et al. 2019). This algorithm aims to maximise intra‐group links and minimise inter‐group links (Rosvall and Bergstrom 2008), aligning well with the definition of a biogeographical region as a composition of sites sharing many common species and differing from the species composition of other regions. However, *Infomap* tends to detect minor clusters composed of very few species restricted to very few sites, such as in cases of localised endemism or sampling bias. Therefore, we discarded clusters with fewer than 20 nodes as our aim was to study large biogeographical regions at the scale of the Southern Ocean.

Because *Infomap* is stochastic, we ran the algorithm 100 times to obtain the optimal clustering result (the partition with the shortest description length). We used the *biogeonetworks* R package (v0.1.2; Leroy 2019) to process the network and calculate the species fidelity to each cluster, defined as:

$$\mathrm{Fi}=\frac{\text{Occurrence of species}\;i\;\text{in the region}}{\text{Total occurrence of species}\;i}$$

Species with high fidelity to a given region are unlikely to be found in other clusters. This metric is useful for characterising the cohesion of regions (high species fidelity) and for identifying potential transition regions (low species fidelity).

#### 2.3.2 Assessing the Likelihood of the Climatic Barrier Hypothesis

To evaluate whether our study model supports the hypothesis of a climate barrier between regions (Figure 1), we analysed the response of all species in our database to various environmental variables. For each species, we visualised its density curve along each environmental gradient. We then examined the overlap between regions: the absence of overlap would indicate the presence of a climatic barrier.

### 2.4 Modelling Biogeographical Regions

To project biogeographical regions across space and time, we modelled the distribution of each region identified by the clustering results using corresponding environmental variables.

#### 2.4.1 Model Calibration

To model biogeographical regions, we used a presence–absence framework. For each region, we defined as presences the cells assigned to that region by the clustering algorithm. We defined absences as the grid cells assigned to other regions, under the assumption that the species composition in these cells was sufficiently distinct. We then calibrated each model using these presence and absence locations, together with environmental values extracted from the baseline predictors.

We employed five distribution modelling techniques implemented in the *biomod2* R package (v3.5.1; Thuiller et al. 2021) with default settings: generalised linear models (GLM), generalised additive models (GAM), artificial neural networks (ANN), flexible discriminant analysis (FDA) and multivariate adaptive regression splines (MARS) (Thuiller et al. 2021; Guisan et al. 2017). Models were only applied to regions with sufficient data (i.e., more than 30 presences) for calibration. We combined the models using an ensemble modelling procedure to summarise the variability in their outputs and uncertainties, which is expected to provide more robust results than relying on a single model and its potential flaws (Araújo and New 2007; Marmion et al. 2009; Guisan et al. 2017).

#### 2.4.2 Variable Selection

We adapted a variable selection protocol from Leroy et al. (2014) and Bellard et al. (2016). First, we selected non‐collinear variables by calculating Spearman's correlation coefficient among all variables, grouping correlated variables with coefficients beyond a 0.7 threshold (Dormann et al. 2013), and keeping only one variable per group. Second, we calibrated all models for each region with the pre‐selected non‐collinear variables and calculated the importance of each variable by analysing the Pearson correlation coefficient (r) between the outputs of the model and those of a model where the variable is randomly resampled. The importance value is given by the formula:

Imp=1−r

The more a variable changes the predicted probabilities when it is resampled, the lower the r, and thus, the higher its importance. Third, for each region, we selected variables with a median importance greater than 10%. To ensure important variables were not mutually exclusive, we analysed the correlation among variable importances (distinct from collinearity analysis) and ensured they were never negatively correlated (r < −0.3).

#### 2.4.3 Model Evaluation

We assessed the ecological plausibility of the models by examining their response to each environmental variable using the evaluation strip method (Elith et al. 2005), which allowed us to discard unrealistic models (Schickele et al. 2021). Next, we evaluated the predictive performance of the models with a four‐fold block cross‐validation procedure. We split our data into several blocks, optimising their size to minimise autocorrelation between calibration and evaluation data sets (Roberts et al. 2017; Valavi et al. 2018). These blocks were randomly assigned to four folds, while ensuring that the four evaluation folds had balanced numbers of presences and absences. For these steps, we used the *blockCV* R package (v2.1.4; Valavi et al. 2018).

We evaluated each model for each region by calculating the Jaccard index, based on the similarity between model predictions and observations:

$$J=\frac{\mathrm{TP}}{\mathrm{TP}+\mathrm{FP}+\mathrm{FN}}$$

where TP represents ‘true positive’ (predicted presences matching observed presences), FP represents ‘false positive’ (predicted presences for observed absences), and FN represents ‘false negative’ (predicted absences for observed presences) (Leroy et al. 2018). We rejected models with over 50% wrong predictions, that is, with a Jaccard index value lower than 0.5.

This similarity index has the advantage of not being dependent on prevalence (i.e., the proportion of species presences among sampled sites), unlike metrics like AUC or TSS, which can be misleading in the case of a low prevalence (Lobo et al. 2008; Jiménez‐Valverde 2012; Leroy et al. 2018). However, as it is a widely used metric, we also provided AUC in Appendix S8 to enhance comparability with other studies.

#### 2.4.4 Ensemble Modelling and Model Projection

For each region, we calibrated a total of 20 models (five distribution modelling techniques x four cross‐validation folds). To map regions, we projected each model onto the baseline data set and onto each of the 10 global climate models for each scenario and time horizon, resulting in an ensemble of 20 predictions for the baseline period and 200 predictions for each future scenario and time horizon. We created an ensemble map for each time horizon and scenario by averaging the outputs of all projections. We calculated the uncertainty in predictions by determining the standard deviation of all predictions for each ensemble map.

To investigate the overlap between regions and thus the existence of transition zones, we calculated a Transition index (TI) designed to identify areas where multiple bioregions can occur simultaneously. In other words, this Transition index aims to highlight areas where the probability of occurrence is similar for multiple bioregions at the same time. We used the following formula:

$$\mathrm{TI}=-\sum_{i=1}^{N}\frac{p_{i}}{P}\log\left(\frac{p_{i}}{P}\right)$$

where $p_{i}$ is the probability of occurrence of region i, N is the total number of regions and $P=\sum_{i=1}^{N}p_{i}$. To map the transition zone, we normalised this index between 0 and 1 ($\frac{\mathrm{TI}}{{\mathrm{TI}}_{\max}}$), such that values close to 1 indicate areas where multiple regions are likely to occur at high probabilities, whereas values close to 0 indicate areas where only one region is likely to occur.

#### 2.4.5 Comparison Between Present and Future Projections

We quantified the potential gain or loss in area of the biogeographical regions for each scenario and each time horizon by calculating the difference in area between future and present distributions across five classes of occurrence probability, using a Lambert azimuthal equal‐area projection to ensure equal‐area grid cells. For each class, we sought to identify significant differences by conducting appropriate statistical tests. Based on the preliminary statistical assumptions, we either performed a paired t‐test to compare the means of all model outputs between the present scenarios or a sign test to compare the medians.

## 3 Results

### 3.1 Biogeographical Regions

**FIGURE 3:**
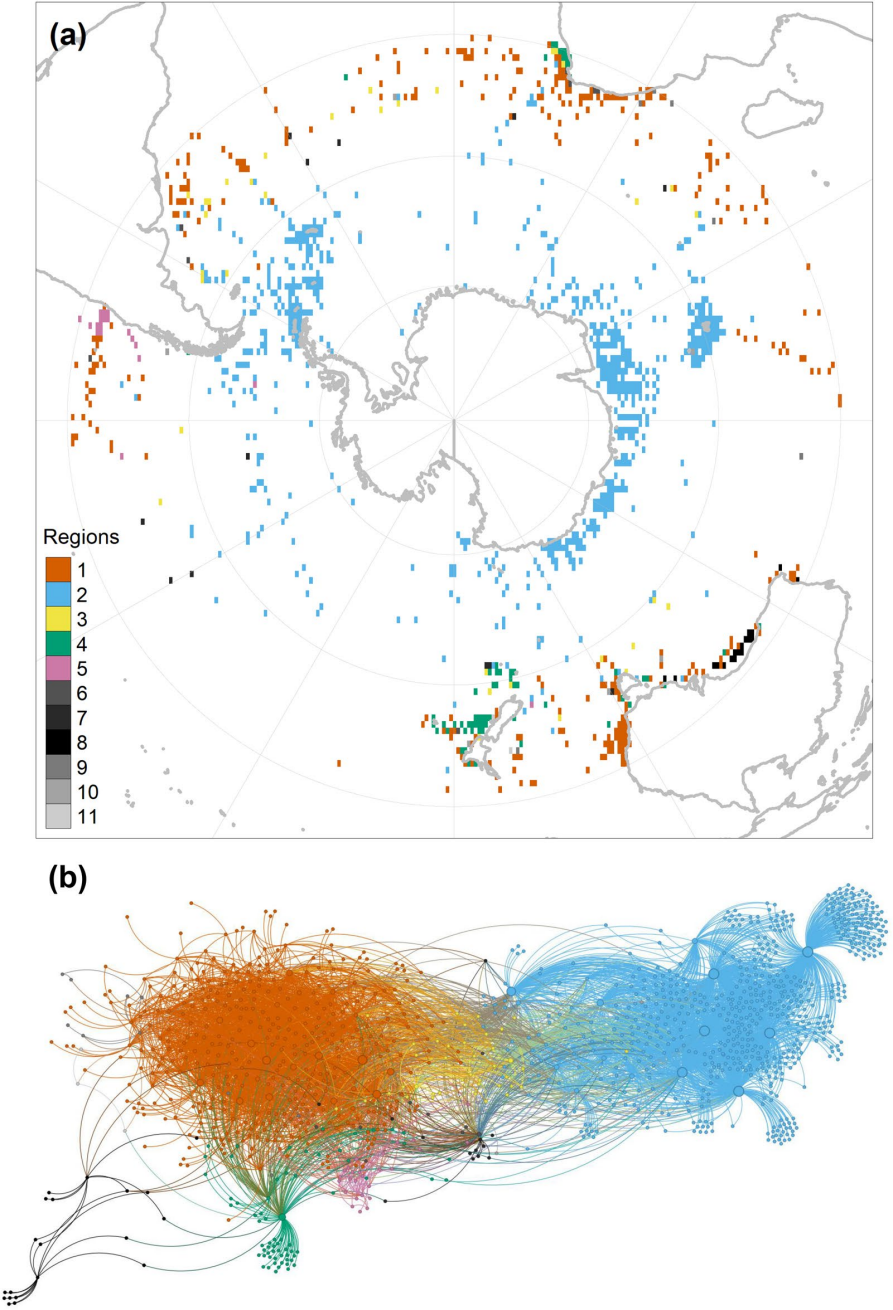
Biogeographical regions and biogeographical network of Myctophidae. (a) The map illustrates the 11 biogeographical regions identified south of 30° S, based on species composition of sampling sites. The five primary regions are highlighted in bright colours. (b) The biogeographical network comprises nodes and links, with each node representing either a species or a sampling site. A species can be linked to multiple sites (where it occurs), and a site can be linked to multiple species (which it contains). We spatialised the network in Gephi with the *Force Atlas 2* algorithm in order to group nodes that are strongly interconnected (i.e., grid cells that have species in common) and spread them away from all other nodes that are not interconnected (i.e., sites that have few or no species in common). The node size is proportional to the number of links to other nodes. Both species and sites are coloured according to their assigned region.

We identified five main clusters, with two prominent clusters notable for their extensive spatial coverage and species richness, designated as the ‘subtropical region’ (region 1) and the ‘southern region’ (region 2) (Figure 3). These regions exhibited strong cohesion and clear latitudinal separation, as evidenced by their separation into two distinct areas in the biogeographical network (Figure 3b). The southern region was characterised by species such as *Electrona antarctica* , *Gymnoscopelus braueri* and *Krefftichthys anderssoni* , typically associated with southern waters. In contrast, the subtropical region harboured species like *Ceratoscopelus warmingii* , *Lampanyctus australis* and *Lampichthys procerus* , known for their tropical or subtropical affiliations, or often found near the subtropical convergence/front (Duhamel et al. 2014). Species within these regions exhibited high fidelity (see Appendix S9), indicating infrequent occurrence in other regions and underscoring their distinct biogeographical identities.

Additional smaller clusters included region 3, with a handful of grid cells scattered across the map. Region 3 is positioned as a transition between the subtropical and southern regions on the network, with nodes connected to both regions 1 and 2 (Figure 3b). In other words, the five species from region 3 also occur in regions 1 and 2, which can be characterised numerically by their low fidelity values and widespread distribution (see Appendix S9). These species are likely to be found anywhere in the area, and especially at the transition between regions 1 and 2. Region 5 was distinguished by high species fidelity and a restricted distribution along the southwestern coast of South America. Region 4 consisted of only two species, reflecting their localised, rather coastal, distribution. The remaining six clusters, each comprising fewer than 20 nodes, were considered anecdotal.

The detailed species composition of each cluster is provided in Appendix S3.

### 3.2 A Climatic Boundary Between Biogeographical Regions

To analyse the climatic boundary between biogeographical regions, we focused our modelling efforts exclusively on the subtropical and southern regions (regions 1 and 2), which were the only regions for which meaningful region–environment relationships could be identified (i.e., high cross‐validation performance with high Jaccard indices > 0.7, see Appendix S8 for detailed model evaluations). Region 5 did not meet the minimum presence threshold of 30 required for model calibration, and we could not find meaningful relationships between the spatial limits of regions 3 and 4 and the environment (Jaccard indices < 0.5, Appendix S8).

**FIGURE 4:**
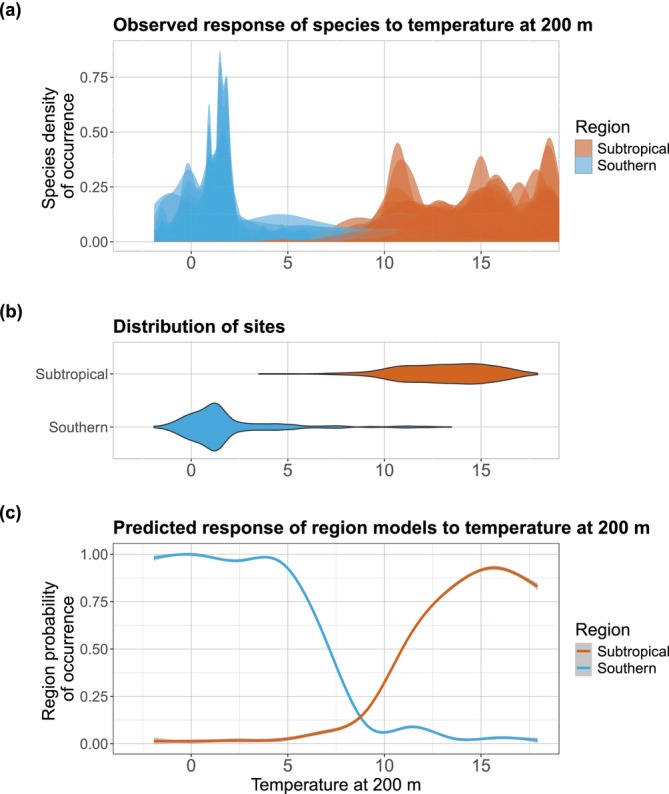
Observed distribution of species and sites and modelled distribution of biogeographical regions along the temperature gradient. (a) The density of species occurrence along the temperature gradient at 200 m depth. Each curve represents one myctophid species, with only species from the subtropical and southern regions included. (b) The distribution of sampling sites from the two regions along the temperature gradient. The violin plots illustrate the density of sites at each temperature value; the thicker the violin, the more abundant the sites. (c) The mean probability of occurrence for the two regions along the temperature gradient, as predicted by the models. The ribbon coloured in light grey shows the variability (i.e., standard deviation) in the individual response curves across the five region distribution models and the four cross‐validation folds.

Our examination of the distribution of species from regions 1 and 2 along the climatic gradient indicated a distinct separation based on temperature: southern species predominantly occupied waters below 5°C–8°C, whereas subtropical species were mainly found in waters above this range, with some overlap within the 5°C–8°C interval (Figure 4a). Species from the minor clusters fell between the two primary regions along the temperature gradient (see Appendix S10). Analysing site distributions also revealed a clear division (Figure 4b), with 90% of the southern sites occurring below 4.9°C and 90% of the subtropical sites above 10.2°C. No clear separation was observed for other environmental variables (see Appendix S11).

Further analysis of variable importance across all models consistently identified temperature at 200 m depth as the only significant predictor of region distribution (see Appendices S8 and S12). We have therefore retained only this variable to calibrate the final set of models. When modelling distributions based on temperature, we illustrated the mean response of each region to this variable (Figure 4c). The models predicted that temperature acts as a sharp climatic barrier between the two regions, approximately around 8°C–9°C.

### 3.3 Projected Impacts of Climate Change

**FIGURE 5:**
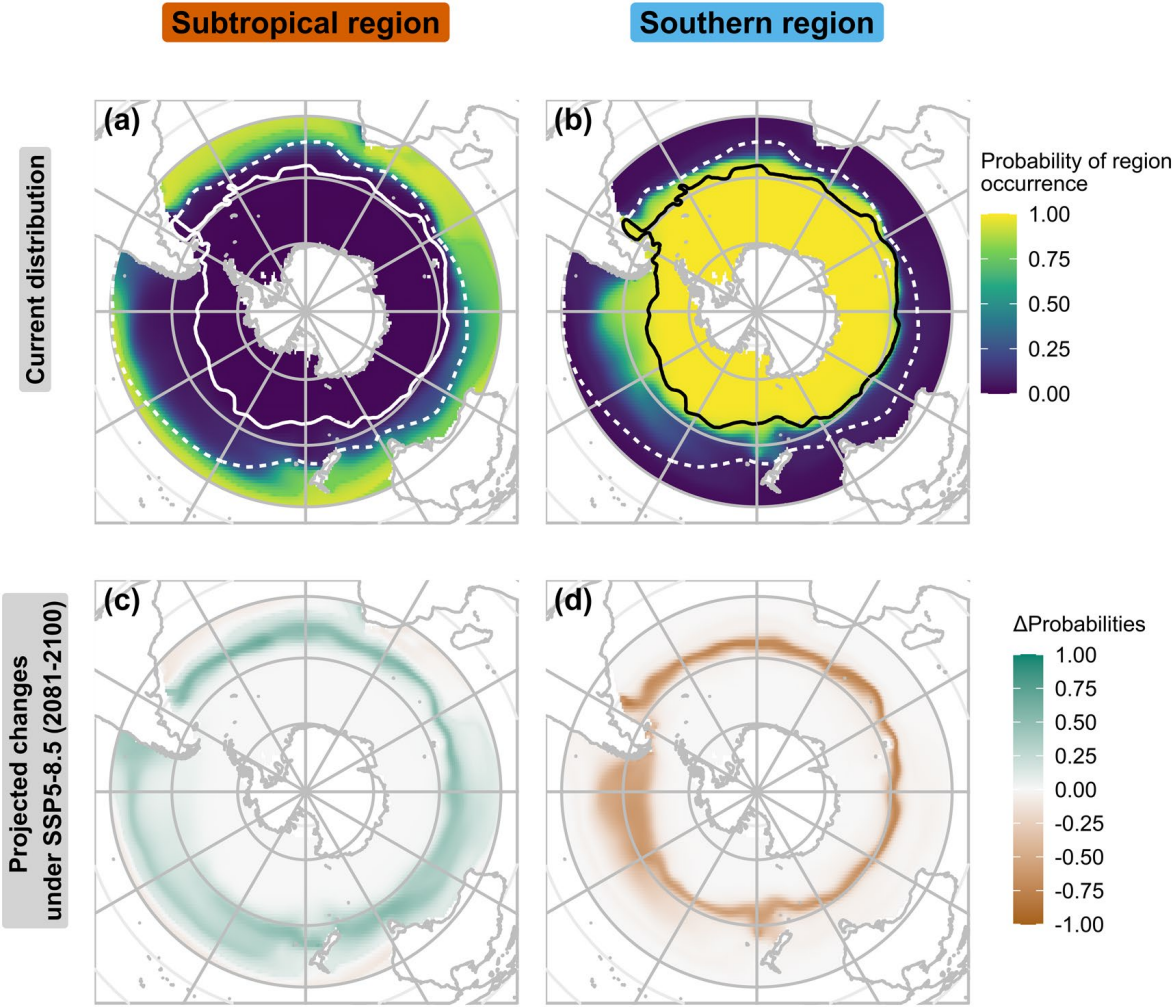
Current distribution and projected changes of the southern and subtropical biogeographical regions. The upper panels (a, b) display the current modelled probabilities of region occurrence, representing the distribution of biogeographical regions. The lower panels (c, d) illustrate the projected changes in the distributions, showing the differences between the historical model outputs and long‐term projections for 2081–2100 under the SSP5‐8.5 scenario. Negative values indicate potential loss of area, while positive values indicate potential gain. The current locations of the Subtropical Front (STF, dashed line) and the Subantarctic Front (SAF, solid line) are plotted for reference on the upper panels (from De Broyer et al. 2014).

We modelled the current distributions of the southern and subtropical regions according to temperature at 200 m, mapping their probability of occurrence over the study area (Figure 5a,b). The southern boundary of the subtropical region appeared to follow the STF, while the southern region was limited by the STF between South America and the Kerguelen Islands, then by the SAF between Kerguelen and southern New Zealand. Individual model projections and standard deviations among individual models showed only limited variability in the distribution of regions, specifically located in areas of intermediate probability at the boundary of regions (see Appendix S13).

**FIGURE 6:**
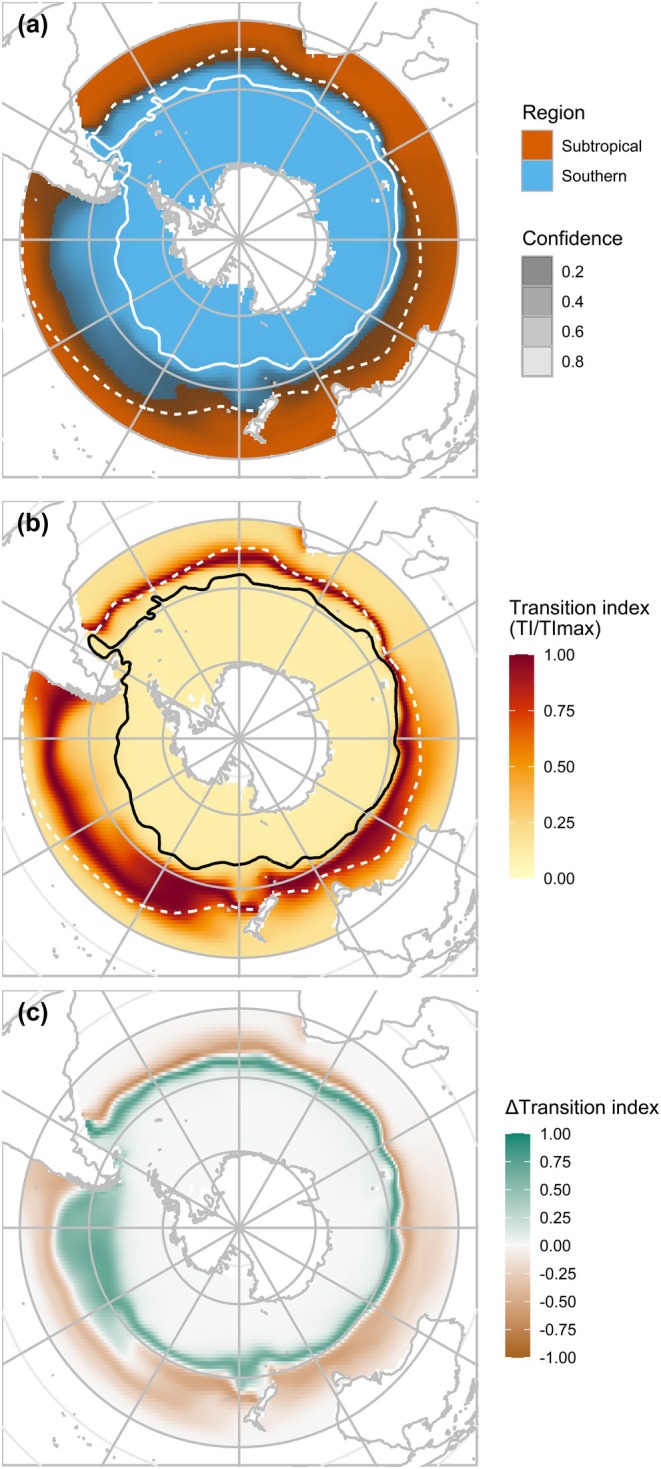
Spatial delineation of the subtropical and southern regions and current distribution and projected changes of the transition zone. (a) Following a hard classification approach, each cell is assigned to the region with the highest probability of occurrence. It is associated with a confidence value which corresponds to the probability value of the assigned region (i.e., if the probability is low, the confidence is low). (b) The current distribution of the transition zone is defined by the normalised Transition index, ranging from 0 to 1 and calculated for *N* = 2 regions. (c) The projected changes in the distribution of the transition zone correspond to the difference between the historical model outputs and long‐term projections for 2081–2100 under the SSP5‐8.5 scenario. Negative values indicate potential loss of area, whereas positive values indicate potential gain. The current locations of the Subtropical Front (STF, dashed line) and the Subantarctic Front (SAF, solid line) are plotted for reference on panels (a) and (b) (from De Broyer et al. 2014).

We combined model projections for both regions to predictively map their distribution across the entire study area with high confidence except in the precise location of the boundary (Figure 6a). This area of uncertainty regarding region boundaries coincided with the transition zone identified using the Transition index, indicating cells where both regions have equivalent probabilities of occurrence (Figure 6b). This transition zone matched the area between the STF and SAF. It was notably narrow in the Atlantic and Western Indian Oceans, especially near the Kerguelen plateau where both fronts are closely positioned, and wider in the Australian and Pacific regions where the fronts are more distant from each other.

**FIGURE 7:**
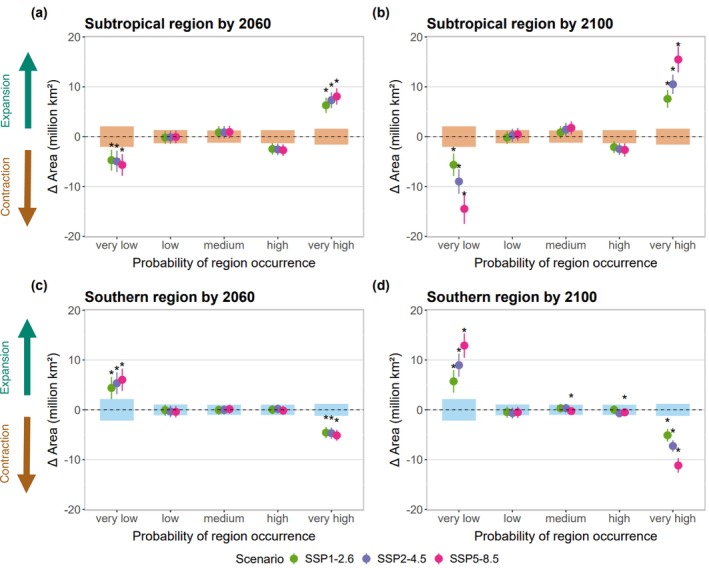
Predicted area difference between future and present probabilities of occurrence of biogeographical regions, for three scenarios and two time horizons. The plots display the changes in area for biogeographical regions based on five classes of occurrence probability: Very low [0; 0.2], low ]0.2;0.4], medium ]0.4;0.6], high ]0.6;0.8], very high ]0.8;1]. Panels (a) and (b) show the mid‐term (2041–2060) and long‐term (2081–2100) projections for the subtropical region, respectively, whereas panels (c) and (d) display the same for the southern region. Points are coloured according to three climate change scenarios: SSP1‐2.6, SSP2‐4.5 and SSP5‐8.5. Negative values indicate a loss of area (contraction), and positive values indicate a gain (expansion). For instance, an increase in areas of very high probability is expected for the subtropical region (a, b), whereas these areas are expected to decrease for the southern region (c, d). Point bars represent the standard deviation of future area predictions for each scenario, whereas blue bars indicate the standard deviation of the present modelled area. Asterisks (*) above points denote significant differences between future and present predictions, as determined by a paired t‐test or a sign test (*p*‐value < 0.05).

We projected future climate suitability of biogeographical regions under three climate change scenarios and two time horizons: mid‐term (2041–2060) and long‐term (2081–2100). Full projections are provided in Appendix S14, along with maps of standard deviation across all model runs. Our models projected a southward shift in areas favourable to the regions, with an expansion of the subtropical region and a contraction of the southern region (Figures 5a,c,d and 7). Considering areas with an occurrence probability higher than 0.8, we projected a gain of 15.5 million km^2^ for the subtropical region by 2100 under the SSP5‐8.5 scenario, and a loss of 11.2 million km^2^ for the southern region (Figure 7b,d). The change in occurrence probabilities was more pronounced in the Atlantic and Indian Oceans but more widespread in the Pacific Ocean. Moreover, the entire study area was projected to become more favourable to the subtropical region, whereas we expected the opposite for the southern region. The extent of these shifts was greatest for the SSP5‐8.5 scenario, followed by SSP2‐4.5 and SSP1‐2.6 (Figure 7).

Our models suggested a southward shift of the transition zone (Figure 6c), which was generally expected to shrink, except in the eastern Pacific Ocean. In this specific area, between 65° W and 130° W, the transition zone was projected to expand by 3.1 million km^2^, while we expected a contraction of 3.4 million km^2^ by 2100 in other regions, according to the SSP5‐8.5 scenario. Projections of the future distribution of the transition zone are provided in Appendix S14.

## 4 Discussion

We identified two large distinct biogeographical regions of lanternfishes spanning the Southern Ocean and adjacent subtropical areas up to 30° S: a southern region and a subtropical region. Our initial hypothesis of a climatic barrier between regions was corroborated by clear separations in species and site distributions along the temperature gradient, as well as by modelled responses to the temperature gradient. Based on projected temperature increases due to climate change, our models forecasted poleward shifts for both regions, as anticipated by the SROCC report (Meredith et al. 2019) and Reygondeau and Huettmann (2014). However, we found that the southern region is likely to shrink the most, with a loss of up to 11 million km^2^ in area, because it cannot expand due to the Antarctic continent acting as a cul‐de‐sac. We argue that this approach could be generalised to forecast the impacts of climate change on entire biogeographical regions—specifically those in which the distribution limits of species are jointly determined by shared climatic conditions.

### 4.1 Biogeographical Regions of Lanternfishes Between the Southern Ocean and the Surrounding Subtropical Areas Are Ecologically Meaningful

We identified 11 regions but focused on two large, circumglobal regions, latitudinally separated and characterised by unique species compositions over the historical period. These regions are composed of high‐fidelity species (i.e., species whose range is almost entirely located in their associated region), adapted to their respective thermal regimes, with minimal overlap between them. The species‐region assignment was consistent with the known biogeographical characteristics of species (Duhamel et al. 2014). The composition of the subtropical region includes tropical, subtropical, convergence and bi‐temperate species, whereas the southern region predominantly hosts Antarctic or subantarctic species, with the exception of one bi‐temperate species, *Lampanyctus macdonaldi* , which is also found at high latitudes in the Northern Hemisphere.

Both regions are found to be spatially cohesive, which is an expected feature of robust biogeographical regions (Divíšek et al. 2016; Victorero et al. 2023). This cohesiveness can be explained by two factors. First, there are nearly no hard barriers to dispersal (i.e., near absence of land masses) within both regions. Second, lanternfishes have great dispersal capabilities due to their life cycle taking place entirely in the water column, including early life, maturity and reproduction (Catul et al. 2011; Moteki et al. 2017).

An important barrier to dispersal of lanternfishes in the Southern Ocean appears to be located between the current subtropical front (STF) and subantarctic front (SAF), where the latitudinal boundary between both regions is situated (Figure 6a). This location was confirmed by our models, based on the temperature at 200 m, which predicted a transition zone (i.e., area where the regions are equally expected to co‐occur) following the variations in the location of these fronts. This corresponds to the long‐identified Subantarctic Zone, where the STF marks an abrupt separation with the warmer and saline subtropical waters, while the SAF marks the limit of the ACC (Deacon 1982; Orsi et al. 1995). The combination of abrupt environmental changes, notably in temperature, with strong currents of the ACC may explain why these fronts appear to be driving the boundaries between both regions.

The two large and circumpolar biogeographical regions we identified are consistent with other large‐scale studies based on biotic and abiotic data (Costello et al. 2017; Proud et al. 2017; Sutton et al. 2017). However, studies at a finer scale (i.e., smaller spatial extent and/or use of abundance data) have revealed more complex patterns in the Southern Ocean (Koubbi et al. 1991, 2010; Hoddell et al. 2000; Grant et al. 2006; Raymond 2011; Testa et al. 2021; Lowe et al. 2022), including for lanternfish regions in the Indian sector (Koubbi et al. 2011, 2023, 2024). These differences among spatial scales suggest a hierarchical structure in the bioregionalisation of the Southern Ocean, with the existence of small regions nested within the large regions we identified.

Region 3 is a transition region composed of five species that are the exception to the biogeographical structure of the area since they occur widely in both regions 1 and 2. These species were likely species that did not clearly belong in either region 1 or 2 due to their widespread distribution; hence, they were clustered into a distinct group by *Map Equation*, a behavior of the algorithm that has been shown previously (Vilhena and Antonelli 2015; Bloomfield et al. 2018). Yet, grid cells of region 3 were scarce, not spatially cohesive, and did not align with any oceanographic feature. This scattered distribution could be due to the very low richness of this transition region, preventing its identification in most of the transition area because of their co‐occurrence with numerous species of either region 1 or 2. Consequently, the composition of region 3 is ecologically meaningful, but its spatial extent is not. We consider that the spatial extent of the transition zone is better defined by our Transition index.

Regions 4 and 5, on the other hand, were spatially cohesive and may represent original biogeographical assemblages of the area. Their small size prevented us from successfully modelling them, but on the basis of their distribution, we can hypothesise that they represent coastal habitat locations (region 4) or local environmental conditions such as the upwelling of nutrient‐rich water (region 5).

### 4.2 Observations and Predictions Corroborate the Hypothesis of a Temperature Barrier

Our hypothesis of a climate barrier between biogeographical regions was supported by three key findings: (i) the gap between the thermal ranges of species along the temperature gradient, (ii) a similar gap in the distribution of sites and (iii) the modelled response curves of regions that confirmed a low probability of occurrence for both regions at 8.6°C (Figure 4). This separation of regions around 8°C aligns with the transition zone located between two fronts: the SAF, defined by isotherms ranging from 4°C to 8°C at 200 m, and the STF, ranging from 8°C to 12°C at 200 m (Park et al. 1993; Belkin and Gordon 1996). The temperature barrier hypothesis is particularly relevant for myctophids, as they are ectothermic organisms highly sensitive to temperature changes (Freer et al. 2019). Intriguingly, a thermal effect around 8°C has already been noted in many other species of teleost fish. For example, certain temperate species cease activity at or below 8°C (Costello et al. 1995, 2023), whereas polar species exhibit increased mortality above 8°C (York and Zakon 2022). In fact, the hypothesis of a critical physiological threshold at 8°C was proposed by Pörtner (2002), who suggested that teleosts experience a marked narrowing of their thermal tolerance window below this temperature. This requires the evolution of specialised adaptations to survive (e.g., metabolic cold adaptation, Watanabe and Payne 2023), ultimately resulting in narrower niches for polar species. In essence, this 8°C thermal threshold likely represents a boundary below which temperate‐adapted biochemical modulations no longer suffice, and further specialised adaptations are needed to cope with the cold. Metabolic cold adaptations include modified cell membrane fluidity, enzyme efficiency and oxygen transport mechanisms (Pörtner 2002; Beers and Jayasundara 2015), with such adaptations having been identified in lanternfishes specifically (e.g., Torres and Somero 1988). These cold adaptations, in turn, result in a range of consequences that render these species unable to cope with higher temperatures, such as the loss of inducible heat shock proteins (Beers and Jayasundara 2015). In sum, multiple physiological pathways exhibit tipping points at temperatures between ~5°C and 8°C, a critical range beyond which lanternfishes adapted to cold waters begin to experience significantly increased mortality rates. Thus, our finding of a climate barrier around 8°C aligns with existing hypotheses and seems to be corroborated by multiple lines of evidence, with the abrupt temperature changes between the SAF and STF likely further aggravating this effect.

While we highlight the role of temperature in distinguishing biogeographical regions, this remains only one dimension of the environmental influences driving species distributions. Although the STF and SAF are formed based on temperature shifts in water masses on either side of them, they also represent a physical barrier in terms of water mass movements in the form of current jets and eddies located between the two fronts in the ACC (Rintoul and da Silva 2019). While oceanographic fronts were not the focus of this study, we acknowledge the importance of their ecological role, for example, allowing fish to more easily move eastward within the ACC, compared to westward movements within the same area. The presence of the ACC also impedes the ability of fish to travel from north to south (or vice versa) across the ACC (Hunt et al. 2016). These purely physical constraints will also influence the distributions of species within and between the subtropical and southern regions. Finally, the area of the ACC itself, which corresponds to the transition region, exhibits unique physical features that distinguish it from the regions to the north and south (Hunt et al. 2016), with the action of eddies, upwelling and nutrient advection promoting primary production within the ACC (Strass et al. 2002).

Ultimately, climate change impacts on temperature and other environmental factors (e.g., salinity, water column structure) are predicted to influence the location and intensity of oceanic fronts, including those in the Southern Ocean (Chapman et al. 2020). This will result in a lessening or intensification of the physical barriers presented by the ACC, and the quality of the environment within its range, which may in turn influence species distributions over time. Future studies may consider integrating Lagrangian dispersal modelling into species distribution models in order to account for the additional function of current systems as physical barriers as well as temperature barriers to species distributions (Holloway and Miller 2017; Castro et al. 2020).

### 4.3 Climate Change Will Severely Impact Lanternfishes in the Southern Ocean

The temperature barrier hypothesis is supported by our observations and by evidence from the ecophysiological literature as explained in paragraph 4.2. For these reasons, we are confident that our projections due to temperature alone represent likely scenarios of future evolution under climate change. Our models projected a circumpolar poleward shift, consistent with the general responses of organisms to global warming, particularly myctophids (Parmesan 2006; Constable et al. 2014; Freer et al. 2019; Meredith et al. 2019; Liu et al. 2024; Zhai et al. 2024). Previous findings of poleward shifts have been contested because key oceanographic features, such as the ACC fronts, may remain stable in the future, unlike temperature, which is clearly shifting poleward (Meijers et al. 2019; Chapman et al. 2020). However, our projections are unlikely to be influenced by this concern because our results combined with ecophysiological evidence specifically suggest that temperature itself acts as a primary physiological barrier separating southern and subtropical species.

This projected shift will have different consequences for the two regions: the subtropical region is likely to expand, while the southern region is restricted to the south by the Antarctic continent, which acts as a cul‐de‐sac. In particular, there is potential for a collapse in lanternfish stocks in the southeast Pacific Ocean, where our models project a widespread contraction of the southern region combined with a limited probability of incursion of species from the subtropical region.

Comparing these projections to existing observations on range shifts in marine environments under climate change, we can make further assumptions on how these changes may actually take place. First, observations suggest that range shifts in marine environments generally follow closely the pace of climate change (Pinsky et al. 2013; Lenoir et al. 2020). Hence, changes are likely to occur within the projected time span (before the end of the century) and magnitude (a shift of up to 15 million km^2^ for the subtropical region in the worst‐case scenario, an area equivalent to the Antarctic continent). Second, range expansions generally outpace contractions, with trailing edges shifting more slowly than leading edges (Poloczanska et al. 2014; Lenoir et al. 2020). For Southern Ocean lanternfishes, this asymmetry may be amplified by the disparity between the projected increase in area for the subtropical region and the decrease for the southern region. If this holds true, it would result in greater overlap between regions in the future. This could potentially increase biotic interactions and accelerate the contraction of southern species due to competition. However, this hypothetical increase in overlap may be counterbalanced by the projected reduction of the transition zone, making the barrier effect even stronger in the future and reducing the likelihood of overlap.

This poleward shift may also have cascading consequences throughout the entire Southern Ocean ecosystem, especially regarding trophic relationships. It could alter predation pressure on their specific prey, as lanternfishes are not generalist predators (Shreeve et al. 2009; Saunders et al. 2015, 2018). Some prey may also undergo distribution shifts in response to climate change, such as Antarctic krill populations, which already exhibit declines and a poleward contraction (Atkinson et al. 2019). Predators of myctophids may also be impacted; some may follow them southwards at higher energetic costs (Péron et al. 2012; Constable et al. 2014; Bost et al. 2015), while others might shift to alternative prey, with the loss of energy‐rich myctophids potentially affecting population dynamics (Staniland et al. 2011; Saunders et al. 2019).

In addition to a taxonomic shift, we could expect a size shift in myctophid distributions, with smaller myctophids common in the subtropical area penetrating further south—as fishes in warmer waters tend to be smaller, complying with Bergmann's rule (Daufresne et al. 2009; Saunders and Tarling 2018; Freer et al. 2019). In addition, temperature change may directly alter size structuring through its effects on predator–prey relationships and size selectivity (Eskuche‐Keith et al. 2024).

Because vertically migrating myctophids contribute to the carbon cycle, a shift in their thermal niche could redistribute carbon export from surface waters to the deep ocean. This highlights the importance of considering shifts in Southern Ocean myctophid distributions in studying the change in the carbon pump under climate change (Henson et al. 2022).

Future research could further develop these hypotheses regarding the ecological consequences of myctophid region shifts by considering potential distribution shifts in trophically linked species. Pelagic ectothermic organisms may thus exhibit coordinated movements similar to myctophids, whereas endothermic predators such as mammals or birds that venture between the sea and land may react differently. Investigating temperature barriers and, if applicable, extending our bioregionalisation approach to other Southern Ocean species would contribute to our understanding of future distribution shifts under climate change.

### 4.4 Limitations

Modelling the distribution of biodiversity requires confronting the limits of our ability to accurately represent ecosystems. Calibrating models over a recent period of occurrence and temperature data, such as ours (since 1950), could pose the risk of overlooking potential variations linked to the response to direct anthropogenic pressures (Faurby and Araújo 2018) or other hidden covariates. This could result in an underestimation of the niche and an overestimation of range shifts, as species distributions might not be at equilibrium with temperature (Santini et al. 2021). At our study scale, we found a consistent separation between regions along the temperature gradient, suggesting that the observed distributions are indeed the result of overall temperature patterns. However, some uncertainty remains, as the calibration period coincides with a period of observed warming in the Southern Ocean since 1950, which may not have maintained stable temperature patterns throughout (Cai et al. 2023).

It should also be noted that the spatial extent of the study area was constrained to 30° S to the north and the physical limit of the Antarctic continent to the south. This constraint made it impossible to estimate the full ranges of thermal tolerance and hence the fundamental niche of lanternfish assemblages (Thuiller et al. 2004). Instead, we focused on the boundary between them, making projections on temperature ranges located on one tail of the response curves: the southern limit of the subtropical region and the northern limit of the southern region.

A major limitation that we could not address is the vertical dimension. We aggregated data from various fishing methods, which do not always provide stratified information, preventing us from accounting for the sampling depths of specimens in the analysis. Given the significant role of the STF and SAF in structuring regions and the evidence that these fronts penetrate the water column from the surface to the seafloor throughout much of their extent (Rintoul and da Silva 2019), it is likely that the vertical dimension would not significantly influence the projected shifts in regions that we identify.

There may be other responses to climate change in terms of phenology, physiology and behaviour not included in this study, which could influence the projected distribution shifts. For example, changes in the timing of life cycle events, physiological adaptations to new temperature regimes, and behavioural changes in response to altered prey availability and habitat conditions could all play a role in shaping future distributions (e.g., Desjonquères et al. 2022; Ponti and Sannolo 2023). Likewise, multiple factors beyond temperature affect species distributions, including other abiotic variables (oxygen, salinity), which were not detected as important at our scale of analysis, but may play a significant role at more localised scales. Additionally, we did not investigate other drivers such as biotic interactions (e.g., prey availability), dispersal and natural or anthropogenic disturbances because current knowledge shortfalls prevent us from doing so. Addressing these factors in future research would provide a more comprehensive understanding of how lanternfishes and other marine organisms will respond to ongoing climate change.

### 4.5 Perspectives in Conservation and Broader Applicability of This Approach

Estimating current and future species distributions provides crucial knowledge to optimise conservation policies in a changing climate. This aligns with the IPCC and IPBES calls for marine protected areas to be designed and managed considering future climate projections, enhancing climate resilience for biodiversity (IPBES 2019; IPCC 2022). This is particularly important for myctophids, which are projected to suffer habitat loss in the Southern Ocean and face increasing anthropogenic pressures, such as by‐catch in krill fisheries (Iwami et al. 2011) and potential direct targeting (Vipin et al. 2012). Such issues could be tackled in conservation policies by accounting for contrasting ecological scenarios in the design of protected area networks, along with dynamic management solutions, for example, incorporating areas predicted to remain stable under climate change or become potential refugia, as well as areas expected to undergo severe changes in species composition and interactions (particularly the transition zone). Mitigating other pressures, especially fishing, in these areas is crucial to support ecosystem resilience.

Studies of species distributions under climate change are particularly relevant to international conservation and management organisations, such as the Commission for the Conservation of Antarctic Marine Living Resources (CCAMLR). These organisations, in addition to managing fisheries, conduct programs of regionalisation and forecasting for spatial planning.

The conceptual approach we used in our study may have broader applicability in regions beyond the Southern Ocean, where oceanic fronts may also act as climatic barriers to dispersal. For example, many mesopelagic regions identified by Sutton et al. (2017) are latitudinally delimited by fronts, such as in the North Atlantic Ocean or the Subarctic Pacific Ocean. Similar situations are less common on land, where temperature is a less important driver of species distributions than in marine ecosystems (Sunday et al. 2012). In terrestrial ecosystems, climate is rarely the only barrier to dispersal, and complex habitat features (e.g., deserts, rainforests) often define these boundaries (Ficetola et al. 2017; Nekola et al. 2022).

Our findings supporting the climate barrier hypothesis offer significant implications for predictive biogeography. They lend credence to the application of the ‘group first, then predict’ approach for making future predictions under climate change scenarios. This suggests that responses and interactions within communities on either side of the climate barrier will remain stable in the future, meaning the current structure of assemblages will persist. However, this assumption relies on the idea that changes in the distribution of other interacting species from different trophic levels (zooplankton, larger predators) will not significantly disturb myctophid responses to climate change. Further research on the interactions between these groups and their collective responses to changing environmental conditions will then be crucial for developing accurate predictions. For instance, a promising approach relies on association distribution modelling (ADM) to model community distribution in space and time (Gaudin et al. 2024). Therefore, when support is found for the climate barrier hypothesis, community‐level models from a ‘group first, then predict’ strategy may succeed in predicting future assemblages, addressing issues identified in previous works (Ferrier and Guisan 2006; Baselga and Araújo 2010; Gros et al. 2022). This approach would provide valuable insights for designing effective conservation strategies to mitigate the impacts of climate change on biodiversity. Future research may investigate the applicability of this approach in other marine ecosystems, while also considering the complex interactions between species and their changing environments.

## Author Contributions

**Berta Ramiro‐Sánchez:** conceptualization, methodology, supervision, writing – review and editing. **Cédric Cotté:** conceptualization, data curation, writing – review and editing. **Clara Azarian:** data curation, methodology, software, writing – review and editing. **Jilda Alicia Caccavo:** conceptualization, writing – review and editing. **Eric Goberville:** conceptualization, methodology, writing – review and editing. **Fabien Leprieur:** conceptualization, methodology, writing – review and editing. **Claire Godet:** investigation, writing – review and editing. **Boris Leroy:** conceptualization, data curation, formal analysis, funding acquisition, investigation, methodology, project administration, resources, software, supervision, validation, visualization, writing – review and editing. **Marine Robuchon:** conceptualization, methodology, writing – review and editing. **Percy Alexander Hulley:** data curation, investigation, writing – review and editing. **Philippe Koubbi:** conceptualization, data curation, funding acquisition, investigation, project administration, resources, supervision, writing – review and editing. **Cam Ly Rintz:** conceptualization, data curation, formal analysis, investigation, methodology, software, visualization, writing – original draft, writing – review and editing. **Rémy Le Goff:** formal analysis, investigation, methodology, writing – review and editing. **Baptiste Serandour:** conceptualization, formal analysis, investigation, methodology, writing – review and editing.

## Conflicts of Interest

The authors declare no conflicts of interest.

## Supporting information


Appendix S1.

Appendix S4.

Appendix S2.
Appendix S3.
Appendix S5.
Appendix S6.
Appendix S7.
Appendix S8.
Appendix S9.
Appendix S10.
Appendix S11.
Appendix S12.
Appendix S13.
Appendix S14.

## Data Availability

The data and code that support the findings of this study are openly available in Zenodo at https://doi.org/10.5281/zenodo.15341993, https://doi.org/10.5281/zenodo.15295380, and https://doi.org/10.5281/zenodo.15306956. A list of the sources of occurrence and distribution data used in this study can be found in Appendix S1. Environmental data was obtained from the 2018 World Ocean Atlas at https://www.ncei.noaa.gov/archive/accession/NCEI‐WOA18. Bathymetry data was obtained from the SRTM30_PLUS dataset from the eAtlas at https://eatlas.org.au/data/uuid/80301676‐97fb‐4bdf‐b06c‐e961e5c0cb0b. The references of the publicly available outputs of CMIP6 models are provided in Appendix S6.
