# Supplementary material for: Biogeographical Regions and Climate Change: Lanternfishes Shed Light on the Role of Climatic Barriers in the Southern Ocean
Source: Glob Chang Biol. 2025 Jun 16;31(6):e70256. doi: 10.1111/gcb.70256 (PMC12168107; doi:10.1111/gcb.70256)
Supplement: Supplementary file 1 — Appendix S1. [file GCB-31-e70256-s003.pdf]

Appendix S1: Citations of datasets downloaded from GBIF and OBIS

05/05/2025

Contents

Electrona antarctica 5
Gymnoscopelus opisthopterus 6
Gymnoscopelus braueri 7
Gymnoscopelus nicholsi 9
Krefftichthys anderssoni 10
Protomyctophum bolini 11
Gymnoscopelus bolini 12
Lampanyctus achirus 14
Protomyctophum andriashevi 16
Lampanyctus macdonaldi 17
Electrona carlsbergi 20
Electrona subaspera 22
Protomyctophum tenisoni 23
Gymnoscopelus microlampas 24
Gymnoscopelus fraseri 25
Gymnoscopelus hintonoides 25
Protomyctophum choriodon 26
Protomyctophum parallelum 27
Protomyctophum normani 27
Protomyctophum gemmatum 28

Protomyctophum subparallelum 29
Gymnoscopelus piabilis 30
Electrona paucirastra 32
Metelectrona ventralis 32
Symbolophorus boops 33
Protomyctophum luciferum 35
Lampanyctus australis 36
Hintonia candens 38
Diaphus hudsoni 39
Lampadena speculigera 41
Lampichthys procerus 45
Lampanyctus intricarius 46
Ceratoscopelus warmingii 49
Diaphus ostenfeldi 54
Taaningichthys bathyphilus 55
Notoscopelus resplendens 58
Lampanyctus ater 63
Electrona risso 63
Bolinichthys supralateralis 67
Diaphus meadi 70
Diaphus mollis 72
Hygophum hanseni 75
Hygophum reinhardtii 76
Lobianchia dofleini 81
Myctophum phengodes 85
Scopelopsis multipunctatus 87
Symbolophorus barnardi 89
Lampanyctus pusillus 91

Symbolophorus evermanni . . . 95
Lampanyctus lepidolychnus . . . 97
Lampanyctus tenuiformis . . . 98
Gonichthys barnesi . . . 100
Lampanyctus alatus . . . 101
Benthosema suborbitale . . . 105
Diogenichthys atlanticus . . . 109
Hygophum hygomii . . . 113
Diaphus fragilis . . . 117
Lampadena luminosa . . . 119
Lampanyctus festivus . . . 121
Diaphus brachycephalus . . . 124
Lampadena chavesi . . . 126
Notolychnus valdiviae . . . 128
Notoscopelus caudispinosus . . . 132
Diaphus metopoclampus . . . 134
Lampadena dea . . . 136
Diaphus anderseni . . . 137
Diaphus perspicillatus . . . 139
Lobianchia gemellarii . . . 141
Bolinichthys longipes . . . 145
Bolinichthys indicus . . . 146
Diaphus danae . . . 149
Benthosema fibulatum . . . 150
Diaphus watasei . . . 152
Gonichthys cocco . . . 153
Myctophum nitidulum . . . 156
Myctophum affine . . . 161

Triphoturus nigrescens 163
Centrobranchus nigroocellatus 164
Lampanyctodes hectoris 167
Myctophum selenops 170
Diaphus effulgens 173
Lampanyctus nobilis 176
Myctophum spinosum 178
Lepidophanes guentheri 180
Diaphus garmani 184
Myctophum obtusirostre 186
Diaphus luetkeni 189
Lepidophanes gaussi 191
Myctophum asperum 194
Hygophum proximum 199
Diaphus dumerilii 201
Diogenichthys panurgus 205
Diaphus parri 206
Bolinichthys photothorax 208
Diaphus lucidus 210
Taaningichthys minimus 213
Diaphus termophilus 215
Diaphus mascarensis 217
Diaphus bertelseni 217
Loweina rara 219
Lampadena notialis 221
Diaphus diadematus 222
Lampanyctus turneri 223
Bolinichthys nikolayi 224

Diaphus coeruleus 225
Loweina interrupta 226
Lampanyctus phyllisae 226
Lampanyctus wisneri 227
Diaphus kapalae 227
Metelectrona herwigi 228
Hygophum bruuni 229
Triphoturus mexicanus 229
Protomyctophum chilense 230
Lampanyctus iselinoides 230
Metelectrona ahlstromi 230

Electrona antarctica

GBIF

GBIF Occurrence Download https://doi.org/10.15468/dd.ygrqdu Accessed from R via rgbif (https://github.com/ropensci/rgbif) on 2019-09-11

OBIS

OBIS (2019) Distribution records of Electrona antarctica [Dataset] (Available: Ocean Biodiversity Information System. Intergovernmental Oceanographic Commission of UNESCO. www.obis.org. Accessed: 2019-08-29)

Dataset details:

Dataset_ID	Name	Citation	License	OBIS_citation
25a2facf-bbfa-4b3a-b350-a9c2dc3edc3e	Biological data from the Soviet Antarctic Expedition (1955-1958)	SWPRON (2014): Biological data from the Soviet Antarctic Expedition (1955-1958). v1.1. Dataset/Occurrence. https://nzobisipt.niwa.co.nz/resource?r=soviet_ant_exp&v=1.1	This work is licensed under a Creative Commons Attribution (CC-BY) 4.0 License	OBIS (2019) [Biological data from the Soviet Antarctic Expedition (1955-1958)] (Available: Ocean Biodiversity Information System. Intergovernmental Oceanographic Commission of UNESCO. https://obis.org. Accessed: 2019-08-29)

49d1b62c-124a-4854-b878-b83de130eb23	International Polar Year and Census of Antarctic Marine Life Ross Sea voyage (TAN0802) biodiversity data	Ocean Survey 20/20 (2013). International Polar Year and Census of Antarctic Marine Life Ross Sea voyage (TAN0802) biodiversity data. Southwestern Pacific OBIS, National Institute of Water and Atmospheric Research, Wellington, New Zealand, 8748 records, Online http://nzobisipt.elasticbeanstalk.com/resource.do?r=mbis_caml released on Dec 12, 2013.	This work is licensed under a Creative Commons Attribution (CC-BY) 4.0 License	OBIS (2019) [International Polar Year and Census of Antarctic Marine Life Ross Sea voyage (TAN0802) biodiversity data] (Available: Ocean Biodiversity Information System. Intergovernmental Oceanographic Commission of UNESCO. https://obis.org. Accessed: 2019-08-29)
8a1ae661-e911-4967-bc06-1168fc5f2d89	iziko South African Museum - Fish Collection	iziko South African Museum - Fish Collection	Restricted	OBIS (2019) [iziko South African Museum - Fish Collection] (Available: Ocean Biodiversity Information System. Intergovernmental Oceanographic Commission of UNESCO. https://obis.org. Accessed: 2019-08-29)
b8617377-eb1c-4db2-baa6-8788a632e810	Ichthyology Collection - Royal Ontario Museum	NA	This work is licensed under a Creative Commons Attribution Non Commercial (CC-BY-NC) 4.0 License	OBIS (2019) [Ichthyology Collection - Royal Ontario Museum] (Available: Ocean Biodiversity Information System. Intergovernmental Oceanographic Commission of UNESCO. https://obis.org. Accessed: 2019-08-29)
d6d6fe4c-425f-4ce7-bf28-7a6befaeb413	National Museum of Natural History Vertebrate Zoology Fishes Collections	National Museum of Natural History, Smithsonian Institution NMNH Fishes Collection Database. National Museum of Natural History, Smithsonian Institution, 10th and Constitution Ave. N.W., Washington, DC 20560-0193, 2007.	This work is licensed under a Creative Commons Attribution (CC-BY) 4.0 License	OBIS (2019) [National Museum of Natural History Vertebrate Zoology Fishes Collections] (Available: Ocean Biodiversity Information System. Intergovernmental Oceanographic Commission of UNESCO. https://obis.org. Accessed: 2019-08-29)

Gymnoscopelus opisthopterus

GBIF

GBIF Occurrence Download https://doi.org/10.15468/dd.9rwn4d Accessed from R via rgbif (https://github.com/ropensci/rgbif) on 2019-09-11

OBIS

OBIS (2019) Distribution records of Gymnoscopelus opisthopterus [Dataset] (Available: Ocean Biodiversity Information System. Intergovernmental Oceanographic Commission of UNESCO. www.obis.org. Accessed: 2019-08-29)

Dataset details:

Dataset_ID	Name	Citation	License	OBIS_citation
25a2facf-bbfa-4b3a-b350-a9c2dc3edc3e	Biological data from the Soviet Antarctic Expedition (1955-1958)	SWPRON (2014): Biological data from the Soviet Antarctic Expedition (1955-1958). v1.1. Dataset/Occurrence. https://nzobisipt.niwa.co.nz/resource?r=soviet_ant_exp&v=1.1	This work is licensed under a Creative Commons Attribution (CC-BY) 4.0 License	OBIS (2019) [Biological data from the Soviet Antarctic Expedition (1955-1958)] (Available: Ocean Biodiversity Information System. Intergovernmental Oceanographic Commission of UNESCO. https://obis.org. Accessed: 2019-08-29)
49d1b62c-124a-4854-b878-b83de130eb23	International Polar Year and Census of Antarctic Marine Life Ross Sea voyage (TAN0802) biodiversity data	Ocean Survey 20/20 (2013). International Polar Year and Census of Antarctic Marine Life Ross Sea voyage (TAN0802) biodiversity data. Southwestern Pacific OBIS, National Institute of Water and Atmospheric Research, Wellington, New Zealand, 8748 records, Online http://nzobisipt.elasticbeanstalk.com/resource.do?r=mbis_caml released on Dec 12, 2013.	This work is licensed under a Creative Commons Attribution (CC-BY) 4.0 License	OBIS (2019) [International Polar Year and Census of Antarctic Marine Life Ross Sea voyage (TAN0802) biodiversity data] (Available: Ocean Biodiversity Information System. Intergovernmental Oceanographic Commission of UNESCO. https://obis.org. Accessed: 2019-08-29)

Gymnoscopelus braueri

GBIF

GBIF Occurrence Download https://doi.org/10.15468/dd.36u4p4 Accessed from R via rgbif (https://github.com/ropensci/rgbif) on 2019-09-11

OBIS

OBIS (2019) Distribution records of Gymnoscopelus braueri [Dataset] (Available: Ocean Biodiversity Information System. Intergovernmental Oceanographic Commission of UNESCO. www.obis.org. Accessed: 2019-08-29)

Dataset details:

Dataset_ID	Name	Citation	License	OBIS_citation

25a2facf-bbfa-4b3a-b350-a9c2dc3edc3e	Biological data from the Soviet Antarctic Expedition (1955-1958)	SWPRON (2014): Biological data from the Soviet Antarctic Expedition (1955-1958). v1.1. Dataset/Occurrence. https://nzobisipt.niwa.co.nz/resource?r=soviet_antt_exp&v=1.1	This work is licensed under a Creative Commons Attribution (CC-BY) 4.0 License	OBIS (2019) [Biological data from the Soviet Antarctic Expedition (1955-1958)] (Available: Ocean Biodiversity Information System. Intergovernmental Oceanographic Commission of UNESCO. https://obis.org. Accessed: 2019-08-29)
49d1b62c-124a-4854-b878-b83de130eb23	International Polar Year and Census of Antarctic Marine Life Ross Sea voyage (TAN0802) biodiversity data	Ocean Survey 20/20 (2013). International Polar Year and Census of Antarctic Marine Life Ross Sea voyage (TAN0802) biodiversity data. Southwestern Pacific OBIS, National Institute of Water and Atmospheric Research, Wellington, New Zealand, 8748 records, Online http://nzobisipt.elasticbeanstalk.com/resource.do?r=mbis_caml released on Dec 12, 2013.	This work is licensed under a Creative Commons Attribution (CC-BY) 4.0 License	OBIS (2019) [International Polar Year and Census of Antarctic Marine Life Ross Sea voyage (TAN0802) biodiversity data] (Available: Ocean Biodiversity Information System. Intergovernmental Oceanographic Commission of UNESCO. https://obis.org. Accessed: 2019-08-29)
8a1ae661-e911-4967-bc06-1168fc5f2d89	iziko South African Museum - Fish Collection	iziko South African Museum - Fish Collection	Restricted	OBIS (2019) [iziko South African Museum - Fish Collection] (Available: Ocean Biodiversity Information System. Intergovernmental Oceanographic Commission of UNESCO. https://obis.org. Accessed: 2019-08-29)
9ff216fc-777e-4f9b-9860-95ed7366870d	Institution SAIAB - Collection SAIAB	No available dataset citation	Unspecified intellectual rights	OBIS (2019) [Institution SAIAB - Collection SAIAB] (Available: Ocean Biodiversity Information System. Intergovernmental Oceanographic Commission of UNESCO. https://obis.org. Accessed: 2019-08-29)
b8617377-eb1c-4db2-baa6-8788a632e810	Ichthyology Collection - Royal Ontario Museum	NA	This work is licensed under a Creative Commons Attribution Non Commercial (CC-BY-NC) 4.0 License	OBIS (2019) [Ichthyology Collection - Royal Ontario Museum] (Available: Ocean Biodiversity Information System. Intergovernmental Oceanographic Commission of UNESCO. https://obis.org. Accessed: 2019-08-29)

d6d6fe4c-425f-4ce7-bf28-7a6befaeb413	National Museum of Natural History Vertebrate Zoology Fishes Collections	National Museum of Natural History, Smithsonian Institution NMNH Fishes Collection Database. National Museum of Natural History, Smithsonian Institution, 10th and Constitution Ave. N.W., Washington, DC 20560-0193, 2007.	This work is licensed under a Creative Commons Attribution (CC-BY) 4.0 License	OBIS (2019) [National Museum of Natural History Vertebrate Zoology Fishes Collections] (Available: Ocean Biodiversity Information System. Intergovernmental Oceanographic Commission of UNESCO. https://obis.org. Accessed: 2019-08-29)

Gymnoscopelus nicholsi

GBIF

GBIF Occurrence Download https://doi.org/10.15468/dd.qyzb3j Accessed from R via rgbif (https://github.com/ropensci/rgbif) on 2019-09-11

OBIS

OBIS (2019) Distribution records of Gymnoscopelus nicholsi [Dataset] (Available: Ocean Biodiversity Information System. Intergovernmental Oceanographic Commission of UNESCO. www.obis.org. Accessed: 2019-08-29)

Dataset details:

Dataset_ID	Name	Citation	License	OBIS_citation
25a2facf-bbfa-4b3a-b350-a9c2dc3edc3e	Biological data from the Soviet Antarctic Expedition (1955-1958)	SWPRON (2014): Biological data from the Soviet Antarctic Expedition (1955-1958). v1.1. Dataset/Occurrence. https://nzobisipt.niwa.co.nz/resource?r=soviet_ant_exp&v=1.1	This work is licensed under a Creative Commons Attribution (CC-BY) 4.0 License	OBIS (2019) [Biological data from the Soviet Antarctic Expedition (1955-1958)] (Available: Ocean Biodiversity Information System. Intergovernmental Oceanographic Commission of UNESCO. https://obis.org. Accessed: 2019-08-29)
49d1b62c-124a-4854-b878-b83de130eb23	International Polar Year and Census of Antarctic Marine Life Ross Sea voyage (TAN0802) biodiversity data	Ocean Survey 20/20 (2013). International Polar Year and Census of Antarctic Marine Life Ross Sea voyage (TAN0802) biodiversity data. Southwestern Pacific OBIS, National Institute of Water and Atmospheric Research, Wellington, New Zealand, 8748 records, Online http://nzobisipt.elasticbeanstalk.com/resource.do?r=mbis_caml released on Dec 12, 2013.	This work is licensed under a Creative Commons Attribution (CC-BY) 4.0 License	OBIS (2019) [International Polar Year and Census of Antarctic Marine Life Ross Sea voyage (TAN0802) biodiversity data] (Available: Ocean Biodiversity Information System. Intergovernmental Oceanographic Commission of UNESCO. https://obis.org. Accessed: 2019-08-29)

8a1ae661-e911-4967-bc06-1168fc5f2d89	iziko South African Museum - Fish Collection	iziko South African Museum - Fish Collection	Restricted	OBIS (2019) [iziko South African Museum - Fish Collection] (Available: Ocean Biodiversity Information System. Intergovernmental Oceanographic Commission of UNESCO. https://obis.org. Accessed: 2019-08-29)
b8617377-eb1c-4db2-baa6-8788a632e810	Ichthyology Collection - Royal Ontario Museum	NA	This work is licensed under a Creative Commons Attribution Non Commercial (CC-BY-NC) 4.0 License	OBIS (2019) [Ichthyology Collection - Royal Ontario Museum] (Available: Ocean Biodiversity Information System. Intergovernmental Oceanographic Commission of UNESCO. https://obis.org. Accessed: 2019-08-29)
d286ae50-ea29-4aa4-8028-2e6e5945a039	Institution REVIZEE - Collection Demersal Fishes	No available dataset citation	Unspecified intellectual rights	OBIS (2019) [Institution REVIZEE - Collection Demersal Fishes] (Available: Ocean Biodiversity Information System. Intergovernmental Oceanographic Commission of UNESCO. https://obis.org. Accessed: 2019-08-29)
d6d6fe4c-425f-4ce7-bf28-7a6befaeb413	National Museum of Natural History Vertebrate Zoology Fishes Collections	National Museum of Natural History, Smithsonian Institution NMNH Fishes Collection Database. National Museum of Natural History, Smithsonian Institution, 10th and Constitution Ave. N.W., Washington, DC 20560-0193, 2007.	This work is licensed under a Creative Commons Attribution (CC-BY) 4.0 License	OBIS (2019) [National Museum of Natural History Vertebrate Zoology Fishes Collections] (Available: Ocean Biodiversity Information System. Intergovernmental Oceanographic Commission of UNESCO. https://obis.org. Accessed: 2019-08-29)

Krefftichthys anderssoni

GBIF

GBIF Occurrence Download https://doi.org/10.15468/dd.f6bc6e Accessed from R via rgbif (https://github.com/ropensci/rgbif) on 2019-09-11

OBIS

OBIS (2019) Distribution records of Krefftichthys anderssoni [Dataset] (Available: Ocean Biodiversity Information System. Intergovernmental Oceanographic Commission of UNESCO. www.obis.org. Accessed: 2019-08-29)

Dataset details:

Dataset_ID	Name	Citation	License	OBIS_citation
25a2facf-bbfa-4b3a-b350-a9c2dc3edc3e	Biological data from the Soviet Antarctic Expedition (1955-1958)	SWPRON (2014): Biological data from the Soviet Antarctic Expedition (1955-1958). v1.1. Dataset/Occurrence. https://nzobisipt.niwa.co.nz/resource?r=soviet_ant_exp&v=1.1	This work is licensed under a Creative Commons Attribution (CC-BY) 4.0 License	OBIS (2019) [Biological data from the Soviet Antarctic Expedition (1955-1958)] (Available: Ocean Biodiversity Information System. Intergovernmental Oceanographic Commission of UNESCO. https://obis.org. Accessed: 2019-08-29)
49d1b62c-124a-4854-b878-b83de130eb23	International Polar Year and Census of Antarctic Marine Life Ross Sea voyage (TAN0802) biodiversity data	Ocean Survey 20/20 (2013). International Polar Year and Census of Antarctic Marine Life Ross Sea voyage (TAN0802) biodiversity data. Southwestern Pacific OBIS, National Institute of Water and Atmospheric Research, Wellington, New Zealand, 8748 records, Online http://nzobisipt.elasticbeanstalk.com/resource.do?r=mbis_caml released on Dec 12, 2013.	This work is licensed under a Creative Commons Attribution (CC-BY) 4.0 License	OBIS (2019) [International Polar Year and Census of Antarctic Marine Life Ross Sea voyage (TAN0802) biodiversity data] (Available: Ocean Biodiversity Information System. Intergovernmental Oceanographic Commission of UNESCO. https://obis.org. Accessed: 2019-08-29)
8a1ae661-e911-4967-bc06-1168fc5f2d89	iziko South African Museum - Fish Collection	iziko South African Museum - Fish Collection	Restricted	OBIS (2019) [iziko South African Museum - Fish Collection] (Available: Ocean Biodiversity Information System. Intergovernmental Oceanographic Commission of UNESCO. https://obis.org. Accessed: 2019-08-29)
b8617377-eb1c-4db2-baa6-8788a632e810	Ichthyology Collection - Royal Ontario Museum	NA	This work is licensed under a Creative Commons Attribution Non Commercial (CC-BY-NC) 4.0 License	OBIS (2019) [Ichthyology Collection - Royal Ontario Museum] (Available: Ocean Biodiversity Information System. Intergovernmental Oceanographic Commission of UNESCO. https://obis.org. Accessed: 2019-08-29)

Protomyctophum bolini

GBIF

GBIF Occurrence Download https://doi.org/10.15468/dd.krtbz7 Accessed from R via rgbif (https://github.com/ropensci/rgbif) on 2019-09-11

OBIS

OBIS (2019) Distribution records of Protomyctophum bolini [Dataset] (Available: Ocean Biodiversity Information System. Intergovernmental Oceanographic Commission of UNESCO. www.obis.org. Accessed: 2019-08-29)

Dataset details:

Dataset_ID	Name	Citation	License	OBIS_citation
25a2facf-bbfa-4b3a-b350-a9c2dc3edc3e	Biological data from the Soviet Antarctic Expedition (1955-1958)	SWPRON (2014): Biological data from the Soviet Antarctic Expedition (1955-1958). v1.1. Dataset/Occurrence. https://nzobisipt.niwa.co.nz/resource?r=soviet_ant_exp&v=1.1	This work is licensed under a Creative Commons Attribution (CC-BY) 4.0 License	OBIS (2019) [Biological data from the Soviet Antarctic Expedition (1955-1958)] (Available: Ocean Biodiversity Information System. Intergovernmental Oceanographic Commission of UNESCO. https://obis.org. Accessed: 2019-08-29)
8a1ae661-e911-4967-bc06-1168fc5f2d89	iziko South African Museum - Fish Collection	iziko South African Museum - Fish Collection	Restricted	OBIS (2019) [iziko South African Museum - Fish Collection] (Available: Ocean Biodiversity Information System. Intergovernmental Oceanographic Commission of UNESCO. https://obis.org. Accessed: 2019-08-29)
b8617377-eb1c-4db2-baa6-8788a632e810	Ichthyology Collection - Royal Ontario Museum	NA	This work is licensed under a Creative Commons Attribution Non Commercial (CC-BY-NC) 4.0 License	OBIS (2019) [Ichthyology Collection - Royal Ontario Museum] (Available: Ocean Biodiversity Information System. Intergovernmental Oceanographic Commission of UNESCO. https://obis.org. Accessed: 2019-08-29)
d6d6fe4c-425f-4ce7-bf28-7a6befaeb413	National Museum of Natural History Vertebrate Zoology Fishes Collections	National Museum of Natural History, Smithsonian Institution NMNH Fishes Collection Database. National Museum of Natural History, Smithsonian Institution, 10th and Constitution Ave. N.W., Washington, DC 20560-0193, 2007.	This work is licensed under a Creative Commons Attribution (CC-BY) 4.0 License	OBIS (2019) [National Museum of Natural History Vertebrate Zoology Fishes Collections] (Available: Ocean Biodiversity Information System. Intergovernmental Oceanographic Commission of UNESCO. https://obis.org. Accessed: 2019-08-29)

Gymnoscopelus bolini

GBIF

GBIF Occurrence Download https://doi.org/10.15468/dd.4gg5xw Accessed from R via rgbif (https://github.com/ropensci/rgbif) on 2019-09-11

OBIS

OBIS (2019) Distribution records of Gymnoscopelus bolini [Dataset] (Available: Ocean Biodiversity Information System. Intergovernmental Oceanographic Commission of UNESCO. www.obis.org. Accessed: 2019-08-29)

Dataset details:

Dataset_ID	Name	Citation	License	OBIS_citation
49d1b62c-124a-4854-b878-b83de130eb23	International Polar Year and Census of Antarctic Marine Life Ross Sea voyage (TAN0802) biodiversity data	Ocean Survey 20/20 (2013). International Polar Year and Census of Antarctic Marine Life Ross Sea voyage (TAN0802) biodiversity data. Southwestern Pacific OBIS, National Institute of Water and Atmospheric Research, Wellington, New Zealand, 8748 records, Online http://nzobisipt.elasticbeanstalk.com/resource.do?r=mbis_caml released on Dec 12, 2013.	This work is licensed under a Creative Commons Attribution (CC-BY) 4.0 License	OBIS (2019) [International Polar Year and Census of Antarctic Marine Life Ross Sea voyage (TAN0802) biodiversity data] (Available: Ocean Biodiversity Information System. Intergovernmental Oceanographic Commission of UNESCO. https://obis.org. Accessed: 2019-08-29)
705770e5-3474-4e69-be8b-3107a0c5610a	The fishes collection (IC) of the Muséum national d'Histoire naturelle (MNHN - Paris)	Gicim data base, Pruvost P. Causse R., 2009 http://doi.org/10.15468/tm7whu	This work is licensed under a Creative Commons Attribution (CC-BY) 4.0 License	OBIS (2019) [The fishes collection (IC) of the Muséum national d'Histoire naturelle (MNHN - Paris)] (Available: Ocean Biodiversity Information System. Intergovernmental Oceanographic Commission of UNESCO. https://obis.org. Accessed: 2019-08-29)
8a1ae661-e911-4967-bc06-1168fc5f2d89	iziko South African Museum - Fish Collection	iziko South African Museum - Fish Collection	Restricted	OBIS (2019) [iziko South African Museum - Fish Collection] (Available: Ocean Biodiversity Information System. Intergovernmental Oceanographic Commission of UNESCO. https://obis.org. Accessed: 2019-08-29)
9ff216fc-777e-4f9b-9860-95ed7366870d	Institution SAIAB - Collection SAIAB	No available dataset citation	Unspecified intellectual rights	OBIS (2019) [Institution SAIAB - Collection SAIAB] (Available: Ocean Biodiversity Information System. Intergovernmental Oceanographic Commission of UNESCO. https://obis.org. Accessed: 2019-08-29)
a4f7ee48-0d0b-4c05-a972-27a43b30db58	Institution MCM - Collection DEM	No available dataset citation	Unspecified intellectual rights	OBIS (2019) [Institution MCM - Collection DEM] (Available: Ocean Biodiversity Information System. Intergovernmental Oceanographic Commission of UNESCO. https://obis.org. Accessed: 2019-08-29)

d6d6fe4c-425f-4ce7-bf28-7a6befaeb413	National Museum of Natural History Vertebrate Zoology Fishes Collections	National Museum of Natural History, Smithsonian Institution NMNH Fishes Collection Database. National Museum of Natural History, Smithsonian Institution, 10th and Constitution Ave. N.W., Washington, DC 20560-0193, 2007.	This work is licensed under a Creative Commons Attribution (CC-BY) 4.0 License	OBIS (2019) [National Museum of Natural History Vertebrate Zoology Fishes Collections] (Available: Ocean Biodiversity Information System. Intergovernmental Oceanographic Commission of UNESCO. https://obis.org. Accessed: 2019-08-29)
f1da0955-5ece-4f98-ab77-f10c20bdd3ca	Institution OGL - Collection OGR	No available dataset citation	Unspecified intellectual rights	OBIS (2019) [Institution OGL - Collection OGR] (Available: Ocean Biodiversity Information System. Intergovernmental Oceanographic Commission of UNESCO. https://obis.org. Accessed: 2019-08-29)

Lampanyctus achirus

GBIF

GBIF Occurrence Download https://doi.org/10.15468/dd.pty8zu Accessed from R via rgbif (https://github.com/ropensci/rgbif) on 2019-09-11

OBIS

OBIS (2019) Distribution records of Lampanyctus achirus [Dataset] (Available: Ocean Biodiversity Information System. Intergovernmental Oceanographic Commission of UNESCO. www.obis.org. Accessed: 2019-08-29)

Dataset details:

Dataset_ID	Name	Citation	License	OBIS_citation
270f3e70-ff9b-411d-b170-2bc914d83f26	Biological Reference Collections ICM CSIC	Olivas González F J (2016): Biological Reference Collections ICM CSIC. Institute of Marine Sciences (ICM-CSIC). https://dx.doi.org/10.15470/qlqqdx	This work is licensed under a Creative Commons Attribution Non Commercial (CC-BY-NC) 4.0 License	OBIS (2019) [Biological Reference Collections ICM CSIC] (Available: Ocean Biodiversity Information System. Intergovernmental Oceanographic Commission of UNESCO. https://obis.org. Accessed: 2019-08-29)

2870c548-343e-4575-ac67-a4da35182c52	Institution Shirshov Institute - Collection SKAO	No available dataset citation	Unspecified intellectual rights	OBIS (2019) [Institution Shirshov Institute - Collection SKAO] (Available: Ocean Biodiversity Information System. Intergovernmental Oceanographic Commission of UNESCO. https://obis.org. Accessed: 2019-08-29)
49d1b62c-124a-4854-b878-b83de130eb23	International Polar Year and Census of Antarctic Marine Life Ross Sea voyage (TAN0802) biodiversity data	Ocean Survey 20/20 (2013). International Polar Year and Census of Antarctic Marine Life Ross Sea voyage (TAN0802) biodiversity data. Southwestern Pacific OBIS, National Institute of Water and Atmospheric Research, Wellington, New Zealand, 8748 records, Online http://nzobisipt.elasticbeanstalk.com/resource.do?r=mbis_caml released on Dec 12, 2013.	This work is licensed under a Creative Commons Attribution (CC-BY) 4.0 License	OBIS (2019) [International Polar Year and Census of Antarctic Marine Life Ross Sea voyage (TAN0802) biodiversity data] (Available: Ocean Biodiversity Information System. Intergovernmental Oceanographic Commission of UNESCO. https://obis.org. Accessed: 2019-08-29)
50903a57-ee9f-4367-b2cd-0b36dcf4a6ad	Catch data from New Zealand research trawls since 2008	SWPRON (2017). Catch data from New Zealand research trawls. Southwestern Pacific OBIS, National Institute of Water and Atmospheric Research (NIWA), Wellington, New Zealand, 15157 records, Online http://nzobisipt.niwa.co.nz/resource.do?r=trawl released on April 19, 2017.	This work is licensed under a Creative Commons Attribution (CC-BY) 4.0 License	OBIS (2019) [Catch data from New Zealand research trawls since 2008] (Available: Ocean Biodiversity Information System. Intergovernmental Oceanographic Commission of UNESCO. https://obis.org. Accessed: 2019-08-29)
8a1ae661-e911-4967-bc06-1168fc5f2d89	iziko South African Museum - Fish Collection	iziko South African Museum - Fish Collection	Restricted	OBIS (2019) [iziko South African Museum - Fish Collection] (Available: Ocean Biodiversity Information System. Intergovernmental Oceanographic Commission of UNESCO. https://obis.org. Accessed: 2019-08-29)
9ff216fc-777e-4f9b-9860-95ed7366870d	Institution SAIAB - Collection SAIAB	No available dataset citation	Unspecified intellectual rights	OBIS (2019) [Institution SAIAB - Collection SAIAB] (Available: Ocean Biodiversity Information System. Intergovernmental Oceanographic Commission of UNESCO. https://obis.org. Accessed: 2019-08-29)

b8617377-eb1c-4db2-baa6-8788a632e810	Ichthyology Collection - Royal Ontario Museum	NA	This work is licensed under a Creative Commons Attribution Non Commercial (CC-BY-NC) 4.0 License	OBIS (2019) [Ichthyology Collection - Royal Ontario Museum] (Available: Ocean Biodiversity Information System. Intergovernmental Oceanographic Commission of UNESCO. https://obis.org. Accessed: 2019-08-29)
d6d6fe4c-425f-4ce7-bf28-7a6befaeb413	National Museum of Natural History Vertebrate Zoology Fishes Collections	National Museum of Natural History, Smithsonian Institution NMNH Fishes Collection Database. National Museum of Natural History, Smithsonian Institution, 10th and Constitution Ave. N.W., Washington, DC 20560-0193, 2007.	This work is licensed under a Creative Commons Attribution (CC-BY) 4.0 License	OBIS (2019) [National Museum of Natural History Vertebrate Zoology Fishes Collections] (Available: Ocean Biodiversity Information System. Intergovernmental Oceanographic Commission of UNESCO. https://obis.org. Accessed: 2019-08-29)

Protomyctophum andriashevi

GBIF

GBIF Occurrence Download https://doi.org/10.15468/dd.mkjbf2 Accessed from R via rgbif (https://github.com/ropensci/rgbif) on 2019-09-11

OBIS

OBIS (2019) Distribution records of Protomyctophum andriashevi [Dataset] (Available: Ocean Biodiversity Information System. Intergovernmental Oceanographic Commission of UNESCO. www.obis.org. Accessed: 2019-08-29)

Dataset details:

Dataset_ID	Name	Citation	License	OBIS_citation
8a1ae661-e911-4967-bc06-1168fc5f2d89	iziko South African Museum - Fish Collection	iziko South African Museum - Fish Collection	Restricted	OBIS (2019) [iziko South African Museum - Fish Collection] (Available: Ocean Biodiversity Information System. Intergovernmental Oceanographic Commission of UNESCO. https://obis.org. Accessed: 2019-08-29)

d6d6fe4c-425f-4ce7-bf28-7a6befaeb413	National Museum of Natural History Vertebrate Zoology Fishes Collections	National Museum of Natural History, Smithsonian Institution NMNH Fishes Collection Database. National Museum of Natural History, Smithsonian Institution, 10th and Constitution Ave. N.W., Washington, DC 20560-0193, 2007.	This work is licensed under a Creative Commons Attribution (CC-BY) 4.0 License	OBIS (2019) [National Museum of Natural History Vertebrate Zoology Fishes Collections] (Available: Ocean Biodiversity Information System. Intergovernmental Oceanographic Commission of UNESCO. https://obis.org. Accessed: 2019-08-29)

Lampanyctus macdonaldi

GBIF

GBIF Occurrence Download https://doi.org/10.15468/dd.7k4z9n Accessed from R via rgbif (https://github.com/ropensci/rgbif) on 2019-09-11

OBIS

OBIS (2019) Distribution records of Lampanyctus macdonaldi [Dataset] (Available: Ocean Biodiversity Information System. Intergovernmental Oceanographic Commission of UNESCO. www.obis.org. Accessed: 2019-08-29)

Dataset details:

Dataset_ID	Name	Citation	License	OBIS_citation
1f59030f-f116-4c34-915e-1882d819cda3	Institution Southampton Oceanography Ceter - Collection discovery	No available dataset citation	Unspecified intellectual rights	OBIS (2019) [Institution Southampton Oceanography Ceter - Collection discovery] (Available: Ocean Biodiversity Information System. Intergovernmental Oceanographic Commission of UNESCO. https://obis.org. Accessed: 2019-08-29)
1febfcdd-3e1b-46db-b73d-1bbe81ce22a6	DFO Quebec Region MLI museum collection	Miller R, Nozères C (2025). DFO Quebec Region MLI museum collection. Version 3.19. Fisheries and Oceans Canada. Occurrence dataset. http://iobis.org/mapper/?resource_id=2673	This work is licensed under a Creative Commons Attribution (CC-BY) 4.0 License	OBIS (2019) [DFO Quebec Region MLI museum collection] (Available: Ocean Biodiversity Information System. Intergovernmental Oceanographic Commission of UNESCO. https://obis.org. Accessed: 2019-08-29)

26de959f-8916-43cf-9472-170c28358a8e	IMR Macroplankton surveys	Bakkeplass, K. (2014). IMR Macroplankton surveys. Institute of Marine Research, Norway	This work is licensed under a Creative Commons Attribution (CC-BY) 4.0 License	OBIS (2019) [IMR Macroplankton surveys] (Available: Ocean Biodiversity Information System. Intergovernmental Oceanographic Commission of UNESCO. https://obis.org. Accessed: 2019-08-29)
3d922162-062c-4ad2-bf4a-f2493bd3a95d	Institution Bedford Institute of Oceanography (BIO) - Collection SUMMER	No available dataset citation	Unspecified intellectual rights	OBIS (2019) [Institution Bedford Institute of Oceanography (BIO) - Collection SUMMER] (Available: Ocean Biodiversity Information System. Intergovernmental Oceanographic Commission of UNESCO. https://obis.org. Accessed: 2019-08-29)
5533fa1e-d1a6-47dc-b93e-bc51e2692589	MAR-ECO 2003 - Arni Fridriksson	Hafsteinn G. Gudfinnson, Høgni Debes, Tone Falkenhaug, Eilif Gaard, Ástthor Gislason, Hildur Petursdottir, Thorsteinn Sigurdsson, and Hedinn Valdimarsson. 2008. Abundance and productivity of the pelagic ecosystem along a transect across the northern Mid- Atlantic Ridge in June 2003. ICES CM 2008/C:12	This work is licensed under a Creative Commons Attribution (CC-BY) 4.0 License	OBIS (2019) [MAR-ECO 2003 - Arni Fridriksson] (Available: Ocean Biodiversity Information System. Intergovernmental Oceanographic Commission of UNESCO. https://obis.org. Accessed: 2019-08-29)
7e4228e5-a962-4b01-952f-7bf33e213a9c	BioChem: Sameoto zooplankton collection	Sameoto, D.D., Kennedy, M., Spry, J.S, Spry, J.M. (2013). Zooplankton datasets collected using the BIONESS sampler, ring nets and an Icelandic high speed sampler, 1967-2006. OBIS Canada Digital Collections. Published by OBIS http://www.iobis.org/. Accessed on –INSERT DATE	rights: http://data.gc.ca/eng/open-government-licence-canada & http://www.canadensys.net/norms rights holder: Her Majesty the Queen in right of Canada, as represented by the Minister of Fisheries and Oceans	OBIS (2019) [BioChem: Sameoto zooplankton collection] (Available: Ocean Biodiversity Information System. Intergovernmental Oceanographic Commission of UNESCO. https://obis.org. Accessed: 2019-08-29)
8629ec33-be4b-4384-933f-a511fbc29967	MAR-ECO 2004	Wenneck, T. de Lange, Falkenhaug, T. and O.A. Bergstad. 2008. Strategies, methods, and technologies adopted on the RV G.O. Sars MAR-ECO expedition to the mid-Atlantic Ridge in 2004. Deep-sea Research II. 55: 6-28.	This work is licensed under a Creative Commons Attribution (CC-BY) 4.0 License	OBIS (2019) [MAR-ECO 2004] (Available: Ocean Biodiversity Information System. Intergovernmental Oceanographic Commission of UNESCO. https://obis.org. Accessed: 2019-08-29)

8a1ae661-e911-4967-bc06-1168fc5f2d89	iziko South African Museum - Fish Collection	iziko South African Museum - Fish Collection	Restricted	OBIS (2019) [iziko South African Museum - Fish Collection] (Available: Ocean Biodiversity Information System. Intergovernmental Oceanographic Commission of UNESCO. https://obis.org. Accessed: 2019-08-29)
a4f7ee48-0d0b-4c05-a972-27a43b30db58	Institution MCM - Collection DEM	No available dataset citation	Unspecified intellectual rights	OBIS (2019) [Institution MCM - Collection DEM] (Available: Ocean Biodiversity Information System. Intergovernmental Oceanographic Commission of UNESCO. https://obis.org. Accessed: 2019-08-29)
b0a7add2-dd9e-4020-9ca4-5df048c8f6a2	Bigood	Fabri, M-C. et al., Ifremer BIOCEAN database (Deep Sea Benthic Fauna). Institut Français de Recherche pour l'Exploitation de la Mer, Ifremer, Issy-les-Moulineaux, France. World Wide Web electronic publication, http://www.ifremer.fr/isi/biocean	This work is licensed under a Creative Commons Attribution (CC-BY) 4.0 License	OBIS (2019) [Bigood] (Available: Ocean Biodiversity Information System. Intergovernmental Oceanographic Commission of UNESCO. https://obis.org. Accessed: 2019-08-29)
b8617377-eb1c-4db2-baa6-8788a632e810	Ichthyology Collection - Royal Ontario Museum	NA	This work is licensed under a Creative Commons Attribution Non Commercial (CC-BY-NC) 4.0 License	OBIS (2019) [Ichthyology Collection - Royal Ontario Museum] (Available: Ocean Biodiversity Information System. Intergovernmental Oceanographic Commission of UNESCO. https://obis.org. Accessed: 2019-08-29)
cc8f28ce-e48d-4945-abfe-9d150a22dcd6	Hamburg pelagic fish database	Post, A. 1987. Pelagic transects of FRVs "Walther Herwig" and "Anton Dohrn" in the Atlantic Ocean 1966 to 1986. Mitt. Inst. f. Seefischerei d. BfaFi Hamburg, 42: 1-68.	This work is licensed under a Creative Commons Attribution (CC-BY) 4.0 License	OBIS (2019) [Hamburg pelagic fish database] (Available: Ocean Biodiversity Information System. Intergovernmental Oceanographic Commission of UNESCO. https://obis.org. Accessed: 2019-08-29)
ce1d93f3-8b0f-4ee7-9a4d-0393a6ec7fea	Atlantic Reference Centre Museum of Canadian Atlantic Organisms - Invertebrates and Fishes Data	Van Guelpen, L., 2016. Atlantic Reference Centre Museum of Canadian Atlantic Organisms - Invertebrates and Fishes Data. Version 4 In OBIS Canada Digital Collections. Bedford Institute of Oceanography, Dartmouth, NS, Canada. Published by OBIS, Digital http://www.iobis.org/. Accessed on –INSERT DATE	This work is licensed under a Creative Commons Attribution (CC-BY) 4.0 License	OBIS (2019) [Atlantic Reference Centre Museum of Canadian Atlantic Organisms - Invertebrates and Fishes Data] (Available: Ocean Biodiversity Information System. Intergovernmental Oceanographic Commission of UNESCO. https://obis.org. Accessed: 2019-08-29)

cfc56587-48c3-4e3d-9350-3a4d9a28b681	Institution NOAA, NMFS, Northeast Fisheries Science Center - Collection DEEPWATER SYSTEMATICS	No available dataset citation	Unspecified intellectual rights	OBIS (2019) [Institution NOAA, NMFS, Northeast Fisheries Science Center - Collection DEEPWATER SYSTEMATICS] (Available: Ocean Biodiversity Information System. Intergovernmental Oceanographic Commission of UNESCO. https://obis.org. Accessed: 2019-08-29)
d6d6fe4c-425f-4ce7-bf28-7a6befaeb413	National Museum of Natural History Vertebrate Zoology Fishes Collections	National Museum of Natural History, Smithsonian Institution NMNH Fishes Collection Database. National Museum of Natural History, Smithsonian Institution, 10th and Constitution Ave. N.W., Washington, DC 20560-0193, 2007.	This work is licensed under a Creative Commons Attribution (CC-BY) 4.0 License	OBIS (2019) [National Museum of Natural History Vertebrate Zoology Fishes Collections] (Available: Ocean Biodiversity Information System. Intergovernmental Oceanographic Commission of UNESCO. https://obis.org. Accessed: 2019-08-29)
f7e3baa2-48c9-47d9-9219-0a457615d610	DFO Central and Arctic Multi-species Stock Assessment Surveys	DFO. (2016). Central and Arctic Multi-Species Stock Assessment Surveys Version 6 In OBIS Canada Digital Collections. Bedford Institute of Oceanography, Dartmouth, NS, Canada. Published by OBIS, Digital http://www.iobis.org/. Accessed on –INSERT DATE	|This work is licensed under a Creative Commons Attribution (CC-BY) 4.0 License	|OBIS (2019) [DFO Central and Arctic Multi-species Stock Assessment Surveys] (Available: Ocean Biodiversity Information System. Intergovernmental Oceanographic Commission of UNESCO. https://obis.org. Accessed: 2019-08-29)

Electrona carlsbergi

GBIF

GBIF Occurrence Download https://doi.org/10.15468/dd.ye9j7d Accessed from R via rgbif (https://github.com/ropensci/rgbif) on 2019-09-11

OBIS

OBIS (2019) Distribution records of Electrona carlsbergi [Dataset] (Available: Ocean Biodiversity Information System. Intergovernmental Oceanographic Commission of UNESCO. www.obis.org. Accessed: 2019-08-29)

Dataset details:

Dataset_ID	Name	Citation	License	OBIS_citation

25a2facf-bbfa-4b3a-b350-a9c2dc3edc3e	Biological data from the Soviet Antarctic Expedition (1955-1958)	SWPRON (2014): Biological data from the Soviet Antarctic Expedition (1955-1958). v1.1. Dataset/Occurrence. https://nzobisipt.niwa.co.nz/resource?r=soviet_ant_exp&v=1.1	This work is licensed under a Creative Commons Attribution (CC-BY) 4.0 License	OBIS (2019) [Biological data from the Soviet Antarctic Expedition (1955-1958)] (Available: Ocean Biodiversity Information System. Intergovernmental Oceanographic Commission of UNESCO. https://obis.org. Accessed: 2019-08-29)
49d1b62c-124a-4854-b878-b83de130eb23	International Polar Year and Census of Antarctic Marine Life Ross Sea voyage (TAN0802) biodiversity data	Ocean Survey 20/20 (2013). International Polar Year and Census of Antarctic Marine Life Ross Sea voyage (TAN0802) biodiversity data. Southwestern Pacific OBIS, National Institute of Water and Atmospheric Research, Wellington, New Zealand, 8748 records, Online http://nzobisipt.elasticbeanstalk.com/resource.do?r=mbis_caml released on Dec 12, 2013.	This work is licensed under a Creative Commons Attribution (CC-BY) 4.0 License	OBIS (2019) [International Polar Year and Census of Antarctic Marine Life Ross Sea voyage (TAN0802) biodiversity data] (Available: Ocean Biodiversity Information System. Intergovernmental Oceanographic Commission of UNESCO. https://obis.org. Accessed: 2019-08-29)
50903a57-ee9f-4367-b2cd-0b36dcf4a6ad	Catch data from New Zealand research trawls since 2008	SWPRON (2017). Catch data from New Zealand research trawls. Southwestern Pacific OBIS, National Institute of Water and Atmospheric Research (NIWA), Wellington, New Zealand, 15157 records, Online http://nzobisipt.niwa.co.nz/resource.do?r=trawl released on April 19, 2017.	This work is licensed under a Creative Commons Attribution (CC-BY) 4.0 License	OBIS (2019) [Catch data from New Zealand research trawls since 2008] (Available: Ocean Biodiversity Information System. Intergovernmental Oceanographic Commission of UNESCO. https://obis.org. Accessed: 2019-08-29)
685b3956-c37a-433a-b661-2bb7b11cf9f8	Soviet Trawl Fishery Data (New Zealand Waters) 1964-1987	Ministry for Primary Industries (2014). Soviet Fishery Data (New Zealand Waters) 1964-1987. Southwestern Pacific OBIS, National Institute of Water and Atmospheric Research (NIWA), Wellington, New Zealand, 111883 records, Online http://nzobisipt.niwa.co.nz/resource.do?r=mbis_soviettrawl released on November 5, 2014.	This work is licensed under a Creative Commons Attribution (CC-BY) 4.0 License	OBIS (2019) [Soviet Trawl Fishery Data (New Zealand Waters) 1964-1987] (Available: Ocean Biodiversity Information System. Intergovernmental Oceanographic Commission of UNESCO. https://obis.org. Accessed: 2019-08-29)
8a1ae661-e911-4967-bc06-1168fc5f2d89	iziko South African Museum - Fish Collection	iziko South African Museum - Fish Collection	Restricted	OBIS (2019) [iziko South African Museum - Fish Collection] (Available: Ocean Biodiversity Information System. Intergovernmental Oceanographic Commission of UNESCO. https://obis.org. Accessed: 2019-08-29)

d6d6fe4c-425f-4ce7-bf28-7a6befaeb413	National Museum of Natural History Vertebrate Zoology Fishes Collections	National Museum of Natural History, Smithsonian Institution NMNH Fishes Collection Database. National Museum of Natural History, Smithsonian Institution, 10th and Constitution Ave. N.W., Washington, DC 20560-0193, 2007.	This work is licensed under a Creative Commons Attribution (CC-BY) 4.0 License	OBIS (2019) [National Museum of Natural History Vertebrate Zoology Fishes Collections] (Available: Ocean Biodiversity Information System. Intergovernmental Oceanographic Commission of UNESCO. https://obis.org. Accessed: 2019-08-29)

Electrona subaspera

GBIF

GBIF Occurrence Download https://doi.org/10.15468/dd.s74ye7 Accessed from R via rgbif (https://github.com/ropensci/rgbif) on 2019-09-11

OBIS

OBIS (2019) Distribution records of Electrona subaspera [Dataset] (Available: Ocean Biodiversity Information System. Intergovernmental Oceanographic Commission of UNESCO. www.obis.org. Accessed: 2019-08-29)

Dataset details:

Dataset_ID	Name	Citation	License	OBIS_citation
685b3956-c37a-433a-b661-2bb7b11cf9f8	Soviet Trawl Fishery Data (New Zealand Waters) 1964-1987	Ministry for Primary Industries (2014). Soviet Fishery Data (New Zealand Waters) 1964-1987. Southwestern Pacific OBIS, National Institute of Water and Atmospheric Research (NIWA), Wellington, New Zealand, 111883 records, Online http://nzobisipt.niwa.co.nz/resource.do?r=mbis_soviettrawl released on November 5, 2014.	This work is licensed under a Creative Commons Attribution (CC-BY) 4.0 License	OBIS (2019) [Soviet Trawl Fishery Data (New Zealand Waters) 1964-1987] (Available: Ocean Biodiversity Information System. Intergovernmental Oceanographic Commission of UNESCO. https://obis.org. Accessed: 2019-08-29)
8a1ae661-e911-4967-bc06-1168fc5f2d89	iziko South African Museum - Fish Collection	iziko South African Museum - Fish Collection	Restricted	OBIS (2019) [iziko South African Museum - Fish Collection] (Available: Ocean Biodiversity Information System. Intergovernmental Oceanographic Commission of UNESCO. https://obis.org. Accessed: 2019-08-29)

9ff216fc-777e-4f9b-9860-95ed7366870d	Institution SAIAB - Collection SAIAB	No available dataset citation	Unspecified intellectual rights	OBIS (2019) [Institution SAIAB - Collection SAIAB] (Available: Ocean Biodiversity Information System. Intergovernmental Oceanographic Commission of UNESCO. https://obis.org. Accessed: 2019-08-29)

Protomyctophum tenisoni

GBIF

GBIF Occurrence Download https://doi.org/10.15468/dd.t8x4pm Accessed from R via rgbif (https://github.com/ropensci/rgbif) on 2019-09-11

OBIS

OBIS (2019) Distribution records of Protomyctophum tenisoni [Dataset] (Available: Ocean Biodiversity Information System. Intergovernmental Oceanographic Commission of UNESCO. www.obis.org. Accessed: 2019-08-29)

Dataset details:

Dataset_ID	Name	Citation	License	OBIS_citation
685b3956-c37a-433a-b661-2bb7b11cf9f8	Soviet Trawl Fishery Data (New Zealand Waters) 1964-1987	Ministry for Primary Industries (2014). Soviet Fishery Data (New Zealand Waters) 1964-1987. Southwestern Pacific OBIS, National Institute of Water and Atmospheric Research (NIWA), Wellington, New Zealand, 111883 records, Online http://nzobisipt.niwa.co.nz/resource.do?r=mbis_soviettrawl released on November 5, 2014.	This work is licensed under a Creative Commons Attribution (CC-BY) 4.0 License	OBIS (2019) [Soviet Trawl Fishery Data (New Zealand Waters) 1964-1987] (Available: Ocean Biodiversity Information System. Intergovernmental Oceanographic Commission of UNESCO. https://obis.org. Accessed: 2019-08-29)
8a1ae661-e911-4967-bc06-1168fc5f2d89	iziko South African Museum - Fish Collection	iziko South African Museum - Fish Collection	Restricted	OBIS (2019) [iziko South African Museum - Fish Collection] (Available: Ocean Biodiversity Information System. Intergovernmental Oceanographic Commission of UNESCO. https://obis.org. Accessed: 2019-08-29)

b8617377-eb1c-4db2-baa6-8788a632e810	Ichthyology Collection - Royal Ontario Museum	NA	This work is licensed under a Creative Commons Attribution Non Commercial (CC-BY-NC) 4.0 License	OBIS (2019) [Ichthyology Collection - Royal Ontario Museum] (Available: Ocean Biodiversity Information System. Intergovernmental Oceanographic Commission of UNESCO. https://obis.org. Accessed: 2019-08-29)
d6d6fe4c-425f-4ce7-bf28-7a6befaeb413	National Museum of Natural History Vertebrate Zoology Fishes Collections	National Museum of Natural History, Smithsonian Institution NMNH Fishes Collection Database. National Museum of Natural History, Smithsonian Institution, 10th and Constitution Ave. N.W., Washington, DC 20560-0193, 2007.	This work is licensed under a Creative Commons Attribution (CC-BY) 4.0 License	OBIS (2019) [National Museum of Natural History Vertebrate Zoology Fishes Collections] (Available: Ocean Biodiversity Information System. Intergovernmental Oceanographic Commission of UNESCO. https://obis.org. Accessed: 2019-08-29)

Gymnoscopelus microlampas

GBIF

GBIF Occurrence Download https://doi.org/10.15468/dd.nkp4mg Accessed from R via rgbif (https://github.com/ropensci/rgbif) on 2019-09-11

OBIS

OBIS (2019) Distribution records of Gymnoscopelus microlampas [Dataset] (Available: Ocean Biodiversity Information System. Intergovernmental Oceanographic Commission of UNESCO. www.obis.org. Accessed: 2019-08-29)

Dataset details:

Dataset_ID	Name	Citation	License	OBIS_citation
8a1ae661-e911-4967-bc06-1168fc5f2d89	iziko South African Museum - Fish Collection	iziko South African Museum - Fish Collection	Restricted	OBIS (2019) [iziko South African Museum - Fish Collection] (Available: Ocean Biodiversity Information System. Intergovernmental Oceanographic Commission of UNESCO. https://obis.org. Accessed: 2019-08-29)

Gymnoscopelus fraseri

GBIF

GBIF Occurrence Download https://doi.org/10.15468/dd.sq7zac Accessed from R via rgbif (https://github.com/ropensci/rgbif) on 2019-09-11

OBIS

OBIS (2019) Distribution records of Gymnoscopelus fraseri [Dataset] (Available: Ocean Biodiversity Information System. Intergovernmental Oceanographic Commission of UNESCO. www.obis.org. Accessed: 2019-08-29)

Dataset details:

Dataset_ID	Name	Citation	License	OBIS_citation
8a1ae661-e911-4967-bc06-1168fc5f2d89	iziko South African Museum - Fish Collection	iziko South African Museum - Fish Collection	Restricted	OBIS (2019) [iziko South African Museum - Fish Collection] (Available: Ocean Biodiversity Information System. Intergovernmental Oceanographic Commission of UNESCO. https://obis.org. Accessed: 2019-08-29)
b8617377-eb1c-4db2-baa6-8788a632e810	Ichthyology Collection - Royal Ontario Museum	NA	This work is licensed under a Creative Commons Attribution Non Commercial (CC-BY-NC) 4.0 License	OBIS (2019) [Ichthyology Collection - Royal Ontario Museum] (Available: Ocean Biodiversity Information System. Intergovernmental Oceanographic Commission of UNESCO. https://obis.org. Accessed: 2019-08-29)
d6d6fe4c-425f-4ce7-bf28-7a6befaeb413	National Museum of Natural History Vertebrate Zoology Fishes Collections	National Museum of Natural History, Smithsonian Institution NMNH Fishes Collection Database. National Museum of Natural History, Smithsonian Institution, 10th and Constitution Ave. N.W., Washington, DC 20560-0193, 2007.	This work is licensed under a Creative Commons Attribution (CC-BY) 4.0 License	OBIS (2019) [National Museum of Natural History Vertebrate Zoology Fishes Collections] (Available: Ocean Biodiversity Information System. Intergovernmental Oceanographic Commission of UNESCO. https://obis.org. Accessed: 2019-08-29)

Gymnoscopelus hintonoides

GBIF

GBIF Occurrence Download https://doi.org/10.15468/dd.9kgqv8 Accessed from R via rgbif (https://github.com/ropensci/rgbif) on 2019-09-11

OBIS

OBIS (2019) Distribution records of Gymnoscopelus hintonoides [Dataset] (Available: Ocean Biodiversity Information System. Intergovernmental Oceanographic Commission of UNESCO. www.obis.org. Accessed: 2019-08-29)

Dataset details:

Dataset_ID	Name	Citation	License	OBIS_citation
49d1b62c-124a-4854-b878-b83de130eb23	International Polar Year and Census of Antarctic Marine Life Ross Sea voyage (TAN0802) biodiversity data	Ocean Survey 20/20 (2013). International Polar Year and Census of Antarctic Marine Life Ross Sea voyage (TAN0802) biodiversity data. Southwestern Pacific OBIS, National Institute of Water and Atmospheric Research, Wellington, New Zealand, 8748 records, Online http://nzobisipt.elasticbeanstalk.com/resource.do?r=mbis_caml released on Dec 12, 2013.	This work is licensed under a Creative Commons Attribution (CC-BY) 4.0 License	OBIS (2019) [International Polar Year and Census of Antarctic Marine Life Ross Sea voyage (TAN0802) biodiversity data] (Available: Ocean Biodiversity Information System. Intergovernmental Oceanographic Commission of UNESCO. https://obis.org. Accessed: 2019-08-29)

Protomyctophum choriodon

GBIF

GBIF Occurrence Download https://doi.org/10.15468/dd.6669cz Accessed from R via rgbif (https://github.com/ropensci/rgbif) on 2019-09-11

OBIS

OBIS (2019) Distribution records of Protomyctophum choriodon [Dataset] (Available: Ocean Biodiversity Information System. Intergovernmental Oceanographic Commission of UNESCO. www.obis.org. Accessed: 2019-08-29)

Dataset details:

Dataset_ID	Name	Citation	License	OBIS_citation
8a1ae661-e911-4967-bc06-1168fc5f2d89	iziko South African Museum - Fish Collection	iziko South African Museum - Fish Collection	Restricted	OBIS (2019) [iziko South African Museum - Fish Collection] (Available: Ocean Biodiversity Information System. Intergovernmental Oceanographic Commission of UNESCO. https://obis.org. Accessed: 2019-08-29)

Protomyctophum parallelum

GBIF

GBIF Occurrence Download https://doi.org/10.15468/dd.4zhcq9 Accessed from R via rgbif (https://github.com/ropensci/rgbif) on 2019-09-11

OBIS

OBIS (2019) Distribution records of Protomyctophum parallelum [Dataset] (Available: Ocean Biodiversity Information System. Intergovernmental Oceanographic Commission of UNESCO. www.obis.org. Accessed: 2019-08-29)

Dataset details:

Dataset_ID	Name	Citation	License	OBIS_citation
8a1ae661-e911-4967-bc06-1168fc5f2d89	iziko South African Museum - Fish Collection	iziko South African Museum - Fish Collection	Restricted	OBIS (2019) [iziko South African Museum - Fish Collection] (Available: Ocean Biodiversity Information System. Intergovernmental Oceanographic Commission of UNESCO. https://obis.org. Accessed: 2019-08-29)
d6d6fe4c-425f-4ce7-bf28-7a6befaeb413	National Museum of Natural History Vertebrate Zoology Fishes Collections	National Museum of Natural History, Smithsonian Institution NMNH Fishes Collection Database. National Museum of Natural History, Smithsonian Institution, 10th and Constitution Ave. N.W., Washington, DC 20560-0193, 2007.	This work is licensed under a Creative Commons Attribution (CC-BY) 4.0 License	OBIS (2019) [National Museum of Natural History Vertebrate Zoology Fishes Collections] (Available: Ocean Biodiversity Information System. Intergovernmental Oceanographic Commission of UNESCO. https://obis.org. Accessed: 2019-08-29)

Protomyctophum normani

GBIF

GBIF Occurrence Download https://doi.org/10.15468/dd.3yqbgd Accessed from R via rgbif (https://github.com/ropensci/rgbif) on 2019-09-11

OBIS

OBIS (2019) Distribution records of Protomyctophum normani [Dataset] (Available: Ocean Biodiversity Information System. Intergovernmental Oceanographic Commission of UNESCO. www.obis.org. Accessed: 2019-08-29)

Dataset details:

Dataset_ID	Name	Citation	License	OBIS_citation
685b3956-c37a-433a-b661-2bb7b11cf9f8	Soviet Trawl Fishery Data (New Zealand Waters) 1964-1987	Ministry for Primary Industries (2014). Soviet Fishery Data (New Zealand Waters) 1964-1987. Southwestern Pacific OBIS, National Institute of Water and Atmospheric Research (NIWA), Wellington, New Zealand, 111883 records, Online http://nzobisipt.niwa.co.nz/resource.do?r=mbis_soviettrawl released on November 5, 2014.	This work is licensed under a Creative Commons Attribution (CC-BY) 4.0 License	OBIS (2019) [Soviet Trawl Fishery Data (New Zealand Waters) 1964-1987] (Available: Ocean Biodiversity Information System. Intergovernmental Oceanographic Commission of UNESCO. https://obis.org. Accessed: 2019-08-29)
8a1ae661-e911-4967-bc06-1168fc5f2d89	iziko South African Museum - Fish Collection	iziko South African Museum - Fish Collection	Restricted	OBIS (2019) [iziko South African Museum - Fish Collection] (Available: Ocean Biodiversity Information System. Intergovernmental Oceanographic Commission of UNESCO. https://obis.org. Accessed: 2019-08-29)
b8617377-eb1c-4db2-baa6-8788a632e810	Ichthyology Collection - Royal Ontario Museum	NA	This work is licensed under a Creative Commons Attribution Non Commercial (CC-BY-NC) 4.0 License	OBIS (2019) [Ichthyology Collection - Royal Ontario Museum] (Available: Ocean Biodiversity Information System. Intergovernmental Oceanographic Commission of UNESCO. https://obis.org. Accessed: 2019-08-29)
d6d6fe4c-425f-4ce7-bf28-7a6befaeb413	National Museum of Natural History Vertebrate Zoology Fishes Collections	National Museum of Natural History, Smithsonian Institution NMNH Fishes Collection Database. National Museum of Natural History, Smithsonian Institution, 10th and Constitution Ave. N.W., Washington, DC 20560-0193, 2007.	This work is licensed under a Creative Commons Attribution (CC-BY) 4.0 License	OBIS (2019) [National Museum of Natural History Vertebrate Zoology Fishes Collections] (Available: Ocean Biodiversity Information System. Intergovernmental Oceanographic Commission of UNESCO. https://obis.org. Accessed: 2019-08-29)

Protomyctophum gemmatum

GBIF

GBIF Occurrence Download https://doi.org/10.15468/dd.bqx3vr Accessed from R via rgbif (https://github.com/ropensci/rgbif) on 2019-09-11

OBIS

OBIS (2019) Distribution records of Protomyctophum gemmatum [Dataset] (Available: Ocean Biodiversity Information System. Intergovernmental Oceanographic Commission of UNESCO. www.obis.org. Accessed: 2019-08-29)

Dataset details:

Dataset_ID	Name	Citation	License	OBIS_citation
8a1ae661-e911-4967-bc06-1168fc5f2d89	iziko South African Museum - Fish Collection	iziko South African Museum - Fish Collection	Restricted	OBIS (2019) [iziko South African Museum - Fish Collection] (Available: Ocean Biodiversity Information System. Intergovernmental Oceanographic Commission of UNESCO. https://obis.org. Accessed: 2019-08-29)
d6d6fe4c-425f-4ce7-bf28-7a6befaeb413	National Museum of Natural History Vertebrate Zoology Fishes Collections	National Museum of Natural History, Smithsonian Institution NMNH Fishes Collection Database. National Museum of Natural History, Smithsonian Institution, 10th and Constitution Ave. N.W., Washington, DC 20560-0193, 2007.	This work is licensed under a Creative Commons Attribution (CC-BY) 4.0 License	OBIS (2019) [National Museum of Natural History Vertebrate Zoology Fishes Collections] (Available: Ocean Biodiversity Information System. Intergovernmental Oceanographic Commission of UNESCO. https://obis.org. Accessed: 2019-08-29)

Protomyctophum subparallelum

GBIF

GBIF Occurrence Download https://doi.org/10.15468/dd.tckzgg Accessed from R via rgbif (https://github.com/ropensci/rgbif) on 2019-09-11

OBIS

OBIS (2019) Distribution records of Protomyctophum subparallelum [Dataset] (Available: Ocean Biodiversity Information System. Intergovernmental Oceanographic Commission of UNESCO. www.obis.org. Accessed: 2019-08-29)

Dataset details:

Dataset_ID	Name	Citation	License	OBIS_citation

25a2facf-bbfa-4b3a-b350-a9c2dc3edc3e	Biological data from the Soviet Antarctic Expedition (1955-1958)	SWPRON (2014): Biological data from the Soviet Antarctic Expedition (1955-1958). v1.1. Dataset/Occurrence. https://nzobisipt.niwa.co.nz/resource?r=soviet_ant_exp&v=1.1	This work is licensed under a Creative Commons Attribution (CC-BY) 4.0 License	OBIS (2019) [Biological data from the Soviet Antarctic Expedition (1955-1958)] (Available: Ocean Biodiversity Information System. Intergovernmental Oceanographic Commission of UNESCO. https://obis.org. Accessed: 2019-08-29)
2870c548-343e-4575-ac67-a4da35182c52	Institution Shirshov Institute - Collection SKAO	No available dataset citation	Unspecified intellectual rights	OBIS (2019) [Institution Shirshov Institute - Collection SKAO] (Available: Ocean Biodiversity Information System. Intergovernmental Oceanographic Commission of UNESCO. https://obis.org. Accessed: 2019-08-29)
8a1ae661-e911-4967-bc06-1168fc5f2d89	iziko South African Museum - Fish Collection	iziko South African Museum - Fish Collection	Restricted	OBIS (2019) [iziko South African Museum - Fish Collection] (Available: Ocean Biodiversity Information System. Intergovernmental Oceanographic Commission of UNESCO. https://obis.org. Accessed: 2019-08-29)
d6d6fe4c-425f-4ce7-bf28-7a6befaeb413	National Museum of Natural History Vertebrate Zoology Fishes Collections	National Museum of Natural History, Smithsonian Institution NMNH Fishes Collection Database. National Museum of Natural History, Smithsonian Institution, 10th and Constitution Ave. N.W., Washington, DC 20560-0193, 2007.	This work is licensed under a Creative Commons Attribution (CC-BY) 4.0 License	OBIS (2019) [National Museum of Natural History Vertebrate Zoology Fishes Collections] (Available: Ocean Biodiversity Information System. Intergovernmental Oceanographic Commission of UNESCO. https://obis.org. Accessed: 2019-08-29)

Gymnoscopelus piabilis

GBIF

GBIF Occurrence Download https://doi.org/10.15468/dd.2vv2c5 Accessed from R via rgbif (https://github.com/ropensci/rgbif) on 2019-09-11

OBIS

OBIS (2019) Distribution records of Gymnoscopelus piabilis [Dataset] (Available: Ocean Biodiversity Information System. Intergovernmental Oceanographic Commission of UNESCO. www.obis.org. Accessed: 2019-08-29)

Dataset details:

Dataset_ID	Name	Citation	License	OBIS_citation
50903a57-ee9f-4367-b2cd-0b36dcf4a6ad	Catch data from New Zealand research trawls since 2008	SWPRON (2017). Catch data from New Zealand research trawls. Southwestern Pacific OBIS, National Institute of Water and Atmospheric Research (NIWA), Wellington, New Zealand, 15157 records, Online http://nzobisipt.niwa.co.nz/resource.do?r=trawl released on April 19, 2017.	This work is licensed under a Creative Commons Attribution (CC-BY) 4.0 License	OBIS (2019) [Catch data from New Zealand research trawls since 2008] (Available: Ocean Biodiversity Information System. Intergovernmental Oceanographic Commission of UNESCO. https://obis.org. Accessed: 2019-08-29)
8a1ae661-e911-4967-bc06-1168fc5f2d89	iziko South African Museum - Fish Collection	iziko South African Museum - Fish Collection	Restricted	OBIS (2019) [iziko South African Museum - Fish Collection] (Available: Ocean Biodiversity Information System. Intergovernmental Oceanographic Commission of UNESCO. https://obis.org. Accessed: 2019-08-29)
9ff216fc-777e-4f9b-9860-95ed7366870d	Institution SAIAB - Collection SAIAB	No available dataset citation	Unspecified intellectual rights	OBIS (2019) [Institution SAIAB - Collection SAIAB] (Available: Ocean Biodiversity Information System. Intergovernmental Oceanographic Commission of UNESCO. https://obis.org. Accessed: 2019-08-29)
a4f7ee48-0d0b-4c05-a972-27a43b30db58	Institution MCM - Collection DEM	No available dataset citation	Unspecified intellectual rights	OBIS (2019) [Institution MCM - Collection DEM] (Available: Ocean Biodiversity Information System. Intergovernmental Oceanographic Commission of UNESCO. https://obis.org. Accessed: 2019-08-29)
b8617377-eb1c-4db2-baa6-8788a632e810	Ichthyology Collection - Royal Ontario Museum	NA	This work is licensed under a Creative Commons Attribution Non Commercial (CC-BY-NC) 4.0 License	OBIS (2019) [Ichthyology Collection - Royal Ontario Museum] (Available: Ocean Biodiversity Information System. Intergovernmental Oceanographic Commission of UNESCO. https://obis.org. Accessed: 2019-08-29)
d6d6fe4c-425f-4ce7-bf28-7a6befaeb413	National Museum of Natural History Vertebrate Zoology Fishes Collections	National Museum of Natural History, Smithsonian Institution NMNH Fishes Collection Database. National Museum of Natural History, Smithsonian Institution, 10th and Constitution Ave. N.W., Washington, DC 20560-0193, 2007.	This work is licensed under a Creative Commons Attribution (CC-BY) 4.0 License	OBIS (2019) [National Museum of Natural History Vertebrate Zoology Fishes Collections] (Available: Ocean Biodiversity Information System. Intergovernmental Oceanographic Commission of UNESCO. https://obis.org. Accessed: 2019-08-29)

Electrona paucirastra

GBIF

GBIF Occurrence Download https://doi.org/10.15468/dd.52bqew Accessed from R via rgbif (https://github.com/ropensci/rgbif) on 2019-09-11

OBIS

OBIS (2019) Distribution records of Electrona paucirastra [Dataset] (Available: Ocean Biodiversity Information System. Intergovernmental Oceanographic Commission of UNESCO. www.obis.org. Accessed: 2019-08-29)

Dataset details:

Dataset_ID	Name	Citation	License	OBIS_citation
0c9db499-759b-46d8-8989-799f9ff9f235	Auckland Museum NZ Marine Collection	Blom W, Moriarty A (2018). Auckland Museum NZ Marine Collection. Version 1.11. Auckland War Memorial Museum. Occurrence Dataset https://doi.org/10.15468/plyefd accessed via GBIF.org on 2018-01-15.	This work is licensed under a Creative Commons Attribution (CC-BY) 4.0 License	OBIS (2019) [Auckland Museum NZ Marine Collection] (Available: Ocean Biodiversity Information System. Intergovernmental Oceanographic Commission of UNESCO. https://obis.org. Accessed: 2019-08-29)
8a1ae661-e911-4967-bc06-1168fc5f2d89	iziko South African Museum - Fish Collection	iziko South African Museum - Fish Collection	Restricted	OBIS (2019) [iziko South African Museum - Fish Collection] (Available: Ocean Biodiversity Information System. Intergovernmental Oceanographic Commission of UNESCO. https://obis.org. Accessed: 2019-08-29)

Metelectrona ventralis

GBIF

GBIF Occurrence Download https://doi.org/10.15468/dd.9wegb4 Accessed from R via rgbif (https://github.com/ropensci/rgbif) on 2019-09-11

OBIS

OBIS (2019) Distribution records of Metelectrona ventralis [Dataset] (Available: Ocean Biodiversity Information System. Intergovernmental Oceanographic Commission of UNESCO. www.obis.org. Accessed: 2019-08-29)

Dataset details:

Dataset_ID	Name	Citation	License	OBIS_citation
50903a57-ee9f-4367-b2cd-0b36dcf4a6ad	Catch data from New Zealand research trawls since 2008	SWPRON (2017). Catch data from New Zealand research trawls. Southwestern Pacific OBIS, National Institute of Water and Atmospheric Research (NIWA), Wellington, New Zealand, 15157 records, Online http://nzobisipt.niwa.co.nz/resource.do?r=trawl released on April 19, 2017.	This work is licensed under a Creative Commons Attribution (CC-BY) 4.0 License	OBIS (2019) [Catch data from New Zealand research trawls since 2008] (Available: Ocean Biodiversity Information System. Intergovernmental Oceanographic Commission of UNESCO. https://obis.org. Accessed: 2019-08-29)
8a1ae661-e911-4967-bc06-1168fc5f2d89	iziko South African Museum - Fish Collection	iziko South African Museum - Fish Collection	Restricted	OBIS (2019) [iziko South African Museum - Fish Collection] (Available: Ocean Biodiversity Information System. Intergovernmental Oceanographic Commission of UNESCO. https://obis.org. Accessed: 2019-08-29)
9ff216fc-777e-4f9b-9860-95ed7366870d	Institution SAIAB - Collection SAIAB	No available dataset citation	Unspecified intellectual rights	OBIS (2019) [Institution SAIAB - Collection SAIAB] (Available: Ocean Biodiversity Information System. Intergovernmental Oceanographic Commission of UNESCO. https://obis.org. Accessed: 2019-08-29)
a4f7ee48-0d0b-4c05-a972-27a43b30db58	Institution MCM - Collection DEM	No available dataset citation	Unspecified intellectual rights	OBIS (2019) [Institution MCM - Collection DEM] (Available: Ocean Biodiversity Information System. Intergovernmental Oceanographic Commission of UNESCO. https://obis.org. Accessed: 2019-08-29)
d6d6fe4c-425f-4ce7-bf28-7a6befaeb413	National Museum of Natural History Vertebrate Zoology Fishes Collections	National Museum of Natural History, Smithsonian Institution NMNH Fishes Collection Database. National Museum of Natural History, Smithsonian Institution, 10th and Constitution Ave. N.W., Washington, DC 20560-0193, 2007.	This work is licensed under a Creative Commons Attribution (CC-BY) 4.0 License	OBIS (2019) [National Museum of Natural History Vertebrate Zoology Fishes Collections] (Available: Ocean Biodiversity Information System. Intergovernmental Oceanographic Commission of UNESCO. https://obis.org. Accessed: 2019-08-29)

Symbolophorus boops

GBIF

GBIF Occurrence Download https://doi.org/10.15468/dd.42swyn Accessed from R via rgbif (https://github.com/ropensci/rgbif) on 2019-09-11

OBIS

OBIS (2019) Distribution records of Symbolophorus boops [Dataset] (Available: Ocean Biodiversity Information System. Intergovernmental Oceanographic Commission of UNESCO. www.obis.org. Accessed: 2019-08-29)

Dataset details:

Dataset_ID	Name	Citation	License	OBIS_citation
270f3e70-ff9b-411d-b170-2bc914d83f26	Biological Reference Collections ICM CSIC	Olivas González F J (2016): Biological Reference Collections ICM CSIC. Institute of Marine Sciences (ICM-CSIC). https://dx.doi.org/10.15470/qlqqdx	This work is licensed under a Creative Commons Attribution Non Commercial (CC-BY-NC) 4.0 License	OBIS (2019) [Biological Reference Collections ICM CSIC] (Available: Ocean Biodiversity Information System. Intergovernmental Oceanographic Commission of UNESCO. https://obis.org. Accessed: 2019-08-29)
4bdc1f6f-e16a-48b4-b995-b51bd41caa8d	Dataset of the multidisciplinary research surveys in the seamounts of Ewing and Valdivia Bank (Walvis Ridge) - SE Atlantic	López-Abellán, L. J.; Sarralde Vizuete, R.; González Jiménez, J. F.; Centro Oceanográfico de Canarias – IEO, Spain (2015). Dataset of the multidisciplinary research surveys in the seamounts of Ewing and Valdivia Bank (Walvis Ridge) - SE Atlantic https://dx.doi.org/10.14284/58	Attribution-NoDerivatives (CC BY-ND)	OBIS (2019) [Dataset of the multidisciplinary research surveys in the seamounts of Ewing and Valdivia Bank (Walvis Ridge) - SE Atlantic] (Available: Ocean Biodiversity Information System. Intergovernmental Oceanographic Commission of UNESCO. https://obis.org. Accessed: 2019-08-29)
50903a57-ee9f-4367-b2cd-0b36dcf4a6ad	Catch data from New Zealand research trawls since 2008	SWPRON (2017). Catch data from New Zealand research trawls. Southwestern Pacific OBIS, National Institute of Water and Atmospheric Research (NIWA), Wellington, New Zealand, 15157 records, Online http://nzobisipt.niwa.co.nz/resource.do?r=trawl released on April 19, 2017.	This work is licensed under a Creative Commons Attribution (CC-BY) 4.0 License	OBIS (2019) [Catch data from New Zealand research trawls since 2008] (Available: Ocean Biodiversity Information System. Intergovernmental Oceanographic Commission of UNESCO. https://obis.org. Accessed: 2019-08-29)
705770e5-3474-4e69-be8b-3107a0c5610a	The fishes collection (IC) of the Muséum national d'Histoire naturelle (MNHN - Paris)	Gicim data base, Pruvost P. Causse R., 2009 http://doi.org/10.15468/tm7whu	This work is licensed under a Creative Commons Attribution (CC-BY) 4.0 License	OBIS (2019) [The fishes collection (IC) of the Muséum national d'Histoire naturelle (MNHN - Paris)] (Available: Ocean Biodiversity Information System. Intergovernmental Oceanographic Commission of UNESCO. https://obis.org. Accessed: 2019-08-29)

8a1ae661-e911-4967-bc06-1168fc5f2d89	iziko South African Museum - Fish Collection	iziko South African Museum - Fish Collection	Restricted	OBIS (2019) [iziko South African Museum - Fish Collection] (Available: Ocean Biodiversity Information System. Intergovernmental Oceanographic Commission of UNESCO. https://obis.org. Accessed: 2019-08-29)
9ff216fc-777e-4f9b-9860-95ed7366870d	Institution SAIAB - Collection SAIAB	No available dataset citation	Unspecified intellectual rights	OBIS (2019) [Institution SAIAB - Collection SAIAB] (Available: Ocean Biodiversity Information System. Intergovernmental Oceanographic Commission of UNESCO. https://obis.org. Accessed: 2019-08-29)
a4f7ee48-0d0b-4c05-a972-27a43b30db58	Institution MCM - Collection DEM	No available dataset citation	Unspecified intellectual rights	OBIS (2019) [Institution MCM - Collection DEM] (Available: Ocean Biodiversity Information System. Intergovernmental Oceanographic Commission of UNESCO. https://obis.org. Accessed: 2019-08-29)
b8617377-eb1c-4db2-baa6-8788a632e810	Ichthyology Collection - Royal Ontario Museum	NA	This work is licensed under a Creative Commons Attribution Non Commercial (CC-BY-NC) 4.0 License	OBIS (2019) [Ichthyology Collection - Royal Ontario Museum] (Available: Ocean Biodiversity Information System. Intergovernmental Oceanographic Commission of UNESCO. https://obis.org. Accessed: 2019-08-29)
d6d6fe4c-425f-4ce7-bf28-7a6befaeb413	National Museum of Natural History Vertebrate Zoology Fishes Collections	National Museum of Natural History, Smithsonian Institution NMNH Fishes Collection Database. National Museum of Natural History, Smithsonian Institution, 10th and Constitution Ave. N.W., Washington, DC 20560-0193, 2007.	This work is licensed under a Creative Commons Attribution (CC-BY) 4.0 License	OBIS (2019) [National Museum of Natural History Vertebrate Zoology Fishes Collections] (Available: Ocean Biodiversity Information System. Intergovernmental Oceanographic Commission of UNESCO. https://obis.org. Accessed: 2019-08-29)

Protomyctophum luciferum

GBIF

GBIF Occurrence Download https://doi.org/10.15468/dd.vaw8w4 Accessed from R via rgbif (https://github.com/ropensci/rgbif) on 2019-09-11

OBIS

OBIS (2019) Distribution records of Protomyctophum luciferum [Dataset] (Available: Ocean Biodiversity Information System. Intergovernmental Oceanographic Commission of UNESCO. www.obis.org. Accessed: 2019-08-29)

Dataset details:

Dataset_ID	Name	Citation	License	OBIS_citation
8a1ae661-e911-4967-bc06-1168fc5f2d89	iziko South African Museum - Fish Collection	iziko South African Museum - Fish Collection	Restricted	OBIS (2019) [iziko South African Museum - Fish Collection] (Available: Ocean Biodiversity Information System. Intergovernmental Oceanographic Commission of UNESCO. https://obis.org. Accessed: 2019-08-29)

Lampanyctus australis

GBIF

GBIF Occurrence Download https://doi.org/10.15468/dd.ws5j8g Accessed from R via rgbif (https://github.com/ropensci/rgbif) on 2019-09-11

OBIS

OBIS (2019) Distribution records of Lampanyctus australis [Dataset] (Available: Ocean Biodiversity Information System. Intergovernmental Oceanographic Commission of UNESCO. www.obis.org. Accessed: 2019-08-29)

Dataset details:

Dataset_ID	Name	Citation	License	OBIS_citation
270f3e70-ff9b-411d-b170-2bc914d83f26	Biological Reference Collections ICM CSIC	Olivas González F J (2016): Biological Reference Collections ICM CSIC. Institute of Marine Sciences (ICM-CSIC). https://dx.doi.org/10.15470/qlqqdx	This work is licensed under a Creative Commons Attribution Non Commercial (CC-BY-NC) 4.0 License	OBIS (2019) [Biological Reference Collections ICM CSIC] (Available: Ocean Biodiversity Information System. Intergovernmental Oceanographic Commission of UNESCO. https://obis.org. Accessed: 2019-08-29)

2870c548-343e-4575-ac67-a4da35182c52	Institution Shirshov Institute - Collection SKAO	No available dataset citation	Unspecified intellectual rights	OBIS (2019) [Institution Shirshov Institute - Collection SKAO] (Available: Ocean Biodiversity Information System. Intergovernmental Oceanographic Commission of UNESCO. https://obis.org. Accessed: 2019-08-29)
50903a57-ee9f-4367-b2cd-0b36dcf4a6ad	Catch data from New Zealand research trawls since 2008	SWPRON (2017). Catch data from New Zealand research trawls. Southwestern Pacific OBIS, National Institute of Water and Atmospheric Research (NIWA), Wellington, New Zealand, 15157 records, Online http://nzobisipt.niwa.co.nz/resource.do?r=trawl released on April 19, 2017.	This work is licensed under a Creative Commons Attribution (CC-BY) 4.0 License	OBIS (2019) [Catch data from New Zealand research trawls since 2008] (Available: Ocean Biodiversity Information System. Intergovernmental Oceanographic Commission of UNESCO. https://obis.org. Accessed: 2019-08-29)
685b3956-c37a-433a-b661-2bb7b11cf9f8	Soviet Trawl Fishery Data (New Zealand Waters) 1964-1987	Ministry for Primary Industries (2014). Soviet Fishery Data (New Zealand Waters) 1964-1987. Southwestern Pacific OBIS, National Institute of Water and Atmospheric Research (NIWA), Wellington, New Zealand, 111883 records, Online http://nzobisipt.niwa.co.nz/resource.do?r=mbis_soviettrawl released on November 5, 2014.	This work is licensed under a Creative Commons Attribution (CC-BY) 4.0 License	OBIS (2019) [Soviet Trawl Fishery Data (New Zealand Waters) 1964-1987] (Available: Ocean Biodiversity Information System. Intergovernmental Oceanographic Commission of UNESCO. https://obis.org. Accessed: 2019-08-29)
8a1ae661-e911-4967-bc06-1168fc5f2d89	iziko South African Museum - Fish Collection	iziko South African Museum - Fish Collection	Restricted	OBIS (2019) [iziko South African Museum - Fish Collection] (Available: Ocean Biodiversity Information System. Intergovernmental Oceanographic Commission of UNESCO. https://obis.org. Accessed: 2019-08-29)
9ff216fc-777e-4f9b-9860-95ed7366870d	Institution SAIAB - Collection SAIAB	No available dataset citation	Unspecified intellectual rights	OBIS (2019) [Institution SAIAB - Collection SAIAB] (Available: Ocean Biodiversity Information System. Intergovernmental Oceanographic Commission of UNESCO. https://obis.org. Accessed: 2019-08-29)
a4f7ee48-0d0b-4c05-a972-27a43b30db58	Institution MCM - Collection DEM	No available dataset citation	Unspecified intellectual rights	OBIS (2019) [Institution MCM - Collection DEM] (Available: Ocean Biodiversity Information System. Intergovernmental Oceanographic Commission of UNESCO. https://obis.org. Accessed: 2019-08-29)

b8617377-eb1c-4db2-baa6-8788a632e810	Ichthyology Collection - Royal Ontario Museum	NA	This work is licensed under a Creative Commons Attribution Non Commercial (CC-BY-NC) 4.0 License	OBIS (2019) [Ichthyology Collection - Royal Ontario Museum] (Available: Ocean Biodiversity Information System. Intergovernmental Oceanographic Commission of UNESCO. https://obis.org. Accessed: 2019-08-29)
d286ae50-ea29-4aa4-8028-2e6e5945a039	Institution REVIZEE - Collection Demersal Fishes	No available dataset citation	Unspecified intellectual rights	OBIS (2019) [Institution REVIZEE - Collection Demersal Fishes] (Available: Ocean Biodiversity Information System. Intergovernmental Oceanographic Commission of UNESCO. https://obis.org. Accessed: 2019-08-29)
d6d6fe4c-425f-4ce7-bf28-7a6befaeb413	National Museum of Natural History Vertebrate Zoology Fishes Collections	National Museum of Natural History, Smithsonian Institution NMNH Fishes Collection Database. National Museum of Natural History, Smithsonian Institution, 10th and Constitution Ave. N.W., Washington, DC 20560-0193, 2007.	This work is licensed under a Creative Commons Attribution (CC-BY) 4.0 License	OBIS (2019) [National Museum of Natural History Vertebrate Zoology Fishes Collections] (Available: Ocean Biodiversity Information System. Intergovernmental Oceanographic Commission of UNESCO. https://obis.org. Accessed: 2019-08-29)

Hintonia candens

GBIF

GBIF Occurrence Download https://doi.org/10.15468/dd.mg6765 Accessed from R via rgbif (https://github.com/ropensci/rgbif) on 2019-09-11

OBIS

OBIS (2019) Distribution records of Hintonia candens [Dataset] (Available: Ocean Biodiversity Information System. Intergovernmental Oceanographic Commission of UNESCO. www.obis.org. Accessed: 2019-08-29)

Dataset details:

Dataset_ID	Name	Citation	License	OBIS_citation

8a1ae661-e911-4967-bc06-1168fc5f2d89	iziko South African Museum - Fish Collection	iziko South African Museum - Fish Collection	Restricted	OBIS (2019) [iziko South African Museum - Fish Collection] (Available: Ocean Biodiversity Information System. Intergovernmental Oceanographic Commission of UNESCO. https://obis.org. Accessed: 2019-08-29)
ff8b7809-41bc-40ad-8160-0e33862817a0	Biodiversity Research Museum, Academia Sinica, Taiwan	TELDAP, Biodiversity Research Museum, Academia Sinica, Taiwan (accessed through GBIF data portal, http://data.gbif.org/datasets/resource/9093, yyyy-mm-dd)	Unrestricted	OBIS (2019) [Biodiversity Research Museum, Academia Sinica, Taiwan] (Available: Ocean Biodiversity Information System. Intergovernmental Oceanographic Commission of UNESCO. https://obis.org. Accessed: 2019-08-29)

Diaphus hudsoni

GBIF

GBIF Occurrence Download https://doi.org/10.15468/dd.eypts4 Accessed from R via rgbif (https://github.com/ropensci/rgbif) on 2019-09-11

OBIS

OBIS (2019) Distribution records of Diaphus hudsoni [Dataset] (Available: Ocean Biodiversity Information System. Intergovernmental Oceanographic Commission of UNESCO. www.obis.org. Accessed: 2019-08-29)

Dataset details:

Dataset_ID	Name	Citation	License	OBIS_citation
2870c548-343e-4575-ac67-a4da35182c52	Institution Shirshov Institute - Collection SKAO	No available dataset citation	Unspecified intellectual rights	OBIS (2019) [Institution Shirshov Institute - Collection SKAO] (Available: Ocean Biodiversity Information System. Intergovernmental Oceanographic Commission of UNESCO. https://obis.org. Accessed: 2019-08-29)

50903a57-ee9f-4367-b2cd-0b36dcf4a6ad	Catch data from New Zealand research trawls since 2008	SWPRON (2017). Catch data from New Zealand research trawls. Southwestern Pacific OBIS, National Institute of Water and Atmospheric Research (NIWA), Wellington, New Zealand, 15157 records, Online http://nzobisipt.niwa.co.nz/resource.do?r=trawl released on April 19, 2017.	This work is licensed under a Creative Commons Attribution (CC-BY) 4.0 License	OBIS (2019) [Catch data from New Zealand research trawls since 2008] (Available: Ocean Biodiversity Information System. Intergovernmental Oceanographic Commission of UNESCO. https://obis.org. Accessed: 2019-08-29)
705770e5-3474-4e69-be8b-3107a0c5610a	The fishes collection (IC) of the Muséum national d'Histoire naturelle (MNHN - Paris)	Gicim data base, Pruvost P. Causse R., 2009 http://doi.org/10.15468/tm7whu	This work is licensed under a Creative Commons Attribution (CC-BY) 4.0 License	OBIS (2019) [The fishes collection (IC) of the Muséum national d'Histoire naturelle (MNHN - Paris)] (Available: Ocean Biodiversity Information System. Intergovernmental Oceanographic Commission of UNESCO. https://obis.org. Accessed: 2019-08-29)
8a1ae661-e911-4967-bc06-1168fc5f2d89	iziko South African Museum - Fish Collection	iziko South African Museum - Fish Collection	Restricted	OBIS (2019) [iziko South African Museum - Fish Collection] (Available: Ocean Biodiversity Information System. Intergovernmental Oceanographic Commission of UNESCO. https://obis.org. Accessed: 2019-08-29)
9ff216fc-777e-4f9b-9860-95ed7366870d	Institution SAIAB - Collection SAIAB	No available dataset citation	Unspecified intellectual rights	OBIS (2019) [Institution SAIAB - Collection SAIAB] (Available: Ocean Biodiversity Information System. Intergovernmental Oceanographic Commission of UNESCO. https://obis.org. Accessed: 2019-08-29)
a4f7ee48-0d0b-4c05-a972-27a43b30db58	Institution MCM - Collection DEM	No available dataset citation	Unspecified intellectual rights	OBIS (2019) [Institution MCM - Collection DEM] (Available: Ocean Biodiversity Information System. Intergovernmental Oceanographic Commission of UNESCO. https://obis.org. Accessed: 2019-08-29)
b8617377-eb1c-4db2-baa6-8788a632e810	Ichthyology Collection - Royal Ontario Museum	NA	This work is licensed under a Creative Commons Attribution Non Commercial (CC-BY-NC) 4.0 License	OBIS (2019) [Ichthyology Collection - Royal Ontario Museum] (Available: Ocean Biodiversity Information System. Intergovernmental Oceanographic Commission of UNESCO. https://obis.org. Accessed: 2019-08-29)

d6d6fe4c-425f-4ce7-bf28-7a6befaeb413	National Museum of Natural History Vertebrate Zoology Fishes Collections	National Museum of Natural History, Smithsonian Institution NMNH Fishes Collection Database. National Museum of Natural History, Smithsonian Institution, 10th and Constitution Ave. N.W., Washington, DC 20560-0193, 2007.	This work is licensed under a Creative Commons Attribution (CC-BY) 4.0 License	OBIS (2019) [National Museum of Natural History Vertebrate Zoology Fishes Collections] (Available: Ocean Biodiversity Information System. Intergovernmental Oceanographic Commission of UNESCO. https://obis.org. Accessed: 2019-08-29)

Lampadena speculigera

GBIF

GBIF Occurrence Download https://doi.org/10.15468/dd.94sg4y Accessed from R via rgbif (https://github.com/ropensci/rgbif) on 2019-09-11

OBIS

OBIS (2019) Distribution records of Lampadena speculigera [Dataset] (Available: Ocean Biodiversity Information System. Intergovernmental Oceanographic Commission of UNESCO. www.obis.org. Accessed: 2019-08-29)

Dataset details:

Dataset_ID	Name	Citation	License	OBIS_citation
18e4fa5b-5f92-4e09-957b-b242003287e9	Northern Gulf of St. Lawrence Fishes	Dutil J, Nozères C (2023). Northern Gulf of St. Lawrence Fishes. Version 40.6. Fisheries and Oceans Canada. Occurrence dataset. http://iobis.org/mapper/?resource_id=2671	This work is licensed under a Creative Commons Attribution (CC-BY) 4.0 License	OBIS (2019) [Northern Gulf of St. Lawrence Fishes] (Available: Ocean Biodiversity Information System. Intergovernmental Oceanographic Commission of UNESCO. https://obis.org. Accessed: 2019-08-29)
1f59030f-f116-4c34-915e-1882d819cda3	Institution Southampton Oceanography Ceter - Collection discovery	No available dataset citation	Unspecified intellectual rights	OBIS (2019) [Institution Southampton Oceanography Ceter - Collection discovery] (Available: Ocean Biodiversity Information System. Intergovernmental Oceanographic Commission of UNESCO. https://obis.org. Accessed: 2019-08-29)

1febfcdd-3e1b-46db-b73d-1bbe81ce22a6	DFO Quebec Region MLI museum collection	Miller R, Nozères C (2025). DFO Quebec Region MLI museum collection. Version 3.19. Fisheries and Oceans Canada. Occurrence dataset. http://iobis.org/mapper/?resource_id=2673	This work is licensed under a Creative Commons Attribution (CC-BY) 4.0 License	OBIS (2019) [DFO Quebec Region MLI museum collection] (Available: Ocean Biodiversity Information System. Intergovernmental Oceanographic Commission of UNESCO. https://obis.org. Accessed: 2019-08-29)
270f3e70-ff9b-411d-b170-2bc914d83f26	Biological Reference Collections ICM CSIC	Olivas González F J (2016): Biological Reference Collections ICM CSIC. Institute of Marine Sciences (ICM-CSIC). https://dx.doi.org/10.15470/qlqqdx	This work is licensed under a Creative Commons Attribution Non Commercial (CC-BY-NC) 4.0 License	OBIS (2019) [Biological Reference Collections ICM CSIC] (Available: Ocean Biodiversity Information System. Intergovernmental Oceanographic Commission of UNESCO. https://obis.org. Accessed: 2019-08-29)
3d922162-062c-4ad2-bf4a-f2493bd3a95d	Institution Bedford Institute of Oceanography (BIO) - Collection SUMMER	No available dataset citation	Unspecified intellectual rights	OBIS (2019) [Institution Bedford Institute of Oceanography (BIO) - Collection SUMMER] (Available: Ocean Biodiversity Information System. Intergovernmental Oceanographic Commission of UNESCO. https://obis.org. Accessed: 2019-08-29)
50903a57-ee9f-4367-b2cd-0b36dcf4a6ad	Catch data from New Zealand research trawls since 2008	SWPRON (2017). Catch data from New Zealand research trawls. Southwestern Pacific OBIS, National Institute of Water and Atmospheric Research (NIWA), Wellington, New Zealand, 15157 records, Online http://nzobisipt.niwa.co.nz/resource.do?r=trawl released on April 19, 2017.	This work is licensed under a Creative Commons Attribution (CC-BY) 4.0 License	OBIS (2019) [Catch data from New Zealand research trawls since 2008] (Available: Ocean Biodiversity Information System. Intergovernmental Oceanographic Commission of UNESCO. https://obis.org. Accessed: 2019-08-29)
5533fa1e-d1a6-47dc-b93e-bc51e2692589	MAR-ECO 2003 - Arni Fridriksson	Hafsteinn G. Gudfinnson, Høgni Debes, Tone Falkenhaug, Eilif Gaard, Ástthor Gislason, Hildur Petursdottir, Thorsteinn Sigurdsson, and Hedinn Valdimarsson. 2008. Abundance and productivity of the pelagic ecosystem along a transect across the northern Mid- Atlantic Ridge in June 2003. ICES CM 2008/C:12	This work is licensed under a Creative Commons Attribution (CC-BY) 4.0 License	OBIS (2019) [MAR-ECO 2003 - Arni Fridriksson] (Available: Ocean Biodiversity Information System. Intergovernmental Oceanographic Commission of UNESCO. https://obis.org. Accessed: 2019-08-29)

8629ec33-be4b-4384-933f-a511fbc29967	MAR-ECO 2004	Wenneck, T. de Lange, Falkenhaug, T. and O.A. Bergstad. 2008. Strategies, methods, and technologies adopted on the RV G.O. Sars MAR-ECO expedition to the mid-Atlantic Ridge in 2004. Deep-sea Research II. 55: 6-28.	This work is licensed under a Creative Commons Attribution (CC-BY) 4.0 License	OBIS (2019) [MAR-ECO 2004] (Available: Ocean Biodiversity Information System. Intergovernmental Oceanographic Commission of UNESCO. https://obis.org. Accessed: 2019-08-29)
8a1ae661-e911-4967-bc06-1168fc5f2d89	iziko South African Museum - Fish Collection	iziko South African Museum - Fish Collection	Restricted	OBIS (2019) [iziko South African Museum - Fish Collection] (Available: Ocean Biodiversity Information System. Intergovernmental Oceanographic Commission of UNESCO. https://obis.org. Accessed: 2019-08-29)
9ff216fc-777e-4f9b-9860-95ed7366870d	Institution SAIAB - Collection SAIAB	No available dataset citation	Unspecified intellectual rights	OBIS (2019) [Institution SAIAB - Collection SAIAB] (Available: Ocean Biodiversity Information System. Intergovernmental Oceanographic Commission of UNESCO. https://obis.org. Accessed: 2019-08-29)
b0a7add2-dd9e-4020-9ca4-5df048c8f6a2	Bigood	Fabri, M-C. et al., Ifremer BIOCEAN database (Deep Sea Benthic Fauna). Institut Français de Recherche pour l'Exploitation de la Mer, Ifremer, Issy-les-Moulineaux, France. World Wide Web electronic publication, http://www.ifremer.fr/isi/biocean	This work is licensed under a Creative Commons Attribution (CC-BY) 4.0 License	OBIS (2019) [Bigood] (Available: Ocean Biodiversity Information System. Intergovernmental Oceanographic Commission of UNESCO. https://obis.org. Accessed: 2019-08-29)
b8617377-eb1c-4db2-baa6-8788a632e810	Ichthyology Collection - Royal Ontario Museum	NA	This work is licensed under a Creative Commons Attribution Non Commercial (CC-BY-NC) 4.0 License	OBIS (2019) [Ichthyology Collection - Royal Ontario Museum] (Available: Ocean Biodiversity Information System. Intergovernmental Oceanographic Commission of UNESCO. https://obis.org. Accessed: 2019-08-29)
c24bf1c2-2c62-4056-a841-56d94e6e876a	Fish specimens	ROM Fish Collection (accessed through GBIF data portal, http://data.gbif.org/datasets/resource/660, 2012-01-20) http://doi.org/10.15468/syisbx	Unrestricted	OBIS (2019) [Fish specimens] (Available: Ocean Biodiversity Information System. Intergovernmental Oceanographic Commission of UNESCO. https://obis.org. Accessed: 2019-08-29)

cc8f28ce-e48d-4945-abfe-9d150a22dcd6	Hamburg pelagic fish database	Post, A. 1987. Pelagic transects of FRVs "Walther Herwig" and "Anton Dohrn" in the Atlantic Ocean 1966 to 1986. Mitt. Inst. f. Seefischerei d. BfaFi Hamburg, 42: 1-68.	This work is licensed under a Creative Commons Attribution (CC-BY) 4.0 License	OBIS (2019) [Hamburg pelagic fish database] (Available: Ocean Biodiversity Information System. Intergovernmental Oceanographic Commission of UNESCO. https://obis.org. Accessed: 2019-08-29)
ce1d93f3-8b0f-4ee7-9a4d-0393a6ec7fea	Atlantic Reference Centre Museum of Canadian Atlantic Organisms - Invertebrates and Fishes Data	Van Guelpen, L., 2016. Atlantic Reference Centre Museum of Canadian Atlantic Organisms - Invertebrates and Fishes Data. Version 4 In OBIS Canada Digital Collections. Bedford Institute of Oceanography, Dartmouth, NS, Canada. Published by OBIS, Digital http://www.iobis.org/. Accessed on –INSERT DATE	This work is licensed under a Creative Commons Attribution (CC-BY) 4.0 License	OBIS (2019) [Atlantic Reference Centre Museum of Canadian Atlantic Organisms - Invertebrates and Fishes Data] (Available: Ocean Biodiversity Information System. Intergovernmental Oceanographic Commission of UNESCO. https://obis.org. Accessed: 2019-08-29)
ce3c5c7d-daa0-42ed-9cdb-7100b1274b55	DFO Quebec Region Ecosystemic bottom trawl surveys 2004-2022	Bernier D, Boulanger M, Bourdages H, Nozères C, Vanier C, Isabel L (2025). DFO Quebec Region Ecosystemic bottom trawl surveys 2004-2022. Version 3.8. Fisheries and Oceans Canada. Samplingevent dataset. http://iobis.org/mapper/?resource_id=2542	This work is licensed under a Creative Commons Attribution (CC-BY) 4.0 License	OBIS (2019) [DFO Quebec Region Ecosystemic bottom trawl surveys 2004-2022] (Available: Ocean Biodiversity Information System. Intergovernmental Oceanographic Commission of UNESCO. https://obis.org. Accessed: 2019-08-29)
cfc56587-48c3-4e3d-9350-3a4d9a28b681	Institution NOAA, NMFS, Northeast Fisheries Science Center - Collection DEEPWATER SYSTEMATICS	No available dataset citation	Unspecified intellectual rights	OBIS (2019) [Institution NOAA, NMFS, Northeast Fisheries Science Center - Collection DEEPWATER SYSTEMATICS] (Available: Ocean Biodiversity Information System. Intergovernmental Oceanographic Commission of UNESCO. https://obis.org. Accessed: 2019-08-29)
d6d6fe4c-425f-4ce7-bf28-7a6befaeb413	National Museum of Natural History Vertebrate Zoology Fishes Collections	National Museum of Natural History, Smithsonian Institution NMNH Fishes Collection Database. National Museum of Natural History, Smithsonian Institution, 10th and Constitution Ave. N.W., Washington, DC 20560-0193, 2007.	This work is licensed under a Creative Commons Attribution (CC-BY) 4.0 License	OBIS (2019) [National Museum of Natural History Vertebrate Zoology Fishes Collections] (Available: Ocean Biodiversity Information System. Intergovernmental Oceanographic Commission of UNESCO. https://obis.org. Accessed: 2019-08-29)

Lampichthys procerus

GBIF

GBIF Occurrence Download https://doi.org/10.15468/dd.a3ug3y Accessed from R via rgbif (https://github.com/ropensci/rgbif) on 2019-09-11

OBIS

OBIS (2019) Distribution records of Lampichthys procerus [Dataset] (Available: Ocean Biodiversity Information System. Intergovernmental Oceanographic Commission of UNESCO. www.obis.org. Accessed: 2019-08-29)

Dataset details:

Dataset_ID	Name	Citation	License	OBIS_citation
231dc661-d8d9-4d49-9ff0-45bd21a08541	New Zealand fish and squid distributions from research bottom trawls 1964-2008	NIWA (2014). New Zealand fish and squid distributions from research bottom trawls. Southwestern Pacific OBIS, National Institute of Water and Atmospheric Research (NIWA), Wellington, New Zealand, 486781 records, Online http://nzobisipt.niwa.co.nz/resource.do?r=obisprovider released on May 8, 2014.	This work is licensed under a Creative Commons Attribution (CC-BY) 4.0 License	OBIS (2019) [New Zealand fish and squid distributions from research bottom trawls 1964-2008] (Available: Ocean Biodiversity Information System. Intergovernmental Oceanographic Commission of UNESCO. https://obis.org. Accessed: 2019-08-29)
2870c548-343e-4575-ac67-a4da35182c52	Institution Shirshov Institute - Collection SKAO	No available dataset citation	Unspecified intellectual rights	OBIS (2019) [Institution Shirshov Institute - Collection SKAO] (Available: Ocean Biodiversity Information System. Intergovernmental Oceanographic Commission of UNESCO. https://obis.org. Accessed: 2019-08-29)
50903a57-ee9f-4367-b2cd-0b36dcf4a6ad	Catch data from New Zealand research trawls since 2008	SWPRON (2017). Catch data from New Zealand research trawls. Southwestern Pacific OBIS, National Institute of Water and Atmospheric Research (NIWA), Wellington, New Zealand, 15157 records, Online http://nzobisipt.niwa.co.nz/resource.do?r=trawl released on April 19, 2017.	This work is licensed under a Creative Commons Attribution (CC-BY) 4.0 License	OBIS (2019) [Catch data from New Zealand research trawls since 2008] (Available: Ocean Biodiversity Information System. Intergovernmental Oceanographic Commission of UNESCO. https://obis.org. Accessed: 2019-08-29)

705770e5-3474-4e69-be8b-3107a0c5610a	The fishes collection (IC) of the Muséum national d'Histoire naturelle (MNHN - Paris)	Gicim data base, Pruvost P. Causse R., 2009 http://doi.org/10.15468/tm7whu	This work is licensed under a Creative Commons Attribution (CC-BY) 4.0 License	OBIS (2019) [The fishes collection (IC) of the Muséum national d'Histoire naturelle (MNHN - Paris)] (Available: Ocean Biodiversity Information System. Intergovernmental Oceanographic Commission of UNESCO. https://obis.org. Accessed: 2019-08-29)
8a1ae661-e911-4967-bc06-1168fc5f2d89	iziko South African Museum - Fish Collection	iziko South African Museum - Fish Collection	Restricted	OBIS (2019) [iziko South African Museum - Fish Collection] (Available: Ocean Biodiversity Information System. Intergovernmental Oceanographic Commission of UNESCO. https://obis.org. Accessed: 2019-08-29)
a4f7ee48-0d0b-4c05-a972-27a43b30db58	Institution MCM - Collection DEM	No available dataset citation	Unspecified intellectual rights	OBIS (2019) [Institution MCM - Collection DEM] (Available: Ocean Biodiversity Information System. Intergovernmental Oceanographic Commission of UNESCO. https://obis.org. Accessed: 2019-08-29)
b8617377-eb1c-4db2-baa6-8788a632e810	Ichthyology Collection - Royal Ontario Museum	NA	This work is licensed under a Creative Commons Attribution Non Commercial (CC-BY-NC) 4.0 License	OBIS (2019) [Ichthyology Collection - Royal Ontario Museum] (Available: Ocean Biodiversity Information System. Intergovernmental Oceanographic Commission of UNESCO. https://obis.org. Accessed: 2019-08-29)
d6d6fe4c-425f-4ce7-bf28-7a6befaeb413	National Museum of Natural History Vertebrate Zoology Fishes Collections	National Museum of Natural History, Smithsonian Institution NMNH Fishes Collection Database. National Museum of Natural History, Smithsonian Institution, 10th and Constitution Ave. N.W., Washington, DC 20560-0193, 2007.	This work is licensed under a Creative Commons Attribution (CC-BY) 4.0 License	OBIS (2019) [National Museum of Natural History Vertebrate Zoology Fishes Collections] (Available: Ocean Biodiversity Information System. Intergovernmental Oceanographic Commission of UNESCO. https://obis.org. Accessed: 2019-08-29)

Lampanyctus intricarius

GBIF

GBIF Occurrence Download https://doi.org/10.15468/dd.z8fq7s Accessed from R via rgbif (https://github.com/ropensci/rgbif) on 2019-09-11

OBIS

OBIS (2019) Distribution records of Lampanyctus intricarius [Dataset] (Available: Ocean Biodiversity Information System. Intergovernmental Oceanographic Commission of UNESCO. www.obis.org. Accessed: 2019-08-29)

Dataset details:

Dataset_ID	Name	Citation	License	OBIS_citation
1f59030f-f116-4c34-915e-1882d819cda3	Institution Southampton Oceanography Ceter - Collection discovery	No available dataset citation	Unspecified intellectual rights	OBIS (2019) [Institution Southampton Oceanography Ceter - Collection discovery] (Available: Ocean Biodiversity Information System. Intergovernmental Oceanographic Commission of UNESCO. https://obis.org. Accessed: 2019-08-29)
270f3e70-ff9b-411d-b170-2bc914d83f26	Biological Reference Collections ICM CSIC	Olivas González F J (2016): Biological Reference Collections ICM CSIC. Institute of Marine Sciences (ICM-CSIC). https://dx.doi.org/10.15470/qlqqdx	This work is licensed under a Creative Commons Attribution Non Commercial (CC-BY-NC) 4.0 License	OBIS (2019) [Biological Reference Collections ICM CSIC] (Available: Ocean Biodiversity Information System. Intergovernmental Oceanographic Commission of UNESCO. https://obis.org. Accessed: 2019-08-29)
2870c548-343e-4575-ac67-a4da35182c52	Institution Shirshov Institute - Collection SKAO	No available dataset citation	Unspecified intellectual rights	OBIS (2019) [Institution Shirshov Institute - Collection SKAO] (Available: Ocean Biodiversity Information System. Intergovernmental Oceanographic Commission of UNESCO. https://obis.org. Accessed: 2019-08-29)
50903a57-ee9f-4367-b2cd-0b36dcf4a6ad	Catch data from New Zealand research trawls since 2008	SWPRON (2017). Catch data from New Zealand research trawls. Southwestern Pacific OBIS, National Institute of Water and Atmospheric Research (NIWA), Wellington, New Zealand, 15157 records, Online http://nzobisipt.niwa.co.nz/resource.do?r=trawl released on April 19, 2017.	This work is licensed under a Creative Commons Attribution (CC-BY) 4.0 License	OBIS (2019) [Catch data from New Zealand research trawls since 2008] (Available: Ocean Biodiversity Information System. Intergovernmental Oceanographic Commission of UNESCO. https://obis.org. Accessed: 2019-08-29)

5533fa1e-d1a6-47dc-b93e-bc51e2692589	MAR-ECO 2003 - Arni Fridriksson	Hafsteinn G. Gudfinnson, Høgni Debes, Tone Falkenhaug, Eilif Gaard, Ástthor Gislason, Hildur Petursdottir, Thorsteinn Sigurdsson, and Hedinn Valdimarsson. 2008. Abundance and productivity of the pelagic ecosystem along a transect across the northern Mid- Atlantic Ridge in June 2003. ICES CM 2008/C:12	This work is licensed under a Creative Commons Attribution (CC-BY) 4.0 License	OBIS (2019) [MAR-ECO 2003 - Arni Fridriksson] (Available: Ocean Biodiversity Information System. Intergovernmental Oceanographic Commission of UNESCO. https://obis.org. Accessed: 2019-08-29)
8629ec33-be4b-4384-933f-a511fbc29967	MAR-ECO 2004	Wenneck, T. de Lange, Falkenhaug, T. and O.A. Bergstad. 2008. Strategies, methods, and technologies adopted on the RV G.O. Sars MAR-ECO expedition to the mid-Atlantic Ridge in 2004. Deep-sea Research II. 55: 6-28.	This work is licensed under a Creative Commons Attribution (CC-BY) 4.0 License	OBIS (2019) [MAR-ECO 2004] (Available: Ocean Biodiversity Information System. Intergovernmental Oceanographic Commission of UNESCO. https://obis.org. Accessed: 2019-08-29)
8a1ae661-e911-4967-bc06-1168fc5f2d89	iziko South African Museum - Fish Collection	iziko South African Museum - Fish Collection	Restricted	OBIS (2019) [iziko South African Museum - Fish Collection] (Available: Ocean Biodiversity Information System. Intergovernmental Oceanographic Commission of UNESCO. https://obis.org. Accessed: 2019-08-29)
9ff216fc-777e-4f9b-9860-95ed7366870d	Institution SAIAB - Collection SAIAB	No available dataset citation	Unspecified intellectual rights	OBIS (2019) [Institution SAIAB - Collection SAIAB] (Available: Ocean Biodiversity Information System. Intergovernmental Oceanographic Commission of UNESCO. https://obis.org. Accessed: 2019-08-29)
b8617377-eb1c-4db2-baa6-8788a632e810	Ichthyology Collection - Royal Ontario Museum	NA	This work is licensed under a Creative Commons Attribution Non Commercial (CC-BY-NC) 4.0 License	OBIS (2019) [Ichthyology Collection - Royal Ontario Museum] (Available: Ocean Biodiversity Information System. Intergovernmental Oceanographic Commission of UNESCO. https://obis.org. Accessed: 2019-08-29)
cc8f28ce-e48d-4945-abfe-9d150a22dcd6	Hamburg pelagic fish database	Post, A. 1987. Pelagic transects of FRVs "Walther Herwig" and "Anton Dohrn" in the Atlantic Ocean 1966 to 1986. Mitt. Inst. f. Seefischerei d. BfaFi Hamburg, 42: 1-68.	This work is licensed under a Creative Commons Attribution (CC-BY) 4.0 License	OBIS (2019) [Hamburg pelagic fish database] (Available: Ocean Biodiversity Information System. Intergovernmental Oceanographic Commission of UNESCO. https://obis.org. Accessed: 2019-08-29)

ce1d93f3-8b0f-4ee7-9a4d-0393a6ec7fea	Atlantic Reference Centre Museum of Canadian Atlantic Organisms - Invertebrates and Fishes Data	Van Guelpen, L., 2016. Atlantic Reference Centre Museum of Canadian Atlantic Organisms - Invertebrates and Fishes Data. Version 4 In OBIS Canada Digital Collections. Bedford Institute of Oceanography, Dartmouth, NS, Canada. Published by OBIS, Digital http://www.iobis.org/. Accessed on –INSERT DATE	This work is licensed under a Creative Commons Attribution (CC-BY) 4.0 License	OBIS (2019) [Atlantic Reference Centre Museum of Canadian Atlantic Organisms - Invertebrates and Fishes Data] (Available: Ocean Biodiversity Information System. Intergovernmental Oceanographic Commission of UNESCO. https://obis.org. Accessed: 2019-08-29)
d6d6fe4c-425f-4ce7-bf28-7a6befaeb413	National Museum of Natural History Vertebrate Zoology Fishes Collections	National Museum of Natural History, Smithsonian Institution NMNH Fishes Collection Database. National Museum of Natural History, Smithsonian Institution, 10th and Constitution Ave. N.W., Washington, DC 20560-0193, 2007.	This work is licensed under a Creative Commons Attribution (CC-BY) 4.0 License	OBIS (2019) [National Museum of Natural History Vertebrate Zoology Fishes Collections] (Available: Ocean Biodiversity Information System. Intergovernmental Oceanographic Commission of UNESCO. https://obis.org. Accessed: 2019-08-29)
ff8b7809-41bc-40ad-8160-0e33862817a0	Biodiversity Research Museum, Academia Sinica, Taiwan	TELDAP, Biodiversity Research Museum, Academia Sinica, Taiwan (accessed through GBIF data portal, http://data.gbif.org/datasets/resource/9093, yyyy-mm-dd)	Unrestricted	OBIS (2019) [Biodiversity Research Museum, Academia Sinica, Taiwan] (Available: Ocean Biodiversity Information System. Intergovernmental Oceanographic Commission of UNESCO. https://obis.org. Accessed: 2019-08-29)

Ceratoscopelus warmingii

GBIF

GBIF Occurrence Download https://doi.org/10.15468/dd.z5fnp2 Accessed from R via rgbif (https://github.com/ropensci/rgbif) on 2019-09-11

OBIS

OBIS (2019) Distribution records of Ceratoscopelus warmingii [Dataset] (Available: Ocean Biodiversity Information System. Intergovernmental Oceanographic Commission of UNESCO. www.obis.org. Accessed: 2019-08-29)

Dataset details:

Dataset_ID	Name	Citation	License	OBIS_citation

0332e1b5-5525-4301-9659-ef3da3e4e2b6	MARMAP Isaacs-Kidd Midwater Trawl 1990-2009	Marcel Reichert, 2010, MARMAP Isaacs-Kidd Midwater Trawl 1990-2009, SCDNR/NOAA MARMAP Program, SCDNR MARMAP Aggregate data surveys, The Marine Resources Monitoring, Assessment, and Prediction (MARMAP) Program, Marine Resources Research Institute, South Carolina Department of Natural Resources, P. O. Box 12559, Charleston SC 29422-2559, U.S.A.Retrieved from http://www.usgs.gov/obis-usa/	Restricted	OBIS (2019) [MARMAP Isaacs-Kidd Midwater Trawl 1990-2009] (Available: Ocean Biodiversity Information System. Intergovernmental Oceanographic Commission of UNESCO. https://obis.org. Accessed: 2019-08-29)
04e3fd32-b08b-4806-a016-d2dff52ae55a	Asia-Pacific Dataset	Jintsu-Uchifune, Y., Yamamoto, H. (2016) Marine organism occurrence data of the Asia-Pacific region extracted from literature. Available at https://doi.org/10.48518/00002. Accessed on yyyy-mm-dd.	This work is licensed under a Creative Commons Attribution (CC-BY) 4.0 License	OBIS (2019) [Asia-Pacific Dataset] (Available: Ocean Biodiversity Information System. Intergovernmental Oceanographic Commission of UNESCO. https://obis.org. Accessed: 2019-08-29)
1f59030f-f116-4c34-915e-1882d819cda3	Institution Southampton Oceanography Ceter - Collection discovery	No available dataset citation	Unspecified intellectual rights	OBIS (2019) [Institution Southampton Oceanography Ceter - Collection discovery] (Available: Ocean Biodiversity Information System. Intergovernmental Oceanographic Commission of UNESCO. https://obis.org. Accessed: 2019-08-29)
270f3e70-ff9b-411d-b170-2bc914d83f26	Biological Reference Collections ICM CSIC	Olivas González F J (2016): Biological Reference Collections ICM CSIC. Institute of Marine Sciences (ICM-CSIC). https://dx.doi.org/10.15470/qlqqdx	This work is licensed under a Creative Commons Attribution Non Commercial (CC-BY-NC) 4.0 License	OBIS (2019) [Biological Reference Collections ICM CSIC] (Available: Ocean Biodiversity Information System. Intergovernmental Oceanographic Commission of UNESCO. https://obis.org. Accessed: 2019-08-29)
2870c548-343e-4575-ac67-a4da35182c52	Institution Shirshov Institute - Collection SKAO	No available dataset citation	Unspecified intellectual rights	OBIS (2019) [Institution Shirshov Institute - Collection SKAO] (Available: Ocean Biodiversity Information System. Intergovernmental Oceanographic Commission of UNESCO. https://obis.org. Accessed: 2019-08-29)

308a501c-a187-498e-a8bd-9cf3d2b70bd9	Institution REVIZEE - Collection Ictioplankton	No available dataset citation	Unspecified intellectual rights	OBIS (2019) [Institution REVIZEE - Collection Ictioplankton] (Available: Ocean Biodiversity Information System. Intergovernmental Oceanographic Commission of UNESCO. https://obis.org. Accessed: 2019-08-29)
4354345d-7faf-4376-b326-ffbc04b6b0cd	No available dataset name	No available dataset citation	Unspecified intellectual rights	OBIS (2019) [No available dataset name] (Available: Ocean Biodiversity Information System. Intergovernmental Oceanographic Commission of UNESCO. https://obis.org. Accessed: 2019-08-29)
4bdc1f6f-e16a-48b4-b995-b51bd41caa8d	Dataset of the multidisciplinary research surveys in the seamounts of Ewing and Valdivia Bank (Walvis Ridge) - SE Atlantic	López-Abellán, L. J.; Sarralde Vizuete, R.; González Jiménez, J. F.; Centro Oceanográfico de Canarias – IEO, Spain (2015). Dataset of the multidisciplinary research surveys in the seamounts of Ewing and Valdivia Bank (Walvis Ridge) - SE Atlantic https://dx.doi.org/10.14284/58	Attribution-NoDerivatives (CC BY-ND)	OBIS (2019) [Dataset of the multidisciplinary research surveys in the seamounts of Ewing and Valdivia Bank (Walvis Ridge) - SE Atlantic] (Available: Ocean Biodiversity Information System. Intergovernmental Oceanographic Commission of UNESCO. https://obis.org. Accessed: 2019-08-29)
4e25e0ce-b17d-4192-9b55-417f1e0c4fc8	Institution - Collection	No available dataset citation	Unspecified intellectual rights	OBIS (2019) [Institution - Collection] (Available: Ocean Biodiversity Information System. Intergovernmental Oceanographic Commission of UNESCO. https://obis.org. Accessed: 2019-08-29)
5f2da252-6d49-4c9f-b3b3-1db53d75b345	MARMAP Bongo Nets 1990-2009	Marcel Reichert, 2010, MARMAP Bongo Nets 1990-2009, SCDNR/NOAA MARMAP Program, SCDNR MARMAP Aggregate data surveys, The Marine Resources Monitoring, Assessment, and Prediction (MARMAP) Program, Marine Resources Research Institute, South Carolina Department of Natural Resources, P. O. Box 12559, Charleston SC 29422-2559, U.S.A.Retrieved from http://www.usgs.gov/obis-usa/	Restricted	OBIS (2019) [MARMAP Bongo Nets 1990-2009] (Available: Ocean Biodiversity Information System. Intergovernmental Oceanographic Commission of UNESCO. https://obis.org. Accessed: 2019-08-29)

6a5bc28f-4dfe-4cbf-8a55-7e3a843997ab	SPC NECTALIS Zooplankton/Micronekton specimens, New Caledonia 2014	Allain, V., Menkes, C., 2014. Nectalis 3 cruise, RV Alis. https://doi.org/10.17600/14004900	This work is licensed under a Creative Commons Attribution Non Commercial (CC-BY-NC) 4.0 License	OBIS (2019) [SPC NECTALIS Zooplankton/Micronekton specimens, New Caledonia 2014] (Available: Ocean Biodiversity Information System. Intergovernmental Oceanographic Commission of UNESCO. https://obis.org. Accessed: 2019-08-29)
6c19184e-c305-4273-8890-6d342d86f865	Institution - Collection	No available dataset citation	Unspecified intellectual rights	OBIS (2019) [Institution - Collection] (Available: Ocean Biodiversity Information System. Intergovernmental Oceanographic Commission of UNESCO. https://obis.org. Accessed: 2019-08-29)
705770e5-3474-4e69-be8b-3107a0c5610a	The fishes collection (IC) of the Muséum national d'Histoire naturelle (MNHN - Paris)	Gicim data base, Pruvost P. Causse R., 2009 http://doi.org/10.15468/tm7whu	This work is licensed under a Creative Commons Attribution (CC-BY) 4.0 License	OBIS (2019) [The fishes collection (IC) of the Muséum national d'Histoire naturelle (MNHN - Paris)] (Available: Ocean Biodiversity Information System. Intergovernmental Oceanographic Commission of UNESCO. https://obis.org. Accessed: 2019-08-29)
8629ec33-be4b-4384-933f-a511fbc29967	MAR-ECO 2004	Wenneck, T. de Lange, Falkenhaug, T. and O.A. Bergstad. 2008. Strategies, methods, and technologies adopted on the RV G.O. Sars MAR-ECO expedition to the mid-Atlantic Ridge in 2004. Deep-sea Research II. 55: 6-28.	This work is licensed under a Creative Commons Attribution (CC-BY) 4.0 License	OBIS (2019) [MAR-ECO 2004] (Available: Ocean Biodiversity Information System. Intergovernmental Oceanographic Commission of UNESCO. https://obis.org. Accessed: 2019-08-29)
87a421bf-4646-49e3-89b7-409b93f2ac7c	Institution - Collection	No available dataset citation	Unspecified intellectual rights	OBIS (2019) [Institution - Collection] (Available: Ocean Biodiversity Information System. Intergovernmental Oceanographic Commission of UNESCO. https://obis.org. Accessed: 2019-08-29)
8a1ae661-e911-4967-bc06-1168fc5f2d89	iziko South African Museum - Fish Collection	iziko South African Museum - Fish Collection	Restricted	OBIS (2019) [iziko South African Museum - Fish Collection] (Available: Ocean Biodiversity Information System. Intergovernmental Oceanographic Commission of UNESCO. https://obis.org. Accessed: 2019-08-29)

9ff216fc-777e-4f9b-9860-95ed7366870d	Institution - Collection	No available dataset citation	Unspecified intellectual rights	OBIS (2019) [Institution - Collection] (Available: Ocean Biodiversity Information System. Intergovernmental Oceanographic Commission of UNESCO. https://obis.org. Accessed: 2019-08-29)
a4f7ee48-0d0b-4c05-a972-27a43b30db58	Institution - Collection	No available dataset citation	Unspecified intellectual rights	OBIS (2019) [Institution - Collection] (Available: Ocean Biodiversity Information System. Intergovernmental Oceanographic Commission of UNESCO. https://obis.org. Accessed: 2019-08-29)
cc8f28ce-e48d-4945-abfe-9d150a22dcd6	Hamburg pelagic fish database	Post, A. 1987. Pelagic transects of FRVs "Walther Herwig" and "Anton Dohrn" in the Atlantic Ocean 1966 to 1986. Mitt. Inst. f. Seefischerei d. BfaFi Hamburg, 42: 1-68.	This work is licensed under a Creative Commons Attribution (CC-BY) 4.0 License	OBIS (2019) [Hamburg pelagic fish database] (Available: Ocean Biodiversity Information System. Intergovernmental Oceanographic Commission of UNESCO. https://obis.org. Accessed: 2019-08-29)
ce1d93f3-8b0f-4ee7-9a4d-0393a6ec7fea	Atlantic Reference Centre Museum of Canadian Atlantic Organisms - Invertebrates and Fishes Data	Van Guelpen, L., 2016. Atlantic Reference Centre Museum of Canadian Atlantic Organisms - Invertebrates and Fishes Data. Version 4 In OBIS Canada Digital Collections. Bedford Institute of Oceanography, Dartmouth, NS, Canada. Published by OBIS, Digital http://www.iobis.org/. Accessed on –INSERT DATE	This work is licensed under a Creative Commons Attribution (CC-BY) 4.0 License	OBIS (2019) [Atlantic Reference Centre Museum of Canadian Atlantic Organisms - Invertebrates and Fishes Data] (Available: Ocean Biodiversity Information System. Intergovernmental Oceanographic Commission of UNESCO. https://obis.org. Accessed: 2019-08-29)
cfc56587-48c3-4e3d-9350-3a4d9a28b681	Institution - Collection	No available dataset citation	Unspecified intellectual rights	OBIS (2019) [Institution - Collection] (Available: Ocean Biodiversity Information System. Intergovernmental Oceanographic Commission of UNESCO. https://obis.org. Accessed: 2019-08-29)
cfc56587-48c3-4e3d-9350-3a4d9a28b681	Institution KU - Collection KUI	No available dataset citation	Unspecified intellectual rights	OBIS (2019) [Institution KU - Collection KUI] (Available: Ocean Biodiversity Information System. Intergovernmental Oceanographic Commission of UNESCO. https://obis.org. Accessed: 2019-08-29)

d6d6fe4c-425f-4ce7-bf28-7a6befaeb413	National Museum of Natural History Vertebrate Zoology Fishes Collections	National Museum of Natural History, Smithsonian Institution NMNH Fishes Collection Database. National Museum of Natural History, Smithsonian Institution, 10th and Constitution Ave. N.W., Washington, DC 20560-0193, 2007.	This work is licensed under a Creative Commons Attribution (CC-BY) 4.0 License	OBIS (2019) [National Museum of Natural History Vertebrate Zoology Fishes Collections] (Available: Ocean Biodiversity Information System. Intergovernmental Oceanographic Commission of UNESCO. https://obis.org. Accessed: 2019-08-29)
ff8b7809-41bc-40ad-8160-0e33862817a0	Biodiversity Research Museum, Academia Sinica, Taiwan	TELDAP, Biodiversity Research Museum, Academia Sinica, Taiwan (accessed through GBIF data portal, http://data.gbif.org/datasets/resource/9093, yyyy-mm-dd)	Unrestricted	OBIS (2019) [Biodiversity Research Museum, Academia Sinica, Taiwan] (Available: Ocean Biodiversity Information System. Intergovernmental Oceanographic Commission of UNESCO. https://obis.org. Accessed: 2019-08-29)

Diaphus ostenfeldi

GBIF

GBIF Occurrence Download https://doi.org/10.15468/dd.n85ben Accessed from R via rgbif (https://github.com/ropensci/rgbif) on 2019-09-11

OBIS

OBIS (2019) Distribution records of Diaphus ostenfeldi [Dataset] (Available: Ocean Biodiversity Information System. Intergovernmental Oceanographic Commission of UNESCO. www.obis.org. Accessed: 2019-08-29)

Dataset details:

Dataset_ID	Name	Citation	License	OBIS_citation
0c9db499-759b-46d8-8989-799f9ff9f235	Auckland Museum NZ Marine Collection	Blom W, Moriarty A (2018). Auckland Museum NZ Marine Collection. Version 1.11. Auckland War Memorial Museum. Occurrence Dataset https://doi.org/10.15468/plyefd accessed via GBIF.org on 2018-01-15.	This work is licensed under a Creative Commons Attribution (CC-BY) 4.0 License	OBIS (2019) [Auckland Museum NZ Marine Collection] (Available: Ocean Biodiversity Information System. Intergovernmental Oceanographic Commission of UNESCO. https://obis.org. Accessed: 2019-08-29)

50903a57-ee9f-4367-b2cd-0b36dcf4a6ad	Catch data from New Zealand research trawls since 2008	SWPRON (2017). Catch data from New Zealand research trawls. Southwestern Pacific OBIS, National Institute of Water and Atmospheric Research (NIWA), Wellington, New Zealand, 15157 records, Online http://nzobisipt.niwa.co.nz/resource.do?r=trawl released on April 19, 2017.	This work is licensed under a Creative Commons Attribution (CC-BY) 4.0 License	OBIS (2019) [Catch data from New Zealand research trawls since 2008] (Available: Ocean Biodiversity Information System. Intergovernmental Oceanographic Commission of UNESCO. https://obis.org. Accessed: 2019-08-29)
8a1ae661-e911-4967-bc06-1168fc5f2d89	iziko South African Museum - Fish Collection	iziko South African Museum - Fish Collection	Restricted	OBIS (2019) [iziko South African Museum - Fish Collection] (Available: Ocean Biodiversity Information System. Intergovernmental Oceanographic Commission of UNESCO. https://obis.org. Accessed: 2019-08-29)
a4f7ee48-0d0b-4c05-a972-27a43b30db58	Institution MCM - Collection DEM	No available dataset citation	Unspecified intellectual rights	OBIS (2019) [Institution MCM - Collection DEM] (Available: Ocean Biodiversity Information System. Intergovernmental Oceanographic Commission of UNESCO. https://obis.org. Accessed: 2019-08-29)
d6d6fe4c-425f-4ce7-bf28-7a6befaeb413	National Museum of Natural History Vertebrate Zoology Fishes Collections	National Museum of Natural History, Smithsonian Institution NMNH Fishes Collection Database. National Museum of Natural History, Smithsonian Institution, 10th and Constitution Ave. N.W., Washington, DC 20560-0193, 2007.	This work is licensed under a Creative Commons Attribution (CC-BY) 4.0 License	OBIS (2019) [National Museum of Natural History Vertebrate Zoology Fishes Collections] (Available: Ocean Biodiversity Information System. Intergovernmental Oceanographic Commission of UNESCO. https://obis.org. Accessed: 2019-08-29)

Taaningichthys bathyphilus

GBIF

GBIF Occurrence Download https://doi.org/10.15468/dd.xespsn Accessed from R via rgbif (https://github.com/ropensci/rgbif) on 2019-09-11

OBIS

OBIS (2019) Distribution records of Taaningichthys bathyphilus [Dataset] (Available: Ocean Biodiversity Information System. Intergovernmental Oceanographic Commission of UNESCO. www.obis.org. Accessed: 2019-08-29)

Dataset details:

Dataset_ID	Name	Citation	License	OBIS_citation
1f59030f-f116-4c34-915e-1882d819cda3	Institution Southampton Oceanography Ceter - Collection discovery	No available dataset citation	Unspecified intellectual rights	OBIS (2019) [Institution Southampton Oceanography Ceter - Collection discovery] (Available: Ocean Biodiversity Information System. Intergovernmental Oceanographic Commission of UNESCO. https://obis.org. Accessed: 2019-08-29)
49d1b62c-124a-4854-b878-b83de130eb23	International Polar Year and Census of Antarctic Marine Life Ross Sea voyage (TAN0802) biodiversity data	Ocean Survey 20/20 (2013). International Polar Year and Census of Antarctic Marine Life Ross Sea voyage (TAN0802) biodiversity data. Southwestern Pacific OBIS, National Institute of Water and Atmospheric Research, Wellington, New Zealand, 8748 records, Online http://nzobisipt.elasticbeanstalk.com/resource.do?r=mbis_caml released on Dec 12, 2013.	This work is licensed under a Creative Commons Attribution (CC-BY) 4.0 License	OBIS (2019) [International Polar Year and Census of Antarctic Marine Life Ross Sea voyage (TAN0802) biodiversity data] (Available: Ocean Biodiversity Information System. Intergovernmental Oceanographic Commission of UNESCO. https://obis.org. Accessed: 2019-08-29)
705770e5-3474-4e69-be8b-3107a0c5610a	The fishes collection (IC) of the Muséum national d'Histoire naturelle (MNHN - Paris)	Gicim data base, Pruvost P. Causse R., 2009 http://doi.org/10.15468/tm7whu	This work is licensed under a Creative Commons Attribution (CC-BY) 4.0 License	OBIS (2019) [The fishes collection (IC) of the Muséum national d'Histoire naturelle (MNHN - Paris)] (Available: Ocean Biodiversity Information System. Intergovernmental Oceanographic Commission of UNESCO. https://obis.org. Accessed: 2019-08-29)
8629ec33-be4b-4384-933f-a511fbc29967	MAR-ECO 2004	Wenneck, T. de Lange, Falkenhaug, T. and O.A. Bergstad. 2008. Strategies, methods, and technologies adopted on the RV G.O. Sars MAR-ECO expedition to the mid-Atlantic Ridge in 2004. Deep-sea Research II. 55: 6-28.	This work is licensed under a Creative Commons Attribution (CC-BY) 4.0 License	OBIS (2019) [MAR-ECO 2004] (Available: Ocean Biodiversity Information System. Intergovernmental Oceanographic Commission of UNESCO. https://obis.org. Accessed: 2019-08-29)
8a1ae661-e911-4967-bc06-1168fc5f2d89	iziko South African Museum - Fish Collection	iziko South African Museum - Fish Collection	Restricted	OBIS (2019) [iziko South African Museum - Fish Collection] (Available: Ocean Biodiversity Information System. Intergovernmental Oceanographic Commission of UNESCO. https://obis.org. Accessed: 2019-08-29)

a4f7ee48-0d0b-4c05-a972-27a43b30db58	Institution MCM - Collection DEM	No available dataset citation	Unspecified intellectual rights	OBIS (2019) [Institution MCM - Collection DEM] (Available: Ocean Biodiversity Information System. Intergovernmental Oceanographic Commission of UNESCO. https://obis.org. Accessed: 2019-08-29)
b0a7add2-dd9e-4020-9ca4-5df048c8f6a2	Bigood	Fabri, M-C. et al., Ifremer BIOCEAN database (Deep Sea Benthic Fauna). Institut Français de Recherche pour l'Exploitation de la Mer, Ifremer, Issy-les-Moulineaux, France. World Wide Web electronic publication, http://www.ifremer.fr/isi/biocean	This work is licensed under a Creative Commons Attribution (CC-BY) 4.0 License	OBIS (2019) [Bigood] (Available: Ocean Biodiversity Information System. Intergovernmental Oceanographic Commission of UNESCO. https://obis.org. Accessed: 2019-08-29)
b8617377-eb1c-4db2-baa6-8788a632e810	Ichthyology Collection - Royal Ontario Museum	NA	This work is licensed under a Creative Commons Attribution Non Commercial (CC-BY-NC) 4.0 License	OBIS (2019) [Ichthyology Collection - Royal Ontario Museum] (Available: Ocean Biodiversity Information System. Intergovernmental Oceanographic Commission of UNESCO. https://obis.org. Accessed: 2019-08-29)
c24bf1c2-2c62-4056-a841-56d94e6e876a	Fish specimens	ROM Fish Collection (accessed through GBIF data portal, http://data.gbif.org/datasets/resource/660, 2012-01-20) http://doi.org/10.15468/syisbx	Unrestricted	OBIS (2019) [Fish specimens] (Available: Ocean Biodiversity Information System. Intergovernmental Oceanographic Commission of UNESCO. https://obis.org. Accessed: 2019-08-29)
cc8f28ce-e48d-4945-abfe-9d150a22dcd6	Hamburg pelagic fish database	Post, A. 1987. Pelagic transects of FRVs "Walther Herwig" and "Anton Dohrn" in the Atlantic Ocean 1966 to 1986. Mitt. Inst. f. Seefischerei d. BfaFi Hamburg, 42: 1-68.	This work is licensed under a Creative Commons Attribution (CC-BY) 4.0 License	OBIS (2019) [Hamburg pelagic fish database] (Available: Ocean Biodiversity Information System. Intergovernmental Oceanographic Commission of UNESCO. https://obis.org. Accessed: 2019-08-29)
ce1d93f3-8b0f-4ee7-9a4d-0393a6ec7fea	Atlantic Reference Centre Museum of Canadian Atlantic Organisms - Invertebrates and Fishes Data	Van Guelpen, L., 2016. Atlantic Reference Centre Museum of Canadian Atlantic Organisms - Invertebrates and Fishes Data. Version 4 In OBIS Canada Digital Collections. Bedford Institute of Oceanography, Dartmouth, NS, Canada. Published by OBIS, Digital http://www.iobis.org/. Accessed on –INSERT DATE	This work is licensed under a Creative Commons Attribution (CC-BY) 4.0 License	OBIS (2019) [Atlantic Reference Centre Museum of Canadian Atlantic Organisms - Invertebrates and Fishes Data] (Available: Ocean Biodiversity Information System. Intergovernmental Oceanographic Commission of UNESCO. https://obis.org. Accessed: 2019-08-29)

cfc56587-48c3-4e3d-9350-3a4d9a28b681	Institution NOAA, NMFS, Northeast Fisheries Science Center - Collection DEEPWATER SYSTEMATICS	No available dataset citation	Unspecified intellectual rights	OBIS (2019) [Institution NOAA, NMFS, Northeast Fisheries Science Center - Collection DEEPWATER SYSTEMATICS] (Available: Ocean Biodiversity Information System. Intergovernmental Oceanographic Commission of UNESCO. https://obis.org. Accessed: 2019-08-29)
d6d6fe4c-425f-4ce7-bf28-7a6befaeb413	National Museum of Natural History Vertebrate Zoology Fishes Collections	National Museum of Natural History, Smithsonian Institution NMNH Fishes Collection Database. National Museum of Natural History, Smithsonian Institution, 10th and Constitution Ave. N.W., Washington, DC 20560-0193, 2007.	This work is licensed under a Creative Commons Attribution (CC-BY) 4.0 License	OBIS (2019) [National Museum of Natural History Vertebrate Zoology Fishes Collections] (Available: Ocean Biodiversity Information System. Intergovernmental Oceanographic Commission of UNESCO. https://obis.org. Accessed: 2019-08-29)

Notoscopelus resplendens

GBIF

GBIF Occurrence Download https://doi.org/10.15468/dd.8ypwgt Accessed from R via rgbif (https://github.com/ropensci/rgbif) on 2019-09-11

OBIS

OBIS (2019) Distribution records of Notoscopelus resplendens [Dataset] (Available: Ocean Biodiversity Information System. Intergovernmental Oceanographic Commission of UNESCO. www.obis.org. Accessed: 2019-08-29)

Dataset details:

Dataset_ID	Name	Citation	License	OBIS_citation

0332e1b5-5525-4301-9659-ef3da3e4e2b6	MARMAP Isaacs-Kidd Midwater Trawl 1990-2009	Marcel Reichert, 2010, MARMAP Isaacs-Kidd Midwater Trawl 1990-2009, SCDNR/NOAA MARMAP Program, SCDNR MARMAP Aggregate data surveys, The Marine Resources Monitoring, Assessment, and Prediction (MARMAP) Program, Marine Resources Research Institute, South Carolina Department of Natural Resources, P. O. Box 12559, Charleston SC 29422-2559, U.S.A.Retrieved from http://www.usgs.gov/obis-usa/	Restricted	OBIS (2019) [MARMAP Isaacs-Kidd Midwater Trawl 1990-2009] (Available: Ocean Biodiversity Information System. Intergovernmental Oceanographic Commission of UNESCO. https://obis.org. Accessed: 2019-08-29)
10b213e6-a9c4-459e-a40c-ef9edc461b97	Marine data from the Bernice P. Bishop Museum	Pyle R (2016). Bernice P. Bishop Museum. Version 8.1. Bernice Pauahi Bishop Museum. Occurrence dataset https://doi.org/10.15468/s6ctus accessed via GBIF.org on 2018-11-16.	To the extent possible under law, the publisher has waived all rights to these data and has dedicated them to the Public Domain (CC0 1.0)	OBIS (2019) [Marine data from the Bernice P. Bishop Museum] (Available: Ocean Biodiversity Information System. Intergovernmental Oceanographic Commission of UNESCO. https://obis.org. Accessed: 2019-08-29)
1f59030f-f116-4c34-915e-1882d819cda3	Institution Southampton Oceanography Ceter - Collection discovery	No available dataset citation	Unspecified intellectual rights	OBIS (2019) [Institution Southampton Oceanography Ceter - Collection discovery] (Available: Ocean Biodiversity Information System. Intergovernmental Oceanographic Commission of UNESCO. https://obis.org. Accessed: 2019-08-29)
2870c548-343e-4575-ac67-a4da35182c52	Institution Shirshov Institute - Collection SKAO	No available dataset citation	Unspecified intellectual rights	OBIS (2019) [Institution Shirshov Institute - Collection SKAO] (Available: Ocean Biodiversity Information System. Intergovernmental Oceanographic Commission of UNESCO. https://obis.org. Accessed: 2019-08-29)
3d922162-062c-4ad2-bf4a-f2493bd3a95d	Institution Bedford Institute of Oceanography (BIO) - Collection SUMMER	No available dataset citation	Unspecified intellectual rights	OBIS (2019) [Institution Bedford Institute of Oceanography (BIO) - Collection SUMMER] (Available: Ocean Biodiversity Information System. Intergovernmental Oceanographic Commission of UNESCO. https://obis.org. Accessed: 2019-08-29)

4354345d-7faf-4376-b326-ffbc04b6b0cd	No available dataset name	No available dataset citation	Unspecified intellectual rights	OBIS (2019) [No available dataset name] (Available: Ocean Biodiversity Information System. Intergovernmental Oceanographic Commission of UNESCO. https://obis.org. Accessed: 2019-08-29)
4bdc1f6f-e16a-48b4-b995-b51bd41caa8d	Dataset of the multidisciplinary research surveys in the seamounts of Ewing and Valdivia Bank (Walvis Ridge) - SE Atlantic	López-Abellán, L. J.; Sarralde Vizuete, R.; González Jiménez, J. F.; Centro Oceanográfico de Canarias – IEO, Spain (2015). Dataset of the multidisciplinary research surveys in the seamounts of Ewing and Valdivia Bank (Walvis Ridge) - SE Atlantic https://dx.doi.org/10.14284/58	Attribution-NoDerivatives (CC BY-ND)	OBIS (2019) [Dataset of the multidisciplinary research surveys in the seamounts of Ewing and Valdivia Bank (Walvis Ridge) - SE Atlantic] (Available: Ocean Biodiversity Information System. Intergovernmental Oceanographic Commission of UNESCO. https://obis.org. Accessed: 2019-08-29)
5f2da252-6d49-4c9f-b3b3-1db53d75b345	MARMAP Bongo Nets 1990-2009	Marcel Reichert, 2010, MARMAP Bongo Nets 1990-2009, SCDNR/NOAA MARMAP Program, SCDNR MARMAP Aggregate data surveys, The Marine Resources Monitoring, Assessment, and Prediction (MARMAP) Program, Marine Resources Research Institute, South Carolina Department of Natural Resources, P. O. Box 12559, Charleston SC 29422-2559, U.S.A.Retrieved from http://www.usgs.gov/obis-usa/	Restricted	OBIS (2019) [MARMAP Bongo Nets 1990-2009] (Available: Ocean Biodiversity Information System. Intergovernmental Oceanographic Commission of UNESCO. https://obis.org. Accessed: 2019-08-29)
6a5bc28f-4dfe-4cbf-8a55-7e3a843997ab	SPC NECTALIS Zooplankton/Micronekton specimens, New Caledonia 2014	Allain, V., Menkes, C., 2014. Nectalis 3 cruise, RV Alis. https://doi.org/10.17600/14004900	This work is licensed under a Creative Commons Attribution Non Commercial (CC-BY-NC) 4.0 License	OBIS (2019) [SPC NECTALIS Zooplankton/Micronekton specimens, New Caledonia 2014] (Available: Ocean Biodiversity Information System. Intergovernmental Oceanographic Commission of UNESCO. https://obis.org. Accessed: 2019-08-29)
6c19184e-c305-4273-8890-6d342d86f865	Institution - Collection	No available dataset citation	Unspecified intellectual rights	OBIS (2019) [Institution - Collection] (Available: Ocean Biodiversity Information System. Intergovernmental Oceanographic Commission of UNESCO. https://obis.org. Accessed: 2019-08-29)

705770e5-3474-4e69-be8b-3107a0c5610a	The fishes collection (IC) of the Muséum national d'Histoire naturelle (MNHN - Paris)	Gicim data base, Pruvost P. Causse R., 2009 http://doi.org/10.15468/tm7whu	This work is licensed under a Creative Commons Attribution (CC-BY) 4.0 License	OBIS (2019) [The fishes collection (IC) of the Muséum national d'Histoire naturelle (MNHN - Paris)] (Available: Ocean Biodiversity Information System. Intergovernmental Oceanographic Commission of UNESCO. https://obis.org. Accessed: 2019-08-29)
784c3f00-9b0b-4b2d-a0e7-2de304537f8b	Institution REVIZEE - Collection Pelagic Fishes	No available dataset citation	Unspecified intellectual rights	OBIS (2019) [Institution REVIZEE - Collection Pelagic Fishes] (Available: Ocean Biodiversity Information System. Intergovernmental Oceanographic Commission of UNESCO. https://obis.org. Accessed: 2019-08-29)
87a421bf-4646-49e3-89b7-409b93f2ac7c	Institution REVIZEE - Collection Pelagic Fishes	No available dataset citation	Unspecified intellectual rights	OBIS (2019) [Institution REVIZEE - Collection Pelagic Fishes] (Available: Ocean Biodiversity Information System. Intergovernmental Oceanographic Commission of UNESCO. https://obis.org. Accessed: 2019-08-29)
8a1ae661-e911-4967-bc06-1168fc5f2d89	iziko South African Museum - Fish Collection	iziko South African Museum - Fish Collection	Restricted	OBIS (2019) [iziko South African Museum - Fish Collection] (Available: Ocean Biodiversity Information System. Intergovernmental Oceanographic Commission of UNESCO. https://obis.org. Accessed: 2019-08-29)
9ff216fc-777e-4f9b-9860-95ed7366870d	Institution TU - Collection Fish	No available dataset citation	Unspecified intellectual rights	OBIS (2019) [Institution TU - Collection Fish] (Available: Ocean Biodiversity Information System. Intergovernmental Oceanographic Commission of UNESCO. https://obis.org. Accessed: 2019-08-29)
9ff216fc-777e-4f9b-9860-95ed7366870d	Institution UWFC - Collection ADULT COLLECTION	No available dataset citation	Unspecified intellectual rights	OBIS (2019) [Institution UWFC - Collection ADULT COLLECTION] (Available: Ocean Biodiversity Information System. Intergovernmental Oceanographic Commission of UNESCO. https://obis.org. Accessed: 2019-08-29)
a4f7ee48-0d0b-4c05-a972-27a43b30db58	Institution SAIAB - Collection SAIAB	No available dataset citation	Unspecified intellectual rights	OBIS (2019) [Institution SAIAB - Collection SAIAB] (Available: Ocean Biodiversity Information System. Intergovernmental Oceanographic Commission of UNESCO. https://obis.org. Accessed: 2019-08-29)

b8617377-eb1c-4db2-baa6-8788a632e810	Ichthyology Collection - Royal Ontario Museum	NA	This work is licensed under a Creative Commons Attribution Non Commercial (CC-BY-NC) 4.0 License	OBIS (2019) [Ichthyology Collection - Royal Ontario Museum] (Available: Ocean Biodiversity Information System. Intergovernmental Oceanographic Commission of UNESCO. https://obis.org. Accessed: 2019-08-29)
c24bf1c2-2c62-4056-a841-56d94e6e876a	Fish specimens	ROM Fish Collection (accessed through GBIF data portal, http://data.gbif.org/datasets/resource/660, 2012-01-20) http://doi.org/10.15468/syisbx	Unrestricted	OBIS (2019) [Fish specimens] (Available: Ocean Biodiversity Information System. Intergovernmental Oceanographic Commission of UNESCO. https://obis.org. Accessed: 2019-08-29)
cc8f28ce-e48d-4945-abfe-9d150a22dcd6	Hamburg pelagic fish database	Post, A. 1987. Pelagic transects of FRVs "Walther Herwig" and "Anton Dohrn" in the Atlantic Ocean 1966 to 1986. Mitt. Inst. f. Seefischerei d. BfaFi Hamburg, 42: 1-68.	This work is licensed under a Creative Commons Attribution (CC-BY) 4.0 License	OBIS (2019) [Hamburg pelagic fish database] (Available: Ocean Biodiversity Information System. Intergovernmental Oceanographic Commission of UNESCO. https://obis.org. Accessed: 2019-08-29)
ce1d93f3-8b0f-4ee7-9a4d-0393a6ec7fea	Atlantic Reference Centre Museum of Canadian Atlantic Organisms - Invertebrates and Fishes Data	Van Guelpen, L., 2016. Atlantic Reference Centre Museum of Canadian Atlantic Organisms - Invertebrates and Fishes Data. Version 4 In OBIS Canada Digital Collections. Bedford Institute of Oceanography, Dartmouth, NS, Canada. Published by OBIS, Digital http://www.iobis.org/. Accessed on –INSERT DATE	This work is licensed under a Creative Commons Attribution (CC-BY) 4.0 License	OBIS (2019) [Atlantic Reference Centre Museum of Canadian Atlantic Organisms - Invertebrates and Fishes Data] (Available: Ocean Biodiversity Information System. Intergovernmental Oceanographic Commission of UNESCO. https://obis.org. Accessed: 2019-08-29)
cfc56587-48c3-4e3d-9350-3a4d9a28b681	Institution SAIAB - Collection SAIAB	No available dataset citation	Unspecified intellectual rights	OBIS (2019) [Institution SAIAB - Collection SAIAB] (Available: Ocean Biodiversity Information System. Intergovernmental Oceanographic Commission of UNESCO. https://obis.org. Accessed: 2019-08-29)
cfc56587-48c3-4e3d-9350-3a4d9a28b681	Institution MCM - Collection DEM	No available dataset citation	Unspecified intellectual rights	OBIS (2019) [Institution MCM - Collection DEM] (Available: Ocean Biodiversity Information System. Intergovernmental Oceanographic Commission of UNESCO. https://obis.org. Accessed: 2019-08-29)

cfc56587-48c3-4e3d-9350-3a4d9a28b681	Institution NOAA, NMFS, Northeast Fisheries Science Center - Collection DEEPWATER SYSTEMATICS	No available dataset citation	Unspecified intellectual rights	OBIS (2019) [Institution NOAA, NMFS, Northeast Fisheries Science Center - Collection DEEPWATER SYSTEMATICS] (Available: Ocean Biodiversity Information System. Intergovernmental Oceanographic Commission of UNESCO. https://obis.org. Accessed: 2019-08-29)
d6d6fe4c-425f-4ce7-bf28-7a6befaeb413	National Museum of Natural History Vertebrate Zoology Fishes Collections	National Museum of Natural History, Smithsonian Institution NMNH Fishes Collection Database. National Museum of Natural History, Smithsonian Institution, 10th and Constitution Ave. N.W., Washington, DC 20560-0193, 2007.	This work is licensed under a Creative Commons Attribution (CC-BY) 4.0 License	OBIS (2019) [National Museum of Natural History Vertebrate Zoology Fishes Collections] (Available: Ocean Biodiversity Information System. Intergovernmental Oceanographic Commission of UNESCO. https://obis.org. Accessed: 2019-08-29)
ff8b7809-41bc-40ad-8160-0e33862817a0	Biodiversity Research Museum, Academia Sinica, Taiwan	TELDAP, Biodiversity Research Museum, Academia Sinica, Taiwan (accessed through GBIF data portal, http://data.gbif.org/datasets/resource/9093, yyyy-mm-dd)	Unrestricted	OBIS (2019) [Biodiversity Research Museum, Academia Sinica, Taiwan] (Available: Ocean Biodiversity Information System. Intergovernmental Oceanographic Commission of UNESCO. https://obis.org. Accessed: 2019-08-29)

Lampanyctus ater

GBIF

GBIF Occurrence Download https://doi.org/10.15468/dd.svwv5p Accessed from R via rgbif (https://github.com/ropensci/rgbif) on 2019-09-11

Electrona risso

GBIF

GBIF Occurrence Download https://doi.org/10.15468/dd.q3uxbf Accessed from R via rgbif (https://github.com/ropensci/rgbif) on 2019-09-11

OBIS

OBIS (2019) Distribution records of Electrona risso [Dataset] (Available: Ocean Biodiversity Information System. Intergovernmental Oceanographic Commission of UNESCO. www.obis.org. Accessed: 2019-08-29)

Dataset details:

Dataset_ID	Name	Citation	License	OBIS_citation
10b213e6-a9c4-459e-a40c-ef9edc461b97	Marine data from the Bernice P. Bishop Museum	Pyle R (2016). Bernice P. Bishop Museum. Version 8.1. Bernice Pauahi Bishop Museum. Occurrence dataset https://doi.org/10.15468/s6ctus accessed via GBIF.org on 2018-11-16.	To the extent possible under law, the publisher has waived all rights to these data and has dedicated them to the Public Domain (CC0 1.0)	OBIS (2019) [Marine data from the Bernice P. Bishop Museum] (Available: Ocean Biodiversity Information System. Intergovernmental Oceanographic Commission of UNESCO. https://obis.org. Accessed: 2019-08-29)
1f59030f-f116-4c34-915e-1882d819cda3	Institution Southampton Oceanography Ceter - Collection discovery	No available dataset citation	Unspecified intellectual rights	OBIS (2019) [Institution Southampton Oceanography Ceter - Collection discovery] (Available: Ocean Biodiversity Information System. Intergovernmental Oceanographic Commission of UNESCO. https://obis.org. Accessed: 2019-08-29)
2870c548-343e-4575-ac67-a4da35182c52	Institution Shirshov Institute - Collection SKAO	No available dataset citation	Unspecified intellectual rights	OBIS (2019) [Institution Shirshov Institute - Collection SKAO] (Available: Ocean Biodiversity Information System. Intergovernmental Oceanographic Commission of UNESCO. https://obis.org. Accessed: 2019-08-29)
4bdc1f6f-e16a-48b4-b995-b51bd41caa8d	Dataset of the multidisciplinary research surveys in the seamounts of Ewing and Valdivia Bank (Walvis Ridge) - SE Atlantic	López-Abellán, L. J.; Sarralde Vizuete, R.; González Jiménez, J. F.; Centro Oceanográfico de Canarias – IEO, Spain (2015). Dataset of the multidisciplinary research surveys in the seamounts of Ewing and Valdivia Bank (Walvis Ridge) - SE Atlantic https://dx.doi.org/10.14284/58	Attribution-NoDerivatives (CC BY-ND)	OBIS (2019) [Dataset of the multidisciplinary research surveys in the seamounts of Ewing and Valdivia Bank (Walvis Ridge) - SE Atlantic] (Available: Ocean Biodiversity Information System. Intergovernmental Oceanographic Commission of UNESCO. https://obis.org. Accessed: 2019-08-29)
623f4f98-4e66-4bee-9e1c-17cb74cd2d21	Institution IEO - Collection MEDITS-Spain	No available dataset citation	Unspecified intellectual rights	OBIS (2019) [Institution IEO - Collection MEDITS-Spain] (Available: Ocean Biodiversity Information System. Intergovernmental Oceanographic Commission of UNESCO. https://obis.org. Accessed: 2019-08-29)

6a5bc28f-4dfe-4cbf-8a55-7e3a843997ab	SPC NECTALIS Zooplankton/Micronekton specimens, New Caledonia 2014	Allain, V., Menkes, C., 2014. Nectalis 3 cruise, RV Alis. https://doi.org/10.17600/14004900	This work is licensed under a Creative Commons Attribution Non Commercial (CC-BY-NC) 4.0 License	OBIS (2019) [SPC NECTALIS Zooplankton/Micronekton specimens, New Caledonia 2014] (Available: Ocean Biodiversity Information System. Intergovernmental Oceanographic Commission of UNESCO. https://obis.org. Accessed: 2019-08-29)
705770e5-3474-4e69-be8b-3107a0c5610a	The fishes collection (IC) of the Muséum national d'Histoire naturelle (MNHN - Paris)	Gicim data base, Pruvost P. Causse R., 2009 http://doi.org/10.15468/tm7whu	This work is licensed under a Creative Commons Attribution (CC-BY) 4.0 License	OBIS (2019) [The fishes collection (IC) of the Muséum national d'Histoire naturelle (MNHN - Paris)] (Available: Ocean Biodiversity Information System. Intergovernmental Oceanographic Commission of UNESCO. https://obis.org. Accessed: 2019-08-29)
8629ec33-be4b-4384-933f-a511fbc29967	MAR-ECO 2004	Wenneck, T. de Lange, Falkenhaug, T. and O.A. Bergstad. 2008. Strategies, methods, and technologies adopted on the RV G.O. Sars MAR-ECO expedition to the mid-Atlantic Ridge in 2004. Deep-sea Research II. 55: 6-28.	This work is licensed under a Creative Commons Attribution (CC-BY) 4.0 License	OBIS (2019) [MAR-ECO 2004] (Available: Ocean Biodiversity Information System. Intergovernmental Oceanographic Commission of UNESCO. https://obis.org. Accessed: 2019-08-29)
8843341c-ddd1-47a0-b4e6-5f37eec9b317	Institution AADC - Collection Historic_Fish	No available dataset citation	Unspecified intellectual rights	OBIS (2019) [Institution AADC - Collection Historic_Fish] (Available: Ocean Biodiversity Information System. Intergovernmental Oceanographic Commission of UNESCO. https://obis.org. Accessed: 2019-08-29)
8a1ae661-e911-4967-bc06-1168fc5f2d89	iziko South African Museum - Fish Collection	iziko South African Museum - Fish Collection	Restricted	OBIS (2019) [iziko South African Museum - Fish Collection] (Available: Ocean Biodiversity Information System. Intergovernmental Oceanographic Commission of UNESCO. https://obis.org. Accessed: 2019-08-29)
9ff216fc-777e-4f9b-9860-95ed7366870d	Institution SAIAB - Collection SAIAB	No available dataset citation	Unspecified intellectual rights	OBIS (2019) [Institution SAIAB - Collection SAIAB] (Available: Ocean Biodiversity Information System. Intergovernmental Oceanographic Commission of UNESCO. https://obis.org. Accessed: 2019-08-29)

a4f7ee48-0d0b-4c05-a972-27a43b30db58	Institution MCM - Collection DEM	No available dataset citation	Unspecified intellectual rights	OBIS (2019) [Institution MCM - Collection DEM] (Available: Ocean Biodiversity Information System. Intergovernmental Oceanographic Commission of UNESCO. https://obis.org. Accessed: 2019-08-29)
a6f8db9e-2794-4260-b7f6-66febc029002	Trawl survey data from the Jabuka Pit area (central-eastern Adriatic Sea, Mediterranean) collected between 1956 and 1971	National Institute of Oceanography and Experimental Geophysics (OGS); Italian National Institute for Environmental Protection and Research (ISPRA), Italy; (2017): Trawl survey data from the Jabuka Pit area (central-eastern Adriatic Sea, Mediterranean) collected between 1956 and 1971. https://dx.doi.org/10.14284/287	This work is licensed under a Creative Commons Attribution (CC-BY) 4.0 License	OBIS (2019) [Trawl survey data from the Jabuka Pit area (central-eastern Adriatic Sea, Mediterranean) collected between 1956 and 1971] (Available: Ocean Biodiversity Information System. Intergovernmental Oceanographic Commission of UNESCO. https://obis.org. Accessed: 2019-08-29)
b8617377-eb1c-4db2-baa6-8788a632e810	Ichthyology Collection - Royal Ontario Museum	NA	This work is licensed under a Creative Commons Attribution Non Commercial (CC-BY-NC) 4.0 License	OBIS (2019) [Ichthyology Collection - Royal Ontario Museum] (Available: Ocean Biodiversity Information System. Intergovernmental Oceanographic Commission of UNESCO. https://obis.org. Accessed: 2019-08-29)
cc8f28ce-e48d-4945-abfe-9d150a22dcd6	Hamburg pelagic fish database	Post, A. 1987. Pelagic transects of FRVs "Walther Herwig" and "Anton Dohrn" in the Atlantic Ocean 1966 to 1986. Mitt. Inst. f. Seefischerei d. BfaFi Hamburg, 42: 1-68.	This work is licensed under a Creative Commons Attribution (CC-BY) 4.0 License	OBIS (2019) [Hamburg pelagic fish database] (Available: Ocean Biodiversity Information System. Intergovernmental Oceanographic Commission of UNESCO. https://obis.org. Accessed: 2019-08-29)
ce1d93f3-8b0f-4ee7-9a4d-0393a6ec7fea	Atlantic Reference Centre Museum of Canadian Atlantic Organisms - Invertebrates and Fishes Data	Van Guelpen, L., 2016. Atlantic Reference Centre Museum of Canadian Atlantic Organisms - Invertebrates and Fishes Data. Version 4 In OBIS Canada Digital Collections. Bedford Institute of Oceanography, Dartmouth, NS, Canada. Published by OBIS, Digital http://www.iobis.org/. Accessed on –INSERT DATE	This work is licensed under a Creative Commons Attribution (CC-BY) 4.0 License	OBIS (2019) [Atlantic Reference Centre Museum of Canadian Atlantic Organisms - Invertebrates and Fishes Data] (Available: Ocean Biodiversity Information System. Intergovernmental Oceanographic Commission of UNESCO. https://obis.org. Accessed: 2019-08-29)

d6d6fe4c-425f-4ce7-bf28-7a6befaeb413	National Museum of Natural History Vertebrate Zoology Fishes Collections	National Museum of Natural History, Smithsonian Institution NMNH Fishes Collection Database. National Museum of Natural History, Smithsonian Institution, 10th and Constitution Ave. N.W., Washington, DC 20560-0193, 2007.	This work is licensed under a Creative Commons Attribution (CC-BY) 4.0 License	OBIS (2019) [National Museum of Natural History Vertebrate Zoology Fishes Collections] (Available: Ocean Biodiversity Information System. Intergovernmental Oceanographic Commission of UNESCO. https://obis.org. Accessed: 2019-08-29)
e09c824c-cbd8-4529-b382-5306b2b3a875	Marine biodiversity atlas of the Balearic Sea	Deudero, Vallespir, Obrador 2011. Atlas de Biodiversidad Marina del Mar Balear. http://www.ba.ieo.es	This work is licensed under a Creative Commons Attribution Non Commercial (CC-BY-NC) 4.0 License	OBIS (2019) [Marine biodiversity atlas of the Balearic Sea] (Available: Ocean Biodiversity Information System. Intergovernmental Oceanographic Commission of UNESCO. https://obis.org. Accessed: 2019-08-29)
ff8b7809-41bc-40ad-8160-0e33862817a0	Biodiversity Research Museum, Academia Sinica, Taiwan	TELDAP, Biodiversity Research Museum, Academia Sinica, Taiwan (accessed through GBIF data portal, http://data.gbif.org/datasets/resource/9093, yyyy-mm-dd)	Unrestricted	OBIS (2019) [Biodiversity Research Museum, Academia Sinica, Taiwan] (Available: Ocean Biodiversity Information System. Intergovernmental Oceanographic Commission of UNESCO. https://obis.org. Accessed: 2019-08-29)

Bolinichthys supralateralis

GBIF

GBIF Occurrence Download https://doi.org/10.15468/dd.twqxqu Accessed from R via rgbif (https://github.com/ropensci/rgbif) on 2019-09-11

OBIS

OBIS (2019) Distribution records of Bolinichthys supralateralis [Dataset] (Available: Ocean Biodiversity Information System. Intergovernmental Oceanographic Commission of UNESCO. www.obis.org. Accessed: 2019-08-29)

Dataset details:

Dataset_ID	Name	Citation	License	OBIS_citation

10b213e6-a9c4-459e-a40c-ef9edc461b97	Marine data from the Bernice P. Bishop Museum	Pyle R (2016). Bernice P. Bishop Museum. Version 8.1. Bernice Pauahi Bishop Museum. Occurrence dataset https://doi.org/10.15468/s6ctus accessed via GBIF.org on 2018-11-16.	To the extent possible under law, the publisher has waived all rights to these data and has dedicated them to the Public Domain (CC0 1.0)	OBIS (2019) [Marine data from the Bernice P. Bishop Museum] (Available: Ocean Biodiversity Information System. Intergovernmental Oceanographic Commission of UNESCO. https://obis.org. Accessed: 2019-08-29)
1f59030f-f116-4c34-915e-1882d819cda3	Institution Southampton Oceanography Ceter - Collection discovery	No available dataset citation	Unspecified intellectual rights	OBIS (2019) [Institution Southampton Oceanography Ceter - Collection discovery] (Available: Ocean Biodiversity Information System. Intergovernmental Oceanographic Commission of UNESCO. https://obis.org. Accessed: 2019-08-29)
4bdc1f6f-e16a-48b4-b995-b51bd41caa8d	Dataset of the multidisciplinary research surveys in the seamounts of Ewing and Valdivia Bank (Walvis Ridge) - SE Atlantic	López-Abellán, L. J.; Sarralde Vizuete, R.; González Jiménez, J. F.; Centro Oceanográfico de Canarias – IEO, Spain (2015). Dataset of the multidisciplinary research surveys in the seamounts of Ewing and Valdivia Bank (Walvis Ridge) - SE Atlantic https://dx.doi.org/10.14284/58	Attribution-NoDerivatives (CC BY-ND)	OBIS (2019) [Dataset of the multidisciplinary research surveys in the seamounts of Ewing and Valdivia Bank (Walvis Ridge) - SE Atlantic] (Available: Ocean Biodiversity Information System. Intergovernmental Oceanographic Commission of UNESCO. https://obis.org. Accessed: 2019-08-29)
513e4437-9cc7-4383-9a69-1bd18ec2046a	MARMAP Neuston Nets 1990-2009	Marcel Reichert, 2010, MARMAP Neuston Nets 1990-2009, SCDNR/NOAA MARMAP Program, SCDNR MARMAP Aggregate data surveys, The Marine Resources Monitoring, Assessment, and Prediction (MARMAP) Program, Marine Resources Research Institute, South Carolina Department of Natural Resources, P. O. Box 12559, Charleston SC 29422-2559, U.S.A.Retrieved from http://www.usgs.gov/obis-usa/	Restricted	OBIS (2019) [MARMAP Neuston Nets 1990-2009] (Available: Ocean Biodiversity Information System. Intergovernmental Oceanographic Commission of UNESCO. https://obis.org. Accessed: 2019-08-29)

5533fa1e-d1a6-47dc-b93e-bc51e2692589	MAR-ECO 2003 - Arni Fridriksson	Hafsteinn G. Gudfinnson, Høgni Debes, Tone Falkenhaug, Eilif Gaard, Ástthor Gislason, Hildur Petursdottir, Thorsteinn Sigurdsson, and Hedinn Valdimarsson. 2008. Abundance and productivity of the pelagic ecosystem along a transect across the northern Mid- Atlantic Ridge in June 2003. ICES CM 2008/C:12	This work is licensed under a Creative Commons Attribution (CC-BY) 4.0 License	OBIS (2019) [MAR-ECO 2003 - Arni Fridriksson] (Available: Ocean Biodiversity Information System. Intergovernmental Oceanographic Commission of UNESCO. https://obis.org. Accessed: 2019-08-29)
685b3956-c37a-433a-b661-2bb7b11cf9f8	Soviet Trawl Fishery Data (New Zealand Waters) 1964-1987	Ministry for Primary Industries (2014). Soviet Fishery Data (New Zealand Waters) 1964-1987. Southwestern Pacific OBIS, National Institute of Water and Atmospheric Research (NIWA), Wellington, New Zealand, 111883 records, Online http://nzobisipt.niwa.co.nz/resource.do?r=mbis_soviettrawl released on November 5, 2014.	This work is licensed under a Creative Commons Attribution (CC-BY) 4.0 License	OBIS (2019) [Soviet Trawl Fishery Data (New Zealand Waters) 1964-1987] (Available: Ocean Biodiversity Information System. Intergovernmental Oceanographic Commission of UNESCO. https://obis.org. Accessed: 2019-08-29)
784c3f00-9b0b-4b2d-a0e7-2de304537f8b	Institution TU - Collection Fish	No available dataset citation	Unspecified intellectual rights	OBIS (2019) [Institution TU - Collection Fish] (Available: Ocean Biodiversity Information System. Intergovernmental Oceanographic Commission of UNESCO. https://obis.org. Accessed: 2019-08-29)
8629ec33-be4b-4384-933f-a511fbc29967	MAR-ECO 2004	Wenneck, T. de Lange, Falkenhaug, T. and O.A. Bergstad. 2008. Strategies, methods, and technologies adopted on the RV G.O. Sars MAR-ECO expedition to the mid-Atlantic Ridge in 2004. Deep-sea Research II. 55: 6-28.	This work is licensed under a Creative Commons Attribution (CC-BY) 4.0 License	OBIS (2019) [MAR-ECO 2004] (Available: Ocean Biodiversity Information System. Intergovernmental Oceanographic Commission of UNESCO. https://obis.org. Accessed: 2019-08-29)
8a1ae661-e911-4967-bc06-1168fc5f2d89	iziko South African Museum - Fish Collection	iziko South African Museum - Fish Collection	Restricted	OBIS (2019) [iziko South African Museum - Fish Collection] (Available: Ocean Biodiversity Information System. Intergovernmental Oceanographic Commission of UNESCO. https://obis.org. Accessed: 2019-08-29)
9ff216fc-777e-4f9b-9860-95ed7366870d	Institution SAIAB - Collection SAIAB	No available dataset citation	Unspecified intellectual rights	OBIS (2019) [Institution SAIAB - Collection SAIAB] (Available: Ocean Biodiversity Information System. Intergovernmental Oceanographic Commission of UNESCO. https://obis.org. Accessed: 2019-08-29)

a4f7ee48-0d0b-4c05-a972-27a43b30db58	Institution MCM - Collection DEM	No available dataset citation	Unspecified intellectual rights	OBIS (2019) [Institution MCM - Collection DEM] (Available: Ocean Biodiversity Information System. Intergovernmental Oceanographic Commission of UNESCO. https://obis.org. Accessed: 2019-08-29)
ce1d93f3-8b0f-4ee7-9a4d-0393a6ec7fea	Atlantic Reference Centre Museum of Canadian Atlantic Organisms - Invertebrates and Fishes Data	Van Guelpen, L., 2016. Atlantic Reference Centre Museum of Canadian Atlantic Organisms - Invertebrates and Fishes Data. Version 4 In OBIS Canada Digital Collections. Bedford Institute of Oceanography, Dartmouth, NS, Canada. Published by OBIS, Digital http://www.iobis.org/. Accessed on –INSERT DATE	This work is licensed under a Creative Commons Attribution (CC-BY) 4.0 License	OBIS (2019) [Atlantic Reference Centre Museum of Canadian Atlantic Organisms - Invertebrates and Fishes Data] (Available: Ocean Biodiversity Information System. Intergovernmental Oceanographic Commission of UNESCO. https://obis.org. Accessed: 2019-08-29)
cfc56587-48c3-4e3d-9350-3a4d9a28b681	Institution NOAA, NMFS, Northeast Fisheries Science Center - Collection DEEPWATER SYSTEMATICS	No available dataset citation	Unspecified intellectual rights	OBIS (2019) [Institution NOAA, NMFS, Northeast Fisheries Science Center - Collection DEEPWATER SYSTEMATICS] (Available: Ocean Biodiversity Information System. Intergovernmental Oceanographic Commission of UNESCO. https://obis.org. Accessed: 2019-08-29)
d6d6fe4c-425f-4ce7-bf28-7a6befaeb413	National Museum of Natural History Vertebrate Zoology Fishes Collections	National Museum of Natural History, Smithsonian Institution NMNH Fishes Collection Database. National Museum of Natural History, Smithsonian Institution, 10th and Constitution Ave. N.W., Washington, DC 20560-0193, 2007.	This work is licensed under a Creative Commons Attribution (CC-BY) 4.0 License	OBIS (2019) [National Museum of Natural History Vertebrate Zoology Fishes Collections] (Available: Ocean Biodiversity Information System. Intergovernmental Oceanographic Commission of UNESCO. https://obis.org. Accessed: 2019-08-29)
ff8b7809-41bc-40ad-8160-0e33862817a0	Biodiversity Research Museum, Academia Sinica, Taiwan	TELDAP, Biodiversity Research Museum, Academia Sinica, Taiwan (accessed through GBIF data portal, http://data.gbif.org/datasets/resource/9093, yyyy-mm-dd)	Unrestricted	OBIS (2019) [Biodiversity Research Museum, Academia Sinica, Taiwan] (Available: Ocean Biodiversity Information System. Intergovernmental Oceanographic Commission of UNESCO. https://obis.org. Accessed: 2019-08-29)

Diaphus meadi

GBIF

GBIF Occurrence Download https://doi.org/10.15468/dd.q4x5hb Accessed from R via rgbif (https://github.com/ropensci/rgbif) on 2019-09-11

OBIS

OBIS (2019) Distribution records of Diaphus meadi [Dataset] (Available: Ocean Biodiversity Information System. Intergovernmental Oceanographic Commission of UNESCO. www.obis.org. Accessed: 2019-08-29)

Dataset details:

Dataset_ID	Name	Citation	License	OBIS_citation
2870c548-343e-4575-ac67-a4da35182c52	Institution Shirshov Institute - Collection SKAO	No available dataset citation	Unspecified intellectual rights	OBIS (2019) [Institution Shirshov Institute - Collection SKAO] (Available: Ocean Biodiversity Information System. Intergovernmental Oceanographic Commission of UNESCO. https://obis.org. Accessed: 2019-08-29)
8843341c-ddd1-47a0-b4e6-5f37eec9b317	Institution AADC - Collection Historic_Fish	No available dataset citation	Unspecified intellectual rights	OBIS (2019) [Institution AADC - Collection Historic_Fish] (Available: Ocean Biodiversity Information System. Intergovernmental Oceanographic Commission of UNESCO. https://obis.org. Accessed: 2019-08-29)
8a1ae661-e911-4967-bc06-1168fc5f2d89	iziko South African Museum - Fish Collection	iziko South African Museum - Fish Collection	Restricted	OBIS (2019) [iziko South African Museum - Fish Collection] (Available: Ocean Biodiversity Information System. Intergovernmental Oceanographic Commission of UNESCO. https://obis.org. Accessed: 2019-08-29)
9ff216fc-777e-4f9b-9860-95ed7366870d	Institution SAIAB - Collection SAIAB	No available dataset citation	Unspecified intellectual rights	OBIS (2019) [Institution SAIAB - Collection SAIAB] (Available: Ocean Biodiversity Information System. Intergovernmental Oceanographic Commission of UNESCO. https://obis.org. Accessed: 2019-08-29)
a4f7ee48-0d0b-4c05-a972-27a43b30db58	Institution MCM - Collection DEM	No available dataset citation	Unspecified intellectual rights	OBIS (2019) [Institution MCM - Collection DEM] (Available: Ocean Biodiversity Information System. Intergovernmental Oceanographic Commission of UNESCO. https://obis.org. Accessed: 2019-08-29)

d6d6fe4c-425f-4ce7-bf28-7a6befaeb413	National Museum of Natural History Vertebrate Zoology Fishes Collections	National Museum of Natural History, Smithsonian Institution NMNH Fishes Collection Database. National Museum of Natural History, Smithsonian Institution, 10th and Constitution Ave. N.W., Washington, DC 20560-0193, 2007.	This work is licensed under a Creative Commons Attribution (CC-BY) 4.0 License	OBIS (2019) [National Museum of Natural History Vertebrate Zoology Fishes Collections] (Available: Ocean Biodiversity Information System. Intergovernmental Oceanographic Commission of UNESCO. https://obis.org. Accessed: 2019-08-29)

Diaphus mollis

GBIF

GBIF Occurrence Download https://doi.org/10.15468/dd.d4rsb4 Accessed from R via rgbif (https://github.com/ropensci/rgbif) on 2019-09-11

OBIS

OBIS (2019) Distribution records of Diaphus mollis [Dataset] (Available: Ocean Biodiversity Information System. Intergovernmental Oceanographic Commission of UNESCO. www.obis.org. Accessed: 2019-08-29)

Dataset details:

Dataset_ID	Name	Citation	License	OBIS_citation
0332e1b5-5525-4301-9659-ef3da3e4e2b6	MARMAP Isaacs-Kidd Midwater Trawl 1990-2009	Marcel Reichert, 2010, MARMAP Isaacs-Kidd Midwater Trawl 1990-2009, SCDNR/NOAA MARMAP Program, SCDNR MARMAP Aggregate data surveys, The Marine Resources Monitoring, Assessment, and Prediction (MARMAP) Program, Marine Resources Research Institute, South Carolina Department of Natural Resources, P. O. Box 12559, Charleston SC 29422-2559, U.S.A.Retrieved from http://www.usgs.gov/obis-usa/	Restricted	OBIS (2019) [MARMAP Isaacs-Kidd Midwater Trawl 1990-2009] (Available: Ocean Biodiversity Information System. Intergovernmental Oceanographic Commission of UNESCO. https://obis.org. Accessed: 2019-08-29)

10b213e6-a9c4-459e-a40c-ef9edc461b97	Marine data from the Bernice P. Bishop Museum	Pyle R (2016). Bernice P. Bishop Museum. Version 8.1. Bernice Pauahi Bishop Museum. Occurrence dataset https://doi.org/10.15468/s6ctus accessed via GBIF.org on 2018-11-16.	To the extent possible under law, the publisher has waived all rights to these data and has dedicated them to the Public Domain (CC0 1.0)	OBIS (2019) [Marine data from the Bernice P. Bishop Museum] (Available: Ocean Biodiversity Information System. Intergovernmental Oceanographic Commission of UNESCO. https://obis.org. Accessed: 2019-08-29)
1f59030f-f116-4c34-915e-1882d819cda3	Institution Southampton Oceanography Ceter - Collection discovery	No available dataset citation	Unspecified intellectual rights	OBIS (2019) [Institution Southampton Oceanography Ceter - Collection discovery] (Available: Ocean Biodiversity Information System. Intergovernmental Oceanographic Commission of UNESCO. https://obis.org. Accessed: 2019-08-29)
270f3e70-ff9b-411d-b170-2bc914d83f26	Biological Reference Collections ICM CSIC	Olivas González F J (2016): Biological Reference Collections ICM CSIC. Institute of Marine Sciences (ICM-CSIC). https://dx.doi.org/10.15470/qlqqdx	This work is licensed under a Creative Commons Attribution Non Commercial (CC-BY-NC) 4.0 License	OBIS (2019) [Biological Reference Collections ICM CSIC] (Available: Ocean Biodiversity Information System. Intergovernmental Oceanographic Commission of UNESCO. https://obis.org. Accessed: 2019-08-29)
2870c548-343e-4575-ac67-a4da35182c52	Institution Shirshov Institute - Collection SKAO	No available dataset citation	Unspecified intellectual rights	OBIS (2019) [Institution Shirshov Institute - Collection SKAO] (Available: Ocean Biodiversity Information System. Intergovernmental Oceanographic Commission of UNESCO. https://obis.org. Accessed: 2019-08-29)
6a5bc28f-4dfe-4cbf-8a55-7e3a843997ab	SPC NECTALIS Zooplankton/Micronekton specimens, New Caledonia 2014	Allain, V., Menkes, C., 2014. Nectalis 3 cruise, RV Alis. https://doi.org/10.17600/14004900	This work is licensed under a Creative Commons Attribution Non Commercial (CC-BY-NC) 4.0 License	OBIS (2019) [SPC NECTALIS Zooplankton/Micronekton specimens, New Caledonia 2014] (Available: Ocean Biodiversity Information System. Intergovernmental Oceanographic Commission of UNESCO. https://obis.org. Accessed: 2019-08-29)
705770e5-3474-4e69-be8b-3107a0c5610a	The fishes collection (IC) of the Muséum national d'Histoire naturelle (MNHN - Paris)	Gicim data base, Pruvost P. Causse R., 2009 http://doi.org/10.15468/tm7whu	This work is licensed under a Creative Commons Attribution (CC-BY) 4.0 License	OBIS (2019) [The fishes collection (IC) of the Muséum national d'Histoire naturelle (MNHN - Paris)] (Available: Ocean Biodiversity Information System. Intergovernmental Oceanographic Commission of UNESCO. https://obis.org. Accessed: 2019-08-29)

8629ec33-be4b-4384-933f-a511fbc29967	MAR-ECO 2004	Wenneck, T. de Lange, Falkenhaug, T. and O.A. Bergstad. 2008. Strategies, methods, and technologies adopted on the RV G.O. Sars MAR-ECO expedition to the mid-Atlantic Ridge in 2004. Deep-sea Research II. 55: 6-28.	This work is licensed under a Creative Commons Attribution (CC-BY) 4.0 License	OBIS (2019) [MAR-ECO 2004] (Available: Ocean Biodiversity Information System. Intergovernmental Oceanographic Commission of UNESCO. https://obis.org. Accessed: 2019-08-29)
87a421bf-4646-49e3-89b7-409b93f2ac7c	Institution UWFC - Collection ADULT COLLECTION	No available dataset citation	Unspecified intellectual rights	OBIS (2019) [Institution UWFC - Collection ADULT COLLECTION] (Available: Ocean Biodiversity Information System. Intergovernmental Oceanographic Commission of UNESCO. https://obis.org. Accessed: 2019-08-29)
8a1ae661-e911-4967-bc06-1168fc5f2d89	iziko South African Museum - Fish Collection	iziko South African Museum - Fish Collection	Restricted	OBIS (2019) [iziko South African Museum - Fish Collection] (Available: Ocean Biodiversity Information System. Intergovernmental Oceanographic Commission of UNESCO. https://obis.org. Accessed: 2019-08-29)
b8617377-eb1c-4db2-baa6-8788a632e810	Ichthyology Collection - Royal Ontario Museum	NA	This work is licensed under a Creative Commons Attribution Non Commercial (CC-BY-NC) 4.0 License	OBIS (2019) [Ichthyology Collection - Royal Ontario Museum] (Available: Ocean Biodiversity Information System. Intergovernmental Oceanographic Commission of UNESCO. https://obis.org. Accessed: 2019-08-29)
c24bf1c2-2c62-4056-a841-56d94e6e876a	Fish specimens	ROM Fish Collection (accessed through GBIF data portal, http://data.gbif.org/datasets/resource/660, 2012-01-20) http://doi.org/10.15468/syisbx	Unrestricted	OBIS (2019) [Fish specimens] (Available: Ocean Biodiversity Information System. Intergovernmental Oceanographic Commission of UNESCO. https://obis.org. Accessed: 2019-08-29)
cc8f28ce-e48d-4945-abfe-9d150a22dcd6	Hamburg pelagic fish database	Post, A. 1987. Pelagic transects of FRVs "Walther Herwig" and "Anton Dohrn" in the Atlantic Ocean 1966 to 1986. Mitt. Inst. f. Seefischerei d. BfaFi Hamburg, 42: 1-68.	This work is licensed under a Creative Commons Attribution (CC-BY) 4.0 License	OBIS (2019) [Hamburg pelagic fish database] (Available: Ocean Biodiversity Information System. Intergovernmental Oceanographic Commission of UNESCO. https://obis.org. Accessed: 2019-08-29)

ce1d93f3-8b0f-4ee7-9a4d-0393a6ec7fea	Atlantic Reference Centre Museum of Canadian Atlantic Organisms - Invertebrates and Fishes Data	Van Guelpen, L., 2016. Atlantic Reference Centre Museum of Canadian Atlantic Organisms - Invertebrates and Fishes Data. Version 4 In OBIS Canada Digital Collections. Bedford Institute of Oceanography, Dartmouth, NS, Canada. Published by OBIS, Digital http://www.iobis.org/. Accessed on –INSERT DATE	This work is licensed under a Creative Commons Attribution (CC-BY) 4.0 License	OBIS (2019) [Atlantic Reference Centre Museum of Canadian Atlantic Organisms - Invertebrates and Fishes Data] (Available: Ocean Biodiversity Information System. Intergovernmental Oceanographic Commission of UNESCO. https://obis.org. Accessed: 2019-08-29)
d286ae50-ea29-4aa4-8028-2e6e5945a039	Institution REVIZEE - Collection Demersal Fishes	No available dataset citation	Unspecified intellectual rights	OBIS (2019) [Institution REVIZEE - Collection Demersal Fishes] (Available: Ocean Biodiversity Information System. Intergovernmental Oceanographic Commission of UNESCO. https://obis.org. Accessed: 2019-08-29)
d6d6fe4c-425f-4ce7-bf28-7a6befaeb413	National Museum of Natural History Vertebrate Zoology Fishes Collections	National Museum of Natural History, Smithsonian Institution NMNH Fishes Collection Database. National Museum of Natural History, Smithsonian Institution, 10th and Constitution Ave. N.W., Washington, DC 20560-0193, 2007.	This work is licensed under a Creative Commons Attribution (CC-BY) 4.0 License	OBIS (2019) [National Museum of Natural History Vertebrate Zoology Fishes Collections] (Available: Ocean Biodiversity Information System. Intergovernmental Oceanographic Commission of UNESCO. https://obis.org. Accessed: 2019-08-29)

Hygophum hanseni

GBIF

GBIF Occurrence Download https://doi.org/10.15468/dd.va9z8y Accessed from R via rgbif (https://github.com/ropensci/rgbif) on 2019-09-11

OBIS

OBIS (2019) Distribution records of Hygophum hanseni [Dataset] (Available: Ocean Biodiversity Information System. Intergovernmental Oceanographic Commission of UNESCO. www.obis.org. Accessed: 2019-08-29)

Dataset details:

Dataset_ID	Name	Citation	License	OBIS_citation

2870c548-343e-4575-ac67-a4da35182c52	Institution Shirshov Institute - Collection SKAO	No available dataset citation	Unspecified intellectual rights	OBIS (2019) [Institution Shirshov Institute - Collection SKAO] (Available: Ocean Biodiversity Information System. Intergovernmental Oceanographic Commission of UNESCO. https://obis.org. Accessed: 2019-08-29)
8a1ae661-e911-4967-bc06-1168fc5f2d89	iziko South African Museum - Fish Collection	iziko South African Museum - Fish Collection	Restricted	OBIS (2019) [iziko South African Museum - Fish Collection] (Available: Ocean Biodiversity Information System. Intergovernmental Oceanographic Commission of UNESCO. https://obis.org. Accessed: 2019-08-29)
a4f7ee48-0d0b-4c05-a972-27a43b30db58	Institution MCM - Collection DEM	No available dataset citation	Unspecified intellectual rights	OBIS (2019) [Institution MCM - Collection DEM] (Available: Ocean Biodiversity Information System. Intergovernmental Oceanographic Commission of UNESCO. https://obis.org. Accessed: 2019-08-29)
b8617377-eb1c-4db2-baa6-8788a632e810	Ichthyology Collection - Royal Ontario Museum	NA	This work is licensed under a Creative Commons Attribution Non Commercial (CC-BY-NC) 4.0 License	OBIS (2019) [Ichthyology Collection - Royal Ontario Museum] (Available: Ocean Biodiversity Information System. Intergovernmental Oceanographic Commission of UNESCO. https://obis.org. Accessed: 2019-08-29)
d6d6fe4c-425f-4ce7-bf28-7a6befaeb413	National Museum of Natural History Vertebrate Zoology Fishes Collections	National Museum of Natural History, Smithsonian Institution NMNH Fishes Collection Database. National Museum of Natural History, Smithsonian Institution, 10th and Constitution Ave. N.W., Washington, DC 20560-0193, 2007.	This work is licensed under a Creative Commons Attribution (CC-BY) 4.0 License	OBIS (2019) [National Museum of Natural History Vertebrate Zoology Fishes Collections] (Available: Ocean Biodiversity Information System. Intergovernmental Oceanographic Commission of UNESCO. https://obis.org. Accessed: 2019-08-29)

Hygophum reinhardtii

GBIF

GBIF Occurrence Download https://doi.org/10.15468/dd.k6uy74 Accessed from R via rgbif (https://github.com/ropensci/rgbif) on 2019-09-11

OBIS

OBIS (2019) Distribution records of Hygophum reinhardtii [Dataset] (Available: Ocean Biodiversity Information System. Intergovernmental Oceanographic Commission of UNESCO. www.obis.org. Accessed: 2019-08-29)

Dataset details:

Dataset_ID	Name	Citation	License	OBIS_citation
0332e1b5-5525-4301-9659-ef3da3e4e2b6	MARMAP Isaacs-Kidd Midwater Trawl 1990-2009	Marcel Reichert, 2010, MARMAP Isaacs-Kidd Midwater Trawl 1990-2009, SCDNR/NOAA MARMAP Program, SCDNR MARMAP Aggregate data surveys, The Marine Resources Monitoring, Assessment, and Prediction (MARMAP) Program, Marine Resources Research Institute, South Carolina Department of Natural Resources, P. O. Box 12559, Charleston SC 29422-2559, U.S.A.Retrieved from http://www.usgs.gov/obis-usa/	Restricted	OBIS (2019) [MARMAP Isaacs-Kidd Midwater Trawl 1990-2009] (Available: Ocean Biodiversity Information System. Intergovernmental Oceanographic Commission of UNESCO. https://obis.org. Accessed: 2019-08-29)
04e3fd32-b08b-4806-a016-d2dff52ae55a	Asia-Pacific Dataset	Jintsu-Uchifune, Y., Yamamoto, H. (2016) Marine organism occurrence data of the Asia-Pacific region extracted from literature. Available at https://doi.org/10.48518/00002. Accessed on yyyy-mm-dd.	This work is licensed under a Creative Commons Attribution (CC-BY) 4.0 License	OBIS (2019) [Asia-Pacific Dataset] (Available: Ocean Biodiversity Information System. Intergovernmental Oceanographic Commission of UNESCO. https://obis.org. Accessed: 2019-08-29)
1f59030f-f116-4c34-915e-1882d819cda3	Institution Southampton Oceanography Ceter - Collection discovery	No available dataset citation	Unspecified intellectual rights	OBIS (2019) [Institution Southampton Oceanography Ceter - Collection discovery] (Available: Ocean Biodiversity Information System. Intergovernmental Oceanographic Commission of UNESCO. https://obis.org. Accessed: 2019-08-29)
2870c548-343e-4575-ac67-a4da35182c52	Institution Shirshov Institute - Collection SKAO	No available dataset citation	Unspecified intellectual rights	OBIS (2019) [Institution Shirshov Institute - Collection SKAO] (Available: Ocean Biodiversity Information System. Intergovernmental Oceanographic Commission of UNESCO. https://obis.org. Accessed: 2019-08-29)

308a501c-a187-498e-a8bd-9cf3d2b70bd9	Institution REVIZEE - Collection Ictioplankton	No available dataset citation	Unspecified intellectual rights	OBIS (2019) [Institution REVIZEE - Collection Ictioplankton] (Available: Ocean Biodiversity Information System. Intergovernmental Oceanographic Commission of UNESCO. https://obis.org. Accessed: 2019-08-29)
4354345d-7faf-4376-b326-ffbc04b6b0cd	No available dataset name	No available dataset citation	Unspecified intellectual rights	OBIS (2019) [No available dataset name] (Available: Ocean Biodiversity Information System. Intergovernmental Oceanographic Commission of UNESCO. https://obis.org. Accessed: 2019-08-29)
5f2da252-6d49-4c9f-b3b3-1db53d75b345	MARMAP Bongo Nets 1990-2009	Marcel Reichert, 2010, MARMAP Bongo Nets 1990-2009, SCDNR/NOAA MARMAP Program, SCDNR MARMAP Aggregate data surveys, The Marine Resources Monitoring, Assessment, and Prediction (MARMAP) Program, Marine Resources Research Institute, South Carolina Department of Natural Resources, P. O. Box 12559, Charleston SC 29422-2559, U.S.A.Retrieved from http://www.usgs.gov/obis-usa/	Restricted	OBIS (2019) [MARMAP Bongo Nets 1990-2009] (Available: Ocean Biodiversity Information System. Intergovernmental Oceanographic Commission of UNESCO. https://obis.org. Accessed: 2019-08-29)
6a5bc28f-4dfe-4cbf-8a55-7e3a843997ab	SPC NECTALIS Zooplankton/Micronekton specimens, New Caledonia 2014	Allain, V., Menkes, C., 2014. Nectalis 3 cruise, RV Alis. https://doi.org/10.17600/14004900	This work is licensed under a Creative Commons Attribution Non Commercial (CC-BY-NC) 4.0 License	OBIS (2019) [SPC NECTALIS Zooplankton/Micronekton specimens, New Caledonia 2014] (Available: Ocean Biodiversity Information System. Intergovernmental Oceanographic Commission of UNESCO. https://obis.org. Accessed: 2019-08-29)
6c19184e-c305-4273-8890-6d342d86f865	Institution - Collection	No available dataset citation	Unspecified intellectual rights	OBIS (2019) [Institution - Collection] (Available: Ocean Biodiversity Information System. Intergovernmental Oceanographic Commission of UNESCO. https://obis.org. Accessed: 2019-08-29)

705770e5-3474-4e69-be8b-3107a0c5610a	The fishes collection (IC) of the Muséum national d'Histoire naturelle (MNHN - Paris)	Gicim data base, Pruvost P. Causse R., 2009 http://doi.org/10.15468/tm7whu	This work is licensed under a Creative Commons Attribution (CC-BY) 4.0 License	OBIS (2019) [The fishes collection (IC) of the Muséum national d'Histoire naturelle (MNHN - Paris)] (Available: Ocean Biodiversity Information System. Intergovernmental Oceanographic Commission of UNESCO. https://obis.org. Accessed: 2019-08-29)
7e4228e5-a962-4b01-952f-7bf33e213a9c	BioChem: Sameoto zooplankton collection	Sameoto, D.D., Kennedy, M., Spry, J.S, Spry, J.M. (2013). Zooplankton datasets collected using the BIONESS sampler, ring nets and an Icelandic high speed sampler, 1967-2006. OBIS Canada Digital Collections. Published by OBIS http://www.iobis.org/. Accessed on –INSERT DATE	rights: http://data.gc.ca/eng/open-government-licence-canada & http://www.canadensys.net/norms rights holder: Her Majesty the Queen in right of Canada, as represented by the Minister of Fisheries and Oceans	OBIS (2019) [BioChem: Sameoto zooplankton collection] (Available: Ocean Biodiversity Information System. Intergovernmental Oceanographic Commission of UNESCO. https://obis.org. Accessed: 2019-08-29)
8629ec33-be4b-4384-933f-a511fbc29967	MAR-ECO 2004	Wenneck, T. de Lange, Falkenhaug, T. and O.A. Bergstad. 2008. Strategies, methods, and technologies adopted on the RV G.O. Sars MAR-ECO expedition to the mid-Atlantic Ridge in 2004. Deep-sea Research II. 55: 6-28.	This work is licensed under a Creative Commons Attribution (CC-BY) 4.0 License	OBIS (2019) [MAR-ECO 2004] (Available: Ocean Biodiversity Information System. Intergovernmental Oceanographic Commission of UNESCO. https://obis.org. Accessed: 2019-08-29)
87a421bf-4646-49e3-89b7-409b93f2ac7c	Institution - Collection	No available dataset citation	Unspecified intellectual rights	OBIS (2019) [Institution - Collection] (Available: Ocean Biodiversity Information System. Intergovernmental Oceanographic Commission of UNESCO. https://obis.org. Accessed: 2019-08-29)
8a1ae661-e911-4967-bc06-1168fc5f2d89	iziko South African Museum - Fish Collection	iziko South African Museum - Fish Collection	Restricted	OBIS (2019) [iziko South African Museum - Fish Collection] (Available: Ocean Biodiversity Information System. Intergovernmental Oceanographic Commission of UNESCO. https://obis.org. Accessed: 2019-08-29)

b8617377-eb1c-4db2-baa6-8788a632e810	Ichthyology Collection - Royal Ontario Museum	NA	This work is licensed under a Creative Commons Attribution Non Commercial (CC-BY-NC) 4.0 License	OBIS (2019) [Ichthyology Collection - Royal Ontario Museum] (Available: Ocean Biodiversity Information System. Intergovernmental Oceanographic Commission of UNESCO. https://obis.org. Accessed: 2019-08-29)
c24bf1c2-2c62-4056-a841-56d94e6e876a	Fish specimens	ROM Fish Collection (accessed through GBIF data portal, http://data.gbif.org/datasets/resource/660, 2012-01-20) http://doi.org/10.15468/syisbx	Unrestricted	OBIS (2019) [Fish specimens] (Available: Ocean Biodiversity Information System. Intergovernmental Oceanographic Commission of UNESCO. https://obis.org. Accessed: 2019-08-29)
cc8f28ce-e48d-4945-abfe-9d150a22dcd6	Hamburg pelagic fish database	Post, A. 1987. Pelagic transects of FRVs "Walther Herwig" and "Anton Dohrn" in the Atlantic Ocean 1966 to 1986. Mitt. Inst. f. Seefischerei d. BfaFi Hamburg, 42: 1-68.	This work is licensed under a Creative Commons Attribution (CC-BY) 4.0 License	OBIS (2019) [Hamburg pelagic fish database] (Available: Ocean Biodiversity Information System. Intergovernmental Oceanographic Commission of UNESCO. https://obis.org. Accessed: 2019-08-29)
ce1d93f3-8b0f-4ee7-9a4d-0393a6ec7fea	Atlantic Reference Centre Museum of Canadian Atlantic Organisms - Invertebrates and Fishes Data	Van Guelpen, L., 2016. Atlantic Reference Centre Museum of Canadian Atlantic Organisms - Invertebrates and Fishes Data. Version 4 In OBIS Canada Digital Collections. Bedford Institute of Oceanography, Dartmouth, NS, Canada. Published by OBIS, Digital http://www.iobis.org/. Accessed on –INSERT DATE	This work is licensed under a Creative Commons Attribution (CC-BY) 4.0 License	OBIS (2019) [Atlantic Reference Centre Museum of Canadian Atlantic Organisms - Invertebrates and Fishes Data] (Available: Ocean Biodiversity Information System. Intergovernmental Oceanographic Commission of UNESCO. https://obis.org. Accessed: 2019-08-29)
cfc56587-48c3-4e3d-9350-3a4d9a28b681	Institution - Collection	No available dataset citation	Unspecified intellectual rights	OBIS (2019) [Institution - Collection] (Available: Ocean Biodiversity Information System. Intergovernmental Oceanographic Commission of UNESCO. https://obis.org. Accessed: 2019-08-29)
d6d6fe4c-425f-4ce7-bf28-7a6befaeb413	National Museum of Natural History Vertebrate Zoology Fishes Collections	National Museum of Natural History, Smithsonian Institution NMNH Fishes Collection Database. National Museum of Natural History, Smithsonian Institution, 10th and Constitution Ave. N.W., Washington, DC 20560-0193, 2007.	This work is licensed under a Creative Commons Attribution (CC-BY) 4.0 License	OBIS (2019) [National Museum of Natural History Vertebrate Zoology Fishes Collections] (Available: Ocean Biodiversity Information System. Intergovernmental Oceanographic Commission of UNESCO. https://obis.org. Accessed: 2019-08-29)

ff8b7809-41bc-40ad-8160-0e33862817a0	Biodiversity Research Museum, Academia Sinica, Taiwan	TELDAP, Biodiversity Research Museum, Academia Sinica, Taiwan (accessed through GBIF data portal, http://data.gbif.org/datasets/resource/9093, yyyy-mm-dd)	Unrestricted	OBIS (2019) [Biodiversity Research Museum, Academia Sinica, Taiwan] (Available: Ocean Biodiversity Information System. Intergovernmental Oceanographic Commission of UNESCO. https://obis.org. Accessed: 2019-08-29)

Lobianchia dofleini

GBIF

GBIF Occurrence Download https://doi.org/10.15468/dd.kyfksg Accessed from R via rgbif (https://github.com/ropensci/rgbif) on 2019-09-11

OBIS

OBIS (2019) Distribution records of Lobianchia dofleini [Dataset] (Available: Ocean Biodiversity Information System. Intergovernmental Oceanographic Commission of UNESCO. www.obis.org. Accessed: 2019-08-29)

Dataset details:

Dataset_ID	Name	Citation	License	OBIS_citation
09be818d-531c-4d86-8eba-5db81d42fdba	COLETA - IMAR/DOP-Uac reference collection from 1977 to 2012	Institute of Marine Research (IMAR - Azores), Portugal; Department of Oceanography and Fisheries (DOP) - UAC, Portugal (2015): COLETA - IMAR/DOP-Uac reference collection from 1977 to 2012. https://dx.doi.org/10.14284/23	This work is licensed under a Creative Commons Attribution (CC-BY) 4.0 License	OBIS (2019) [COLETA - IMAR/DOP-Uac reference collection from 1977 to 2012] (Available: Ocean Biodiversity Information System. Intergovernmental Oceanographic Commission of UNESCO. https://obis.org. Accessed: 2019-08-29)
0c9db499-759b-46d8-8989-799f9ff9f235	Auckland Museum NZ Marine Collection	Blom W, Moriarty A (2018). Auckland Museum NZ Marine Collection. Version 1.11. Auckland War Memorial Museum. Occurrence Dataset https://doi.org/10.15468/plyefd accessed via GBIF.org on 2018-01-15.	This work is licensed under a Creative Commons Attribution (CC-BY) 4.0 License	OBIS (2019) [Auckland Museum NZ Marine Collection] (Available: Ocean Biodiversity Information System. Intergovernmental Oceanographic Commission of UNESCO. https://obis.org. Accessed: 2019-08-29)

1f59030f-f116-4c34-915e-1882d819cda3	Institution Southampton Oceanography Ceter - Collection discovery	No available dataset citation	Unspecified intellectual rights	OBIS (2019) [Institution Southampton Oceanography Ceter - Collection discovery] (Available: Ocean Biodiversity Information System. Intergovernmental Oceanographic Commission of UNESCO. https://obis.org. Accessed: 2019-08-29)
270f3e70-ff9b-411d-b170-2bc914d83f26	Biological Reference Collections ICM CSIC	Olivas González F J (2016): Biological Reference Collections ICM CSIC. Institute of Marine Sciences (ICM-CSIC). https://dx.doi.org/10.15470/qlqqdx	This work is licensed under a Creative Commons Attribution Non Commercial (CC-BY-NC) 4.0 License	OBIS (2019) [Biological Reference Collections ICM CSIC] (Available: Ocean Biodiversity Information System. Intergovernmental Oceanographic Commission of UNESCO. https://obis.org. Accessed: 2019-08-29)
276b09b1-0e57-4128-a8b1-c15998da3081	Megafaunal data from the 2009 BIOFUN trans-Mediterranean deep-sea cruise	Tecchio, S.; Ramirez-Llodra, E. (2018): Megafaunal data from the 2009 BIOFUN trans-Mediterranean deep-sea cruise https://dx.doi.org/10.14284/311	To the extent possible under law, the publisher has waived all rights to these data and has dedicated them to the Public Domain (CC0 1.0)	OBIS (2019) [Megafaunal data from the 2009 BIOFUN trans-Mediterranean deep-sea cruise] (Available: Ocean Biodiversity Information System. Intergovernmental Oceanographic Commission of UNESCO. https://obis.org. Accessed: 2019-08-29)
2870c548-343e-4575-ac67-a4da35182c52	Institution Shirshov Institute - Collection SKAO	No available dataset citation	Unspecified intellectual rights	OBIS (2019) [Institution Shirshov Institute - Collection SKAO] (Available: Ocean Biodiversity Information System. Intergovernmental Oceanographic Commission of UNESCO. https://obis.org. Accessed: 2019-08-29)
3d922162-062c-4ad2-bf4a-f2493bd3a95d	Institution Bedford Institute of Oceanography (BIO) - Collection SUMMER	No available dataset citation	Unspecified intellectual rights	OBIS (2019) [Institution Bedford Institute of Oceanography (BIO) - Collection SUMMER] (Available: Ocean Biodiversity Information System. Intergovernmental Oceanographic Commission of UNESCO. https://obis.org. Accessed: 2019-08-29)
4e25e0ce-b17d-4192-9b55-417f1e0c4fc8	Institution KU - Collection KUI	No available dataset citation	Unspecified intellectual rights	OBIS (2019) [Institution KU - Collection KUI] (Available: Ocean Biodiversity Information System. Intergovernmental Oceanographic Commission of UNESCO. https://obis.org. Accessed: 2019-08-29)

5bf31864-f4f6-4986-a2c5-1a0e5e9d9e26	Otolith reference collection, Instituto de Ciencias del Mar-CSIC	Institute of Marine Sciences (ICM-CSIC): Colección de referencia de otolitos, Instituto de Ciencias del Mar-CSIC. Accessed via http://www.gbif.org/dataset/e95d0010-b3f1-11de-82f8-b8a03c50a862 on yyyy-mm-dd https://doi.org/10.15468/wdwxid	This work is licensed under a Creative Commons Attribution (CC-BY) 4.0 License	OBIS (2019) [Otolith reference collection, Instituto de Ciencias del Mar-CSIC] (Available: Ocean Biodiversity Information System. Intergovernmental Oceanographic Commission of UNESCO. https://obis.org. Accessed: 2019-08-29)
623f4f98-4e66-4bee-9e1c-17cb74cd2d21	Institution IEO - Collection MEDITS-Spain	No available dataset citation	Unspecified intellectual rights	OBIS (2019) [Institution IEO - Collection MEDITS-Spain] (Available: Ocean Biodiversity Information System. Intergovernmental Oceanographic Commission of UNESCO. https://obis.org. Accessed: 2019-08-29)
705770e5-3474-4e69-be8b-3107a0c5610a	The fishes collection (IC) of the Muséum national d'Histoire naturelle (MNHN - Paris)	Gicim data base, Pruvost P. Causse R., 2009 http://doi.org/10.15468/tm7whu	This work is licensed under a Creative Commons Attribution (CC-BY) 4.0 License	OBIS (2019) [The fishes collection (IC) of the Muséum national d'Histoire naturelle (MNHN - Paris)] (Available: Ocean Biodiversity Information System. Intergovernmental Oceanographic Commission of UNESCO. https://obis.org. Accessed: 2019-08-29)
8629ec33-be4b-4384-933f-a511fbc29967	MAR-ECO 2004	Wenneck, T. de Lange, Falkenhaug, T. and O.A. Bergstad. 2008. Strategies, methods, and technologies adopted on the RV G.O. Sars MAR-ECO expedition to the mid-Atlantic Ridge in 2004. Deep-sea Research II. 55: 6-28.	This work is licensed under a Creative Commons Attribution (CC-BY) 4.0 License	OBIS (2019) [MAR-ECO 2004] (Available: Ocean Biodiversity Information System. Intergovernmental Oceanographic Commission of UNESCO. https://obis.org. Accessed: 2019-08-29)
8a1ae661-e911-4967-bc06-1168fc5f2d89	iziko South African Museum - Fish Collection	iziko South African Museum - Fish Collection	Restricted	OBIS (2019) [iziko South African Museum - Fish Collection] (Available: Ocean Biodiversity Information System. Intergovernmental Oceanographic Commission of UNESCO. https://obis.org. Accessed: 2019-08-29)
b8617377-eb1c-4db2-baa6-8788a632e810	Ichthyology Collection - Royal Ontario Museum	NA	This work is licensed under a Creative Commons Attribution Non Commercial (CC-BY-NC) 4.0 License	OBIS (2019) [Ichthyology Collection - Royal Ontario Museum] (Available: Ocean Biodiversity Information System. Intergovernmental Oceanographic Commission of UNESCO. https://obis.org. Accessed: 2019-08-29)

c24bf1c2-2c62-4056-a841-56d94e6e876a	Fish specimens	ROM Fish Collection (accessed through GBIF data portal, http://data.gbif.org/datasets/resource/660, 2012-01-20) http://doi.org/10.15468/syisbx	Unrestricted	OBIS (2019) [Fish specimens] (Available: Ocean Biodiversity Information System. Intergovernmental Oceanographic Commission of UNESCO. https://obis.org. Accessed: 2019-08-29)
cc8f28ce-e48d-4945-abfe-9d150a22dcd6	Hamburg pelagic fish database	Post, A. 1987. Pelagic transects of FRVs "Walther Herwig" and "Anton Dohrn" in the Atlantic Ocean 1966 to 1986. Mitt. Inst. f. Seefischerei d. BfaFi Hamburg, 42: 1-68.	This work is licensed under a Creative Commons Attribution (CC-BY) 4.0 License	OBIS (2019) [Hamburg pelagic fish database] (Available: Ocean Biodiversity Information System. Intergovernmental Oceanographic Commission of UNESCO. https://obis.org. Accessed: 2019-08-29)
ce1d93f3-8b0f-4ee7-9a4d-0393a6ec7fea	Atlantic Reference Centre Museum of Canadian Atlantic Organisms - Invertebrates and Fishes Data	Van Guelpen, L., 2016. Atlantic Reference Centre Museum of Canadian Atlantic Organisms - Invertebrates and Fishes Data. Version 4 In OBIS Canada Digital Collections. Bedford Institute of Oceanography, Dartmouth, NS, Canada. Published by OBIS, Digital http://www.iobis.org/. Accessed on –INSERT DATE	This work is licensed under a Creative Commons Attribution (CC-BY) 4.0 License	OBIS (2019) [Atlantic Reference Centre Museum of Canadian Atlantic Organisms - Invertebrates and Fishes Data] (Available: Ocean Biodiversity Information System. Intergovernmental Oceanographic Commission of UNESCO. https://obis.org. Accessed: 2019-08-29)
cfc56587-48c3-4e3d-9350-3a4d9a28b681	Institution NOAA, NMFS, Northeast Fisheries Science Center - Collection DEEPWATER SYSTEMATICS	No available dataset citation	Unspecified intellectual rights	OBIS (2019) [Institution NOAA, NMFS, Northeast Fisheries Science Center - Collection DEEPWATER SYSTEMATICS] (Available: Ocean Biodiversity Information System. Intergovernmental Oceanographic Commission of UNESCO. https://obis.org. Accessed: 2019-08-29)
d6d6fe4c-425f-4ce7-bf28-7a6befaeb413	National Museum of Natural History Vertebrate Zoology Fishes Collections	National Museum of Natural History, Smithsonian Institution NMNH Fishes Collection Database. National Museum of Natural History, Smithsonian Institution, 10th and Constitution Ave. N.W., Washington, DC 20560-0193, 2007.	This work is licensed under a Creative Commons Attribution (CC-BY) 4.0 License	OBIS (2019) [National Museum of Natural History Vertebrate Zoology Fishes Collections] (Available: Ocean Biodiversity Information System. Intergovernmental Oceanographic Commission of UNESCO. https://obis.org. Accessed: 2019-08-29)

e09c824c-cbd8-4529-b382-5306b2b3a875	Marine biodiversity atlas of the Balearic Sea	Deudero, Vallespir, Obrador 2011. Atlas de Biodiversidad Marina del Mar Balear. http://www.ba.ieo.es	This work is licensed under a Creative Commons Attribution Non Commercial (CC-BY-NC) 4.0 License	OBIS (2019) [Marine biodiversity atlas of the Balearic Sea] (Available: Ocean Biodiversity Information System. Intergovernmental Oceanographic Commission of UNESCO. https://obis.org. Accessed: 2019-08-29)

Myctophum phengodes

GBIF

GBIF Occurrence Download https://doi.org/10.15468/dd.c9kv58 Accessed from R via rgbif (https://github.com/ropensci/rgbif) on 2019-09-11

OBIS

OBIS (2019) Distribution records of Myctophum phengodes [Dataset] (Available: Ocean Biodiversity Information System. Intergovernmental Oceanographic Commission of UNESCO. www.obis.org. Accessed: 2019-08-29)

Dataset details:

Dataset_ID	Name	Citation	License	OBIS_citation
0c9db499-759b-46d8-8989-799f9ff9f235	Auckland Museum NZ Marine Collection	Blom W, Moriarty A (2018). Auckland Museum NZ Marine Collection. Version 1.11. Auckland War Memorial Museum. Occurrence Dataset https://doi.org/10.15468/plyefd accessed via GBIF.org on 2018-01-15.	This work is licensed under a Creative Commons Attribution (CC-BY) 4.0 License	OBIS (2019) [Auckland Museum NZ Marine Collection] (Available: Ocean Biodiversity Information System. Intergovernmental Oceanographic Commission of UNESCO. https://obis.org. Accessed: 2019-08-29)
270f3e70-ff9b-411d-b170-2bc914d83f26	Biological Reference Collections ICM CSIC	Olivas González F J (2016): Biological Reference Collections ICM CSIC. Institute of Marine Sciences (ICM-CSIC). https://dx.doi.org/10.15470/qlqqdx	This work is licensed under a Creative Commons Attribution Non Commercial (CC-BY-NC) 4.0 License	OBIS (2019) [Biological Reference Collections ICM CSIC] (Available: Ocean Biodiversity Information System. Intergovernmental Oceanographic Commission of UNESCO. https://obis.org. Accessed: 2019-08-29)

2870c548-343e-4575-ac67-a4da35182c52	Institution Shirshov Institute - Collection SKAO	No available dataset citation	Unspecified intellectual rights	OBIS (2019) [Institution Shirshov Institute - Collection SKAO] (Available: Ocean Biodiversity Information System. Intergovernmental Oceanographic Commission of UNESCO. https://obis.org. Accessed: 2019-08-29)
4bdc1f6f-e16a-48b4-b995-b51bd41caa8d	Dataset of the multidisciplinary research surveys in the seamounts of Ewing and Valdivia Bank (Walvis Ridge) - SE Atlantic	López-Abellán, L. J.; Sarralde Vizuete, R.; González Jiménez, J. F.; Centro Oceanográfico de Canarias – IEO, Spain (2015). Dataset of the multidisciplinary research surveys in the seamounts of Ewing and Valdivia Bank (Walvis Ridge) - SE Atlantic https://dx.doi.org/10.14284/58	Attribution-NoDerivatives (CC BY-ND)	OBIS (2019) [Dataset of the multidisciplinary research surveys in the seamounts of Ewing and Valdivia Bank (Walvis Ridge) - SE Atlantic] (Available: Ocean Biodiversity Information System. Intergovernmental Oceanographic Commission of UNESCO. https://obis.org. Accessed: 2019-08-29)
6c19184e-c305-4273-8890-6d342d86f865	Institution REVIZEE - Collection Pelagic Fishes	No available dataset citation	Unspecified intellectual rights	OBIS (2019) [Institution REVIZEE - Collection Pelagic Fishes] (Available: Ocean Biodiversity Information System. Intergovernmental Oceanographic Commission of UNESCO. https://obis.org. Accessed: 2019-08-29)
705770e5-3474-4e69-be8b-3107a0c5610a	The fishes collection (IC) of the Muséum national d'Histoire naturelle (MNHN - Paris)	Gicim data base, Pruvost P. Causse R., 2009 http://doi.org/10.15468/tm7whu	This work is licensed under a Creative Commons Attribution (CC-BY) 4.0 License	OBIS (2019) [The fishes collection (IC) of the Muséum national d'Histoire naturelle (MNHN - Paris)] (Available: Ocean Biodiversity Information System. Intergovernmental Oceanographic Commission of UNESCO. https://obis.org. Accessed: 2019-08-29)
8a1ae661-e911-4967-bc06-1168fc5f2d89	iziko South African Museum - Fish Collection	iziko South African Museum - Fish Collection	Restricted	OBIS (2019) [iziko South African Museum - Fish Collection] (Available: Ocean Biodiversity Information System. Intergovernmental Oceanographic Commission of UNESCO. https://obis.org. Accessed: 2019-08-29)
9ff216fc-777e-4f9b-9860-95ed7366870d	Institution SAIAB - Collection SAIAB	No available dataset citation	Unspecified intellectual rights	OBIS (2019) [Institution SAIAB - Collection SAIAB] (Available: Ocean Biodiversity Information System. Intergovernmental Oceanographic Commission of UNESCO. https://obis.org. Accessed: 2019-08-29)

a4f7ee48-0d0b-4c05-a972-27a43b30db58	Institution MCM - Collection DEM	No available dataset citation	Unspecified intellectual rights	OBIS (2019) [Institution MCM - Collection DEM] (Available: Ocean Biodiversity Information System. Intergovernmental Oceanographic Commission of UNESCO. https://obis.org. Accessed: 2019-08-29)
b8617377-eb1c-4db2-baa6-8788a632e810	Ichthyology Collection - Royal Ontario Museum	NA	This work is licensed under a Creative Commons Attribution Non Commercial (CC-BY-NC) 4.0 License	OBIS (2019) [Ichthyology Collection - Royal Ontario Museum] (Available: Ocean Biodiversity Information System. Intergovernmental Oceanographic Commission of UNESCO. https://obis.org. Accessed: 2019-08-29)
d6d6fe4c-425f-4ce7-bf28-7a6befaeb413	National Museum of Natural History Vertebrate Zoology Fishes Collections	National Museum of Natural History, Smithsonian Institution NMNH Fishes Collection Database. National Museum of Natural History, Smithsonian Institution, 10th and Constitution Ave. N.W., Washington, DC 20560-0193, 2007.	This work is licensed under a Creative Commons Attribution (CC-BY) 4.0 License	OBIS (2019) [National Museum of Natural History Vertebrate Zoology Fishes Collections] (Available: Ocean Biodiversity Information System. Intergovernmental Oceanographic Commission of UNESCO. https://obis.org. Accessed: 2019-08-29)

Scopelopsis multipunctatus

GBIF

GBIF Occurrence Download https://doi.org/10.15468/dd.tdh5u8 Accessed from R via rgbif (https://github.com/ropensci/rgbif) on 2019-09-11

OBIS

OBIS (2019) Distribution records of Scopelopsis multipunctatus [Dataset] (Available: Ocean Biodiversity Information System. Intergovernmental Oceanographic Commission of UNESCO. www.obis.org. Accessed: 2019-08-29)

Dataset details:

Dataset_ID	Name	Citation	License	OBIS_citation

2870c548-343e-4575-ac67-a4da35182c52	Institution Shirshov Institute - Collection SKAO	No available dataset citation	Unspecified intellectual rights	OBIS (2019) [Institution Shirshov Institute - Collection SKAO] (Available: Ocean Biodiversity Information System. Intergovernmental Oceanographic Commission of UNESCO. https://obis.org. Accessed: 2019-08-29)
6a5bc28f-4dfe-4cbf-8a55-7e3a843997ab	SPC NECTALIS Zooplankton/Micronekton specimens, New Caledonia 2014	Allain, V., Menkes, C., 2014. Nectalis 3 cruise, RV Alis. https://doi.org/10.17600/14004900	This work is licensed under a Creative Commons Attribution Non Commercial (CC-BY-NC) 4.0 License	OBIS (2019) [SPC NECTALIS Zooplankton/Micronekton specimens, New Caledonia 2014] (Available: Ocean Biodiversity Information System. Intergovernmental Oceanographic Commission of UNESCO. https://obis.org. Accessed: 2019-08-29)
6c19184e-c305-4273-8890-6d342d86f865	Institution REVIZEE - Collection Pelagic Fishes	No available dataset citation	Unspecified intellectual rights	OBIS (2019) [Institution REVIZEE - Collection Pelagic Fishes] (Available: Ocean Biodiversity Information System. Intergovernmental Oceanographic Commission of UNESCO. https://obis.org. Accessed: 2019-08-29)
8a1ae661-e911-4967-bc06-1168fc5f2d89	iziko South African Museum - Fish Collection	iziko South African Museum - Fish Collection	Restricted	OBIS (2019) [iziko South African Museum - Fish Collection] (Available: Ocean Biodiversity Information System. Intergovernmental Oceanographic Commission of UNESCO. https://obis.org. Accessed: 2019-08-29)
9ff216fc-777e-4f9b-9860-95ed7366870d	Institution SAIAB - Collection SAIAB	No available dataset citation	Unspecified intellectual rights	OBIS (2019) [Institution SAIAB - Collection SAIAB] (Available: Ocean Biodiversity Information System. Intergovernmental Oceanographic Commission of UNESCO. https://obis.org. Accessed: 2019-08-29)
a4f7ee48-0d0b-4c05-a972-27a43b30db58	Institution MCM - Collection DEM	No available dataset citation	Unspecified intellectual rights	OBIS (2019) [Institution MCM - Collection DEM] (Available: Ocean Biodiversity Information System. Intergovernmental Oceanographic Commission of UNESCO. https://obis.org. Accessed: 2019-08-29)

b8617377-eb1c-4db2-baa6-8788a632e810	Ichthyology Collection - Royal Ontario Museum	NA	This work is licensed under a Creative Commons Attribution Non Commercial (CC-BY-NC) 4.0 License	OBIS (2019) [Ichthyology Collection - Royal Ontario Museum] (Available: Ocean Biodiversity Information System. Intergovernmental Oceanographic Commission of UNESCO. https://obis.org. Accessed: 2019-08-29)
d6d6fe4c-425f-4ce7-bf28-7a6befaeb413	National Museum of Natural History Vertebrate Zoology Fishes Collections	National Museum of Natural History, Smithsonian Institution NMNH Fishes Collection Database. National Museum of Natural History, Smithsonian Institution, 10th and Constitution Ave. N.W., Washington, DC 20560-0193, 2007.	This work is licensed under a Creative Commons Attribution (CC-BY) 4.0 License	OBIS (2019) [National Museum of Natural History Vertebrate Zoology Fishes Collections] (Available: Ocean Biodiversity Information System. Intergovernmental Oceanographic Commission of UNESCO. https://obis.org. Accessed: 2019-08-29)

Symbolophorus barnardi

GBIF

GBIF Occurrence Download https://doi.org/10.15468/dd.m5zdw6 Accessed from R via rgbif (https://github.com/ropensci/rgbif) on 2019-09-11

OBIS

OBIS (2019) Distribution records of Symbolophorus barnardi [Dataset] (Available: Ocean Biodiversity Information System. Intergovernmental Oceanographic Commission of UNESCO. www.obis.org. Accessed: 2019-08-29)

Dataset details:

Dataset_ID	Name	Citation	License	OBIS_citation
0c9db499-759b-46d8-8989-799f9ff9f235	Auckland Museum NZ Marine Collection	Blom W, Moriarty A (2018). Auckland Museum NZ Marine Collection. Version 1.11. Auckland War Memorial Museum. Occurrence Dataset https://doi.org/10.15468/plyefd accessed via GBIF.org on 2018-01-15.	This work is licensed under a Creative Commons Attribution (CC-BY) 4.0 License	OBIS (2019) [Auckland Museum NZ Marine Collection] (Available: Ocean Biodiversity Information System. Intergovernmental Oceanographic Commission of UNESCO. https://obis.org. Accessed: 2019-08-29)

270f3e70-ff9b-411d-b170-2bc914d83f26	Biological Reference Collections ICM CSIC	Olivas González F J (2016): Biological Reference Collections ICM CSIC. Institute of Marine Sciences (ICM-CSIC). https://dx.doi.org/10.15470/qlqqdx	This work is licensed under a Creative Commons Attribution Non Commercial (CC-BY-NC) 4.0 License	OBIS (2019) [Biological Reference Collections ICM CSIC] (Available: Ocean Biodiversity Information System. Intergovernmental Oceanographic Commission of UNESCO. https://obis.org. Accessed: 2019-08-29)
2870c548-343e-4575-ac67-a4da35182c52	Institution Shirshov Institute - Collection SKAO	No available dataset citation	Unspecified intellectual rights	OBIS (2019) [Institution Shirshov Institute - Collection SKAO] (Available: Ocean Biodiversity Information System. Intergovernmental Oceanographic Commission of UNESCO. https://obis.org. Accessed: 2019-08-29)
705770e5-3474-4e69-be8b-3107a0c5610a	The fishes collection (IC) of the Muséum national d'Histoire naturelle (MNHN - Paris)	Gicim data base, Pruvost P. Causse R., 2009 http://doi.org/10.15468/tm7whu	This work is licensed under a Creative Commons Attribution (CC-BY) 4.0 License	OBIS (2019) [The fishes collection (IC) of the Muséum national d'Histoire naturelle (MNHN - Paris)] (Available: Ocean Biodiversity Information System. Intergovernmental Oceanographic Commission of UNESCO. https://obis.org. Accessed: 2019-08-29)
8a1ae661-e911-4967-bc06-1168fc5f2d89	iziko South African Museum - Fish Collection	iziko South African Museum - Fish Collection	Restricted	OBIS (2019) [iziko South African Museum - Fish Collection] (Available: Ocean Biodiversity Information System. Intergovernmental Oceanographic Commission of UNESCO. https://obis.org. Accessed: 2019-08-29)
9ff216fc-777e-4f9b-9860-95ed7366870d	Institution SAIAB - Collection SAIAB	No available dataset citation	Unspecified intellectual rights	OBIS (2019) [Institution SAIAB - Collection SAIAB] (Available: Ocean Biodiversity Information System. Intergovernmental Oceanographic Commission of UNESCO. https://obis.org. Accessed: 2019-08-29)
a4f7ee48-0d0b-4c05-a972-27a43b30db58	Institution MCM - Collection DEM	No available dataset citation	Unspecified intellectual rights	OBIS (2019) [Institution MCM - Collection DEM] (Available: Ocean Biodiversity Information System. Intergovernmental Oceanographic Commission of UNESCO. https://obis.org. Accessed: 2019-08-29)

d286ae50-ea29-4aa4-8028-2e6e5945a039	Institution REVIZEE - Collection Demersal Fishes	No available dataset citation	Unspecified intellectual rights	OBIS (2019) [Institution REVIZEE - Collection Demersal Fishes] (Available: Ocean Biodiversity Information System. Intergovernmental Oceanographic Commission of UNESCO. https://obis.org. Accessed: 2019-08-29)
d6d6fe4c-425f-4ce7-bf28-7a6befaeb413	National Museum of Natural History Vertebrate Zoology Fishes Collections	National Museum of Natural History, Smithsonian Institution NMNH Fishes Collection Database. National Museum of Natural History, Smithsonian Institution, 10th and Constitution Ave. N.W., Washington, DC 20560-0193, 2007.	This work is licensed under a Creative Commons Attribution (CC-BY) 4.0 License	OBIS (2019) [National Museum of Natural History Vertebrate Zoology Fishes Collections] (Available: Ocean Biodiversity Information System. Intergovernmental Oceanographic Commission of UNESCO. https://obis.org. Accessed: 2019-08-29)

Lampanyctus pusillus

GBIF

GBIF Occurrence Download https://doi.org/10.15468/dd.wf73wg Accessed from R via rgbif (https://github.com/ropensci/rgbif) on 2019-09-11

OBIS

OBIS (2019) Distribution records of Lampanyctus pusillus [Dataset] (Available: Ocean Biodiversity Information System. Intergovernmental Oceanographic Commission of UNESCO. www.obis.org. Accessed: 2019-08-29)

Dataset details:

Dataset_ID	Name	Citation	License	OBIS_citation
09be818d-531c-4d86-8eba-5db81d42fdba	COLETA - IMAR/DOP-Uac reference collection from 1977 to 2012	Institute of Marine Research (IMAR - Azores), Portugal; Department of Oceanography and Fisheries (DOP) - UAC, Portugal (2015): COLETA - IMAR/DOP-Uac reference collection from 1977 to 2012. https://dx.doi.org/10.14284/23	This work is licensed under a Creative Commons Attribution (CC-BY) 4.0 License	OBIS (2019) [COLETA - IMAR/DOP-Uac reference collection from 1977 to 2012] (Available: Ocean Biodiversity Information System. Intergovernmental Oceanographic Commission of UNESCO. https://obis.org. Accessed: 2019-08-29)

1f59030f-f116-4c34-915e-1882d819cda3	Institution Southampton Oceanography Ceter - Collection discovery	No available dataset citation	Unspecified intellectual rights	OBIS (2019) [Institution Southampton Oceanography Ceter - Collection discovery] (Available: Ocean Biodiversity Information System. Intergovernmental Oceanographic Commission of UNESCO. https://obis.org. Accessed: 2019-08-29)
2870c548-343e-4575-ac67-a4da35182c52	Institution Shirshov Institute - Collection SKAO	No available dataset citation	Unspecified intellectual rights	OBIS (2019) [Institution Shirshov Institute - Collection SKAO] (Available: Ocean Biodiversity Information System. Intergovernmental Oceanographic Commission of UNESCO. https://obis.org. Accessed: 2019-08-29)
3d922162-062c-4ad2-bf4a-f2493bd3a95d	Institution Bedford Institute of Oceanography (BIO) - Collection SUMMER	No available dataset citation	Unspecified intellectual rights	OBIS (2019) [Institution Bedford Institute of Oceanography (BIO) - Collection SUMMER] (Available: Ocean Biodiversity Information System. Intergovernmental Oceanographic Commission of UNESCO. https://obis.org. Accessed: 2019-08-29)
4e25e0ce-b17d-4192-9b55-417f1e0c4fc8	Institution KU - Collection KUI	No available dataset citation	Unspecified intellectual rights	OBIS (2019) [Institution KU - Collection KUI] (Available: Ocean Biodiversity Information System. Intergovernmental Oceanographic Commission of UNESCO. https://obis.org. Accessed: 2019-08-29)
705770e5-3474-4e69-be8b-3107a0c5610a	The fishes collection (IC) of the Muséum national d'Histoire naturelle (MNHN - Paris)	Gicim data base, Pruvost P. Causse R., 2009 http://doi.org/10.15468/tm7whu	This work is licensed under a Creative Commons Attribution (CC-BY) 4.0 License	OBIS (2019) [The fishes collection (IC) of the Muséum national d'Histoire naturelle (MNHN - Paris)] (Available: Ocean Biodiversity Information System. Intergovernmental Oceanographic Commission of UNESCO. https://obis.org. Accessed: 2019-08-29)
8629ec33-be4b-4384-933f-a511fbc29967	MAR-ECO 2004	Wenneck, T. de Lange, Falkenhaug, T. and O.A. Bergstad. 2008. Strategies, methods, and technologies adopted on the RV G.O. Sars MAR-ECO expedition to the mid-Atlantic Ridge in 2004. Deep-sea Research II. 55: 6-28.	This work is licensed under a Creative Commons Attribution (CC-BY) 4.0 License	OBIS (2019) [MAR-ECO 2004] (Available: Ocean Biodiversity Information System. Intergovernmental Oceanographic Commission of UNESCO. https://obis.org. Accessed: 2019-08-29)

8843341c-ddd1-47a0-b4e6-5f37eec9b317	Institution AADC - Collection Historic_Fish	No available dataset citation	Unspecified intellectual rights	OBIS (2019) [Institution AADC - Collection Historic_Fish] (Available: Ocean Biodiversity Information System. Intergovernmental Oceanographic Commission of UNESCO. https://obis.org. Accessed: 2019-08-29)
8a1ae661-e911-4967-bc06-1168fc5f2d89	iziko South African Museum - Fish Collection	iziko South African Museum - Fish Collection	Restricted	OBIS (2019) [iziko South African Museum - Fish Collection] (Available: Ocean Biodiversity Information System. Intergovernmental Oceanographic Commission of UNESCO. https://obis.org. Accessed: 2019-08-29)
a4f7ee48-0d0b-4c05-a972-27a43b30db58	Institution MCM - Collection DEM	No available dataset citation	Unspecified intellectual rights	OBIS (2019) [Institution MCM - Collection DEM] (Available: Ocean Biodiversity Information System. Intergovernmental Oceanographic Commission of UNESCO. https://obis.org. Accessed: 2019-08-29)
b0a7add2-dd9e-4020-9ca4-5df048c8f6a2	Bigood	Fabri, M-C. et al., Ifremer BIOCEAN database (Deep Sea Benthic Fauna). Institut Français de Recherche pour l'Exploitation de la Mer, Ifremer, Issy-les-Moulineaux, France. World Wide Web electronic publication, http://www.ifremer.fr/isi/biocean	This work is licensed under a Creative Commons Attribution (CC-BY) 4.0 License	OBIS (2019) [Bigood] (Available: Ocean Biodiversity Information System. Intergovernmental Oceanographic Commission of UNESCO. https://obis.org. Accessed: 2019-08-29)
b8617377-eb1c-4db2-baa6-8788a632e810	Ichthyology Collection - Royal Ontario Museum	NA	This work is licensed under a Creative Commons Attribution Non Commercial (CC-BY-NC) 4.0 License	OBIS (2019) [Ichthyology Collection - Royal Ontario Museum] (Available: Ocean Biodiversity Information System. Intergovernmental Oceanographic Commission of UNESCO. https://obis.org. Accessed: 2019-08-29)
c24bf1c2-2c62-4056-a841-56d94e6e876a	Fish specimens	ROM Fish Collection (accessed through GBIF data portal, http://data.gbif.org/datasets/resource/660, 2012-01-20) http://doi.org/10.15468/syisbx	Unrestricted	OBIS (2019) [Fish specimens] (Available: Ocean Biodiversity Information System. Intergovernmental Oceanographic Commission of UNESCO. https://obis.org. Accessed: 2019-08-29)

cc8f28ce-e48d-4945-abfe-9d150a22dcd6	Hamburg pelagic fish database	Post, A. 1987. Pelagic transects of FRVs "Walther Herwig" and "Anton Dohrn" in the Atlantic Ocean 1966 to 1986. Mitt. Inst. f. Seefischerei d. BfaFi Hamburg, 42: 1-68.	This work is licensed under a Creative Commons Attribution (CC-BY) 4.0 License	OBIS (2019) [Hamburg pelagic fish database] (Available: Ocean Biodiversity Information System. Intergovernmental Oceanographic Commission of UNESCO. https://obis.org. Accessed: 2019-08-29)
ce1d93f3-8b0f-4ee7-9a4d-0393a6ec7fea	Atlantic Reference Centre Museum of Canadian Atlantic Organisms - Invertebrates and Fishes Data	Van Guelpen, L., 2016. Atlantic Reference Centre Museum of Canadian Atlantic Organisms - Invertebrates and Fishes Data. Version 4 In OBIS Canada Digital Collections. Bedford Institute of Oceanography, Dartmouth, NS, Canada. Published by OBIS, Digital http://www.iobis.org/. Accessed on –INSERT DATE	This work is licensed under a Creative Commons Attribution (CC-BY) 4.0 License	OBIS (2019) [Atlantic Reference Centre Museum of Canadian Atlantic Organisms - Invertebrates and Fishes Data] (Available: Ocean Biodiversity Information System. Intergovernmental Oceanographic Commission of UNESCO. https://obis.org. Accessed: 2019-08-29)
d6d6fe4c-425f-4ce7-bf28-7a6befaeb413	National Museum of Natural History Vertebrate Zoology Fishes Collections	National Museum of Natural History, Smithsonian Institution NMNH Fishes Collection Database. National Museum of Natural History, Smithsonian Institution, 10th and Constitution Ave. N.W., Washington, DC 20560-0193, 2007.	This work is licensed under a Creative Commons Attribution (CC-BY) 4.0 License	OBIS (2019) [National Museum of Natural History Vertebrate Zoology Fishes Collections] (Available: Ocean Biodiversity Information System. Intergovernmental Oceanographic Commission of UNESCO. https://obis.org. Accessed: 2019-08-29)
e09c824c-cbd8-4529-b382-5306b2b3a875	Marine biodiversity atlas of the Balearic Sea	Deudero, Vallespir, Obrador 2011. Atlas de Biodiversidad Marina del Mar Balear. http://www.ba.ieo.es	This work is licensed under a Creative Commons Attribution Non Commercial (CC-BY-NC) 4.0 License	OBIS (2019) [Marine biodiversity atlas of the Balearic Sea] (Available: Ocean Biodiversity Information System. Intergovernmental Oceanographic Commission of UNESCO. https://obis.org. Accessed: 2019-08-29)
ff8b7809-41bc-40ad-8160-0e33862817a0	Biodiversity Research Museum, Academia Sinica, Taiwan	TELDAP, Biodiversity Research Museum, Academia Sinica, Taiwan (accessed through GBIF data portal, http://data.gbif.org/datasets/resource/9093, yyyy-mm-dd)	Unrestricted	OBIS (2019) [Biodiversity Research Museum, Academia Sinica, Taiwan] (Available: Ocean Biodiversity Information System. Intergovernmental Oceanographic Commission of UNESCO. https://obis.org. Accessed: 2019-08-29)

Symbolophorus evermanni

GBIF

GBIF Occurrence Download https://doi.org/10.15468/dd.t7nhkr Accessed from R via rgbif (https://github.com/ropensci/rgbif) on 2019-09-11

OBIS

OBIS (2019) Distribution records of Symbolophorus evermanni [Dataset] (Available: Ocean Biodiversity Information System. Intergovernmental Oceanographic Commission of UNESCO. www.obis.org. Accessed: 2019-08-29)

Dataset details:

Dataset_ID	Name	Citation	License	OBIS_citation
04e3fd32-b08b-4806-a016-d2dff52ae55a	Asia-Pacific Dataset	Jintsu-Uchifune, Y., Yamamoto, H. (2016) Marine organism occurrence data of the Asia-Pacific region extracted from literature. Available at https://doi.org/10.48518/00002. Accessed on yyyy-mm-dd.	This work is licensed under a Creative Commons Attribution (CC-BY) 4.0 License	OBIS (2019) [Asia-Pacific Dataset] (Available: Ocean Biodiversity Information System. Intergovernmental Oceanographic Commission of UNESCO. https://obis.org. Accessed: 2019-08-29)
10b213e6-a9c4-459e-a40c-ef9edc461b97	Marine data from the Bernice P. Bishop Museum	Pyle R (2016). Bernice P. Bishop Museum. Version 8.1. Bernice Pauahi Bishop Museum. Occurrence dataset https://doi.org/10.15468/s6ctus accessed via GBIF.org on 2018-11-16.	To the extent possible under law, the publisher has waived all rights to these data and has dedicated them to the Public Domain (CC0 1.0)	OBIS (2019) [Marine data from the Bernice P. Bishop Museum] (Available: Ocean Biodiversity Information System. Intergovernmental Oceanographic Commission of UNESCO. https://obis.org. Accessed: 2019-08-29)
270f3e70-ff9b-411d-b170-2bc914d83f26	Biological Reference Collections ICM CSIC	Olivas González F J (2016): Biological Reference Collections ICM CSIC. Institute of Marine Sciences (ICM-CSIC). https://dx.doi.org/10.15470/qlqqdx	This work is licensed under a Creative Commons Attribution Non Commercial (CC-BY-NC) 4.0 License	OBIS (2019) [Biological Reference Collections ICM CSIC] (Available: Ocean Biodiversity Information System. Intergovernmental Oceanographic Commission of UNESCO. https://obis.org. Accessed: 2019-08-29)

685b3956-c37a-433a-b661-2bb7b11cf9f8	Soviet Trawl Fishery Data (New Zealand Waters) 1964-1987	Ministry for Primary Industries (2014). Soviet Fishery Data (New Zealand Waters) 1964-1987. Southwestern Pacific OBIS, National Institute of Water and Atmospheric Research (NIWA), Wellington, New Zealand, 111883 records, Online http://nzobisipt.niwa.co.nz/resource.do?r=mbis_soviettrawl released on November 5, 2014.	This work is licensed under a Creative Commons Attribution (CC-BY) 4.0 License	OBIS (2019) [Soviet Trawl Fishery Data (New Zealand Waters) 1964-1987] (Available: Ocean Biodiversity Information System. Intergovernmental Oceanographic Commission of UNESCO. https://obis.org. Accessed: 2019-08-29)
6a5bc28f-4dfe-4cbf-8a55-7e3a843997ab	SPC NECTALIS Zooplankton/Micronekton specimens, New Caledonia 2014	Allain, V., Menkes, C., 2014. Nectalis 3 cruise, RV Alis. https://doi.org/10.17600/14004900	This work is licensed under a Creative Commons Attribution Non Commercial (CC-BY-NC) 4.0 License	OBIS (2019) [SPC NECTALIS Zooplankton/Micronekton specimens, New Caledonia 2014] (Available: Ocean Biodiversity Information System. Intergovernmental Oceanographic Commission of UNESCO. https://obis.org. Accessed: 2019-08-29)
705770e5-3474-4e69-be8b-3107a0c5610a	The fishes collection (IC) of the Muséum national d'Histoire naturelle (MNHN - Paris)	Gicim data base, Pruvost P. Causse R., 2009 http://doi.org/10.15468/tm7whu	This work is licensed under a Creative Commons Attribution (CC-BY) 4.0 License	OBIS (2019) [The fishes collection (IC) of the Muséum national d'Histoire naturelle (MNHN - Paris)] (Available: Ocean Biodiversity Information System. Intergovernmental Oceanographic Commission of UNESCO. https://obis.org. Accessed: 2019-08-29)
87a421bf-4646-49e3-89b7-409b93f2ac7c	Institution UWFC - Collection ADULT COLLECTION	No available dataset citation	Unspecified intellectual rights	OBIS (2019) [Institution UWFC - Collection ADULT COLLECTION] (Available: Ocean Biodiversity Information System. Intergovernmental Oceanographic Commission of UNESCO. https://obis.org. Accessed: 2019-08-29)
8a1ae661-e911-4967-bc06-1168fc5f2d89	iziko South African Museum - Fish Collection	iziko South African Museum - Fish Collection	Restricted	OBIS (2019) [iziko South African Museum - Fish Collection] (Available: Ocean Biodiversity Information System. Intergovernmental Oceanographic Commission of UNESCO. https://obis.org. Accessed: 2019-08-29)
9ff216fc-777e-4f9b-9860-95ed7366870d	Institution SAIAB - Collection SAIAB	No available dataset citation	Unspecified intellectual rights	OBIS (2019) [Institution SAIAB - Collection SAIAB] (Available: Ocean Biodiversity Information System. Intergovernmental Oceanographic Commission of UNESCO. https://obis.org. Accessed: 2019-08-29)

b8617377-eb1c-4db2-baa6-8788a632e810	Ichthyology Collection - Royal Ontario Museum	NA	This work is licensed under a Creative Commons Attribution Non Commercial (CC-BY-NC) 4.0 License	OBIS (2019) [Ichthyology Collection - Royal Ontario Museum] (Available: Ocean Biodiversity Information System. Intergovernmental Oceanographic Commission of UNESCO. https://obis.org. Accessed: 2019-08-29)
c24bf1c2-2c62-4056-a841-56d94e6e876a	Fish specimens	ROM Fish Collection (accessed through GBIF data portal, http://data.gbif.org/datasets/resource/660, 2012-01-20) http://doi.org/10.15468/syisbx	Unrestricted	OBIS (2019) [Fish specimens] (Available: Ocean Biodiversity Information System. Intergovernmental Oceanographic Commission of UNESCO. https://obis.org. Accessed: 2019-08-29)
d6d6fe4c-425f-4ce7-bf28-7a6befaeb413	National Museum of Natural History Vertebrate Zoology Fishes Collections	National Museum of Natural History, Smithsonian Institution NMNH Fishes Collection Database. National Museum of Natural History, Smithsonian Institution, 10th and Constitution Ave. N.W., Washington, DC 20560-0193, 2007.	This work is licensed under a Creative Commons Attribution (CC-BY) 4.0 License	OBIS (2019) [National Museum of Natural History Vertebrate Zoology Fishes Collections] (Available: Ocean Biodiversity Information System. Intergovernmental Oceanographic Commission of UNESCO. https://obis.org. Accessed: 2019-08-29)
ff8b7809-41bc-40ad-8160-0e33862817a0	Biodiversity Research Museum, Academia Sinica, Taiwan	TELDAP, Biodiversity Research Museum, Academia Sinica, Taiwan (accessed through GBIF data portal, http://data.gbif.org/datasets/resource/9093, yyyy-mm-dd)	Unrestricted	OBIS (2019) [Biodiversity Research Museum, Academia Sinica, Taiwan] (Available: Ocean Biodiversity Information System. Intergovernmental Oceanographic Commission of UNESCO. https://obis.org. Accessed: 2019-08-29)

Lampanyctus lepidolychnus

GBIF

GBIF Occurrence Download https://doi.org/10.15468/dd.jfsfnw Accessed from R via rgbif (https://github.com/ropensci/rgbif) on 2019-09-11

OBIS

OBIS (2019) Distribution records of Lampanyctus lepidolychnus [Dataset] (Available: Ocean Biodiversity Information System. Intergovernmental Oceanographic Commission of UNESCO. www.obis.org. Accessed: 2019-08-29)

Dataset details:

Dataset_ID	Name	Citation	License	OBIS_citation
270f3e70-ff9b-411d-b170-2bc914d83f26	Biological Reference Collections ICM CSIC	Olivas González F J (2016): Biological Reference Collections ICM CSIC. Institute of Marine Sciences (ICM-CSIC). https://dx.doi.org/10.15470/qlqqdx	This work is licensed under a Creative Commons Attribution Non Commercial (CC-BY-NC) 4.0 License	OBIS (2019) [Biological Reference Collections ICM CSIC] (Available: Ocean Biodiversity Information System. Intergovernmental Oceanographic Commission of UNESCO. https://obis.org. Accessed: 2019-08-29)
8a1ae661-e911-4967-bc06-1168fc5f2d89	iziko South African Museum - Fish Collection	iziko South African Museum - Fish Collection	Restricted	OBIS (2019) [iziko South African Museum - Fish Collection] (Available: Ocean Biodiversity Information System. Intergovernmental Oceanographic Commission of UNESCO. https://obis.org. Accessed: 2019-08-29)
9ff216fc-777e-4f9b-9860-95ed7366870d	Institution SAIAB - Collection SAIAB	No available dataset citation	Unspecified intellectual rights	OBIS (2019) [Institution SAIAB - Collection SAIAB] (Available: Ocean Biodiversity Information System. Intergovernmental Oceanographic Commission of UNESCO. https://obis.org. Accessed: 2019-08-29)
a4f7ee48-0d0b-4c05-a972-27a43b30db58	Institution MCM - Collection DEM	No available dataset citation	Unspecified intellectual rights	OBIS (2019) [Institution MCM - Collection DEM] (Available: Ocean Biodiversity Information System. Intergovernmental Oceanographic Commission of UNESCO. https://obis.org. Accessed: 2019-08-29)

Lampanyctus tenuiformis

GBIF

GBIF Occurrence Download https://doi.org/10.15468/dd.r3p2dk Accessed from R via rgbif (https://github.com/ropensci/rgbif) on 2019-09-11

OBIS

OBIS (2019) Distribution records of Lampanyctus tenuiformis [Dataset] (Available: Ocean Biodiversity Information System. Intergovernmental Oceanographic Commission of UNESCO. www.obis.org. Accessed: 2019-08-29)

Dataset details:

Dataset_ID	Name	Citation	License	OBIS_citation
270f3e70-ff9b-411d-b170-2bc914d83f26	Biological Reference Collections ICM CSIC	Olivas González F J (2016): Biological Reference Collections ICM CSIC. Institute of Marine Sciences (ICM-CSIC). https://dx.doi.org/10.15470/qlqqdx	This work is licensed under a Creative Commons Attribution Non Commercial (CC-BY-NC) 4.0 License	OBIS (2019) [Biological Reference Collections ICM CSIC] (Available: Ocean Biodiversity Information System. Intergovernmental Oceanographic Commission of UNESCO. https://obis.org. Accessed: 2019-08-29)
2870c548-343e-4575-ac67-a4da35182c52	Institution Shirshov Institute - Collection SKAO	No available dataset citation	Unspecified intellectual rights	OBIS (2019) [Institution Shirshov Institute - Collection SKAO] (Available: Ocean Biodiversity Information System. Intergovernmental Oceanographic Commission of UNESCO. https://obis.org. Accessed: 2019-08-29)
705770e5-3474-4e69-be8b-3107a0c5610a	The fishes collection (IC) of the Muséum national d'Histoire naturelle (MNHN - Paris)	Gicim data base, Pruvost P. Causse R., 2009 http://doi.org/10.15468/tm7whu	This work is licensed under a Creative Commons Attribution (CC-BY) 4.0 License	OBIS (2019) [The fishes collection (IC) of the Muséum national d'Histoire naturelle (MNHN - Paris)] (Available: Ocean Biodiversity Information System. Intergovernmental Oceanographic Commission of UNESCO. https://obis.org. Accessed: 2019-08-29)
8a1ae661-e911-4967-bc06-1168fc5f2d89	iziko South African Museum - Fish Collection	iziko South African Museum - Fish Collection	Restricted	OBIS (2019) [iziko South African Museum - Fish Collection] (Available: Ocean Biodiversity Information System. Intergovernmental Oceanographic Commission of UNESCO. https://obis.org. Accessed: 2019-08-29)
ce1d93f3-8b0f-4ee7-9a4d-0393a6ec7fea	Atlantic Reference Centre Museum of Canadian Atlantic Organisms - Invertebrates and Fishes Data	Van Guelpen, L., 2016. Atlantic Reference Centre Museum of Canadian Atlantic Organisms - Invertebrates and Fishes Data. Version 4 In OBIS Canada Digital Collections. Bedford Institute of Oceanography, Dartmouth, NS, Canada. Published by OBIS, Digital http://www.iobis.org/. Accessed on –INSERT DATE	This work is licensed under a Creative Commons Attribution (CC-BY) 4.0 License	OBIS (2019) [Atlantic Reference Centre Museum of Canadian Atlantic Organisms - Invertebrates and Fishes Data] (Available: Ocean Biodiversity Information System. Intergovernmental Oceanographic Commission of UNESCO. https://obis.org. Accessed: 2019-08-29)

d6d6fe4c-425f-4ce7-bf28-7a6befaeb413	National Museum of Natural History Vertebrate Zoology Fishes Collections	National Museum of Natural History, Smithsonian Institution NMNH Fishes Collection Database. National Museum of Natural History, Smithsonian Institution, 10th and Constitution Ave. N.W., Washington, DC 20560-0193, 2007.	This work is licensed under a Creative Commons Attribution (CC-BY) 4.0 License	OBIS (2019) [National Museum of Natural History Vertebrate Zoology Fishes Collections] (Available: Ocean Biodiversity Information System. Intergovernmental Oceanographic Commission of UNESCO. https://obis.org. Accessed: 2019-08-29)

Gonichthys barnesi

GBIF

GBIF Occurrence Download https://doi.org/10.15468/dd.8tgv6a Accessed from R via rgbif (https://github.com/ropensci/rgbif) on 2019-09-11

OBIS

OBIS (2019) Distribution records of Gonichthys barnesi [Dataset] (Available: Ocean Biodiversity Information System. Intergovernmental Oceanographic Commission of UNESCO. www.obis.org. Accessed: 2019-08-29)

Dataset details:

Dataset_ID	Name	Citation	License	OBIS_citation
8a1ae661-e911-4967-bc06-1168fc5f2d89	iziko South African Museum - Fish Collection	iziko South African Museum - Fish Collection	Restricted	OBIS (2019) [iziko South African Museum - Fish Collection] (Available: Ocean Biodiversity Information System. Intergovernmental Oceanographic Commission of UNESCO. https://obis.org. Accessed: 2019-08-29)
d6d6fe4c-425f-4ce7-bf28-7a6befaeb413	National Museum of Natural History Vertebrate Zoology Fishes Collections	National Museum of Natural History, Smithsonian Institution NMNH Fishes Collection Database. National Museum of Natural History, Smithsonian Institution, 10th and Constitution Ave. N.W., Washington, DC 20560-0193, 2007.	This work is licensed under a Creative Commons Attribution (CC-BY) 4.0 License	OBIS (2019) [National Museum of Natural History Vertebrate Zoology Fishes Collections] (Available: Ocean Biodiversity Information System. Intergovernmental Oceanographic Commission of UNESCO. https://obis.org. Accessed: 2019-08-29)

Lampanyctus alatus

GBIF

GBIF Occurrence Download https://doi.org/10.15468/dd.hcj3r3 Accessed from R via rgbif (https://github.com/ropensci/rgbif) on 2019-09-11

OBIS

OBIS (2019) Distribution records of Lampanyctus alatus [Dataset] (Available: Ocean Biodiversity Information System. Intergovernmental Oceanographic Commission of UNESCO. www.obis.org. Accessed: 2019-08-29)

Dataset details:

Dataset_ID	Name	Citation	License	OBIS_citation
0332e1b5-5525-4301-9659-ef3da3e4e2b6	MARMAP Isaacs-Kidd Midwater Trawl 1990-2009	Marcel Reichert, 2010, MARMAP Isaacs-Kidd Midwater Trawl 1990-2009, SCDNR/NOAA MARMAP Program, SCDNR MARMAP Aggregate data surveys, The Marine Resources Monitoring, Assessment, and Prediction (MARMAP) Program, Marine Resources Research Institute, South Carolina Department of Natural Resources, P. O. Box 12559, Charleston SC 29422-2559, U.S.A.Retrieved from http://www.usgs.gov/obis-usa/	Restricted	OBIS (2019) [MARMAP Isaacs-Kidd Midwater Trawl 1990-2009] (Available: Ocean Biodiversity Information System. Intergovernmental Oceanographic Commission of UNESCO. https://obis.org. Accessed: 2019-08-29)
1f59030f-f116-4c34-915e-1882d819cda3	Institution Southampton Oceanography Ceter - Collection discovery	No available dataset citation	Unspecified intellectual rights	OBIS (2019) [Institution Southampton Oceanography Ceter - Collection discovery] (Available: Ocean Biodiversity Information System. Intergovernmental Oceanographic Commission of UNESCO. https://obis.org. Accessed: 2019-08-29)
270f3e70-ff9b-411d-b170-2bc914d83f26	Biological Reference Collections ICM CSIC	Olivas González F J (2016): Biological Reference Collections ICM CSIC. Institute of Marine Sciences (ICM-CSIC). https://dx.doi.org/10.15470/qlqqdx	This work is licensed under a Creative Commons Attribution Non Commercial (CC-BY-NC) 4.0 License	OBIS (2019) [Biological Reference Collections ICM CSIC] (Available: Ocean Biodiversity Information System. Intergovernmental Oceanographic Commission of UNESCO. https://obis.org. Accessed: 2019-08-29)

2870c548-343e-4575-ac67-a4da35182c52	Institution Shirshov Institute - Collection SKAO	No available dataset citation	Unspecified intellectual rights	OBIS (2019) [Institution Shirshov Institute - Collection SKAO] (Available: Ocean Biodiversity Information System. Intergovernmental Oceanographic Commission of UNESCO. https://obis.org. Accessed: 2019-08-29)
685b3956-c37a-433a-b661-2bb7b11cf9f8	Soviet Trawl Fishery Data (New Zealand Waters) 1964-1987	Ministry for Primary Industries (2014). Soviet Fishery Data (New Zealand Waters) 1964-1987. Southwestern Pacific OBIS, National Institute of Water and Atmospheric Research (NIWA), Wellington, New Zealand, 111883 records, Online http://nzobisipt.niwa.co.nz/resource.do?r=mbis_soviettrawl released on November 5, 2014.	This work is licensed under a Creative Commons Attribution (CC-BY) 4.0 License	OBIS (2019) [Soviet Trawl Fishery Data (New Zealand Waters) 1964-1987] (Available: Ocean Biodiversity Information System. Intergovernmental Oceanographic Commission of UNESCO. https://obis.org. Accessed: 2019-08-29)
6a5bc28f-4dfe-4cbf-8a55-7e3a843997ab	SPC NECTALIS Zooplankton/Micronekton specimens, New Caledonia 2014	Allain, V., Menkes, C., 2014. Nectalis 3 cruise, RV Alis. https://doi.org/10.17600/14004900	This work is licensed under a Creative Commons Attribution Non Commercial (CC-BY-NC) 4.0 License	OBIS (2019) [SPC NECTALIS Zooplankton/Micronekton specimens, New Caledonia 2014] (Available: Ocean Biodiversity Information System. Intergovernmental Oceanographic Commission of UNESCO. https://obis.org. Accessed: 2019-08-29)
6f44d12a-ce3a-4589-9b3a-69234ee207d1	MARMAP Yankee Trawl 1990-2009	Marcel Reichert, 2010, MARMAP Yankee Trawl 1990-2009, SCDNR/NOAA MARMAP Program, SCDNR MARMAP Aggregate data surveys, The Marine Resources Monitoring, Assessment, and Prediction (MARMAP) Program, Marine Resources Research Institute, South Carolina Department of Natural Resources, P. O. Box 12559, Charleston SC 29422-2559, U.S.A. Retrieve from http://obisusa.nbii.gov	Restricted	OBIS (2019) [MARMAP Yankee Trawl 1990-2009] (Available: Ocean Biodiversity Information System. Intergovernmental Oceanographic Commission of UNESCO. https://obis.org. Accessed: 2019-08-29)
705770e5-3474-4e69-be8b-3107a0c5610a	The fishes collection (IC) of the Muséum national d'Histoire naturelle (MNHN - Paris)	Gicim data base, Pruvost P. Causse R., 2009 http://doi.org/10.15468/tm7whu	This work is licensed under a Creative Commons Attribution (CC-BY) 4.0 License	OBIS (2019) [The fishes collection (IC) of the Muséum national d'Histoire naturelle (MNHN - Paris)] (Available: Ocean Biodiversity Information System. Intergovernmental Oceanographic Commission of UNESCO. https://obis.org. Accessed: 2019-08-29)

7e4228e5-a962-4b01-952f-7bf33e213a9c	BioChem: Sameoto zooplankton collection	Sameoto, D.D., Kennedy, M., Spry, J.S, Spry, J.M. (2013). Zooplankton datasets collected using the BIONESS sampler, ring nets and an Icelandic high speed sampler, 1967-2006. OBIS Canada Digital Collections. Published by OBIS http://www.iobis.org/. Accessed on –INSERT DATE	rights: http://data.gc.ca/eng/open-government-licence-canada & http://www.canadensys.net/norms rights holder: Her Majesty the Queen in right of Canada, as represented by the Minister of Fisheries and Oceans	OBIS (2019) [BioChem: Sameoto zooplankton collection] (Available: Ocean Biodiversity Information System. Intergovernmental Oceanographic Commission of UNESCO. https://obis.org. Accessed: 2019-08-29)
8a1ae661-e911-4967-bc06-1168fc5f2d89	iziko South African Museum - Fish Collection	iziko South African Museum - Fish Collection	Restricted	OBIS (2019) [iziko South African Museum - Fish Collection] (Available: Ocean Biodiversity Information System. Intergovernmental Oceanographic Commission of UNESCO. https://obis.org. Accessed: 2019-08-29)
a4f7ee48-0d0b-4c05-a972-27a43b30db58	Institution MCM - Collection DEM	No available dataset citation	Unspecified intellectual rights	OBIS (2019) [Institution MCM - Collection DEM] (Available: Ocean Biodiversity Information System. Intergovernmental Oceanographic Commission of UNESCO. https://obis.org. Accessed: 2019-08-29)
b8617377-eb1c-4db2-baa6-8788a632e810	Ichthyology Collection - Royal Ontario Museum	NA	This work is licensed under a Creative Commons Attribution Non Commercial (CC-BY-NC) 4.0 License	OBIS (2019) [Ichthyology Collection - Royal Ontario Museum] (Available: Ocean Biodiversity Information System. Intergovernmental Oceanographic Commission of UNESCO. https://obis.org. Accessed: 2019-08-29)
c24bf1c2-2c62-4056-a841-56d94e6e876a	Fish specimens	ROM Fish Collection (accessed through GBIF data portal, http://data.gbif.org/datasets/resource/660, 2012-01-20) http://doi.org/10.15468/syisbx	Unrestricted	OBIS (2019) [Fish specimens] (Available: Ocean Biodiversity Information System. Intergovernmental Oceanographic Commission of UNESCO. https://obis.org. Accessed: 2019-08-29)

cc8f28ce-e48d-4945-abfe-9d150a22dcd6	Hamburg pelagic fish database	Post, A. 1987. Pelagic transects of FRVs "Walther Herwig" and "Anton Dohrn" in the Atlantic Ocean 1966 to 1986. Mitt. Inst. f. Seefischerei d. BfaFi Hamburg, 42: 1-68.	This work is licensed under a Creative Commons Attribution (CC-BY) 4.0 License	OBIS (2019) [Hamburg pelagic fish database] (Available: Ocean Biodiversity Information System. Intergovernmental Oceanographic Commission of UNESCO. https://obis.org. Accessed: 2019-08-29)
ce1d93f3-8b0f-4ee7-9a4d-0393a6ec7fea	Atlantic Reference Centre Museum of Canadian Atlantic Organisms - Invertebrates and Fishes Data	Van Guelpen, L., 2016. Atlantic Reference Centre Museum of Canadian Atlantic Organisms - Invertebrates and Fishes Data. Version 4 In OBIS Canada Digital Collections. Bedford Institute of Oceanography, Dartmouth, NS, Canada. Published by OBIS, Digital http://www.iobis.org/. Accessed on –INSERT DATE	This work is licensed under a Creative Commons Attribution (CC-BY) 4.0 License	OBIS (2019) [Atlantic Reference Centre Museum of Canadian Atlantic Organisms - Invertebrates and Fishes Data] (Available: Ocean Biodiversity Information System. Intergovernmental Oceanographic Commission of UNESCO. https://obis.org. Accessed: 2019-08-29)
cfc56587-48c3-4e3d-9350-3a4d9a28b681	Institution NOAA, NMFS, Northeast Fisheries Science Center - Collection DEEPWATER SYSTEMATICS	No available dataset citation	Unspecified intellectual rights	OBIS (2019) [Institution NOAA, NMFS, Northeast Fisheries Science Center - Collection DEEPWATER SYSTEMATICS] (Available: Ocean Biodiversity Information System. Intergovernmental Oceanographic Commission of UNESCO. https://obis.org. Accessed: 2019-08-29)
d6d6fe4c-425f-4ce7-bf28-7a6befaeb413	National Museum of Natural History Vertebrate Zoology Fishes Collections	National Museum of Natural History, Smithsonian Institution NMNH Fishes Collection Database. National Museum of Natural History, Smithsonian Institution, 10th and Constitution Ave. N.W., Washington, DC 20560-0193, 2007.	This work is licensed under a Creative Commons Attribution (CC-BY) 4.0 License	OBIS (2019) [National Museum of Natural History Vertebrate Zoology Fishes Collections] (Available: Ocean Biodiversity Information System. Intergovernmental Oceanographic Commission of UNESCO. https://obis.org. Accessed: 2019-08-29)
ff8b7809-41bc-40ad-8160-0e33862817a0	Biodiversity Research Museum, Academia Sinica, Taiwan	TELDAP, Biodiversity Research Museum, Academia Sinica, Taiwan (accessed through GBIF data portal, http://data.gbif.org/datasets/resource/9093, yyyy-mm-dd)	Unrestricted	OBIS (2019) [Biodiversity Research Museum, Academia Sinica, Taiwan] (Available: Ocean Biodiversity Information System. Intergovernmental Oceanographic Commission of UNESCO. https://obis.org. Accessed: 2019-08-29)

Benthosema suborbitale

GBIF

GBIF Occurrence Download https://doi.org/10.15468/dd.w3rdqk Accessed from R via rgbif (https://github.com/ropensci/rgbif) on 2019-09-11

OBIS

OBIS (2019) Distribution records of Benthosema suborbitale [Dataset] (Available: Ocean Biodiversity Information System. Intergovernmental Oceanographic Commission of UNESCO. www.obis.org. Accessed: 2019-08-29)

Dataset details:

Dataset_ID	Name	Citation	License	OBIS_citation
0332e1b5-5525-4301-9659-ef3da3e4e2b6	MARMAP Isaacs-Kidd Midwater Trawl 1990-2009	Marcel Reichert, 2010, MARMAP Isaacs-Kidd Midwater Trawl 1990-2009, SCDNR/NOAA MARMAP Program, SCDNR MARMAP Aggregate data surveys, The Marine Resources Monitoring, Assessment, and Prediction (MARMAP) Program, Marine Resources Research Institute, South Carolina Department of Natural Resources, P. O. Box 12559, Charleston SC 29422-2559, U.S.A.Retrieved from http://www.usgs.gov/obis-usa/	Restricted	OBIS (2019) [MARMAP Isaacs-Kidd Midwater Trawl 1990-2009] (Available: Ocean Biodiversity Information System. Intergovernmental Oceanographic Commission of UNESCO. https://obis.org. Accessed: 2019-08-29)
04e3fd32-b08b-4806-a016-d2dff52ae55a	Asia-Pacific Dataset	Jintsu-Uchifune, Y., Yamamoto, H. (2016) Marine organism occurrence data of the Asia-Pacific region extracted from literature. Available at https://doi.org/10.48518/00002. Accessed on yyyy-mm-dd.	This work is licensed under a Creative Commons Attribution (CC-BY) 4.0 License	OBIS (2019) [Asia-Pacific Dataset] (Available: Ocean Biodiversity Information System. Intergovernmental Oceanographic Commission of UNESCO. https://obis.org. Accessed: 2019-08-29)
10b213e6-a9c4-459e-a40c-ef9edc461b97	Marine data from the Bernice P. Bishop Museum	Pyle R (2016). Bernice P. Bishop Museum. Version 8.1. Bernice Pauahi Bishop Museum. Occurrence dataset https://doi.org/10.15468/s6ctus accessed via GBIF.org on 2018-11-16.	To the extent possible under law, the publisher has waived all rights to these data and has dedicated them to the Public Domain (CC0 1.0)	OBIS (2019) [Marine data from the Bernice P. Bishop Museum] (Available: Ocean Biodiversity Information System. Intergovernmental Oceanographic Commission of UNESCO. https://obis.org. Accessed: 2019-08-29)

1f59030f-f116-4c34-915e-1882d819cda3	Institution Southampton Oceanography Ceter - Collection discovery	No available dataset citation	Unspecified intellectual rights	OBIS (2019) [Institution Southampton Oceanography Ceter - Collection discovery] (Available: Ocean Biodiversity Information System. Intergovernmental Oceanographic Commission of UNESCO. https://obis.org. Accessed: 2019-08-29)
231dc661-d8d9-4d49-9ff0-45bd21a08541	New Zealand fish and squid distributions from research bottom trawls 1964-2008	NIWA (2014). New Zealand fish and squid distributions from research bottom trawls. Southwestern Pacific OBIS, National Institute of Water and Atmospheric Research (NIWA), Wellington, New Zealand, 486781 records, Online http://nzobisipt.niwa.co.nz/resource.do?r=obisprovider released on May 8, 2014.	This work is licensed under a Creative Commons Attribution (CC-BY) 4.0 License	OBIS (2019) [New Zealand fish and squid distributions from research bottom trawls 1964-2008] (Available: Ocean Biodiversity Information System. Intergovernmental Oceanographic Commission of UNESCO. https://obis.org. Accessed: 2019-08-29)
270f3e70-ff9b-411d-b170-2bc914d83f26	Biological Reference Collections ICM CSIC	Olivas González F J (2016): Biological Reference Collections ICM CSIC. Institute of Marine Sciences (ICM-CSIC). https://dx.doi.org/10.15470/qlqqdx	This work is licensed under a Creative Commons Attribution Non Commercial (CC-BY-NC) 4.0 License	OBIS (2019) [Biological Reference Collections ICM CSIC] (Available: Ocean Biodiversity Information System. Intergovernmental Oceanographic Commission of UNESCO. https://obis.org. Accessed: 2019-08-29)
2870c548-343e-4575-ac67-a4da35182c52	Institution Shirshov Institute - Collection SKAO	No available dataset citation	Unspecified intellectual rights	OBIS (2019) [Institution Shirshov Institute - Collection SKAO] (Available: Ocean Biodiversity Information System. Intergovernmental Oceanographic Commission of UNESCO. https://obis.org. Accessed: 2019-08-29)
308a501c-a187-498e-a8bd-9cf3d2b70bd9	Institution REVIZEE - Collection Ictioplankton	No available dataset citation	Unspecified intellectual rights	OBIS (2019) [Institution REVIZEE - Collection Ictioplankton] (Available: Ocean Biodiversity Information System. Intergovernmental Oceanographic Commission of UNESCO. https://obis.org. Accessed: 2019-08-29)
4354345d-7faf-4376-b326-ffbc04b6b0cd	No available dataset name	No available dataset citation	Unspecified intellectual rights	OBIS (2019) [No available dataset name] (Available: Ocean Biodiversity Information System. Intergovernmental Oceanographic Commission of UNESCO. https://obis.org. Accessed: 2019-08-29)

5f2da252-6d49-4c9f-b3b3-1db53d75b345	MARMAP Bongo Nets 1990-2009	Marcel Reichert, 2010, MARMAP Bongo Nets 1990-2009, SCDNR/NOAA MARMAP Program, SCDNR MARMAP Aggregate data surveys, The Marine Resources Monitoring, Assessment, and Prediction (MARMAP) Program, Marine Resources Research Institute, South Carolina Department of Natural Resources, P. O. Box 12559, Charleston SC 29422-2559, U.S.A.Retrieved from http://www.usgs.gov/obis-usa/	Restricted	OBIS (2019) [MARMAP Bongo Nets 1990-2009] (Available: Ocean Biodiversity Information System. Intergovernmental Oceanographic Commission of UNESCO. https://obis.org. Accessed: 2019-08-29)
6a5bc28f-4dfe-4cbf-8a55-7e3a843997ab	SPC NECTALIS Zooplankton/Micronekton specimens, New Caledonia 2014	Allain, V., Menkes, C., 2014. Nectalis 3 cruise, RV Alis. https://doi.org/10.17600/14004900	This work is licensed under a Creative Commons Attribution Non Commercial (CC-BY-NC) 4.0 License	OBIS (2019) [SPC NECTALIS Zooplankton/Micronekton specimens, New Caledonia 2014] (Available: Ocean Biodiversity Information System. Intergovernmental Oceanographic Commission of UNESCO. https://obis.org. Accessed: 2019-08-29)
705770e5-3474-4e69-be8b-3107a0c5610a	The fishes collection (IC) of the Muséum national d'Histoire naturelle (MNHN - Paris)	Gicim data base, Pruvost P. Causse R., 2009 http://doi.org/10.15468/tm7whu	This work is licensed under a Creative Commons Attribution (CC-BY) 4.0 License	OBIS (2019) [The fishes collection (IC) of the Muséum national d'Histoire naturelle (MNHN - Paris)] (Available: Ocean Biodiversity Information System. Intergovernmental Oceanographic Commission of UNESCO. https://obis.org. Accessed: 2019-08-29)
7e4228e5-a962-4b01-952f-7bf33e213a9c	BioChem: Sameoto zooplankton collection	Sameoto, D.D., Kennedy, M., Spry, J.S, Spry, J.M. (2013). Zooplankton datasets collected using the BIONESS sampler, ring nets and an Icelandic high speed sampler, 1967-2006. OBIS Canada Digital Collections. Published by OBIS http://www.iobis.org/. Accessed on –INSERT DATE	rights: http://data.gc.ca/eng/open-government-licence-canada & http://www.canadensys.net/norms rights holder: Her Majesty the Queen in right of Canada, as represented by the Minister of Fisheries and Oceans	OBIS (2019) [BioChem: Sameoto zooplankton collection] (Available: Ocean Biodiversity Information System. Intergovernmental Oceanographic Commission of UNESCO. https://obis.org. Accessed: 2019-08-29)

8629ec33-be4b-4384-933f-a511fbc29967	MAR-ECO 2004	Wenneck, T. de Lange, Falkenhaug, T. and O.A. Bergstad. 2008. Strategies, methods, and technologies adopted on the RV G.O. Sars MAR-ECO expedition to the mid-Atlantic Ridge in 2004. Deep-sea Research II. 55: 6-28.	This work is licensed under a Creative Commons Attribution (CC-BY) 4.0 License	OBIS (2019) [MAR-ECO 2004] (Available: Ocean Biodiversity Information System. Intergovernmental Oceanographic Commission of UNESCO. https://obis.org. Accessed: 2019-08-29)
8a1ae661-e911-4967-bc06-1168fc5f2d89	iziko South African Museum - Fish Collection	iziko South African Museum - Fish Collection	Restricted	OBIS (2019) [iziko South African Museum - Fish Collection] (Available: Ocean Biodiversity Information System. Intergovernmental Oceanographic Commission of UNESCO. https://obis.org. Accessed: 2019-08-29)
a4f7ee48-0d0b-4c05-a972-27a43b30db58	Institution - Collection	No available dataset citation	Unspecified intellectual rights	OBIS (2019) [Institution - Collection] (Available: Ocean Biodiversity Information System. Intergovernmental Oceanographic Commission of UNESCO. https://obis.org. Accessed: 2019-08-29)
a4f7ee48-0d0b-4c05-a972-27a43b30db58	Institution MCM - Collection DEM	No available dataset citation	Unspecified intellectual rights	OBIS (2019) [Institution MCM - Collection DEM] (Available: Ocean Biodiversity Information System. Intergovernmental Oceanographic Commission of UNESCO. https://obis.org. Accessed: 2019-08-29)
b8617377-eb1c-4db2-baa6-8788a632e810	Ichthyology Collection - Royal Ontario Museum	NA	This work is licensed under a Creative Commons Attribution Non Commercial (CC-BY-NC) 4.0 License	OBIS (2019) [Ichthyology Collection - Royal Ontario Museum] (Available: Ocean Biodiversity Information System. Intergovernmental Oceanographic Commission of UNESCO. https://obis.org. Accessed: 2019-08-29)
c24bf1c2-2c62-4056-a841-56d94e6e876a	Fish specimens	ROM Fish Collection (accessed through GBIF data portal, http://data.gbif.org/datasets/resource/660, 2012-01-20) http://doi.org/10.15468/syisbx	Unrestricted	OBIS (2019) [Fish specimens] (Available: Ocean Biodiversity Information System. Intergovernmental Oceanographic Commission of UNESCO. https://obis.org. Accessed: 2019-08-29)
cc8f28ce-e48d-4945-abfe-9d150a22dcd6	Hamburg pelagic fish database	Post, A. 1987. Pelagic transects of FRVs "Walther Herwig" and "Anton Dohrn" in the Atlantic Ocean 1966 to 1986. Mitt. Inst. f. Seefischerei d. BfaFi Hamburg, 42: 1-68.	This work is licensed under a Creative Commons Attribution (CC-BY) 4.0 License	OBIS (2019) [Hamburg pelagic fish database] (Available: Ocean Biodiversity Information System. Intergovernmental Oceanographic Commission of UNESCO. https://obis.org. Accessed: 2019-08-29)

ce1d93f3-8b0f-4ee7-9a4d-0393a6ec7fea	Atlantic Reference Centre Museum of Canadian Atlantic Organisms - Invertebrates and Fishes Data	Van Guelpen, L., 2016. Atlantic Reference Centre Museum of Canadian Atlantic Organisms - Invertebrates and Fishes Data. Version 4 In OBIS Canada Digital Collections. Bedford Institute of Oceanography, Dartmouth, NS, Canada. Published by OBIS, Digital http://www.iobis.org/. Accessed on –INSERT DATE	This work is licensed under a Creative Commons Attribution (CC-BY) 4.0 License	OBIS (2019) [Atlantic Reference Centre Museum of Canadian Atlantic Organisms - Invertebrates and Fishes Data] (Available: Ocean Biodiversity Information System. Intergovernmental Oceanographic Commission of UNESCO. https://obis.org. Accessed: 2019-08-29)
d6d6fe4c-425f-4ce7-bf28-7a6befaeb413	National Museum of Natural History Vertebrate Zoology Fishes Collections	National Museum of Natural History, Smithsonian Institution NMNH Fishes Collection Database. National Museum of Natural History, Smithsonian Institution, 10th and Constitution Ave. N.W., Washington, DC 20560-0193, 2007.	This work is licensed under a Creative Commons Attribution (CC-BY) 4.0 License	OBIS (2019) [National Museum of Natural History Vertebrate Zoology Fishes Collections] (Available: Ocean Biodiversity Information System. Intergovernmental Oceanographic Commission of UNESCO. https://obis.org. Accessed: 2019-08-29)

Diogenichthys atlanticus

GBIF

GBIF Occurrence Download https://doi.org/10.15468/dd.682mnr Accessed from R via rgbif (https://github.com/ropensci/rgbif) on 2019-09-11

OBIS

OBIS (2019) Distribution records of Diogenichthys atlanticus [Dataset] (Available: Ocean Biodiversity Information System. Intergovernmental Oceanographic Commission of UNESCO. www.obis.org. Accessed: 2019-08-29)

Dataset details:

Dataset_ID	Name	Citation	License	OBIS_citation

0332e1b5-5525-4301-9659-ef3da3e4e2b6	MARMAP Isaacs-Kidd Midwater Trawl 1990-2009	Marcel Reichert, 2010, MARMAP Isaacs-Kidd Midwater Trawl 1990-2009, SCDNR/NOAA MARMAP Program, SCDNR MARMAP Aggregate data surveys, The Marine Resources Monitoring, Assessment, and Prediction (MARMAP) Program, Marine Resources Research Institute, South Carolina Department of Natural Resources, P. O. Box 12559, Charleston SC 29422-2559, U.S.A.Retrieved from http://www.usgs.gov/obis-usa/	Restricted	OBIS (2019) [MARMAP Isaacs-Kidd Midwater Trawl 1990-2009] (Available: Ocean Biodiversity Information System. Intergovernmental Oceanographic Commission of UNESCO. https://obis.org. Accessed: 2019-08-29)
04e3fd32-b08b-4806-a016-d2dff52ae55a	Asia-Pacific Dataset	Jintsu-Uchifune, Y., Yamamoto, H. (2016) Marine organism occurrence data of the Asia-Pacific region extracted from literature. Available at https://doi.org/10.48518/00002. Accessed on yyyy-mm-dd.	This work is licensed under a Creative Commons Attribution (CC-BY) 4.0 License	OBIS (2019) [Asia-Pacific Dataset] (Available: Ocean Biodiversity Information System. Intergovernmental Oceanographic Commission of UNESCO. https://obis.org. Accessed: 2019-08-29)
1f59030f-f116-4c34-915e-1882d819cda3	Institution Southampton Oceanography Ceter - Collection discovery	No available dataset citation	Unspecified intellectual rights	OBIS (2019) [Institution Southampton Oceanography Ceter - Collection discovery] (Available: Ocean Biodiversity Information System. Intergovernmental Oceanographic Commission of UNESCO. https://obis.org. Accessed: 2019-08-29)
2870c548-343e-4575-ac67-a4da35182c52	Institution Shirshov Institute - Collection SKAO	No available dataset citation	Unspecified intellectual rights	OBIS (2019) [Institution Shirshov Institute - Collection SKAO] (Available: Ocean Biodiversity Information System. Intergovernmental Oceanographic Commission of UNESCO. https://obis.org. Accessed: 2019-08-29)
4354345d-7faf-4376-b326-ffbc04b6b0cd	No available dataset name	No available dataset citation	Unspecified intellectual rights	OBIS (2019) [No available dataset name] (Available: Ocean Biodiversity Information System. Intergovernmental Oceanographic Commission of UNESCO. https://obis.org. Accessed: 2019-08-29)

5f2da252-6d49-4c9f-b3b3-1db53d75b345	MARMAP Bongo Nets 1990-2009	Marcel Reichert, 2010, MARMAP Bongo Nets 1990-2009, SCDNR/NOAA MARMAP Program, SCDNR MARMAP Aggregate data surveys, The Marine Resources Monitoring, Assessment, and Prediction (MARMAP) Program, Marine Resources Research Institute, South Carolina Department of Natural Resources, P. O. Box 12559, Charleston SC 29422-2559, U.S.A.Retrieved from http://www.usgs.gov/obis-usa/	Restricted	OBIS (2019) [MARMAP Bongo Nets 1990-2009] (Available: Ocean Biodiversity Information System. Intergovernmental Oceanographic Commission of UNESCO. https://obis.org. Accessed: 2019-08-29)
6a5bc28f-4dfe-4cbf-8a55-7e3a843997ab	SPC NECTALIS Zooplankton/Micronekton specimens, New Caledonia 2014	Allain, V., Menkes, C., 2014. Nectalis 3 cruise, RV Alis. https://doi.org/10.17600/14004900	This work is licensed under a Creative Commons Attribution Non Commercial (CC-BY-NC) 4.0 License	OBIS (2019) [SPC NECTALIS Zooplankton/Micronekton specimens, New Caledonia 2014] (Available: Ocean Biodiversity Information System. Intergovernmental Oceanographic Commission of UNESCO. https://obis.org. Accessed: 2019-08-29)
705770e5-3474-4e69-be8b-3107a0c5610a	The fishes collection (IC) of the Muséum national d'Histoire naturelle (MNHN - Paris)	Gicim data base, Pruvost P. Causse R., 2009 http://doi.org/10.15468/tm7whu	This work is licensed under a Creative Commons Attribution (CC-BY) 4.0 License	OBIS (2019) [The fishes collection (IC) of the Muséum national d'Histoire naturelle (MNHN - Paris)] (Available: Ocean Biodiversity Information System. Intergovernmental Oceanographic Commission of UNESCO. https://obis.org. Accessed: 2019-08-29)
7e4228e5-a962-4b01-952f-7bf33e213a9c	BioChem: Sameoto zooplankton collection	Sameoto, D.D., Kennedy, M., Spry, J.S, Spry, J.M. (2013). Zooplankton datasets collected using the BIONESS sampler, ring nets and an Icelandic high speed sampler, 1967-2006. OBIS Canada Digital Collections. Published by OBIS http://www.iobis.org/. Accessed on –INSERT DATE	rights: http://data.gc.ca/eng/open-government-licence-canada & http://www.canadensys.net/norms rights holder: Her Majesty the Queen in right of Canada, as represented by the Minister of Fisheries and Oceans	OBIS (2019) [BioChem: Sameoto zooplankton collection] (Available: Ocean Biodiversity Information System. Intergovernmental Oceanographic Commission of UNESCO. https://obis.org. Accessed: 2019-08-29)

8629ec33-be4b-4384-933f-a511fbc29967	MAR-ECO 2004	Wenneck, T. de Lange, Falkenhaug, T. and O.A. Bergstad. 2008. Strategies, methods, and technologies adopted on the RV G.O. Sars MAR-ECO expedition to the mid-Atlantic Ridge in 2004. Deep-sea Research II. 55: 6-28.	This work is licensed under a Creative Commons Attribution (CC-BY) 4.0 License	OBIS (2019) [MAR-ECO 2004] (Available: Ocean Biodiversity Information System. Intergovernmental Oceanographic Commission of UNESCO. https://obis.org. Accessed: 2019-08-29)
8a1ae661-e911-4967-bc06-1168fc5f2d89	iziko South African Museum - Fish Collection	iziko South African Museum - Fish Collection	Restricted	OBIS (2019) [iziko South African Museum - Fish Collection] (Available: Ocean Biodiversity Information System. Intergovernmental Oceanographic Commission of UNESCO. https://obis.org. Accessed: 2019-08-29)
b0a7add2-dd9e-4020-9ca4-5df048c8f6a2	Bigood	Fabri, M-C. et al., Ifremer BIOCEAN database (Deep Sea Benthic Fauna). Institut Français de Recherche pour l'Exploitation de la Mer, Ifremer, Issy-les-Moulineaux, France. World Wide Web electronic publication, http://www.ifremer.fr/isi/biocean	This work is licensed under a Creative Commons Attribution (CC-BY) 4.0 License	OBIS (2019) [Bigood] (Available: Ocean Biodiversity Information System. Intergovernmental Oceanographic Commission of UNESCO. https://obis.org. Accessed: 2019-08-29)
b8617377-eb1c-4db2-baa6-8788a632e810	Ichthyology Collection - Royal Ontario Museum	NA	This work is licensed under a Creative Commons Attribution Non Commercial (CC-BY-NC) 4.0 License	OBIS (2019) [Ichthyology Collection - Royal Ontario Museum] (Available: Ocean Biodiversity Information System. Intergovernmental Oceanographic Commission of UNESCO. https://obis.org. Accessed: 2019-08-29)
c24bf1c2-2c62-4056-a841-56d94e6e876a	Fish specimens	ROM Fish Collection (accessed through GBIF data portal, http://data.gbif.org/datasets/resource/660, 2012-01-20) http://doi.org/10.15468/syisbx	Unrestricted	OBIS (2019) [Fish specimens] (Available: Ocean Biodiversity Information System. Intergovernmental Oceanographic Commission of UNESCO. https://obis.org. Accessed: 2019-08-29)
cc8f28ce-e48d-4945-abfe-9d150a22dcd6	Hamburg pelagic fish database	Post, A. 1987. Pelagic transects of FRVs "Walther Herwig" and "Anton Dohrn" in the Atlantic Ocean 1966 to 1986. Mitt. Inst. f. Seefischerei d. BfaFi Hamburg, 42: 1-68.	This work is licensed under a Creative Commons Attribution (CC-BY) 4.0 License	OBIS (2019) [Hamburg pelagic fish database] (Available: Ocean Biodiversity Information System. Intergovernmental Oceanographic Commission of UNESCO. https://obis.org. Accessed: 2019-08-29)

ce1d93f3-8b0f-4ee7-9a4d-0393a6ec7fea	Atlantic Reference Centre Museum of Canadian Atlantic Organisms - Invertebrates and Fishes Data	Van Guelpen, L., 2016. Atlantic Reference Centre Museum of Canadian Atlantic Organisms - Invertebrates and Fishes Data. Version 4 In OBIS Canada Digital Collections. Bedford Institute of Oceanography, Dartmouth, NS, Canada. Published by OBIS, Digital http://www.iobis.org/. Accessed on –INSERT DATE	This work is licensed under a Creative Commons Attribution (CC-BY) 4.0 License	OBIS (2019) [Atlantic Reference Centre Museum of Canadian Atlantic Organisms - Invertebrates and Fishes Data] (Available: Ocean Biodiversity Information System. Intergovernmental Oceanographic Commission of UNESCO. https://obis.org. Accessed: 2019-08-29)
d6d6fe4c-425f-4ce7-bf28-7a6befaeb413	National Museum of Natural History Vertebrate Zoology Fishes Collections	National Museum of Natural History, Smithsonian Institution NMNH Fishes Collection Database. National Museum of Natural History, Smithsonian Institution, 10th and Constitution Ave. N.W., Washington, DC 20560-0193, 2007.	This work is licensed under a Creative Commons Attribution (CC-BY) 4.0 License	OBIS (2019) [National Museum of Natural History Vertebrate Zoology Fishes Collections] (Available: Ocean Biodiversity Information System. Intergovernmental Oceanographic Commission of UNESCO. https://obis.org. Accessed: 2019-08-29)

Hygophum hygomii

GBIF

GBIF Occurrence Download https://doi.org/10.15468/dd.uypnkr Accessed from R via rgbif (https://github.com/ropensci/rgbif) on 2019-09-11

OBIS

OBIS (2019) Distribution records of Hygophum hygomii [Dataset] (Available: Ocean Biodiversity Information System. Intergovernmental Oceanographic Commission of UNESCO. www.obis.org. Accessed: 2019-08-29)

Dataset details:

Dataset_ID	Name	Citation	License	OBIS_citation
09be818d-531c-4d86-8eba-5db81d42fdba	COLETA - IMAR/DOP-Uac reference collection from 1977 to 2012	Institute of Marine Research (IMAR - Azores), Portugal; Department of Oceanography and Fisheries (DOP) - UAC, Portugal (2015): COLETA - IMAR/DOP-Uac reference collection from 1977 to 2012. https://dx.doi.org/10.14284/23	This work is licensed under a Creative Commons Attribution (CC-BY) 4.0 License	OBIS (2019) [COLETA - IMAR/DOP-Uac reference collection from 1977 to 2012] (Available: Ocean Biodiversity Information System. Intergovernmental Oceanographic Commission of UNESCO. https://obis.org. Accessed: 2019-08-29)

0c9db499-759b-46d8-8989-799f9ff9f235	Auckland Museum NZ Marine Collection	Blom W, Moriarty A (2018). Auckland Museum NZ Marine Collection. Version 1.11. Auckland War Memorial Museum. Occurrence Dataset https://doi.org/10.15468/plyefd accessed via GBIF.org on 2018-01-15.	This work is licensed under a Creative Commons Attribution (CC-BY) 4.0 License	OBIS (2019) [Auckland Museum NZ Marine Collection] (Available: Ocean Biodiversity Information System. Intergovernmental Oceanographic Commission of UNESCO. https://obis.org. Accessed: 2019-08-29)
1f59030f-f116-4c34-915e-1882d819cda3	Institution Southampton Oceanography Ceter - Collection discovery	No available dataset citation	Unspecified intellectual rights	OBIS (2019) [Institution Southampton Oceanography Ceter - Collection discovery] (Available: Ocean Biodiversity Information System. Intergovernmental Oceanographic Commission of UNESCO. https://obis.org. Accessed: 2019-08-29)
270f3e70-ff9b-411d-b170-2bc914d83f26	Biological Reference Collections ICM CSIC	Olivas González F J (2016): Biological Reference Collections ICM CSIC. Institute of Marine Sciences (ICM-CSIC). https://dx.doi.org/10.15470/qlqqdx	This work is licensed under a Creative Commons Attribution Non Commercial (CC-BY-NC) 4.0 License	OBIS (2019) [Biological Reference Collections ICM CSIC] (Available: Ocean Biodiversity Information System. Intergovernmental Oceanographic Commission of UNESCO. https://obis.org. Accessed: 2019-08-29)
2870c548-343e-4575-ac67-a4da35182c52	Institution Shirshov Institute - Collection SKAO	No available dataset citation	Unspecified intellectual rights	OBIS (2019) [Institution Shirshov Institute - Collection SKAO] (Available: Ocean Biodiversity Information System. Intergovernmental Oceanographic Commission of UNESCO. https://obis.org. Accessed: 2019-08-29)
308a501c-a187-498e-a8bd-9cf3d2b70bd9	Institution REVIZEE - Collection Ictioplankton	No available dataset citation	Unspecified intellectual rights	OBIS (2019) [Institution REVIZEE - Collection Ictioplankton] (Available: Ocean Biodiversity Information System. Intergovernmental Oceanographic Commission of UNESCO. https://obis.org. Accessed: 2019-08-29)
3d922162-062c-4ad2-bf4a-f2493bd3a95d	Institution Bedford Institute of Oceanography (BIO) - Collection SUMMER	No available dataset citation	Unspecified intellectual rights	OBIS (2019) [Institution Bedford Institute of Oceanography (BIO) - Collection SUMMER] (Available: Ocean Biodiversity Information System. Intergovernmental Oceanographic Commission of UNESCO. https://obis.org. Accessed: 2019-08-29)

4bdc1f6f-e16a-48b4-b995-b51bd41caa8d	Dataset of the multidisciplinary research surveys in the seamounts of Ewing and Valdivia Bank (Walvis Ridge) - SE Atlantic	López-Abellán, L. J.; Sarralde Vizuete, R.; González Jiménez, J. F.; Centro Oceanográfico de Canarias – IEO, Spain (2015). Dataset of the multidisciplinary research surveys in the seamounts of Ewing and Valdivia Bank (Walvis Ridge) - SE Atlantic https://dx.doi.org/10.14284/58	Attribution-NoDerivatives (CC BY-ND)	OBIS (2019) [Dataset of the multidisciplinary research surveys in the seamounts of Ewing and Valdivia Bank (Walvis Ridge) - SE Atlantic] (Available: Ocean Biodiversity Information System. Intergovernmental Oceanographic Commission of UNESCO. https://obis.org. Accessed: 2019-08-29)
623f4f98-4e66-4bee-9e1c-17cb74cd2d21	Institution IEO - Collection MEDITS-Spain	No available dataset citation	Unspecified intellectual rights	OBIS (2019) [Institution IEO - Collection MEDITS-Spain] (Available: Ocean Biodiversity Information System. Intergovernmental Oceanographic Commission of UNESCO. https://obis.org. Accessed: 2019-08-29)
6a5bc28f-4dfe-4cbf-8a55-7e3a843997ab	SPC NECTALIS Zooplankton/Micronekton specimens, New Caledonia 2014	Allain, V., Menkes, C., 2014. Nectalis 3 cruise, RV Alis. https://doi.org/10.17600/14004900	This work is licensed under a Creative Commons Attribution Non Commercial (CC-BY-NC) 4.0 License	OBIS (2019) [SPC NECTALIS Zooplankton/Micronekton specimens, New Caledonia 2014] (Available: Ocean Biodiversity Information System. Intergovernmental Oceanographic Commission of UNESCO. https://obis.org. Accessed: 2019-08-29)
6c19184e-c305-4273-8890-6d342d86f865	Institution REVIZEE - Collection Pelagic Fishes	No available dataset citation	Unspecified intellectual rights	OBIS (2019) [Institution REVIZEE - Collection Pelagic Fishes] (Available: Ocean Biodiversity Information System. Intergovernmental Oceanographic Commission of UNESCO. https://obis.org. Accessed: 2019-08-29)
705770e5-3474-4e69-be8b-3107a0c5610a	The fishes collection (IC) of the Muséum national d'Histoire naturelle (MNHN - Paris)	Gicim data base, Pruvost P. Causse R., 2009 http://doi.org/10.15468/tm7whu	This work is licensed under a Creative Commons Attribution (CC-BY) 4.0 License	OBIS (2019) [The fishes collection (IC) of the Muséum national d'Histoire naturelle (MNHN - Paris)] (Available: Ocean Biodiversity Information System. Intergovernmental Oceanographic Commission of UNESCO. https://obis.org. Accessed: 2019-08-29)
8629ec33-be4b-4384-933f-a511fbc29967	MAR-ECO 2004	Wenneck, T. de Lange, Falkenhaug, T. and O.A. Bergstad. 2008. Strategies, methods, and technologies adopted on the RV G.O. Sars MAR-ECO expedition to the mid-Atlantic Ridge in 2004. Deep-sea Research II. 55: 6-28.	This work is licensed under a Creative Commons Attribution (CC-BY) 4.0 License	OBIS (2019) [MAR-ECO 2004] (Available: Ocean Biodiversity Information System. Intergovernmental Oceanographic Commission of UNESCO. https://obis.org. Accessed: 2019-08-29)

8a1ae661-e911-4967-bc06-1168fc5f2d89	iziko South African Museum - Fish Collection	iziko South African Museum - Fish Collection	Restricted	OBIS (2019) [iziko South African Museum - Fish Collection] (Available: Ocean Biodiversity Information System. Intergovernmental Oceanographic Commission of UNESCO. https://obis.org. Accessed: 2019-08-29)
9ff216fc-777e-4f9b-9860-95ed7366870d	Institution SAIAB - Collection SAIAB	No available dataset citation	Unspecified intellectual rights	OBIS (2019) [Institution SAIAB - Collection SAIAB] (Available: Ocean Biodiversity Information System. Intergovernmental Oceanographic Commission of UNESCO. https://obis.org. Accessed: 2019-08-29)
b8617377-eb1c-4db2-baa6-8788a632e810	Ichthyology Collection - Royal Ontario Museum	NA	This work is licensed under a Creative Commons Attribution Non Commercial (CC-BY-NC) 4.0 License	OBIS (2019) [Ichthyology Collection - Royal Ontario Museum] (Available: Ocean Biodiversity Information System. Intergovernmental Oceanographic Commission of UNESCO. https://obis.org. Accessed: 2019-08-29)
c24bf1c2-2c62-4056-a841-56d94e6e876a	Fish specimens	ROM Fish Collection (accessed through GBIF data portal, http://data.gbif.org/datasets/resource/660, 2012-01-20) http://doi.org/10.15468/syisbx	Unrestricted	OBIS (2019) [Fish specimens] (Available: Ocean Biodiversity Information System. Intergovernmental Oceanographic Commission of UNESCO. https://obis.org. Accessed: 2019-08-29)
cc8f28ce-e48d-4945-abfe-9d150a22dcd6	Hamburg pelagic fish database	Post, A. 1987. Pelagic transects of FRVs "Walther Herwig" and "Anton Dohrn" in the Atlantic Ocean 1966 to 1986. Mitt. Inst. f. Seefischerei d. BfaFi Hamburg, 42: 1-68.	This work is licensed under a Creative Commons Attribution (CC-BY) 4.0 License	OBIS (2019) [Hamburg pelagic fish database] (Available: Ocean Biodiversity Information System. Intergovernmental Oceanographic Commission of UNESCO. https://obis.org. Accessed: 2019-08-29)
ce1d93f3-8b0f-4ee7-9a4d-0393a6ec7fea	Atlantic Reference Centre Museum of Canadian Atlantic Organisms - Invertebrates and Fishes Data	Van Guelpen, L., 2016. Atlantic Reference Centre Museum of Canadian Atlantic Organisms - Invertebrates and Fishes Data. Version 4 In OBIS Canada Digital Collections. Bedford Institute of Oceanography, Dartmouth, NS, Canada. Published by OBIS, Digital http://www.iobis.org/. Accessed on –INSERT DATE	This work is licensed under a Creative Commons Attribution (CC-BY) 4.0 License	OBIS (2019) [Atlantic Reference Centre Museum of Canadian Atlantic Organisms - Invertebrates and Fishes Data] (Available: Ocean Biodiversity Information System. Intergovernmental Oceanographic Commission of UNESCO. https://obis.org. Accessed: 2019-08-29)

cfc56587-48c3-4e3d-9350-3a4d9a28b681	Institution NOAA, NMFS, Northeast Fisheries Science Center - Collection DEEPWATER SYSTEMATICS	No available dataset citation	Unspecified intellectual rights	OBIS (2019) [Institution NOAA, NMFS, Northeast Fisheries Science Center - Collection DEEPWATER SYSTEMATICS] (Available: Ocean Biodiversity Information System. Intergovernmental Oceanographic Commission of UNESCO. https://obis.org. Accessed: 2019-08-29)
d6d6fe4c-425f-4ce7-bf28-7a6befaeb413	National Museum of Natural History Vertebrate Zoology Fishes Collections	National Museum of Natural History, Smithsonian Institution NMNH Fishes Collection Database. National Museum of Natural History, Smithsonian Institution, 10th and Constitution Ave. N.W., Washington, DC 20560-0193, 2007.	This work is licensed under a Creative Commons Attribution (CC-BY) 4.0 License	OBIS (2019) [National Museum of Natural History Vertebrate Zoology Fishes Collections] (Available: Ocean Biodiversity Information System. Intergovernmental Oceanographic Commission of UNESCO. https://obis.org. Accessed: 2019-08-29)
e09c824c-cbd8-4529-b382-5306b2b3a875	Marine biodiversity atlas of the Balearic Sea	Deudero, Vallespir, Obrador 2011. Atlas de Biodiversidad Marina del Mar Balear. http://www.ba.ieo.es	This work is licensed under a Creative Commons Attribution Non Commercial (CC-BY-NC) 4.0 License	OBIS (2019) [Marine biodiversity atlas of the Balearic Sea] (Available: Ocean Biodiversity Information System. Intergovernmental Oceanographic Commission of UNESCO. https://obis.org. Accessed: 2019-08-29)

Diaphus fragilis

GBIF

GBIF Occurrence Download https://doi.org/10.15468/dd.2msggt Accessed from R via rgbif (https://github.com/ropensci/rgbif) on 2019-09-11

OBIS

OBIS (2019) Distribution records of Diaphus fragilis [Dataset] (Available: Ocean Biodiversity Information System. Intergovernmental Oceanographic Commission of UNESCO. www.obis.org. Accessed: 2019-08-29)

Dataset details:

Dataset_ID	Name	Citation	License	OBIS_citation

10b213e6-a9c4-459e-a40c-ef9edc461b97	Marine data from the Bernice P. Bishop Museum	Pyle R (2016). Bernice P. Bishop Museum. Version 8.1. Bernice Pauahi Bishop Museum. Occurrence dataset https://doi.org/10.15468/s6ctus accessed via GBIF.org on 2018-11-16.	To the extent possible under law, the publisher has waived all rights to these data and has dedicated them to the Public Domain (CC0 1.0)	OBIS (2019) [Marine data from the Bernice P. Bishop Museum] (Available: Ocean Biodiversity Information System. Intergovernmental Oceanographic Commission of UNESCO. https://obis.org. Accessed: 2019-08-29)
6c19184e-c305-4273-8890-6d342d86f865	Institution REVIZEE - Collection Pelagic Fishes	No available dataset citation	Unspecified intellectual rights	OBIS (2019) [Institution REVIZEE - Collection Pelagic Fishes] (Available: Ocean Biodiversity Information System. Intergovernmental Oceanographic Commission of UNESCO. https://obis.org. Accessed: 2019-08-29)
705770e5-3474-4e69-be8b-3107a0c5610a	The fishes collection (IC) of the Muséum national d'Histoire naturelle (MNHN - Paris)	Gicim data base, Pruvost P. Causse R., 2009 http://doi.org/10.15468/tm7whu	This work is licensed under a Creative Commons Attribution (CC-BY) 4.0 License	OBIS (2019) [The fishes collection (IC) of the Muséum national d'Histoire naturelle (MNHN - Paris)] (Available: Ocean Biodiversity Information System. Intergovernmental Oceanographic Commission of UNESCO. https://obis.org. Accessed: 2019-08-29)
b8617377-eb1c-4db2-baa6-8788a632e810	Ichthyology Collection - Royal Ontario Museum	NA	This work is licensed under a Creative Commons Attribution Non Commercial (CC-BY-NC) 4.0 License	OBIS (2019) [Ichthyology Collection - Royal Ontario Museum] (Available: Ocean Biodiversity Information System. Intergovernmental Oceanographic Commission of UNESCO. https://obis.org. Accessed: 2019-08-29)
c24bf1c2-2c62-4056-a841-56d94e6e876a	Fish specimens	ROM Fish Collection (accessed through GBIF data portal, http://data.gbif.org/datasets/resource/660, 2012-01-20) http://doi.org/10.15468/syisbx	Unrestricted	OBIS (2019) [Fish specimens] (Available: Ocean Biodiversity Information System. Intergovernmental Oceanographic Commission of UNESCO. https://obis.org. Accessed: 2019-08-29)
d6d6fe4c-425f-4ce7-bf28-7a6befaeb413	National Museum of Natural History Vertebrate Zoology Fishes Collections	National Museum of Natural History, Smithsonian Institution NMNH Fishes Collection Database. National Museum of Natural History, Smithsonian Institution, 10th and Constitution Ave. N.W., Washington, DC 20560-0193, 2007.	This work is licensed under a Creative Commons Attribution (CC-BY) 4.0 License	OBIS (2019) [National Museum of Natural History Vertebrate Zoology Fishes Collections] (Available: Ocean Biodiversity Information System. Intergovernmental Oceanographic Commission of UNESCO. https://obis.org. Accessed: 2019-08-29)

ff8b7809-41bc-40ad-8160-0e33862817a0	Biodiversity Research Museum, Academia Sinica, Taiwan	TELDAP, Biodiversity Research Museum, Academia Sinica, Taiwan (accessed through GBIF data portal, http://data.gbif.org/datasets/resource/9093, yyyy-mm-dd)	Unrestricted	OBIS (2019) [Biodiversity Research Museum, Academia Sinica, Taiwan] (Available: Ocean Biodiversity Information System. Intergovernmental Oceanographic Commission of UNESCO. https://obis.org. Accessed: 2019-08-29)

Lampadena luminosa

GBIF

GBIF Occurrence Download https://doi.org/10.15468/dd.uvb57s Accessed from R via rgbif (https://github.com/ropensci/rgbif) on 2019-09-11

OBIS

OBIS (2019) Distribution records of Lampadena luminosa [Dataset] (Available: Ocean Biodiversity Information System. Intergovernmental Oceanographic Commission of UNESCO. www.obis.org. Accessed: 2019-08-29)

Dataset details:

Dataset_ID	Name	Citation	License	OBIS_citation
04e3fd32-b08b-4806-a016-d2dff52ae55a	Asia-Pacific Dataset	Jintsu-Uchifune, Y., Yamamoto, H. (2016) Marine organism occurrence data of the Asia-Pacific region extracted from literature. Available at https://doi.org/10.48518/00002. Accessed on yyyy-mm-dd.	This work is licensed under a Creative Commons Attribution (CC-BY) 4.0 License	OBIS (2019) [Asia-Pacific Dataset] (Available: Ocean Biodiversity Information System. Intergovernmental Oceanographic Commission of UNESCO. https://obis.org. Accessed: 2019-08-29)
270f3e70-ff9b-411d-b170-2bc914d83f26	Biological Reference Collections ICM CSIC	Olivas González F J (2016): Biological Reference Collections ICM CSIC. Institute of Marine Sciences (ICM-CSIC). https://dx.doi.org/10.15470/qlqqdx	This work is licensed under a Creative Commons Attribution Non Commercial (CC-BY-NC) 4.0 License	OBIS (2019) [Biological Reference Collections ICM CSIC] (Available: Ocean Biodiversity Information System. Intergovernmental Oceanographic Commission of UNESCO. https://obis.org. Accessed: 2019-08-29)

2870c548-343e-4575-ac67-a4da35182c52	Institution Shirshov Institute - Collection SKAO	No available dataset citation	Unspecified intellectual rights	OBIS (2019) [Institution Shirshov Institute - Collection SKAO] (Available: Ocean Biodiversity Information System. Intergovernmental Oceanographic Commission of UNESCO. https://obis.org. Accessed: 2019-08-29)
705770e5-3474-4e69-be8b-3107a0c5610a	The fishes collection (IC) of the Muséum national d'Histoire naturelle (MNHN - Paris)	Gicim data base, Pruvost P. Causse R., 2009 http://doi.org/10.15468/tm7whu	This work is licensed under a Creative Commons Attribution (CC-BY) 4.0 License	OBIS (2019) [The fishes collection (IC) of the Muséum national d'Histoire naturelle (MNHN - Paris)] (Available: Ocean Biodiversity Information System. Intergovernmental Oceanographic Commission of UNESCO. https://obis.org. Accessed: 2019-08-29)
8a1ae661-e911-4967-bc06-1168fc5f2d89	iziko South African Museum - Fish Collection	iziko South African Museum - Fish Collection	Restricted	OBIS (2019) [iziko South African Museum - Fish Collection] (Available: Ocean Biodiversity Information System. Intergovernmental Oceanographic Commission of UNESCO. https://obis.org. Accessed: 2019-08-29)
9ff216fc-777e-4f9b-9860-95ed7366870d	Institution SAIAB - Collection SAIAB	No available dataset citation	Unspecified intellectual rights	OBIS (2019) [Institution SAIAB - Collection SAIAB] (Available: Ocean Biodiversity Information System. Intergovernmental Oceanographic Commission of UNESCO. https://obis.org. Accessed: 2019-08-29)
a4f7ee48-0d0b-4c05-a972-27a43b30db58	Institution MCM - Collection DEM	No available dataset citation	Unspecified intellectual rights	OBIS (2019) [Institution MCM - Collection DEM] (Available: Ocean Biodiversity Information System. Intergovernmental Oceanographic Commission of UNESCO. https://obis.org. Accessed: 2019-08-29)
cc8f28ce-e48d-4945-abfe-9d150a22dcd6	Hamburg pelagic fish database	Post, A. 1987. Pelagic transects of FRVs "Walther Herwig" and "Anton Dohrn" in the Atlantic Ocean 1966 to 1986. Mitt. Inst. f. Seefischerei d. BfaFi Hamburg, 42: 1-68.	This work is licensed under a Creative Commons Attribution (CC-BY) 4.0 License	OBIS (2019) [Hamburg pelagic fish database] (Available: Ocean Biodiversity Information System. Intergovernmental Oceanographic Commission of UNESCO. https://obis.org. Accessed: 2019-08-29)

ce1d93f3-8b0f-4ee7-9a4d-0393a6ec7fea	Atlantic Reference Centre Museum of Canadian Atlantic Organisms - Invertebrates and Fishes Data	Van Guelpen, L., 2016. Atlantic Reference Centre Museum of Canadian Atlantic Organisms - Invertebrates and Fishes Data. Version 4 In OBIS Canada Digital Collections. Bedford Institute of Oceanography, Dartmouth, NS, Canada. Published by OBIS, Digital http://www.iobis.org/. Accessed on –INSERT DATE	This work is licensed under a Creative Commons Attribution (CC-BY) 4.0 License	OBIS (2019) [Atlantic Reference Centre Museum of Canadian Atlantic Organisms - Invertebrates and Fishes Data] (Available: Ocean Biodiversity Information System. Intergovernmental Oceanographic Commission of UNESCO. https://obis.org. Accessed: 2019-08-29)
cfc56587-48c3-4e3d-9350-3a4d9a28b681	Institution NOAA, NMFS, Northeast Fisheries Science Center - Collection DEEPWATER SYSTEMATICS	No available dataset citation	Unspecified intellectual rights	OBIS (2019) [Institution NOAA, NMFS, Northeast Fisheries Science Center - Collection DEEPWATER SYSTEMATICS] (Available: Ocean Biodiversity Information System. Intergovernmental Oceanographic Commission of UNESCO. https://obis.org. Accessed: 2019-08-29)
d6d6fe4c-425f-4ce7-bf28-7a6befaeb413	National Museum of Natural History Vertebrate Zoology Fishes Collections	National Museum of Natural History, Smithsonian Institution NMNH Fishes Collection Database. National Museum of Natural History, Smithsonian Institution, 10th and Constitution Ave. N.W., Washington, DC 20560-0193, 2007.	This work is licensed under a Creative Commons Attribution (CC-BY) 4.0 License	OBIS (2019) [National Museum of Natural History Vertebrate Zoology Fishes Collections] (Available: Ocean Biodiversity Information System. Intergovernmental Oceanographic Commission of UNESCO. https://obis.org. Accessed: 2019-08-29)
ff8b7809-41bc-40ad-8160-0e33862817a0	Biodiversity Research Museum, Academia Sinica, Taiwan	TELDAP, Biodiversity Research Museum, Academia Sinica, Taiwan (accessed through GBIF data portal, http://data.gbif.org/datasets/resource/9093, yyyy-mm-dd)	Unrestricted	OBIS (2019) [Biodiversity Research Museum, Academia Sinica, Taiwan] (Available: Ocean Biodiversity Information System. Intergovernmental Oceanographic Commission of UNESCO. https://obis.org. Accessed: 2019-08-29)

Lampanyctus festivus

GBIF

GBIF Occurrence Download https://doi.org/10.15468/dd.f8cqf2 Accessed from R via rgbif (https://github.com/ropensci/rgbif) on 2019-09-11

OBIS

OBIS (2019) Distribution records of Lampanyctus festivus [Dataset] (Available: Ocean Biodiversity Information System. Intergovernmental Oceanographic Commission of UNESCO. www.obis.org. Accessed: 2019-08-29)

Dataset details:

Dataset_ID	Name	Citation	License	OBIS_citation
0c9db499-759b-46d8-8989-799f9ff9f235	Auckland Museum NZ Marine Collection	Blom W, Moriarty A (2018). Auckland Museum NZ Marine Collection. Version 1.11. Auckland War Memorial Museum. Occurrence Dataset https://doi.org/10.15468/plyefd accessed via GBIF.org on 2018-01-15.	This work is licensed under a Creative Commons Attribution (CC-BY) 4.0 License	OBIS (2019) [Auckland Museum NZ Marine Collection] (Available: Ocean Biodiversity Information System. Intergovernmental Oceanographic Commission of UNESCO. https://obis.org. Accessed: 2019-08-29)
1f59030f-f116-4c34-915e-1882d819cda3	Institution Southampton Oceanography Ceter - Collection discovery	No available dataset citation	Unspecified intellectual rights	OBIS (2019) [Institution Southampton Oceanography Ceter - Collection discovery] (Available: Ocean Biodiversity Information System. Intergovernmental Oceanographic Commission of UNESCO. https://obis.org. Accessed: 2019-08-29)
6a5bc28f-4dfe-4cbf-8a55-7e3a843997ab	SPC NECTALIS Zooplankton/Micronekton specimens, New Caledonia 2014	Allain, V., Menkes, C., 2014. Nectalis 3 cruise, RV Alis. https://doi.org/10.17600/14004900	This work is licensed under a Creative Commons Attribution Non Commercial (CC-BY-NC) 4.0 License	OBIS (2019) [SPC NECTALIS Zooplankton/Micronekton specimens, New Caledonia 2014] (Available: Ocean Biodiversity Information System. Intergovernmental Oceanographic Commission of UNESCO. https://obis.org. Accessed: 2019-08-29)
705770e5-3474-4e69-be8b-3107a0c5610a	The fishes collection (IC) of the Muséum national d'Histoire naturelle (MNHN - Paris)	Gicim data base, Pruvost P. Causse R., 2009 http://doi.org/10.15468/tm7whu	This work is licensed under a Creative Commons Attribution (CC-BY) 4.0 License	OBIS (2019) [The fishes collection (IC) of the Muséum national d'Histoire naturelle (MNHN - Paris)] (Available: Ocean Biodiversity Information System. Intergovernmental Oceanographic Commission of UNESCO. https://obis.org. Accessed: 2019-08-29)
8629ec33-be4b-4384-933f-a511fbc29967	MAR-ECO 2004	Wenneck, T. de Lange, Falkenhaug, T. and O.A. Bergstad. 2008. Strategies, methods, and technologies adopted on the RV G.O. Sars MAR-ECO expedition to the mid-Atlantic Ridge in 2004. Deep-sea Research II. 55: 6-28.	This work is licensed under a Creative Commons Attribution (CC-BY) 4.0 License	OBIS (2019) [MAR-ECO 2004] (Available: Ocean Biodiversity Information System. Intergovernmental Oceanographic Commission of UNESCO. https://obis.org. Accessed: 2019-08-29)

8a1ae661-e911-4967-bc06-1168fc5f2d89	iziko South African Museum - Fish Collection	iziko South African Museum - Fish Collection	Restricted	OBIS (2019) [iziko South African Museum - Fish Collection] (Available: Ocean Biodiversity Information System. Intergovernmental Oceanographic Commission of UNESCO. https://obis.org. Accessed: 2019-08-29)
9ff216fc-777e-4f9b-9860-95ed7366870d	Institution SAIAB - Collection SAIAB	No available dataset citation	Unspecified intellectual rights	OBIS (2019) [Institution SAIAB - Collection SAIAB] (Available: Ocean Biodiversity Information System. Intergovernmental Oceanographic Commission of UNESCO. https://obis.org. Accessed: 2019-08-29)
cc8f28ce-e48d-4945-abfe-9d150a22dcd6	Hamburg pelagic fish database	Post, A. 1987. Pelagic transects of FRVs "Walther Herwig" and "Anton Dohrn" in the Atlantic Ocean 1966 to 1986. Mitt. Inst. f. Seefischerei d. BfaFi Hamburg, 42: 1-68.	This work is licensed under a Creative Commons Attribution (CC-BY) 4.0 License	OBIS (2019) [Hamburg pelagic fish database] (Available: Ocean Biodiversity Information System. Intergovernmental Oceanographic Commission of UNESCO. https://obis.org. Accessed: 2019-08-29)
ce1d93f3-8b0f-4ee7-9a4d-0393a6ec7fea	Atlantic Reference Centre Museum of Canadian Atlantic Organisms - Invertebrates and Fishes Data	Van Guelpen, L., 2016. Atlantic Reference Centre Museum of Canadian Atlantic Organisms - Invertebrates and Fishes Data. Version 4 In OBIS Canada Digital Collections. Bedford Institute of Oceanography, Dartmouth, NS, Canada. Published by OBIS, Digital http://www.iobis.org/. Accessed on –INSERT DATE	This work is licensed under a Creative Commons Attribution (CC-BY) 4.0 License	OBIS (2019) [Atlantic Reference Centre Museum of Canadian Atlantic Organisms - Invertebrates and Fishes Data] (Available: Ocean Biodiversity Information System. Intergovernmental Oceanographic Commission of UNESCO. https://obis.org. Accessed: 2019-08-29)
d6d6fe4c-425f-4ce7-bf28-7a6befaeb413	National Museum of Natural History Vertebrate Zoology Fishes Collections	National Museum of Natural History, Smithsonian Institution NMNH Fishes Collection Database. National Museum of Natural History, Smithsonian Institution, 10th and Constitution Ave. N.W., Washington, DC 20560-0193, 2007.	This work is licensed under a Creative Commons Attribution (CC-BY) 4.0 License	OBIS (2019) [National Museum of Natural History Vertebrate Zoology Fishes Collections] (Available: Ocean Biodiversity Information System. Intergovernmental Oceanographic Commission of UNESCO. https://obis.org. Accessed: 2019-08-29)
ff8b7809-41bc-40ad-8160-0e33862817a0	Biodiversity Research Museum, Academia Sinica, Taiwan	TELDAP, Biodiversity Research Museum, Academia Sinica, Taiwan (accessed through GBIF data portal, http://data.gbif.org/datasets/resource/9093, yyyy-mm-dd)	Unrestricted	OBIS (2019) [Biodiversity Research Museum, Academia Sinica, Taiwan] (Available: Ocean Biodiversity Information System. Intergovernmental Oceanographic Commission of UNESCO. https://obis.org. Accessed: 2019-08-29)

Diaphus brachycephalus

GBIF

GBIF Occurrence Download https://doi.org/10.15468/dd.vcnpme Accessed from R via rgbif (https://github.com/ropensci/rgbif) on 2019-09-11

OBIS

OBIS (2019) Distribution records of Diaphus brachycephalus [Dataset] (Available: Ocean Biodiversity Information System. Intergovernmental Oceanographic Commission of UNESCO. www.obis.org. Accessed: 2019-08-29)

Dataset details:

Dataset_ID	Name	Citation	License	OBIS_citation
10b213e6-a9c4-459e-a40c-ef9edc461b97	Marine data from the Bernice P. Bishop Museum	Pyle R (2016). Bernice P. Bishop Museum. Version 8.1. Bernice Pauahi Bishop Museum. Occurrence dataset https://doi.org/10.15468/s6ctus accessed via GBIF.org on 2018-11-16.	To the extent possible under law, the publisher has waived all rights to these data and has dedicated them to the Public Domain (CC0 1.0)	OBIS (2019) [Marine data from the Bernice P. Bishop Museum] (Available: Ocean Biodiversity Information System. Intergovernmental Oceanographic Commission of UNESCO. https://obis.org. Accessed: 2019-08-29)
270f3e70-ff9b-411d-b170-2bc914d83f26	Biological Reference Collections ICM CSIC	Olivas González F J (2016): Biological Reference Collections ICM CSIC. Institute of Marine Sciences (ICM-CSIC). https://dx.doi.org/10.15470/qlqqdx	This work is licensed under a Creative Commons Attribution Non Commercial (CC-BY-NC) 4.0 License	OBIS (2019) [Biological Reference Collections ICM CSIC] (Available: Ocean Biodiversity Information System. Intergovernmental Oceanographic Commission of UNESCO. https://obis.org. Accessed: 2019-08-29)
2870c548-343e-4575-ac67-a4da35182c52	Institution Shirshov Institute - Collection SKAO	No available dataset citation	Unspecified intellectual rights	OBIS (2019) [Institution Shirshov Institute - Collection SKAO] (Available: Ocean Biodiversity Information System. Intergovernmental Oceanographic Commission of UNESCO. https://obis.org. Accessed: 2019-08-29)

6a5bc28f-4dfe-4cbf-8a55-7e3a843997ab	SPC NECTALIS Zooplankton/Micronekton specimens, New Caledonia 2014	Allain, V., Menkes, C., 2014. Nectalis 3 cruise, RV Alis. https://doi.org/10.17600/14004900	This work is licensed under a Creative Commons Attribution Non Commercial (CC-BY-NC) 4.0 License	OBIS (2019) [SPC NECTALIS Zooplankton/Micronekton specimens, New Caledonia 2014] (Available: Ocean Biodiversity Information System. Intergovernmental Oceanographic Commission of UNESCO. https://obis.org. Accessed: 2019-08-29)
705770e5-3474-4e69-be8b-3107a0c5610a	The fishes collection (IC) of the Muséum national d'Histoire naturelle (MNHN - Paris)	Gicim data base, Pruvost P. Causse R., 2009 http://doi.org/10.15468/tm7whu	This work is licensed under a Creative Commons Attribution (CC-BY) 4.0 License	OBIS (2019) [The fishes collection (IC) of the Muséum national d'Histoire naturelle (MNHN - Paris)] (Available: Ocean Biodiversity Information System. Intergovernmental Oceanographic Commission of UNESCO. https://obis.org. Accessed: 2019-08-29)
8a1ae661-e911-4967-bc06-1168fc5f2d89	iziko South African Museum - Fish Collection	iziko South African Museum - Fish Collection	Restricted	OBIS (2019) [iziko South African Museum - Fish Collection] (Available: Ocean Biodiversity Information System. Intergovernmental Oceanographic Commission of UNESCO. https://obis.org. Accessed: 2019-08-29)
b8617377-eb1c-4db2-baa6-8788a632e810	Ichthyology Collection - Royal Ontario Museum	NA	This work is licensed under a Creative Commons Attribution Non Commercial (CC-BY-NC) 4.0 License	OBIS (2019) [Ichthyology Collection - Royal Ontario Museum] (Available: Ocean Biodiversity Information System. Intergovernmental Oceanographic Commission of UNESCO. https://obis.org. Accessed: 2019-08-29)
c24bf1c2-2c62-4056-a841-56d94e6e876a	Fish specimens	ROM Fish Collection (accessed through GBIF data portal, http://data.gbif.org/datasets/resource/660, 2012-01-20) http://doi.org/10.15468/syisbx	Unrestricted	OBIS (2019) [Fish specimens] (Available: Ocean Biodiversity Information System. Intergovernmental Oceanographic Commission of UNESCO. https://obis.org. Accessed: 2019-08-29)
cc8f28ce-e48d-4945-abfe-9d150a22dcd6	Hamburg pelagic fish database	Post, A. 1987. Pelagic transects of FRVs "Walther Herwig" and "Anton Dohrn" in the Atlantic Ocean 1966 to 1986. Mitt. Inst. f. Seefischerei d. BfaFi Hamburg, 42: 1-68.	This work is licensed under a Creative Commons Attribution (CC-BY) 4.0 License	OBIS (2019) [Hamburg pelagic fish database] (Available: Ocean Biodiversity Information System. Intergovernmental Oceanographic Commission of UNESCO. https://obis.org. Accessed: 2019-08-29)

ce1d93f3-8b0f-4ee7-9a4d-0393a6ec7fea	Atlantic Reference Centre Museum of Canadian Atlantic Organisms - Invertebrates and Fishes Data	Van Guelpen, L., 2016. Atlantic Reference Centre Museum of Canadian Atlantic Organisms - Invertebrates and Fishes Data. Version 4 In OBIS Canada Digital Collections. Bedford Institute of Oceanography, Dartmouth, NS, Canada. Published by OBIS, Digital http://www.iobis.org/. Accessed on –INSERT DATE	This work is licensed under a Creative Commons Attribution (CC-BY) 4.0 License	OBIS (2019) [Atlantic Reference Centre Museum of Canadian Atlantic Organisms - Invertebrates and Fishes Data] (Available: Ocean Biodiversity Information System. Intergovernmental Oceanographic Commission of UNESCO. https://obis.org. Accessed: 2019-08-29)
d6d6fe4c-425f-4ce7-bf28-7a6befaeb413	National Museum of Natural History Vertebrate Zoology Fishes Collections	National Museum of Natural History, Smithsonian Institution NMNH Fishes Collection Database. National Museum of Natural History, Smithsonian Institution, 10th and Constitution Ave. N.W., Washington, DC 20560-0193, 2007.	This work is licensed under a Creative Commons Attribution (CC-BY) 4.0 License	OBIS (2019) [National Museum of Natural History Vertebrate Zoology Fishes Collections] (Available: Ocean Biodiversity Information System. Intergovernmental Oceanographic Commission of UNESCO. https://obis.org. Accessed: 2019-08-29)
ff8b7809-41bc-40ad-8160-0e33862817a0	Biodiversity Research Museum, Academia Sinica, Taiwan	TELDAP, Biodiversity Research Museum, Academia Sinica, Taiwan (accessed through GBIF data portal, http://data.gbif.org/datasets/resource/9093, yyyy-mm-dd)	Unrestricted	OBIS (2019) [Biodiversity Research Museum, Academia Sinica, Taiwan] (Available: Ocean Biodiversity Information System. Intergovernmental Oceanographic Commission of UNESCO. https://obis.org. Accessed: 2019-08-29)

Lampadena chavesi

GBIF

GBIF Occurrence Download https://doi.org/10.15468/dd.8388ep Accessed from R via rgbif (https://github.com/ropensci/rgbif) on 2019-09-11

OBIS

OBIS (2019) Distribution records of Lampadena chavesi [Dataset] (Available: Ocean Biodiversity Information System. Intergovernmental Oceanographic Commission of UNESCO. www.obis.org. Accessed: 2019-08-29)

Dataset details:

Dataset_ID	Name	Citation	License	OBIS_citation

1f59030f-f116-4c34-915e-1882d819cda3	Institution Southampton Oceanography Ceter - Collection discovery	No available dataset citation	Unspecified intellectual rights	OBIS (2019) [Institution Southampton Oceanography Ceter - Collection discovery] (Available: Ocean Biodiversity Information System. Intergovernmental Oceanographic Commission of UNESCO. https://obis.org. Accessed: 2019-08-29)
2870c548-343e-4575-ac67-a4da35182c52	Institution Shirshov Institute - Collection SKAO	No available dataset citation	Unspecified intellectual rights	OBIS (2019) [Institution Shirshov Institute - Collection SKAO] (Available: Ocean Biodiversity Information System. Intergovernmental Oceanographic Commission of UNESCO. https://obis.org. Accessed: 2019-08-29)
8629ec33-be4b-4384-933f-a511fbc29967	MAR-ECO 2004	Wenneck, T. de Lange, Falkenhaug, T. and O.A. Bergstad. 2008. Strategies, methods, and technologies adopted on the RV G.O. Sars MAR-ECO expedition to the mid-Atlantic Ridge in 2004. Deep-sea Research II. 55: 6-28.	This work is licensed under a Creative Commons Attribution (CC-BY) 4.0 License	OBIS (2019) [MAR-ECO 2004] (Available: Ocean Biodiversity Information System. Intergovernmental Oceanographic Commission of UNESCO. https://obis.org. Accessed: 2019-08-29)
8a1ae661-e911-4967-bc06-1168fc5f2d89	iziko South African Museum - Fish Collection	iziko South African Museum - Fish Collection	Restricted	OBIS (2019) [iziko South African Museum - Fish Collection] (Available: Ocean Biodiversity Information System. Intergovernmental Oceanographic Commission of UNESCO. https://obis.org. Accessed: 2019-08-29)
b8617377-eb1c-4db2-baa6-8788a632e810	Ichthyology Collection - Royal Ontario Museum	NA	This work is licensed under a Creative Commons Attribution Non Commercial (CC-BY-NC) 4.0 License	OBIS (2019) [Ichthyology Collection - Royal Ontario Museum] (Available: Ocean Biodiversity Information System. Intergovernmental Oceanographic Commission of UNESCO. https://obis.org. Accessed: 2019-08-29)
c24bf1c2-2c62-4056-a841-56d94e6e876a	Fish specimens	ROM Fish Collection (accessed through GBIF data portal, http://data.gbif.org/datasets/resource/660, 2012-01-20) http://doi.org/10.15468/syisbx	Unrestricted	OBIS (2019) [Fish specimens] (Available: Ocean Biodiversity Information System. Intergovernmental Oceanographic Commission of UNESCO. https://obis.org. Accessed: 2019-08-29)

cc8f28ce-e48d-4945-abfe-9d150a22dcd6	Hamburg pelagic fish database	Post, A. 1987. Pelagic transects of FRVs "Walther Herwig" and "Anton Dohrn" in the Atlantic Ocean 1966 to 1986. Mitt. Inst. f. Seefischerei d. BfaFi Hamburg, 42: 1-68.	This work is licensed under a Creative Commons Attribution (CC-BY) 4.0 License	OBIS (2019) [Hamburg pelagic fish database] (Available: Ocean Biodiversity Information System. Intergovernmental Oceanographic Commission of UNESCO. https://obis.org. Accessed: 2019-08-29)
ce1d93f3-8b0f-4ee7-9a4d-0393a6ec7fea	Atlantic Reference Centre Museum of Canadian Atlantic Organisms - Invertebrates and Fishes Data	Van Guelpen, L., 2016. Atlantic Reference Centre Museum of Canadian Atlantic Organisms - Invertebrates and Fishes Data. Version 4 In OBIS Canada Digital Collections. Bedford Institute of Oceanography, Dartmouth, NS, Canada. Published by OBIS, Digital http://www.iobis.org/. Accessed on –INSERT DATE	This work is licensed under a Creative Commons Attribution (CC-BY) 4.0 License	OBIS (2019) [Atlantic Reference Centre Museum of Canadian Atlantic Organisms - Invertebrates and Fishes Data] (Available: Ocean Biodiversity Information System. Intergovernmental Oceanographic Commission of UNESCO. https://obis.org. Accessed: 2019-08-29)
d6d6fe4c-425f-4ce7-bf28-7a6befaeb413	National Museum of Natural History Vertebrate Zoology Fishes Collections	National Museum of Natural History, Smithsonian Institution NMNH Fishes Collection Database. National Museum of Natural History, Smithsonian Institution, 10th and Constitution Ave. N.W., Washington, DC 20560-0193, 2007.	This work is licensed under a Creative Commons Attribution (CC-BY) 4.0 License	OBIS (2019) [National Museum of Natural History Vertebrate Zoology Fishes Collections] (Available: Ocean Biodiversity Information System. Intergovernmental Oceanographic Commission of UNESCO. https://obis.org. Accessed: 2019-08-29)

Notolychnus valdiviae

GBIF

GBIF Occurrence Download https://doi.org/10.15468/dd.tz5b3y Accessed from R via rgbif (https://github.com/ropensci/rgbif) on 2019-09-11

OBIS

OBIS (2019) Distribution records of Notolychnus valdiviae [Dataset] (Available: Ocean Biodiversity Information System. Intergovernmental Oceanographic Commission of UNESCO. www.obis.org. Accessed: 2019-08-29)

Dataset details:

Dataset_ID	Name	Citation	License	OBIS_citation

04e3fd32-b08b-4806-a016-d2dff52ae55a	Asia-Pacific Dataset	Jintsu-Uchifune, Y., Yamamoto, H. (2016) Marine organism occurrence data of the Asia-Pacific region extracted from literature. Available at https://doi.org/10.48518/00002. Accessed on yyyy-mm-dd.	This work is licensed under a Creative Commons Attribution (CC-BY) 4.0 License	OBIS (2019) [Asia-Pacific Dataset] (Available: Ocean Biodiversity Information System. Intergovernmental Oceanographic Commission of UNESCO. https://obis.org. Accessed: 2019-08-29)
09be818d-531c-4d86-8eba-5db81d42fdba	COLETA - IMAR/DOP-Uac reference collection from 1977 to 2012	Institute of Marine Research (IMAR - Azores), Portugal; Department of Oceanography and Fisheries (DOP) - UAC, Portugal (2015): COLETA - IMAR/DOP-Uac reference collection from 1977 to 2012. https://dx.doi.org/10.14284/23	This work is licensed under a Creative Commons Attribution (CC-BY) 4.0 License	OBIS (2019) [COLETA - IMAR/DOP-Uac reference collection from 1977 to 2012] (Available: Ocean Biodiversity Information System. Intergovernmental Oceanographic Commission of UNESCO. https://obis.org. Accessed: 2019-08-29)
1f59030f-f116-4c34-915e-1882d819cda3	Institution Southampton Oceanography Ceter - Collection discovery	No available dataset citation	Unspecified intellectual rights	OBIS (2019) [Institution Southampton Oceanography Ceter - Collection discovery] (Available: Ocean Biodiversity Information System. Intergovernmental Oceanographic Commission of UNESCO. https://obis.org. Accessed: 2019-08-29)
2870c548-343e-4575-ac67-a4da35182c52	Institution Shirshov Institute - Collection SKAO	No available dataset citation	Unspecified intellectual rights	OBIS (2019) [Institution Shirshov Institute - Collection SKAO] (Available: Ocean Biodiversity Information System. Intergovernmental Oceanographic Commission of UNESCO. https://obis.org. Accessed: 2019-08-29)
308a501c-a187-498e-a8bd-9cf3d2b70bd9	Institution REVIZEE - Collection Ictioplankton	No available dataset citation	Unspecified intellectual rights	OBIS (2019) [Institution REVIZEE - Collection Ictioplankton] (Available: Ocean Biodiversity Information System. Intergovernmental Oceanographic Commission of UNESCO. https://obis.org. Accessed: 2019-08-29)
4e25e0ce-b17d-4192-9b55-417f1e0c4fc8	Institution KU - Collection KUI	No available dataset citation	Unspecified intellectual rights	OBIS (2019) [Institution KU - Collection KUI] (Available: Ocean Biodiversity Information System. Intergovernmental Oceanographic Commission of UNESCO. https://obis.org. Accessed: 2019-08-29)

5f2da252-6d49-4c9f-b3b3-1db53d75b345	MARMAP Bongo Nets 1990-2009	Marcel Reichert, 2010, MARMAP Bongo Nets 1990-2009, SCDNR/NOAA MARMAP Program, SCDNR MARMAP Aggregate data surveys, The Marine Resources Monitoring, Assessment, and Prediction (MARMAP) Program, Marine Resources Research Institute, South Carolina Department of Natural Resources, P. O. Box 12559, Charleston SC 29422-2559, U.S.A.Retrieved from http://www.usgs.gov/obis-usa/	Restricted	OBIS (2019) [MARMAP Bongo Nets 1990-2009] (Available: Ocean Biodiversity Information System. Intergovernmental Oceanographic Commission of UNESCO. https://obis.org. Accessed: 2019-08-29)
705770e5-3474-4e69-be8b-3107a0c5610a	The fishes collection (IC) of the Muséum national d'Histoire naturelle (MNHN - Paris)	Gicim data base, Pruvost P. Causse R., 2009 http://doi.org/10.15468/tm7whu	This work is licensed under a Creative Commons Attribution (CC-BY) 4.0 License	OBIS (2019) [The fishes collection (IC) of the Muséum national d'Histoire naturelle (MNHN - Paris)] (Available: Ocean Biodiversity Information System. Intergovernmental Oceanographic Commission of UNESCO. https://obis.org. Accessed: 2019-08-29)
7e4228e5-a962-4b01-952f-7bf33e213a9c	BioChem: Sameoto zooplankton collection	Sameoto, D.D., Kennedy, M., Spry, J.S, Spry, J.M. (2013). Zooplankton datasets collected using the BIONESS sampler, ring nets and an Icelandic high speed sampler, 1967-2006. OBIS Canada Digital Collections. Published by OBIS http://www.iobis.org/. Accessed on –INSERT DATE	rights: http://data.gc.ca/eng/open-government-licence-canada & http://www.canadensys.net/norms rights holder: Her Majesty the Queen in right of Canada, as represented by the Minister of Fisheries and Oceans	OBIS (2019) [BioChem: Sameoto zooplankton collection] (Available: Ocean Biodiversity Information System. Intergovernmental Oceanographic Commission of UNESCO. https://obis.org. Accessed: 2019-08-29)
8629ec33-be4b-4384-933f-a511fbc29967	MAR-ECO 2004	Wenneck, T. de Lange, Falkenhaug, T. and O.A. Bergstad. 2008. Strategies, methods, and technologies adopted on the RV G.O. Sars MAR-ECO expedition to the mid-Atlantic Ridge in 2004. Deep-sea Research II. 55: 6-28.	This work is licensed under a Creative Commons Attribution (CC-BY) 4.0 License	OBIS (2019) [MAR-ECO 2004] (Available: Ocean Biodiversity Information System. Intergovernmental Oceanographic Commission of UNESCO. https://obis.org. Accessed: 2019-08-29)

8a1ae661-e911-4967-bc06-1168fc5f2d89	iziko South African Museum - Fish Collection	iziko South African Museum - Fish Collection	Restricted	OBIS (2019) [iziko South African Museum - Fish Collection] (Available: Ocean Biodiversity Information System. Intergovernmental Oceanographic Commission of UNESCO. https://obis.org. Accessed: 2019-08-29)
b8617377-eb1c-4db2-baa6-8788a632e810	Ichthyology Collection - Royal Ontario Museum	NA	This work is licensed under a Creative Commons Attribution Non Commercial (CC-BY-NC) 4.0 License	OBIS (2019) [Ichthyology Collection - Royal Ontario Museum] (Available: Ocean Biodiversity Information System. Intergovernmental Oceanographic Commission of UNESCO. https://obis.org. Accessed: 2019-08-29)
c24bf1c2-2c62-4056-a841-56d94e6e876a	Fish specimens	ROM Fish Collection (accessed through GBIF data portal, http://data.gbif.org/datasets/resource/660, 2012-01-20) http://doi.org/10.15468/syisbx	Unrestricted	OBIS (2019) [Fish specimens] (Available: Ocean Biodiversity Information System. Intergovernmental Oceanographic Commission of UNESCO. https://obis.org. Accessed: 2019-08-29)
cc8f28ce-e48d-4945-abfe-9d150a22dcd6	Hamburg pelagic fish database	Post, A. 1987. Pelagic transects of FRVs "Walther Herwig" and "Anton Dohrn" in the Atlantic Ocean 1966 to 1986. Mitt. Inst. f. Seefischerei d. BfaFi Hamburg, 42: 1-68.	This work is licensed under a Creative Commons Attribution (CC-BY) 4.0 License	OBIS (2019) [Hamburg pelagic fish database] (Available: Ocean Biodiversity Information System. Intergovernmental Oceanographic Commission of UNESCO. https://obis.org. Accessed: 2019-08-29)
ce1d93f3-8b0f-4ee7-9a4d-0393a6ec7fea	Atlantic Reference Centre Museum of Canadian Atlantic Organisms - Invertebrates and Fishes Data	Van Guelpen, L., 2016. Atlantic Reference Centre Museum of Canadian Atlantic Organisms - Invertebrates and Fishes Data. Version 4 In OBIS Canada Digital Collections. Bedford Institute of Oceanography, Dartmouth, NS, Canada. Published by OBIS, Digital http://www.iobis.org/. Accessed on –INSERT DATE	This work is licensed under a Creative Commons Attribution (CC-BY) 4.0 License	OBIS (2019) [Atlantic Reference Centre Museum of Canadian Atlantic Organisms - Invertebrates and Fishes Data] (Available: Ocean Biodiversity Information System. Intergovernmental Oceanographic Commission of UNESCO. https://obis.org. Accessed: 2019-08-29)
d6d6fe4c-425f-4ce7-bf28-7a6befaeb413	National Museum of Natural History Vertebrate Zoology Fishes Collections	National Museum of Natural History, Smithsonian Institution NMNH Fishes Collection Database. National Museum of Natural History, Smithsonian Institution, 10th and Constitution Ave. N.W., Washington, DC 20560-0193, 2007.	This work is licensed under a Creative Commons Attribution (CC-BY) 4.0 License	OBIS (2019) [National Museum of Natural History Vertebrate Zoology Fishes Collections] (Available: Ocean Biodiversity Information System. Intergovernmental Oceanographic Commission of UNESCO. https://obis.org. Accessed: 2019-08-29)

Notoscopelus caudispinosus

GBIF

GBIF Occurrence Download https://doi.org/10.15468/dd.feq999 Accessed from R via rgbif (https://github.com/ropensci/rgbif) on 2019-09-11

OBIS

OBIS (2019) Distribution records of Notoscopelus caudispinosus [Dataset] (Available: Ocean Biodiversity Information System. Intergovernmental Oceanographic Commission of UNESCO. www.obis.org. Accessed: 2019-08-29)

Dataset details:

Dataset_ID	Name	Citation	License	OBIS_citation
10b213e6-a9c4-459e-a40c-ef9edc461b97	Marine data from the Bernice P. Bishop Museum	Pyle R (2016). Bernice P. Bishop Museum. Version 8.1. Bernice Pauahi Bishop Museum. Occurrence dataset https://doi.org/10.15468/s6ctus accessed via GBIF.org on 2018-11-16.	To the extent possible under law, the publisher has waived all rights to these data and has dedicated them to the Public Domain (CC0 1.0)	OBIS (2019) [Marine data from the Bernice P. Bishop Museum] (Available: Ocean Biodiversity Information System. Intergovernmental Oceanographic Commission of UNESCO. https://obis.org. Accessed: 2019-08-29)
1f59030f-f116-4c34-915e-1882d819cda3	Institution Southampton Oceanography Ceter - Collection discovery	No available dataset citation	Unspecified intellectual rights	OBIS (2019) [Institution Southampton Oceanography Ceter - Collection discovery] (Available: Ocean Biodiversity Information System. Intergovernmental Oceanographic Commission of UNESCO. https://obis.org. Accessed: 2019-08-29)
2870c548-343e-4575-ac67-a4da35182c52	Institution Shirshov Institute - Collection SKAO	No available dataset citation	Unspecified intellectual rights	OBIS (2019) [Institution Shirshov Institute - Collection SKAO] (Available: Ocean Biodiversity Information System. Intergovernmental Oceanographic Commission of UNESCO. https://obis.org. Accessed: 2019-08-29)
3d922162-062c-4ad2-bf4a-f2493bd3a95d	Institution Bedford Institute of Oceanography (BIO) - Collection SUMMER	No available dataset citation	Unspecified intellectual rights	OBIS (2019) [Institution Bedford Institute of Oceanography (BIO) - Collection SUMMER] (Available: Ocean Biodiversity Information System. Intergovernmental Oceanographic Commission of UNESCO. https://obis.org. Accessed: 2019-08-29)

6c19184e-c305-4273-8890-6d342d86f865	Institution REVIZEE - Collection Pelagic Fishes	No available dataset citation	Unspecified intellectual rights	OBIS (2019) [Institution REVIZEE - Collection Pelagic Fishes] (Available: Ocean Biodiversity Information System. Intergovernmental Oceanographic Commission of UNESCO. https://obis.org. Accessed: 2019-08-29)
8a1ae661-e911-4967-bc06-1168fc5f2d89	iziko South African Museum - Fish Collection	iziko South African Museum - Fish Collection	Restricted	OBIS (2019) [iziko South African Museum - Fish Collection] (Available: Ocean Biodiversity Information System. Intergovernmental Oceanographic Commission of UNESCO. https://obis.org. Accessed: 2019-08-29)
cc8f28ce-e48d-4945-abfe-9d150a22dcd6	Hamburg pelagic fish database	Post, A. 1987. Pelagic transects of FRVs "Walther Herwig" and "Anton Dohrn" in the Atlantic Ocean 1966 to 1986. Mitt. Inst. f. Seefischerei d. BfaFi Hamburg, 42: 1-68.	This work is licensed under a Creative Commons Attribution (CC-BY) 4.0 License	OBIS (2019) [Hamburg pelagic fish database] (Available: Ocean Biodiversity Information System. Intergovernmental Oceanographic Commission of UNESCO. https://obis.org. Accessed: 2019-08-29)
ce1d93f3-8b0f-4ee7-9a4d-0393a6ec7fea	Atlantic Reference Centre Museum of Canadian Atlantic Organisms - Invertebrates and Fishes Data	Van Guelpen, L., 2016. Atlantic Reference Centre Museum of Canadian Atlantic Organisms - Invertebrates and Fishes Data. Version 4 In OBIS Canada Digital Collections. Bedford Institute of Oceanography, Dartmouth, NS, Canada. Published by OBIS, Digital http://www.iobis.org/. Accessed on –INSERT DATE	This work is licensed under a Creative Commons Attribution (CC-BY) 4.0 License	OBIS (2019) [Atlantic Reference Centre Museum of Canadian Atlantic Organisms - Invertebrates and Fishes Data] (Available: Ocean Biodiversity Information System. Intergovernmental Oceanographic Commission of UNESCO. https://obis.org. Accessed: 2019-08-29)
cfc56587-48c3-4e3d-9350-3a4d9a28b681	Institution NOAA, NMFS, Northeast Fisheries Science Center - Collection DEEPWATER SYSTEMATICS	No available dataset citation	Unspecified intellectual rights	OBIS (2019) [Institution NOAA, NMFS, Northeast Fisheries Science Center - Collection DEEPWATER SYSTEMATICS] (Available: Ocean Biodiversity Information System. Intergovernmental Oceanographic Commission of UNESCO. https://obis.org. Accessed: 2019-08-29)

d6d6fe4c-425f-4ce7-bf28-7a6befaeb413	National Museum of Natural History Vertebrate Zoology Fishes Collections	National Museum of Natural History, Smithsonian Institution NMNH Fishes Collection Database. National Museum of Natural History, Smithsonian Institution, 10th and Constitution Ave. N.W., Washington, DC 20560-0193, 2007.	This work is licensed under a Creative Commons Attribution (CC-BY) 4.0 License	OBIS (2019) [National Museum of Natural History Vertebrate Zoology Fishes Collections] (Available: Ocean Biodiversity Information System. Intergovernmental Oceanographic Commission of UNESCO. https://obis.org. Accessed: 2019-08-29)

Diaphus metopoclampus

GBIF

GBIF Occurrence Download https://doi.org/10.15468/dd.cssan7 Accessed from R via rgbif (https://github.com/ropensci/rgbif) on 2019-09-11

OBIS

OBIS (2019) Distribution records of Diaphus metopoclampus [Dataset] (Available: Ocean Biodiversity Information System. Intergovernmental Oceanographic Commission of UNESCO. www.obis.org. Accessed: 2019-08-29)

Dataset details:

Dataset_ID	Name	Citation	License	OBIS_citation
1f59030f-f116-4c34-915e-1882d819cda3	Institution Southampton Oceanography Ceter - Collection discovery	No available dataset citation	Unspecified intellectual rights	OBIS (2019) [Institution Southampton Oceanography Ceter - Collection discovery] (Available: Ocean Biodiversity Information System. Intergovernmental Oceanographic Commission of UNESCO. https://obis.org. Accessed: 2019-08-29)
270f3e70-ff9b-411d-b170-2bc914d83f26	Biological Reference Collections ICM CSIC	Olivas González F J (2016): Biological Reference Collections ICM CSIC. Institute of Marine Sciences (ICM-CSIC). https://dx.doi.org/10.15470/qlqqdx	This work is licensed under a Creative Commons Attribution Non Commercial (CC-BY-NC) 4.0 License	OBIS (2019) [Biological Reference Collections ICM CSIC] (Available: Ocean Biodiversity Information System. Intergovernmental Oceanographic Commission of UNESCO. https://obis.org. Accessed: 2019-08-29)

2870c548-343e-4575-ac67-a4da35182c52	Institution Shirshov Institute - Collection SKAO	No available dataset citation	Unspecified intellectual rights	OBIS (2019) [Institution Shirshov Institute - Collection SKAO] (Available: Ocean Biodiversity Information System. Intergovernmental Oceanographic Commission of UNESCO. https://obis.org. Accessed: 2019-08-29)
8629ec33-be4b-4384-933f-a511fbc29967	MAR-ECO 2004	Wenneck, T. de Lange, Falkenhaug, T. and O.A. Bergstad. 2008. Strategies, methods, and technologies adopted on the RV G.O. Sars MAR-ECO expedition to the mid-Atlantic Ridge in 2004. Deep-sea Research II. 55: 6-28.	This work is licensed under a Creative Commons Attribution (CC-BY) 4.0 License	OBIS (2019) [MAR-ECO 2004] (Available: Ocean Biodiversity Information System. Intergovernmental Oceanographic Commission of UNESCO. https://obis.org. Accessed: 2019-08-29)
8a1ae661-e911-4967-bc06-1168fc5f2d89	iziko South African Museum - Fish Collection	iziko South African Museum - Fish Collection	Restricted	OBIS (2019) [iziko South African Museum - Fish Collection] (Available: Ocean Biodiversity Information System. Intergovernmental Oceanographic Commission of UNESCO. https://obis.org. Accessed: 2019-08-29)
b8617377-eb1c-4db2-baa6-8788a632e810	Ichthyology Collection - Royal Ontario Museum	NA	This work is licensed under a Creative Commons Attribution Non Commercial (CC-BY-NC) 4.0 License	OBIS (2019) [Ichthyology Collection - Royal Ontario Museum] (Available: Ocean Biodiversity Information System. Intergovernmental Oceanographic Commission of UNESCO. https://obis.org. Accessed: 2019-08-29)
cc8f28ce-e48d-4945-abfe-9d150a22dcd6	Hamburg pelagic fish database	Post, A. 1987. Pelagic transects of FRVs "Walther Herwig" and "Anton Dohrn" in the Atlantic Ocean 1966 to 1986. Mitt. Inst. f. Seefischerei d. BfaFi Hamburg, 42: 1-68.	This work is licensed under a Creative Commons Attribution (CC-BY) 4.0 License	OBIS (2019) [Hamburg pelagic fish database] (Available: Ocean Biodiversity Information System. Intergovernmental Oceanographic Commission of UNESCO. https://obis.org. Accessed: 2019-08-29)
ce1d93f3-8b0f-4ee7-9a4d-0393a6ec7fea	Atlantic Reference Centre Museum of Canadian Atlantic Organisms - Invertebrates and Fishes Data	Van Guelpen, L., 2016. Atlantic Reference Centre Museum of Canadian Atlantic Organisms - Invertebrates and Fishes Data. Version 4 In OBIS Canada Digital Collections. Bedford Institute of Oceanography, Dartmouth, NS, Canada. Published by OBIS, Digital http://www.iobis.org/. Accessed on –INSERT DATE	This work is licensed under a Creative Commons Attribution (CC-BY) 4.0 License	OBIS (2019) [Atlantic Reference Centre Museum of Canadian Atlantic Organisms - Invertebrates and Fishes Data] (Available: Ocean Biodiversity Information System. Intergovernmental Oceanographic Commission of UNESCO. https://obis.org. Accessed: 2019-08-29)

d6d6fe4c-425f-4ce7-bf28-7a6befaeb413	National Museum of Natural History Vertebrate Zoology Fishes Collections	National Museum of Natural History, Smithsonian Institution NMNH Fishes Collection Database. National Museum of Natural History, Smithsonian Institution, 10th and Constitution Ave. N.W., Washington, DC 20560-0193, 2007.	This work is licensed under a Creative Commons Attribution (CC-BY) 4.0 License	OBIS (2019) [National Museum of Natural History Vertebrate Zoology Fishes Collections] (Available: Ocean Biodiversity Information System. Intergovernmental Oceanographic Commission of UNESCO. https://obis.org. Accessed: 2019-08-29)

Lampadena dea

GBIF

GBIF Occurrence Download https://doi.org/10.15468/dd.dkzt9k Accessed from R via rgbif (https://github.com/ropensci/rgbif) on 2019-09-11

OBIS

OBIS (2019) Distribution records of Lampadena dea [Dataset] (Available: Ocean Biodiversity Information System. Intergovernmental Oceanographic Commission of UNESCO. www.obis.org. Accessed: 2019-08-29)

Dataset details:

Dataset_ID	Name	Citation	License	OBIS_citation
2870c548-343e-4575-ac67-a4da35182c52	Institution Shirshov Institute - Collection SKAO	No available dataset citation	Unspecified intellectual rights	OBIS (2019) [Institution Shirshov Institute - Collection SKAO] (Available: Ocean Biodiversity Information System. Intergovernmental Oceanographic Commission of UNESCO. https://obis.org. Accessed: 2019-08-29)
4bdc1f6f-e16a-48b4-b995-b51bd41caa8d	Dataset of the multidisciplinary research surveys in the seamounts of Ewing and Valdivia Bank (Walvis Ridge) - SE Atlantic	López-Abellán, L. J.; Sarralde Vizuete, R.; González Jiménez, J. F.; Centro Oceanográfico de Canarias – IEO, Spain (2015). Dataset of the multidisciplinary research surveys in the seamounts of Ewing and Valdivia Bank (Walvis Ridge) - SE Atlantic https://dx.doi.org/10.14284/58	Attribution-NoDerivatives (CC BY-ND)	OBIS (2019) [Dataset of the multidisciplinary research surveys in the seamounts of Ewing and Valdivia Bank (Walvis Ridge) - SE Atlantic] (Available: Ocean Biodiversity Information System. Intergovernmental Oceanographic Commission of UNESCO. https://obis.org. Accessed: 2019-08-29)

8a1ae661-e911-4967-bc06-1168fc5f2d89	iziko South African Museum - Fish Collection	iziko South African Museum - Fish Collection	Restricted	OBIS (2019) [iziko South African Museum - Fish Collection] (Available: Ocean Biodiversity Information System. Intergovernmental Oceanographic Commission of UNESCO. https://obis.org. Accessed: 2019-08-29)
d6d6fe4c-425f-4ce7-bf28-7a6befaeb413	National Museum of Natural History Vertebrate Zoology Fishes Collections	National Museum of Natural History, Smithsonian Institution NMNH Fishes Collection Database. National Museum of Natural History, Smithsonian Institution, 10th and Constitution Ave. N.W., Washington, DC 20560-0193, 2007.	This work is licensed under a Creative Commons Attribution (CC-BY) 4.0 License	OBIS (2019) [National Museum of Natural History Vertebrate Zoology Fishes Collections] (Available: Ocean Biodiversity Information System. Intergovernmental Oceanographic Commission of UNESCO. https://obis.org. Accessed: 2019-08-29)

Diaphus anderseni

GBIF

GBIF Occurrence Download https://doi.org/10.15468/dd.gbmsdr Accessed from R via rgbif (https://github.com/ropensci/rgbif) on 2019-09-11

OBIS

OBIS (2019) Distribution records of Diaphus anderseni [Dataset] (Available: Ocean Biodiversity Information System. Intergovernmental Oceanographic Commission of UNESCO. www.obis.org. Accessed: 2019-08-29)

Dataset details:

Dataset_ID	Name	Citation	License	OBIS_citation
10b213e6-a9c4-459e-a40c-ef9edc461b97	Marine data from the Bernice P. Bishop Museum	Pyle R (2016). Bernice P. Bishop Museum. Version 8.1. Bernice Pauahi Bishop Museum. Occurrence dataset https://doi.org/10.15468/s6ctus accessed via GBIF.org on 2018-11-16.	To the extent possible under law, the publisher has waived all rights to these data and has dedicated them to the Public Domain (CC0 1.0)	OBIS (2019) [Marine data from the Bernice P. Bishop Museum] (Available: Ocean Biodiversity Information System. Intergovernmental Oceanographic Commission of UNESCO. https://obis.org. Accessed: 2019-08-29)

2870c548-343e-4575-ac67-a4da35182c52	Institution Shirshov Institute - Collection SKAO	No available dataset citation	Unspecified intellectual rights	OBIS (2019) [Institution Shirshov Institute - Collection SKAO] (Available: Ocean Biodiversity Information System. Intergovernmental Oceanographic Commission of UNESCO. https://obis.org. Accessed: 2019-08-29)
6a5bc28f-4dfe-4cbf-8a55-7e3a843997ab	SPC NECTALIS Zooplankton/Micronekton specimens, New Caledonia 2014	Allain, V., Menkes, C., 2014. Nectalis 3 cruise, RV Alis. https://doi.org/10.17600/14004900	This work is licensed under a Creative Commons Attribution Non Commercial (CC-BY-NC) 4.0 License	OBIS (2019) [SPC NECTALIS Zooplankton/Micronekton specimens, New Caledonia 2014] (Available: Ocean Biodiversity Information System. Intergovernmental Oceanographic Commission of UNESCO. https://obis.org. Accessed: 2019-08-29)
705770e5-3474-4e69-be8b-3107a0c5610a	The fishes collection (IC) of the Muséum national d'Histoire naturelle (MNHN - Paris)	Gicim data base, Pruvost P. Causse R., 2009 http://doi.org/10.15468/tm7whu	This work is licensed under a Creative Commons Attribution (CC-BY) 4.0 License	OBIS (2019) [The fishes collection (IC) of the Muséum national d'Histoire naturelle (MNHN - Paris)] (Available: Ocean Biodiversity Information System. Intergovernmental Oceanographic Commission of UNESCO. https://obis.org. Accessed: 2019-08-29)
8a1ae661-e911-4967-bc06-1168fc5f2d89	iziko South African Museum - Fish Collection	iziko South African Museum - Fish Collection	Restricted	OBIS (2019) [iziko South African Museum - Fish Collection] (Available: Ocean Biodiversity Information System. Intergovernmental Oceanographic Commission of UNESCO. https://obis.org. Accessed: 2019-08-29)
b8617377-eb1c-4db2-baa6-8788a632e810	Ichthyology Collection - Royal Ontario Museum	NA	This work is licensed under a Creative Commons Attribution Non Commercial (CC-BY-NC) 4.0 License	OBIS (2019) [Ichthyology Collection - Royal Ontario Museum] (Available: Ocean Biodiversity Information System. Intergovernmental Oceanographic Commission of UNESCO. https://obis.org. Accessed: 2019-08-29)
c24bf1c2-2c62-4056-a841-56d94e6e876a	Fish specimens	ROM Fish Collection (accessed through GBIF data portal, http://data.gbif.org/datasets/resource/660, 2012-01-20) http://doi.org/10.15468/syisbx	Unrestricted	OBIS (2019) [Fish specimens] (Available: Ocean Biodiversity Information System. Intergovernmental Oceanographic Commission of UNESCO. https://obis.org. Accessed: 2019-08-29)

ce1d93f3-8b0f-4ee7-9a4d-0393a6ec7fea	Atlantic Reference Centre Museum of Canadian Atlantic Organisms - Invertebrates and Fishes Data	Van Guelpen, L., 2016. Atlantic Reference Centre Museum of Canadian Atlantic Organisms - Invertebrates and Fishes Data. Version 4 In OBIS Canada Digital Collections. Bedford Institute of Oceanography, Dartmouth, NS, Canada. Published by OBIS, Digital http://www.iobis.org/. Accessed on –INSERT DATE	This work is licensed under a Creative Commons Attribution (CC-BY) 4.0 License	OBIS (2019) [Atlantic Reference Centre Museum of Canadian Atlantic Organisms - Invertebrates and Fishes Data] (Available: Ocean Biodiversity Information System. Intergovernmental Oceanographic Commission of UNESCO. https://obis.org. Accessed: 2019-08-29)

Diaphus perspicillatus

GBIF

GBIF Occurrence Download https://doi.org/10.15468/dd.pgvcra Accessed from R via rgbif (https://github.com/ropensci/rgbif) on 2019-09-11

OBIS

OBIS (2019) Distribution records of Diaphus perspicillatus [Dataset] (Available: Ocean Biodiversity Information System. Intergovernmental Oceanographic Commission of UNESCO. www.obis.org. Accessed: 2019-08-29)

Dataset details:

Dataset_ID	Name	Citation	License	OBIS_citation
270f3e70-ff9b-411d-b170-2bc914d83f26	Biological Reference Collections ICM CSIC	Olivas González F J (2016): Biological Reference Collections ICM CSIC. Institute of Marine Sciences (ICM-CSIC). https://dx.doi.org/10.15470/qlqqdx	This work is licensed under a Creative Commons Attribution Non Commercial (CC-BY-NC) 4.0 License	OBIS (2019) [Biological Reference Collections ICM CSIC] (Available: Ocean Biodiversity Information System. Intergovernmental Oceanographic Commission of UNESCO. https://obis.org. Accessed: 2019-08-29)
6a5bc28f-4dfe-4cbf-8a55-7e3a843997ab	SPC NECTALIS Zooplankton/Micronekton specimens, New Caledonia 2014	Allain, V., Menkes, C., 2014. Nectalis 3 cruise, RV Alis. https://doi.org/10.17600/14004900	This work is licensed under a Creative Commons Attribution Non Commercial (CC-BY-NC) 4.0 License	OBIS (2019) [SPC NECTALIS Zooplankton/Micronekton specimens, New Caledonia 2014] (Available: Ocean Biodiversity Information System. Intergovernmental Oceanographic Commission of UNESCO. https://obis.org. Accessed: 2019-08-29)

6c19184e-c305-4273-8890-6d342d86f865	Institution REVIZEE - Collection Pelagic Fishes	No available dataset citation	Unspecified intellectual rights	OBIS (2019) [Institution REVIZEE - Collection Pelagic Fishes] (Available: Ocean Biodiversity Information System. Intergovernmental Oceanographic Commission of UNESCO. https://obis.org. Accessed: 2019-08-29)
705770e5-3474-4e69-be8b-3107a0c5610a	The fishes collection (IC) of the Muséum national d'Histoire naturelle (MNHN - Paris)	Gicim data base, Pruvost P. Causse R., 2009 http://doi.org/10.15468/tm7whu	This work is licensed under a Creative Commons Attribution (CC-BY) 4.0 License	OBIS (2019) [The fishes collection (IC) of the Muséum national d'Histoire naturelle (MNHN - Paris)] (Available: Ocean Biodiversity Information System. Intergovernmental Oceanographic Commission of UNESCO. https://obis.org. Accessed: 2019-08-29)
8a1ae661-e911-4967-bc06-1168fc5f2d89	iziko South African Museum - Fish Collection	iziko South African Museum - Fish Collection	Restricted	OBIS (2019) [iziko South African Museum - Fish Collection] (Available: Ocean Biodiversity Information System. Intergovernmental Oceanographic Commission of UNESCO. https://obis.org. Accessed: 2019-08-29)
b8617377-eb1c-4db2-baa6-8788a632e810	Ichthyology Collection - Royal Ontario Museum	NA	This work is licensed under a Creative Commons Attribution Non Commercial (CC-BY-NC) 4.0 License	OBIS (2019) [Ichthyology Collection - Royal Ontario Museum] (Available: Ocean Biodiversity Information System. Intergovernmental Oceanographic Commission of UNESCO. https://obis.org. Accessed: 2019-08-29)
c24bf1c2-2c62-4056-a841-56d94e6e876a	Fish specimens	ROM Fish Collection (accessed through GBIF data portal, http://data.gbif.org/datasets/resource/660, 2012-01-20) http://doi.org/10.15468/syisbx	Unrestricted	OBIS (2019) [Fish specimens] (Available: Ocean Biodiversity Information System. Intergovernmental Oceanographic Commission of UNESCO. https://obis.org. Accessed: 2019-08-29)
cc8f28ce-e48d-4945-abfe-9d150a22dcd6	Hamburg pelagic fish database	Post, A. 1987. Pelagic transects of FRVs "Walther Herwig" and "Anton Dohrn" in the Atlantic Ocean 1966 to 1986. Mitt. Inst. f. Seefischerei d. BfaFi Hamburg, 42: 1-68.	This work is licensed under a Creative Commons Attribution (CC-BY) 4.0 License	OBIS (2019) [Hamburg pelagic fish database] (Available: Ocean Biodiversity Information System. Intergovernmental Oceanographic Commission of UNESCO. https://obis.org. Accessed: 2019-08-29)

ce1d93f3-8b0f-4ee7-9a4d-0393a6ec7fea	Atlantic Reference Centre Museum of Canadian Atlantic Organisms - Invertebrates and Fishes Data	Van Guelpen, L., 2016. Atlantic Reference Centre Museum of Canadian Atlantic Organisms - Invertebrates and Fishes Data. Version 4 In OBIS Canada Digital Collections. Bedford Institute of Oceanography, Dartmouth, NS, Canada. Published by OBIS, Digital http://www.iobis.org/. Accessed on –INSERT DATE	This work is licensed under a Creative Commons Attribution (CC-BY) 4.0 License	OBIS (2019) [Atlantic Reference Centre Museum of Canadian Atlantic Organisms - Invertebrates and Fishes Data] (Available: Ocean Biodiversity Information System. Intergovernmental Oceanographic Commission of UNESCO. https://obis.org. Accessed: 2019-08-29)
d6d6fe4c-425f-4ce7-bf28-7a6befaeb413	National Museum of Natural History Vertebrate Zoology Fishes Collections	National Museum of Natural History, Smithsonian Institution NMNH Fishes Collection Database. National Museum of Natural History, Smithsonian Institution, 10th and Constitution Ave. N.W., Washington, DC 20560-0193, 2007.	This work is licensed under a Creative Commons Attribution (CC-BY) 4.0 License	OBIS (2019) [National Museum of Natural History Vertebrate Zoology Fishes Collections] (Available: Ocean Biodiversity Information System. Intergovernmental Oceanographic Commission of UNESCO. https://obis.org. Accessed: 2019-08-29)

Lobianchia gemellarii

GBIF

GBIF Occurrence Download https://doi.org/10.15468/dd.8zha5j Accessed from R via rgbif (https://github.com/ropensci/rgbif) on 2019-09-11

OBIS

OBIS (2019) Distribution records of Lobianchia gemellarii [Dataset] (Available: Ocean Biodiversity Information System. Intergovernmental Oceanographic Commission of UNESCO. www.obis.org. Accessed: 2019-08-29)

Dataset details:

Dataset_ID	Name	Citation	License	OBIS_citation
10b213e6-a9c4-459e-a40c-ef9edc461b97	Marine data from the Bernice P. Bishop Museum	Pyle R (2016). Bernice P. Bishop Museum. Version 8.1. Bernice Pauahi Bishop Museum. Occurrence dataset https://doi.org/10.15468/s6ctus accessed via GBIF.org on 2018-11-16.	To the extent possible under law, the publisher has waived all rights to these data and has dedicated them to the Public Domain (CC0 1.0)	OBIS (2019) [Marine data from the Bernice P. Bishop Museum] (Available: Ocean Biodiversity Information System. Intergovernmental Oceanographic Commission of UNESCO. https://obis.org. Accessed: 2019-08-29)

1f59030f-f116-4c34-915e-1882d819cda3	Institution Southampton Oceanography Ceter - Collection discovery	No available dataset citation	Unspecified intellectual rights	OBIS (2019) [Institution Southampton Oceanography Ceter - Collection discovery] (Available: Ocean Biodiversity Information System. Intergovernmental Oceanographic Commission of UNESCO. https://obis.org. Accessed: 2019-08-29)
270f3e70-ff9b-411d-b170-2bc914d83f26	Biological Reference Collections ICM CSIC	Olivas González F J (2016): Biological Reference Collections ICM CSIC. Institute of Marine Sciences (ICM-CSIC). https://dx.doi.org/10.15470/qlqqdx	This work is licensed under a Creative Commons Attribution Non Commercial (CC-BY-NC) 4.0 License	OBIS (2019) [Biological Reference Collections ICM CSIC] (Available: Ocean Biodiversity Information System. Intergovernmental Oceanographic Commission of UNESCO. https://obis.org. Accessed: 2019-08-29)
2870c548-343e-4575-ac67-a4da35182c52	Institution Shirshov Institute - Collection SKAO	No available dataset citation	Unspecified intellectual rights	OBIS (2019) [Institution Shirshov Institute - Collection SKAO] (Available: Ocean Biodiversity Information System. Intergovernmental Oceanographic Commission of UNESCO. https://obis.org. Accessed: 2019-08-29)
308a501c-a187-498e-a8bd-9cf3d2b70bd9	Institution REVIZEE - Collection Ictioplankton	No available dataset citation	Unspecified intellectual rights	OBIS (2019) [Institution REVIZEE - Collection Ictioplankton] (Available: Ocean Biodiversity Information System. Intergovernmental Oceanographic Commission of UNESCO. https://obis.org. Accessed: 2019-08-29)
4e25e0ce-b17d-4192-9b55-417f1e0c4fc8	Institution KU - Collection KUI	No available dataset citation	Unspecified intellectual rights	OBIS (2019) [Institution KU - Collection KUI] (Available: Ocean Biodiversity Information System. Intergovernmental Oceanographic Commission of UNESCO. https://obis.org. Accessed: 2019-08-29)
623f4f98-4e66-4bee-9e1c-17cb74cd2d21	Institution IEO - Collection MEDITS-Spain	No available dataset citation	Unspecified intellectual rights	OBIS (2019) [Institution IEO - Collection MEDITS-Spain] (Available: Ocean Biodiversity Information System. Intergovernmental Oceanographic Commission of UNESCO. https://obis.org. Accessed: 2019-08-29)

6a5bc28f-4dfe-4cbf-8a55-7e3a843997ab	SPC NECTALIS Zooplankton/Micronekton specimens, New Caledonia 2014	Allain, V., Menkes, C., 2014. Nectalis 3 cruise, RV Alis. https://doi.org/10.17600/14004900	This work is licensed under a Creative Commons Attribution Non Commercial (CC-BY-NC) 4.0 License	OBIS (2019) [SPC NECTALIS Zooplankton/Micronekton specimens, New Caledonia 2014] (Available: Ocean Biodiversity Information System. Intergovernmental Oceanographic Commission of UNESCO. https://obis.org. Accessed: 2019-08-29)
705770e5-3474-4e69-be8b-3107a0c5610a	The fishes collection (IC) of the Muséum national d'Histoire naturelle (MNHN - Paris)	Gicim data base, Pruvost P. Causse R., 2009 http://doi.org/10.15468/tm7whu	This work is licensed under a Creative Commons Attribution (CC-BY) 4.0 License	OBIS (2019) [The fishes collection (IC) of the Muséum national d'Histoire naturelle (MNHN - Paris)] (Available: Ocean Biodiversity Information System. Intergovernmental Oceanographic Commission of UNESCO. https://obis.org. Accessed: 2019-08-29)
8629ec33-be4b-4384-933f-a511fbc29967	MAR-ECO 2004	Wenneck, T. de Lange, Falkenhaug, T. and O.A. Bergstad. 2008. Strategies, methods, and technologies adopted on the RV G.O. Sars MAR-ECO expedition to the mid-Atlantic Ridge in 2004. Deep-sea Research II. 55: 6-28.	This work is licensed under a Creative Commons Attribution (CC-BY) 4.0 License	OBIS (2019) [MAR-ECO 2004] (Available: Ocean Biodiversity Information System. Intergovernmental Oceanographic Commission of UNESCO. https://obis.org. Accessed: 2019-08-29)
8a1ae661-e911-4967-bc06-1168fc5f2d89	iziko South African Museum - Fish Collection	iziko South African Museum - Fish Collection	Restricted	OBIS (2019) [iziko South African Museum - Fish Collection] (Available: Ocean Biodiversity Information System. Intergovernmental Oceanographic Commission of UNESCO. https://obis.org. Accessed: 2019-08-29)
b8617377-eb1c-4db2-baa6-8788a632e810	Ichthyology Collection - Royal Ontario Museum	NA	This work is licensed under a Creative Commons Attribution Non Commercial (CC-BY-NC) 4.0 License	OBIS (2019) [Ichthyology Collection - Royal Ontario Museum] (Available: Ocean Biodiversity Information System. Intergovernmental Oceanographic Commission of UNESCO. https://obis.org. Accessed: 2019-08-29)
c24bf1c2-2c62-4056-a841-56d94e6e876a	Fish specimens	ROM Fish Collection (accessed through GBIF data portal, http://data.gbif.org/datasets/resource/660, 2012-01-20) http://doi.org/10.15468/syisbx	Unrestricted	OBIS (2019) [Fish specimens] (Available: Ocean Biodiversity Information System. Intergovernmental Oceanographic Commission of UNESCO. https://obis.org. Accessed: 2019-08-29)

cc8f28ce-e48d-4945-abfe-9d150a22dcd6	Hamburg pelagic fish database	Post, A. 1987. Pelagic transects of FRVs "Walther Herwig" and "Anton Dohrn" in the Atlantic Ocean 1966 to 1986. Mitt. Inst. f. Seefischerei d. BfaFi Hamburg, 42: 1-68.	This work is licensed under a Creative Commons Attribution (CC-BY) 4.0 License	OBIS (2019) [Hamburg pelagic fish database] (Available: Ocean Biodiversity Information System. Intergovernmental Oceanographic Commission of UNESCO. https://obis.org. Accessed: 2019-08-29)
ce1d93f3-8b0f-4ee7-9a4d-0393a6ec7fea	Atlantic Reference Centre Museum of Canadian Atlantic Organisms - Invertebrates and Fishes Data	Van Guelpen, L., 2016. Atlantic Reference Centre Museum of Canadian Atlantic Organisms - Invertebrates and Fishes Data. Version 4 In OBIS Canada Digital Collections. Bedford Institute of Oceanography, Dartmouth, NS, Canada. Published by OBIS, Digital http://www.iobis.org/. Accessed on –INSERT DATE	This work is licensed under a Creative Commons Attribution (CC-BY) 4.0 License	OBIS (2019) [Atlantic Reference Centre Museum of Canadian Atlantic Organisms - Invertebrates and Fishes Data] (Available: Ocean Biodiversity Information System. Intergovernmental Oceanographic Commission of UNESCO. https://obis.org. Accessed: 2019-08-29)
cfc56587-48c3-4e3d-9350-3a4d9a28b681	Institution NOAA, NMFS, Northeast Fisheries Science Center - Collection DEEPWATER SYSTEMATICS	No available dataset citation	Unspecified intellectual rights	OBIS (2019) [Institution NOAA, NMFS, Northeast Fisheries Science Center - Collection DEEPWATER SYSTEMATICS] (Available: Ocean Biodiversity Information System. Intergovernmental Oceanographic Commission of UNESCO. https://obis.org. Accessed: 2019-08-29)
d286ae50-ea29-4aa4-8028-2e6e5945a039	Institution REVIZEE - Collection Demersal Fishes	No available dataset citation	Unspecified intellectual rights	OBIS (2019) [Institution REVIZEE - Collection Demersal Fishes] (Available: Ocean Biodiversity Information System. Intergovernmental Oceanographic Commission of UNESCO. https://obis.org. Accessed: 2019-08-29)
d6d6fe4c-425f-4ce7-bf28-7a6befaeb413	National Museum of Natural History Vertebrate Zoology Fishes Collections	National Museum of Natural History, Smithsonian Institution NMNH Fishes Collection Database. National Museum of Natural History, Smithsonian Institution, 10th and Constitution Ave. N.W., Washington, DC 20560-0193, 2007.	This work is licensed under a Creative Commons Attribution (CC-BY) 4.0 License	OBIS (2019) [National Museum of Natural History Vertebrate Zoology Fishes Collections] (Available: Ocean Biodiversity Information System. Intergovernmental Oceanographic Commission of UNESCO. https://obis.org. Accessed: 2019-08-29)

ff8b7809-41bc-40ad-8160-0e33862817a0	Biodiversity Research Museum, Academia Sinica, Taiwan	TELDAP, Biodiversity Research Museum, Academia Sinica, Taiwan (accessed through GBIF data portal, http://data.gbif.org/datasets/resource/9093, yyyy-mm-dd)	Unrestricted	OBIS (2019) [Biodiversity Research Museum, Academia Sinica, Taiwan] (Available: Ocean Biodiversity Information System. Intergovernmental Oceanographic Commission of UNESCO. https://obis.org. Accessed: 2019-08-29)

Bolinichthys longipes

GBIF

GBIF Occurrence Download https://doi.org/10.15468/dd.5gbk95 Accessed from R via rgbif (https://github.com/ropensci/rgbif) on 2019-09-11

OBIS

OBIS (2019) Distribution records of Bolinichthys longipes [Dataset] (Available: Ocean Biodiversity Information System. Intergovernmental Oceanographic Commission of UNESCO. www.obis.org. Accessed: 2019-08-29)

Dataset details:

Dataset_ID	Name	Citation	License	OBIS_citation
10b213e6-a9c4-459e-a40c-ef9edc461b97	Marine data from the Bernice P. Bishop Museum	Pyle R (2016). Bernice P. Bishop Museum. Version 8.1. Bernice Pauahi Bishop Museum. Occurrence dataset https://doi.org/10.15468/s6ctus accessed via GBIF.org on 2018-11-16.	To the extent possible under law, the publisher has waived all rights to these data and has dedicated them to the Public Domain (CC0 1.0)	OBIS (2019) [Marine data from the Bernice P. Bishop Museum] (Available: Ocean Biodiversity Information System. Intergovernmental Oceanographic Commission of UNESCO. https://obis.org. Accessed: 2019-08-29)
1f59030f-f116-4c34-915e-1882d819cda3	Institution Southampton Oceanography Ceter - Collection discovery	No available dataset citation	Unspecified intellectual rights	OBIS (2019) [Institution Southampton Oceanography Ceter - Collection discovery] (Available: Ocean Biodiversity Information System. Intergovernmental Oceanographic Commission of UNESCO. https://obis.org. Accessed: 2019-08-29)

6a5bc28f-4dfe-4cbf-8a55-7e3a843997ab	SPC NECTALIS Zooplankton/Micronekton specimens, New Caledonia 2014	Allain, V., Menkes, C., 2014. Nectalis 3 cruise, RV Alis. https://doi.org/10.17600/14004900	This work is licensed under a Creative Commons Attribution Non Commercial (CC-BY-NC) 4.0 License	OBIS (2019) [SPC NECTALIS Zooplankton/Micronekton specimens, New Caledonia 2014] (Available: Ocean Biodiversity Information System. Intergovernmental Oceanographic Commission of UNESCO. https://obis.org. Accessed: 2019-08-29)
705770e5-3474-4e69-be8b-3107a0c5610a	The fishes collection (IC) of the Muséum national d'Histoire naturelle (MNHN - Paris)	Gicim data base, Pruvost P. Causse R., 2009 http://doi.org/10.15468/tm7whu	This work is licensed under a Creative Commons Attribution (CC-BY) 4.0 License	OBIS (2019) [The fishes collection (IC) of the Muséum national d'Histoire naturelle (MNHN - Paris)] (Available: Ocean Biodiversity Information System. Intergovernmental Oceanographic Commission of UNESCO. https://obis.org. Accessed: 2019-08-29)
d6d6fe4c-425f-4ce7-bf28-7a6befaeb413	National Museum of Natural History Vertebrate Zoology Fishes Collections	National Museum of Natural History, Smithsonian Institution NMNH Fishes Collection Database. National Museum of Natural History, Smithsonian Institution, 10th and Constitution Ave. N.W., Washington, DC 20560-0193, 2007.	This work is licensed under a Creative Commons Attribution (CC-BY) 4.0 License	OBIS (2019) [National Museum of Natural History Vertebrate Zoology Fishes Collections] (Available: Ocean Biodiversity Information System. Intergovernmental Oceanographic Commission of UNESCO. https://obis.org. Accessed: 2019-08-29)
ff8b7809-41bc-40ad-8160-0e33862817a0	Biodiversity Research Museum, Academia Sinica, Taiwan	TELDAP, Biodiversity Research Museum, Academia Sinica, Taiwan (accessed through GBIF data portal, http://data.gbif.org/datasets/resource/9093, yyyy-mm-dd)	Unrestricted	OBIS (2019) [Biodiversity Research Museum, Academia Sinica, Taiwan] (Available: Ocean Biodiversity Information System. Intergovernmental Oceanographic Commission of UNESCO. https://obis.org. Accessed: 2019-08-29)

Bolinichthys indicus

GBIF

GBIF Occurrence Download https://doi.org/10.15468/dd.vtqmxt Accessed from R via rgbif (https://github.com/ropensci/rgbif) on 2019-09-11

OBIS

OBIS (2019) Distribution records of Bolinichthys indicus [Dataset] (Available: Ocean Biodiversity Information System. Intergovernmental Oceanographic Commission of UNESCO. www.obis.org. Accessed: 2019-08-29)

Dataset details:

Dataset_ID	Name	Citation	License	OBIS_citation
09be818d-531c-4d86-8eba-5db81d42fdba	COLETA - IMAR/DOP-Uac reference collection from 1977 to 2012	Institute of Marine Research (IMAR - Azores), Portugal; Department of Oceanography and Fisheries (DOP) - UAC, Portugal (2015): COLETA - IMAR/DOP-Uac reference collection from 1977 to 2012. https://dx.doi.org/10.14284/23	This work is licensed under a Creative Commons Attribution (CC-BY) 4.0 License	OBIS (2019) [COLETA - IMAR/DOP-Uac reference collection from 1977 to 2012] (Available: Ocean Biodiversity Information System. Intergovernmental Oceanographic Commission of UNESCO. https://obis.org. Accessed: 2019-08-29)
1f59030f-f116-4c34-915e-1882d819cda3	Institution Southampton Oceanography Ceter - Collection discovery	No available dataset citation	Unspecified intellectual rights	OBIS (2019) [Institution Southampton Oceanography Ceter - Collection discovery] (Available: Ocean Biodiversity Information System. Intergovernmental Oceanographic Commission of UNESCO. https://obis.org. Accessed: 2019-08-29)
2870c548-343e-4575-ac67-a4da35182c52	Institution Shirshov Institute - Collection SKAO	No available dataset citation	Unspecified intellectual rights	OBIS (2019) [Institution Shirshov Institute - Collection SKAO] (Available: Ocean Biodiversity Information System. Intergovernmental Oceanographic Commission of UNESCO. https://obis.org. Accessed: 2019-08-29)
705770e5-3474-4e69-be8b-3107a0c5610a	The fishes collection (IC) of the Muséum national d'Histoire naturelle (MNHN - Paris)	Gicim data base, Pruvost P. Causse R., 2009 http://doi.org/10.15468/tm7whu	This work is licensed under a Creative Commons Attribution (CC-BY) 4.0 License	OBIS (2019) [The fishes collection (IC) of the Muséum national d'Histoire naturelle (MNHN - Paris)] (Available: Ocean Biodiversity Information System. Intergovernmental Oceanographic Commission of UNESCO. https://obis.org. Accessed: 2019-08-29)
7e4228e5-a962-4b01-952f-7bf33e213a9c	BioChem: Sameoto zooplankton collection	Sameoto, D.D., Kennedy, M., Spry, J.S, Spry, J.M. (2013). Zooplankton datasets collected using the BIONESS sampler, ring nets and an Icelandic high speed sampler, 1967-2006. OBIS Canada Digital Collections. Published by OBIS http://www.iobis.org/. Accessed on –INSERT DATE	rights: http://data.gc.ca/eng/open-government-licence-canada & http://www.canadensys.net/norms rights holder: Her Majesty the Queen in right of Canada, as represented by the Minister of Fisheries and Oceans	OBIS (2019) [BioChem: Sameoto zooplankton collection] (Available: Ocean Biodiversity Information System. Intergovernmental Oceanographic Commission of UNESCO. https://obis.org. Accessed: 2019-08-29)

8629ec33-be4b-4384-933f-a511fbc29967	MAR-ECO 2004	Wenneck, T. de Lange, Falkenhaug, T. and O.A. Bergstad. 2008. Strategies, methods, and technologies adopted on the RV G.O. Sars MAR-ECO expedition to the mid-Atlantic Ridge in 2004. Deep-sea Research II. 55: 6-28.	This work is licensed under a Creative Commons Attribution (CC-BY) 4.0 License	OBIS (2019) [MAR-ECO 2004] (Available: Ocean Biodiversity Information System. Intergovernmental Oceanographic Commission of UNESCO. https://obis.org. Accessed: 2019-08-29)
8a1ae661-e911-4967-bc06-1168fc5f2d89	iziko South African Museum - Fish Collection	iziko South African Museum - Fish Collection	Restricted	OBIS (2019) [iziko South African Museum - Fish Collection] (Available: Ocean Biodiversity Information System. Intergovernmental Oceanographic Commission of UNESCO. https://obis.org. Accessed: 2019-08-29)
b8617377-eb1c-4db2-baa6-8788a632e810	Ichthyology Collection - Royal Ontario Museum	NA	This work is licensed under a Creative Commons Attribution Non Commercial (CC-BY-NC) 4.0 License	OBIS (2019) [Ichthyology Collection - Royal Ontario Museum] (Available: Ocean Biodiversity Information System. Intergovernmental Oceanographic Commission of UNESCO. https://obis.org. Accessed: 2019-08-29)
c24bf1c2-2c62-4056-a841-56d94e6e876a	Fish specimens	ROM Fish Collection (accessed through GBIF data portal, http://data.gbif.org/datasets/resource/660, 2012-01-20) http://doi.org/10.15468/syisbx	Unrestricted	OBIS (2019) [Fish specimens] (Available: Ocean Biodiversity Information System. Intergovernmental Oceanographic Commission of UNESCO. https://obis.org. Accessed: 2019-08-29)
cc8f28ce-e48d-4945-abfe-9d150a22dcd6	Hamburg pelagic fish database	Post, A. 1987. Pelagic transects of FRVs "Walther Herwig" and "Anton Dohrn" in the Atlantic Ocean 1966 to 1986. Mitt. Inst. f. Seefischerei d. BfaFi Hamburg, 42: 1-68.	This work is licensed under a Creative Commons Attribution (CC-BY) 4.0 License	OBIS (2019) [Hamburg pelagic fish database] (Available: Ocean Biodiversity Information System. Intergovernmental Oceanographic Commission of UNESCO. https://obis.org. Accessed: 2019-08-29)
ce1d93f3-8b0f-4ee7-9a4d-0393a6ec7fea	Atlantic Reference Centre Museum of Canadian Atlantic Organisms - Invertebrates and Fishes Data	Van Guelpen, L., 2016. Atlantic Reference Centre Museum of Canadian Atlantic Organisms - Invertebrates and Fishes Data. Version 4 In OBIS Canada Digital Collections. Bedford Institute of Oceanography, Dartmouth, NS, Canada. Published by OBIS, Digital http://www.iobis.org/. Accessed on –INSERT DATE	This work is licensed under a Creative Commons Attribution (CC-BY) 4.0 License	OBIS (2019) [Atlantic Reference Centre Museum of Canadian Atlantic Organisms - Invertebrates and Fishes Data] (Available: Ocean Biodiversity Information System. Intergovernmental Oceanographic Commission of UNESCO. https://obis.org. Accessed: 2019-08-29)

d6d6fe4c-425f-4ce7-bf28-7a6befaeb413	National Museum of Natural History Vertebrate Zoology Fishes Collections	National Museum of Natural History, Smithsonian Institution NMNH Fishes Collection Database. National Museum of Natural History, Smithsonian Institution, 10th and Constitution Ave. N.W., Washington, DC 20560-0193, 2007.	This work is licensed under a Creative Commons Attribution (CC-BY) 4.0 License	OBIS (2019) [National Museum of Natural History Vertebrate Zoology Fishes Collections] (Available: Ocean Biodiversity Information System. Intergovernmental Oceanographic Commission of UNESCO. https://obis.org. Accessed: 2019-08-29)

Diaphus danae

GBIF

GBIF Occurrence Download https://doi.org/10.15468/dd.z2fwh9 Accessed from R via rgbif (https://github.com/ropensci/rgbif) on 2019-09-11

OBIS

OBIS (2019) Distribution records of Diaphus danae [Dataset] (Available: Ocean Biodiversity Information System. Intergovernmental Oceanographic Commission of UNESCO. www.obis.org. Accessed: 2019-08-29)

Dataset details:

Dataset_ID	Name	Citation	License	OBIS_citation
0c9db499-759b-46d8-8989-799f9ff9f235	Auckland Museum NZ Marine Collection	Blom W, Moriarty A (2018). Auckland Museum NZ Marine Collection. Version 1.11. Auckland War Memorial Museum. Occurrence Dataset https://doi.org/10.15468/plyefd accessed via GBIF.org on 2018-01-15.	This work is licensed under a Creative Commons Attribution (CC-BY) 4.0 License	OBIS (2019) [Auckland Museum NZ Marine Collection] (Available: Ocean Biodiversity Information System. Intergovernmental Oceanographic Commission of UNESCO. https://obis.org. Accessed: 2019-08-29)
50903a57-ee9f-4367-b2cd-0b36dcf4a6ad	Catch data from New Zealand research trawls since 2008	SWPRON (2017). Catch data from New Zealand research trawls. Southwestern Pacific OBIS, National Institute of Water and Atmospheric Research (NIWA), Wellington, New Zealand, 15157 records, Online http://nzobisipt.niwa.co.nz/resource.do?r=trawl released on April 19, 2017.	This work is licensed under a Creative Commons Attribution (CC-BY) 4.0 License	OBIS (2019) [Catch data from New Zealand research trawls since 2008] (Available: Ocean Biodiversity Information System. Intergovernmental Oceanographic Commission of UNESCO. https://obis.org. Accessed: 2019-08-29)

d6d6fe4c-425f-4ce7-bf28-7a6befaeb413	National Museum of Natural History Vertebrate Zoology Fishes Collections	National Museum of Natural History, Smithsonian Institution NMNH Fishes Collection Database. National Museum of Natural History, Smithsonian Institution, 10th and Constitution Ave. N.W., Washington, DC 20560-0193, 2007.	This work is licensed under a Creative Commons Attribution (CC-BY) 4.0 License	OBIS (2019) [National Museum of Natural History Vertebrate Zoology Fishes Collections] (Available: Ocean Biodiversity Information System. Intergovernmental Oceanographic Commission of UNESCO. https://obis.org. Accessed: 2019-08-29)

Benthosema fibulatum

GBIF

GBIF Occurrence Download https://doi.org/10.15468/dd.dumknc Accessed from R via rgbif (https://github.com/ropensci/rgbif) on 2019-09-11

OBIS

OBIS (2019) Distribution records of Benthosema fibulatum [Dataset] (Available: Ocean Biodiversity Information System. Intergovernmental Oceanographic Commission of UNESCO. www.obis.org. Accessed: 2019-08-29)

Dataset details:

Dataset_ID	Name	Citation	License	OBIS_citation
04e3fd32-b08b-4806-a016-d2dff52ae55a	Asia-Pacific Dataset	Jintsu-Uchifune, Y., Yamamoto, H. (2016) Marine organism occurrence data of the Asia-Pacific region extracted from literature. Available at https://doi.org/10.48518/00002. Accessed on yyyy-mm-dd.	This work is licensed under a Creative Commons Attribution (CC-BY) 4.0 License	OBIS (2019) [Asia-Pacific Dataset] (Available: Ocean Biodiversity Information System. Intergovernmental Oceanographic Commission of UNESCO. https://obis.org. Accessed: 2019-08-29)
10b213e6-a9c4-459e-a40c-ef9edc461b97	Marine data from the Bernice P. Bishop Museum	Pyle R (2016). Bernice P. Bishop Museum. Version 8.1. Bernice Pauahi Bishop Museum. Occurrence dataset https://doi.org/10.15468/s6ctus accessed via GBIF.org on 2018-11-16.	To the extent possible under law, the publisher has waived all rights to these data and has dedicated them to the Public Domain (CC0 1.0)	OBIS (2019) [Marine data from the Bernice P. Bishop Museum] (Available: Ocean Biodiversity Information System. Intergovernmental Oceanographic Commission of UNESCO. https://obis.org. Accessed: 2019-08-29)

1f59030f-f116-4c34-915e-1882d819cda3	Institution Southampton Oceanography Ceter - Collection discovery	No available dataset citation	Unspecified intellectual rights	OBIS (2019) [Institution Southampton Oceanography Ceter - Collection discovery] (Available: Ocean Biodiversity Information System. Intergovernmental Oceanographic Commission of UNESCO. https://obis.org. Accessed: 2019-08-29)
270f3e70-ff9b-411d-b170-2bc914d83f26	Biological Reference Collections ICM CSIC	Olivas González F J (2016): Biological Reference Collections ICM CSIC. Institute of Marine Sciences (ICM-CSIC). https://dx.doi.org/10.15470/qlqqdx	This work is licensed under a Creative Commons Attribution Non Commercial (CC-BY-NC) 4.0 License	OBIS (2019) [Biological Reference Collections ICM CSIC] (Available: Ocean Biodiversity Information System. Intergovernmental Oceanographic Commission of UNESCO. https://obis.org. Accessed: 2019-08-29)
3a96d77d-a1fa-491d-9705-d885fe93a802	Taiwan bottom trawl survey	NA	Unrestricted	OBIS (2019) [Taiwan bottom trawl survey] (Available: Ocean Biodiversity Information System. Intergovernmental Oceanographic Commission of UNESCO. https://obis.org. Accessed: 2019-08-29)
705770e5-3474-4e69-be8b-3107a0c5610a	The fishes collection (IC) of the Muséum national d'Histoire naturelle (MNHN - Paris)	Gicim data base, Pruvost P. Causse R., 2009 http://doi.org/10.15468/tm7whu	This work is licensed under a Creative Commons Attribution (CC-BY) 4.0 License	OBIS (2019) [The fishes collection (IC) of the Muséum national d'Histoire naturelle (MNHN - Paris)] (Available: Ocean Biodiversity Information System. Intergovernmental Oceanographic Commission of UNESCO. https://obis.org. Accessed: 2019-08-29)
8a1ae661-e911-4967-bc06-1168fc5f2d89	iziko South African Museum - Fish Collection	iziko South African Museum - Fish Collection	Restricted	OBIS (2019) [iziko South African Museum - Fish Collection] (Available: Ocean Biodiversity Information System. Intergovernmental Oceanographic Commission of UNESCO. https://obis.org. Accessed: 2019-08-29)
9ff216fc-777e-4f9b-9860-95ed7366870d	Institution SAIAB - Collection SAIAB	No available dataset citation	Unspecified intellectual rights	OBIS (2019) [Institution SAIAB - Collection SAIAB] (Available: Ocean Biodiversity Information System. Intergovernmental Oceanographic Commission of UNESCO. https://obis.org. Accessed: 2019-08-29)

ff8b7809-41bc-40ad-8160-0e33862817a0	Biodiversity Research Museum, Academia Sinica, Taiwan	TELDAP, Biodiversity Research Museum, Academia Sinica, Taiwan (accessed through GBIF data portal, http://data.gbif.org/datasets/resource/9093, yyyy-mm-dd)	Unrestricted	OBIS (2019) [Biodiversity Research Museum, Academia Sinica, Taiwan] (Available: Ocean Biodiversity Information System. Intergovernmental Oceanographic Commission of UNESCO. https://obis.org. Accessed: 2019-08-29)

Diaphus watasei

GBIF

GBIF Occurrence Download https://doi.org/10.15468/dd.naajep Accessed from R via rgbif (https://github.com/ropensci/rgbif) on 2019-09-11

OBIS

OBIS (2019) Distribution records of Diaphus watasei [Dataset] (Available: Ocean Biodiversity Information System. Intergovernmental Oceanographic Commission of UNESCO. www.obis.org. Accessed: 2019-08-29)

Dataset details:

Dataset_ID	Name	Citation	License	OBIS_citation
3a96d77d-a1fa-491d-9705-d885fe93a802	Taiwan bottom trawl survey	NA	Unrestricted	OBIS (2019) [Taiwan bottom trawl survey] (Available: Ocean Biodiversity Information System. Intergovernmental Oceanographic Commission of UNESCO. https://obis.org. Accessed: 2019-08-29)
6963aa86-a154-459c-aeb0-0496668512f6	Marine Biological Sample Database, JAMSTEC	Japan Agency for Marine-Earth Science and Technology (2016 onwards). JAMSTEC Marine Biological Samples Database. https://doi.org/10.48518/00001. Accessed on yyyy-mm-dd.	This work is licensed under a Creative Commons Attribution Non Commercial (CC-BY-NC) 4.0 License	OBIS (2019) [Marine Biological Sample Database, JAMSTEC] (Available: Ocean Biodiversity Information System. Intergovernmental Oceanographic Commission of UNESCO. https://obis.org. Accessed: 2019-08-29)

705770e5-3474-4e69-be8b-3107a0c5610a	The fishes collection (IC) of the Muséum national d'Histoire naturelle (MNHN - Paris)	Gicim data base, Pruvost P. Causse R., 2009 http://doi.org/10.15468/tm7whu	This work is licensed under a Creative Commons Attribution (CC-BY) 4.0 License	OBIS (2019) [The fishes collection (IC) of the Muséum national d'Histoire naturelle (MNHN - Paris)] (Available: Ocean Biodiversity Information System. Intergovernmental Oceanographic Commission of UNESCO. https://obis.org. Accessed: 2019-08-29)
8a1ae661-e911-4967-bc06-1168fc5f2d89	iziko South African Museum - Fish Collection	iziko South African Museum - Fish Collection	Restricted	OBIS (2019) [iziko South African Museum - Fish Collection] (Available: Ocean Biodiversity Information System. Intergovernmental Oceanographic Commission of UNESCO. https://obis.org. Accessed: 2019-08-29)
9ff216fc-777e-4f9b-9860-95ed7366870d	Institution SAIAB - Collection SAIAB	No available dataset citation	Unspecified intellectual rights	OBIS (2019) [Institution SAIAB - Collection SAIAB] (Available: Ocean Biodiversity Information System. Intergovernmental Oceanographic Commission of UNESCO. https://obis.org. Accessed: 2019-08-29)
ff8b7809-41bc-40ad-8160-0e33862817a0	Biodiversity Research Museum, Academia Sinica, Taiwan	TELDAP, Biodiversity Research Museum, Academia Sinica, Taiwan (accessed through GBIF data portal, http://data.gbif.org/datasets/resource/9093, yyyy-mm-dd)	Unrestricted	OBIS (2019) [Biodiversity Research Museum, Academia Sinica, Taiwan] (Available: Ocean Biodiversity Information System. Intergovernmental Oceanographic Commission of UNESCO. https://obis.org. Accessed: 2019-08-29)

Gonichthys cocco

GBIF

GBIF Occurrence Download https://doi.org/10.15468/dd.ufmhzj Accessed from R via rgbif (https://github.com/ropensci/rgbif) on 2019-09-11

OBIS

OBIS (2019) Distribution records of Gonichthys cocco [Dataset] (Available: Ocean Biodiversity Information System. Intergovernmental Oceanographic Commission of UNESCO. www.obis.org. Accessed: 2019-08-29)

Dataset details:

Dataset_ID	Name	Citation	License	OBIS_citation

0332e1b5-5525-4301-9659-ef3da3e4e2b6	MARMAP Isaacs-Kidd Midwater Trawl 1990-2009	Marcel Reichert, 2010, MARMAP Isaacs-Kidd Midwater Trawl 1990-2009, SCDNR/NOAA MARMAP Program, SCDNR MARMAP Aggregate data surveys, The Marine Resources Monitoring, Assessment, and Prediction (MARMAP) Program, Marine Resources Research Institute, South Carolina Department of Natural Resources, P. O. Box 12559, Charleston SC 29422-2559, U.S.A.Retrieved from http://www.usgs.gov/obis-usa/	Restricted	OBIS (2019) [MARMAP Isaacs-Kidd Midwater Trawl 1990-2009] (Available: Ocean Biodiversity Information System. Intergovernmental Oceanographic Commission of UNESCO. https://obis.org. Accessed: 2019-08-29)
1f59030f-f116-4c34-915e-1882d819cda3	Institution Southampton Oceanography Ceter - Collection discovery	No available dataset citation	Unspecified intellectual rights	OBIS (2019) [Institution Southampton Oceanography Ceter - Collection discovery] (Available: Ocean Biodiversity Information System. Intergovernmental Oceanographic Commission of UNESCO. https://obis.org. Accessed: 2019-08-29)
2870c548-343e-4575-ac67-a4da35182c52	Institution Shirshov Institute - Collection SKAO	No available dataset citation	Unspecified intellectual rights	OBIS (2019) [Institution Shirshov Institute - Collection SKAO] (Available: Ocean Biodiversity Information System. Intergovernmental Oceanographic Commission of UNESCO. https://obis.org. Accessed: 2019-08-29)
5f2da252-6d49-4c9f-b3b3-1db53d75b345	MARMAP Bongo Nets 1990-2009	Marcel Reichert, 2010, MARMAP Bongo Nets 1990-2009, SCDNR/NOAA MARMAP Program, SCDNR MARMAP Aggregate data surveys, The Marine Resources Monitoring, Assessment, and Prediction (MARMAP) Program, Marine Resources Research Institute, South Carolina Department of Natural Resources, P. O. Box 12559, Charleston SC 29422-2559, U.S.A.Retrieved from http://www.usgs.gov/obis-usa/	Restricted	OBIS (2019) [MARMAP Bongo Nets 1990-2009] (Available: Ocean Biodiversity Information System. Intergovernmental Oceanographic Commission of UNESCO. https://obis.org. Accessed: 2019-08-29)

623f4f98-4e66-4bee-9e1c-17cb74cd2d21	Institution IEO - Collection MEDITS-Spain	No available dataset citation	Unspecified intellectual rights	OBIS (2019) [Institution IEO - Collection MEDITS-Spain] (Available: Ocean Biodiversity Information System. Intergovernmental Oceanographic Commission of UNESCO. https://obis.org. Accessed: 2019-08-29)
705770e5-3474-4e69-be8b-3107a0c5610a	The fishes collection (IC) of the Muséum national d'Histoire naturelle (MNHN - Paris)	Gicim data base, Pruvost P. Causse R., 2009 http://doi.org/10.15468/tm7whu	This work is licensed under a Creative Commons Attribution (CC-BY) 4.0 License	OBIS (2019) [The fishes collection (IC) of the Muséum national d'Histoire naturelle (MNHN - Paris)] (Available: Ocean Biodiversity Information System. Intergovernmental Oceanographic Commission of UNESCO. https://obis.org. Accessed: 2019-08-29)
784c3f00-9b0b-4b2d-a0e7-2de304537f8b	Institution TU - Collection Fish	No available dataset citation	Unspecified intellectual rights	OBIS (2019) [Institution TU - Collection Fish] (Available: Ocean Biodiversity Information System. Intergovernmental Oceanographic Commission of UNESCO. https://obis.org. Accessed: 2019-08-29)
8629ec33-be4b-4384-933f-a511fbc29967	MAR-ECO 2004	Wenneck, T. de Lange, Falkenhaug, T. and O.A. Bergstad. 2008. Strategies, methods, and technologies adopted on the RV G.O. Sars MAR-ECO expedition to the mid-Atlantic Ridge in 2004. Deep-sea Research II. 55: 6-28.	This work is licensed under a Creative Commons Attribution (CC-BY) 4.0 License	OBIS (2019) [MAR-ECO 2004] (Available: Ocean Biodiversity Information System. Intergovernmental Oceanographic Commission of UNESCO. https://obis.org. Accessed: 2019-08-29)
c24bf1c2-2c62-4056-a841-56d94e6e876a	Fish specimens	ROM Fish Collection (accessed through GBIF data portal, http://data.gbif.org/datasets/resource/660, 2012-01-20) http://doi.org/10.15468/syisbx	Unrestricted	OBIS (2019) [Fish specimens] (Available: Ocean Biodiversity Information System. Intergovernmental Oceanographic Commission of UNESCO. https://obis.org. Accessed: 2019-08-29)
cc8f28ce-e48d-4945-abfe-9d150a22dcd6	Hamburg pelagic fish database	Post, A. 1987. Pelagic transects of FRVs "Walther Herwig" and "Anton Dohrn" in the Atlantic Ocean 1966 to 1986. Mitt. Inst. f. Seefischerei d. BfaFi Hamburg, 42: 1-68.	This work is licensed under a Creative Commons Attribution (CC-BY) 4.0 License	OBIS (2019) [Hamburg pelagic fish database] (Available: Ocean Biodiversity Information System. Intergovernmental Oceanographic Commission of UNESCO. https://obis.org. Accessed: 2019-08-29)

ce1d93f3-8b0f-4ee7-9a4d-0393a6ec7fea	Atlantic Reference Centre Museum of Canadian Atlantic Organisms - Invertebrates and Fishes Data	Van Guelpen, L., 2016. Atlantic Reference Centre Museum of Canadian Atlantic Organisms - Invertebrates and Fishes Data. Version 4 In OBIS Canada Digital Collections. Bedford Institute of Oceanography, Dartmouth, NS, Canada. Published by OBIS, Digital http://www.iobis.org/. Accessed on –INSERT DATE	This work is licensed under a Creative Commons Attribution (CC-BY) 4.0 License	OBIS (2019) [Atlantic Reference Centre Museum of Canadian Atlantic Organisms - Invertebrates and Fishes Data] (Available: Ocean Biodiversity Information System. Intergovernmental Oceanographic Commission of UNESCO. https://obis.org. Accessed: 2019-08-29)
cfc56587-48c3-4e3d-9350-3a4d9a28b681	Institution NOAA, NMFS, Northeast Fisheries Science Center - Collection DEEPWATER SYSTEMATICS	No available dataset citation	Unspecified intellectual rights	OBIS (2019) [Institution NOAA, NMFS, Northeast Fisheries Science Center - Collection DEEPWATER SYSTEMATICS] (Available: Ocean Biodiversity Information System. Intergovernmental Oceanographic Commission of UNESCO. https://obis.org. Accessed: 2019-08-29)
d6d6fe4c-425f-4ce7-bf28-7a6befaeb413	National Museum of Natural History Vertebrate Zoology Fishes Collections	National Museum of Natural History, Smithsonian Institution NMNH Fishes Collection Database. National Museum of Natural History, Smithsonian Institution, 10th and Constitution Ave. N.W., Washington, DC 20560-0193, 2007.	This work is licensed under a Creative Commons Attribution (CC-BY) 4.0 License	OBIS (2019) [National Museum of Natural History Vertebrate Zoology Fishes Collections] (Available: Ocean Biodiversity Information System. Intergovernmental Oceanographic Commission of UNESCO. https://obis.org. Accessed: 2019-08-29)

Myctophum nitidulum

GBIF

GBIF Occurrence Download https://doi.org/10.15468/dd.tfgfqj Accessed from R via rgbif (https://github.com/ropensci/rgbif) on 2019-09-11

OBIS

OBIS (2019) Distribution records of Myctophum nitidulum [Dataset] (Available: Ocean Biodiversity Information System. Intergovernmental Oceanographic Commission of UNESCO. www.obis.org. Accessed: 2019-08-29)

Dataset details:

Dataset_ID	Name	Citation	License	OBIS_citation

0332e1b5-5525-4301-9659-ef3da3e4e2b6	MARMAP Isaacs-Kidd Midwater Trawl 1990-2009	Marcel Reichert, 2010, MARMAP Isaacs-Kidd Midwater Trawl 1990-2009, SCDNR/NOAA MARMAP Program, SCDNR MARMAP Aggregate data surveys, The Marine Resources Monitoring, Assessment, and Prediction (MARMAP) Program, Marine Resources Research Institute, South Carolina Department of Natural Resources, P. O. Box 12559, Charleston SC 29422-2559, U.S.A.Retrieved from http://www.usgs.gov/obis-usa/	Restricted	OBIS (2019) [MARMAP Isaacs-Kidd Midwater Trawl 1990-2009] (Available: Ocean Biodiversity Information System. Intergovernmental Oceanographic Commission of UNESCO. https://obis.org. Accessed: 2019-08-29)
04e3fd32-b08b-4806-a016-d2dff52ae55a	Asia-Pacific Dataset	Jintsu-Uchifune, Y., Yamamoto, H. (2016) Marine organism occurrence data of the Asia-Pacific region extracted from literature. Available at https://doi.org/10.48518/00002. Accessed on yyyy-mm-dd.	This work is licensed under a Creative Commons Attribution (CC-BY) 4.0 License	OBIS (2019) [Asia-Pacific Dataset] (Available: Ocean Biodiversity Information System. Intergovernmental Oceanographic Commission of UNESCO. https://obis.org. Accessed: 2019-08-29)
10b213e6-a9c4-459e-a40c-ef9edc461b97	Marine data from the Bernice P. Bishop Museum	Pyle R (2016). Bernice P. Bishop Museum. Version 8.1. Bernice Pauahi Bishop Museum. Occurrence dataset https://doi.org/10.15468/s6ctus accessed via GBIF.org on 2018-11-16.	To the extent possible under law, the publisher has waived all rights to these data and has dedicated them to the Public Domain (CC0 1.0)	OBIS (2019) [Marine data from the Bernice P. Bishop Museum] (Available: Ocean Biodiversity Information System. Intergovernmental Oceanographic Commission of UNESCO. https://obis.org. Accessed: 2019-08-29)
1f59030f-f116-4c34-915e-1882d819cda3	Institution Southampton Oceanography Ceter - Collection discovery	No available dataset citation	Unspecified intellectual rights	OBIS (2019) [Institution Southampton Oceanography Ceter - Collection discovery] (Available: Ocean Biodiversity Information System. Intergovernmental Oceanographic Commission of UNESCO. https://obis.org. Accessed: 2019-08-29)
270f3e70-ff9b-411d-b170-2bc914d83f26	Biological Reference Collections ICM CSIC	Olivas González F J (2016): Biological Reference Collections ICM CSIC. Institute of Marine Sciences (ICM-CSIC). https://dx.doi.org/10.15470/qlqqdx	This work is licensed under a Creative Commons Attribution Non Commercial (CC-BY-NC) 4.0 License	OBIS (2019) [Biological Reference Collections ICM CSIC] (Available: Ocean Biodiversity Information System. Intergovernmental Oceanographic Commission of UNESCO. https://obis.org. Accessed: 2019-08-29)

2870c548-343e-4575-ac67-a4da35182c52	Institution Shirshov Institute - Collection SKAO	No available dataset citation	Unspecified intellectual rights	OBIS (2019) [Institution Shirshov Institute - Collection SKAO] (Available: Ocean Biodiversity Information System. Intergovernmental Oceanographic Commission of UNESCO. https://obis.org. Accessed: 2019-08-29)
308a501c-a187-498e-a8bd-9cf3d2b70bd9	Institution REVIZEE - Collection Ictioplankton	No available dataset citation	Unspecified intellectual rights	OBIS (2019) [Institution REVIZEE - Collection Ictioplankton] (Available: Ocean Biodiversity Information System. Intergovernmental Oceanographic Commission of UNESCO. https://obis.org. Accessed: 2019-08-29)
513e4437-9cc7-4383-9a69-1bd18ec2046a	MARMAP Neuston Nets 1990-2009	Marcel Reichert, 2010, MARMAP Neuston Nets 1990-2009, SCDNR/NOAA MARMAP Program, SCDNR MARMAP Aggregate data surveys, The Marine Resources Monitoring, Assessment, and Prediction (MARMAP) Program, Marine Resources Research Institute, South Carolina Department of Natural Resources, P. O. Box 12559, Charleston SC 29422-2559, U.S.A.Retrieved from http://www.usgs.gov/obis-usa/	Restricted	OBIS (2019) [MARMAP Neuston Nets 1990-2009] (Available: Ocean Biodiversity Information System. Intergovernmental Oceanographic Commission of UNESCO. https://obis.org. Accessed: 2019-08-29)
5f2da252-6d49-4c9f-b3b3-1db53d75b345	MARMAP Bongo Nets 1990-2009	Marcel Reichert, 2010, MARMAP Bongo Nets 1990-2009, SCDNR/NOAA MARMAP Program, SCDNR MARMAP Aggregate data surveys, The Marine Resources Monitoring, Assessment, and Prediction (MARMAP) Program, Marine Resources Research Institute, South Carolina Department of Natural Resources, P. O. Box 12559, Charleston SC 29422-2559, U.S.A.Retrieved from http://www.usgs.gov/obis-usa/	Restricted	OBIS (2019) [MARMAP Bongo Nets 1990-2009] (Available: Ocean Biodiversity Information System. Intergovernmental Oceanographic Commission of UNESCO. https://obis.org. Accessed: 2019-08-29)

6a5bc28f-4dfe-4cbf-8a55-7e3a843997ab	SPC NECTALIS Zooplankton/Micronekton specimens, New Caledonia 2014	Allain, V., Menkes, C., 2014. Nectalis 3 cruise, RV Alis. https://doi.org/10.17600/14004900	This work is licensed under a Creative Commons Attribution Non Commercial (CC-BY-NC) 4.0 License	OBIS (2019) [SPC NECTALIS Zooplankton/Micronekton specimens, New Caledonia 2014] (Available: Ocean Biodiversity Information System. Intergovernmental Oceanographic Commission of UNESCO. https://obis.org. Accessed: 2019-08-29)
6c19184e-c305-4273-8890-6d342d86f865	Institution REVIZEE - Collection Pelagic Fishes	No available dataset citation	Unspecified intellectual rights	OBIS (2019) [Institution REVIZEE - Collection Pelagic Fishes] (Available: Ocean Biodiversity Information System. Intergovernmental Oceanographic Commission of UNESCO. https://obis.org. Accessed: 2019-08-29)
705770e5-3474-4e69-be8b-3107a0c5610a	The fishes collection (IC) of the Muséum national d'Histoire naturelle (MNHN - Paris)	Gicim data base, Pruvost P. Causse R., 2009 http://doi.org/10.15468/tm7whu	This work is licensed under a Creative Commons Attribution (CC-BY) 4.0 License	OBIS (2019) [The fishes collection (IC) of the Muséum national d'Histoire naturelle (MNHN - Paris)] (Available: Ocean Biodiversity Information System. Intergovernmental Oceanographic Commission of UNESCO. https://obis.org. Accessed: 2019-08-29)
784c3f00-9b0b-4b2d-a0e7-2de304537f8b	Institution TU - Collection Fish	No available dataset citation	Unspecified intellectual rights	OBIS (2019) [Institution TU - Collection Fish] (Available: Ocean Biodiversity Information System. Intergovernmental Oceanographic Commission of UNESCO. https://obis.org. Accessed: 2019-08-29)
87a421bf-4646-49e3-89b7-409b93f2ac7c	Institution UWFC - Collection ADULT COLLECTION	No available dataset citation	Unspecified intellectual rights	OBIS (2019) [Institution UWFC - Collection ADULT COLLECTION] (Available: Ocean Biodiversity Information System. Intergovernmental Oceanographic Commission of UNESCO. https://obis.org. Accessed: 2019-08-29)
8a1ae661-e911-4967-bc06-1168fc5f2d89	iziko South African Museum - Fish Collection	iziko South African Museum - Fish Collection	Restricted	OBIS (2019) [iziko South African Museum - Fish Collection] (Available: Ocean Biodiversity Information System. Intergovernmental Oceanographic Commission of UNESCO. https://obis.org. Accessed: 2019-08-29)

b8617377-eb1c-4db2-baa6-8788a632e810	Ichthyology Collection - Royal Ontario Museum	NA	This work is licensed under a Creative Commons Attribution Non Commercial (CC-BY-NC) 4.0 License	OBIS (2019) [Ichthyology Collection - Royal Ontario Museum] (Available: Ocean Biodiversity Information System. Intergovernmental Oceanographic Commission of UNESCO. https://obis.org. Accessed: 2019-08-29)
c24bf1c2-2c62-4056-a841-56d94e6e876a	Fish specimens	ROM Fish Collection (accessed through GBIF data portal, http://data.gbif.org/datasets/resource/660, 2012-01-20) http://doi.org/10.15468/syisbx	Unrestricted	OBIS (2019) [Fish specimens] (Available: Ocean Biodiversity Information System. Intergovernmental Oceanographic Commission of UNESCO. https://obis.org. Accessed: 2019-08-29)
cc8f28ce-e48d-4945-abfe-9d150a22dcd6	Hamburg pelagic fish database	Post, A. 1987. Pelagic transects of FRVs "Walther Herwig" and "Anton Dohrn" in the Atlantic Ocean 1966 to 1986. Mitt. Inst. f. Seefischerei d. BfaFi Hamburg, 42: 1-68.	This work is licensed under a Creative Commons Attribution (CC-BY) 4.0 License	OBIS (2019) [Hamburg pelagic fish database] (Available: Ocean Biodiversity Information System. Intergovernmental Oceanographic Commission of UNESCO. https://obis.org. Accessed: 2019-08-29)
ce1d93f3-8b0f-4ee7-9a4d-0393a6ec7fea	Atlantic Reference Centre Museum of Canadian Atlantic Organisms - Invertebrates and Fishes Data	Van Guelpen, L., 2016. Atlantic Reference Centre Museum of Canadian Atlantic Organisms - Invertebrates and Fishes Data. Version 4 In OBIS Canada Digital Collections. Bedford Institute of Oceanography, Dartmouth, NS, Canada. Published by OBIS, Digital http://www.iobis.org/. Accessed on –INSERT DATE	This work is licensed under a Creative Commons Attribution (CC-BY) 4.0 License	OBIS (2019) [Atlantic Reference Centre Museum of Canadian Atlantic Organisms - Invertebrates and Fishes Data] (Available: Ocean Biodiversity Information System. Intergovernmental Oceanographic Commission of UNESCO. https://obis.org. Accessed: 2019-08-29)
cfc56587-48c3-4e3d-9350-3a4d9a28b681	Institution NOAA, NMFS, Northeast Fisheries Science Center - Collection DEEPWATER SYSTEMATICS	No available dataset citation	Unspecified intellectual rights	OBIS (2019) [Institution NOAA, NMFS, Northeast Fisheries Science Center - Collection DEEPWATER SYSTEMATICS] (Available: Ocean Biodiversity Information System. Intergovernmental Oceanographic Commission of UNESCO. https://obis.org. Accessed: 2019-08-29)
d6d6fe4c-425f-4ce7-bf28-7a6befaeb413	National Museum of Natural History Vertebrate Zoology Fishes Collections	National Museum of Natural History, Smithsonian Institution NMNH Fishes Collection Database. National Museum of Natural History, Smithsonian Institution, 10th and Constitution Ave. N.W., Washington, DC 20560-0193, 2007.	This work is licensed under a Creative Commons Attribution (CC-BY) 4.0 License	OBIS (2019) [National Museum of Natural History Vertebrate Zoology Fishes Collections] (Available: Ocean Biodiversity Information System. Intergovernmental Oceanographic Commission of UNESCO. https://obis.org. Accessed: 2019-08-29)

f1da0955-5ece-4f98-ab77-f10c20bdd3ca	Institution OGL - Collection OGR	No available dataset citation	Unspecified intellectual rights	OBIS (2019) [Institution OGL - Collection OGR] (Available: Ocean Biodiversity Information System. Intergovernmental Oceanographic Commission of UNESCO. https://obis.org. Accessed: 2019-08-29)
ff8b7809-41bc-40ad-8160-0e33862817a0	Biodiversity Research Museum, Academia Sinica, Taiwan	TELDAP, Biodiversity Research Museum, Academia Sinica, Taiwan (accessed through GBIF data portal, http://data.gbif.org/datasets/resource/9093, yyyy-mm-dd)	Unrestricted	OBIS (2019) [Biodiversity Research Museum, Academia Sinica, Taiwan] (Available: Ocean Biodiversity Information System. Intergovernmental Oceanographic Commission of UNESCO. https://obis.org. Accessed: 2019-08-29)

Myctophum affine

GBIF

GBIF Occurrence Download https://doi.org/10.15468/dd.dv6q5a Accessed from R via rgbif (https://github.com/ropensci/rgbif) on 2019-09-11

OBIS

OBIS (2019) Distribution records of Myctophum affine [Dataset] (Available: Ocean Biodiversity Information System. Intergovernmental Oceanographic Commission of UNESCO. www.obis.org. Accessed: 2019-08-29)

Dataset details:

Dataset_ID	Name	Citation	License	OBIS_citation
2870c548-343e-4575-ac67-a4da35182c52	Institution Shirshov Institute - Collection SKAO	No available dataset citation	Unspecified intellectual rights	OBIS (2019) [Institution Shirshov Institute - Collection SKAO] (Available: Ocean Biodiversity Information System. Intergovernmental Oceanographic Commission of UNESCO. https://obis.org. Accessed: 2019-08-29)
308a501c-a187-498e-a8bd-9cf3d2b70bd9	Institution REVIZEE - Collection Ictioplankton	No available dataset citation	Unspecified intellectual rights	OBIS (2019) [Institution REVIZEE - Collection Ictioplankton] (Available: Ocean Biodiversity Information System. Intergovernmental Oceanographic Commission of UNESCO. https://obis.org. Accessed: 2019-08-29)

6c19184e-c305-4273-8890-6d342d86f865	Institution REVIZEE - Collection Pelagic Fishes	No available dataset citation	Unspecified intellectual rights	OBIS (2019) [Institution REVIZEE - Collection Pelagic Fishes] (Available: Ocean Biodiversity Information System. Intergovernmental Oceanographic Commission of UNESCO. https://obis.org. Accessed: 2019-08-29)
705770e5-3474-4e69-be8b-3107a0c5610a	The fishes collection (IC) of the Muséum national d'Histoire naturelle (MNHN - Paris)	Gicim data base, Pruvost P. Causse R., 2009 http://doi.org/10.15468/tm7whu	This work is licensed under a Creative Commons Attribution (CC-BY) 4.0 License	OBIS (2019) [The fishes collection (IC) of the Muséum national d'Histoire naturelle (MNHN - Paris)] (Available: Ocean Biodiversity Information System. Intergovernmental Oceanographic Commission of UNESCO. https://obis.org. Accessed: 2019-08-29)
784c3f00-9b0b-4b2d-a0e7-2de304537f8b	Institution TU - Collection Fish	No available dataset citation	Unspecified intellectual rights	OBIS (2019) [Institution TU - Collection Fish] (Available: Ocean Biodiversity Information System. Intergovernmental Oceanographic Commission of UNESCO. https://obis.org. Accessed: 2019-08-29)
b8617377-eb1c-4db2-baa6-8788a632e810	Ichthyology Collection - Royal Ontario Museum	NA	This work is licensed under a Creative Commons Attribution Non Commercial (CC-BY-NC) 4.0 License	OBIS (2019) [Ichthyology Collection - Royal Ontario Museum] (Available: Ocean Biodiversity Information System. Intergovernmental Oceanographic Commission of UNESCO. https://obis.org. Accessed: 2019-08-29)
c24bf1c2-2c62-4056-a841-56d94e6e876a	Fish specimens	ROM Fish Collection (accessed through GBIF data portal, http://data.gbif.org/datasets/resource/660, 2012-01-20) http://doi.org/10.15468/syisbx	Unrestricted	OBIS (2019) [Fish specimens] (Available: Ocean Biodiversity Information System. Intergovernmental Oceanographic Commission of UNESCO. https://obis.org. Accessed: 2019-08-29)
ce1d93f3-8b0f-4ee7-9a4d-0393a6ec7fea	Atlantic Reference Centre Museum of Canadian Atlantic Organisms - Invertebrates and Fishes Data	Van Guelpen, L., 2016. Atlantic Reference Centre Museum of Canadian Atlantic Organisms - Invertebrates and Fishes Data. Version 4 In OBIS Canada Digital Collections. Bedford Institute of Oceanography, Dartmouth, NS, Canada. Published by OBIS, Digital http://www.iobis.org/. Accessed on –INSERT DATE	This work is licensed under a Creative Commons Attribution (CC-BY) 4.0 License	OBIS (2019) [Atlantic Reference Centre Museum of Canadian Atlantic Organisms - Invertebrates and Fishes Data] (Available: Ocean Biodiversity Information System. Intergovernmental Oceanographic Commission of UNESCO. https://obis.org. Accessed: 2019-08-29)

cfc56587-48c3-4e3d-9350-3a4d9a28b681	Institution NOAA, NMFS, Northeast Fisheries Science Center - Collection DEEPWATER SYSTEMATICS	No available dataset citation	Unspecified intellectual rights	OBIS (2019) [Institution NOAA, NMFS, Northeast Fisheries Science Center - Collection DEEPWATER SYSTEMATICS] (Available: Ocean Biodiversity Information System. Intergovernmental Oceanographic Commission of UNESCO. https://obis.org. Accessed: 2019-08-29)
d6d6fe4c-425f-4ce7-bf28-7a6befaeb413	National Museum of Natural History Vertebrate Zoology Fishes Collections	National Museum of Natural History, Smithsonian Institution NMNH Fishes Collection Database. National Museum of Natural History, Smithsonian Institution, 10th and Constitution Ave. N.W., Washington, DC 20560-0193, 2007.	This work is licensed under a Creative Commons Attribution (CC-BY) 4.0 License	OBIS (2019) [National Museum of Natural History Vertebrate Zoology Fishes Collections] (Available: Ocean Biodiversity Information System. Intergovernmental Oceanographic Commission of UNESCO. https://obis.org. Accessed: 2019-08-29)

Triphoturus nigrescens

GBIF

GBIF Occurrence Download https://doi.org/10.15468/dd.hsaw9e Accessed from R via rgbif (https://github.com/ropensci/rgbif) on 2019-09-11

OBIS

OBIS (2019) Distribution records of Triphoturus nigrescens [Dataset] (Available: Ocean Biodiversity Information System. Intergovernmental Oceanographic Commission of UNESCO. www.obis.org. Accessed: 2019-08-29)

Dataset details:

Dataset_ID	Name	Citation	License	OBIS_citation
04e3fd32-b08b-4806-a016-d2dff52ae55a	Asia-Pacific Dataset	Jintsu-Uchifune, Y., Yamamoto, H. (2016) Marine organism occurrence data of the Asia-Pacific region extracted from literature. Available at https://doi.org/10.48518/00002. Accessed on yyyy-mm-dd.	This work is licensed under a Creative Commons Attribution (CC-BY) 4.0 License	OBIS (2019) [Asia-Pacific Dataset] (Available: Ocean Biodiversity Information System. Intergovernmental Oceanographic Commission of UNESCO. https://obis.org. Accessed: 2019-08-29)

270f3e70-ff9b-411d-b170-2bc914d83f26	Biological Reference Collections ICM CSIC	Olivas González F J (2016): Biological Reference Collections ICM CSIC. Institute of Marine Sciences (ICM-CSIC). https://dx.doi.org/10.15470/qlqqdx	This work is licensed under a Creative Commons Attribution Non Commercial (CC-BY-NC) 4.0 License	OBIS (2019) [Biological Reference Collections ICM CSIC] (Available: Ocean Biodiversity Information System. Intergovernmental Oceanographic Commission of UNESCO. https://obis.org. Accessed: 2019-08-29)
705770e5-3474-4e69-be8b-3107a0c5610a	The fishes collection (IC) of the Muséum national d'Histoire naturelle (MNHN - Paris)	Gicim data base, Pruvost P. Causse R., 2009 http://doi.org/10.15468/tm7whu	This work is licensed under a Creative Commons Attribution (CC-BY) 4.0 License	OBIS (2019) [The fishes collection (IC) of the Muséum national d'Histoire naturelle (MNHN - Paris)] (Available: Ocean Biodiversity Information System. Intergovernmental Oceanographic Commission of UNESCO. https://obis.org. Accessed: 2019-08-29)
8a1ae661-e911-4967-bc06-1168fc5f2d89	iziko South African Museum - Fish Collection	iziko South African Museum - Fish Collection	Restricted	OBIS (2019) [iziko South African Museum - Fish Collection] (Available: Ocean Biodiversity Information System. Intergovernmental Oceanographic Commission of UNESCO. https://obis.org. Accessed: 2019-08-29)
d6d6fe4c-425f-4ce7-bf28-7a6befaeb413	National Museum of Natural History Vertebrate Zoology Fishes Collections	National Museum of Natural History, Smithsonian Institution NMNH Fishes Collection Database. National Museum of Natural History, Smithsonian Institution, 10th and Constitution Ave. N.W., Washington, DC 20560-0193, 2007.	This work is licensed under a Creative Commons Attribution (CC-BY) 4.0 License	OBIS (2019) [National Museum of Natural History Vertebrate Zoology Fishes Collections] (Available: Ocean Biodiversity Information System. Intergovernmental Oceanographic Commission of UNESCO. https://obis.org. Accessed: 2019-08-29)

Centrobranchus nigroocellatus

GBIF

GBIF Occurrence Download https://doi.org/10.15468/dd.g8bc9t Accessed from R via rgbif (https://github.com/ropensci/rgbif) on 2019-09-11

OBIS

OBIS (2019) Distribution records of Centrobranchus nigroocellatus [Dataset] (Available: Ocean Biodiversity Information System. Intergovernmental Oceanographic Commission of UNESCO. www.obis.org. Accessed: 2019-08-29)

Dataset details:

Dataset_ID	Name	Citation	License	OBIS_citation
0332e1b5-5525-4301-9659-ef3da3e4e2b6	MARMAP Isaacs-Kidd Midwater Trawl 1990-2009	Marcel Reichert, 2010, MARMAP Isaacs-Kidd Midwater Trawl 1990-2009, SCDNR/NOAA MARMAP Program, SCDNR MARMAP Aggregate data surveys, The Marine Resources Monitoring, Assessment, and Prediction (MARMAP) Program, Marine Resources Research Institute, South Carolina Department of Natural Resources, P. O. Box 12559, Charleston SC 29422-2559, U.S.A.Retrieved from http://www.usgs.gov/obis-usa/	Restricted	OBIS (2019) [MARMAP Isaacs-Kidd Midwater Trawl 1990-2009] (Available: Ocean Biodiversity Information System. Intergovernmental Oceanographic Commission of UNESCO. https://obis.org. Accessed: 2019-08-29)
04e3fd32-b08b-4806-a016-d2dff52ae55a	Asia-Pacific Dataset	Jintsu-Uchifune, Y., Yamamoto, H. (2016) Marine organism occurrence data of the Asia-Pacific region extracted from literature. Available at https://doi.org/10.48518/00002. Accessed on yyyy-mm-dd.	This work is licensed under a Creative Commons Attribution (CC-BY) 4.0 License	OBIS (2019) [Asia-Pacific Dataset] (Available: Ocean Biodiversity Information System. Intergovernmental Oceanographic Commission of UNESCO. https://obis.org. Accessed: 2019-08-29)
10b213e6-a9c4-459e-a40c-ef9edc461b97	Marine data from the Bernice P. Bishop Museum	Pyle R (2016). Bernice P. Bishop Museum. Version 8.1. Bernice Pauahi Bishop Museum. Occurrence dataset https://doi.org/10.15468/s6ctus accessed via GBIF.org on 2018-11-16.	To the extent possible under law, the publisher has waived all rights to these data and has dedicated them to the Public Domain (CC0 1.0)	OBIS (2019) [Marine data from the Bernice P. Bishop Museum] (Available: Ocean Biodiversity Information System. Intergovernmental Oceanographic Commission of UNESCO. https://obis.org. Accessed: 2019-08-29)
2870c548-343e-4575-ac67-a4da35182c52	Institution Shirshov Institute - Collection SKAO	No available dataset citation	Unspecified intellectual rights	OBIS (2019) [Institution Shirshov Institute - Collection SKAO] (Available: Ocean Biodiversity Information System. Intergovernmental Oceanographic Commission of UNESCO. https://obis.org. Accessed: 2019-08-29)
308a501c-a187-498e-a8bd-9cf3d2b70bd9	Institution REVIZEE - Collection Ictioplankton	No available dataset citation	Unspecified intellectual rights	OBIS (2019) [Institution REVIZEE - Collection Ictioplankton] (Available: Ocean Biodiversity Information System. Intergovernmental Oceanographic Commission of UNESCO. https://obis.org. Accessed: 2019-08-29)

513e4437-9cc7-4383-9a69-1bd18ec2046a	MARMAP Neuston Nets 1990-2009	Marcel Reichert, 2010, MARMAP Neuston Nets 1990-2009, SCDNR/NOAA MARMAP Program, SCDNR MARMAP Aggregate data surveys, The Marine Resources Monitoring, Assessment, and Prediction (MARMAP) Program, Marine Resources Research Institute, South Carolina Department of Natural Resources, P. O. Box 12559, Charleston SC 29422-2559, U.S.A.Retrieved from http://www.usgs.gov/obis-usa/	Restricted	OBIS (2019) [MARMAP Neuston Nets 1990-2009] (Available: Ocean Biodiversity Information System. Intergovernmental Oceanographic Commission of UNESCO. https://obis.org. Accessed: 2019-08-29)
6a5bc28f-4dfe-4cbf-8a55-7e3a843997ab	SPC NECTALIS Zooplankton/Micronekton specimens, New Caledonia 2014	Allain, V., Menkes, C., 2014. Nectalis 3 cruise, RV Alis. https://doi.org/10.17600/14004900	This work is licensed under a Creative Commons Attribution Non Commercial (CC-BY-NC) 4.0 License	OBIS (2019) [SPC NECTALIS Zooplankton/Micronekton specimens, New Caledonia 2014] (Available: Ocean Biodiversity Information System. Intergovernmental Oceanographic Commission of UNESCO. https://obis.org. Accessed: 2019-08-29)
705770e5-3474-4e69-be8b-3107a0c5610a	The fishes collection (IC) of the Muséum national d'Histoire naturelle (MNHN - Paris)	Gicim data base, Pruvost P. Causse R., 2009 http://doi.org/10.15468/tm7whu	This work is licensed under a Creative Commons Attribution (CC-BY) 4.0 License	OBIS (2019) [The fishes collection (IC) of the Muséum national d'Histoire naturelle (MNHN - Paris)] (Available: Ocean Biodiversity Information System. Intergovernmental Oceanographic Commission of UNESCO. https://obis.org. Accessed: 2019-08-29)
784c3f00-9b0b-4b2d-a0e7-2de304537f8b	Institution TU - Collection Fish	No available dataset citation	Unspecified intellectual rights	OBIS (2019) [Institution TU - Collection Fish] (Available: Ocean Biodiversity Information System. Intergovernmental Oceanographic Commission of UNESCO. https://obis.org. Accessed: 2019-08-29)
b8617377-eb1c-4db2-baa6-8788a632e810	Ichthyology Collection - Royal Ontario Museum	NA	This work is licensed under a Creative Commons Attribution Non Commercial (CC-BY-NC) 4.0 License	OBIS (2019) [Ichthyology Collection - Royal Ontario Museum] (Available: Ocean Biodiversity Information System. Intergovernmental Oceanographic Commission of UNESCO. https://obis.org. Accessed: 2019-08-29)

c24bf1c2-2c62-4056-a841-56d94e6e876a	Fish specimens	ROM Fish Collection (accessed through GBIF data portal, http://data.gbif.org/datasets/resource/660, 2012-01-20) http://doi.org/10.15468/syisbx	Unrestricted	OBIS (2019) [Fish specimens] (Available: Ocean Biodiversity Information System. Intergovernmental Oceanographic Commission of UNESCO. https://obis.org. Accessed: 2019-08-29)
cc8f28ce-e48d-4945-abfe-9d150a22dcd6	Hamburg pelagic fish database	Post, A. 1987. Pelagic transects of FRVs "Walther Herwig" and "Anton Dohrn" in the Atlantic Ocean 1966 to 1986. Mitt. Inst. f. Seefischerei d. BfaFi Hamburg, 42: 1-68.	This work is licensed under a Creative Commons Attribution (CC-BY) 4.0 License	OBIS (2019) [Hamburg pelagic fish database] (Available: Ocean Biodiversity Information System. Intergovernmental Oceanographic Commission of UNESCO. https://obis.org. Accessed: 2019-08-29)
cfc56587-48c3-4e3d-9350-3a4d9a28b681	Institution NOAA, NMFS, Northeast Fisheries Science Center - Collection DEEPWATER SYSTEMATICS	No available dataset citation	Unspecified intellectual rights	OBIS (2019) [Institution NOAA, NMFS, Northeast Fisheries Science Center - Collection DEEPWATER SYSTEMATICS] (Available: Ocean Biodiversity Information System. Intergovernmental Oceanographic Commission of UNESCO. https://obis.org. Accessed: 2019-08-29)
d6d6fe4c-425f-4ce7-bf28-7a6befaeb413	National Museum of Natural History Vertebrate Zoology Fishes Collections	National Museum of Natural History, Smithsonian Institution NMNH Fishes Collection Database. National Museum of Natural History, Smithsonian Institution, 10th and Constitution Ave. N.W., Washington, DC 20560-0193, 2007.	This work is licensed under a Creative Commons Attribution (CC-BY) 4.0 License	OBIS (2019) [National Museum of Natural History Vertebrate Zoology Fishes Collections] (Available: Ocean Biodiversity Information System. Intergovernmental Oceanographic Commission of UNESCO. https://obis.org. Accessed: 2019-08-29)

Lampanyctodes hectoris

GBIF

GBIF Occurrence Download https://doi.org/10.15468/dd.66t25q Accessed from R via rgbif (https://github.com/ropensci/rgbif) on 2019-09-11

OBIS

OBIS (2019) Distribution records of Lampanyctodes hectoris [Dataset] (Available: Ocean Biodiversity Information System. Intergovernmental Oceanographic Commission of UNESCO. www.obis.org. Accessed: 2019-08-29)

Dataset details:

Dataset_ID	Name	Citation	License	OBIS_citation
0c9db499-759b-46d8-8989-799f9ff9f235	Auckland Museum NZ Marine Collection	Blom W, Moriarty A (2018). Auckland Museum NZ Marine Collection. Version 1.11. Auckland War Memorial Museum. Occurrence Dataset https://doi.org/10.15468/plyefd accessed via GBIF.org on 2018-01-15.	This work is licensed under a Creative Commons Attribution (CC-BY) 4.0 License	OBIS (2019) [Auckland Museum NZ Marine Collection] (Available: Ocean Biodiversity Information System. Intergovernmental Oceanographic Commission of UNESCO. https://obis.org. Accessed: 2019-08-29)
231dc661-d8d9-4d49-9ff0-45bd21a08541	New Zealand fish and squid distributions from research bottom trawls 1964-2008	NIWA (2014). New Zealand fish and squid distributions from research bottom trawls. Southwestern Pacific OBIS, National Institute of Water and Atmospheric Research (NIWA), Wellington, New Zealand, 486781 records, Online http://nzobisipt.niwa.co.nz/resource.do?r=obisprovider released on May 8, 2014.	This work is licensed under a Creative Commons Attribution (CC-BY) 4.0 License	OBIS (2019) [New Zealand fish and squid distributions from research bottom trawls 1964-2008] (Available: Ocean Biodiversity Information System. Intergovernmental Oceanographic Commission of UNESCO. https://obis.org. Accessed: 2019-08-29)
270f3e70-ff9b-411d-b170-2bc914d83f26	Biological Reference Collections ICM CSIC	Olivas González F J (2016): Biological Reference Collections ICM CSIC. Institute of Marine Sciences (ICM-CSIC). https://dx.doi.org/10.15470/qlqqdx	This work is licensed under a Creative Commons Attribution Non Commercial (CC-BY-NC) 4.0 License	OBIS (2019) [Biological Reference Collections ICM CSIC] (Available: Ocean Biodiversity Information System. Intergovernmental Oceanographic Commission of UNESCO. https://obis.org. Accessed: 2019-08-29)
4bdc1f6f-e16a-48b4-b995-b51bd41caa8d	Dataset of the multidisciplinary research surveys in the seamounts of Ewing and Valdivia Bank (Walvis Ridge) - SE Atlantic	López-Abellán, L. J.; Sarralde Vizuete, R.; González Jiménez, J. F.; Centro Oceanográfico de Canarias – IEO, Spain (2015). Dataset of the multidisciplinary research surveys in the seamounts of Ewing and Valdivia Bank (Walvis Ridge) - SE Atlantic https://dx.doi.org/10.14284/58	Attribution-NoDerivatives (CC BY-ND)	OBIS (2019) [Dataset of the multidisciplinary research surveys in the seamounts of Ewing and Valdivia Bank (Walvis Ridge) - SE Atlantic] (Available: Ocean Biodiversity Information System. Intergovernmental Oceanographic Commission of UNESCO. https://obis.org. Accessed: 2019-08-29)
50903a57-ee9f-4367-b2cd-0b36dcf4a6ad	Catch data from New Zealand research trawls since 2008	SWPRON (2017). Catch data from New Zealand research trawls. Southwestern Pacific OBIS, National Institute of Water and Atmospheric Research (NIWA), Wellington, New Zealand, 15157 records, Online http://nzobisipt.niwa.co.nz/resource.do?r=trawl released on April 19, 2017.	This work is licensed under a Creative Commons Attribution (CC-BY) 4.0 License	OBIS (2019) [Catch data from New Zealand research trawls since 2008] (Available: Ocean Biodiversity Information System. Intergovernmental Oceanographic Commission of UNESCO. https://obis.org. Accessed: 2019-08-29)

685b3956-c37a-433a-b661-2bb7b11cf9f8	Soviet Trawl Fishery Data (New Zealand Waters) 1964-1987	Ministry for Primary Industries (2014). Soviet Fishery Data (New Zealand Waters) 1964-1987. Southwestern Pacific OBIS, National Institute of Water and Atmospheric Research (NIWA), Wellington, New Zealand, 111883 records, Online http://nzobisipt.niwa.co.nz/resource.do?r=mbis_soviettrawl released on November 5, 2014.	This work is licensed under a Creative Commons Attribution (CC-BY) 4.0 License	OBIS (2019) [Soviet Trawl Fishery Data (New Zealand Waters) 1964-1987] (Available: Ocean Biodiversity Information System. Intergovernmental Oceanographic Commission of UNESCO. https://obis.org. Accessed: 2019-08-29)
705770e5-3474-4e69-be8b-3107a0c5610a	The fishes collection (IC) of the Muséum national d'Histoire naturelle (MNHN - Paris)	Gicim data base, Pruvost P. Causse R., 2009 http://doi.org/10.15468/tm7whu	This work is licensed under a Creative Commons Attribution (CC-BY) 4.0 License	OBIS (2019) [The fishes collection (IC) of the Muséum national d'Histoire naturelle (MNHN - Paris)] (Available: Ocean Biodiversity Information System. Intergovernmental Oceanographic Commission of UNESCO. https://obis.org. Accessed: 2019-08-29)
8a1ae661-e911-4967-bc06-1168fc5f2d89	iziko South African Museum - Fish Collection	iziko South African Museum - Fish Collection	Restricted	OBIS (2019) [iziko South African Museum - Fish Collection] (Available: Ocean Biodiversity Information System. Intergovernmental Oceanographic Commission of UNESCO. https://obis.org. Accessed: 2019-08-29)
9ff216fc-777e-4f9b-9860-95ed7366870d	Institution SAIAB - Collection SAIAB	No available dataset citation	Unspecified intellectual rights	OBIS (2019) [Institution SAIAB - Collection SAIAB] (Available: Ocean Biodiversity Information System. Intergovernmental Oceanographic Commission of UNESCO. https://obis.org. Accessed: 2019-08-29)
a4f7ee48-0d0b-4c05-a972-27a43b30db58	Institution MCM - Collection DEM	No available dataset citation	Unspecified intellectual rights	OBIS (2019) [Institution MCM - Collection DEM] (Available: Ocean Biodiversity Information System. Intergovernmental Oceanographic Commission of UNESCO. https://obis.org. Accessed: 2019-08-29)
d6d6fe4c-425f-4ce7-bf28-7a6befaeb413	National Museum of Natural History Vertebrate Zoology Fishes Collections	National Museum of Natural History, Smithsonian Institution NMNH Fishes Collection Database. National Museum of Natural History, Smithsonian Institution, 10th and Constitution Ave. N.W., Washington, DC 20560-0193, 2007.	This work is licensed under a Creative Commons Attribution (CC-BY) 4.0 License	OBIS (2019) [National Museum of Natural History Vertebrate Zoology Fishes Collections] (Available: Ocean Biodiversity Information System. Intergovernmental Oceanographic Commission of UNESCO. https://obis.org. Accessed: 2019-08-29)

Myctophum selenops

GBIF

GBIF Occurrence Download https://doi.org/10.15468/dd.urppe4 Accessed from R via rgbif (https://github.com/ropensci/rgbif) on 2019-09-11

OBIS

OBIS (2019) Distribution records of Myctophum selenops [Dataset] (Available: Ocean Biodiversity Information System. Intergovernmental Oceanographic Commission of UNESCO. www.obis.org. Accessed: 2019-08-29)

Dataset details:

Dataset_ID	Name	Citation	License	OBIS_citation
0332e1b5-5525-4301-9659-ef3da3e4e2b6	MARMAP Isaacs-Kidd Midwater Trawl 1990-2009	Marcel Reichert, 2010, MARMAP Isaacs-Kidd Midwater Trawl 1990-2009, SCDNR/NOAA MARMAP Program, SCDNR MARMAP Aggregate data surveys, The Marine Resources Monitoring, Assessment, and Prediction (MARMAP) Program, Marine Resources Research Institute, South Carolina Department of Natural Resources, P. O. Box 12559, Charleston SC 29422-2559, U.S.A.Retrieved from http://www.usgs.gov/obis-usa/	Restricted	OBIS (2019) [MARMAP Isaacs-Kidd Midwater Trawl 1990-2009] (Available: Ocean Biodiversity Information System. Intergovernmental Oceanographic Commission of UNESCO. https://obis.org. Accessed: 2019-08-29)
10b213e6-a9c4-459e-a40c-ef9edc461b97	Marine data from the Bernice P. Bishop Museum	Pyle R (2016). Bernice P. Bishop Museum. Version 8.1. Bernice Pauahi Bishop Museum. Occurrence dataset https://doi.org/10.15468/s6ctus accessed via GBIF.org on 2018-11-16.	To the extent possible under law, the publisher has waived all rights to these data and has dedicated them to the Public Domain (CC0 1.0)	OBIS (2019) [Marine data from the Bernice P. Bishop Museum] (Available: Ocean Biodiversity Information System. Intergovernmental Oceanographic Commission of UNESCO. https://obis.org. Accessed: 2019-08-29)
1f59030f-f116-4c34-915e-1882d819cda3	Institution Southampton Oceanography Ceter - Collection discovery	No available dataset citation	Unspecified intellectual rights	OBIS (2019) [Institution Southampton Oceanography Ceter - Collection discovery] (Available: Ocean Biodiversity Information System. Intergovernmental Oceanographic Commission of UNESCO. https://obis.org. Accessed: 2019-08-29)

270f3e70-ff9b-411d-b170-2bc914d83f26	Biological Reference Collections ICM CSIC	Olivas González F J (2016): Biological Reference Collections ICM CSIC. Institute of Marine Sciences (ICM-CSIC). https://dx.doi.org/10.15470/qlqqdx	This work is licensed under a Creative Commons Attribution Non Commercial (CC-BY-NC) 4.0 License	OBIS (2019) [Biological Reference Collections ICM CSIC] (Available: Ocean Biodiversity Information System. Intergovernmental Oceanographic Commission of UNESCO. https://obis.org. Accessed: 2019-08-29)
2870c548-343e-4575-ac67-a4da35182c52	Institution Shirshov Institute - Collection SKAO	No available dataset citation	Unspecified intellectual rights	OBIS (2019) [Institution Shirshov Institute - Collection SKAO] (Available: Ocean Biodiversity Information System. Intergovernmental Oceanographic Commission of UNESCO. https://obis.org. Accessed: 2019-08-29)
308a501c-a187-498e-a8bd-9cf3d2b70bd9	Institution REVIZEE - Collection Ictioplankton	No available dataset citation	Unspecified intellectual rights	OBIS (2019) [Institution REVIZEE - Collection Ictioplankton] (Available: Ocean Biodiversity Information System. Intergovernmental Oceanographic Commission of UNESCO. https://obis.org. Accessed: 2019-08-29)
4354345d-7faf-4376-b326-ffbc04b6b0cd	No available dataset name	No available dataset citation	Unspecified intellectual rights	OBIS (2019) [No available dataset name] (Available: Ocean Biodiversity Information System. Intergovernmental Oceanographic Commission of UNESCO. https://obis.org. Accessed: 2019-08-29)
5f2da252-6d49-4c9f-b3b3-1db53d75b345	MARMAP Bongo Nets 1990-2009	Marcel Reichert, 2010, MARMAP Bongo Nets 1990-2009, SCDNR/NOAA MARMAP Program, SCDNR MARMAP Aggregate data surveys, The Marine Resources Monitoring, Assessment, and Prediction (MARMAP) Program, Marine Resources Research Institute, South Carolina Department of Natural Resources, P. O. Box 12559, Charleston SC 29422-2559, U.S.A.Retrieved from http://www.usgs.gov/obis-usa/	Restricted	OBIS (2019) [MARMAP Bongo Nets 1990-2009] (Available: Ocean Biodiversity Information System. Intergovernmental Oceanographic Commission of UNESCO. https://obis.org. Accessed: 2019-08-29)

6a5bc28f-4dfe-4cbf-8a55-7e3a843997ab	SPC NECTALIS Zooplankton/Micronekton specimens, New Caledonia 2014	Allain, V., Menkes, C., 2014. Nectalis 3 cruise, RV Alis. https://doi.org/10.17600/14004900	This work is licensed under a Creative Commons Attribution Non Commercial (CC-BY-NC) 4.0 License	OBIS (2019) [SPC NECTALIS Zooplankton/Micronekton specimens, New Caledonia 2014] (Available: Ocean Biodiversity Information System. Intergovernmental Oceanographic Commission of UNESCO. https://obis.org. Accessed: 2019-08-29)
6c19184e-c305-4273-8890-6d342d86f865	Institution - Collection	No available dataset citation	Unspecified intellectual rights	OBIS (2019) [Institution - Collection] (Available: Ocean Biodiversity Information System. Intergovernmental Oceanographic Commission of UNESCO. https://obis.org. Accessed: 2019-08-29)
705770e5-3474-4e69-be8b-3107a0c5610a	The fishes collection (IC) of the Muséum national d'Histoire naturelle (MNHN - Paris)	Gicim data base, Pruvost P. Causse R., 2009 http://doi.org/10.15468/tm7whu	This work is licensed under a Creative Commons Attribution (CC-BY) 4.0 License	OBIS (2019) [The fishes collection (IC) of the Muséum national d'Histoire naturelle (MNHN - Paris)] (Available: Ocean Biodiversity Information System. Intergovernmental Oceanographic Commission of UNESCO. https://obis.org. Accessed: 2019-08-29)
8a1ae661-e911-4967-bc06-1168fc5f2d89	iziko South African Museum - Fish Collection	iziko South African Museum - Fish Collection	Restricted	OBIS (2019) [iziko South African Museum - Fish Collection] (Available: Ocean Biodiversity Information System. Intergovernmental Oceanographic Commission of UNESCO. https://obis.org. Accessed: 2019-08-29)
a4f7ee48-0d0b-4c05-a972-27a43b30db58	Institution - Collection	No available dataset citation	Unspecified intellectual rights	OBIS (2019) [Institution - Collection] (Available: Ocean Biodiversity Information System. Intergovernmental Oceanographic Commission of UNESCO. https://obis.org. Accessed: 2019-08-29)
a4f7ee48-0d0b-4c05-a972-27a43b30db58	Institution REVIZEE - Collection Pelagic Fishes	No available dataset citation	Unspecified intellectual rights	OBIS (2019) [Institution REVIZEE - Collection Pelagic Fishes] (Available: Ocean Biodiversity Information System. Intergovernmental Oceanographic Commission of UNESCO. https://obis.org. Accessed: 2019-08-29)
a4f7ee48-0d0b-4c05-a972-27a43b30db58	Institution MCM - Collection DEM	No available dataset citation	Unspecified intellectual rights	OBIS (2019) [Institution MCM - Collection DEM] (Available: Ocean Biodiversity Information System. Intergovernmental Oceanographic Commission of UNESCO. https://obis.org. Accessed: 2019-08-29)

cc8f28ce-e48d-4945-abfe-9d150a22dcd6	Hamburg pelagic fish database	Post, A. 1987. Pelagic transects of FRVs "Walther Herwig" and "Anton Dohrn" in the Atlantic Ocean 1966 to 1986. Mitt. Inst. f. Seefischerei d. BfaFi Hamburg, 42: 1-68.	This work is licensed under a Creative Commons Attribution (CC-BY) 4.0 License	OBIS (2019) [Hamburg pelagic fish database] (Available: Ocean Biodiversity Information System. Intergovernmental Oceanographic Commission of UNESCO. https://obis.org. Accessed: 2019-08-29)
ce1d93f3-8b0f-4ee7-9a4d-0393a6ec7fea	Atlantic Reference Centre Museum of Canadian Atlantic Organisms - Invertebrates and Fishes Data	Van Guelpen, L., 2016. Atlantic Reference Centre Museum of Canadian Atlantic Organisms - Invertebrates and Fishes Data. Version 4 In OBIS Canada Digital Collections. Bedford Institute of Oceanography, Dartmouth, NS, Canada. Published by OBIS, Digital http://www.iobis.org/. Accessed on –INSERT DATE	This work is licensed under a Creative Commons Attribution (CC-BY) 4.0 License	OBIS (2019) [Atlantic Reference Centre Museum of Canadian Atlantic Organisms - Invertebrates and Fishes Data] (Available: Ocean Biodiversity Information System. Intergovernmental Oceanographic Commission of UNESCO. https://obis.org. Accessed: 2019-08-29)
cfc56587-48c3-4e3d-9350-3a4d9a28b681	Institution MCM - Collection DEM	No available dataset citation	Unspecified intellectual rights	OBIS (2019) [Institution MCM - Collection DEM] (Available: Ocean Biodiversity Information System. Intergovernmental Oceanographic Commission of UNESCO. https://obis.org. Accessed: 2019-08-29)
cfc56587-48c3-4e3d-9350-3a4d9a28b681	Institution NOAA, NMFS, Northeast Fisheries Science Center - Collection DEEPWATER SYSTEMATICS	No available dataset citation	Unspecified intellectual rights	OBIS (2019) [Institution NOAA, NMFS, Northeast Fisheries Science Center - Collection DEEPWATER SYSTEMATICS] (Available: Ocean Biodiversity Information System. Intergovernmental Oceanographic Commission of UNESCO. https://obis.org. Accessed: 2019-08-29)
d6d6fe4c-425f-4ce7-bf28-7a6befaeb413	National Museum of Natural History Vertebrate Zoology Fishes Collections	National Museum of Natural History, Smithsonian Institution NMNH Fishes Collection Database. National Museum of Natural History, Smithsonian Institution, 10th and Constitution Ave. N.W., Washington, DC 20560-0193, 2007.	This work is licensed under a Creative Commons Attribution (CC-BY) 4.0 License	OBIS (2019) [National Museum of Natural History Vertebrate Zoology Fishes Collections] (Available: Ocean Biodiversity Information System. Intergovernmental Oceanographic Commission of UNESCO. https://obis.org. Accessed: 2019-08-29)

Diaphus effulgens

GBIF

GBIF Occurrence Download https://doi.org/10.15468/dd.76wfp2 Accessed from R via rgbif (https://github.com/ropensci/rgbif) on 2019-09-11

OBIS

OBIS (2019) Distribution records of Diaphus effulgens [Dataset] (Available: Ocean Biodiversity Information System. Intergovernmental Oceanographic Commission of UNESCO. www.obis.org. Accessed: 2019-08-29)

Dataset details:

Dataset_ID	Name	Citation	License	OBIS_citation
1f59030f-f116-4c34-915e-1882d819cda3	Institution Southampton Oceanography Ceter - Collection discovery	No available dataset citation	Unspecified intellectual rights	OBIS (2019) [Institution Southampton Oceanography Ceter - Collection discovery] (Available: Ocean Biodiversity Information System. Intergovernmental Oceanographic Commission of UNESCO. https://obis.org. Accessed: 2019-08-29)
705770e5-3474-4e69-be8b-3107a0c5610a	The fishes collection (IC) of the Muséum national d'Histoire naturelle (MNHN - Paris)	Gicim data base, Pruvost P. Causse R., 2009 http://doi.org/10.15468/tm7whu	This work is licensed under a Creative Commons Attribution (CC-BY) 4.0 License	OBIS (2019) [The fishes collection (IC) of the Muséum national d'Histoire naturelle (MNHN - Paris)] (Available: Ocean Biodiversity Information System. Intergovernmental Oceanographic Commission of UNESCO. https://obis.org. Accessed: 2019-08-29)
8629ec33-be4b-4384-933f-a511fbc29967	MAR-ECO 2004	Wenneck, T. de Lange, Falkenhaug, T. and O.A. Bergstad. 2008. Strategies, methods, and technologies adopted on the RV G.O. Sars MAR-ECO expedition to the mid-Atlantic Ridge in 2004. Deep-sea Research II. 55: 6-28.	This work is licensed under a Creative Commons Attribution (CC-BY) 4.0 License	OBIS (2019) [MAR-ECO 2004] (Available: Ocean Biodiversity Information System. Intergovernmental Oceanographic Commission of UNESCO. https://obis.org. Accessed: 2019-08-29)
8a1ae661-e911-4967-bc06-1168fc5f2d89	iziko South African Museum - Fish Collection	iziko South African Museum - Fish Collection	Restricted	OBIS (2019) [iziko South African Museum - Fish Collection] (Available: Ocean Biodiversity Information System. Intergovernmental Oceanographic Commission of UNESCO. https://obis.org. Accessed: 2019-08-29)
a4f7ee48-0d0b-4c05-a972-27a43b30db58	Institution MCM - Collection DEM	No available dataset citation	Unspecified intellectual rights	OBIS (2019) [Institution MCM - Collection DEM] (Available: Ocean Biodiversity Information System. Intergovernmental Oceanographic Commission of UNESCO. https://obis.org. Accessed: 2019-08-29)

b8617377-eb1c-4db2-baa6-8788a632e810	Ichthyology Collection - Royal Ontario Museum	NA	This work is licensed under a Creative Commons Attribution Non Commercial (CC-BY-NC) 4.0 License	OBIS (2019) [Ichthyology Collection - Royal Ontario Museum] (Available: Ocean Biodiversity Information System. Intergovernmental Oceanographic Commission of UNESCO. https://obis.org. Accessed: 2019-08-29)
c24bf1c2-2c62-4056-a841-56d94e6e876a	Fish specimens	ROM Fish Collection (accessed through GBIF data portal, http://data.gbif.org/datasets/resource/660, 2012-01-20) http://doi.org/10.15468/syisbx	Unrestricted	OBIS (2019) [Fish specimens] (Available: Ocean Biodiversity Information System. Intergovernmental Oceanographic Commission of UNESCO. https://obis.org. Accessed: 2019-08-29)
cc8f28ce-e48d-4945-abfe-9d150a22dcd6	Hamburg pelagic fish database	Post, A. 1987. Pelagic transects of FRVs "Walther Herwig" and "Anton Dohrn" in the Atlantic Ocean 1966 to 1986. Mitt. Inst. f. Seefischerei d. BfaFi Hamburg, 42: 1-68.	This work is licensed under a Creative Commons Attribution (CC-BY) 4.0 License	OBIS (2019) [Hamburg pelagic fish database] (Available: Ocean Biodiversity Information System. Intergovernmental Oceanographic Commission of UNESCO. https://obis.org. Accessed: 2019-08-29)
ce1d93f3-8b0f-4ee7-9a4d-0393a6ec7fea	Atlantic Reference Centre Museum of Canadian Atlantic Organisms - Invertebrates and Fishes Data	Van Guelpen, L., 2016. Atlantic Reference Centre Museum of Canadian Atlantic Organisms - Invertebrates and Fishes Data. Version 4 In OBIS Canada Digital Collections. Bedford Institute of Oceanography, Dartmouth, NS, Canada. Published by OBIS, Digital http://www.iobis.org/. Accessed on –INSERT DATE	This work is licensed under a Creative Commons Attribution (CC-BY) 4.0 License	OBIS (2019) [Atlantic Reference Centre Museum of Canadian Atlantic Organisms - Invertebrates and Fishes Data] (Available: Ocean Biodiversity Information System. Intergovernmental Oceanographic Commission of UNESCO. https://obis.org. Accessed: 2019-08-29)
cfc56587-48c3-4e3d-9350-3a4d9a28b681	Institution NOAA, NMFS, Northeast Fisheries Science Center - Collection DEEPWATER SYSTEMATICS	No available dataset citation	Unspecified intellectual rights	OBIS (2019) [Institution NOAA, NMFS, Northeast Fisheries Science Center - Collection DEEPWATER SYSTEMATICS] (Available: Ocean Biodiversity Information System. Intergovernmental Oceanographic Commission of UNESCO. https://obis.org. Accessed: 2019-08-29)
d286ae50-ea29-4aa4-8028-2e6e5945a039	Institution REVIZEE - Collection Demersal Fishes	No available dataset citation	Unspecified intellectual rights	OBIS (2019) [Institution REVIZEE - Collection Demersal Fishes] (Available: Ocean Biodiversity Information System. Intergovernmental Oceanographic Commission of UNESCO. https://obis.org. Accessed: 2019-08-29)

d6d6fe4c-425f-4ce7-bf28-7a6befaeb413	National Museum of Natural History Vertebrate Zoology Fishes Collections	National Museum of Natural History, Smithsonian Institution NMNH Fishes Collection Database. National Museum of Natural History, Smithsonian Institution, 10th and Constitution Ave. N.W., Washington, DC 20560-0193, 2007.	This work is licensed under a Creative Commons Attribution (CC-BY) 4.0 License	OBIS (2019) [National Museum of Natural History Vertebrate Zoology Fishes Collections] (Available: Ocean Biodiversity Information System. Intergovernmental Oceanographic Commission of UNESCO. https://obis.org. Accessed: 2019-08-29)

Lampanyctus nobilis

GBIF

GBIF Occurrence Download https://doi.org/10.15468/dd.6eay78 Accessed from R via rgbif (https://github.com/ropensci/rgbif) on 2019-09-11

OBIS

OBIS (2019) Distribution records of Lampanyctus nobilis [Dataset] (Available: Ocean Biodiversity Information System. Intergovernmental Oceanographic Commission of UNESCO. www.obis.org. Accessed: 2019-08-29)

Dataset details:

Dataset_ID	Name	Citation	License	OBIS_citation
270f3e70-ff9b-411d-b170-2bc914d83f26	Biological Reference Collections ICM CSIC	Olivas González F J (2016): Biological Reference Collections ICM CSIC. Institute of Marine Sciences (ICM-CSIC). https://dx.doi.org/10.15470/qlqqdx	This work is licensed under a Creative Commons Attribution Non Commercial (CC-BY-NC) 4.0 License	OBIS (2019) [Biological Reference Collections ICM CSIC] (Available: Ocean Biodiversity Information System. Intergovernmental Oceanographic Commission of UNESCO. https://obis.org. Accessed: 2019-08-29)
2870c548-343e-4575-ac67-a4da35182c52	Institution Shirshov Institute - Collection SKAO	No available dataset citation	Unspecified intellectual rights	OBIS (2019) [Institution Shirshov Institute - Collection SKAO] (Available: Ocean Biodiversity Information System. Intergovernmental Oceanographic Commission of UNESCO. https://obis.org. Accessed: 2019-08-29)

705770e5-3474-4e69-be8b-3107a0c5610a	The fishes collection (IC) of the Muséum national d'Histoire naturelle (MNHN - Paris)	Gicim data base, Pruvost P. Causse R., 2009 http://doi.org/10.15468/tm7whu	This work is licensed under a Creative Commons Attribution (CC-BY) 4.0 License	OBIS (2019) [The fishes collection (IC) of the Muséum national d'Histoire naturelle (MNHN - Paris)] (Available: Ocean Biodiversity Information System. Intergovernmental Oceanographic Commission of UNESCO. https://obis.org. Accessed: 2019-08-29)
8a1ae661-e911-4967-bc06-1168fc5f2d89	iziko South African Museum - Fish Collection	iziko South African Museum - Fish Collection	Restricted	OBIS (2019) [iziko South African Museum - Fish Collection] (Available: Ocean Biodiversity Information System. Intergovernmental Oceanographic Commission of UNESCO. https://obis.org. Accessed: 2019-08-29)
cc8f28ce-e48d-4945-abfe-9d150a22dcd6	Hamburg pelagic fish database	Post, A. 1987. Pelagic transects of FRVs "Walther Herwig" and "Anton Dohrn" in the Atlantic Ocean 1966 to 1986. Mitt. Inst. f. Seefischerei d. BfaFi Hamburg, 42: 1-68.	This work is licensed under a Creative Commons Attribution (CC-BY) 4.0 License	OBIS (2019) [Hamburg pelagic fish database] (Available: Ocean Biodiversity Information System. Intergovernmental Oceanographic Commission of UNESCO. https://obis.org. Accessed: 2019-08-29)
ce1d93f3-8b0f-4ee7-9a4d-0393a6ec7fea	Atlantic Reference Centre Museum of Canadian Atlantic Organisms - Invertebrates and Fishes Data	Van Guelpen, L., 2016. Atlantic Reference Centre Museum of Canadian Atlantic Organisms - Invertebrates and Fishes Data. Version 4 In OBIS Canada Digital Collections. Bedford Institute of Oceanography, Dartmouth, NS, Canada. Published by OBIS, Digital http://www.iobis.org/. Accessed on –INSERT DATE	This work is licensed under a Creative Commons Attribution (CC-BY) 4.0 License	OBIS (2019) [Atlantic Reference Centre Museum of Canadian Atlantic Organisms - Invertebrates and Fishes Data] (Available: Ocean Biodiversity Information System. Intergovernmental Oceanographic Commission of UNESCO. https://obis.org. Accessed: 2019-08-29)
d6d6fe4c-425f-4ce7-bf28-7a6befaeb413	National Museum of Natural History Vertebrate Zoology Fishes Collections	National Museum of Natural History, Smithsonian Institution NMNH Fishes Collection Database. National Museum of Natural History, Smithsonian Institution, 10th and Constitution Ave. N.W., Washington, DC 20560-0193, 2007.	This work is licensed under a Creative Commons Attribution (CC-BY) 4.0 License	OBIS (2019) [National Museum of Natural History Vertebrate Zoology Fishes Collections] (Available: Ocean Biodiversity Information System. Intergovernmental Oceanographic Commission of UNESCO. https://obis.org. Accessed: 2019-08-29)

ff8b7809-41bc-40ad-8160-0e33862817a0	Biodiversity Research Museum, Academia Sinica, Taiwan	TELDAP, Biodiversity Research Museum, Academia Sinica, Taiwan (accessed through GBIF data portal, http://data.gbif.org/datasets/resource/9093, yyyy-mm-dd)	Unrestricted	OBIS (2019) [Biodiversity Research Museum, Academia Sinica, Taiwan] (Available: Ocean Biodiversity Information System. Intergovernmental Oceanographic Commission of UNESCO. https://obis.org. Accessed: 2019-08-29)

Myctophum spinosum

GBIF

GBIF Occurrence Download https://doi.org/10.15468/dd.3kccnx Accessed from R via rgbif (https://github.com/ropensci/rgbif) on 2019-09-11

OBIS

OBIS (2019) Distribution records of Myctophum spinosum [Dataset] (Available: Ocean Biodiversity Information System. Intergovernmental Oceanographic Commission of UNESCO. www.obis.org. Accessed: 2019-08-29)

Dataset details:

Dataset_ID	Name	Citation	License	OBIS_citation
04e3fd32-b08b-4806-a016-d2dff52ae55a	Asia-Pacific Dataset	Jintsu-Uchifune, Y., Yamamoto, H. (2016) Marine organism occurrence data of the Asia-Pacific region extracted from literature. Available at https://doi.org/10.48518/00002. Accessed on yyyy-mm-dd.	This work is licensed under a Creative Commons Attribution (CC-BY) 4.0 License	OBIS (2019) [Asia-Pacific Dataset] (Available: Ocean Biodiversity Information System. Intergovernmental Oceanographic Commission of UNESCO. https://obis.org. Accessed: 2019-08-29)
10b213e6-a9c4-459e-a40c-ef9edc461b97	Marine data from the Bernice P. Bishop Museum	Pyle R (2016). Bernice P. Bishop Museum. Version 8.1. Bernice Pauahi Bishop Museum. Occurrence dataset https://doi.org/10.15468/s6ctus accessed via GBIF.org on 2018-11-16.	To the extent possible under law, the publisher has waived all rights to these data and has dedicated them to the Public Domain (CC0 1.0)	OBIS (2019) [Marine data from the Bernice P. Bishop Museum] (Available: Ocean Biodiversity Information System. Intergovernmental Oceanographic Commission of UNESCO. https://obis.org. Accessed: 2019-08-29)

685b3956-c37a-433a-b661-2bb7b11cf9f8	Soviet Trawl Fishery Data (New Zealand Waters) 1964-1987	Ministry for Primary Industries (2014). Soviet Fishery Data (New Zealand Waters) 1964-1987. Southwestern Pacific OBIS, National Institute of Water and Atmospheric Research (NIWA), Wellington, New Zealand, 111883 records, Online http://nzobisipt.niwa.co.nz/resource.do?r=mbis_soviettrawl released on November 5, 2014.	This work is licensed under a Creative Commons Attribution (CC-BY) 4.0 License	OBIS (2019) [Soviet Trawl Fishery Data (New Zealand Waters) 1964-1987] (Available: Ocean Biodiversity Information System. Intergovernmental Oceanographic Commission of UNESCO. https://obis.org. Accessed: 2019-08-29)
6a5bc28f-4dfe-4cbf-8a55-7e3a843997ab	SPC NECTALIS Zooplankton/Micronekton specimens, New Caledonia 2014	Allain, V., Menkes, C., 2014. Nectalis 3 cruise, RV Alis. https://doi.org/10.17600/14004900	This work is licensed under a Creative Commons Attribution Non Commercial (CC-BY-NC) 4.0 License	OBIS (2019) [SPC NECTALIS Zooplankton/Micronekton specimens, New Caledonia 2014] (Available: Ocean Biodiversity Information System. Intergovernmental Oceanographic Commission of UNESCO. https://obis.org. Accessed: 2019-08-29)
705770e5-3474-4e69-be8b-3107a0c5610a	The fishes collection (IC) of the Muséum national d'Histoire naturelle (MNHN - Paris)	Gicim data base, Pruvost P. Causse R., 2009 http://doi.org/10.15468/tm7whu	This work is licensed under a Creative Commons Attribution (CC-BY) 4.0 License	OBIS (2019) [The fishes collection (IC) of the Muséum national d'Histoire naturelle (MNHN - Paris)] (Available: Ocean Biodiversity Information System. Intergovernmental Oceanographic Commission of UNESCO. https://obis.org. Accessed: 2019-08-29)
87a421bf-4646-49e3-89b7-409b93f2ac7c	Institution UWFC - Collection ADULT COLLECTION	No available dataset citation	Unspecified intellectual rights	OBIS (2019) [Institution UWFC - Collection ADULT COLLECTION] (Available: Ocean Biodiversity Information System. Intergovernmental Oceanographic Commission of UNESCO. https://obis.org. Accessed: 2019-08-29)
b8617377-eb1c-4db2-baa6-8788a632e810	Ichthyology Collection - Royal Ontario Museum	NA	This work is licensed under a Creative Commons Attribution Non Commercial (CC-BY-NC) 4.0 License	OBIS (2019) [Ichthyology Collection - Royal Ontario Museum] (Available: Ocean Biodiversity Information System. Intergovernmental Oceanographic Commission of UNESCO. https://obis.org. Accessed: 2019-08-29)
c24bf1c2-2c62-4056-a841-56d94e6e876a	Fish specimens	ROM Fish Collection (accessed through GBIF data portal, http://data.gbif.org/datasets/resource/660, 2012-01-20) http://doi.org/10.15468/syisbx	Unrestricted	OBIS (2019) [Fish specimens] (Available: Ocean Biodiversity Information System. Intergovernmental Oceanographic Commission of UNESCO. https://obis.org. Accessed: 2019-08-29)

d6d6fe4c-425f-4ce7-bf28-7a6befaeb413	National Museum of Natural History Vertebrate Zoology Fishes Collections	National Museum of Natural History, Smithsonian Institution NMNH Fishes Collection Database. National Museum of Natural History, Smithsonian Institution, 10th and Constitution Ave. N.W., Washington, DC 20560-0193, 2007.	This work is licensed under a Creative Commons Attribution (CC-BY) 4.0 License	OBIS (2019) [National Museum of Natural History Vertebrate Zoology Fishes Collections] (Available: Ocean Biodiversity Information System. Intergovernmental Oceanographic Commission of UNESCO. https://obis.org. Accessed: 2019-08-29)
ff8b7809-41bc-40ad-8160-0e33862817a0	Biodiversity Research Museum, Academia Sinica, Taiwan	TELDAP, Biodiversity Research Museum, Academia Sinica, Taiwan (accessed through GBIF data portal, http://data.gbif.org/datasets/resource/9093, yyyy-mm-dd)	Unrestricted	OBIS (2019) [Biodiversity Research Museum, Academia Sinica, Taiwan] (Available: Ocean Biodiversity Information System. Intergovernmental Oceanographic Commission of UNESCO. https://obis.org. Accessed: 2019-08-29)

Lepidophanes guentheri

GBIF

GBIF Occurrence Download https://doi.org/10.15468/dd.cnwubu Accessed from R via rgbif (https://github.com/ropensci/rgbif) on 2019-09-11

OBIS

OBIS (2019) Distribution records of Lepidophanes guentheri [Dataset] (Available: Ocean Biodiversity Information System. Intergovernmental Oceanographic Commission of UNESCO. www.obis.org. Accessed: 2019-08-29)

Dataset details:

Dataset_ID	Name	Citation	License	OBIS_citation
09be818d-531c-4d86-8eba-5db81d42fdba	COLETA - IMAR/DOP-Uac reference collection from 1977 to 2012	Institute of Marine Research (IMAR - Azores), Portugal; Department of Oceanography and Fisheries (DOP) - UAC, Portugal (2015): COLETA - IMAR/DOP-Uac reference collection from 1977 to 2012. https://dx.doi.org/10.14284/23	This work is licensed under a Creative Commons Attribution (CC-BY) 4.0 License	OBIS (2019) [COLETA - IMAR/DOP-Uac reference collection from 1977 to 2012] (Available: Ocean Biodiversity Information System. Intergovernmental Oceanographic Commission of UNESCO. https://obis.org. Accessed: 2019-08-29)

1f59030f-f116-4c34-915e-1882d819cda3	Institution Southampton Oceanography Ceter - Collection discovery	No available dataset citation	Unspecified intellectual rights	OBIS (2019) [Institution Southampton Oceanography Ceter - Collection discovery] (Available: Ocean Biodiversity Information System. Intergovernmental Oceanographic Commission of UNESCO. https://obis.org. Accessed: 2019-08-29)
2870c548-343e-4575-ac67-a4da35182c52	Institution Shirshov Institute - Collection SKAO	No available dataset citation	Unspecified intellectual rights	OBIS (2019) [Institution Shirshov Institute - Collection SKAO] (Available: Ocean Biodiversity Information System. Intergovernmental Oceanographic Commission of UNESCO. https://obis.org. Accessed: 2019-08-29)
308a501c-a187-498e-a8bd-9cf3d2b70bd9	Institution REVIZEE - Collection Ictioplankton	No available dataset citation	Unspecified intellectual rights	OBIS (2019) [Institution REVIZEE - Collection Ictioplankton] (Available: Ocean Biodiversity Information System. Intergovernmental Oceanographic Commission of UNESCO. https://obis.org. Accessed: 2019-08-29)
3d922162-062c-4ad2-bf4a-f2493bd3a95d	Institution Bedford Institute of Oceanography (BIO) - Collection SUMMER	No available dataset citation	Unspecified intellectual rights	OBIS (2019) [Institution Bedford Institute of Oceanography (BIO) - Collection SUMMER] (Available: Ocean Biodiversity Information System. Intergovernmental Oceanographic Commission of UNESCO. https://obis.org. Accessed: 2019-08-29)
6c19184e-c305-4273-8890-6d342d86f865	Institution REVIZEE - Collection Pelagic Fishes	No available dataset citation	Unspecified intellectual rights	OBIS (2019) [Institution REVIZEE - Collection Pelagic Fishes] (Available: Ocean Biodiversity Information System. Intergovernmental Oceanographic Commission of UNESCO. https://obis.org. Accessed: 2019-08-29)

7e4228e5-a962-4b01-952f-7bf33e213a9c	BioChem: Sameoto zooplankton collection	Sameoto, D.D., Kennedy, M., Spry, J.S, Spry, J.M. (2013). Zooplankton datasets collected using the BIONESS sampler, ring nets and an Icelandic high speed sampler, 1967-2006. OBIS Canada Digital Collections. Published by OBIS http://www.iobis.org/. Accessed on –INSERT DATE	rights: http://data.gc.ca/eng/open-government-licence-canada & http://www.canadensys.net/norms rights holder: Her Majesty the Queen in right of Canada, as represented by the Minister of Fisheries and Oceans	OBIS (2019) [BioChem: Sameoto zooplankton collection] (Available: Ocean Biodiversity Information System. Intergovernmental Oceanographic Commission of UNESCO. https://obis.org. Accessed: 2019-08-29)
8629ec33-be4b-4384-933f-a511fbc29967	MAR-ECO 2004	Wenneck, T. de Lange, Falkenhaug, T. and O.A. Bergstad. 2008. Strategies, methods, and technologies adopted on the RV G.O. Sars MAR-ECO expedition to the mid-Atlantic Ridge in 2004. Deep-sea Research II. 55: 6-28.	This work is licensed under a Creative Commons Attribution (CC-BY) 4.0 License	OBIS (2019) [MAR-ECO 2004] (Available: Ocean Biodiversity Information System. Intergovernmental Oceanographic Commission of UNESCO. https://obis.org. Accessed: 2019-08-29)
8a1ae661-e911-4967-bc06-1168fc5f2d89	iziko South African Museum - Fish Collection	iziko South African Museum - Fish Collection	Restricted	OBIS (2019) [iziko South African Museum - Fish Collection] (Available: Ocean Biodiversity Information System. Intergovernmental Oceanographic Commission of UNESCO. https://obis.org. Accessed: 2019-08-29)
b8617377-eb1c-4db2-baa6-8788a632e810	Ichthyology Collection - Royal Ontario Museum	NA	This work is licensed under a Creative Commons Attribution Non Commercial (CC-BY-NC) 4.0 License	OBIS (2019) [Ichthyology Collection - Royal Ontario Museum] (Available: Ocean Biodiversity Information System. Intergovernmental Oceanographic Commission of UNESCO. https://obis.org. Accessed: 2019-08-29)
c24bf1c2-2c62-4056-a841-56d94e6e876a	Fish specimens	ROM Fish Collection (accessed through GBIF data portal, http://data.gbif.org/datasets/resource/660, 2012-01-20) http://doi.org/10.15468/syisbx	Unrestricted	OBIS (2019) [Fish specimens] (Available: Ocean Biodiversity Information System. Intergovernmental Oceanographic Commission of UNESCO. https://obis.org. Accessed: 2019-08-29)

cc8f28ce-e48d-4945-abfe-9d150a22dcd6	Hamburg pelagic fish database	Post, A. 1987. Pelagic transects of FRVs "Walther Herwig" and "Anton Dohrn" in the Atlantic Ocean 1966 to 1986. Mitt. Inst. f. Seefischerei d. BfaFi Hamburg, 42: 1-68.	This work is licensed under a Creative Commons Attribution (CC-BY) 4.0 License	OBIS (2019) [Hamburg pelagic fish database] (Available: Ocean Biodiversity Information System. Intergovernmental Oceanographic Commission of UNESCO. https://obis.org. Accessed: 2019-08-29)
ce1d93f3-8b0f-4ee7-9a4d-0393a6ec7fea	Atlantic Reference Centre Museum of Canadian Atlantic Organisms - Invertebrates and Fishes Data	Van Guelpen, L., 2016. Atlantic Reference Centre Museum of Canadian Atlantic Organisms - Invertebrates and Fishes Data. Version 4 In OBIS Canada Digital Collections. Bedford Institute of Oceanography, Dartmouth, NS, Canada. Published by OBIS, Digital http://www.iobis.org/. Accessed on –INSERT DATE	This work is licensed under a Creative Commons Attribution (CC-BY) 4.0 License	OBIS (2019) [Atlantic Reference Centre Museum of Canadian Atlantic Organisms - Invertebrates and Fishes Data] (Available: Ocean Biodiversity Information System. Intergovernmental Oceanographic Commission of UNESCO. https://obis.org. Accessed: 2019-08-29)
cfc56587-48c3-4e3d-9350-3a4d9a28b681	Institution NOAA, NMFS, Northeast Fisheries Science Center - Collection DEEPWATER SYSTEMATICS	No available dataset citation	Unspecified intellectual rights	OBIS (2019) [Institution NOAA, NMFS, Northeast Fisheries Science Center - Collection DEEPWATER SYSTEMATICS] (Available: Ocean Biodiversity Information System. Intergovernmental Oceanographic Commission of UNESCO. https://obis.org. Accessed: 2019-08-29)
d286ae50-ea29-4aa4-8028-2e6e5945a039	Institution REVIZEE - Collection Demersal Fishes	No available dataset citation	Unspecified intellectual rights	OBIS (2019) [Institution REVIZEE - Collection Demersal Fishes] (Available: Ocean Biodiversity Information System. Intergovernmental Oceanographic Commission of UNESCO. https://obis.org. Accessed: 2019-08-29)
d6d6fe4c-425f-4ce7-bf28-7a6befaeb413	National Museum of Natural History Vertebrate Zoology Fishes Collections	National Museum of Natural History, Smithsonian Institution NMNH Fishes Collection Database. National Museum of Natural History, Smithsonian Institution, 10th and Constitution Ave. N.W., Washington, DC 20560-0193, 2007.	This work is licensed under a Creative Commons Attribution (CC-BY) 4.0 License	OBIS (2019) [National Museum of Natural History Vertebrate Zoology Fishes Collections] (Available: Ocean Biodiversity Information System. Intergovernmental Oceanographic Commission of UNESCO. https://obis.org. Accessed: 2019-08-29)

Diaphus garmani

GBIF

GBIF Occurrence Download https://doi.org/10.15468/dd.zxxxpv Accessed from R via rgbif (https://github.com/ropensci/rgbif) on 2019-09-11

OBIS

OBIS (2019) Distribution records of Diaphus garmani [Dataset] (Available: Ocean Biodiversity Information System. Intergovernmental Oceanographic Commission of UNESCO. www.obis.org. Accessed: 2019-08-29)

Dataset details:

Dataset_ID	Name	Citation	License	OBIS_citation
270f3e70-ff9b-411d-b170-2bc914d83f26	Biological Reference Collections ICM CSIC	Olivas González F J (2016): Biological Reference Collections ICM CSIC. Institute of Marine Sciences (ICM-CSIC). https://dx.doi.org/10.15470/qlqqdx	This work is licensed under a Creative Commons Attribution Non Commercial (CC-BY-NC) 4.0 License	OBIS (2019) [Biological Reference Collections ICM CSIC] (Available: Ocean Biodiversity Information System. Intergovernmental Oceanographic Commission of UNESCO. https://obis.org. Accessed: 2019-08-29)
4e25e0ce-b17d-4192-9b55-417f1e0c4fc8	Institution KU - Collection KUI	No available dataset citation	Unspecified intellectual rights	OBIS (2019) [Institution KU - Collection KUI] (Available: Ocean Biodiversity Information System. Intergovernmental Oceanographic Commission of UNESCO. https://obis.org. Accessed: 2019-08-29)
6a5bc28f-4dfe-4cbf-8a55-7e3a843997ab	SPC NECTALIS Zooplankton/Micronekton specimens, New Caledonia 2014	Allain, V., Menkes, C., 2014. Nectalis 3 cruise, RV Alis. https://doi.org/10.17600/14004900	This work is licensed under a Creative Commons Attribution Non Commercial (CC-BY-NC) 4.0 License	OBIS (2019) [SPC NECTALIS Zooplankton/Micronekton specimens, New Caledonia 2014] (Available: Ocean Biodiversity Information System. Intergovernmental Oceanographic Commission of UNESCO. https://obis.org. Accessed: 2019-08-29)
6c19184e-c305-4273-8890-6d342d86f865	Institution REVIZEE - Collection Pelagic Fishes	No available dataset citation	Unspecified intellectual rights	OBIS (2019) [Institution REVIZEE - Collection Pelagic Fishes] (Available: Ocean Biodiversity Information System. Intergovernmental Oceanographic Commission of UNESCO. https://obis.org. Accessed: 2019-08-29)

705770e5-3474-4e69-be8b-3107a0c5610a	The fishes collection (IC) of the Muséum national d'Histoire naturelle (MNHN - Paris)	Gicim data base, Pruvost P. Causse R., 2009 http://doi.org/10.15468/tm7whu	This work is licensed under a Creative Commons Attribution (CC-BY) 4.0 License	OBIS (2019) [The fishes collection (IC) of the Muséum national d'Histoire naturelle (MNHN - Paris)] (Available: Ocean Biodiversity Information System. Intergovernmental Oceanographic Commission of UNESCO. https://obis.org. Accessed: 2019-08-29)
784c3f00-9b0b-4b2d-a0e7-2de304537f8b	Institution TU - Collection Fish	No available dataset citation	Unspecified intellectual rights	OBIS (2019) [Institution TU - Collection Fish] (Available: Ocean Biodiversity Information System. Intergovernmental Oceanographic Commission of UNESCO. https://obis.org. Accessed: 2019-08-29)
8a1ae661-e911-4967-bc06-1168fc5f2d89	iziko South African Museum - Fish Collection	iziko South African Museum - Fish Collection	Restricted	OBIS (2019) [iziko South African Museum - Fish Collection] (Available: Ocean Biodiversity Information System. Intergovernmental Oceanographic Commission of UNESCO. https://obis.org. Accessed: 2019-08-29)
9ff216fc-777e-4f9b-9860-95ed7366870d	Institution SAIAB - Collection SAIAB	No available dataset citation	Unspecified intellectual rights	OBIS (2019) [Institution SAIAB - Collection SAIAB] (Available: Ocean Biodiversity Information System. Intergovernmental Oceanographic Commission of UNESCO. https://obis.org. Accessed: 2019-08-29)
b8617377-eb1c-4db2-baa6-8788a632e810	Ichthyology Collection - Royal Ontario Museum	NA	This work is licensed under a Creative Commons Attribution Non Commercial (CC-BY-NC) 4.0 License	OBIS (2019) [Ichthyology Collection - Royal Ontario Museum] (Available: Ocean Biodiversity Information System. Intergovernmental Oceanographic Commission of UNESCO. https://obis.org. Accessed: 2019-08-29)
c24bf1c2-2c62-4056-a841-56d94e6e876a	Fish specimens	ROM Fish Collection (accessed through GBIF data portal, http://data.gbif.org/datasets/resource/660, 2012-01-20) http://doi.org/10.15468/syisbx	Unrestricted	OBIS (2019) [Fish specimens] (Available: Ocean Biodiversity Information System. Intergovernmental Oceanographic Commission of UNESCO. https://obis.org. Accessed: 2019-08-29)
d286ae50-ea29-4aa4-8028-2e6e5945a039	Institution REVIZEE - Collection Demersal Fishes	No available dataset citation	Unspecified intellectual rights	OBIS (2019) [Institution REVIZEE - Collection Demersal Fishes] (Available: Ocean Biodiversity Information System. Intergovernmental Oceanographic Commission of UNESCO. https://obis.org. Accessed: 2019-08-29)

d6d6fe4c-425f-4ce7-bf28-7a6befaeb413	National Museum of Natural History Vertebrate Zoology Fishes Collections	National Museum of Natural History, Smithsonian Institution NMNH Fishes Collection Database. National Museum of Natural History, Smithsonian Institution, 10th and Constitution Ave. N.W., Washington, DC 20560-0193, 2007.	This work is licensed under a Creative Commons Attribution (CC-BY) 4.0 License	OBIS (2019) [National Museum of Natural History Vertebrate Zoology Fishes Collections] (Available: Ocean Biodiversity Information System. Intergovernmental Oceanographic Commission of UNESCO. https://obis.org. Accessed: 2019-08-29)
ff8b7809-41bc-40ad-8160-0e33862817a0	Biodiversity Research Museum, Academia Sinica, Taiwan	TELDAP, Biodiversity Research Museum, Academia Sinica, Taiwan (accessed through GBIF data portal, http://data.gbif.org/datasets/resource/9093, yyyy-mm-dd)	Unrestricted	OBIS (2019) [Biodiversity Research Museum, Academia Sinica, Taiwan] (Available: Ocean Biodiversity Information System. Intergovernmental Oceanographic Commission of UNESCO. https://obis.org. Accessed: 2019-08-29)

Myctophum obtusirostre

GBIF

GBIF Occurrence Download https://doi.org/10.15468/dd.rgqt55 Accessed from R via rgbif (https://github.com/ropensci/rgbif) on 2019-09-11

OBIS

OBIS (2019) Distribution records of Myctophum obtusirostre [Dataset] (Available: Ocean Biodiversity Information System. Intergovernmental Oceanographic Commission of UNESCO. www.obis.org. Accessed: 2019-08-29)

Dataset details:

Dataset_ID	Name	Citation	License	OBIS_citation
04e3fd32-b08b-4806-a016-d2dff52ae55a	Asia-Pacific Dataset	Jintsu-Uchifune, Y., Yamamoto, H. (2016) Marine organism occurrence data of the Asia-Pacific region extracted from literature. Available at https://doi.org/10.48518/00002. Accessed on yyyy-mm-dd.	This work is licensed under a Creative Commons Attribution (CC-BY) 4.0 License	OBIS (2019) [Asia-Pacific Dataset] (Available: Ocean Biodiversity Information System. Intergovernmental Oceanographic Commission of UNESCO. https://obis.org. Accessed: 2019-08-29)

270f3e70-ff9b-411d-b170-2bc914d83f26	Biological Reference Collections ICM CSIC	Olivas González F J (2016): Biological Reference Collections ICM CSIC. Institute of Marine Sciences (ICM-CSIC). https://dx.doi.org/10.15470/qlqqdx	This work is licensed under a Creative Commons Attribution Non Commercial (CC-BY-NC) 4.0 License	OBIS (2019) [Biological Reference Collections ICM CSIC] (Available: Ocean Biodiversity Information System. Intergovernmental Oceanographic Commission of UNESCO. https://obis.org. Accessed: 2019-08-29)
308a501c-a187-498e-a8bd-9cf3d2b70bd9	Institution REVIZEE - Collection Ictioplankton	No available dataset citation	Unspecified intellectual rights	OBIS (2019) [Institution REVIZEE - Collection Ictioplankton] (Available: Ocean Biodiversity Information System. Intergovernmental Oceanographic Commission of UNESCO. https://obis.org. Accessed: 2019-08-29)
4354345d-7faf-4376-b326-ffbc04b6b0cd	No available dataset name	No available dataset citation	Unspecified intellectual rights	OBIS (2019) [No available dataset name] (Available: Ocean Biodiversity Information System. Intergovernmental Oceanographic Commission of UNESCO. https://obis.org. Accessed: 2019-08-29)
513e4437-9cc7-4383-9a69-1bd18ec2046a	MARMAP Neuston Nets 1990-2009	Marcel Reichert, 2010, MARMAP Neuston Nets 1990-2009, SCDNR/NOAA MARMAP Program, SCDNR MARMAP Aggregate data surveys, The Marine Resources Monitoring, Assessment, and Prediction (MARMAP) Program, Marine Resources Research Institute, South Carolina Department of Natural Resources, P. O. Box 12559, Charleston SC 29422-2559, U.S.A.Retrieved from http://www.usgs.gov/obis-usa/	Restricted	OBIS (2019) [MARMAP Neuston Nets 1990-2009] (Available: Ocean Biodiversity Information System. Intergovernmental Oceanographic Commission of UNESCO. https://obis.org. Accessed: 2019-08-29)
6a5bc28f-4dfe-4cbf-8a55-7e3a843997ab	SPC NECTALIS Zooplankton/Micronekton specimens, New Caledonia 2014	Allain, V., Menkes, C., 2014. Nectalis 3 cruise, RV Alis. https://doi.org/10.17600/14004900	This work is licensed under a Creative Commons Attribution Non Commercial (CC-BY-NC) 4.0 License	OBIS (2019) [SPC NECTALIS Zooplankton/Micronekton specimens, New Caledonia 2014] (Available: Ocean Biodiversity Information System. Intergovernmental Oceanographic Commission of UNESCO. https://obis.org. Accessed: 2019-08-29)

6c19184e-c305-4273-8890-6d342d86f865	Institution - Collection	No available dataset citation	Unspecified intellectual rights	OBIS (2019) [Institution - Collection] (Available: Ocean Biodiversity Information System. Intergovernmental Oceanographic Commission of UNESCO. https://obis.org. Accessed: 2019-08-29)
705770e5-3474-4e69-be8b-3107a0c5610a	The fishes collection (IC) of the Muséum national d'Histoire naturelle (MNHN - Paris)	Gicim data base, Pruvost P. Causse R., 2009 http://doi.org/10.15468/tm7whu	This work is licensed under a Creative Commons Attribution (CC-BY) 4.0 License	OBIS (2019) [The fishes collection (IC) of the Muséum national d'Histoire naturelle (MNHN - Paris)] (Available: Ocean Biodiversity Information System. Intergovernmental Oceanographic Commission of UNESCO. https://obis.org. Accessed: 2019-08-29)
87a421bf-4646-49e3-89b7-409b93f2ac7c	Institution - Collection	No available dataset citation	Unspecified intellectual rights	OBIS (2019) [Institution - Collection] (Available: Ocean Biodiversity Information System. Intergovernmental Oceanographic Commission of UNESCO. https://obis.org. Accessed: 2019-08-29)
87a421bf-4646-49e3-89b7-409b93f2ac7c	Institution REVIZEE - Collection Pelagic Fishes	No available dataset citation	Unspecified intellectual rights	OBIS (2019) [Institution REVIZEE - Collection Pelagic Fishes] (Available: Ocean Biodiversity Information System. Intergovernmental Oceanographic Commission of UNESCO. https://obis.org. Accessed: 2019-08-29)
9ff216fc-777e-4f9b-9860-95ed7366870d	Institution REVIZEE - Collection Pelagic Fishes	No available dataset citation	Unspecified intellectual rights	OBIS (2019) [Institution REVIZEE - Collection Pelagic Fishes] (Available: Ocean Biodiversity Information System. Intergovernmental Oceanographic Commission of UNESCO. https://obis.org. Accessed: 2019-08-29)
b8617377-eb1c-4db2-baa6-8788a632e810	Ichthyology Collection - Royal Ontario Museum	NA	This work is licensed under a Creative Commons Attribution Non Commercial (CC-BY-NC) 4.0 License	OBIS (2019) [Ichthyology Collection - Royal Ontario Museum] (Available: Ocean Biodiversity Information System. Intergovernmental Oceanographic Commission of UNESCO. https://obis.org. Accessed: 2019-08-29)
c24bf1c2-2c62-4056-a841-56d94e6e876a	Fish specimens	ROM Fish Collection (accessed through GBIF data portal, http://data.gbif.org/datasets/resource/660, 2012-01-20) http://doi.org/10.15468/syisbx	Unrestricted	OBIS (2019) [Fish specimens] (Available: Ocean Biodiversity Information System. Intergovernmental Oceanographic Commission of UNESCO. https://obis.org. Accessed: 2019-08-29)

cc8f28ce-e48d-4945-abfe-9d150a22dcd6	Hamburg pelagic fish database	Post, A. 1987. Pelagic transects of FRVs "Walther Herwig" and "Anton Dohrn" in the Atlantic Ocean 1966 to 1986. Mitt. Inst. f. Seefischerei d. BfaFi Hamburg, 42: 1-68.	This work is licensed under a Creative Commons Attribution (CC-BY) 4.0 License	OBIS (2019) [Hamburg pelagic fish database] (Available: Ocean Biodiversity Information System. Intergovernmental Oceanographic Commission of UNESCO. https://obis.org. Accessed: 2019-08-29)
d286ae50-ea29-4aa4-8028-2e6e5945a039	Institution REVIZEE - Collection Pelagic Fishes	No available dataset citation	Unspecified intellectual rights	OBIS (2019) [Institution REVIZEE - Collection Pelagic Fishes] (Available: Ocean Biodiversity Information System. Intergovernmental Oceanographic Commission of UNESCO. https://obis.org. Accessed: 2019-08-29)
d6d6fe4c-425f-4ce7-bf28-7a6befaeb413	National Museum of Natural History Vertebrate Zoology Fishes Collections	National Museum of Natural History, Smithsonian Institution NMNH Fishes Collection Database. National Museum of Natural History, Smithsonian Institution, 10th and Constitution Ave. N.W., Washington, DC 20560-0193, 2007.	This work is licensed under a Creative Commons Attribution (CC-BY) 4.0 License	OBIS (2019) [National Museum of Natural History Vertebrate Zoology Fishes Collections] (Available: Ocean Biodiversity Information System. Intergovernmental Oceanographic Commission of UNESCO. https://obis.org. Accessed: 2019-08-29)
f1da0955-5ece-4f98-ab77-f10c20bdd3ca	Institution UWFC - Collection ADULT COLLECTION	No available dataset citation	Unspecified intellectual rights	OBIS (2019) [Institution UWFC - Collection ADULT COLLECTION] (Available: Ocean Biodiversity Information System. Intergovernmental Oceanographic Commission of UNESCO. https://obis.org. Accessed: 2019-08-29)
ff8b7809-41bc-40ad-8160-0e33862817a0	Biodiversity Research Museum, Academia Sinica, Taiwan	TELDAP, Biodiversity Research Museum, Academia Sinica, Taiwan (accessed through GBIF data portal, http://data.gbif.org/datasets/resource/9093, yyyy-mm-dd)	Unrestricted	OBIS (2019) [Biodiversity Research Museum, Academia Sinica, Taiwan] (Available: Ocean Biodiversity Information System. Intergovernmental Oceanographic Commission of UNESCO. https://obis.org. Accessed: 2019-08-29)

Diaphus luetkeni

GBIF

GBIF Occurrence Download https://doi.org/10.15468/dd.2kr2fy Accessed from R via rgbif (https://github.com/ropensci/rgbif) on 2019-09-11

OBIS

OBIS (2019) Distribution records of Diaphus luetkeni [Dataset] (Available: Ocean Biodiversity Information System. Intergovernmental Oceanographic Commission of UNESCO. www.obis.org. Accessed: 2019-08-29)

Dataset details:

Dataset_ID	Name	Citation	License	OBIS_citation
6a5bc28f-4dfe-4cbf-8a55-7e3a843997ab	SPC NECTALIS Zooplankton/Micronekton specimens, New Caledonia 2014	Allain, V., Menkes, C., 2014. Nectalis 3 cruise, RV Alis. https://doi.org/10.17600/14004900	This work is licensed under a Creative Commons Attribution Non Commercial (CC-BY-NC) 4.0 License	OBIS (2019) [SPC NECTALIS Zooplankton/Micronekton specimens, New Caledonia 2014] (Available: Ocean Biodiversity Information System. Intergovernmental Oceanographic Commission of UNESCO. https://obis.org. Accessed: 2019-08-29)
705770e5-3474-4e69-be8b-3107a0c5610a	The fishes collection (IC) of the Muséum national d'Histoire naturelle (MNHN - Paris)	Gicim data base, Pruvost P. Causse R., 2009 http://doi.org/10.15468/tm7whu	This work is licensed under a Creative Commons Attribution (CC-BY) 4.0 License	OBIS (2019) [The fishes collection (IC) of the Muséum national d'Histoire naturelle (MNHN - Paris)] (Available: Ocean Biodiversity Information System. Intergovernmental Oceanographic Commission of UNESCO. https://obis.org. Accessed: 2019-08-29)
8a1ae661-e911-4967-bc06-1168fc5f2d89	iziko South African Museum - Fish Collection	iziko South African Museum - Fish Collection	Restricted	OBIS (2019) [iziko South African Museum - Fish Collection] (Available: Ocean Biodiversity Information System. Intergovernmental Oceanographic Commission of UNESCO. https://obis.org. Accessed: 2019-08-29)
b8617377-eb1c-4db2-baa6-8788a632e810	Ichthyology Collection - Royal Ontario Museum	NA	This work is licensed under a Creative Commons Attribution Non Commercial (CC-BY-NC) 4.0 License	OBIS (2019) [Ichthyology Collection - Royal Ontario Museum] (Available: Ocean Biodiversity Information System. Intergovernmental Oceanographic Commission of UNESCO. https://obis.org. Accessed: 2019-08-29)
c24bf1c2-2c62-4056-a841-56d94e6e876a	Fish specimens	ROM Fish Collection (accessed through GBIF data portal, http://data.gbif.org/datasets/resource/660, 2012-01-20) http://doi.org/10.15468/syisbx	Unrestricted	OBIS (2019) [Fish specimens] (Available: Ocean Biodiversity Information System. Intergovernmental Oceanographic Commission of UNESCO. https://obis.org. Accessed: 2019-08-29)

d6d6fe4c-425f-4ce7-bf28-7a6befaeb413	National Museum of Natural History Vertebrate Zoology Fishes Collections	National Museum of Natural History, Smithsonian Institution NMNH Fishes Collection Database. National Museum of Natural History, Smithsonian Institution, 10th and Constitution Ave. N.W., Washington, DC 20560-0193, 2007.	This work is licensed under a Creative Commons Attribution (CC-BY) 4.0 License	OBIS (2019) [National Museum of Natural History Vertebrate Zoology Fishes Collections] (Available: Ocean Biodiversity Information System. Intergovernmental Oceanographic Commission of UNESCO. https://obis.org. Accessed: 2019-08-29)
ff8b7809-41bc-40ad-8160-0e33862817a0	Biodiversity Research Museum, Academia Sinica, Taiwan	TELDAP, Biodiversity Research Museum, Academia Sinica, Taiwan (accessed through GBIF data portal, http://data.gbif.org/datasets/resource/9093, yyyy-mm-dd)	Unrestricted	OBIS (2019) [Biodiversity Research Museum, Academia Sinica, Taiwan] (Available: Ocean Biodiversity Information System. Intergovernmental Oceanographic Commission of UNESCO. https://obis.org. Accessed: 2019-08-29)

Lepidophanes gaussi

GBIF

GBIF Occurrence Download https://doi.org/10.15468/dd.gnytc5 Accessed from R via rgbif (https://github.com/ropensci/rgbif) on 2019-09-11

OBIS

OBIS (2019) Distribution records of Lepidophanes gaussi [Dataset] (Available: Ocean Biodiversity Information System. Intergovernmental Oceanographic Commission of UNESCO. www.obis.org. Accessed: 2019-08-29)

Dataset details:

Dataset_ID	Name	Citation	License	OBIS_citation

0332e1b5-5525-4301-9659-ef3da3e4e2b6	MARMAP Isaacs-Kidd Midwater Trawl 1990-2009	Marcel Reichert, 2010, MARMAP Isaacs-Kidd Midwater Trawl 1990-2009, SCDNR/NOAA MARMAP Program, SCDNR MARMAP Aggregate data surveys, The Marine Resources Monitoring, Assessment, and Prediction (MARMAP) Program, Marine Resources Research Institute, South Carolina Department of Natural Resources, P. O. Box 12559, Charleston SC 29422-2559, U.S.A.Retrieved from http://www.usgs.gov/obis-usa/	Restricted	OBIS (2019) [MARMAP Isaacs-Kidd Midwater Trawl 1990-2009] (Available: Ocean Biodiversity Information System. Intergovernmental Oceanographic Commission of UNESCO. https://obis.org. Accessed: 2019-08-29)
1f59030f-f116-4c34-915e-1882d819cda3	Institution Southampton Oceanography Ceter - Collection discovery	No available dataset citation	Unspecified intellectual rights	OBIS (2019) [Institution Southampton Oceanography Ceter - Collection discovery] (Available: Ocean Biodiversity Information System. Intergovernmental Oceanographic Commission of UNESCO. https://obis.org. Accessed: 2019-08-29)
2870c548-343e-4575-ac67-a4da35182c52	Institution Shirshov Institute - Collection SKAO	No available dataset citation	Unspecified intellectual rights	OBIS (2019) [Institution Shirshov Institute - Collection SKAO] (Available: Ocean Biodiversity Information System. Intergovernmental Oceanographic Commission of UNESCO. https://obis.org. Accessed: 2019-08-29)
308a501c-a187-498e-a8bd-9cf3d2b70bd9	Institution REVIZEE - Collection Ictioplankton	No available dataset citation	Unspecified intellectual rights	OBIS (2019) [Institution REVIZEE - Collection Ictioplankton] (Available: Ocean Biodiversity Information System. Intergovernmental Oceanographic Commission of UNESCO. https://obis.org. Accessed: 2019-08-29)

513e4437-9cc7-4383-9a69-1bd18ec2046a	MARMAP Neuston Nets 1990-2009	Marcel Reichert, 2010, MARMAP Neuston Nets 1990-2009, SCDNR/NOAA MARMAP Program, SCDNR MARMAP Aggregate data surveys, The Marine Resources Monitoring, Assessment, and Prediction (MARMAP) Program, Marine Resources Research Institute, South Carolina Department of Natural Resources, P. O. Box 12559, Charleston SC 29422-2559, U.S.A.Retrieved from http://www.usgs.gov/obis-usa/	Restricted	OBIS (2019) [MARMAP Neuston Nets 1990-2009] (Available: Ocean Biodiversity Information System. Intergovernmental Oceanographic Commission of UNESCO. https://obis.org. Accessed: 2019-08-29)
5f2da252-6d49-4c9f-b3b3-1db53d75b345	MARMAP Bongo Nets 1990-2009	Marcel Reichert, 2010, MARMAP Bongo Nets 1990-2009, SCDNR/NOAA MARMAP Program, SCDNR MARMAP Aggregate data surveys, The Marine Resources Monitoring, Assessment, and Prediction (MARMAP) Program, Marine Resources Research Institute, South Carolina Department of Natural Resources, P. O. Box 12559, Charleston SC 29422-2559, U.S.A.Retrieved from http://www.usgs.gov/obis-usa/	Restricted	OBIS (2019) [MARMAP Bongo Nets 1990-2009] (Available: Ocean Biodiversity Information System. Intergovernmental Oceanographic Commission of UNESCO. https://obis.org. Accessed: 2019-08-29)
8629ec33-be4b-4384-933f-a511fbc29967	MAR-ECO 2004	Wenneck, T. de Lange, Falkenhaug, T. and O.A. Bergstad. 2008. Strategies, methods, and technologies adopted on the RV G.O. Sars MAR-ECO expedition to the mid-Atlantic Ridge in 2004. Deep-sea Research II. 55: 6-28.	This work is licensed under a Creative Commons Attribution (CC-BY) 4.0 License	OBIS (2019) [MAR-ECO 2004] (Available: Ocean Biodiversity Information System. Intergovernmental Oceanographic Commission of UNESCO. https://obis.org. Accessed: 2019-08-29)
8a1ae661-e911-4967-bc06-1168fc5f2d89	iziko South African Museum - Fish Collection	iziko South African Museum - Fish Collection	Restricted	OBIS (2019) [iziko South African Museum - Fish Collection] (Available: Ocean Biodiversity Information System. Intergovernmental Oceanographic Commission of UNESCO. https://obis.org. Accessed: 2019-08-29)

b8617377-eb1c-4db2-baa6-8788a632e810	Ichthyology Collection - Royal Ontario Museum	NA	This work is licensed under a Creative Commons Attribution Non Commercial (CC-BY-NC) 4.0 License	OBIS (2019) [Ichthyology Collection - Royal Ontario Museum] (Available: Ocean Biodiversity Information System. Intergovernmental Oceanographic Commission of UNESCO. https://obis.org. Accessed: 2019-08-29)
c24bf1c2-2c62-4056-a841-56d94e6e876a	Fish specimens	ROM Fish Collection (accessed through GBIF data portal, http://data.gbif.org/datasets/resource/660, 2012-01-20) http://doi.org/10.15468/syisbx	Unrestricted	OBIS (2019) [Fish specimens] (Available: Ocean Biodiversity Information System. Intergovernmental Oceanographic Commission of UNESCO. https://obis.org. Accessed: 2019-08-29)
cc8f28ce-e48d-4945-abfe-9d150a22dcd6	Hamburg pelagic fish database	Post, A. 1987. Pelagic transects of FRVs "Walther Herwig" and "Anton Dohrn" in the Atlantic Ocean 1966 to 1986. Mitt. Inst. f. Seefischerei d. BfaFi Hamburg, 42: 1-68.	This work is licensed under a Creative Commons Attribution (CC-BY) 4.0 License	OBIS (2019) [Hamburg pelagic fish database] (Available: Ocean Biodiversity Information System. Intergovernmental Oceanographic Commission of UNESCO. https://obis.org. Accessed: 2019-08-29)
ce1d93f3-8b0f-4ee7-9a4d-0393a6ec7fea	Atlantic Reference Centre Museum of Canadian Atlantic Organisms - Invertebrates and Fishes Data	Van Guelpen, L., 2016. Atlantic Reference Centre Museum of Canadian Atlantic Organisms - Invertebrates and Fishes Data. Version 4 In OBIS Canada Digital Collections. Bedford Institute of Oceanography, Dartmouth, NS, Canada. Published by OBIS, Digital http://www.iobis.org/. Accessed on –INSERT DATE	This work is licensed under a Creative Commons Attribution (CC-BY) 4.0 License	OBIS (2019) [Atlantic Reference Centre Museum of Canadian Atlantic Organisms - Invertebrates and Fishes Data] (Available: Ocean Biodiversity Information System. Intergovernmental Oceanographic Commission of UNESCO. https://obis.org. Accessed: 2019-08-29)
d6d6fe4c-425f-4ce7-bf28-7a6befaeb413	National Museum of Natural History Vertebrate Zoology Fishes Collections	National Museum of Natural History, Smithsonian Institution NMNH Fishes Collection Database. National Museum of Natural History, Smithsonian Institution, 10th and Constitution Ave. N.W., Washington, DC 20560-0193, 2007.	This work is licensed under a Creative Commons Attribution (CC-BY) 4.0 License	OBIS (2019) [National Museum of Natural History Vertebrate Zoology Fishes Collections] (Available: Ocean Biodiversity Information System. Intergovernmental Oceanographic Commission of UNESCO. https://obis.org. Accessed: 2019-08-29)

Myctophum asperum

GBIF

GBIF Occurrence Download https://doi.org/10.15468/dd.u67vne Accessed from R via rgbif (https://github.com/ropensci/rgbif) on 2019-09-11

OBIS

OBIS (2019) Distribution records of Myctophum asperum [Dataset] (Available: Ocean Biodiversity Information System. Intergovernmental Oceanographic Commission of UNESCO. www.obis.org. Accessed: 2019-08-29)

Dataset details:

Dataset_ID	Name	Citation	License	OBIS_citation
0332e1b5-5525-4301-9659-ef3da3e4e2b6	MARMAP Isaacs-Kidd Midwater Trawl 1990-2009	Marcel Reichert, 2010, MARMAP Isaacs-Kidd Midwater Trawl 1990-2009, SCDNR/NOAA MARMAP Program, SCDNR MARMAP Aggregate data surveys, The Marine Resources Monitoring, Assessment, and Prediction (MARMAP) Program, Marine Resources Research Institute, South Carolina Department of Natural Resources, P. O. Box 12559, Charleston SC 29422-2559, U.S.A.Retrieved from http://www.usgs.gov/obis-usa/	Restricted	OBIS (2019) [MARMAP Isaacs-Kidd Midwater Trawl 1990-2009] (Available: Ocean Biodiversity Information System. Intergovernmental Oceanographic Commission of UNESCO. https://obis.org. Accessed: 2019-08-29)
04e3fd32-b08b-4806-a016-d2dff52ae55a	Asia-Pacific Dataset	Jintsu-Uchifune, Y., Yamamoto, H. (2016) Marine organism occurrence data of the Asia-Pacific region extracted from literature. Available at https://doi.org/10.48518/00002. Accessed on yyyy-mm-dd.	This work is licensed under a Creative Commons Attribution (CC-BY) 4.0 License	OBIS (2019) [Asia-Pacific Dataset] (Available: Ocean Biodiversity Information System. Intergovernmental Oceanographic Commission of UNESCO. https://obis.org. Accessed: 2019-08-29)
10b213e6-a9c4-459e-a40c-ef9edc461b97	Marine data from the Bernice P. Bishop Museum	Pyle R (2016). Bernice P. Bishop Museum. Version 8.1. Bernice Pauahi Bishop Museum. Occurrence dataset https://doi.org/10.15468/s6ctus accessed via GBIF.org on 2018-11-16.	To the extent possible under law, the publisher has waived all rights to these data and has dedicated them to the Public Domain (CC0 1.0)	OBIS (2019) [Marine data from the Bernice P. Bishop Museum] (Available: Ocean Biodiversity Information System. Intergovernmental Oceanographic Commission of UNESCO. https://obis.org. Accessed: 2019-08-29)
270f3e70-ff9b-411d-b170-2bc914d83f26	Biological Reference Collections ICM CSIC	Olivas González F J (2016): Biological Reference Collections ICM CSIC. Institute of Marine Sciences (ICM-CSIC). https://dx.doi.org/10.15470/qlqqdx	This work is licensed under a Creative Commons Attribution Non Commercial (CC-BY-NC) 4.0 License	OBIS (2019) [Biological Reference Collections ICM CSIC] (Available: Ocean Biodiversity Information System. Intergovernmental Oceanographic Commission of UNESCO. https://obis.org. Accessed: 2019-08-29)

2870c548-343e-4575-ac67-a4da35182c52	Institution Shirshov Institute - Collection SKAO	No available dataset citation	Unspecified intellectual rights	OBIS (2019) [Institution Shirshov Institute - Collection SKAO] (Available: Ocean Biodiversity Information System. Intergovernmental Oceanographic Commission of UNESCO. https://obis.org. Accessed: 2019-08-29)
4354345d-7faf-4376-b326-ffbc04b6b0cd	No available dataset name	No available dataset citation	Unspecified intellectual rights	OBIS (2019) [No available dataset name] (Available: Ocean Biodiversity Information System. Intergovernmental Oceanographic Commission of UNESCO. https://obis.org. Accessed: 2019-08-29)
513e4437-9cc7-4383-9a69-1bd18ec2046a	MARMAP Neuston Nets 1990-2009	Marcel Reichert, 2010, MARMAP Neuston Nets 1990-2009, SCDNR/NOAA MARMAP Program, SCDNR MARMAP Aggregate data surveys, The Marine Resources Monitoring, Assessment, and Prediction (MARMAP) Program, Marine Resources Research Institute, South Carolina Department of Natural Resources, P. O. Box 12559, Charleston SC 29422-2559, U.S.A.Retrieved from http://www.usgs.gov/obis-usa/	Restricted	OBIS (2019) [MARMAP Neuston Nets 1990-2009] (Available: Ocean Biodiversity Information System. Intergovernmental Oceanographic Commission of UNESCO. https://obis.org. Accessed: 2019-08-29)
5f2da252-6d49-4c9f-b3b3-1db53d75b345	MARMAP Bongo Nets 1990-2009	Marcel Reichert, 2010, MARMAP Bongo Nets 1990-2009, SCDNR/NOAA MARMAP Program, SCDNR MARMAP Aggregate data surveys, The Marine Resources Monitoring, Assessment, and Prediction (MARMAP) Program, Marine Resources Research Institute, South Carolina Department of Natural Resources, P. O. Box 12559, Charleston SC 29422-2559, U.S.A.Retrieved from http://www.usgs.gov/obis-usa/	Restricted	OBIS (2019) [MARMAP Bongo Nets 1990-2009] (Available: Ocean Biodiversity Information System. Intergovernmental Oceanographic Commission of UNESCO. https://obis.org. Accessed: 2019-08-29)

685b3956-c37a-433a-b661-2bb7b11cf9f8	Soviet Trawl Fishery Data (New Zealand Waters) 1964-1987	Ministry for Primary Industries (2014). Soviet Fishery Data (New Zealand Waters) 1964-1987. Southwestern Pacific OBIS, National Institute of Water and Atmospheric Research (NIWA), Wellington, New Zealand, 111883 records, Online http://nzobisipt.niwa.co.nz/resource.do?r=mbis_soviettrawl released on November 5, 2014.	This work is licensed under a Creative Commons Attribution (CC-BY) 4.0 License	OBIS (2019) [Soviet Trawl Fishery Data (New Zealand Waters) 1964-1987] (Available: Ocean Biodiversity Information System. Intergovernmental Oceanographic Commission of UNESCO. https://obis.org. Accessed: 2019-08-29)
6a5bc28f-4dfe-4cbf-8a55-7e3a843997ab	SPC NECTALIS Zooplankton/Micronekton specimens, New Caledonia 2014	Allain, V., Menkes, C., 2014. Nectalis 3 cruise, RV Alis. https://doi.org/10.17600/14004900	This work is licensed under a Creative Commons Attribution Non Commercial (CC-BY-NC) 4.0 License	OBIS (2019) [SPC NECTALIS Zooplankton/Micronekton specimens, New Caledonia 2014] (Available: Ocean Biodiversity Information System. Intergovernmental Oceanographic Commission of UNESCO. https://obis.org. Accessed: 2019-08-29)
705770e5-3474-4e69-be8b-3107a0c5610a	The fishes collection (IC) of the Muséum national d'Histoire naturelle (MNHN - Paris)	Gicim data base, Pruvost P. Causse R., 2009 http://doi.org/10.15468/tm7whu	This work is licensed under a Creative Commons Attribution (CC-BY) 4.0 License	OBIS (2019) [The fishes collection (IC) of the Muséum national d'Histoire naturelle (MNHN - Paris)] (Available: Ocean Biodiversity Information System. Intergovernmental Oceanographic Commission of UNESCO. https://obis.org. Accessed: 2019-08-29)
87a421bf-4646-49e3-89b7-409b93f2ac7c	Institution - Collection	No available dataset citation	Unspecified intellectual rights	OBIS (2019) [Institution - Collection] (Available: Ocean Biodiversity Information System. Intergovernmental Oceanographic Commission of UNESCO. https://obis.org. Accessed: 2019-08-29)
87a421bf-4646-49e3-89b7-409b93f2ac7c	Institution UWFC - Collection ADULT COLLECTION	No available dataset citation	Unspecified intellectual rights	OBIS (2019) [Institution UWFC - Collection ADULT COLLECTION] (Available: Ocean Biodiversity Information System. Intergovernmental Oceanographic Commission of UNESCO. https://obis.org. Accessed: 2019-08-29)
8a1ae661-e911-4967-bc06-1168fc5f2d89	iziko South African Museum - Fish Collection	iziko South African Museum - Fish Collection	Restricted	OBIS (2019) [iziko South African Museum - Fish Collection] (Available: Ocean Biodiversity Information System. Intergovernmental Oceanographic Commission of UNESCO. https://obis.org. Accessed: 2019-08-29)

8cb3a754-1d96-4b48-87f2-05468b9b3aae	Bottom Trawl Surveys: Catch and Effort Data For South East Australia, 1898-1996	Novaglio C (2023): Bottom Trawl Surveys: Catch and Effort Data For South East Australia, 1898-1996. v1.10. CSIRO National Collections and Marine Infrastructure (NCMI) Information and Data Centre (IDC). Dataset/Occurrence. https://www.marine.csiro.au/ipt/resource?r=csiro_bottom_trawls_1898_1996&v=1.10	This work is licensed under a Creative Commons Attribution Non Commercial (CC-BY-NC) 4.0 License	OBIS (2019) [Bottom Trawl Surveys: Catch and Effort Data For South East Australia, 1898-1996] (Available: Ocean Biodiversity Information System. Intergovernmental Oceanographic Commission of UNESCO. https://obis.org. Accessed: 2019-08-29)
b8617377-eb1c-4db2-baa6-8788a632e810	Ichthyology Collection - Royal Ontario Museum	NA	This work is licensed under a Creative Commons Attribution Non Commercial (CC-BY-NC) 4.0 License	OBIS (2019) [Ichthyology Collection - Royal Ontario Museum] (Available: Ocean Biodiversity Information System. Intergovernmental Oceanographic Commission of UNESCO. https://obis.org. Accessed: 2019-08-29)
c24bf1c2-2c62-4056-a841-56d94e6e876a	Fish specimens	ROM Fish Collection (accessed through GBIF data portal, http://data.gbif.org/datasets/resource/660, 2012-01-20) http://doi.org/10.15468/syisbx	Unrestricted	OBIS (2019) [Fish specimens] (Available: Ocean Biodiversity Information System. Intergovernmental Oceanographic Commission of UNESCO. https://obis.org. Accessed: 2019-08-29)
ce1d93f3-8b0f-4ee7-9a4d-0393a6ec7fea	Atlantic Reference Centre Museum of Canadian Atlantic Organisms - Invertebrates and Fishes Data	Van Guelpen, L., 2016. Atlantic Reference Centre Museum of Canadian Atlantic Organisms - Invertebrates and Fishes Data. Version 4 In OBIS Canada Digital Collections. Bedford Institute of Oceanography, Dartmouth, NS, Canada. Published by OBIS, Digital http://www.iobis.org/. Accessed on –INSERT DATE	This work is licensed under a Creative Commons Attribution (CC-BY) 4.0 License	OBIS (2019) [Atlantic Reference Centre Museum of Canadian Atlantic Organisms - Invertebrates and Fishes Data] (Available: Ocean Biodiversity Information System. Intergovernmental Oceanographic Commission of UNESCO. https://obis.org. Accessed: 2019-08-29)
cfc56587-48c3-4e3d-9350-3a4d9a28b681	Institution UWFC - Collection ADULT COLLECTION	No available dataset citation	Unspecified intellectual rights	OBIS (2019) [Institution UWFC - Collection ADULT COLLECTION] (Available: Ocean Biodiversity Information System. Intergovernmental Oceanographic Commission of UNESCO. https://obis.org. Accessed: 2019-08-29)

d6d6fe4c-425f-4ce7-bf28-7a6befaeb413	National Museum of Natural History Vertebrate Zoology Fishes Collections	National Museum of Natural History, Smithsonian Institution NMNH Fishes Collection Database. National Museum of Natural History, Smithsonian Institution, 10th and Constitution Ave. N.W., Washington, DC 20560-0193, 2007.	This work is licensed under a Creative Commons Attribution (CC-BY) 4.0 License	OBIS (2019) [National Museum of Natural History Vertebrate Zoology Fishes Collections] (Available: Ocean Biodiversity Information System. Intergovernmental Oceanographic Commission of UNESCO. https://obis.org. Accessed: 2019-08-29)
f1da0955-5ece-4f98-ab77-f10c20bdd3ca	Institution NOAA, NMFS, Northeast Fisheries Science Center - Collection DEEPWATER SYSTEMATICS	No available dataset citation	Unspecified intellectual rights	OBIS (2019) [Institution NOAA, NMFS, Northeast Fisheries Science Center - Collection DEEPWATER SYSTEMATICS] (Available: Ocean Biodiversity Information System. Intergovernmental Oceanographic Commission of UNESCO. https://obis.org. Accessed: 2019-08-29)
ff8b7809-41bc-40ad-8160-0e33862817a0	Biodiversity Research Museum, Academia Sinica, Taiwan	TELDAP, Biodiversity Research Museum, Academia Sinica, Taiwan (accessed through GBIF data portal, http://data.gbif.org/datasets/resource/9093, yyyy-mm-dd)	Unrestricted	OBIS (2019) [Biodiversity Research Museum, Academia Sinica, Taiwan] (Available: Ocean Biodiversity Information System. Intergovernmental Oceanographic Commission of UNESCO. https://obis.org. Accessed: 2019-08-29)

Hygophum proximum

GBIF

GBIF Occurrence Download https://doi.org/10.15468/dd.h358yd Accessed from R via rgbif (https://github.com/ropensci/rgbif) on 2019-09-11

OBIS

OBIS (2019) Distribution records of Hygophum proximum [Dataset] (Available: Ocean Biodiversity Information System. Intergovernmental Oceanographic Commission of UNESCO. www.obis.org. Accessed: 2019-08-29)

Dataset details:

Dataset_ID	Name	Citation	License	OBIS_citation

04e3fd32-b08b-4806-a016-d2dff52ae55a	Asia-Pacific Dataset	Jintsu-Uchifune, Y., Yamamoto, H. (2016) Marine organism occurrence data of the Asia-Pacific region extracted from literature. Available at https://doi.org/10.48518/00002. Accessed on yyyy-mm-dd.	This work is licensed under a Creative Commons Attribution (CC-BY) 4.0 License	OBIS (2019) [Asia-Pacific Dataset] (Available: Ocean Biodiversity Information System. Intergovernmental Oceanographic Commission of UNESCO. https://obis.org. Accessed: 2019-08-29)
10b213e6-a9c4-459e-a40c-ef9edc461b97	Marine data from the Bernice P. Bishop Museum	Pyle R (2016). Bernice P. Bishop Museum. Version 8.1. Bernice Pauahi Bishop Museum. Occurrence dataset https://doi.org/10.15468/s6ctus accessed via GBIF.org on 2018-11-16.	To the extent possible under law, the publisher has waived all rights to these data and has dedicated them to the Public Domain (CC0 1.0)	OBIS (2019) [Marine data from the Bernice P. Bishop Museum] (Available: Ocean Biodiversity Information System. Intergovernmental Oceanographic Commission of UNESCO. https://obis.org. Accessed: 2019-08-29)
1f59030f-f116-4c34-915e-1882d819cda3	Institution Southampton Oceanography Ceter - Collection discovery	No available dataset citation	Unspecified intellectual rights	OBIS (2019) [Institution Southampton Oceanography Ceter - Collection discovery] (Available: Ocean Biodiversity Information System. Intergovernmental Oceanographic Commission of UNESCO. https://obis.org. Accessed: 2019-08-29)
270f3e70-ff9b-411d-b170-2bc914d83f26	Biological Reference Collections ICM CSIC	Olivas González F J (2016): Biological Reference Collections ICM CSIC. Institute of Marine Sciences (ICM-CSIC). https://dx.doi.org/10.15470/qlqqdx	This work is licensed under a Creative Commons Attribution Non Commercial (CC-BY-NC) 4.0 License	OBIS (2019) [Biological Reference Collections ICM CSIC] (Available: Ocean Biodiversity Information System. Intergovernmental Oceanographic Commission of UNESCO. https://obis.org. Accessed: 2019-08-29)
6a5bc28f-4dfe-4cbf-8a55-7e3a843997ab	SPC NECTALIS Zooplankton/Micronekton specimens, New Caledonia 2014	Allain, V., Menkes, C., 2014. Nectalis 3 cruise, RV Alis. https://doi.org/10.17600/14004900	This work is licensed under a Creative Commons Attribution Non Commercial (CC-BY-NC) 4.0 License	OBIS (2019) [SPC NECTALIS Zooplankton/Micronekton specimens, New Caledonia 2014] (Available: Ocean Biodiversity Information System. Intergovernmental Oceanographic Commission of UNESCO. https://obis.org. Accessed: 2019-08-29)
705770e5-3474-4e69-be8b-3107a0c5610a	The fishes collection (IC) of the Muséum national d'Histoire naturelle (MNHN - Paris)	Gicim data base, Pruvost P. Causse R., 2009 http://doi.org/10.15468/tm7whu	This work is licensed under a Creative Commons Attribution (CC-BY) 4.0 License	OBIS (2019) [The fishes collection (IC) of the Muséum national d'Histoire naturelle (MNHN - Paris)] (Available: Ocean Biodiversity Information System. Intergovernmental Oceanographic Commission of UNESCO. https://obis.org. Accessed: 2019-08-29)

87a421bf-4646-49e3-89b7-409b93f2ac7c	Institution UWFC - Collection ADULT COLLECTION	No available dataset citation	Unspecified intellectual rights	OBIS (2019) [Institution UWFC - Collection ADULT COLLECTION] (Available: Ocean Biodiversity Information System. Intergovernmental Oceanographic Commission of UNESCO. https://obis.org. Accessed: 2019-08-29)
8a1ae661-e911-4967-bc06-1168fc5f2d89	iziko South African Museum - Fish Collection	iziko South African Museum - Fish Collection	Restricted	OBIS (2019) [iziko South African Museum - Fish Collection] (Available: Ocean Biodiversity Information System. Intergovernmental Oceanographic Commission of UNESCO. https://obis.org. Accessed: 2019-08-29)
d6d6fe4c-425f-4ce7-bf28-7a6befaeb413	National Museum of Natural History Vertebrate Zoology Fishes Collections	National Museum of Natural History, Smithsonian Institution NMNH Fishes Collection Database. National Museum of Natural History, Smithsonian Institution, 10th and Constitution Ave. N.W., Washington, DC 20560-0193, 2007.	This work is licensed under a Creative Commons Attribution (CC-BY) 4.0 License	OBIS (2019) [National Museum of Natural History Vertebrate Zoology Fishes Collections] (Available: Ocean Biodiversity Information System. Intergovernmental Oceanographic Commission of UNESCO. https://obis.org. Accessed: 2019-08-29)

Diaphus dumerilii

GBIF

GBIF Occurrence Download https://doi.org/10.15468/dd.3z286f Accessed from R via rgbif (https://github.com/ropensci/rgbif) on 2019-09-11

OBIS

OBIS (2019) Distribution records of Diaphus dumerilii [Dataset] (Available: Ocean Biodiversity Information System. Intergovernmental Oceanographic Commission of UNESCO. www.obis.org. Accessed: 2019-08-29)

Dataset details:

Dataset_ID	Name	Citation	License	OBIS_citation

11bc2cb2-a837-4cd1-9450-52e3bb427cf6	ECNASAP - East Coast North America Strategic Assessment	Brown S.K.R., Zwanenburg K., Branton R. (2005). ECNASAP - East Coast North America Strategic Assessment. Version 1 In OBIS Canada Digital Collections. Bedford Institute of Oceanography, Dartmouth, NS, Canada. Published by OBIS, Digital http://www.iobis.org/. Accessed on –INSERT DATE	This work is licensed under a Creative Commons Attribution (CC-BY) 4.0 License	OBIS (2019) [ECNASAP - East Coast North America Strategic Assessment] (Available: Ocean Biodiversity Information System. Intergovernmental Oceanographic Commission of UNESCO. https://obis.org. Accessed: 2019-08-29)
270f3e70-ff9b-411d-b170-2bc914d83f26	Biological Reference Collections ICM CSIC	Olivas González F J (2016): Biological Reference Collections ICM CSIC. Institute of Marine Sciences (ICM-CSIC). https://dx.doi.org/10.15470/qlqqdx	This work is licensed under a Creative Commons Attribution Non Commercial (CC-BY-NC) 4.0 License	OBIS (2019) [Biological Reference Collections ICM CSIC] (Available: Ocean Biodiversity Information System. Intergovernmental Oceanographic Commission of UNESCO. https://obis.org. Accessed: 2019-08-29)
2870c548-343e-4575-ac67-a4da35182c52	Institution Shirshov Institute - Collection SKAO	No available dataset citation	Unspecified intellectual rights	OBIS (2019) [Institution Shirshov Institute - Collection SKAO] (Available: Ocean Biodiversity Information System. Intergovernmental Oceanographic Commission of UNESCO. https://obis.org. Accessed: 2019-08-29)
3d922162-062c-4ad2-bf4a-f2493bd3a95d	Institution Bedford Institute of Oceanography (BIO) - Collection SUMMER	No available dataset citation	Unspecified intellectual rights	OBIS (2019) [Institution Bedford Institute of Oceanography (BIO) - Collection SUMMER] (Available: Ocean Biodiversity Information System. Intergovernmental Oceanographic Commission of UNESCO. https://obis.org. Accessed: 2019-08-29)
4e25e0ce-b17d-4192-9b55-417f1e0c4fc8	Institution KU - Collection KUI	No available dataset citation	Unspecified intellectual rights	OBIS (2019) [Institution KU - Collection KUI] (Available: Ocean Biodiversity Information System. Intergovernmental Oceanographic Commission of UNESCO. https://obis.org. Accessed: 2019-08-29)
6c19184e-c305-4273-8890-6d342d86f865	Institution REVIZEE - Collection Pelagic Fishes	No available dataset citation	Unspecified intellectual rights	OBIS (2019) [Institution REVIZEE - Collection Pelagic Fishes] (Available: Ocean Biodiversity Information System. Intergovernmental Oceanographic Commission of UNESCO. https://obis.org. Accessed: 2019-08-29)

6f44d12a-ce3a-4589-9b3a-69234ee207d1	MARMAP Yankee Trawl 1990-2009	Marcel Reichert, 2010, MARMAP Yankee Trawl 1990-2009, SCDNR/NOAA MARMAP Program, SCDNR MARMAP Aggregate data surveys, The Marine Resources Monitoring, Assessment, and Prediction (MARMAP) Program, Marine Resources Research Institute, South Carolina Department of Natural Resources, P. O. Box 12559, Charleston SC 29422-2559, U.S.A. Retrieve from http://obisusa.nbii.gov	Restricted	OBIS (2019) [MARMAP Yankee Trawl 1990-2009] (Available: Ocean Biodiversity Information System. Intergovernmental Oceanographic Commission of UNESCO. https://obis.org. Accessed: 2019-08-29)
784c3f00-9b0b-4b2d-a0e7-2de304537f8b	Institution TU - Collection Fish	No available dataset citation	Unspecified intellectual rights	OBIS (2019) [Institution TU - Collection Fish] (Available: Ocean Biodiversity Information System. Intergovernmental Oceanographic Commission of UNESCO. https://obis.org. Accessed: 2019-08-29)
8a1ae661-e911-4967-bc06-1168fc5f2d89	iziko South African Museum - Fish Collection	iziko South African Museum - Fish Collection	Restricted	OBIS (2019) [iziko South African Museum - Fish Collection] (Available: Ocean Biodiversity Information System. Intergovernmental Oceanographic Commission of UNESCO. https://obis.org. Accessed: 2019-08-29)
b8617377-eb1c-4db2-baa6-8788a632e810	Ichthyology Collection - Royal Ontario Museum	NA	This work is licensed under a Creative Commons Attribution Non Commercial (CC-BY-NC) 4.0 License	OBIS (2019) [Ichthyology Collection - Royal Ontario Museum] (Available: Ocean Biodiversity Information System. Intergovernmental Oceanographic Commission of UNESCO. https://obis.org. Accessed: 2019-08-29)
c24bf1c2-2c62-4056-a841-56d94e6e876a	Fish specimens	ROM Fish Collection (accessed through GBIF data portal, http://data.gbif.org/datasets/resource/660, 2012-01-20) http://doi.org/10.15468/syisbx	Unrestricted	OBIS (2019) [Fish specimens] (Available: Ocean Biodiversity Information System. Intergovernmental Oceanographic Commission of UNESCO. https://obis.org. Accessed: 2019-08-29)
cc8f28ce-e48d-4945-abfe-9d150a22dcd6	Hamburg pelagic fish database	Post, A. 1987. Pelagic transects of FRVs "Walther Herwig" and "Anton Dohrn" in the Atlantic Ocean 1966 to 1986. Mitt. Inst. f. Seefischerei d. BfaFi Hamburg, 42: 1-68.	This work is licensed under a Creative Commons Attribution (CC-BY) 4.0 License	OBIS (2019) [Hamburg pelagic fish database] (Available: Ocean Biodiversity Information System. Intergovernmental Oceanographic Commission of UNESCO. https://obis.org. Accessed: 2019-08-29)

ce1d93f3-8b0f-4ee7-9a4d-0393a6ec7fea	Atlantic Reference Centre Museum of Canadian Atlantic Organisms - Invertebrates and Fishes Data	Van Guelpen, L., 2016. Atlantic Reference Centre Museum of Canadian Atlantic Organisms - Invertebrates and Fishes Data. Version 4 In OBIS Canada Digital Collections. Bedford Institute of Oceanography, Dartmouth, NS, Canada. Published by OBIS, Digital http://www.iobis.org/. Accessed on –INSERT DATE	This work is licensed under a Creative Commons Attribution (CC-BY) 4.0 License	OBIS (2019) [Atlantic Reference Centre Museum of Canadian Atlantic Organisms - Invertebrates and Fishes Data] (Available: Ocean Biodiversity Information System. Intergovernmental Oceanographic Commission of UNESCO. https://obis.org. Accessed: 2019-08-29)
cfc56587-48c3-4e3d-9350-3a4d9a28b681	Institution NOAA, NMFS, Northeast Fisheries Science Center - Collection DEEPWATER SYSTEMATICS	No available dataset citation	Unspecified intellectual rights	OBIS (2019) [Institution NOAA, NMFS, Northeast Fisheries Science Center - Collection DEEPWATER SYSTEMATICS] (Available: Ocean Biodiversity Information System. Intergovernmental Oceanographic Commission of UNESCO. https://obis.org. Accessed: 2019-08-29)
cfc56587-48c3-4e3d-9350-3a4d9a28b681	Institution NOAA, NMFS, Northeast Fisheries Science Center - Collection SUMMER NMFS NEFSC BOTTOM TRAWL SURVEY	No available dataset citation	Unspecified intellectual rights	OBIS (2019) [Institution NOAA, NMFS, Northeast Fisheries Science Center - Collection SUMMER NMFS NEFSC BOTTOM TRAWL SURVEY] (Available: Ocean Biodiversity Information System. Intergovernmental Oceanographic Commission of UNESCO. https://obis.org. Accessed: 2019-08-29)
cfc56587-48c3-4e3d-9350-3a4d9a28b681	Institution NOAA, NMFS, Northeast Fisheries Science Center - Collection SPRING NMFS NEFSC BOTTOM TRAWL SURVEY	No available dataset citation	Unspecified intellectual rights	OBIS (2019) [Institution NOAA, NMFS, Northeast Fisheries Science Center - Collection SPRING NMFS NEFSC BOTTOM TRAWL SURVEY] (Available: Ocean Biodiversity Information System. Intergovernmental Oceanographic Commission of UNESCO. https://obis.org. Accessed: 2019-08-29)
d286ae50-ea29-4aa4-8028-2e6e5945a039	Institution REVIZEE - Collection Demersal Fishes	No available dataset citation	Unspecified intellectual rights	OBIS (2019) [Institution REVIZEE - Collection Demersal Fishes] (Available: Ocean Biodiversity Information System. Intergovernmental Oceanographic Commission of UNESCO. https://obis.org. Accessed: 2019-08-29)

d6d6fe4c-425f-4ce7-bf28-7a6befaeb413	National Museum of Natural History Vertebrate Zoology Fishes Collections	National Museum of Natural History, Smithsonian Institution NMNH Fishes Collection Database. National Museum of Natural History, Smithsonian Institution, 10th and Constitution Ave. N.W., Washington, DC 20560-0193, 2007.	This work is licensed under a Creative Commons Attribution (CC-BY) 4.0 License	OBIS (2019) [National Museum of Natural History Vertebrate Zoology Fishes Collections] (Available: Ocean Biodiversity Information System. Intergovernmental Oceanographic Commission of UNESCO. https://obis.org. Accessed: 2019-08-29)

Diogenichthys panurgus

GBIF

GBIF Occurrence Download https://doi.org/10.15468/dd.v6sdj9 Accessed from R via rgbif (https://github.com/ropensci/rgbif) on 2019-09-11

OBIS

OBIS (2019) Distribution records of Diogenichthys panurgus [Dataset] (Available: Ocean Biodiversity Information System. Intergovernmental Oceanographic Commission of UNESCO. www.obis.org. Accessed: 2019-08-29)

Dataset details:

Dataset_ID	Name	Citation	License	OBIS_citation
1f59030f-f116-4c34-915e-1882d819cda3	Institution Southampton Oceanography Ceter - Collection discovery	No available dataset citation	Unspecified intellectual rights	OBIS (2019) [Institution Southampton Oceanography Ceter - Collection discovery] (Available: Ocean Biodiversity Information System. Intergovernmental Oceanographic Commission of UNESCO. https://obis.org. Accessed: 2019-08-29)
270f3e70-ff9b-411d-b170-2bc914d83f26	Biological Reference Collections ICM CSIC	Olivas González F J (2016): Biological Reference Collections ICM CSIC. Institute of Marine Sciences (ICM-CSIC). https://dx.doi.org/10.15470/qlqqdx	This work is licensed under a Creative Commons Attribution Non Commercial (CC-BY-NC) 4.0 License	OBIS (2019) [Biological Reference Collections ICM CSIC] (Available: Ocean Biodiversity Information System. Intergovernmental Oceanographic Commission of UNESCO. https://obis.org. Accessed: 2019-08-29)

4bdc1f6f-e16a-48b4-b995-b51bd41caa8d	Dataset of the multidisciplinary research surveys in the seamounts of Ewing and Valdivia Bank (Walvis Ridge) - SE Atlantic	López-Abellán, L. J.; Sarralde Vizuete, R.; González Jiménez, J. F.; Centro Oceanográfico de Canarias – IEO, Spain (2015). Dataset of the multidisciplinary research surveys in the seamounts of Ewing and Valdivia Bank (Walvis Ridge) - SE Atlantic https://dx.doi.org/10.14284/58	Attribution-NoDerivatives (CC BY-ND)	OBIS (2019) [Dataset of the multidisciplinary research surveys in the seamounts of Ewing and Valdivia Bank (Walvis Ridge) - SE Atlantic] (Available: Ocean Biodiversity Information System. Intergovernmental Oceanographic Commission of UNESCO. https://obis.org. Accessed: 2019-08-29)
705770e5-3474-4e69-be8b-3107a0c5610a	The fishes collection (IC) of the Muséum national d'Histoire naturelle (MNHN - Paris)	Gicim data base, Pruvost P. Causse R., 2009 http://doi.org/10.15468/tm7whu	This work is licensed under a Creative Commons Attribution (CC-BY) 4.0 License	OBIS (2019) [The fishes collection (IC) of the Muséum national d'Histoire naturelle (MNHN - Paris)] (Available: Ocean Biodiversity Information System. Intergovernmental Oceanographic Commission of UNESCO. https://obis.org. Accessed: 2019-08-29)
8a1ae661-e911-4967-bc06-1168fc5f2d89	iziko South African Museum - Fish Collection	iziko South African Museum - Fish Collection	Restricted	OBIS (2019) [iziko South African Museum - Fish Collection] (Available: Ocean Biodiversity Information System. Intergovernmental Oceanographic Commission of UNESCO. https://obis.org. Accessed: 2019-08-29)
a4f7ee48-0d0b-4c05-a972-27a43b30db58	Institution MCM - Collection DEM	No available dataset citation	Unspecified intellectual rights	OBIS (2019) [Institution MCM - Collection DEM] (Available: Ocean Biodiversity Information System. Intergovernmental Oceanographic Commission of UNESCO. https://obis.org. Accessed: 2019-08-29)
ff8b7809-41bc-40ad-8160-0e33862817a0	Biodiversity Research Museum, Academia Sinica, Taiwan	TELDAP, Biodiversity Research Museum, Academia Sinica, Taiwan (accessed through GBIF data portal, http://data.gbif.org/datasets/resource/9093, yyyy-mm-dd)	Unrestricted	OBIS (2019) [Biodiversity Research Museum, Academia Sinica, Taiwan] (Available: Ocean Biodiversity Information System. Intergovernmental Oceanographic Commission of UNESCO. https://obis.org. Accessed: 2019-08-29)

Diaphus parri

GBIF

GBIF Occurrence Download https://doi.org/10.15468/dd.bnmw87 Accessed from R via rgbif (https://github.com/ropensci/rgbif) on 2019-09-11

OBIS

OBIS (2019) Distribution records of Diaphus parri [Dataset] (Available: Ocean Biodiversity Information System. Intergovernmental Oceanographic Commission of UNESCO. www.obis.org. Accessed: 2019-08-29)

Dataset details:

Dataset_ID	Name	Citation	License	OBIS_citation
1f59030f-f116-4c34-915e-1882d819cda3	Institution Southampton Oceanography Ceter - Collection discovery	No available dataset citation	Unspecified intellectual rights	OBIS (2019) [Institution Southampton Oceanography Ceter - Collection discovery] (Available: Ocean Biodiversity Information System. Intergovernmental Oceanographic Commission of UNESCO. https://obis.org. Accessed: 2019-08-29)
6a5bc28f-4dfe-4cbf-8a55-7e3a843997ab	SPC NECTALIS Zooplankton/Micronekton specimens, New Caledonia 2014	Allain, V., Menkes, C., 2014. Nectalis 3 cruise, RV Alis. https://doi.org/10.17600/14004900	This work is licensed under a Creative Commons Attribution Non Commercial (CC-BY-NC) 4.0 License	OBIS (2019) [SPC NECTALIS Zooplankton/Micronekton specimens, New Caledonia 2014] (Available: Ocean Biodiversity Information System. Intergovernmental Oceanographic Commission of UNESCO. https://obis.org. Accessed: 2019-08-29)
705770e5-3474-4e69-be8b-3107a0c5610a	The fishes collection (IC) of the Muséum national d'Histoire naturelle (MNHN - Paris)	Gicim data base, Pruvost P. Causse R., 2009 http://doi.org/10.15468/tm7whu	This work is licensed under a Creative Commons Attribution (CC-BY) 4.0 License	OBIS (2019) [The fishes collection (IC) of the Muséum national d'Histoire naturelle (MNHN - Paris)] (Available: Ocean Biodiversity Information System. Intergovernmental Oceanographic Commission of UNESCO. https://obis.org. Accessed: 2019-08-29)
8a1ae661-e911-4967-bc06-1168fc5f2d89	iziko South African Museum - Fish Collection	iziko South African Museum - Fish Collection	Restricted	OBIS (2019) [iziko South African Museum - Fish Collection] (Available: Ocean Biodiversity Information System. Intergovernmental Oceanographic Commission of UNESCO. https://obis.org. Accessed: 2019-08-29)
9ff216fc-777e-4f9b-9860-95ed7366870d	Institution SAIAB - Collection SAIAB	No available dataset citation	Unspecified intellectual rights	OBIS (2019) [Institution SAIAB - Collection SAIAB] (Available: Ocean Biodiversity Information System. Intergovernmental Oceanographic Commission of UNESCO. https://obis.org. Accessed: 2019-08-29)

d6d6fe4c-425f-4ce7-bf28-7a6befaeb413	National Museum of Natural History Vertebrate Zoology Fishes Collections	National Museum of Natural History, Smithsonian Institution NMNH Fishes Collection Database. National Museum of Natural History, Smithsonian Institution, 10th and Constitution Ave. N.W., Washington, DC 20560-0193, 2007.	This work is licensed under a Creative Commons Attribution (CC-BY) 4.0 License	OBIS (2019) [National Museum of Natural History Vertebrate Zoology Fishes Collections] (Available: Ocean Biodiversity Information System. Intergovernmental Oceanographic Commission of UNESCO. https://obis.org. Accessed: 2019-08-29)

Bolinichthys photothorax

GBIF

GBIF Occurrence Download https://doi.org/10.15468/dd.3wqv8h Accessed from R via rgbif (https://github.com/ropensci/rgbif) on 2019-09-11

OBIS

OBIS (2019) Distribution records of Bolinichthys photothorax [Dataset] (Available: Ocean Biodiversity Information System. Intergovernmental Oceanographic Commission of UNESCO. www.obis.org. Accessed: 2019-08-29)

Dataset details:

Dataset_ID	Name	Citation	License	OBIS_citation
10b213e6-a9c4-459e-a40c-ef9edc461b97	Marine data from the Bernice P. Bishop Museum	Pyle R (2016). Bernice P. Bishop Museum. Version 8.1. Bernice Pauahi Bishop Museum. Occurrence dataset https://doi.org/10.15468/s6ctus accessed via GBIF.org on 2018-11-16.	To the extent possible under law, the publisher has waived all rights to these data and has dedicated them to the Public Domain (CC0 1.0)	OBIS (2019) [Marine data from the Bernice P. Bishop Museum] (Available: Ocean Biodiversity Information System. Intergovernmental Oceanographic Commission of UNESCO. https://obis.org. Accessed: 2019-08-29)
2870c548-343e-4575-ac67-a4da35182c52	Institution Shirshov Institute - Collection SKAO	No available dataset citation	Unspecified intellectual rights	OBIS (2019) [Institution Shirshov Institute - Collection SKAO] (Available: Ocean Biodiversity Information System. Intergovernmental Oceanographic Commission of UNESCO. https://obis.org. Accessed: 2019-08-29)

5f2da252-6d49-4c9f-b3b3-1db53d75b345	MARMAP Bongo Nets 1990-2009	Marcel Reichert, 2010, MARMAP Bongo Nets 1990-2009, SCDNR/NOAA MARMAP Program, SCDNR MARMAP Aggregate data surveys, The Marine Resources Monitoring, Assessment, and Prediction (MARMAP) Program, Marine Resources Research Institute, South Carolina Department of Natural Resources, P. O. Box 12559, Charleston SC 29422-2559, U.S.A.Retrieved from http://www.usgs.gov/obis-usa/	Restricted	OBIS (2019) [MARMAP Bongo Nets 1990-2009] (Available: Ocean Biodiversity Information System. Intergovernmental Oceanographic Commission of UNESCO. https://obis.org. Accessed: 2019-08-29)
705770e5-3474-4e69-be8b-3107a0c5610a	The fishes collection (IC) of the Muséum national d'Histoire naturelle (MNHN - Paris)	Gicim data base, Pruvost P. Causse R., 2009 http://doi.org/10.15468/tm7whu	This work is licensed under a Creative Commons Attribution (CC-BY) 4.0 License	OBIS (2019) [The fishes collection (IC) of the Muséum national d'Histoire naturelle (MNHN - Paris)] (Available: Ocean Biodiversity Information System. Intergovernmental Oceanographic Commission of UNESCO. https://obis.org. Accessed: 2019-08-29)
cc8f28ce-e48d-4945-abfe-9d150a22dcd6	Hamburg pelagic fish database	Post, A. 1987. Pelagic transects of FRVs "Walther Herwig" and "Anton Dohrn" in the Atlantic Ocean 1966 to 1986. Mitt. Inst. f. Seefischerei d. BfaFi Hamburg, 42: 1-68.	This work is licensed under a Creative Commons Attribution (CC-BY) 4.0 License	OBIS (2019) [Hamburg pelagic fish database] (Available: Ocean Biodiversity Information System. Intergovernmental Oceanographic Commission of UNESCO. https://obis.org. Accessed: 2019-08-29)
ce1d93f3-8b0f-4ee7-9a4d-0393a6ec7fea	Atlantic Reference Centre Museum of Canadian Atlantic Organisms - Invertebrates and Fishes Data	Van Guelpen, L., 2016. Atlantic Reference Centre Museum of Canadian Atlantic Organisms - Invertebrates and Fishes Data. Version 4 In OBIS Canada Digital Collections. Bedford Institute of Oceanography, Dartmouth, NS, Canada. Published by OBIS, Digital http://www.iobis.org/. Accessed on –INSERT DATE	This work is licensed under a Creative Commons Attribution (CC-BY) 4.0 License	OBIS (2019) [Atlantic Reference Centre Museum of Canadian Atlantic Organisms - Invertebrates and Fishes Data] (Available: Ocean Biodiversity Information System. Intergovernmental Oceanographic Commission of UNESCO. https://obis.org. Accessed: 2019-08-29)

d6d6fe4c-425f-4ce7-bf28-7a6befaeb413	National Museum of Natural History Vertebrate Zoology Fishes Collections	National Museum of Natural History, Smithsonian Institution NMNH Fishes Collection Database. National Museum of Natural History, Smithsonian Institution, 10th and Constitution Ave. N.W., Washington, DC 20560-0193, 2007.	This work is licensed under a Creative Commons Attribution (CC-BY) 4.0 License	OBIS (2019) [National Museum of Natural History Vertebrate Zoology Fishes Collections] (Available: Ocean Biodiversity Information System. Intergovernmental Oceanographic Commission of UNESCO. https://obis.org. Accessed: 2019-08-29)

Diaphus lucidus

GBIF

GBIF Occurrence Download https://doi.org/10.15468/dd.zet7ap Accessed from R via rgbif (https://github.com/ropensci/rgbif) on 2019-09-11

OBIS

OBIS (2019) Distribution records of Diaphus lucidus [Dataset] (Available: Ocean Biodiversity Information System. Intergovernmental Oceanographic Commission of UNESCO. www.obis.org. Accessed: 2019-08-29)

Dataset details:

Dataset_ID	Name	Citation	License	OBIS_citation
10b213e6-a9c4-459e-a40c-ef9edc461b97	Marine data from the Bernice P. Bishop Museum	Pyle R (2016). Bernice P. Bishop Museum. Version 8.1. Bernice Pauahi Bishop Museum. Occurrence dataset https://doi.org/10.15468/s6ctus accessed via GBIF.org on 2018-11-16.	To the extent possible under law, the publisher has waived all rights to these data and has dedicated them to the Public Domain (CC0 1.0)	OBIS (2019) [Marine data from the Bernice P. Bishop Museum] (Available: Ocean Biodiversity Information System. Intergovernmental Oceanographic Commission of UNESCO. https://obis.org. Accessed: 2019-08-29)
1f59030f-f116-4c34-915e-1882d819cda3	Institution Southampton Oceanography Ceter - Collection discovery	No available dataset citation	Unspecified intellectual rights	OBIS (2019) [Institution Southampton Oceanography Ceter - Collection discovery] (Available: Ocean Biodiversity Information System. Intergovernmental Oceanographic Commission of UNESCO. https://obis.org. Accessed: 2019-08-29)

2870c548-343e-4575-ac67-a4da35182c52	Institution Shirshov Institute - Collection SKAO	No available dataset citation	Unspecified intellectual rights	OBIS (2019) [Institution Shirshov Institute - Collection SKAO] (Available: Ocean Biodiversity Information System. Intergovernmental Oceanographic Commission of UNESCO. https://obis.org. Accessed: 2019-08-29)
705770e5-3474-4e69-be8b-3107a0c5610a	The fishes collection (IC) of the Muséum national d'Histoire naturelle (MNHN - Paris)	Gicim data base, Pruvost P. Causse R., 2009 http://doi.org/10.15468/tm7whu	This work is licensed under a Creative Commons Attribution (CC-BY) 4.0 License	OBIS (2019) [The fishes collection (IC) of the Muséum national d'Histoire naturelle (MNHN - Paris)] (Available: Ocean Biodiversity Information System. Intergovernmental Oceanographic Commission of UNESCO. https://obis.org. Accessed: 2019-08-29)
8a1ae661-e911-4967-bc06-1168fc5f2d89	iziko South African Museum - Fish Collection	iziko South African Museum - Fish Collection	Restricted	OBIS (2019) [iziko South African Museum - Fish Collection] (Available: Ocean Biodiversity Information System. Intergovernmental Oceanographic Commission of UNESCO. https://obis.org. Accessed: 2019-08-29)
9ff216fc-777e-4f9b-9860-95ed7366870d	Institution SAIAB - Collection SAIAB	No available dataset citation	Unspecified intellectual rights	OBIS (2019) [Institution SAIAB - Collection SAIAB] (Available: Ocean Biodiversity Information System. Intergovernmental Oceanographic Commission of UNESCO. https://obis.org. Accessed: 2019-08-29)
a4f7ee48-0d0b-4c05-a972-27a43b30db58	Institution MCM - Collection DEM	No available dataset citation	Unspecified intellectual rights	OBIS (2019) [Institution MCM - Collection DEM] (Available: Ocean Biodiversity Information System. Intergovernmental Oceanographic Commission of UNESCO. https://obis.org. Accessed: 2019-08-29)
b8617377-eb1c-4db2-baa6-8788a632e810	Ichthyology Collection - Royal Ontario Museum	NA	This work is licensed under a Creative Commons Attribution Non Commercial (CC-BY-NC) 4.0 License	OBIS (2019) [Ichthyology Collection - Royal Ontario Museum] (Available: Ocean Biodiversity Information System. Intergovernmental Oceanographic Commission of UNESCO. https://obis.org. Accessed: 2019-08-29)
c24bf1c2-2c62-4056-a841-56d94e6e876a	Fish specimens	ROM Fish Collection (accessed through GBIF data portal, http://data.gbif.org/datasets/resource/660, 2012-01-20) http://doi.org/10.15468/syisbx	Unrestricted	OBIS (2019) [Fish specimens] (Available: Ocean Biodiversity Information System. Intergovernmental Oceanographic Commission of UNESCO. https://obis.org. Accessed: 2019-08-29)

cc8f28ce-e48d-4945-abfe-9d150a22dcd6	Hamburg pelagic fish database	Post, A. 1987. Pelagic transects of FRVs "Walther Herwig" and "Anton Dohrn" in the Atlantic Ocean 1966 to 1986. Mitt. Inst. f. Seefischerei d. BfaFi Hamburg, 42: 1-68.	This work is licensed under a Creative Commons Attribution (CC-BY) 4.0 License	OBIS (2019) [Hamburg pelagic fish database] (Available: Ocean Biodiversity Information System. Intergovernmental Oceanographic Commission of UNESCO. https://obis.org. Accessed: 2019-08-29)
ce1d93f3-8b0f-4ee7-9a4d-0393a6ec7fea	Atlantic Reference Centre Museum of Canadian Atlantic Organisms - Invertebrates and Fishes Data	Van Guelpen, L., 2016. Atlantic Reference Centre Museum of Canadian Atlantic Organisms - Invertebrates and Fishes Data. Version 4 In OBIS Canada Digital Collections. Bedford Institute of Oceanography, Dartmouth, NS, Canada. Published by OBIS, Digital http://www.iobis.org/. Accessed on –INSERT DATE	This work is licensed under a Creative Commons Attribution (CC-BY) 4.0 License	OBIS (2019) [Atlantic Reference Centre Museum of Canadian Atlantic Organisms - Invertebrates and Fishes Data] (Available: Ocean Biodiversity Information System. Intergovernmental Oceanographic Commission of UNESCO. https://obis.org. Accessed: 2019-08-29)
cfc56587-48c3-4e3d-9350-3a4d9a28b681	Institution NOAA, NMFS, Northeast Fisheries Science Center - Collection DEEPWATER SYSTEMATICS	No available dataset citation	Unspecified intellectual rights	OBIS (2019) [Institution NOAA, NMFS, Northeast Fisheries Science Center - Collection DEEPWATER SYSTEMATICS] (Available: Ocean Biodiversity Information System. Intergovernmental Oceanographic Commission of UNESCO. https://obis.org. Accessed: 2019-08-29)
d6d6fe4c-425f-4ce7-bf28-7a6befaeb413	National Museum of Natural History Vertebrate Zoology Fishes Collections	National Museum of Natural History, Smithsonian Institution NMNH Fishes Collection Database. National Museum of Natural History, Smithsonian Institution, 10th and Constitution Ave. N.W., Washington, DC 20560-0193, 2007.	This work is licensed under a Creative Commons Attribution (CC-BY) 4.0 License	OBIS (2019) [National Museum of Natural History Vertebrate Zoology Fishes Collections] (Available: Ocean Biodiversity Information System. Intergovernmental Oceanographic Commission of UNESCO. https://obis.org. Accessed: 2019-08-29)
ff8b7809-41bc-40ad-8160-0e33862817a0	Biodiversity Research Museum, Academia Sinica, Taiwan	TELDAP, Biodiversity Research Museum, Academia Sinica, Taiwan (accessed through GBIF data portal, http://data.gbif.org/datasets/resource/9093, yyyy-mm-dd)	Unrestricted	OBIS (2019) [Biodiversity Research Museum, Academia Sinica, Taiwan] (Available: Ocean Biodiversity Information System. Intergovernmental Oceanographic Commission of UNESCO. https://obis.org. Accessed: 2019-08-29)

Taaningichthys minimus

GBIF

GBIF Occurrence Download https://doi.org/10.15468/dd.m772za Accessed from R via rgbif (https://github.com/ropensci/rgbif) on 2019-09-11

OBIS

OBIS (2019) Distribution records of Taaningichthys minimus [Dataset] (Available: Ocean Biodiversity Information System. Intergovernmental Oceanographic Commission of UNESCO. www.obis.org. Accessed: 2019-08-29)

Dataset details:

Dataset_ID	Name	Citation	License	OBIS_citation
10b213e6-a9c4-459e-a40c-ef9edc461b97	Marine data from the Bernice P. Bishop Museum	Pyle R (2016). Bernice P. Bishop Museum. Version 8.1. Bernice Pauahi Bishop Museum. Occurrence dataset https://doi.org/10.15468/s6ctus accessed via GBIF.org on 2018-11-16.	To the extent possible under law, the publisher has waived all rights to these data and has dedicated them to the Public Domain (CC0 1.0)	OBIS (2019) [Marine data from the Bernice P. Bishop Museum] (Available: Ocean Biodiversity Information System. Intergovernmental Oceanographic Commission of UNESCO. https://obis.org. Accessed: 2019-08-29)
1f59030f-f116-4c34-915e-1882d819cda3	Institution Southampton Oceanography Ceter - Collection discovery	No available dataset citation	Unspecified intellectual rights	OBIS (2019) [Institution Southampton Oceanography Ceter - Collection discovery] (Available: Ocean Biodiversity Information System. Intergovernmental Oceanographic Commission of UNESCO. https://obis.org. Accessed: 2019-08-29)
3d922162-062c-4ad2-bf4a-f2493bd3a95d	Institution Bedford Institute of Oceanography (BIO) - Collection SUMMER	No available dataset citation	Unspecified intellectual rights	OBIS (2019) [Institution Bedford Institute of Oceanography (BIO) - Collection SUMMER] (Available: Ocean Biodiversity Information System. Intergovernmental Oceanographic Commission of UNESCO. https://obis.org. Accessed: 2019-08-29)
8a1ae661-e911-4967-bc06-1168fc5f2d89	iziko South African Museum - Fish Collection	iziko South African Museum - Fish Collection	Restricted	OBIS (2019) [iziko South African Museum - Fish Collection] (Available: Ocean Biodiversity Information System. Intergovernmental Oceanographic Commission of UNESCO. https://obis.org. Accessed: 2019-08-29)

b8617377-eb1c-4db2-baa6-8788a632e810	Ichthyology Collection - Royal Ontario Museum	NA	This work is licensed under a Creative Commons Attribution Non Commercial (CC-BY-NC) 4.0 License	OBIS (2019) [Ichthyology Collection - Royal Ontario Museum] (Available: Ocean Biodiversity Information System. Intergovernmental Oceanographic Commission of UNESCO. https://obis.org. Accessed: 2019-08-29)
c24bf1c2-2c62-4056-a841-56d94e6e876a	Fish specimens	ROM Fish Collection (accessed through GBIF data portal, http://data.gbif.org/datasets/resource/660, 2012-01-20) http://doi.org/10.15468/syisbx	Unrestricted	OBIS (2019) [Fish specimens] (Available: Ocean Biodiversity Information System. Intergovernmental Oceanographic Commission of UNESCO. https://obis.org. Accessed: 2019-08-29)
cc8f28ce-e48d-4945-abfe-9d150a22dcd6	Hamburg pelagic fish database	Post, A. 1987. Pelagic transects of FRVs "Walther Herwig" and "Anton Dohrn" in the Atlantic Ocean 1966 to 1986. Mitt. Inst. f. Seefischerei d. BfaFi Hamburg, 42: 1-68.	This work is licensed under a Creative Commons Attribution (CC-BY) 4.0 License	OBIS (2019) [Hamburg pelagic fish database] (Available: Ocean Biodiversity Information System. Intergovernmental Oceanographic Commission of UNESCO. https://obis.org. Accessed: 2019-08-29)
ce1d93f3-8b0f-4ee7-9a4d-0393a6ec7fea	Atlantic Reference Centre Museum of Canadian Atlantic Organisms - Invertebrates and Fishes Data	Van Guelpen, L., 2016. Atlantic Reference Centre Museum of Canadian Atlantic Organisms - Invertebrates and Fishes Data. Version 4 In OBIS Canada Digital Collections. Bedford Institute of Oceanography, Dartmouth, NS, Canada. Published by OBIS, Digital http://www.iobis.org/. Accessed on –INSERT DATE	This work is licensed under a Creative Commons Attribution (CC-BY) 4.0 License	OBIS (2019) [Atlantic Reference Centre Museum of Canadian Atlantic Organisms - Invertebrates and Fishes Data] (Available: Ocean Biodiversity Information System. Intergovernmental Oceanographic Commission of UNESCO. https://obis.org. Accessed: 2019-08-29)
cfc56587-48c3-4e3d-9350-3a4d9a28b681	Institution NOAA, NMFS, Northeast Fisheries Science Center - Collection DEEPWATER SYSTEMATICS	No available dataset citation	Unspecified intellectual rights	OBIS (2019) [Institution NOAA, NMFS, Northeast Fisheries Science Center - Collection DEEPWATER SYSTEMATICS] (Available: Ocean Biodiversity Information System. Intergovernmental Oceanographic Commission of UNESCO. https://obis.org. Accessed: 2019-08-29)
d6d6fe4c-425f-4ce7-bf28-7a6befaeb413	National Museum of Natural History Vertebrate Zoology Fishes Collections	National Museum of Natural History, Smithsonian Institution NMNH Fishes Collection Database. National Museum of Natural History, Smithsonian Institution, 10th and Constitution Ave. N.W., Washington, DC 20560-0193, 2007.	This work is licensed under a Creative Commons Attribution (CC-BY) 4.0 License	OBIS (2019) [National Museum of Natural History Vertebrate Zoology Fishes Collections] (Available: Ocean Biodiversity Information System. Intergovernmental Oceanographic Commission of UNESCO. https://obis.org. Accessed: 2019-08-29)

ff8b7809-41bc-40ad-8160-0e33862817a0	Biodiversity Research Museum, Academia Sinica, Taiwan	TELDAP, Biodiversity Research Museum, Academia Sinica, Taiwan (accessed through GBIF data portal, http://data.gbif.org/datasets/resource/9093, yyyy-mm-dd)	Unrestricted	OBIS (2019) [Biodiversity Research Museum, Academia Sinica, Taiwan] (Available: Ocean Biodiversity Information System. Intergovernmental Oceanographic Commission of UNESCO. https://obis.org. Accessed: 2019-08-29)

Diaphus termophilus

GBIF

GBIF Occurrence Download https://doi.org/10.15468/dd.qbhhdv Accessed from R via rgbif (https://github.com/ropensci/rgbif) on 2019-09-11

OBIS

OBIS (2019) Distribution records of Diaphus termophilus [Dataset] (Available: Ocean Biodiversity Information System. Intergovernmental Oceanographic Commission of UNESCO. www.obis.org. Accessed: 2019-08-29)

Dataset details:

Dataset_ID	Name	Citation	License	OBIS_citation
0332e1b5-5525-4301-9659-ef3da3e4e2b6	MARMAP Isaacs-Kidd Midwater Trawl 1990-2009	Marcel Reichert, 2010, MARMAP Isaacs-Kidd Midwater Trawl 1990-2009, SCDNR/NOAA MARMAP Program, SCDNR MARMAP Aggregate data surveys, The Marine Resources Monitoring, Assessment, and Prediction (MARMAP) Program, Marine Resources Research Institute, South Carolina Department of Natural Resources, P. O. Box 12559, Charleston SC 29422-2559, U.S.A.Retrieved from http://www.usgs.gov/obis-usa/	Restricted	OBIS (2019) [MARMAP Isaacs-Kidd Midwater Trawl 1990-2009] (Available: Ocean Biodiversity Information System. Intergovernmental Oceanographic Commission of UNESCO. https://obis.org. Accessed: 2019-08-29)

10b213e6-a9c4-459e-a40c-ef9edc461b97	Marine data from the Bernice P. Bishop Museum	Pyle R (2016). Bernice P. Bishop Museum. Version 8.1. Bernice Pauahi Bishop Museum. Occurrence dataset https://doi.org/10.15468/s6ctus accessed via GBIF.org on 2018-11-16.	To the extent possible under law, the publisher has waived all rights to these data and has dedicated them to the Public Domain (CC0 1.0)	OBIS (2019) [Marine data from the Bernice P. Bishop Museum] (Available: Ocean Biodiversity Information System. Intergovernmental Oceanographic Commission of UNESCO. https://obis.org. Accessed: 2019-08-29)
6a5bc28f-4dfe-4cbf-8a55-7e3a843997ab	SPC NECTALIS Zooplankton/Micronekton specimens, New Caledonia 2014	Allain, V., Menkes, C., 2014. Nectalis 3 cruise, RV Alis. https://doi.org/10.17600/14004900	This work is licensed under a Creative Commons Attribution Non Commercial (CC-BY-NC) 4.0 License	OBIS (2019) [SPC NECTALIS Zooplankton/Micronekton specimens, New Caledonia 2014] (Available: Ocean Biodiversity Information System. Intergovernmental Oceanographic Commission of UNESCO. https://obis.org. Accessed: 2019-08-29)
705770e5-3474-4e69-be8b-3107a0c5610a	The fishes collection (IC) of the Muséum national d'Histoire naturelle (MNHN - Paris)	Gicim data base, Pruvost P. Causse R., 2009 http://doi.org/10.15468/tm7whu	This work is licensed under a Creative Commons Attribution (CC-BY) 4.0 License	OBIS (2019) [The fishes collection (IC) of the Muséum national d'Histoire naturelle (MNHN - Paris)] (Available: Ocean Biodiversity Information System. Intergovernmental Oceanographic Commission of UNESCO. https://obis.org. Accessed: 2019-08-29)
cc8f28ce-e48d-4945-abfe-9d150a22dcd6	Hamburg pelagic fish database	Post, A. 1987. Pelagic transects of FRVs "Walther Herwig" and "Anton Dohrn" in the Atlantic Ocean 1966 to 1986. Mitt. Inst. f. Seefischerei d. BfaFi Hamburg, 42: 1-68.	This work is licensed under a Creative Commons Attribution (CC-BY) 4.0 License	OBIS (2019) [Hamburg pelagic fish database] (Available: Ocean Biodiversity Information System. Intergovernmental Oceanographic Commission of UNESCO. https://obis.org. Accessed: 2019-08-29)
ce1d93f3-8b0f-4ee7-9a4d-0393a6ec7fea	Atlantic Reference Centre Museum of Canadian Atlantic Organisms - Invertebrates and Fishes Data	Van Guelpen, L., 2016. Atlantic Reference Centre Museum of Canadian Atlantic Organisms - Invertebrates and Fishes Data. Version 4 In OBIS Canada Digital Collections. Bedford Institute of Oceanography, Dartmouth, NS, Canada. Published by OBIS, Digital http://www.iobis.org/. Accessed on –INSERT DATE	This work is licensed under a Creative Commons Attribution (CC-BY) 4.0 License	OBIS (2019) [Atlantic Reference Centre Museum of Canadian Atlantic Organisms - Invertebrates and Fishes Data] (Available: Ocean Biodiversity Information System. Intergovernmental Oceanographic Commission of UNESCO. https://obis.org. Accessed: 2019-08-29)

d6d6fe4c-425f-4ce7-bf28-7a6befaeb413	National Museum of Natural History Vertebrate Zoology Fishes Collections	National Museum of Natural History, Smithsonian Institution NMNH Fishes Collection Database. National Museum of Natural History, Smithsonian Institution, 10th and Constitution Ave. N.W., Washington, DC 20560-0193, 2007.	This work is licensed under a Creative Commons Attribution (CC-BY) 4.0 License	OBIS (2019) [National Museum of Natural History Vertebrate Zoology Fishes Collections] (Available: Ocean Biodiversity Information System. Intergovernmental Oceanographic Commission of UNESCO. https://obis.org. Accessed: 2019-08-29)

Diaphus mascarensis

GBIF

GBIF Occurrence Download https://doi.org/10.15468/dd.d7z8rh Accessed from R via rgbif (https://github.com/ropensci/rgbif) on 2019-09-11

OBIS

OBIS (2019) Distribution records of Diaphus mascarensis [Dataset] (Available: Ocean Biodiversity Information System. Intergovernmental Oceanographic Commission of UNESCO. www.obis.org. Accessed: 2019-08-29)

Dataset details:

Dataset_ID	Name	Citation	License	OBIS_citation
8a1ae661-e911-4967-bc06-1168fc5f2d89	iziko South African Museum - Fish Collection	iziko South African Museum - Fish Collection	Restricted	OBIS (2019) [iziko South African Museum - Fish Collection] (Available: Ocean Biodiversity Information System. Intergovernmental Oceanographic Commission of UNESCO. https://obis.org. Accessed: 2019-08-29)

Diaphus bertelseni

GBIF

GBIF Occurrence Download https://doi.org/10.15468/dd.r5vp4m Accessed from R via rgbif (https://github.com/ropensci/rgbif) on 2019-09-11

OBIS

OBIS (2019) Distribution records of Diaphus bertelseni [Dataset] (Available: Ocean Biodiversity Information System. Intergovernmental Oceanographic Commission of UNESCO. www.obis.org. Accessed: 2019-08-29)

Dataset details:

Dataset_ID	Name	Citation	License	OBIS_citation
6c19184e-c305-4273-8890-6d342d86f865	Institution REVIZEE - Collection Pelagic Fishes	No available dataset citation	Unspecified intellectual rights	OBIS (2019) [Institution REVIZEE - Collection Pelagic Fishes] (Available: Ocean Biodiversity Information System. Intergovernmental Oceanographic Commission of UNESCO. https://obis.org. Accessed: 2019-08-29)
8629ec33-be4b-4384-933f-a511fbc29967	MAR-ECO 2004	Wenneck, T. de Lange, Falkenhaug, T. and O.A. Bergstad. 2008. Strategies, methods, and technologies adopted on the RV G.O. Sars MAR-ECO expedition to the mid-Atlantic Ridge in 2004. Deep-sea Research II. 55: 6-28.	This work is licensed under a Creative Commons Attribution (CC-BY) 4.0 License	OBIS (2019) [MAR-ECO 2004] (Available: Ocean Biodiversity Information System. Intergovernmental Oceanographic Commission of UNESCO. https://obis.org. Accessed: 2019-08-29)
cc8f28ce-e48d-4945-abfe-9d150a22dcd6	Hamburg pelagic fish database	Post, A. 1987. Pelagic transects of FRVs "Walther Herwig" and "Anton Dohrn" in the Atlantic Ocean 1966 to 1986. Mitt. Inst. f. Seefischerei d. BfaFi Hamburg, 42: 1-68.	This work is licensed under a Creative Commons Attribution (CC-BY) 4.0 License	OBIS (2019) [Hamburg pelagic fish database] (Available: Ocean Biodiversity Information System. Intergovernmental Oceanographic Commission of UNESCO. https://obis.org. Accessed: 2019-08-29)
ce1d93f3-8b0f-4ee7-9a4d-0393a6ec7fea	Atlantic Reference Centre Museum of Canadian Atlantic Organisms - Invertebrates and Fishes Data	Van Guelpen, L., 2016. Atlantic Reference Centre Museum of Canadian Atlantic Organisms - Invertebrates and Fishes Data. Version 4 In OBIS Canada Digital Collections. Bedford Institute of Oceanography, Dartmouth, NS, Canada. Published by OBIS, Digital http://www.iobis.org/. Accessed on –INSERT DATE	This work is licensed under a Creative Commons Attribution (CC-BY) 4.0 License	OBIS (2019) [Atlantic Reference Centre Museum of Canadian Atlantic Organisms - Invertebrates and Fishes Data] (Available: Ocean Biodiversity Information System. Intergovernmental Oceanographic Commission of UNESCO. https://obis.org. Accessed: 2019-08-29)

d6d6fe4c-425f-4ce7-bf28-7a6befaeb413	National Museum of Natural History Vertebrate Zoology Fishes Collections	National Museum of Natural History, Smithsonian Institution NMNH Fishes Collection Database. National Museum of Natural History, Smithsonian Institution, 10th and Constitution Ave. N.W., Washington, DC 20560-0193, 2007.	This work is licensed under a Creative Commons Attribution (CC-BY) 4.0 License	OBIS (2019) [National Museum of Natural History Vertebrate Zoology Fishes Collections] (Available: Ocean Biodiversity Information System. Intergovernmental Oceanographic Commission of UNESCO. https://obis.org. Accessed: 2019-08-29)

Loweina rara

GBIF

GBIF Occurrence Download https://doi.org/10.15468/dd.r99b46 Accessed from R via rgbif (https://github.com/ropensci/rgbif) on 2019-09-11

OBIS

OBIS (2019) Distribution records of Loweina rara [Dataset] (Available: Ocean Biodiversity Information System. Intergovernmental Oceanographic Commission of UNESCO. www.obis.org. Accessed: 2019-08-29)

Dataset details:

Dataset_ID	Name	Citation	License	OBIS_citation
10b213e6-a9c4-459e-a40c-ef9edc461b97	Marine data from the Bernice P. Bishop Museum	Pyle R (2016). Bernice P. Bishop Museum. Version 8.1. Bernice Pauahi Bishop Museum. Occurrence dataset https://doi.org/10.15468/s6ctus accessed via GBIF.org on 2018-11-16.	To the extent possible under law, the publisher has waived all rights to these data and has dedicated them to the Public Domain (CC0 1.0)	OBIS (2019) [Marine data from the Bernice P. Bishop Museum] (Available: Ocean Biodiversity Information System. Intergovernmental Oceanographic Commission of UNESCO. https://obis.org. Accessed: 2019-08-29)
1f59030f-f116-4c34-915e-1882d819cda3	Institution Southampton Oceanography Ceter - Collection discovery	No available dataset citation	Unspecified intellectual rights	OBIS (2019) [Institution Southampton Oceanography Ceter - Collection discovery] (Available: Ocean Biodiversity Information System. Intergovernmental Oceanographic Commission of UNESCO. https://obis.org. Accessed: 2019-08-29)

2870c548-343e-4575-ac67-a4da35182c52	Institution Shirshov Institute - Collection SKAO	No available dataset citation	Unspecified intellectual rights	OBIS (2019) [Institution Shirshov Institute - Collection SKAO] (Available: Ocean Biodiversity Information System. Intergovernmental Oceanographic Commission of UNESCO. https://obis.org. Accessed: 2019-08-29)
705770e5-3474-4e69-be8b-3107a0c5610a	The fishes collection (IC) of the Muséum national d'Histoire naturelle (MNHN - Paris)	Gicim data base, Pruvost P. Causse R., 2009 http://doi.org/10.15468/tm7whu	This work is licensed under a Creative Commons Attribution (CC-BY) 4.0 License	OBIS (2019) [The fishes collection (IC) of the Muséum national d'Histoire naturelle (MNHN - Paris)] (Available: Ocean Biodiversity Information System. Intergovernmental Oceanographic Commission of UNESCO. https://obis.org. Accessed: 2019-08-29)
87a421bf-4646-49e3-89b7-409b93f2ac7c	Institution UWFC - Collection ADULT COLLECTION	No available dataset citation	Unspecified intellectual rights	OBIS (2019) [Institution UWFC - Collection ADULT COLLECTION] (Available: Ocean Biodiversity Information System. Intergovernmental Oceanographic Commission of UNESCO. https://obis.org. Accessed: 2019-08-29)
87a421bf-4646-49e3-89b7-409b93f2ac7c	Institution UWFC - Collection LARVAL COLLECTION	No available dataset citation	Unspecified intellectual rights	OBIS (2019) [Institution UWFC - Collection LARVAL COLLECTION] (Available: Ocean Biodiversity Information System. Intergovernmental Oceanographic Commission of UNESCO. https://obis.org. Accessed: 2019-08-29)
cc8f28ce-e48d-4945-abfe-9d150a22dcd6	Hamburg pelagic fish database	Post, A. 1987. Pelagic transects of FRVs "Walther Herwig" and "Anton Dohrn" in the Atlantic Ocean 1966 to 1986. Mitt. Inst. f. Seefischerei d. BfaFi Hamburg, 42: 1-68.	This work is licensed under a Creative Commons Attribution (CC-BY) 4.0 License	OBIS (2019) [Hamburg pelagic fish database] (Available: Ocean Biodiversity Information System. Intergovernmental Oceanographic Commission of UNESCO. https://obis.org. Accessed: 2019-08-29)
ce1d93f3-8b0f-4ee7-9a4d-0393a6ec7fea	Atlantic Reference Centre Museum of Canadian Atlantic Organisms - Invertebrates and Fishes Data	Van Guelpen, L., 2016. Atlantic Reference Centre Museum of Canadian Atlantic Organisms - Invertebrates and Fishes Data. Version 4 In OBIS Canada Digital Collections. Bedford Institute of Oceanography, Dartmouth, NS, Canada. Published by OBIS, Digital http://www.iobis.org/. Accessed on –INSERT DATE	This work is licensed under a Creative Commons Attribution (CC-BY) 4.0 License	OBIS (2019) [Atlantic Reference Centre Museum of Canadian Atlantic Organisms - Invertebrates and Fishes Data] (Available: Ocean Biodiversity Information System. Intergovernmental Oceanographic Commission of UNESCO. https://obis.org. Accessed: 2019-08-29)

d6d6fe4c-425f-4ce7-bf28-7a6befaeb413	National Museum of Natural History Vertebrate Zoology Fishes Collections	National Museum of Natural History, Smithsonian Institution NMNH Fishes Collection Database. National Museum of Natural History, Smithsonian Institution, 10th and Constitution Ave. N.W., Washington, DC 20560-0193, 2007.	This work is licensed under a Creative Commons Attribution (CC-BY) 4.0 License	OBIS (2019) [National Museum of Natural History Vertebrate Zoology Fishes Collections] (Available: Ocean Biodiversity Information System. Intergovernmental Oceanographic Commission of UNESCO. https://obis.org. Accessed: 2019-08-29)
f1da0955-5ece-4f98-ab77-f10c20bdd3ca	Institution OGL - Collection OGR	No available dataset citation	Unspecified intellectual rights	OBIS (2019) [Institution OGL - Collection OGR] (Available: Ocean Biodiversity Information System. Intergovernmental Oceanographic Commission of UNESCO. https://obis.org. Accessed: 2019-08-29)

Lampadena notialis

GBIF

GBIF Occurrence Download https://doi.org/10.15468/dd.ntrqj9 Accessed from R via rgbif (https://github.com/ropensci/rgbif) on 2019-09-11

OBIS

OBIS (2019) Distribution records of Lampadena notialis [Dataset] (Available: Ocean Biodiversity Information System. Intergovernmental Oceanographic Commission of UNESCO. www.obis.org. Accessed: 2019-08-29)

Dataset details:

Dataset_ID	Name	Citation	License	OBIS_citation
50903a57-ee9f-4367-b2cd-0b36dcf4a6ad	Catch data from New Zealand research trawls since 2008	SWPRON (2017). Catch data from New Zealand research trawls. Southwestern Pacific OBIS, National Institute of Water and Atmospheric Research (NIWA), Wellington, New Zealand, 15157 records, Online http://nzobisipt.niwa.co.nz/resource.do?r=trawl released on April 19, 2017.	This work is licensed under a Creative Commons Attribution (CC-BY) 4.0 License	OBIS (2019) [Catch data from New Zealand research trawls since 2008] (Available: Ocean Biodiversity Information System. Intergovernmental Oceanographic Commission of UNESCO. https://obis.org. Accessed: 2019-08-29)

8a1ae661-e911-4967-bc06-1168fc5f2d89	iziko South African Museum - Fish Collection	iziko South African Museum - Fish Collection	Restricted	OBIS (2019) [iziko South African Museum - Fish Collection] (Available: Ocean Biodiversity Information System. Intergovernmental Oceanographic Commission of UNESCO. https://obis.org. Accessed: 2019-08-29)
a4f7ee48-0d0b-4c05-a972-27a43b30db58	Institution MCM - Collection DEM	No available dataset citation	Unspecified intellectual rights	OBIS (2019) [Institution MCM - Collection DEM] (Available: Ocean Biodiversity Information System. Intergovernmental Oceanographic Commission of UNESCO. https://obis.org. Accessed: 2019-08-29)
d6d6fe4c-425f-4ce7-bf28-7a6befaeb413	National Museum of Natural History Vertebrate Zoology Fishes Collections	National Museum of Natural History, Smithsonian Institution NMNH Fishes Collection Database. National Museum of Natural History, Smithsonian Institution, 10th and Constitution Ave. N.W., Washington, DC 20560-0193, 2007.	This work is licensed under a Creative Commons Attribution (CC-BY) 4.0 License	OBIS (2019) [National Museum of Natural History Vertebrate Zoology Fishes Collections] (Available: Ocean Biodiversity Information System. Intergovernmental Oceanographic Commission of UNESCO. https://obis.org. Accessed: 2019-08-29)

Diaphus diadematus

GBIF

GBIF Occurrence Download https://doi.org/10.15468/dd.j9wwt4 Accessed from R via rgbif (https://github.com/ropensci/rgbif) on 2019-09-11

OBIS

OBIS (2019) Distribution records of Diaphus diadematus [Dataset] (Available: Ocean Biodiversity Information System. Intergovernmental Oceanographic Commission of UNESCO. www.obis.org. Accessed: 2019-08-29)

Dataset details:

Dataset_ID	Name	Citation	License	OBIS_citation

270f3e70-ff9b-411d-b170-2bc914d83f26	Biological Reference Collections ICM CSIC	Olivas González F J (2016): Biological Reference Collections ICM CSIC. Institute of Marine Sciences (ICM-CSIC). https://dx.doi.org/10.15470/qlqqdx	This work is licensed under a Creative Commons Attribution Non Commercial (CC-BY-NC) 4.0 License	OBIS (2019) [Biological Reference Collections ICM CSIC] (Available: Ocean Biodiversity Information System. Intergovernmental Oceanographic Commission of UNESCO. https://obis.org. Accessed: 2019-08-29)
705770e5-3474-4e69-be8b-3107a0c5610a	The fishes collection (IC) of the Muséum national d'Histoire naturelle (MNHN - Paris)	Gicim data base, Pruvost P. Causse R., 2009 http://doi.org/10.15468/tm7whu	This work is licensed under a Creative Commons Attribution (CC-BY) 4.0 License	OBIS (2019) [The fishes collection (IC) of the Muséum national d'Histoire naturelle (MNHN - Paris)] (Available: Ocean Biodiversity Information System. Intergovernmental Oceanographic Commission of UNESCO. https://obis.org. Accessed: 2019-08-29)
8a1ae661-e911-4967-bc06-1168fc5f2d89	iziko South African Museum - Fish Collection	iziko South African Museum - Fish Collection	Restricted	OBIS (2019) [iziko South African Museum - Fish Collection] (Available: Ocean Biodiversity Information System. Intergovernmental Oceanographic Commission of UNESCO. https://obis.org. Accessed: 2019-08-29)
9ff216fc-777e-4f9b-9860-95ed7366870d	Institution SAIAB - Collection SAIAB	No available dataset citation	Unspecified intellectual rights	OBIS (2019) [Institution SAIAB - Collection SAIAB] (Available: Ocean Biodiversity Information System. Intergovernmental Oceanographic Commission of UNESCO. https://obis.org. Accessed: 2019-08-29)
d6d6fe4c-425f-4ce7-bf28-7a6befaeb413	National Museum of Natural History Vertebrate Zoology Fishes Collections	National Museum of Natural History, Smithsonian Institution NMNH Fishes Collection Database. National Museum of Natural History, Smithsonian Institution, 10th and Constitution Ave. N.W., Washington, DC 20560-0193, 2007.	This work is licensed under a Creative Commons Attribution (CC-BY) 4.0 License	OBIS (2019) [National Museum of Natural History Vertebrate Zoology Fishes Collections] (Available: Ocean Biodiversity Information System. Intergovernmental Oceanographic Commission of UNESCO. https://obis.org. Accessed: 2019-08-29)

Lampanyctus turneri

GBIF

GBIF Occurrence Download https://doi.org/10.15468/dd.jz6dxh Accessed from R via rgbif (https://github.com/ropensci/rgbif) on 2019-09-11

OBIS

OBIS (2019) Distribution records of Lampanyctus turneri [Dataset] (Available: Ocean Biodiversity Information System. Intergovernmental Oceanographic Commission of UNESCO. www.obis.org. Accessed: 2019-08-29)

Dataset details:

Dataset_ID	Name	Citation	License	OBIS_citation
705770e5-3474-4e69-be8b-3107a0c5610a	The fishes collection (IC) of the Muséum national d'Histoire naturelle (MNHN - Paris)	Gicim data base, Pruvost P. Causse R., 2009 http://doi.org/10.15468/tm7whu	This work is licensed under a Creative Commons Attribution (CC-BY) 4.0 License	OBIS (2019) [The fishes collection (IC) of the Muséum national d'Histoire naturelle (MNHN - Paris)] (Available: Ocean Biodiversity Information System. Intergovernmental Oceanographic Commission of UNESCO. https://obis.org. Accessed: 2019-08-29)
8a1ae661-e911-4967-bc06-1168fc5f2d89	iziko South African Museum - Fish Collection	iziko South African Museum - Fish Collection	Restricted	OBIS (2019) [iziko South African Museum - Fish Collection] (Available: Ocean Biodiversity Information System. Intergovernmental Oceanographic Commission of UNESCO. https://obis.org. Accessed: 2019-08-29)

Bolinichthys nikolayi

GBIF

GBIF Occurrence Download https://doi.org/10.15468/dd.vfbnhr Accessed from R via rgbif (https://github.com/ropensci/rgbif) on 2019-09-11

OBIS

OBIS (2019) Distribution records of Bolinichthys nikolayi [Dataset] (Available: Ocean Biodiversity Information System. Intergovernmental Oceanographic Commission of UNESCO. www.obis.org. Accessed: 2019-08-29)

Dataset details:

Dataset_ID	Name	Citation	License	OBIS_citation

705770e5-3474-4e69-be8b-3107a0c5610a	The fishes collection (IC) of the Muséum national d'Histoire naturelle (MNHN - Paris)	Gicim data base, Pruvost P. Causse R., 2009 http://doi.org/10.15468/tm7whu	This work is licensed under a Creative Commons Attribution (CC-BY) 4.0 License	OBIS (2019) [The fishes collection (IC) of the Muséum national d'Histoire naturelle (MNHN - Paris)] (Available: Ocean Biodiversity Information System. Intergovernmental Oceanographic Commission of UNESCO. https://obis.org. Accessed: 2019-08-29)

Diaphus coeruleus

GBIF

GBIF Occurrence Download https://doi.org/10.15468/dd.rkmebs Accessed from R via rgbif (https://github.com/ropensci/rgbif) on 2019-09-11

OBIS

OBIS (2019) Distribution records of Diaphus coeruleus [Dataset] (Available: Ocean Biodiversity Information System. Intergovernmental Oceanographic Commission of UNESCO. www.obis.org. Accessed: 2019-08-29)

Dataset details:

Dataset_ID	Name	Citation	License	OBIS_citation
d6d6fe4c-425f-4ce7-bf28-7a6befaeb413	National Museum of Natural History Vertebrate Zoology Fishes Collections	National Museum of Natural History, Smithsonian Institution NMNH Fishes Collection Database. National Museum of Natural History, Smithsonian Institution, 10th and Constitution Ave. N.W., Washington, DC 20560-0193, 2007.	This work is licensed under a Creative Commons Attribution (CC-BY) 4.0 License	OBIS (2019) [National Museum of Natural History Vertebrate Zoology Fishes Collections] (Available: Ocean Biodiversity Information System. Intergovernmental Oceanographic Commission of UNESCO. https://obis.org. Accessed: 2019-08-29)
ff8b7809-41bc-40ad-8160-0e33862817a0	Biodiversity Research Museum, Academia Sinica, Taiwan	TELDAP, Biodiversity Research Museum, Academia Sinica, Taiwan (accessed through GBIF data portal, http://data.gbif.org/datasets/resource/9093, yyyy-mm-dd)	Unrestricted	OBIS (2019) [Biodiversity Research Museum, Academia Sinica, Taiwan] (Available: Ocean Biodiversity Information System. Intergovernmental Oceanographic Commission of UNESCO. https://obis.org. Accessed: 2019-08-29)

Loweina interrupta

GBIF

GBIF Occurrence Download https://doi.org/10.15468/dd.m2juy5 Accessed from R via rgbif (https://github.com/ropensci/rgbif) on 2019-09-11

OBIS

OBIS (2019) Distribution records of Loweina interrupta [Dataset] (Available: Ocean Biodiversity Information System. Intergovernmental Oceanographic Commission of UNESCO. www.obis.org. Accessed: 2019-08-29)

Dataset details:

Dataset_ID	Name	Citation	License	OBIS_citation
8629ec33-be4b-4384-933f-a511fbc29967	MAR-ECO 2004	Wenneck, T. de Lange, Falkenhaug, T. and O.A. Bergstad. 2008. Strategies, methods, and technologies adopted on the RV G.O. Sars MAR-ECO expedition to the mid-Atlantic Ridge in 2004. Deep-sea Research II. 55: 6-28.	This work is licensed under a Creative Commons Attribution (CC-BY) 4.0 License	OBIS (2019) [MAR-ECO 2004] (Available: Ocean Biodiversity Information System. Intergovernmental Oceanographic Commission of UNESCO. https://obis.org. Accessed: 2019-08-29)
cc8f28ce-e48d-4945-abfe-9d150a22dcd6	Hamburg pelagic fish database	Post, A. 1987. Pelagic transects of FRVs "Walther Herwig" and "Anton Dohrn" in the Atlantic Ocean 1966 to 1986. Mitt. Inst. f. Seefischerei d. BfaFi Hamburg, 42: 1-68.	This work is licensed under a Creative Commons Attribution (CC-BY) 4.0 License	OBIS (2019) [Hamburg pelagic fish database] (Available: Ocean Biodiversity Information System. Intergovernmental Oceanographic Commission of UNESCO. https://obis.org. Accessed: 2019-08-29)

Lampanyctus phyllisae

GBIF

GBIF Occurrence Download https://doi.org/10.15468/dd.hw7693 Accessed from R via rgbif (https://github.com/ropensci/rgbif) on 2019-09-11

OBIS

OBIS (2019) Distribution records of Lampanyctus phyllisae [Dataset] (Available: Ocean Biodiversity Information System. Intergovernmental Oceanographic Commission of UNESCO. www.obis.org. Accessed: 2019-08-29)

Dataset details:

Dataset_ID	Name	Citation	License	OBIS_citation
d6d6fe4c-425f-4ce7-bf28-7a6befaeb413	National Museum of Natural History Vertebrate Zoology Fishes Collections	National Museum of Natural History, Smithsonian Institution NMNH Fishes Collection Database. National Museum of Natural History, Smithsonian Institution, 10th and Constitution Ave. N.W., Washington, DC 20560-0193, 2007.	This work is licensed under a Creative Commons Attribution (CC-BY) 4.0 License	OBIS (2019) [National Museum of Natural History Vertebrate Zoology Fishes Collections] (Available: Ocean Biodiversity Information System. Intergovernmental Oceanographic Commission of UNESCO. https://obis.org. Accessed: 2019-08-29)

Lampanyctus wisneri

GBIF

GBIF Occurrence Download https://doi.org/10.15468/dd.s53x7j Accessed from R via rgbif (https://github.com/ropensci/rgbif) on 2019-09-11

OBIS

OBIS (2019) Distribution records of Lampanyctus wisneri [Dataset] (Available: Ocean Biodiversity Information System. Intergovernmental Oceanographic Commission of UNESCO. www.obis.org. Accessed: 2019-08-29)

Dataset details:

Dataset_ID	Name	Citation	License	OBIS_citation
d6d6fe4c-425f-4ce7-bf28-7a6befaeb413	National Museum of Natural History Vertebrate Zoology Fishes Collections	National Museum of Natural History, Smithsonian Institution NMNH Fishes Collection Database. National Museum of Natural History, Smithsonian Institution, 10th and Constitution Ave. N.W., Washington, DC 20560-0193, 2007.	This work is licensed under a Creative Commons Attribution (CC-BY) 4.0 License	OBIS (2019) [National Museum of Natural History Vertebrate Zoology Fishes Collections] (Available: Ocean Biodiversity Information System. Intergovernmental Oceanographic Commission of UNESCO. https://obis.org. Accessed: 2019-08-29)

Diaphus kapalae

GBIF

GBIF Occurrence Download https://doi.org/10.15468/dd.d2p9mq Accessed from R via rgbif (https://github.com/ropensci/rgbif) on 2019-09-11

OBIS

OBIS (2019) Distribution records of Diaphus kapalae [Dataset] (Available: Ocean Biodiversity Information System. Intergovernmental Oceanographic Commission of UNESCO. www.obis.org. Accessed: 2019-08-29)

Dataset details:

Dataset_ID	Name	Citation	License	OBIS_citation
8843341c-ddd1-47a0-b4e6-5f37eec9b317	Institution AADC - Collection Historic_Fish	No available dataset citation	Unspecified intellectual rights	OBIS (2019) [Institution AADC - Collection Historic_Fish] (Available: Ocean Biodiversity Information System. Intergovernmental Oceanographic Commission of UNESCO. https://obis.org. Accessed: 2019-08-29)

Metelectrona herwigi

GBIF

GBIF Occurrence Download https://doi.org/10.15468/dd.peht5n Accessed from R via rgbif (https://github.com/ropensci/rgbif) on 2019-09-11

OBIS

OBIS (2019) Distribution records of Metelectrona herwigi [Dataset] (Available: Ocean Biodiversity Information System. Intergovernmental Oceanographic Commission of UNESCO. www.obis.org. Accessed: 2019-08-29)

Dataset details:

Dataset_ID	Name	Citation	License	OBIS_citation
8a1ae661-e911-4967-bc06-1168fc5f2d89	iziko South African Museum - Fish Collection	iziko South African Museum - Fish Collection	Restricted	OBIS (2019) [iziko South African Museum - Fish Collection] (Available: Ocean Biodiversity Information System. Intergovernmental Oceanographic Commission of UNESCO. https://obis.org. Accessed: 2019-08-29)

Hygophum bruuni

GBIF

GBIF Occurrence Download https://doi.org/10.15468/dd.7bez9y Accessed from R via rgbif (https://github.com/ropensci/rgbif) on 2019-09-11

OBIS

OBIS (2019) Distribution records of Hygophum bruuni [Dataset] (Available: Ocean Biodiversity Information System. Intergovernmental Oceanographic Commission of UNESCO. www.obis.org. Accessed: 2019-08-29)

Dataset details:

Dataset_ID	Name	Citation	License	OBIS_citation
d6d6fe4c-425f-4ce7-bf28-7a6befaeb413	National Museum of Natural History Vertebrate Zoology Fishes Collections	National Museum of Natural History, Smithsonian Institution NMNH Fishes Collection Database. National Museum of Natural History, Smithsonian Institution, 10th and Constitution Ave. N.W., Washington, DC 20560-0193, 2007.	This work is licensed under a Creative Commons Attribution (CC-BY) 4.0 License	OBIS (2019) [National Museum of Natural History Vertebrate Zoology Fishes Collections] (Available: Ocean Biodiversity Information System. Intergovernmental Oceanographic Commission of UNESCO. https://obis.org. Accessed: 2019-08-29)

Triphoturus mexicanus

GBIF

GBIF Occurrence Download https://doi.org/10.15468/dd.kmqk5r Accessed from R via rgbif (https://github.com/ropensci/rgbif) on 2019-09-11

OBIS

OBIS (2019) Distribution records of Triphoturus mexicanus [Dataset] (Available: Ocean Biodiversity Information System. Intergovernmental Oceanographic Commission of UNESCO. www.obis.org. Accessed: 2019-08-29)

Dataset details:

Dataset_ID	Name	Citation	License	OBIS_citation

10b213e6-a9c4-459e-a40c-ef9edc461b97	Marine data from the Bernice P. Bishop Museum	Pyle R (2016). Bernice P. Bishop Museum. Version 8.1. Bernice Pauahi Bishop Museum. Occurrence dataset https://doi.org/10.15468/s6ctus accessed via GBIF.org on 2018-11-16.	To the extent possible under law, the publisher has waived all rights to these data and has dedicated them to the Public Domain (CC0 1.0)	OBIS (2019) [Marine data from the Bernice P. Bishop Museum] (Available: Ocean Biodiversity Information System. Intergovernmental Oceanographic Commission of UNESCO. https://obis.org. Accessed: 2019-08-29)
8a1ae661-e911-4967-bc06-1168fc5f2d89	iziko South African Museum - Fish Collection	iziko South African Museum - Fish Collection	Restricted	OBIS (2019) [iziko South African Museum - Fish Collection] (Available: Ocean Biodiversity Information System. Intergovernmental Oceanographic Commission of UNESCO. https://obis.org. Accessed: 2019-08-29)

Protomyctophum chilense

GBIF

GBIF Occurrence Download https://doi.org/10.15468/dd.k9g869 Accessed from R via rgbif (https://github.com/ropensci/rgbif) on 2019-09-11

Lampanyctus iselinoides

GBIF

GBIF Occurrence Download https://doi.org/10.15468/dd.sqexzn Accessed from R via rgbif (https://github.com/ropensci/rgbif) on 2019-09-11

Metelectrona ahlstromi

GBIF

GBIF Occurrence Download https://doi.org/10.15468/dd.typsgp Accessed from R via rgbif (https://github.com/ropensci/rgbif) on 2019-09-11
