# Supplementary material for: Biogeographical Regions and Climate Change: Lanternfishes Shed Light on the Role of Climatic Barriers in the Southern Ocean
Source: Glob Chang Biol. 2025 Jun 16;31(6):e70256. doi: 10.1111/gcb.70256 (PMC12168107; doi:10.1111/gcb.70256)
Supplement: Supplementary file 2 — Appendix S4. [file GCB-31-e70256-s001.pdf]

Appendix S4: Myctophid occurrence corrections

Cam Ly Rintz, Boris Leroy, P.A. Hulley, Philippe Koubbi

10/06/2022

Contents

Introduction **6**

Automatic error and outlier detection **6**

Expert-based validation of occurrence records **6**

Benthosema fibulatum 6

Benthosema suborbitale 7

Bolinichthys indicus 8

Bolinichthys longipes 10

Bolinichthys nikolayi 11

Bolinichthys photothorax 12

Bolinichthys supralateralis 13

Centrobranchus nigroocellatus 14

Ceratoscopelus townsendi 15

Ceratoscopelus warmingii 16

Diaphus anderseni 17

Diaphus bertelseni 18

Diaphus brachycephalus 19

Diaphus coeruleus 20

Diaphus danae 21
Diaphus diadematus 22
Diaphus dumerilii 24
Diaphus effulgens 25
Diaphus fragilis 26
Diaphus garmani 27
Diaphus holti 28
Diaphus hudsoni 29
Diaphus kapalae 31
Diaphus lucidus 32
Diaphus luetkeni 33
Diaphus mascarensis 34
Diaphus meadi 34
Diaphus metopoclampus 35
Diaphus mollis 36
Diaphus ostenfeldi 37
Diaphus parri 39
Diaphus perspicillatus 39
Diaphus splendidus 40
Diaphus taaningi 41
Diaphus termophilus 43
Diaphus theta 44
Diaphus watasei 45
Diogenichthys atlanticus 46
Diogenichthys panurgus 47
Electrona antarctica 48
Electrona carlsbergi 50
Electrona paucirastra 52

Electrona risso 53
Electrona subaspera 54
Gonichthys barnesi 56
Gonichthys cocco 57
Gymnoscopelus bolini 59
Gymnoscopelus braueri 60
Gymnoscopelus fraseri 62
Gymnoscopelus hintonoides 64
Gymnoscopelus microlampas 65
Gymnoscopelus nicholsi 66
Gymnoscopelus opisthopterus 69
Gymnoscopelus piabilis 70
Hintonia candens 72
Hygophum bruuni 74
Hygophum hanseni 75
Hygophum hygomii 77
Hygophum macrochir 78
Hygophum proximum 79
Hygophum reinhardtii 80
Krefftichthys anderssoni 81
Lampadena chavesi 84
Lampadena dea 85
Lampadena luminosa 85
Lampadena notialis 86
Lampadena speculigera 87
Lampanyctodes hectoris 88
Lampanyctus achirus 90
Lampanyctus alatus 93

Lampanyctus ater 94
Lampanyctus australis 97
Lampanyctus festivus 102
Lampanyctus intricarius 103
Lampanyctus iselinoides 103
Lampanyctus lepidolychnus 105
Lampanyctus macdonaldi 106
Lampanyctus nobilis 109
Lampanyctus phyllisae 110
Lampanyctus pusillus 110
Lampanyctus tenuiformis 111
Lampanyctus turneri 112
Lampanyctus wisneri 113
Lampichthys procerus 114
Lepidophanes gaussi 116
Lepidophanes guentheri 117
Lobianchia dofleini 118
Lobianchia gemellarii 120
Loweina interrupta 121
Loweina rara 121
Metelectrona ahlstromi 122
Metelectrona herwigi 123
Metelectrona ventralis 124
Myctophum affine 126
Myctophum asperum 127
Myctophum nitidulum 128
Myctophum obtusirostre 129
Myctophum phengodes 130

Myctophum punctatum . . . 132
Myctophum selenops . . . 133
Myctophum spinosum . . . 134
Notolychnus valdiviae . . . 135
Notoscopelus caudispinosus . . . 136
Notoscopelus resplendens . . . 137
Protomyctophum andriashevi . . . 138
Protomyctophum bolini . . . 140
Protomyctophum chilense . . . 143
Protomyctophum choriodon . . . 144
Protomyctophum gemmatum . . . 144
Protomyctophum luciferum . . . 145
Protomyctophum normani . . . 146
Protomyctophum parallelum . . . 148
Protomyctophum subparallelum . . . 150
Protomyctophum tenisoni . . . 152
Scopelopsis multipunctatus . . . 154
Symbolophorus barnardi . . . 156
Symbolophorus boops . . . 157
Symbolophorus evermanni . . . 158
Taaningichthys bathyphilus . . . 160
Taaningichthys minimus . . . 160
Triphoturus mexicanus . . . 161
Triphoturus nigrescens . . . 163

References **163**

Introduction

We compiled a global occurrence database for all myctophid species from multiple sources: the Ocean Biodiversity Information System (see Appendix S1); the Global Biodiversity Information Facility (see Appendix S1); the Biogeographic Atlas of the Southern Ocean (Duhamel et al., 2014). Additionally, we incorporated supplementary data from recent oceanographic surveys (Koubbi et al., 2023), available on https://doi.org/10.5281/zenodo.15306956. We also included records from our own datasets, available on https://doi.org/10.5281/zenodo.15295380.

We applied a multi-step verification procedure combining automatic error detections and expert validation. All these verification steps and associated actions are detailed below.

Automatic error and outlier detection

We used the function clean_coordinates from the R package CoordinateCleaner version 2.0-20 (Zizka et al., 2019). Specifically, we tested for equal coordinates, coordinates over land using the Natural Earth data ocean shapefile version 4.1.0 (www.naturalearthdata.com, accessed August 2019), zero coordinates and outliers.

Expert-based validation of occurrence records

We carefully mapped and screened all occurrence records, relying on expert-based knowledge (P.A. Hulley, comm. pers.) on specific taxonomic issues (taxonomic confusion, difficult identification) to remove occurrence records. All our decisions are provided with details and rationale below, for each species.

Benthosema fibulatum

We found no outliers for this species.

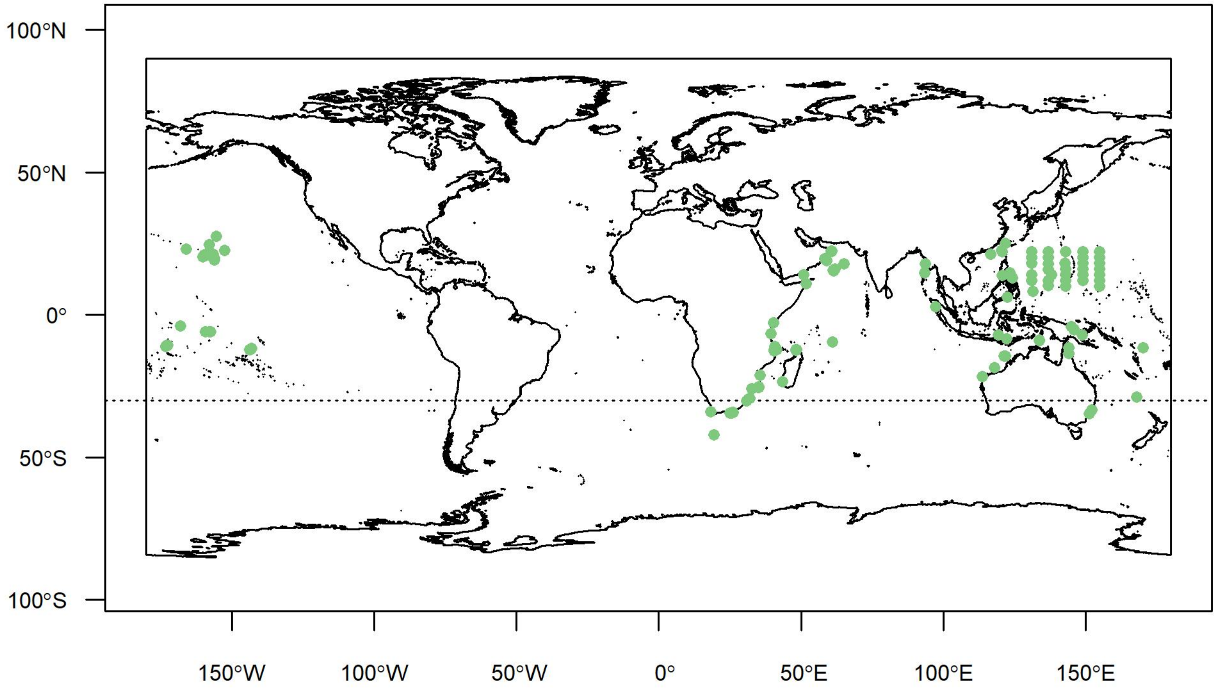

Benthosema suborbitale

We found no outliers for this species.

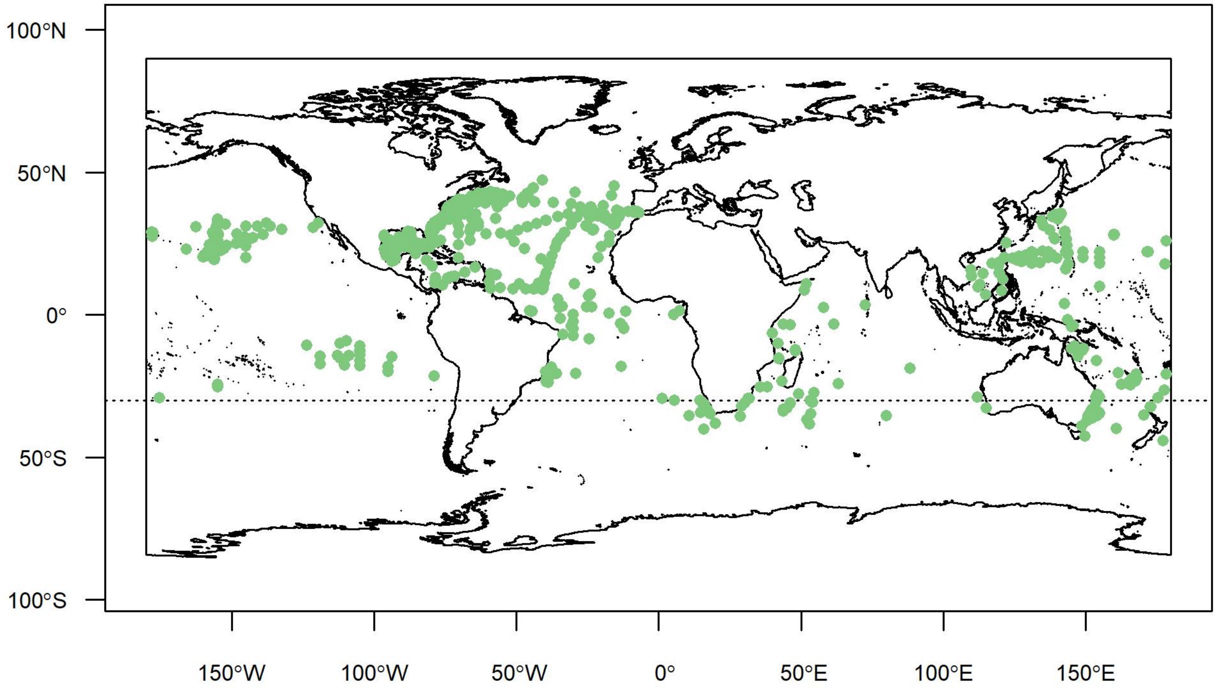

Bolinichthys indicus

We found three outliers in the Indian Ocean (red points on the map), which we removed after verification. (Hulley & Duhamel, 2009)

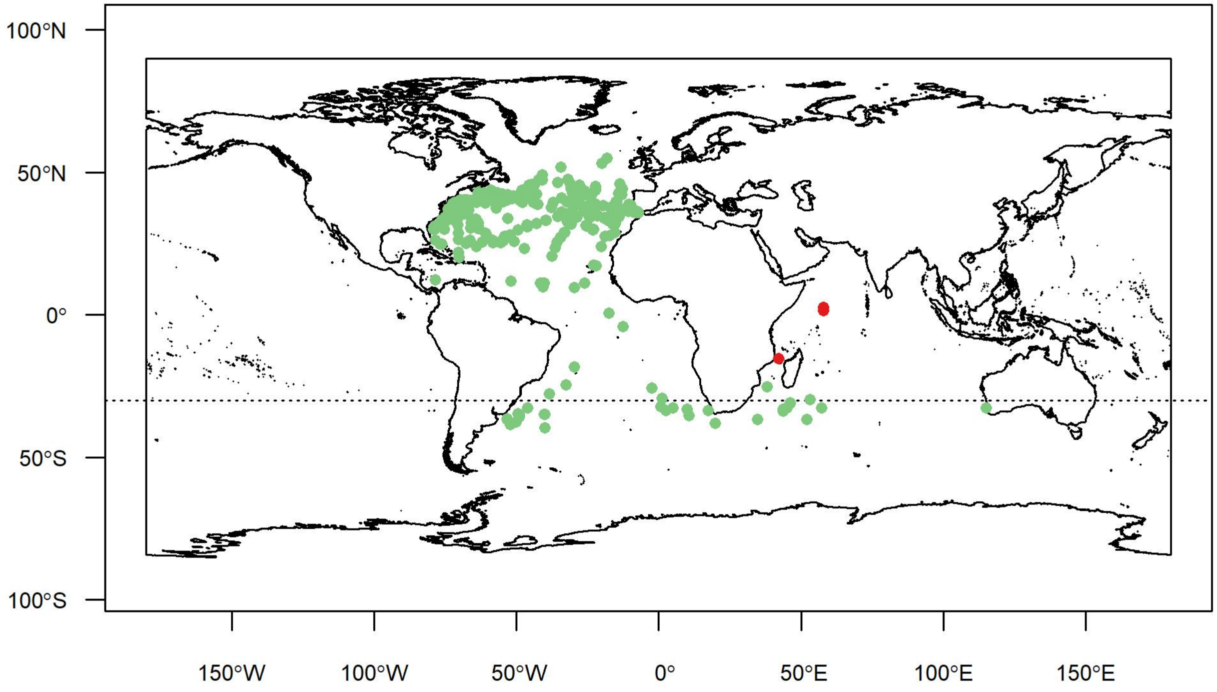

Information about the outliers :

	source	decimalLongitude	decimalLatitude	year	references
942161	GBIF	58.0217	2.4367	1964	
140422	GBIF	42.2500	-15.4500	1988	
180675	GBIF	57.9917	1.4867	1964	

Bolinichthys longipes

We found several outliers in the South Indian Ocean (red points on the map), which we removed. (Hulley & Duhamel, 2009)

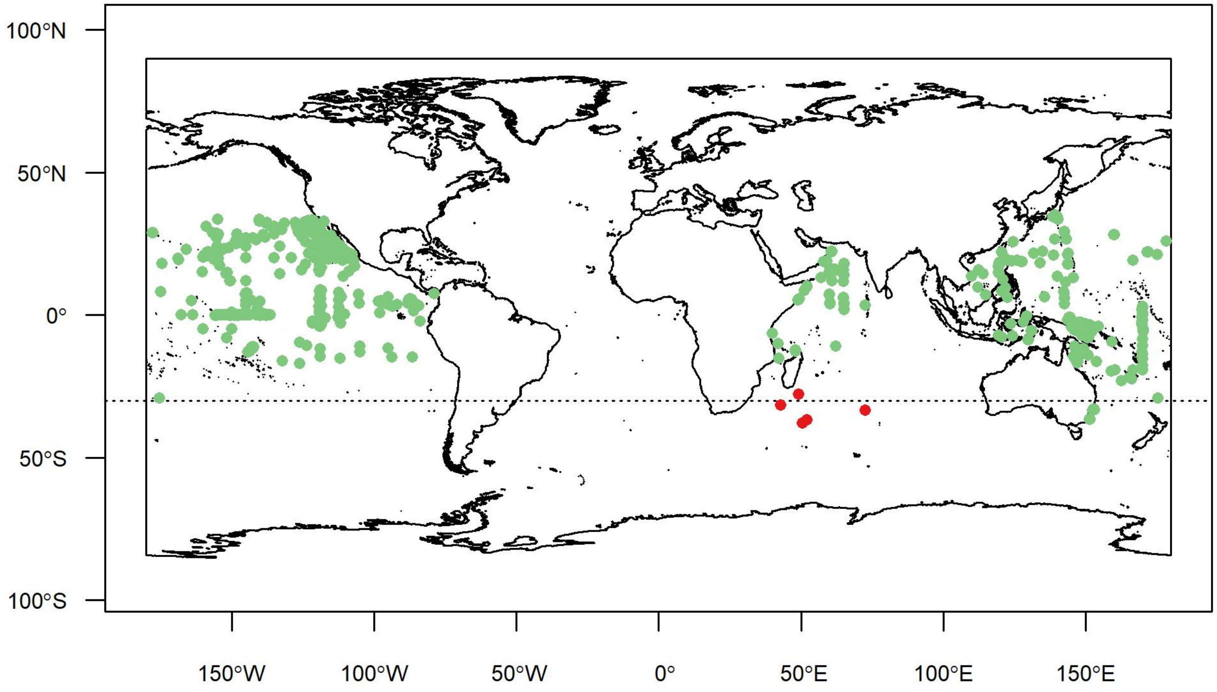

Information about the outliers :

	source	decimalLongitude	decimalLatitude	year	references
207901	GBIF	72.5450	-33.4617	1960	http://portal.vertnet.org/o/sio/marine-vertebrates?id=df8818f1-fb90-4383-b32b-eada36a1edb6
889301	GBIF	52.0555	-36.8420	2009	
891931	GBIF	50.4089	-37.9573	2009	

	source	decimalLongitude	decimalLatitude	year	references
895721	GBIF	52.0539	-36.8556	2009	
895971	GBIF	42.8798	-31.5963	2009	
896231	GBIF	42.8325	-31.6410	2009	
140413	GBIF	49.1667	-27.9500	1988	

Bolinichthys nikolayi

This species is restricted to the Pacific Ocean. We found two outliers in the Indian Ocean (red points on the map), which we removed. (Hulley & Duhamel, 2009)

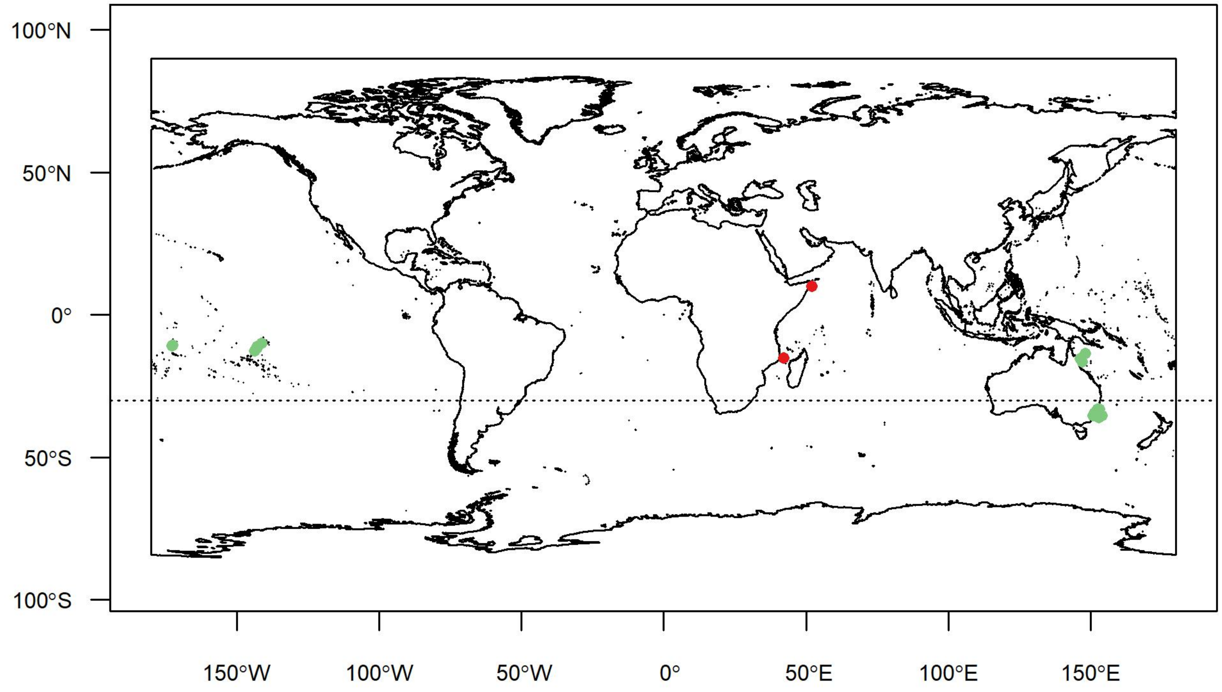

Information about the outliers :

	source	decimalLongitude	decimalLatitude	year	references
134758	GBIF	51.9983	10.0017	1995	http://mczbase.mcz.harvard.edu/guid/MCZ:Ich:151737
167364	GBIF	42.2000	-15.3500	1988	

Bolinichthys photothorax

We found no outliers for this species.

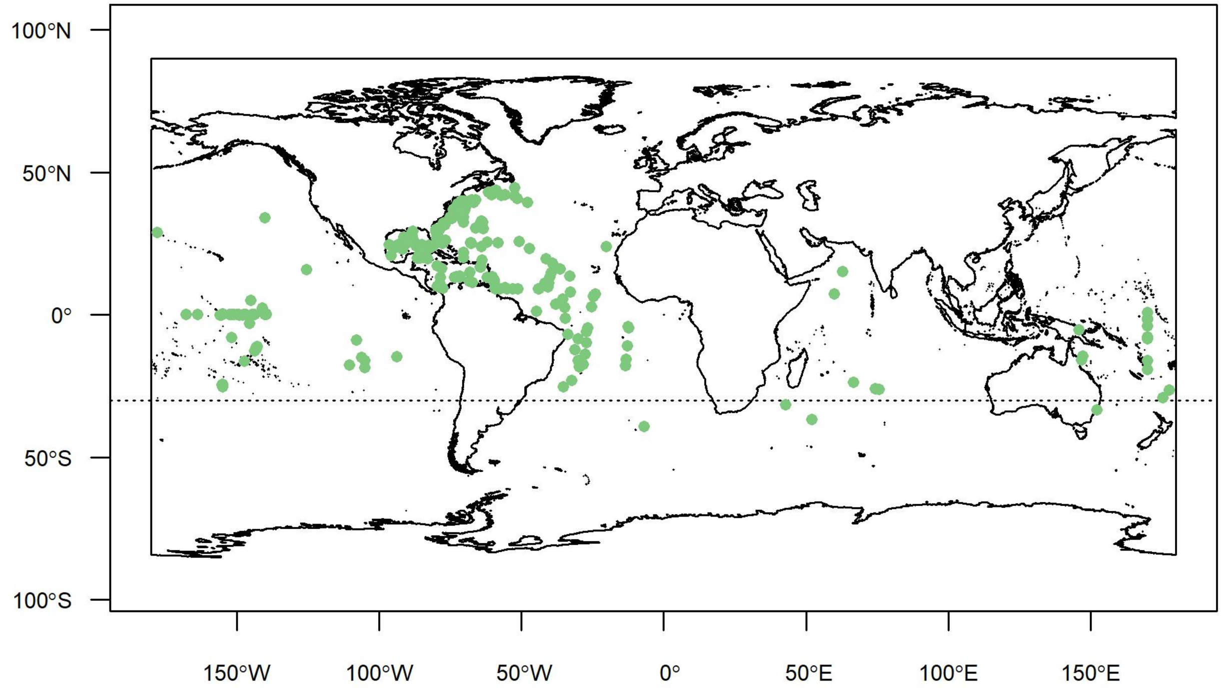

Bolinichthys supralateralis

We found one outlier in the Arabic Sea (red points on the map), which we removed after verification. (Hulley & Duhamel, 2009 and Meera, 2018)

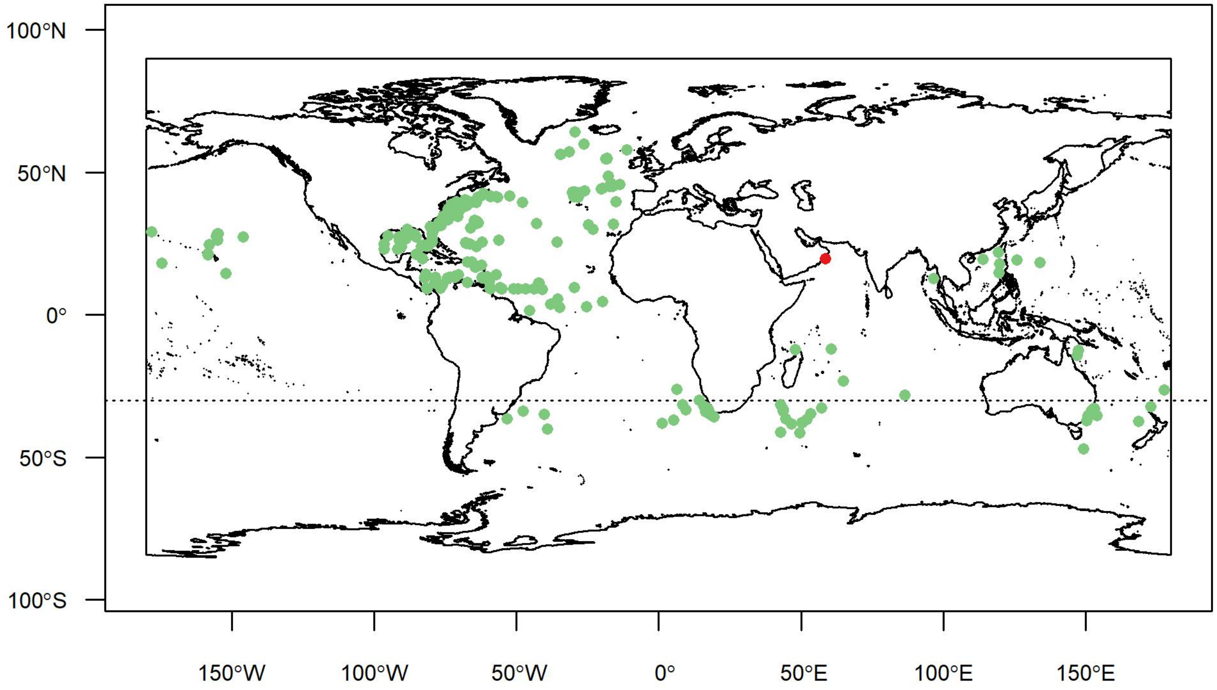

Information about the outliers :

	source	decimalLongitude	decimalLatitude	year	references
180665	GBIF	58.51	19.4925	1994	

Centrobranchus nigroocellatus

We found no outliers for this species.

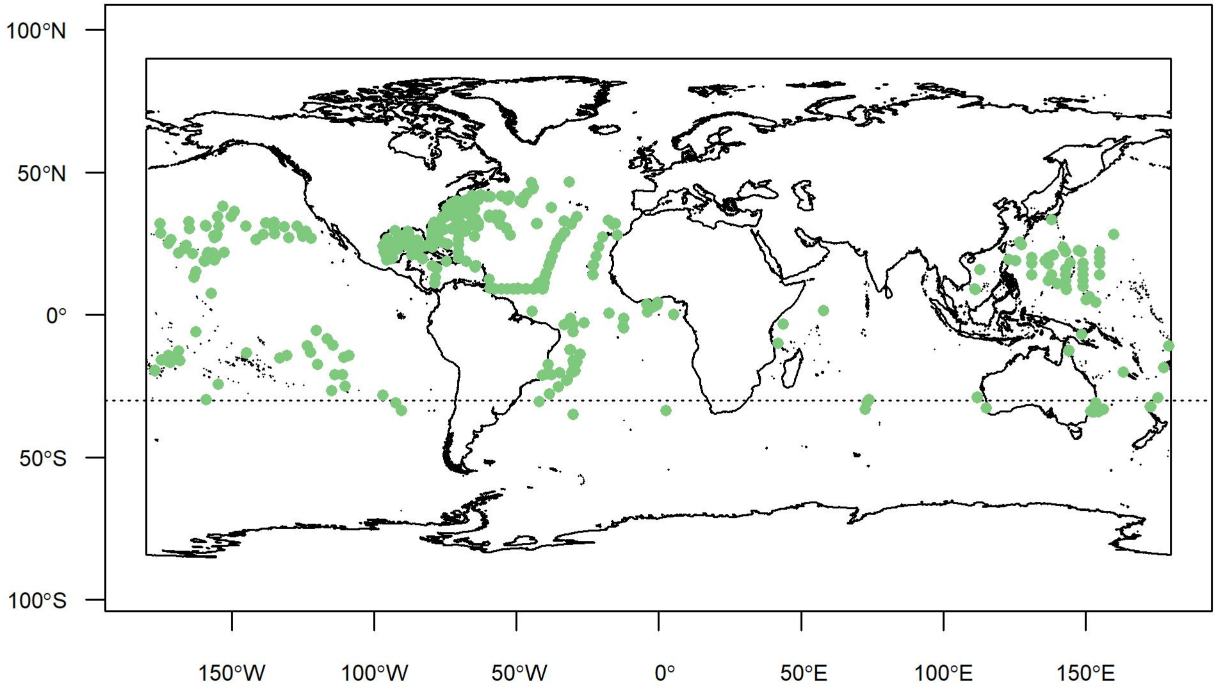

Ceratoscopelus townsendi

This species is distributed only in the North-Eastern Pacific, and does not occur below -30° latitude so we removed it from our dataset.

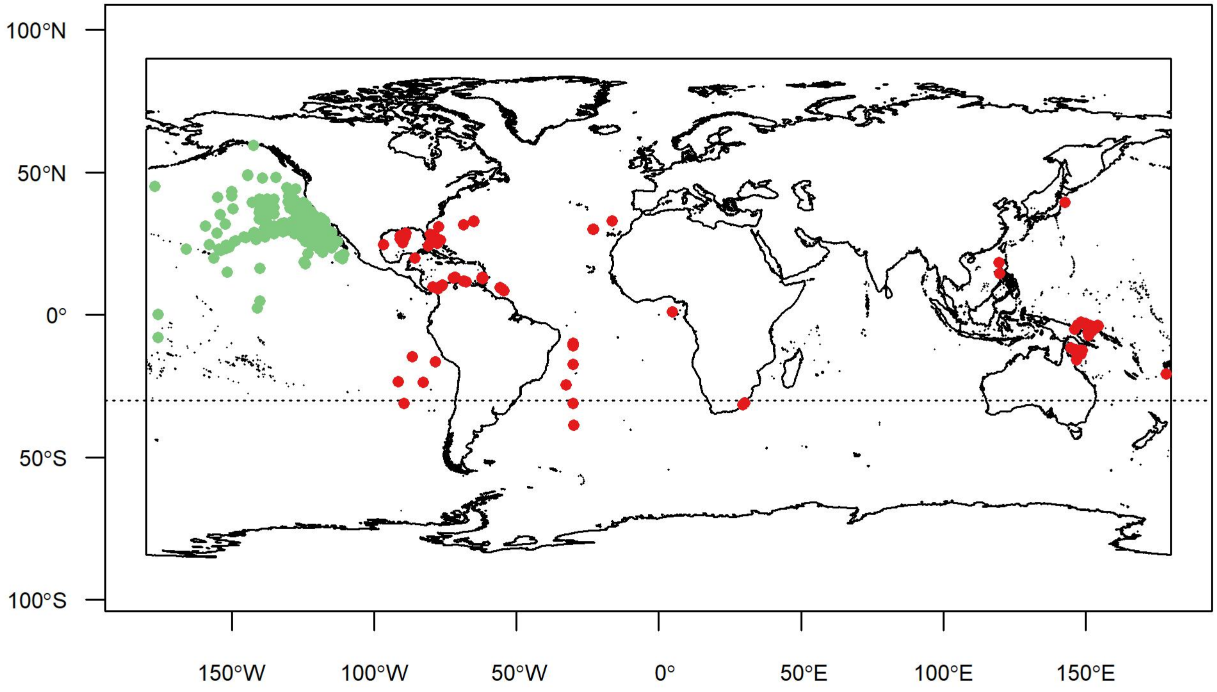

Ceratoscopelus warmingii

This species may occur in pair with other species, e.g. with *C. townsendi* off the Californian coast, and with *C. maderensis* off the Northwestern African coast. The distinction between these pairs of species is problematic and records should be checked for validity, which could not be done here because the records were extracted from OBIS and GBIF. The localisation of these records (above the prediction area, 30°S) implies that they are only used for the calibration of niche models. Deleting these records would reduce commission errors, but would also result in a loss of information about the suitable niche for this species, as it is known to occur in these areas. Keeping these records would increase commission errors, but avoid information loss about the suitable climate. We deemed more important to avoid information loss about suitable climate here, especially since our modelling procedure includes an environmental filtering step which will avoid overrepresentation of data (e.g. because of commission errors). Therefore, we decided to keep these records for

the calibration of ecological niche models.

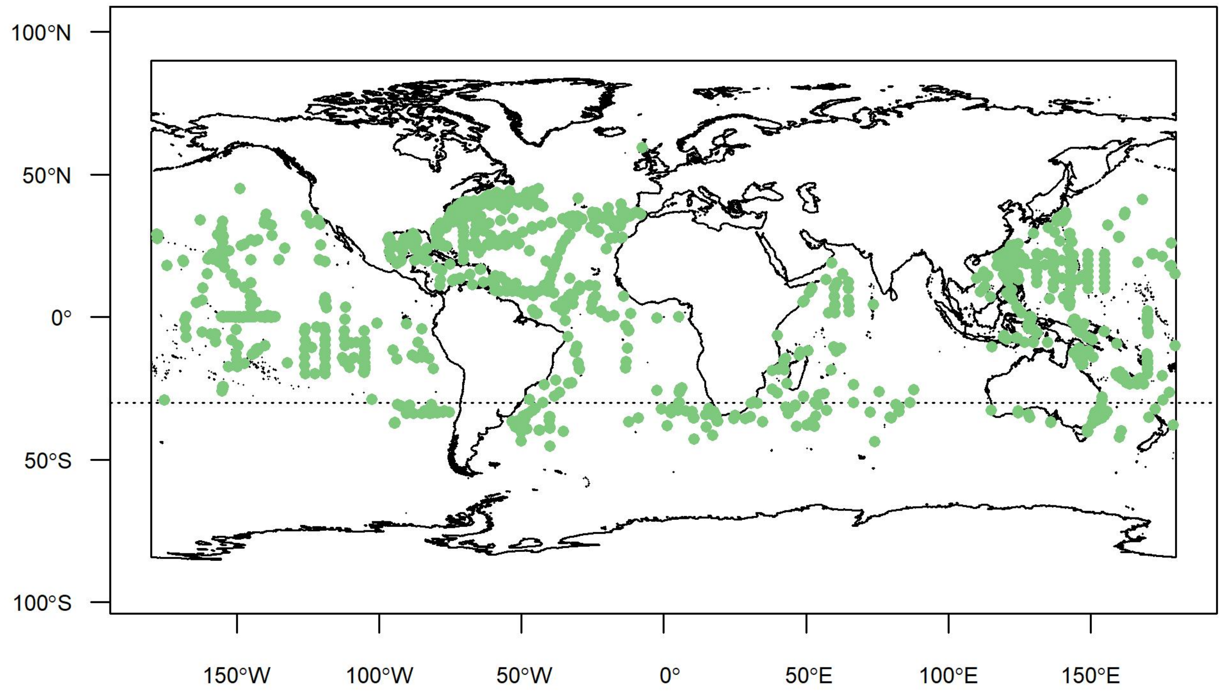

Diaphus anderseni

We found no outliers for this species.

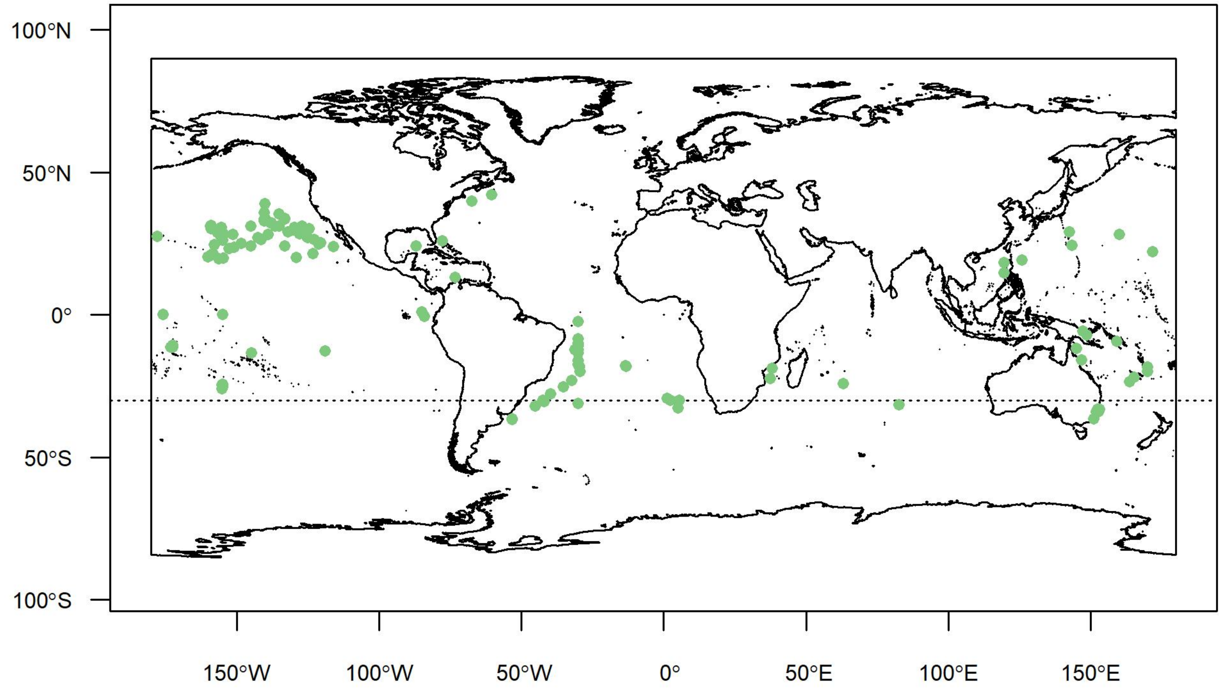

Diaphus bertelseni

We found no outliers for this species.

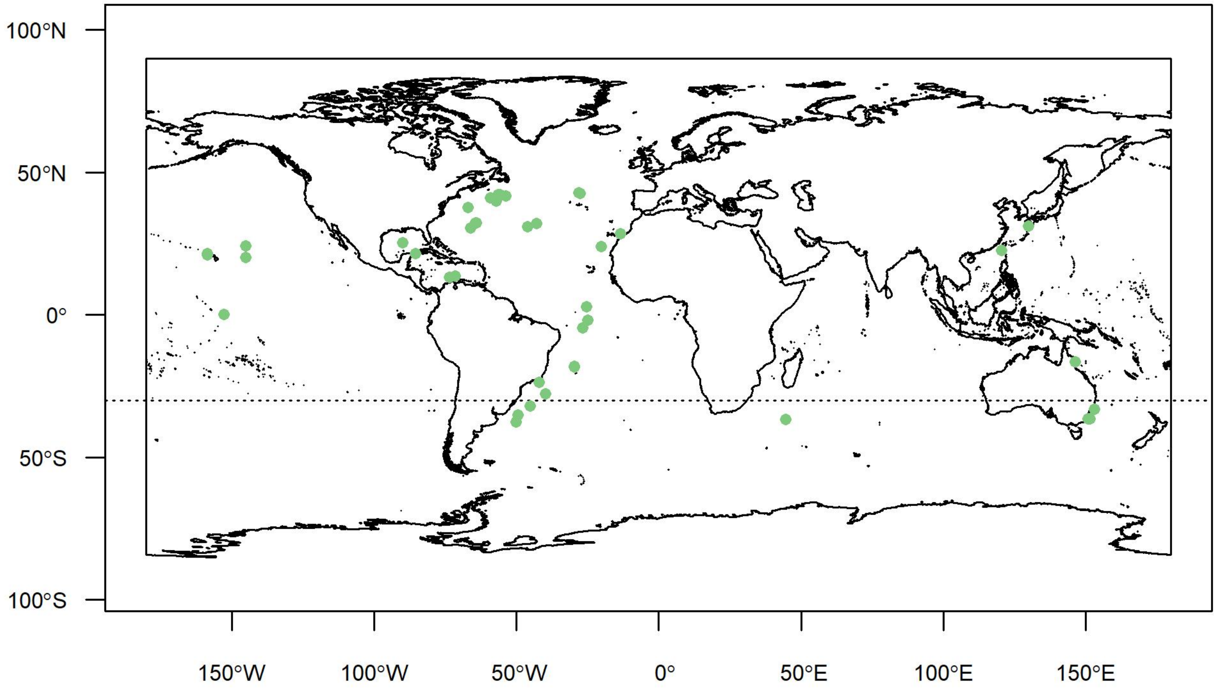

Diaphus brachycephalus

We found one outlier North of the Pacific Ocean (in red on the map), which we removed.

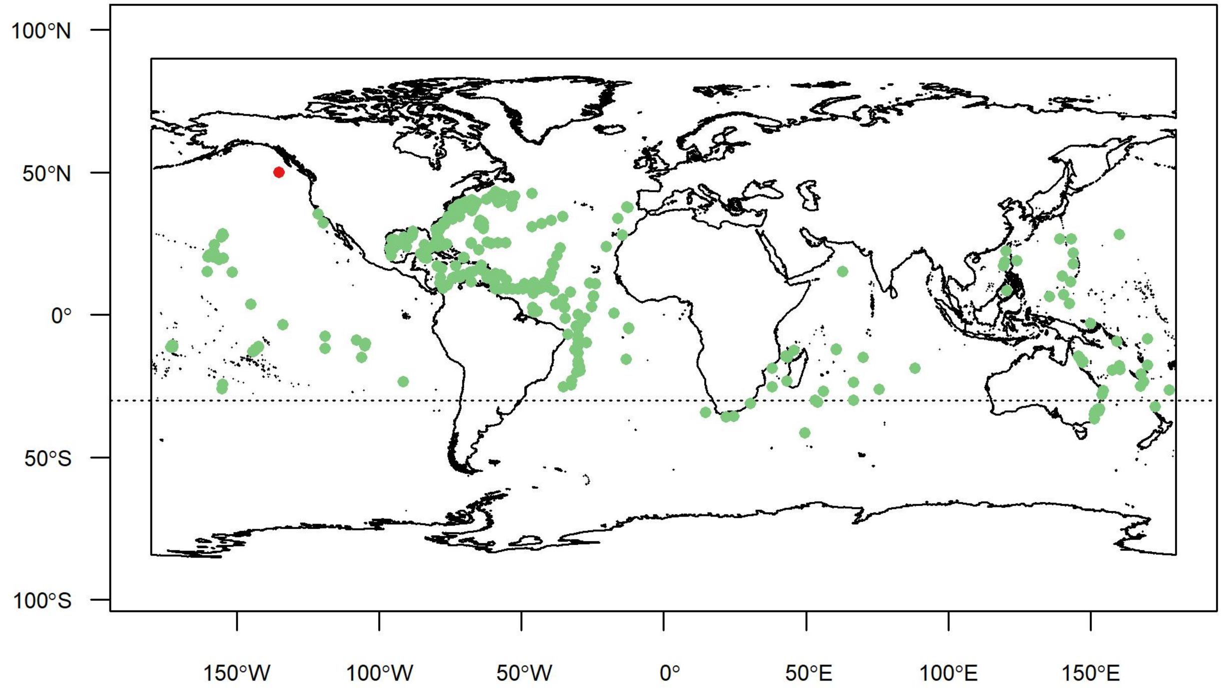

Information about the outliers :

	source	decimalLongitude	decimalLatitude	year	references
226331	GBIF	-135	50	1961	http://portal.vertnet.org/o/sio/marine-vertebrates?id=7e003807-7f0d-4f22-8837-fc9770cc9562

Diaphus coeruleus

We found no outliers for this species.

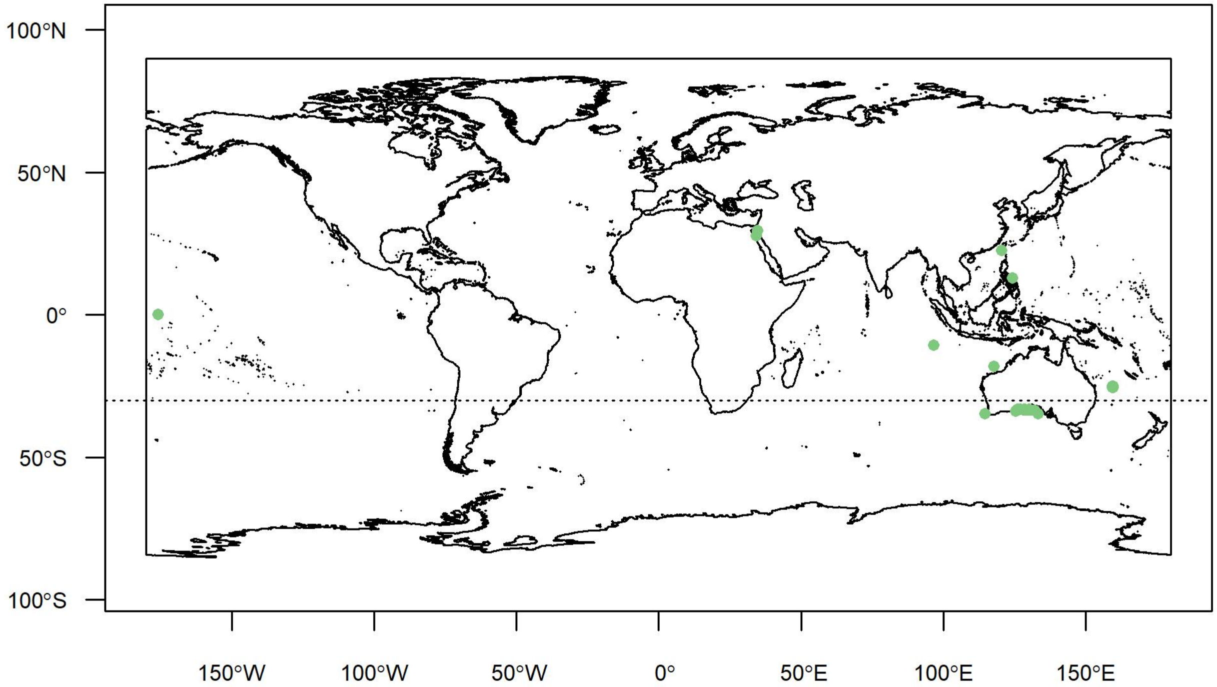

Diaphus danae

We used (Flynn & Paxton, 2012) to verify dubious points North of Australia, which we kept.

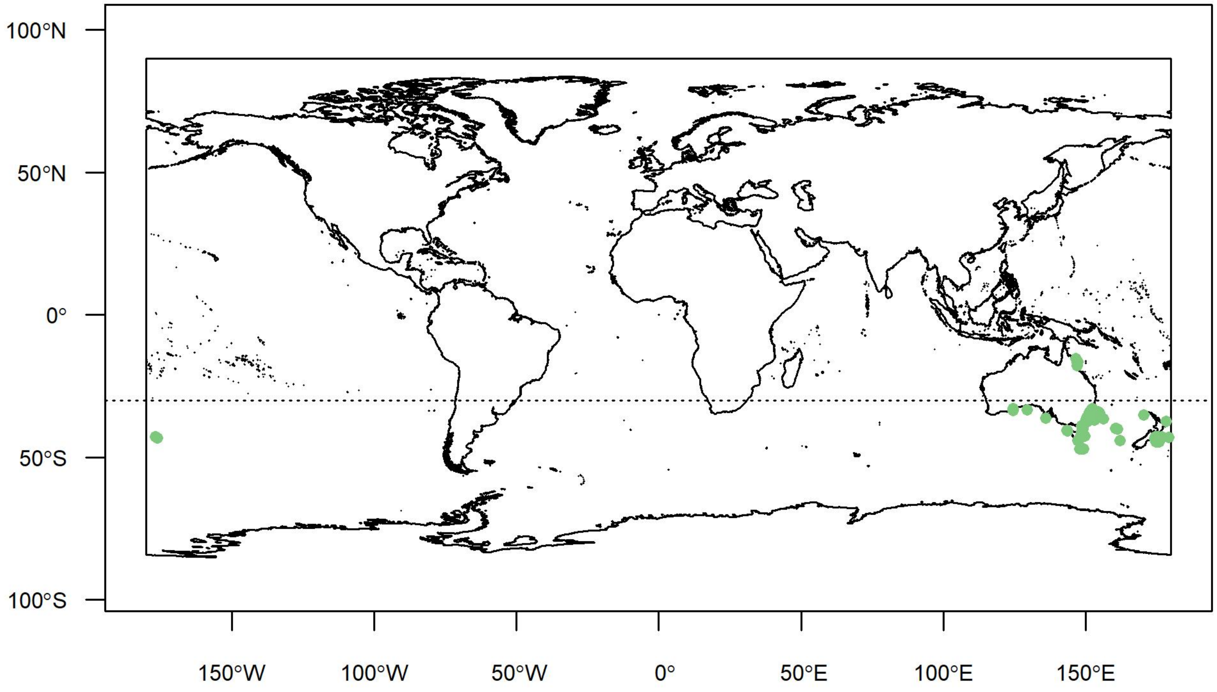

Diaphus diadematus

We found several outliers in Central Pacific (in red on the map), which we removed.

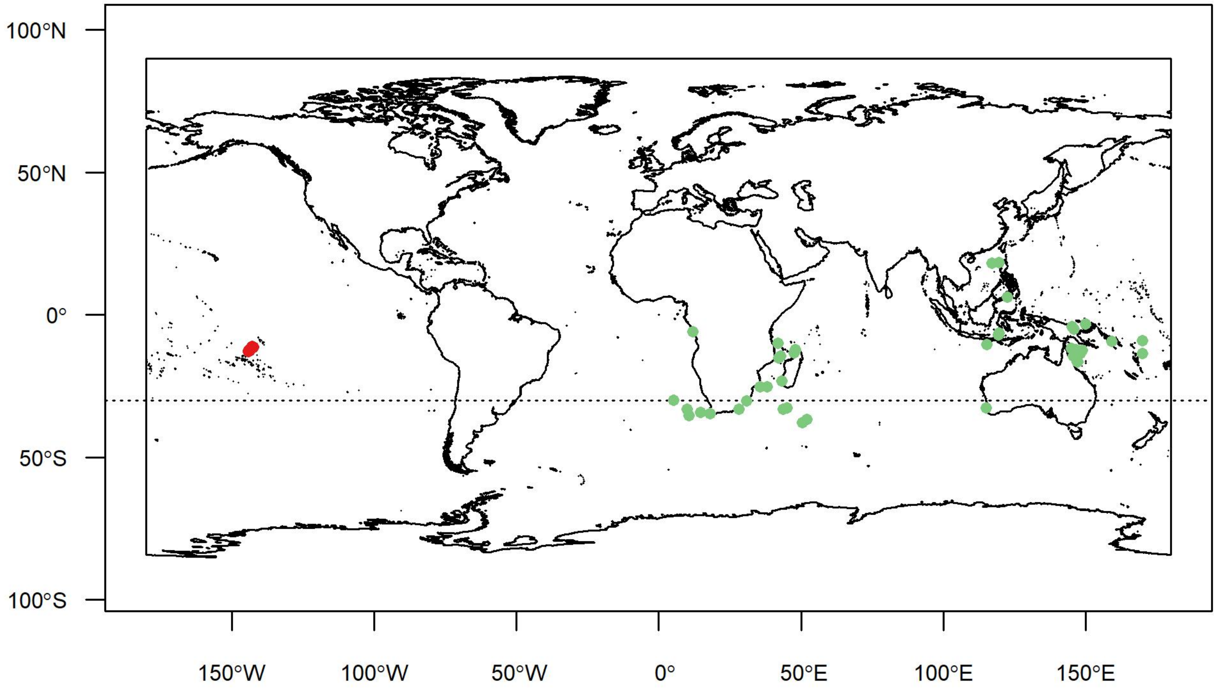

Information about the potential outliers :

	source	decimalLongitude	decimalLatitude	year	references
359310	OBIS	-143.017	-11.517	1969	
23247	OBIS	-143.167	-11.967	1968	
25385	OBIS	-143.517	-12.300	1968	
37805	OBIS	-144.217	-13.067	1969	
44088	OBIS	-143.617	-12.750	1969	
55570	OBIS	-143.633	-12.400	1968	
57896	OBIS	-144.033	-12.733	1968	

	source	decimalLongitude	decimalLatitude	year	references
60273	OBIS	-142.583	-11.617	1968	
69305	OBIS	-142.817	-11.317	1969	
75498	OBIS	-142.400	-11.450	1968	
89367	OBIS	-143.050	-11.867	1968	

Diaphus dumerilii

We found one dubious point in Western Pacific, which we kept after verification.

We used (Cavallaro et al, 2017) to verify dubious points in the Indian Ocean and Ireland, which we kept.

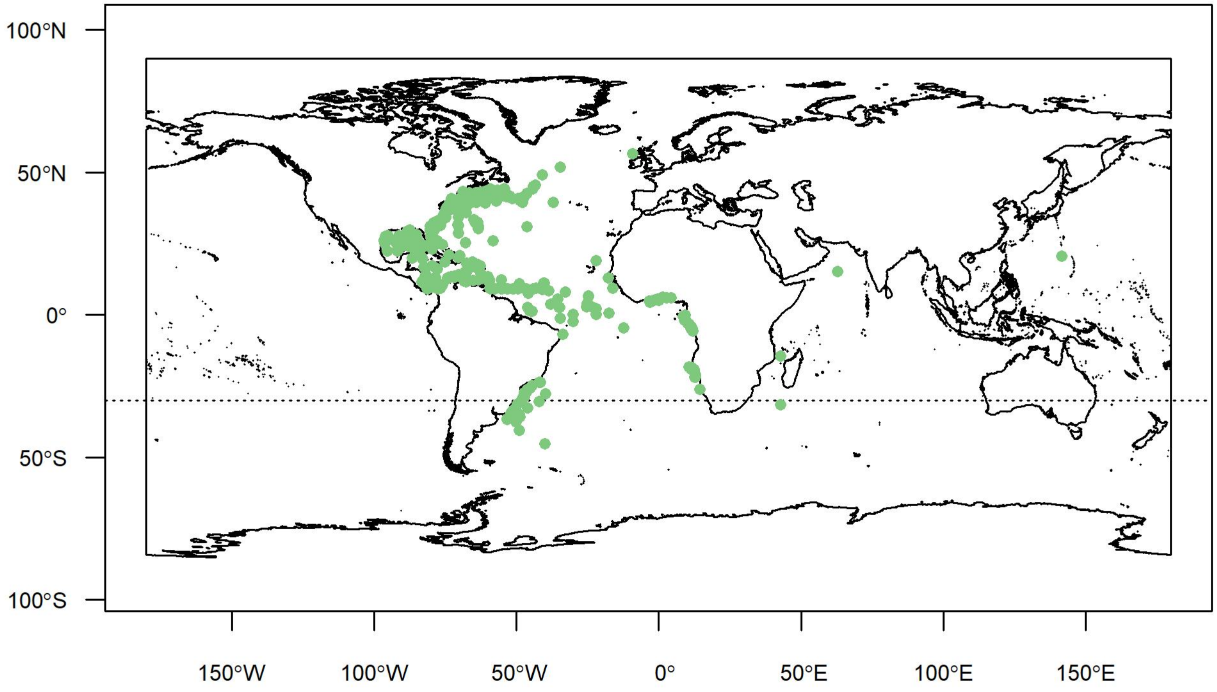

Diaphus effulgens

We found no outliers for this species.

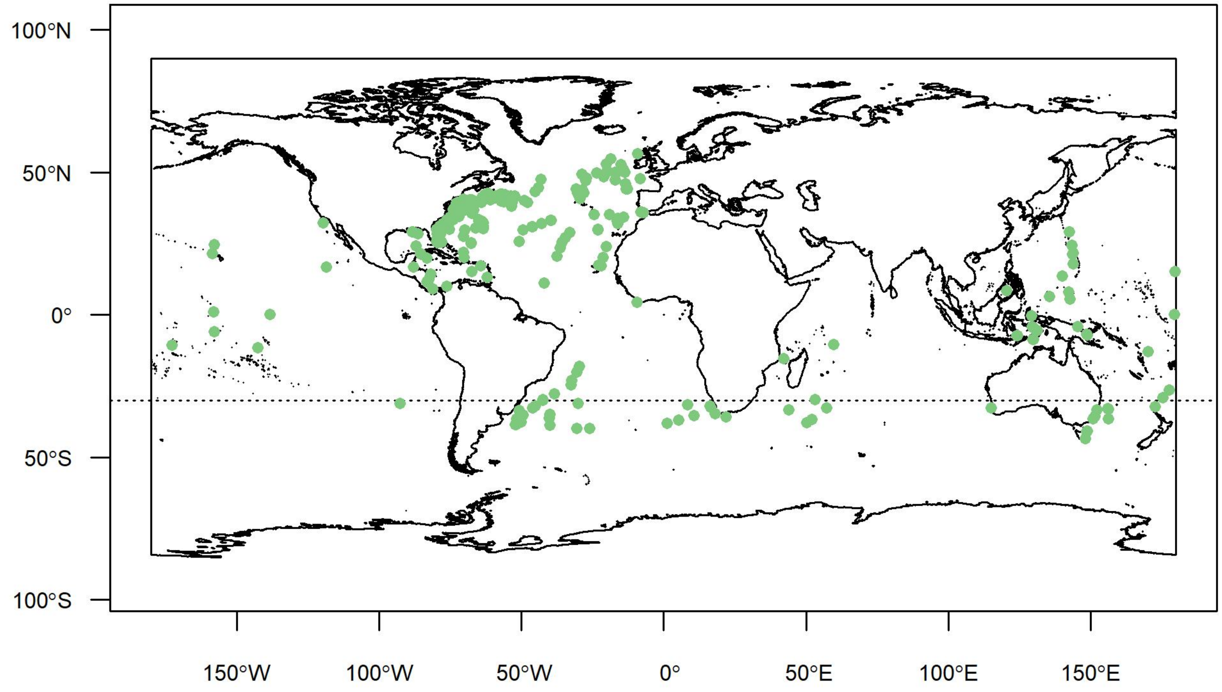

Diaphus fragilis

We found no outliers for this species.

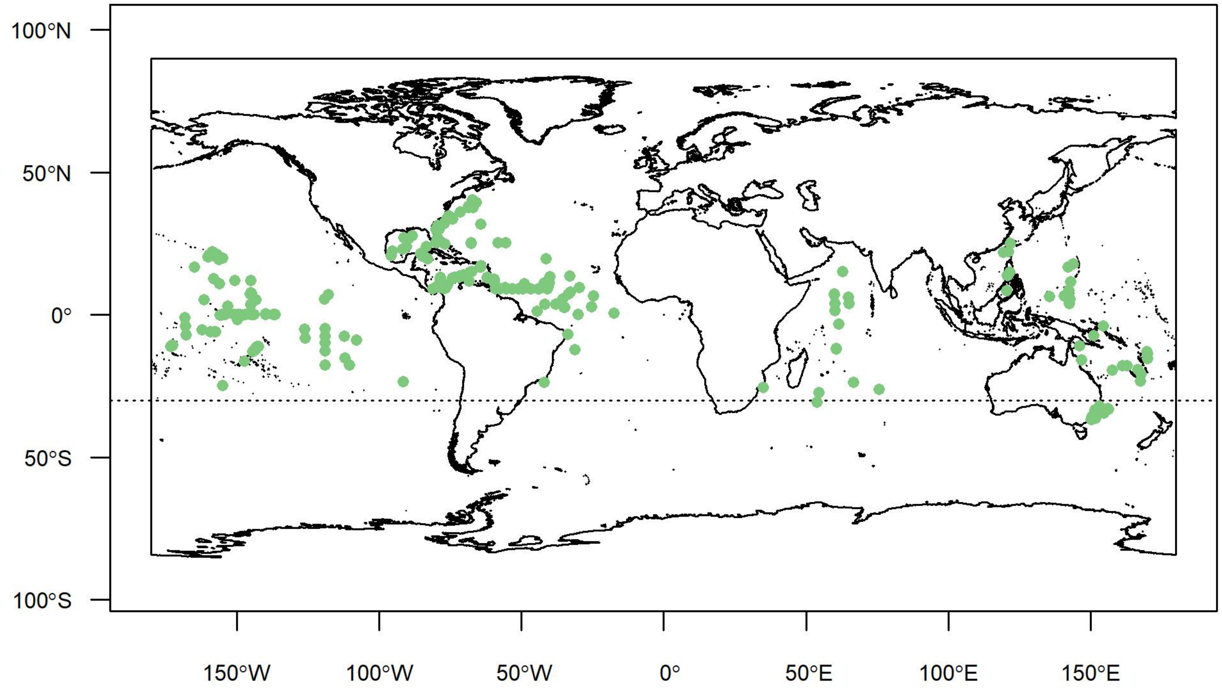

Diaphus garmani

We found no outliers for this species.

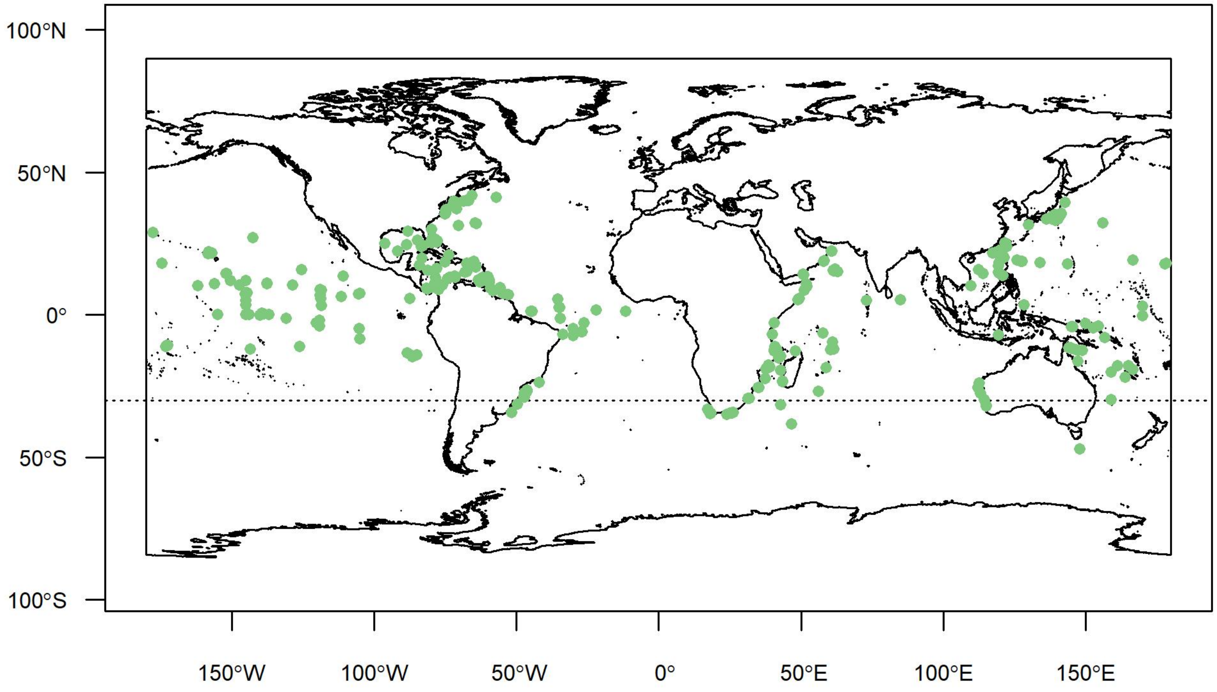

Diaphus holti

According to (Sutton et al, 2020), this species does not occur South of the Atlantic Ocean, so we removed it from our dataset.

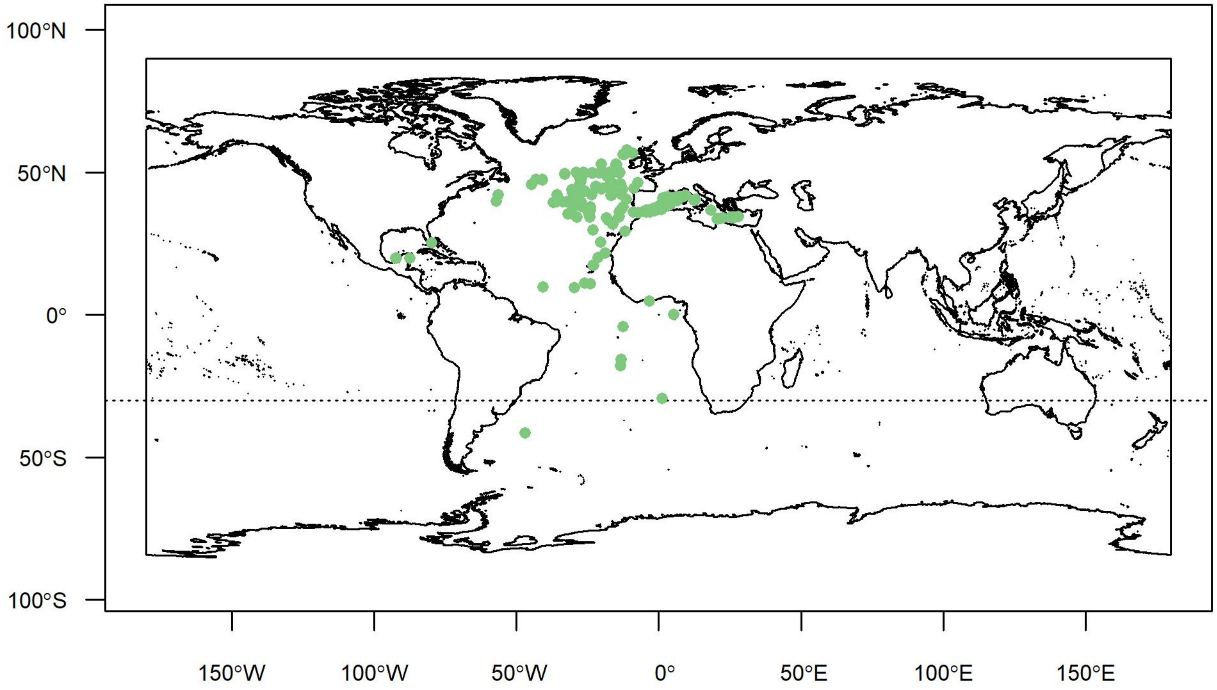

Diaphus hudsoni

We found only one apparent outlier in New Caledonia (in red in the figure), which we removed from the dataset. We also found a few data points close to the Antarctic continent which correspond to swapped latitudes and longitudes in the GBIF data (in blue in the figure). This is confirmed by the links to VerNet in the id tables below, which indicate that these records should be located off the coast of Chile. We swapped latitude and longitude for these specific data points in order to correct this error (corrected coordinates are shown in purple on the map).

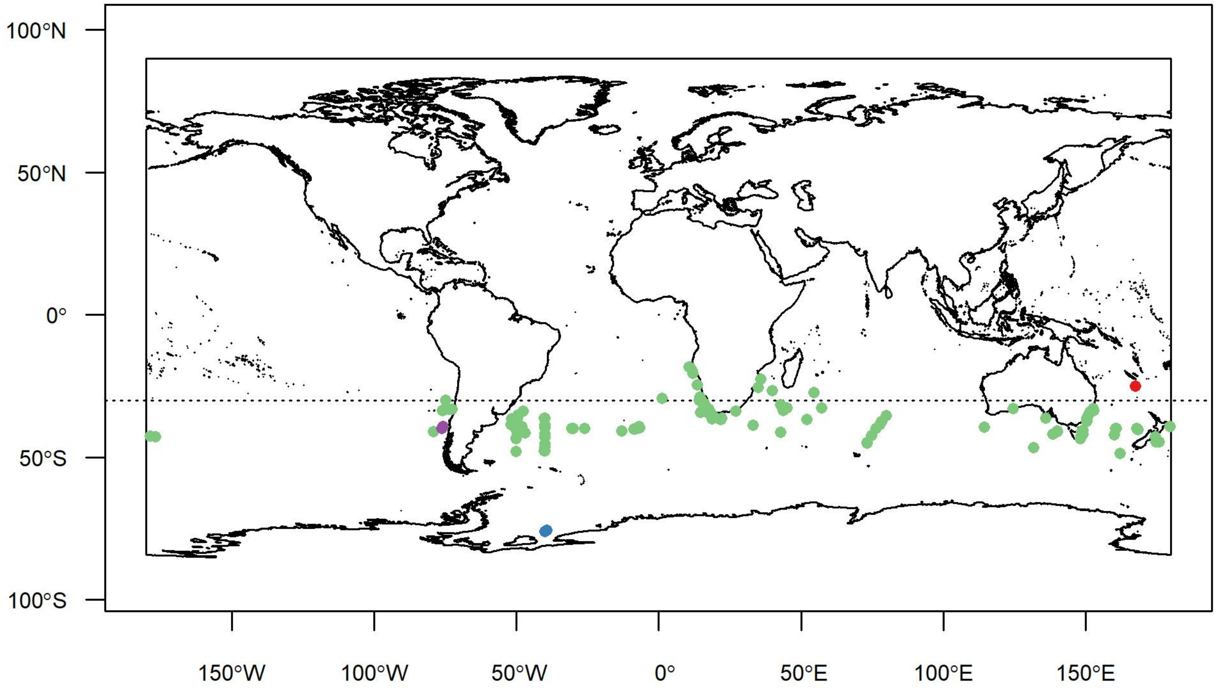

Information about New Caledonia outliers are indicated here:

	source	decimalLongitude	decimalLatitude	year	references
39160	OBIS	167.4999	-25.0618	2014	

Information about Eltanin records with swapped coordinates are indicated here:

	source	year	references	bibliographicCitation
581381	GBIF	1964	http://portal.vertnet.org/o/lacm/fish?id=279960ad-ad42-4ab4-9fc2-d8eac16fc9c0	279960AD-AD42-4AB4-9FC2-D8EAC16FC9C0. http://ipt.vertnet.org:8080/ipt/resource.do?r=lacm_verts.
652291	GBIF	1964	http://portal.vertnet.org/o/lacm/fish?id=95f1931f-9339-4b0f-8554-93a77f9fb7dd	95F1931F-9339-4B0F-8554-93A77F9FB7DD. http://ipt.vertnet.org:8080/ipt/resource.do?r=lacm_verts.

Diaphus kapalae

We found two potential outliers in the Indian Ocean and Philippines. After verification, we only removed the Indian Ocean record (red point on the map).

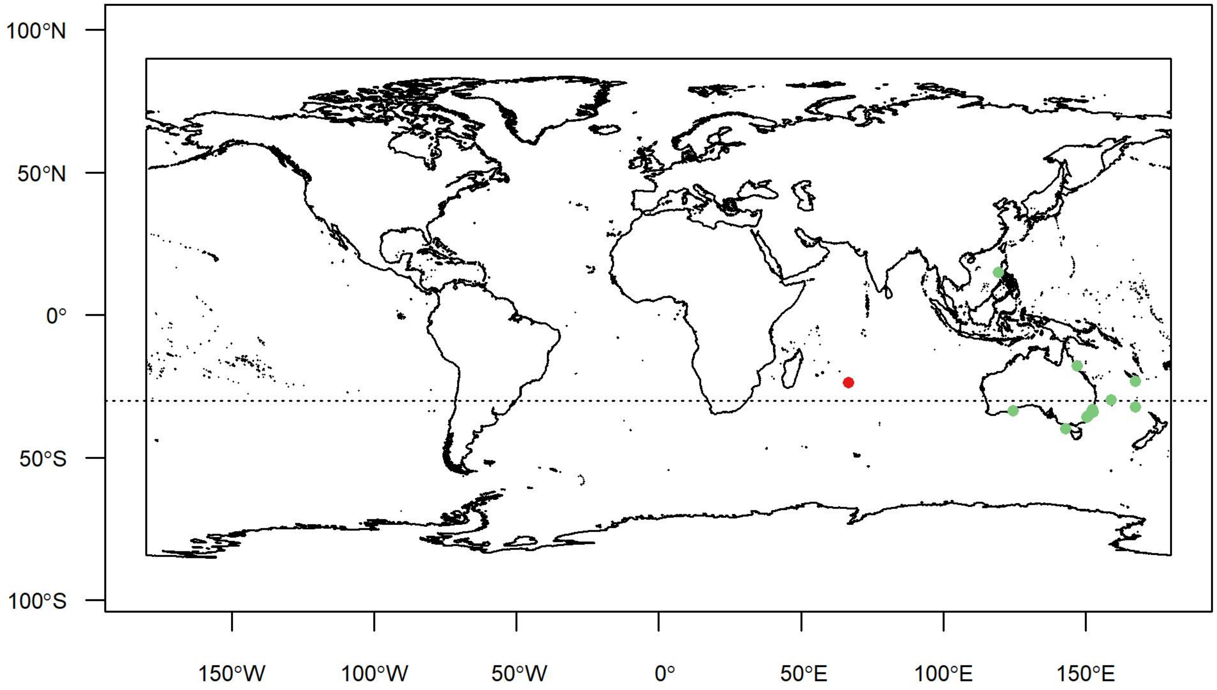

Information about the outliers :

	source	decimalLongitude	decimalLatitude	year	references
150385	GBIF	66.7195	-23.84	2014	

Diaphus lucidus

We found no outliers for this species.

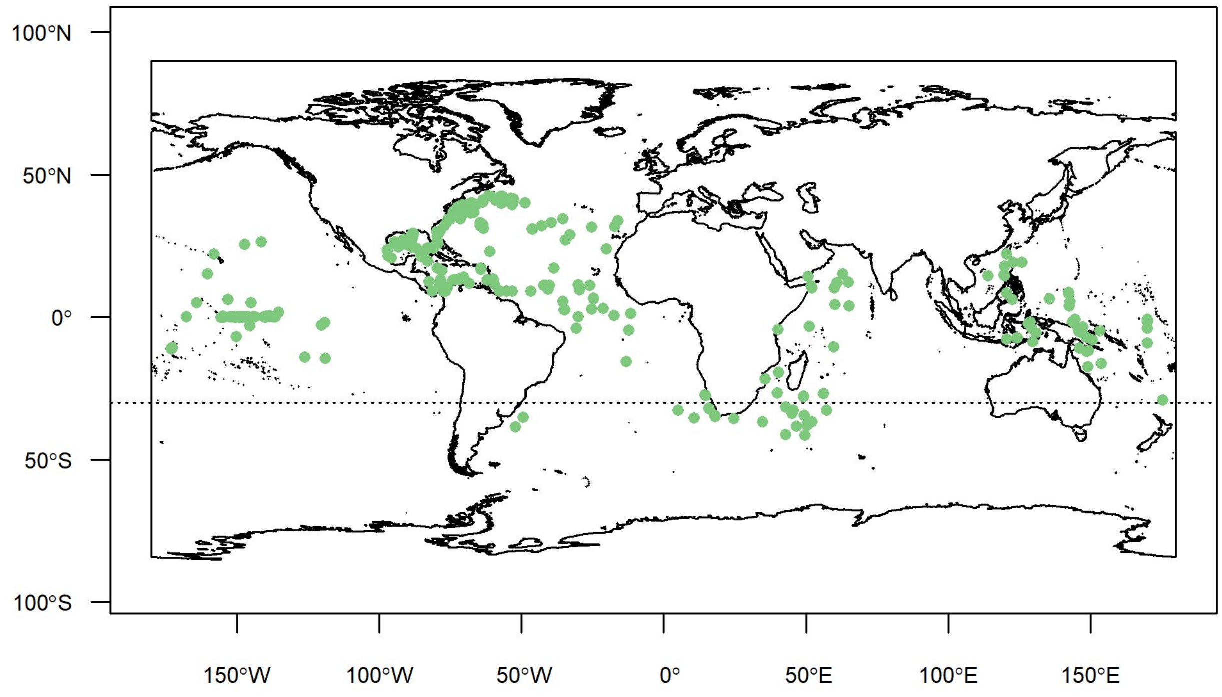

Diaphus luetkeni

We found one outlier in Alaska (in red on the map), whiwh we removed.

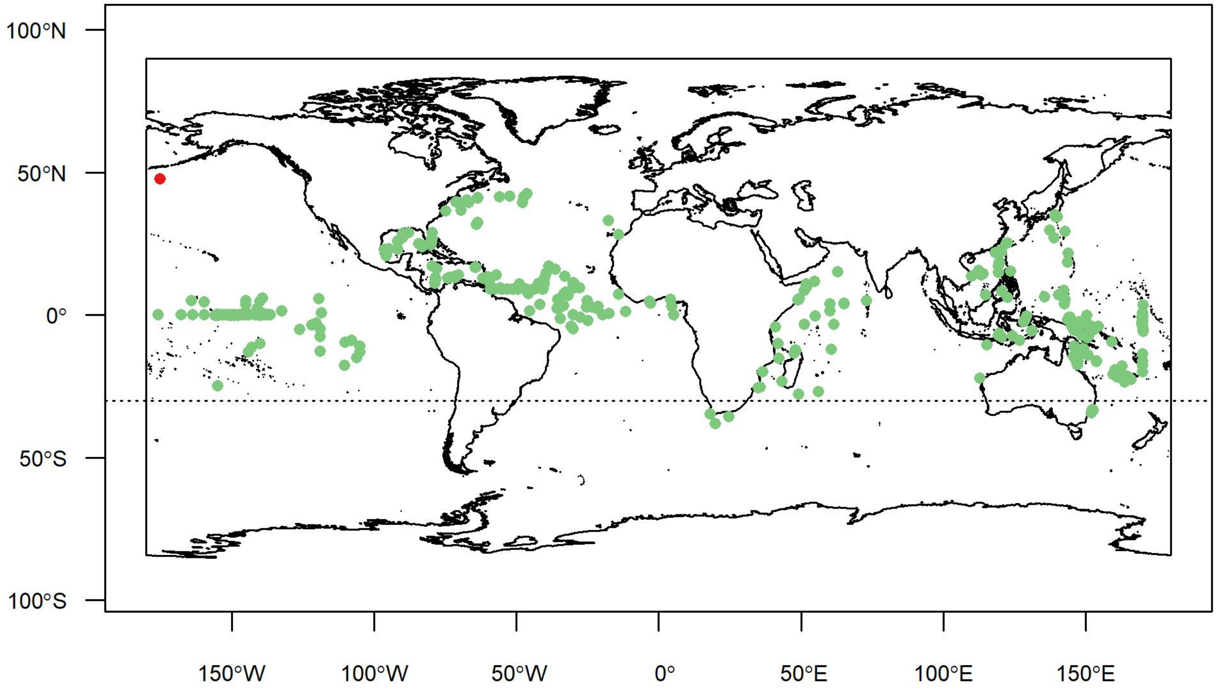

Information about the outlier :

	source	decimalLongitude	decimalLatitude	year	references
876261	GBIF	-175.1833	47.75	1966	

Diaphus mascarensis

We found no outliers for this species.

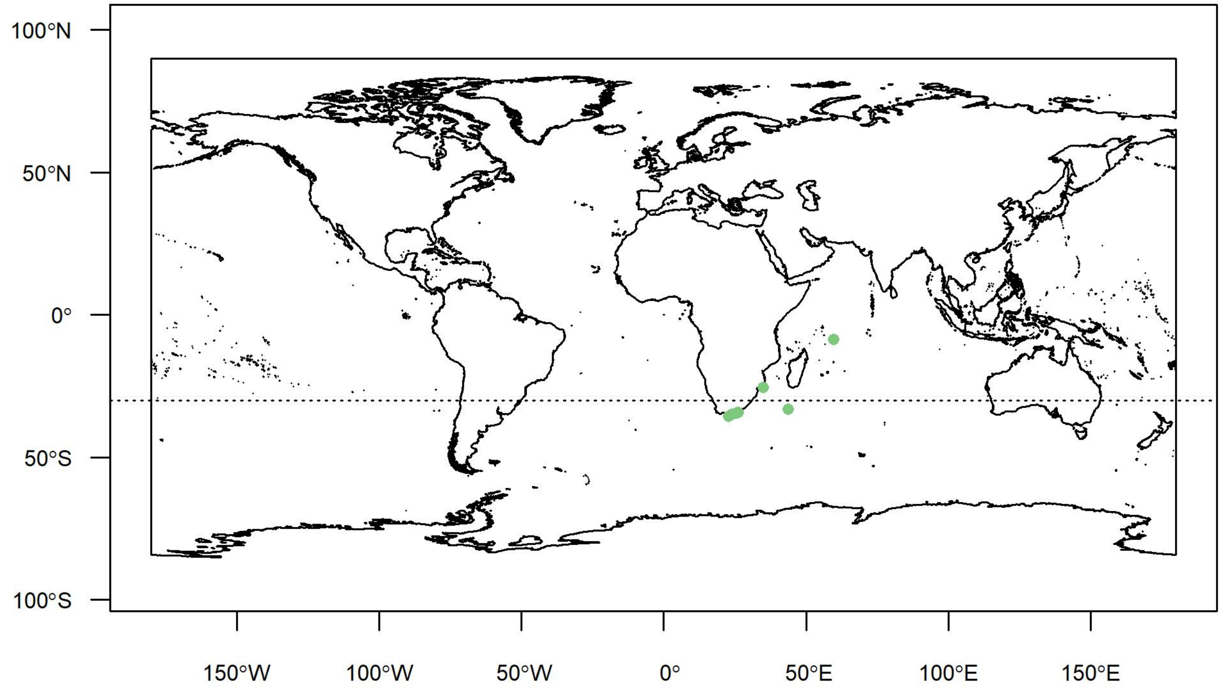

Diaphus meadi

We found one outlier close to Japan (red point on the map), which we removed.

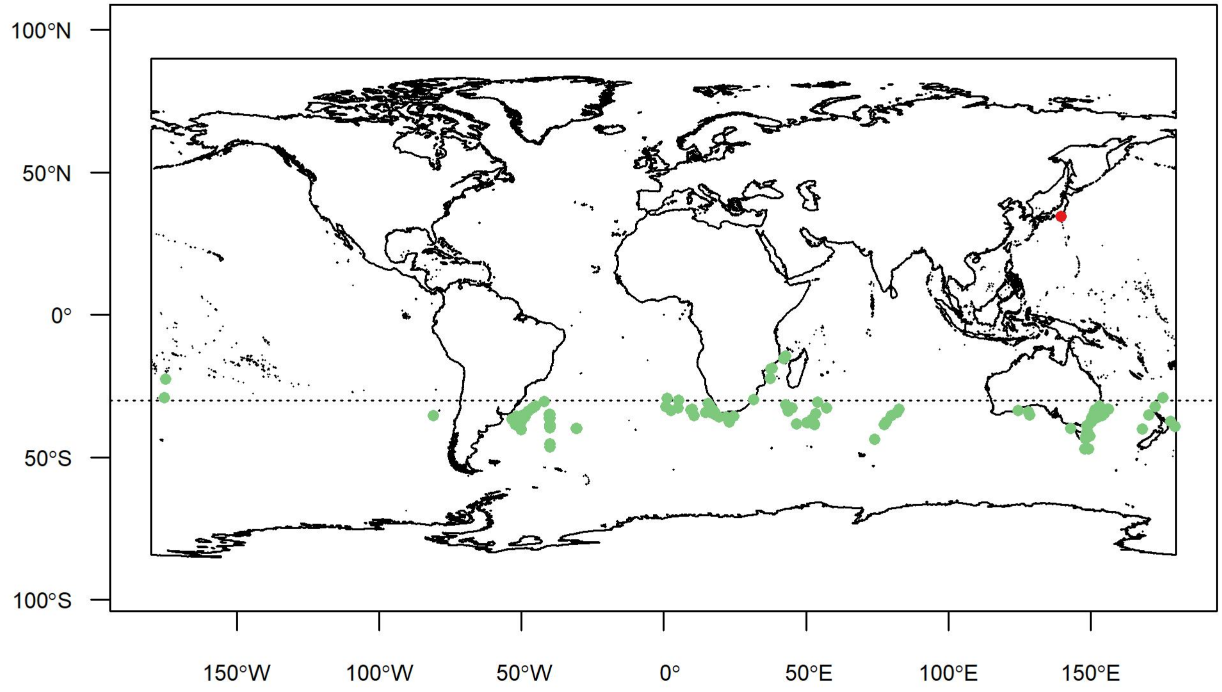

Information about the outliers :

	source	decimalLongitude	decimalLatitude	year	references
530911	GBIF	139.5908	34.4407	1983	

Diaphus metopoclampus

We found no outliers for this species.

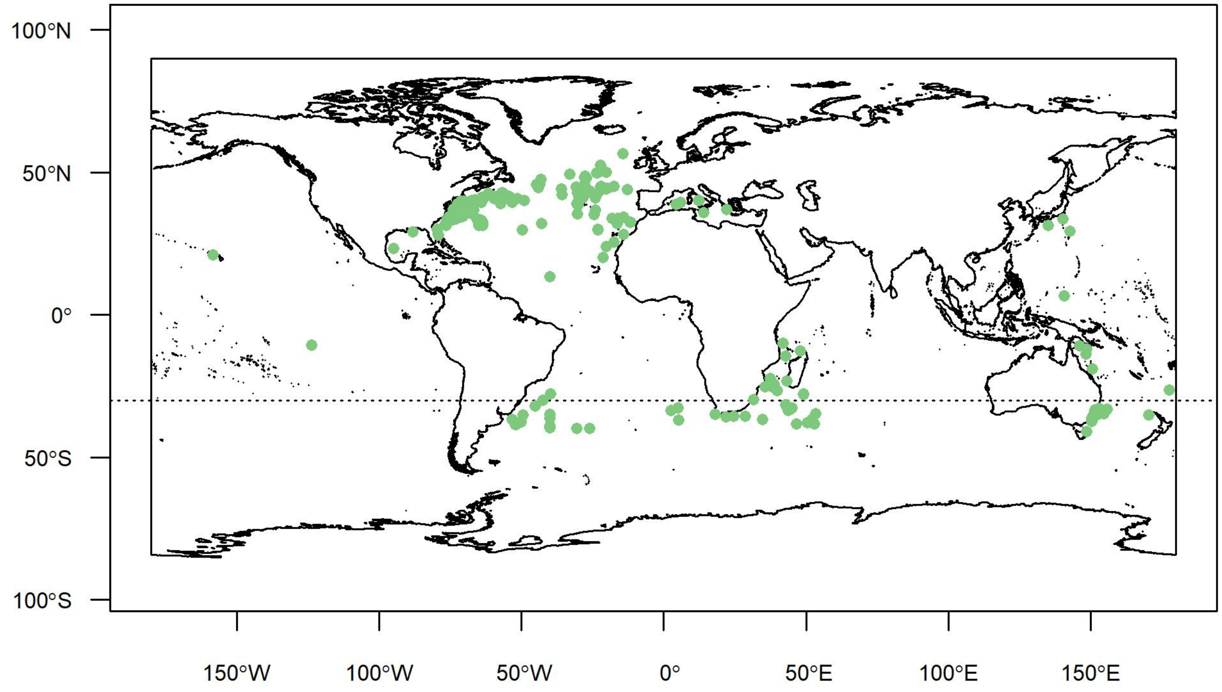

Diaphus mollis

We found no outliers for this species.

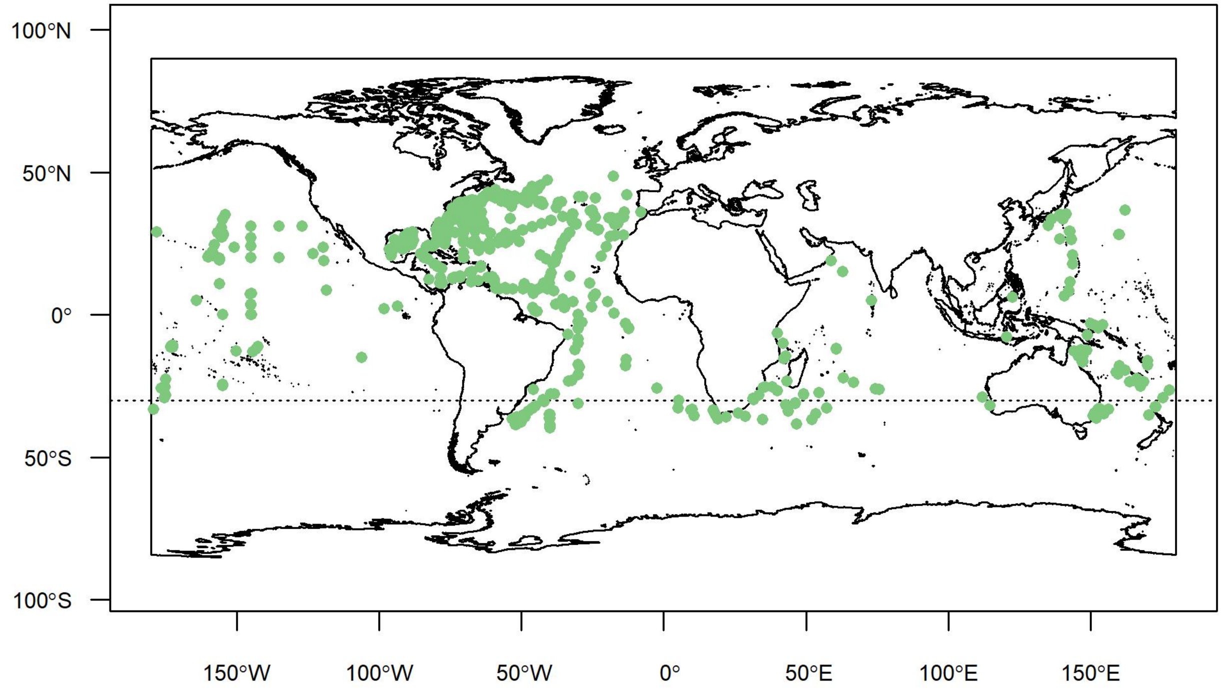

Diaphus ostenfeldi

We found two outliers in the Indian and Pacific oceans (in red on the map), which we removed

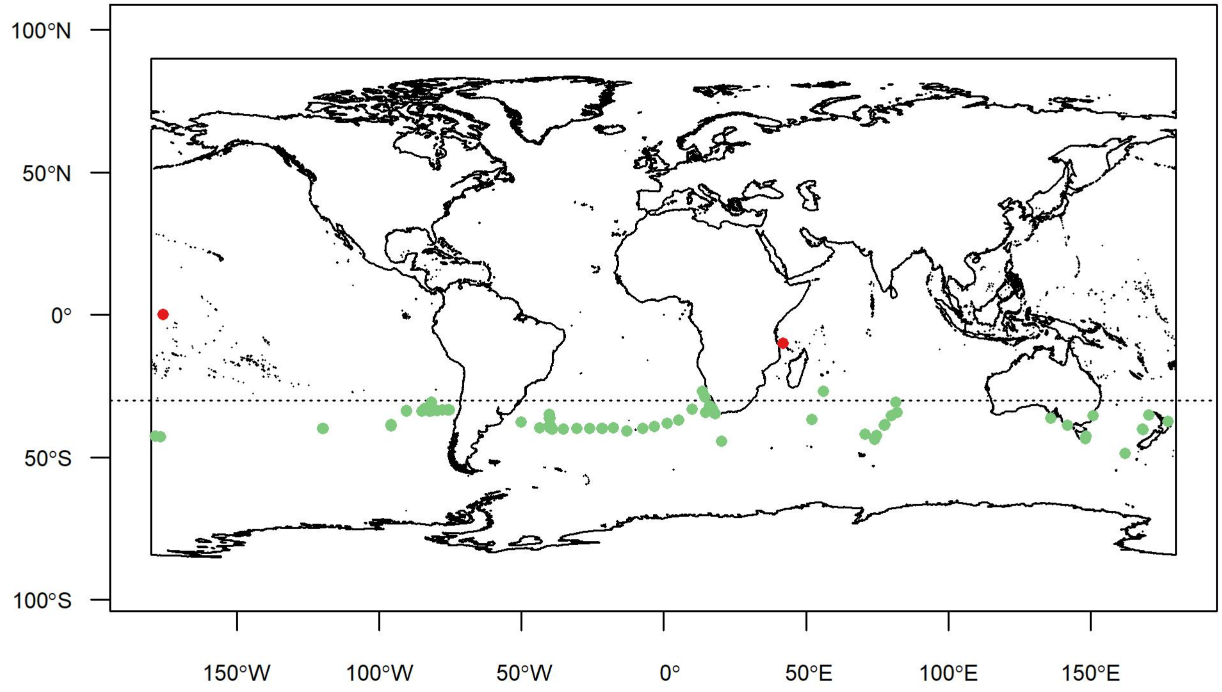

Information about the outliers :

	source	decimalLongitude	decimalLatitude	year	references
254081	GBIF	41.8950	-10.1667	1971	http://portal.vertnet.org/o/sio/marine-vertebrates?id=425d24ed-89cc-4905-aa22-e9f8dc9f5a70
136852	GBIF	-175.9283	-0.0183	1961	

Diaphus parri

We found no outliers for this species.

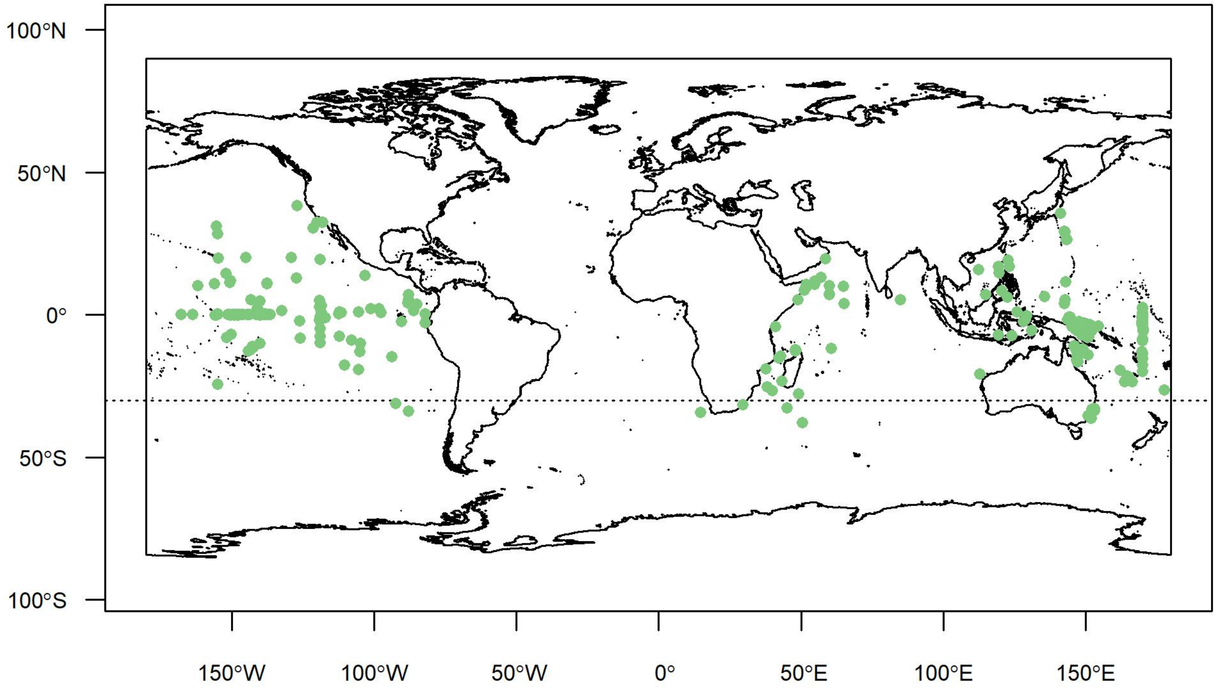

Diaphus perspicillatus

We found no outliers for this species.

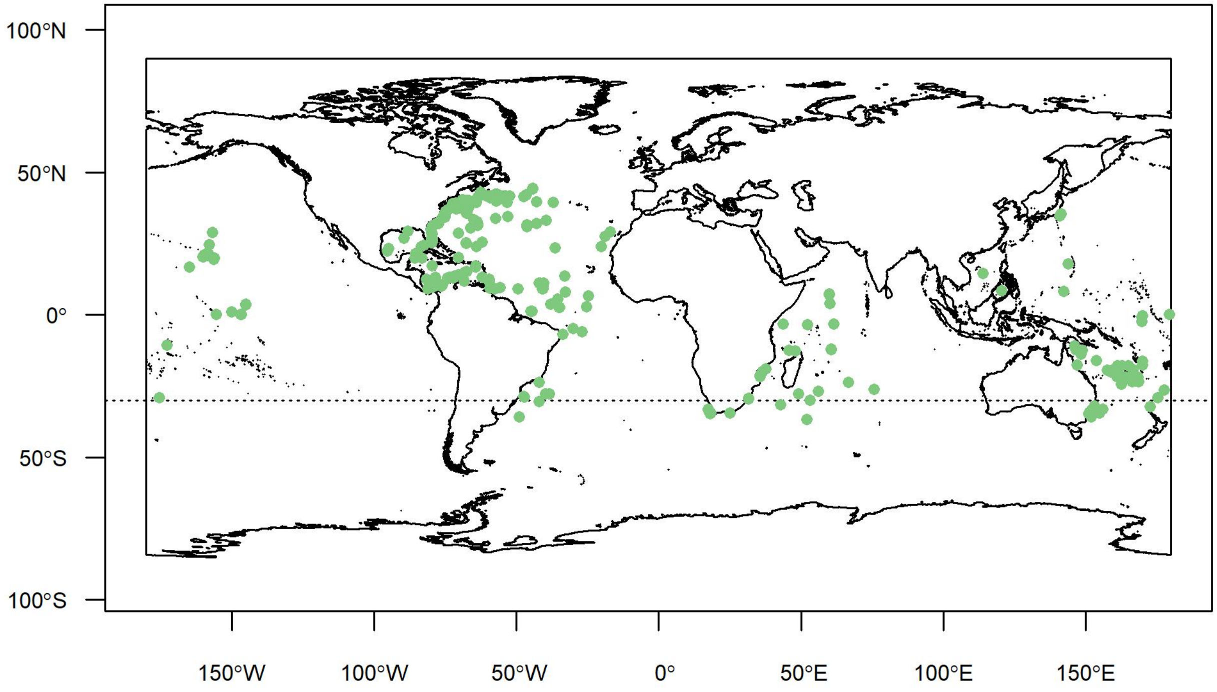

Diaphus splendidus

We removed this species because after temporal filtering there is no records left below -30° latitude.

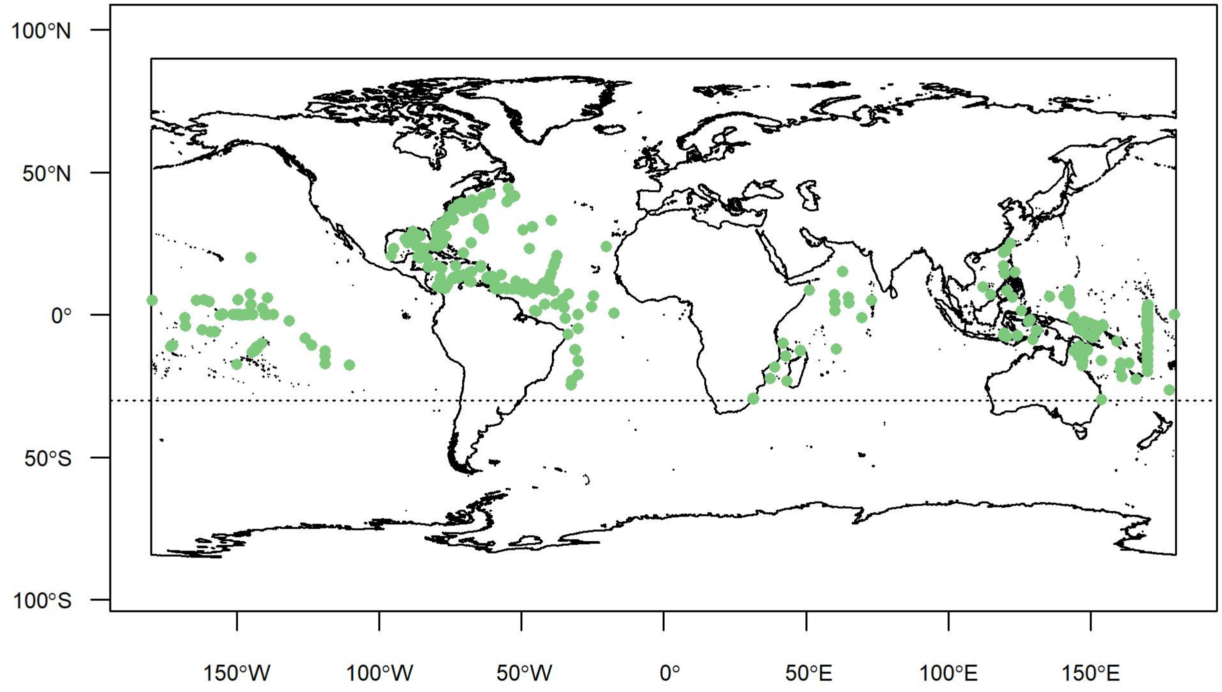

Diaphus taaningi

We considered that all records from the Indian and Pacific Oceans for this species were dubious (red points on the map), so we removed this species from our dataset since it does not occur below -30° latitude.

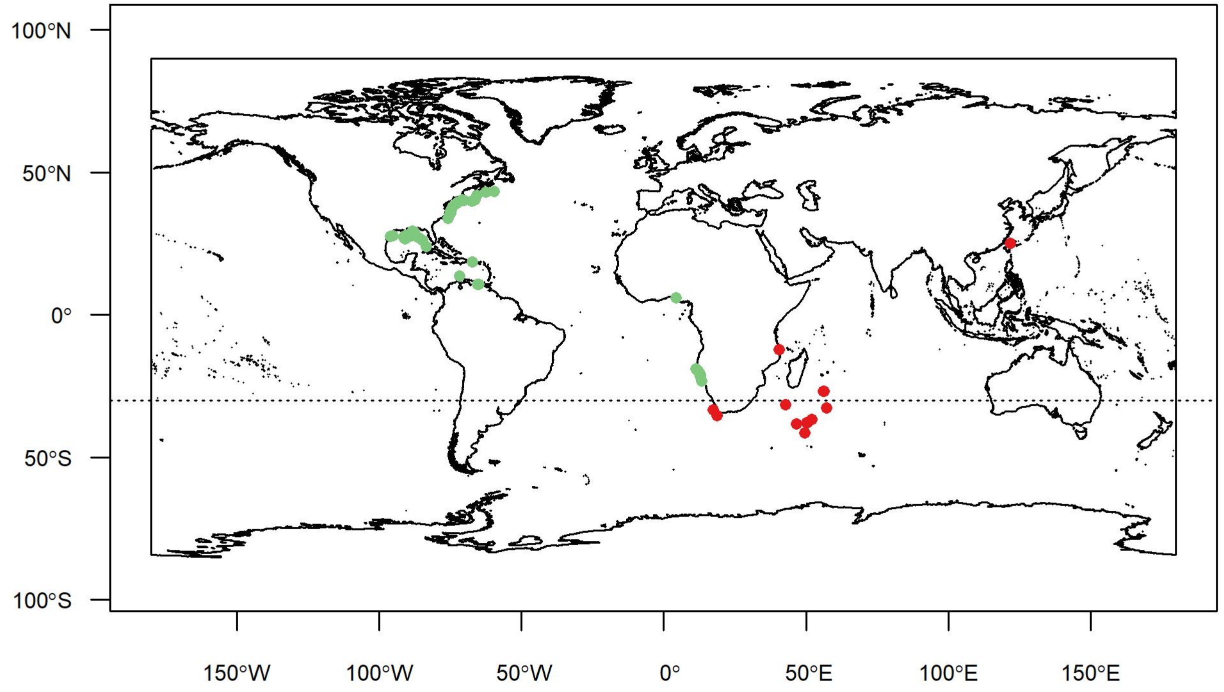

Information about the outliers :

	source	decimalLongitude	decimalLatitude	year	references
46329	OBIS	18.7000	-35.3833	2000	
886461	GBIF	40.6765	-12.4260	2007	
886981	GBIF	56.2370	-26.9410	2009	
887001	GBIF	56.1890	-26.9310	2009	
887831	GBIF	17.4667	-33.5333	2013	
888381	GBIF	50.3767	-37.9552	2009	
888641	GBIF	50.4213	-37.9532	2009	

	source	decimalLongitude	decimalLatitude	year	references
888881	GBIF	50.4227	-37.9584	2009	
888912	GBIF	49.5036	-41.5095	2009	
889161	GBIF	49.5343	-41.4796	2009	
889391	GBIF	56.2825	-26.9469	2009	
889521	GBIF	57.2970	-32.7247	2009	
889721	GBIF	57.2970	-32.7269	2009	
890261	GBIF	56.2368	-26.9407	2009	
890461	GBIF	57.2968	-32.7252	2009	
890741	GBIF	50.4029	-37.9560	2009	
893601	GBIF	46.7589	-38.5044	2009	
893791	GBIF	52.0539	-36.8556	2009	
896521	GBIF	42.8133	-31.6475	2009	
176205	GBIF	121.9009	24.9411	2009	

Diaphus termophilus

We found two outliers in the Indian Ocean (in red on the map), which we removed.

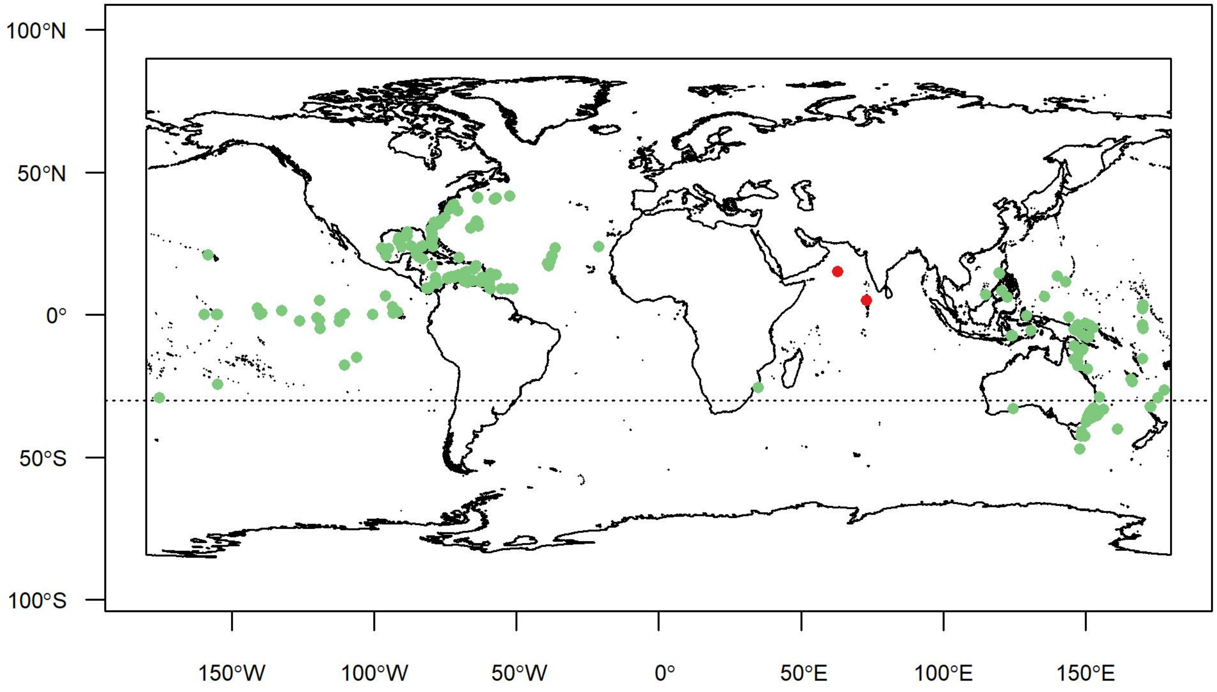

Information about the outliers :

	source	decimalLongitude	decimalLatitude	year	references
138932	GBIF	63.0000	15.00	1974	
176181	GBIF	73.1167	5.05	1964	

Diaphus theta

This species is often subject to misidentification, so we decided to remove it from our dataset.

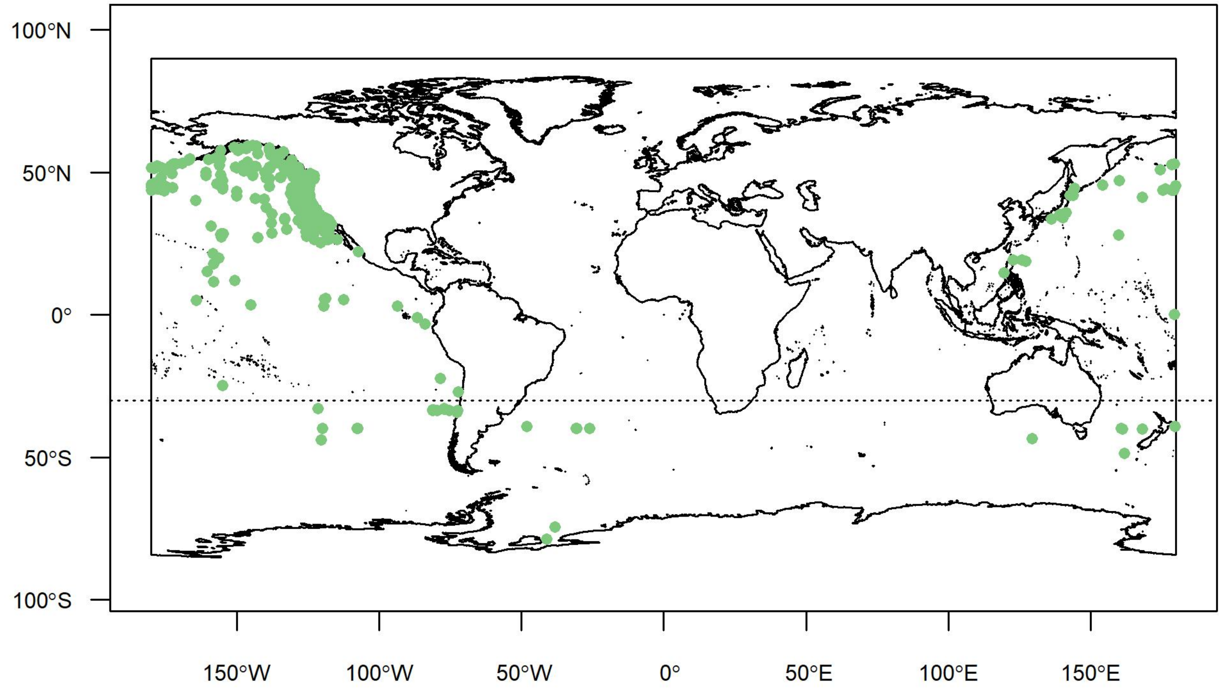

Diaphus watasei

We found one outlier in the Pacific Ocean (red point on the map), which we removed.

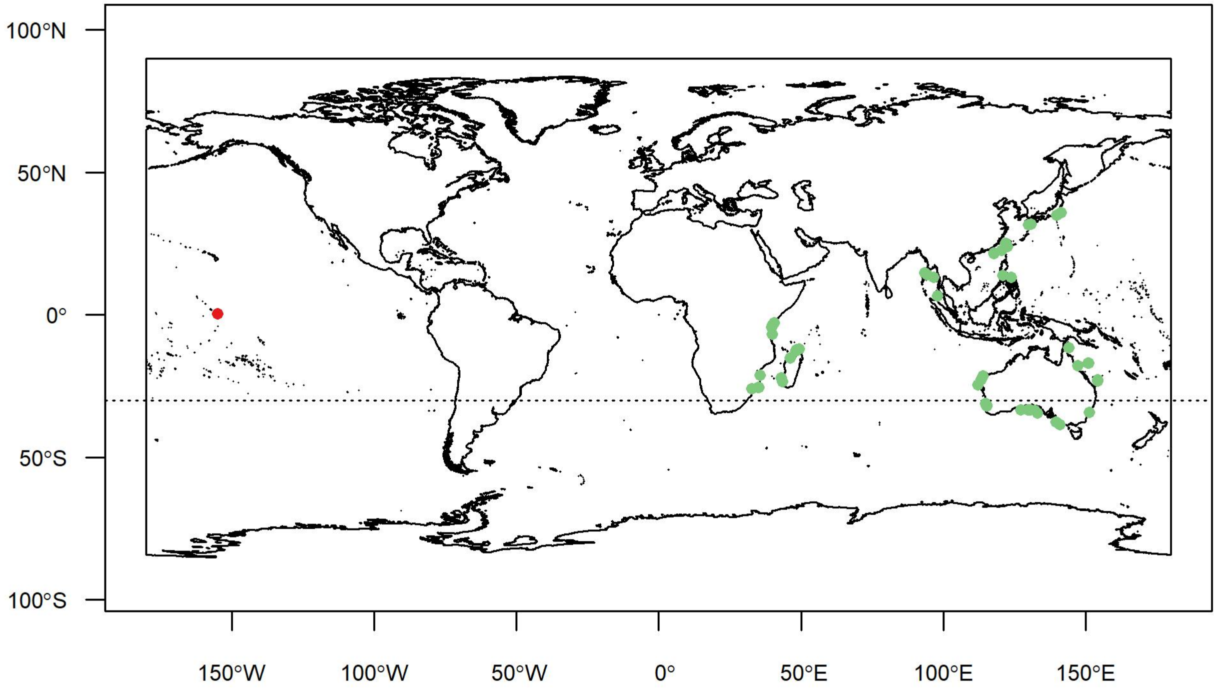

Information about the outlier :

	source	decimalLongitude	decimalLatitude	year	references
319941	GBIF	-155	0.1433	1972	http://portal.vertnet.org/o/sio/marine-vertebrates?id=8b0fbf10-774f-4e9c-9bdd-48831dbab8cb

Diogenichthys atlanticus

We found no outliers for this species.

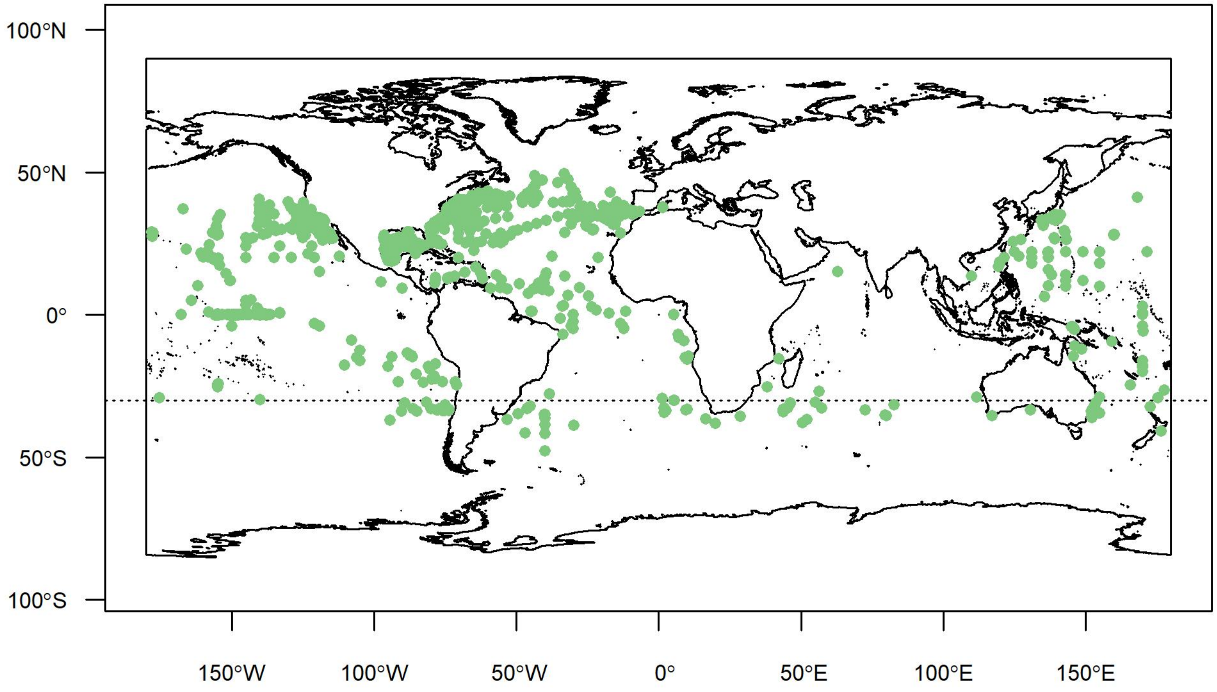

Diogenichthys panurgus

We found no outliers for this species.

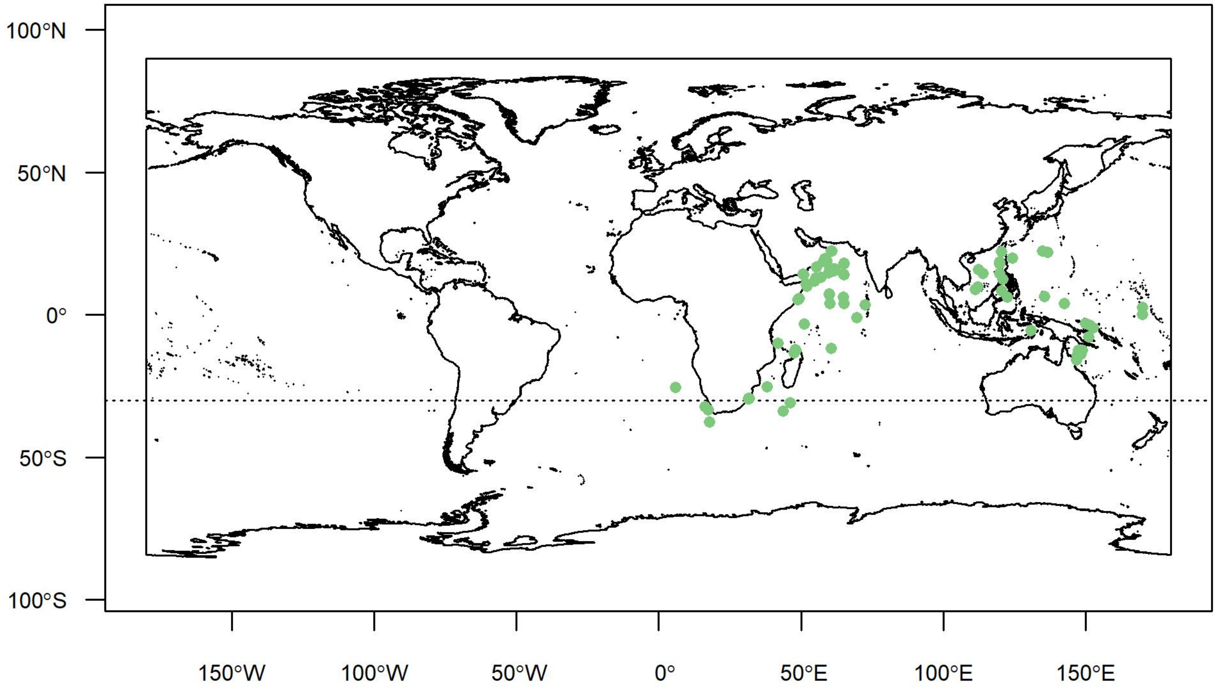

Electrona antarctica

One record of this species corresponded to the batch of swapped coordinates of Eltanin expeditions (in purple on the map), which we corrected (in blue on the map).

Several outliers were detected in the Southern hemisphere (in red on the map), and were removed because specimen could not be accessed for record verification.

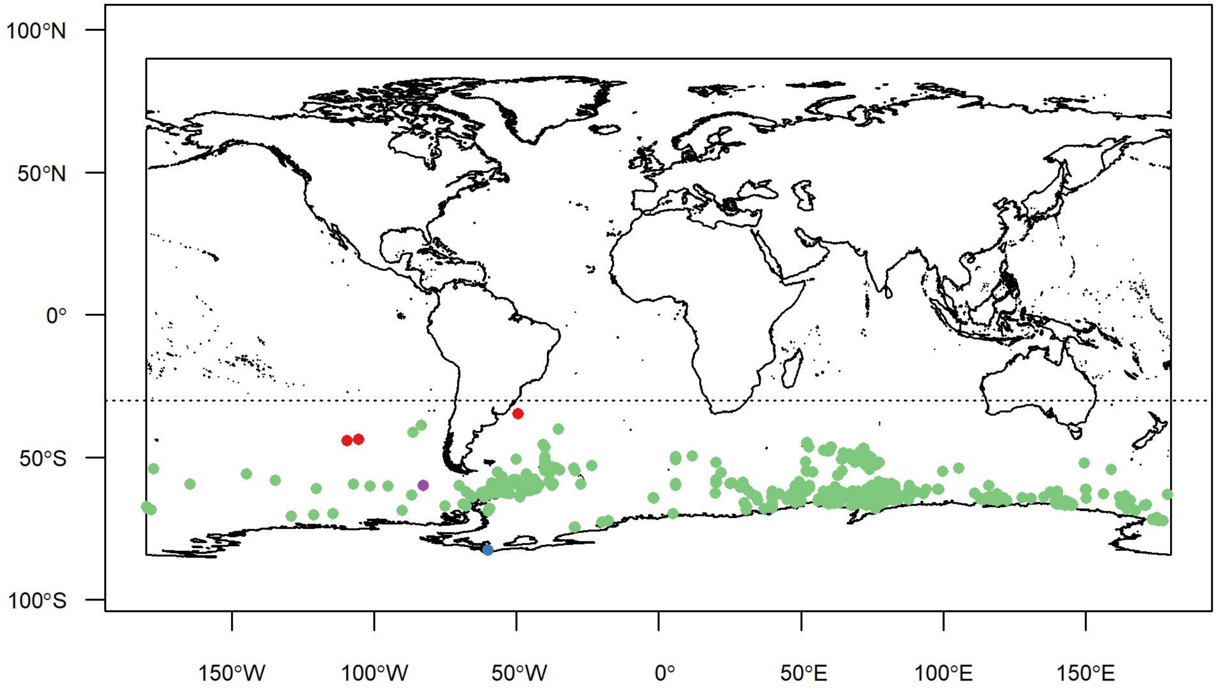

Information about Eltanin records with swapped coordinates are indicated here:

	source	year	references	bibliographicCitation
560541	GBIF	1963	http://portal.vertnet.org/o/lacm/fish?id=080281ec-047f-4a95-9967-76faa806bbe3	080281EC-047F-4A95-9967-76FAA806BBE3. http://ipt.vertnet.org:8080/ipt/resource.do?r=lacm_verts.

Information about the outliers :

	source	decimalLongitude	decimalLatitude	year	references
16586	MyctoDB	-109.5500	-44.3000	1953	Downwind
16694	MyctoDB	-105.5000	-43.8333	1953	Downwind
18400	MyctoDB	-49.4667	-34.7167	1967	RV_Atlantis_II_031
199652	GBIF	-105.5000	-43.8333	1957	http://portal.vertnet.org/o/sio/marine-vertebrates?id=2def2dd7-4132-46fb-9d4b-fe3e9bbdb61d
200132	GBIF	-109.5500	-44.3000	1957	http://portal.vertnet.org/o/sio/marine-vertebrates?id=4352fc9c-4af2-41d6-a4a1-9aed2f0d381c
1165461	GBIF	-49.4750	-34.7250	1967	http://mczbase.mcz.harvard.edu/guid/MCZ:Ich:102916

Electrona carlsbergi

Three records were swapped because of the Eltanin GBIF errors (blue points on map), which we corrected (purple points on map).

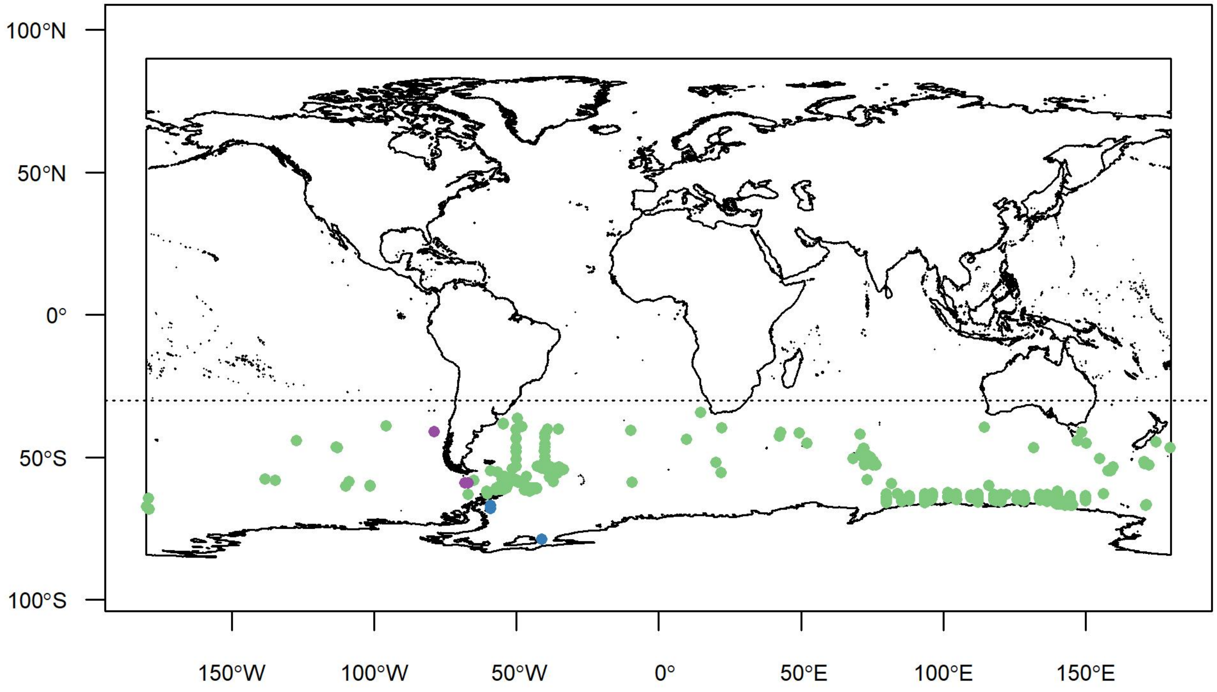

Information about Eltanin records with swapped coordinates are indicated here:

	source	year	references	bibliographicCitation
616131	GBIF	1962	http://portal.vertnet.org/o/lacm/fish?id=5d5171ac-7c20-41aa-8da9-acd34dca98c4	5D5171AC-7C20-41AA-8DA9-ACD34DCA98C4. http://ipt.vertnet.org:8080/ipt/resource.do?r=lacm_verts.
629191	GBIF	1962	http://portal.vertnet.org/o/lacm/fish?id=7238e080-9a1a-447d-ad32-db79ef6b84a4	7238E080-9A1A-447D-AD32-DB79EF6B84A4. http://ipt.vertnet.org:8080/ipt/resource.do?r=lacm_verts.
652881	GBIF	1966	http://portal.vertnet.org/o/lacm/fish?id=970cc9eb-c919-494e-894b-6b23b71bdb36	970CC9EB-C919-494E-894B-6B23B71BDB36. http://ipt.vertnet.org:8080/ipt/resource.do?r=lacm_verts.

Electrona paucirastra

Multiple records were swapped because of the Eltanin GBIF errors (blue points on map), which we corrected (purple points on map).

One outlier in the Southern Ocean could not be verified and was deleted.

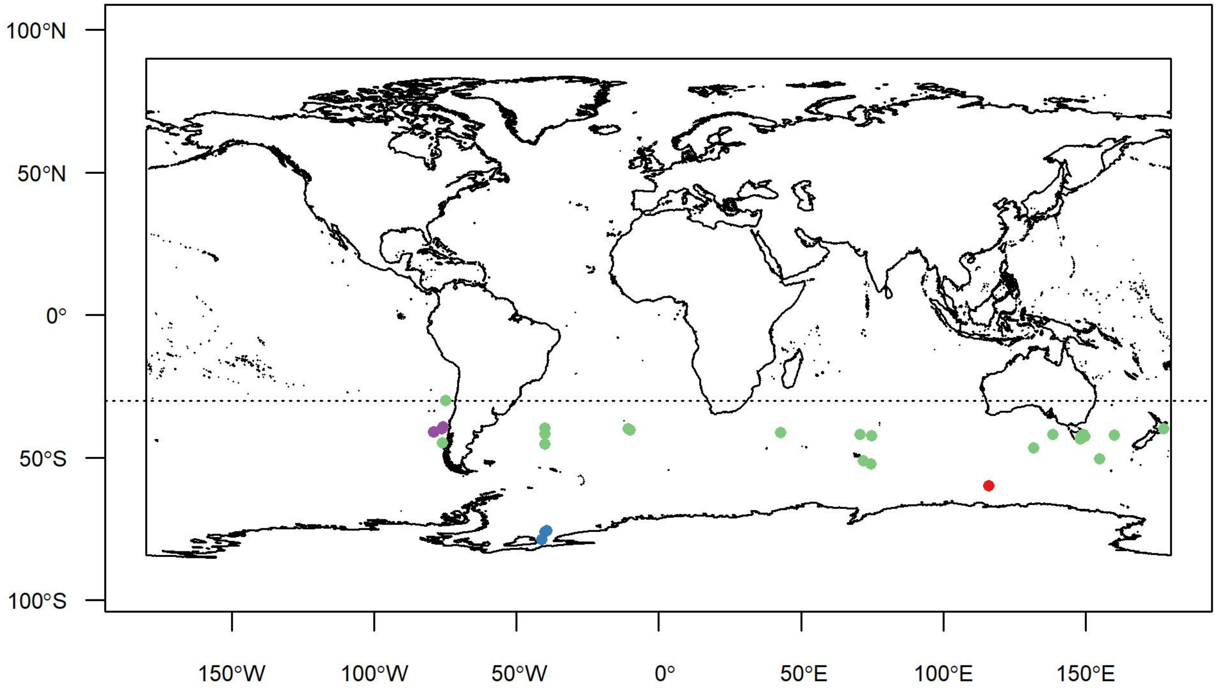

Information about Eltanin records with swapped coordinates are indicated here:

	source	year	references	bibliographicCitation
580431	GBIF	1964	http://portal.vertnet.org/o/lacm/fish?id=26261d67-1e17-4b41-a146-dce8ad3b5032	26261D67-1E17-4B41-A146-DCE8AD3B5032. http://ipt.vertnet.org:8080/ipt/resource.do?r=lacm_verts.

	source	year	references	bibliographicCitation
606271	GBIF	1966	http://portal.vertnet.org/o/lacm/fish?id=4e1fedc5-faf6-4978-a7a7-1b6b2900a125	4E1FEDC5-FAF6-4978-A7A7-1B6B2900A125. http://ipt.vertnet.org:8080/ipt/resource.do?r=lacm_verts.
701921	GBIF	1964	http://portal.vertnet.org/o/lacm/fish?id=e346ff74-4965-470d-a229-367f89dca8a7	E346FF74-4965-470D-A229-367F89DCA8A7. http://ipt.vertnet.org:8080/ipt/resource.do?r=lacm_verts.
714371	GBIF	1966	http://portal.vertnet.org/o/lacm/fish?id=f62a33a9-87dd-4707-99dd-cf8bfde4f1d8	F62A33A9-87DD-4707-99DD-CF8BFDE4F1D8. http://ipt.vertnet.org:8080/ipt/resource.do?r=lacm_verts.

Information about the outliers :

	source	decimalLongitude	decimalLatitude	year	references
529811	GBIF	116.0003	-60.0003	1984	

Electrona risso

We found no outliers for this species.

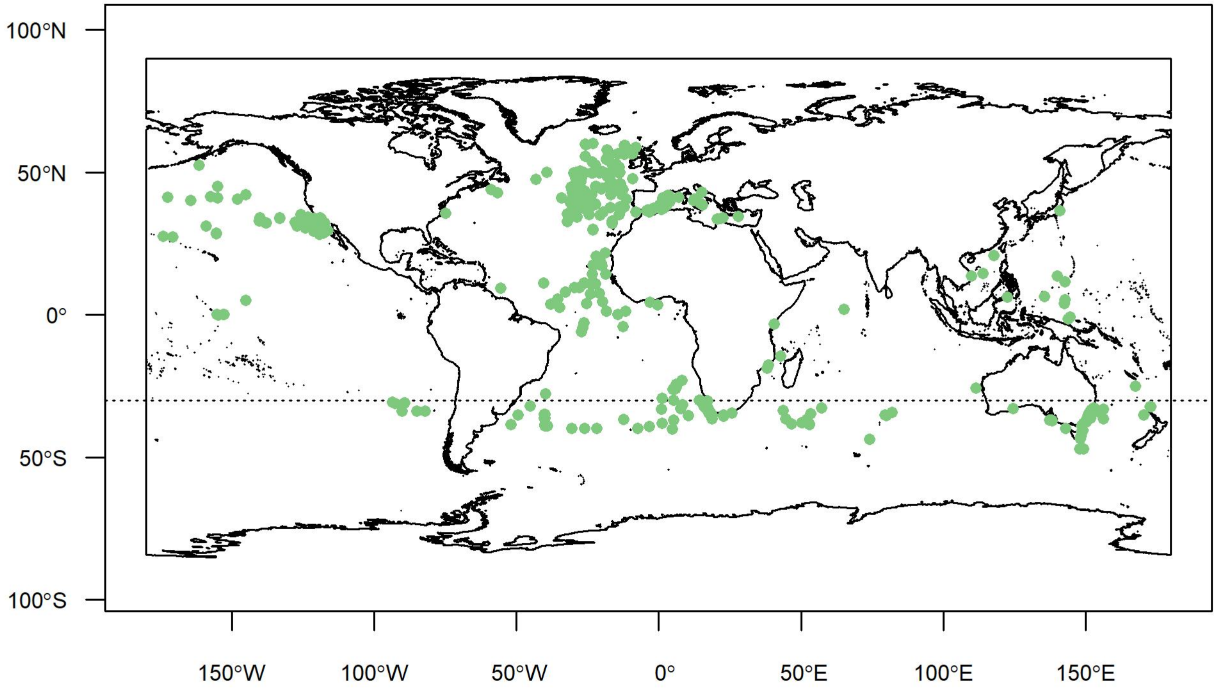

Electrona subaspera

Several records was swapped because of the Eltanin GBIF error (blue points on map), which we corrected (purple points on map).

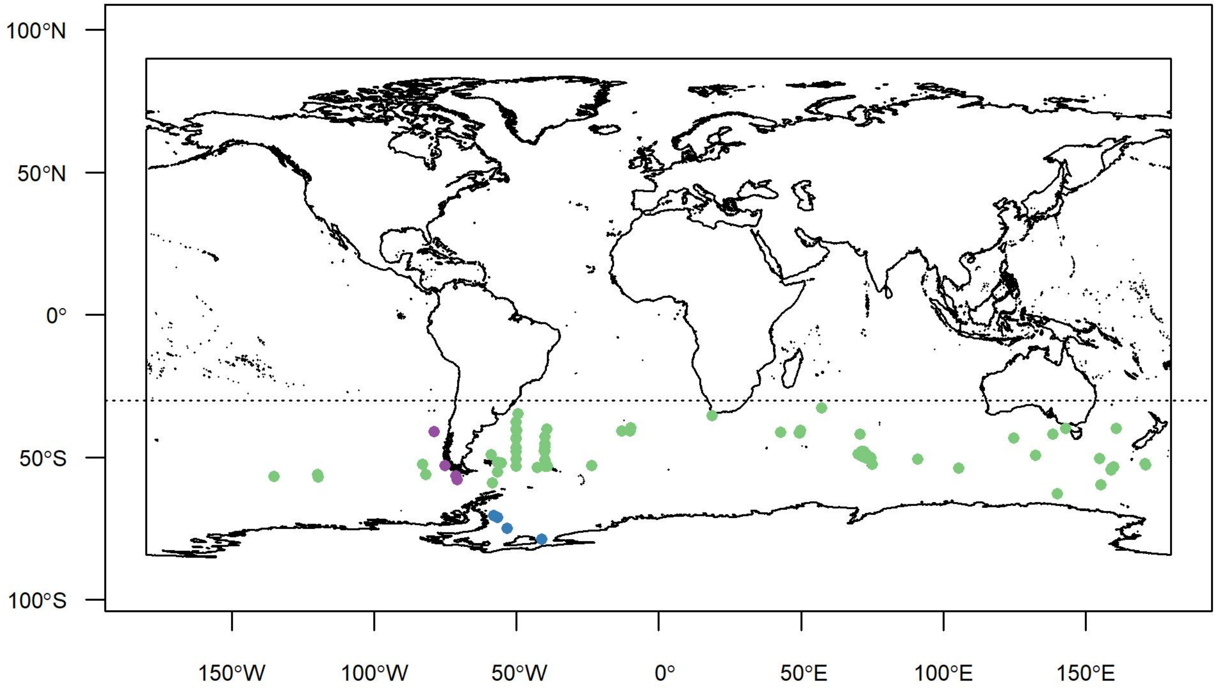

Information about Eltanin records with swapped coordinates are indicated here:

	source	year	references	bibliographicCitation
640551	GBIF	1962	http://portal.vertnet.org/o/lacm/fish?id=839a60e4-b800-45a8-ba71-1b4aa0f292dc	839A60E4-B800-45A8-BA71-1B4AA0F292DC. http://ipt.vertnet.org:8080/ipt/resource.do?r=lacm_verts.
649612	GBIF	1964	http://portal.vertnet.org/o/lacm/fish?id=9188eb06-1f28-49cb-958b-aee6a613d8d2	9188EB06-1F28-49CB-958B-AEE6A613D8D2. http://ipt.vertnet.org:8080/ipt/resource.do?r=lacm_verts.
658271	GBIF	1962	http://portal.vertnet.org/o/lacm/fish?id=9f31e775-5a75-4459-affb-17925d443120	9F31E775-5A75-4459-AFFB-17925D443120. http://ipt.vertnet.org:8080/ipt/resource.do?r=lacm_verts.

	source	year	references	bibliographicCitation
699261	GBIF	1966	http://portal.vertnet.org/o/lacm/fish?id=df6250ef-bd23-460a-826d-72e8fde84dcc	DF6250EF-BD23-460A-826D-72E8FDE84DCC. http://ipt.vertnet.org:8080/ipt/resource.do?r=lacm_verts.

Gonichthys barnesi

Several outliers were identified in tropical areas (red points on map). Pacific outliers probably correspond to species *Gonichthys venetus*, whereas Atlantic outliers probably correspond to species *Gonichthys cocco*.

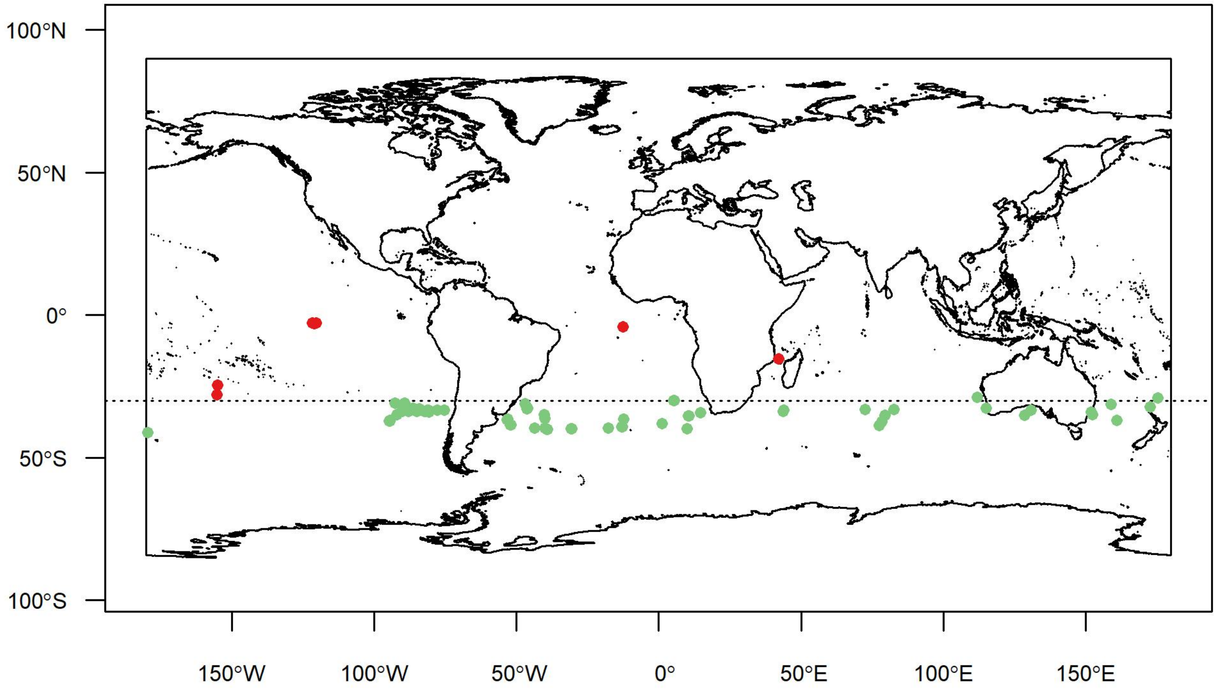

Information about the outliers :

	source	decimalLongitude	decimalLatitude	year	references
30417	OBIS	-12.4050	-4.1578	2009	
248541	GBIF	-155.0050	-24.6450	1969	http://portal.vertnet.org/o/sio/marine-vertebrates?id=c599e473-3c5b-4ed0-8341-89d7c3f9d2d7
278801	GBIF	-121.0000	-3.0333	1970	http://portal.vertnet.org/o/sio/marine-vertebrates?id=5aab2563-9e49-4175-9404-250ffee694cb
287821	GBIF	-155.0300	-27.9733	1971	http://portal.vertnet.org/o/sio/marine-vertebrates?id=f2348698-f14c-4a30-916c-70954c6a3ef9
354412	GBIF	-120.3667	-2.8000	1970	http://portal.vertnet.org/o/sio/marine-vertebrates?id=2a4e14c8-af51-445d-9ad9-36fd623b4b80
643291	GBIF	-121.6000	-2.9500	1970	http://portal.vertnet.org/o/lacm/fish?id=885201cf-a0bd-4e4a-9200-8545ed5ade32
140446	GBIF	42.2500	-15.4500	1988	

Gonichthys cocco

We found two potential outliers in the Indian Ocean (red points on the map), which we removed.

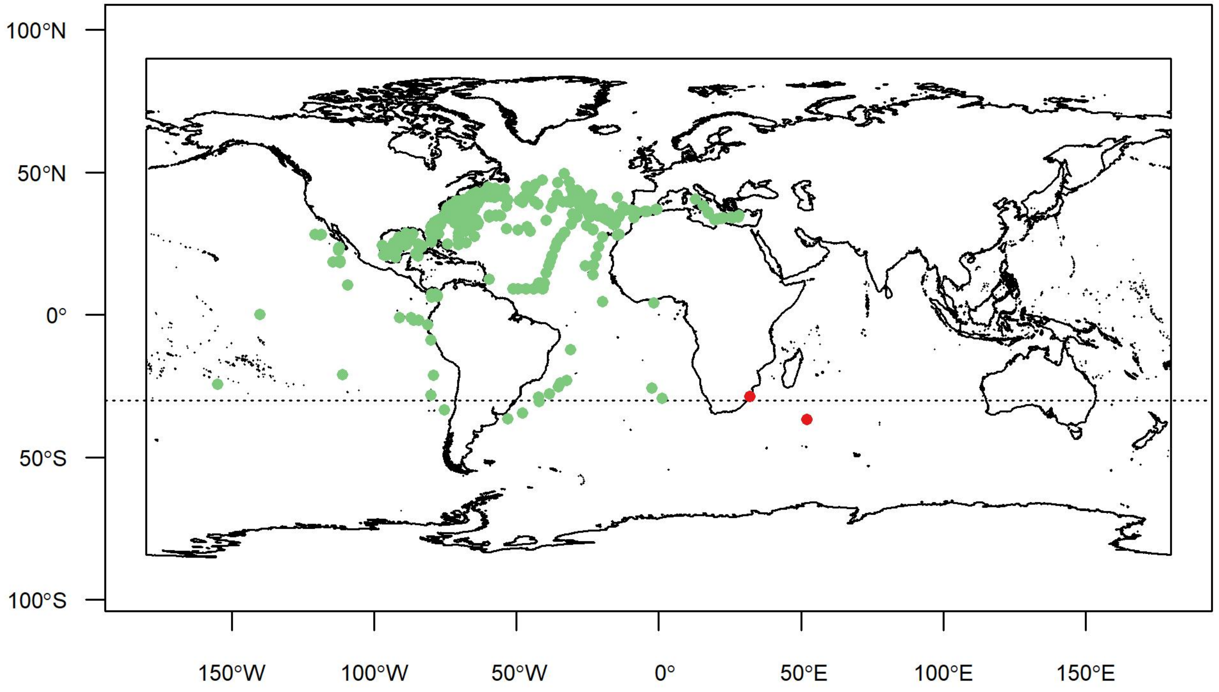

Information about the outliers :

	source	decimalLongitude	decimalLatitude	year	references
70682	OBIS	32.1000	-28.800	1971	
890241	GBIF	52.0555	-36.842	2009	

Gymnoscopelus bolini

We found 4 records with swapped coordinates from the Eltanin GBIF erroneous batch (blue points in map). We corrected their coordinates (purple points in map).

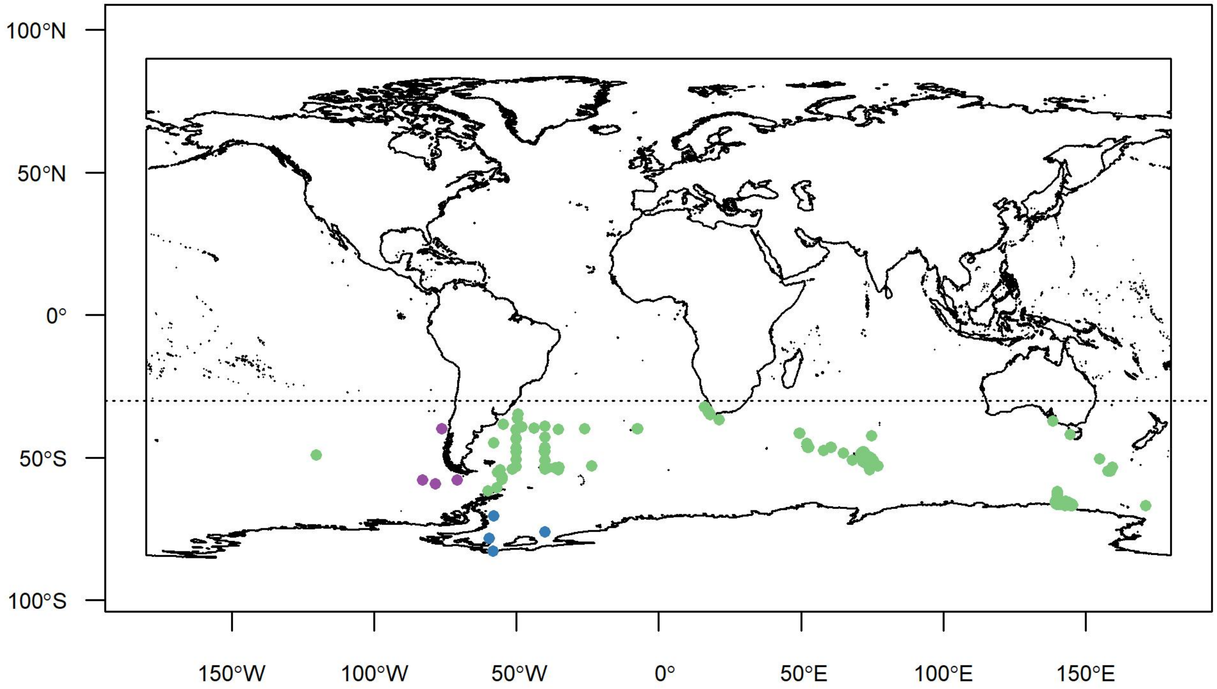

Information about Eltanin records with swapped coordinates are indicated here:

	source	year	references	bibliographicCitation
653761	GBIF	1964	http://portal.vertnet.org/o/lacm/fish?id=98aea7cc-6829-44fd-9029-f52d37f7564a	98AEA7CC-6829-44FD-9029-F52D37F7564A. http://ipt.vertnet.org:8080/ipt/resource.do?r=lacm_verts.

	source	year	references	bibliographicCitation
662641	GBIF	1962	http://portal.vertnet.org/o/lacm/fish?id=a6ac1b1c-5b36-4819-a438-fb0a84316132	A6AC1B1C-5B36-4819-A438-FB0A84316132. http://ipt.vertnet.org:8080/ipt/resource.do?r=lacm_verts.
664661	GBIF	1963	http://portal.vertnet.org/o/lacm/fish?id=a9b5f7a9-cbd4-4491-8281-001e9e059545	A9B5F7A9-CBD4-4491-8281-001E9E059545. http://ipt.vertnet.org:8080/ipt/resource.do?r=lacm_verts.
702951	GBIF	1963	http://portal.vertnet.org/o/lacm/fish?id=e4ec844e-a2cc-4663-a46c-82bef09bd11f	E4EC844E-A2CC-4663-A46C-82BEF09BD11F. http://ipt.vertnet.org:8080/ipt/resource.do?r=lacm_verts.

Gymnoscopelus braueri

We found multiple records with swapped coordinates from the Eltanin GBIF erroneous batch (blue points in map). We corrected their coordinates (purple points in map).

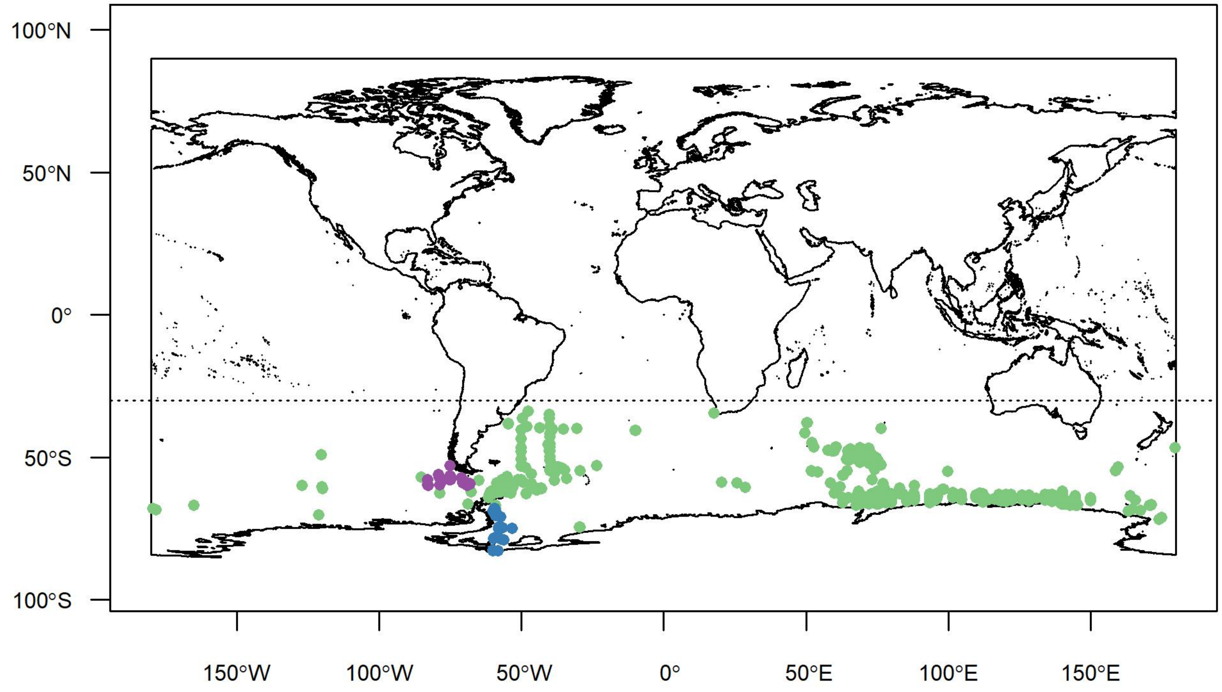

Information about Eltanin records with swapped coordinates are indicated here:

	source	year	references	bibliographicCitation
563021	GBIF	1962	http://portal.vertnet.org/o/lacm/fish?id=0bb3630c-38e5-4d42-86ea-a9418a9ddec7	0BB3630C-38E5-4D42-86EA-A9418A9DDEC7. http://ipt.vertnet.org:8080/ipt/resource.do?r=lacm_verts.
564071	GBIF	1962	http://portal.vertnet.org/o/lacm/fish?id=0d2cb780-d5fd-47d5-a461-59c37b43ab77	0D2CB780-D5FD-47D5-A461-59C37B43AB77. http://ipt.vertnet.org:8080/ipt/resource.do?r=lacm_verts.
568431	GBIF	1963	http://portal.vertnet.org/o/lacm/fish?id=1405fe95-6d7b-4ba6-a672-e7cfbef7f48e	1405FE95-6D7B-4BA6-A672-E7CFBEF7F48E. http://ipt.vertnet.org:8080/ipt/resource.do?r=lacm_verts.

	source	year	references	bibliographicCitation
578671	GBIF	1962	http://portal.vertnet.org/o/lacm/fish?id=2356f516-c697-4dbe-922a-39c3426b59d2	2356F516-C697-4DBE-922A-39C3426B59D2. http://ipt.vertnet.org:8080/ipt/resource.do?r=lacm_verts.
592491	GBIF	1963	http://portal.vertnet.org/o/lacm/fish?id=38ffeff2-2ba5-442a-befe-bcf49faeb6c9	38FFEFF2-2BA5-442A-BEFE-BCF49FAEB6C9. http://ipt.vertnet.org:8080/ipt/resource.do?r=lacm_verts.
604221	GBIF	1963	http://portal.vertnet.org/o/lacm/fish?id=4b3e6ff2-b211-4499-9a9c-0917886bf238	4B3E6FF2-B211-4499-9A9C-0917886BF238. http://ipt.vertnet.org:8080/ipt/resource.do?r=lacm_verts.
606041	GBIF	1963	http://portal.vertnet.org/o/lacm/fish?id=4dcbf4b8-a8e6-4c9a-91b2-e6fd32b9556f	4DCBF4B8-A8E6-4C9A-91B2-E6FD32B9556F. http://ipt.vertnet.org:8080/ipt/resource.do?r=lacm_verts.
608991	GBIF	1962	http://portal.vertnet.org/o/lacm/fish?id=51fc5ff0-8f2f-4fe3-a881-6b882c180f5e	51FC5FF0-8F2F-4FE3-A881-6B882C180F5E. http://ipt.vertnet.org:8080/ipt/resource.do?r=lacm_verts.
613391	GBIF	1963	http://portal.vertnet.org/o/lacm/fish?id=58df7d6f-6865-45f9-9a8c-65c8e88d06a2	58DF7D6F-6865-45F9-9A8C-65C8E88D06A2. http://ipt.vertnet.org:8080/ipt/resource.do?r=lacm_verts.
621971	GBIF	1963	http://portal.vertnet.org/o/lacm/fish?id=6701fdbd-f10d-43eb-88a1-296d66ee3716	6701FDBD-F10D-43EB-88A1-296D66EE3716. http://ipt.vertnet.org:8080/ipt/resource.do?r=lacm_verts.
630112	GBIF	1962	http://portal.vertnet.org/o/lacm/fish?id=73e13845-d1ee-4e7b-a8a2-1f83fea036d8	73E13845-D1EE-4E7B-A8A2-1F83FEA036D8. http://ipt.vertnet.org:8080/ipt/resource.do?r=lacm_verts.
640412	GBIF	1962	http://portal.vertnet.org/o/lacm/fish?id=83d1c5db-62a7-4637-9ecf-8b9572abf228	83D1C5DB-62A7-4637-9ECF-8B9572ABF228. http://ipt.vertnet.org:8080/ipt/resource.do?r=lacm_verts.
659102	GBIF	1964	http://portal.vertnet.org/o/lacm/fish?id=a0d36bf5-a624-432c-8147-5cdc3c5c2942	A0D36BF5-A624-432C-8147-5CDC3C5C2942. http://ipt.vertnet.org:8080/ipt/resource.do?r=lacm_verts.
663701	GBIF	1963	http://portal.vertnet.org/o/lacm/fish?id=a83af5b1-aa3b-48ed-ac0b-d1fc1411386e	A83AF5B1-AA3B-48ED-AC0B-D1FC1411386E. http://ipt.vertnet.org:8080/ipt/resource.do?r=lacm_verts.
677351	GBIF	1963	http://portal.vertnet.org/o/lacm/fish?id=bcc35991-286b-4b92-adcd-038de87934be	BCC35991-286B-4B92-ADCD-038DE87934BE. http://ipt.vertnet.org:8080/ipt/resource.do?r=lacm_verts.
680791	GBIF	1962	http://portal.vertnet.org/o/lacm/fish?id=c255ad56-cbe4-4042-bb32-e449244c3a58	C255AD56-CBE4-4042-BB32-E449244C3A58. http://ipt.vertnet.org:8080/ipt/resource.do?r=lacm_verts.
690841	GBIF	1963	http://portal.vertnet.org/o/lacm/fish?id=d254f62f-2af0-47ae-93c8-6b3ed81efa22	D254F62F-2AF0-47AE-93C8-6B3ED81EFA22. http://ipt.vertnet.org:8080/ipt/resource.do?r=lacm_verts.
707051	GBIF	1963	http://portal.vertnet.org/o/lacm/fish?id=eb3c57c3-a73b-444f-8174-5c361c18ddfc	EB3C57C3-A73B-444F-8174-5C361C18DDFC. http://ipt.vertnet.org:8080/ipt/resource.do?r=lacm_verts.
708551	GBIF	1963	http://portal.vertnet.org/o/lacm/fish?id=ed673ca2-d99e-4430-be3b-72bfa2d2879a	ED673CA2-D99E-4430-BE3B-72BFA2D2879A. http://ipt.vertnet.org:8080/ipt/resource.do?r=lacm_verts.

Gymnoscopelus fraseri

We found multiple records with swapped coordinates from the Eltanin GBIF erroneous batch (blue points in map). We corrected their coordinates (purple points in map).

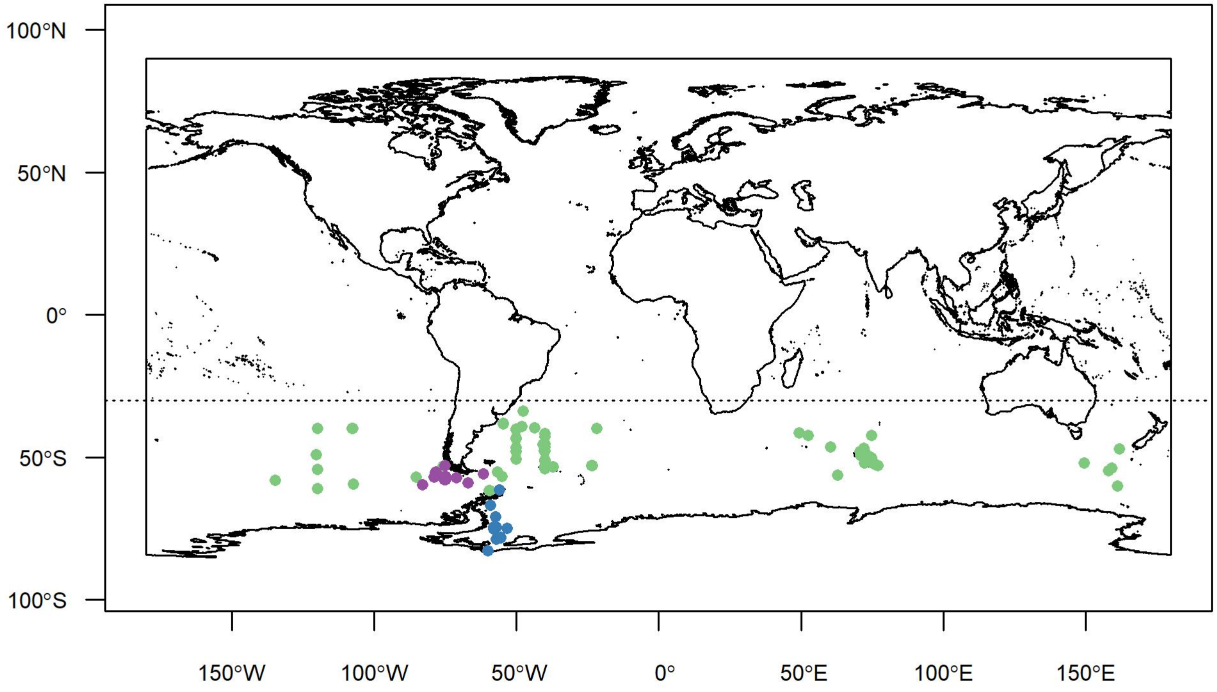

Information about Eltanin records with swapped coordinates are indicated here:

	source	year	references	bibliographicCitation
555771	GBIF	1962	http://portal.vertnet.org/o/lacm/fish?id=00d0a5eb-2527-454f-a67e-e7d1a95f49a6	00D0A5EB-2527-454F-A67E-E7D1A95F49A6. http://ipt.vertnet.org:8080/ipt/resource.do?r=lacm_verts.
557431	GBIF	1963	http://portal.vertnet.org/o/lacm/fish?id=02f81f21-b768-4d8b-b2cf-8b9547d9217a	02F81F21-B768-4D8B-B2CF-8B9547D9217A. http://ipt.vertnet.org:8080/ipt/resource.do?r=lacm_verts.
588821	GBIF	1963	http://portal.vertnet.org/o/lacm/fish?id=33442f1e-3b54-4cc3-8023-a0a503d7b984	33442F1E-3B54-4CC3-8023-A0A503D7B984. http://ipt.vertnet.org:8080/ipt/resource.do?r=lacm_verts.

	source	year	references	bibliographicCitation
600131	GBIF	1963	http://portal.vertnet.org/o/lacm/fish?id=45137b3a-d61f-4c0b-95fc-57b2982f6bf9	45137B3A-D61F-4C0B-95FC-57B2982F6BF9. http://ipt.vertnet.org:8080/ipt/resource.do?r=lacm_verts.
609612	GBIF	1963	http://portal.vertnet.org/o/lacm/fish?id=53339b16-7b2d-42ff-9d9e-b4336a0298f2	53339B16-7B2D-42FF-9D9E-B4336A0298F2. http://ipt.vertnet.org:8080/ipt/resource.do?r=lacm_verts.
634631	GBIF	1963	http://portal.vertnet.org/o/lacm/fish?id=7aed3f60-68ed-4918-b2f1-10e7aeb51ce4	7AED3F60-68ED-4918-B2F1-10E7AEB51CE4. http://ipt.vertnet.org:8080/ipt/resource.do?r=lacm_verts.
663091	GBIF	1964	http://portal.vertnet.org/o/lacm/fish?id=a75f7c3a-a296-455e-a723-6283273e95a7	A75F7C3A-A296-455E-A723-6283273E95A7. http://ipt.vertnet.org:8080/ipt/resource.do?r=lacm_verts.
691261	GBIF	1963	http://portal.vertnet.org/o/lacm/fish?id=d2cf9bee-368a-4541-8438-e2b5552dac73	D2CF9BEE-368A-4541-8438-E2B5552DAC73. http://ipt.vertnet.org:8080/ipt/resource.do?r=lacm_verts.
700961	GBIF	1962	http://portal.vertnet.org/o/lacm/fish?id=e204b177-b877-4f06-a51f-23e97853fef4	E204B177-B877-4F06-A51F-23E97853FEF4. http://ipt.vertnet.org:8080/ipt/resource.do?r=lacm_verts.
701961	GBIF	1963	http://portal.vertnet.org/o/lacm/fish?id=e349371f-6353-4136-be87-ed666e7fb270	E349371F-6353-4136-BE87-ED666E7FB270. http://ipt.vertnet.org:8080/ipt/resource.do?r=lacm_verts.
708871	GBIF	1966	http://portal.vertnet.org/o/lacm/fish?id=ee1c6a5b-4c60-4fba-a9d3-6e3cc9a0f61f	EE1C6A5B-4C60-4FBA-A9D3-6E3CC9A0F61F. http://ipt.vertnet.org:8080/ipt/resource.do?r=lacm_verts.
714361	GBIF	1963	http://portal.vertnet.org/o/lacm/fish?id=f61e9a4e-7210-4b61-a10d-f12e719bf89b	F61E9A4E-7210-4B61-A10D-F12E719BF89B. http://ipt.vertnet.org:8080/ipt/resource.do?r=lacm_verts.

Gymnoscopelus hintonoides

We found no outliers for this species.

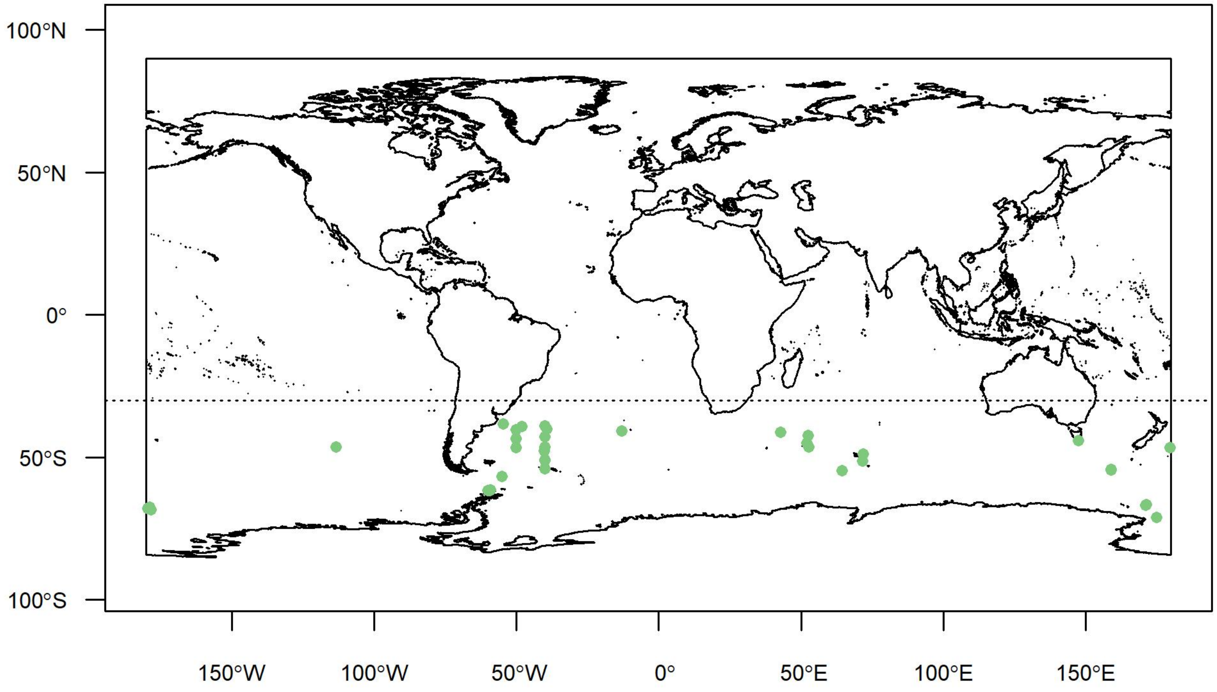

Gymnoscopelus microlampas

We found one outlier in the Mediterranean Sea, which we removed.

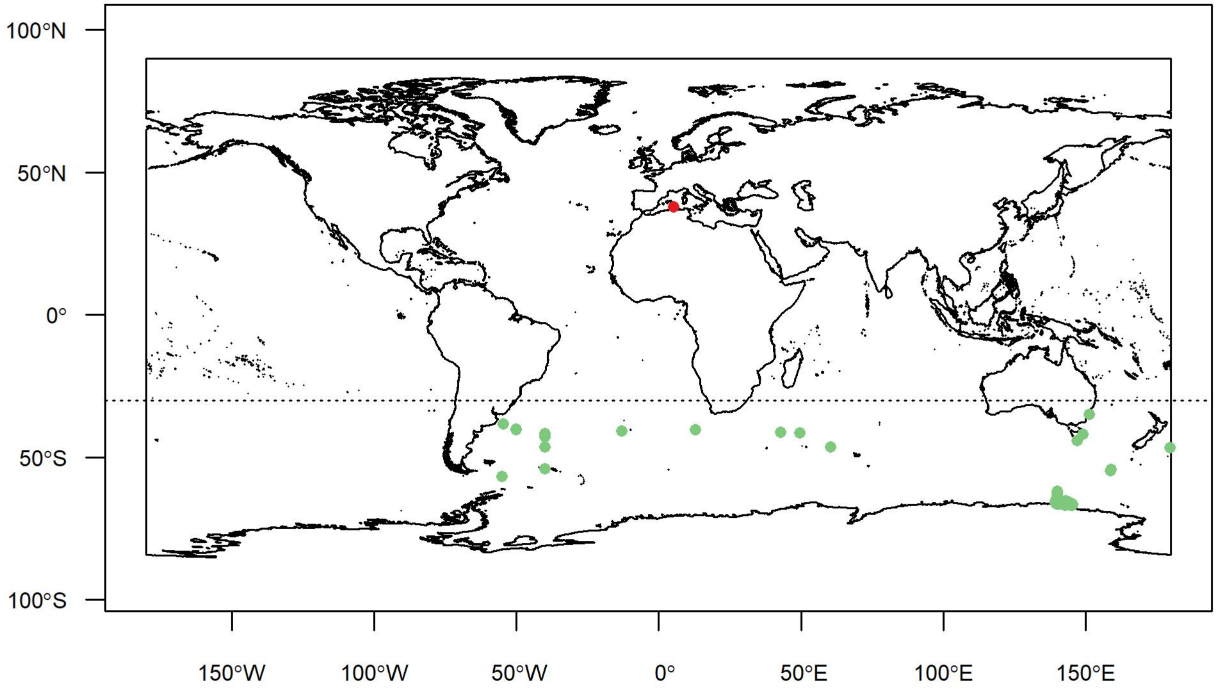

Information about the outlier :

	source	decimalLongitude	decimalLatitude	year	references
32298	OBIS	5.201	37.835	1970	

Gymnoscopelus nicholsi

We found multiple records with swapped coordinates from the Eltanin GBIF erroneous batch (blue points in map). We corrected their coordinates (purple points in map).

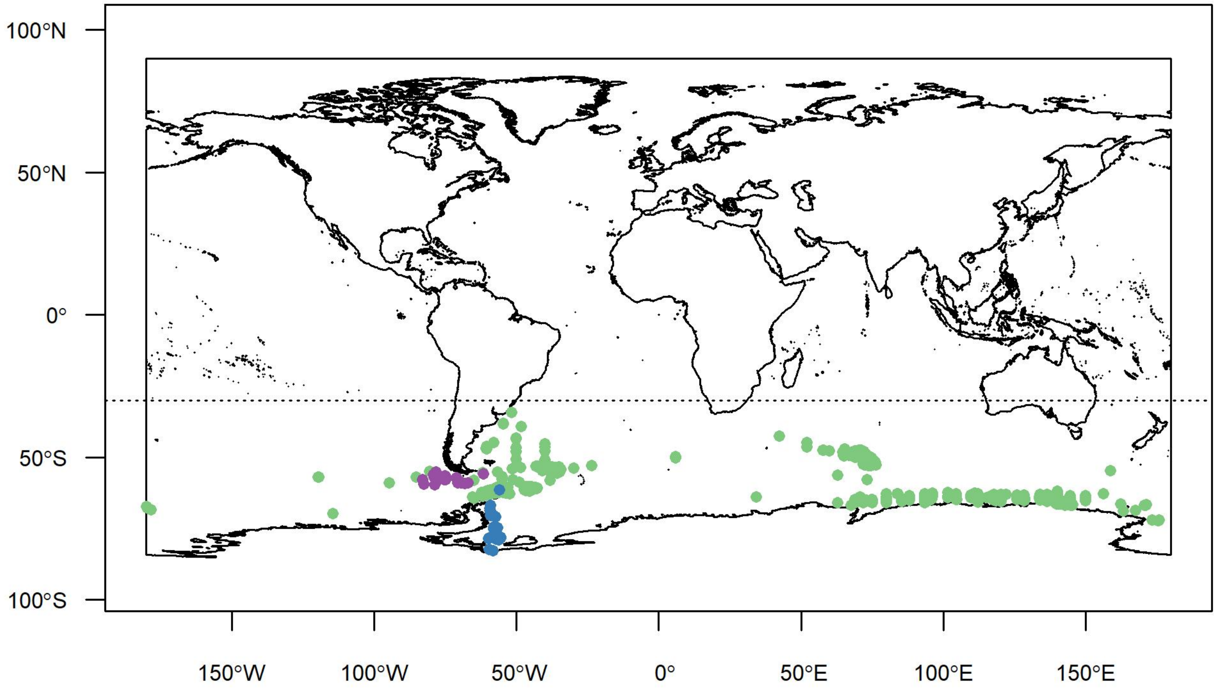

Information about Eltanin records with swapped coordinates are indicated here:

	source	year	references	bibliographicCitation
558102	GBIF	1962	http://portal.vertnet.org/o/lacm/fish?id=0404a055-07a7-4187-9132-0990473d79bc	0404A055-07A7-4187-9132-0990473D79BC. http://ipt.vertnet.org:8080/ipt/resource.do?r=lacm_verts.
569512	GBIF	1962	http://portal.vertnet.org/o/lacm/fish?id=157229f7-0053-4fe1-b35c-4c93c1e0c251	157229F7-0053-4FE1-B35C-4C93C1E0C251. http://ipt.vertnet.org:8080/ipt/resource.do?r=lacm_verts.
573461	GBIF	1963	http://portal.vertnet.org/o/lacm/fish?id=1b4a5263-6eef-4cff-94d0-4934f0e4ef86	1B4A5263-6EEF-4CFF-94D0-4934F0E4EF86. http://ipt.vertnet.org:8080/ipt/resource.do?r=lacm_verts.

	source	year	references	bibliographicCitation
600971	GBIF	1962	http://portal.vertnet.org/o/lacm/fish?id=466f20d5-44a0-4bc8-a83c-128d3dad2183	466F20D5-44A0-4BC8-A83C-128D3DAD2183. http://ipt.vertnet.org:8080/ipt/resource.do?r=lacm_verts.
602741	GBIF	1963	http://portal.vertnet.org/o/lacm/fish?id=49335b90-520e-4881-ba7b-81fded4116db	49335B90-520E-4881-BA7B-81FDED4116DB. http://ipt.vertnet.org:8080/ipt/resource.do?r=lacm_verts.
603301	GBIF	1963	http://portal.vertnet.org/o/lacm/fish?id=4a005464-5128-44c4-8c7c-fb30dc9f16cb	4A005464-5128-44C4-8C7C-FB30DC9F16CB. http://ipt.vertnet.org:8080/ipt/resource.do?r=lacm_verts.
606971	GBIF	1963	http://portal.vertnet.org/o/lacm/fish?id=4f3ffe51-c7ec-4678-bf3d-dc78ff9bbb05	4F3FFE51-C7EC-4678-BF3D-DC78FF9BBB05. http://ipt.vertnet.org:8080/ipt/resource.do?r=lacm_verts.
618821	GBIF	1962	http://portal.vertnet.org/o/lacm/fish?id=61c0dfb5-a29b-4804-9446-9a26b446d0f5	61C0DFB5-A29B-4804-9446-9A26B446D0F5. http://ipt.vertnet.org:8080/ipt/resource.do?r=lacm_verts.
619521	GBIF	1963	http://portal.vertnet.org/o/lacm/fish?id=62d8436b-43ac-43a6-b55d-7aae754c7728	62D8436B-43AC-43A6-B55D-7AAE754C7728. http://ipt.vertnet.org:8080/ipt/resource.do?r=lacm_verts.
622212	GBIF	1963	http://portal.vertnet.org/o/lacm/fish?id=67a37adc-999a-41d1-94cd-6ef587a00404	67A37ADC-999A-41D1-94CD-6EF587A00404. http://ipt.vertnet.org:8080/ipt/resource.do?r=lacm_verts.
623471	GBIF	1963	http://portal.vertnet.org/o/lacm/fish?id=6938ca5c-f923-45c5-aa99-62fabb87b660	6938CA5C-F923-45C5-AA99-62FABB87B660. http://ipt.vertnet.org:8080/ipt/resource.do?r=lacm_verts.
635071	GBIF	1963	http://portal.vertnet.org/o/lacm/fish?id=7b62a94d-ab19-4cbb-a02f-45b28b89c12b	7B62A94D-AB19-4CBB-A02F-45B28B89C12B. http://ipt.vertnet.org:8080/ipt/resource.do?r=lacm_verts.
636551	GBIF	1963	http://portal.vertnet.org/o/lacm/fish?id=7db78099-b188-4d6e-ab23-979fcda2237f	7DB78099-B188-4D6E-AB23-979FCDA2237F. http://ipt.vertnet.org:8080/ipt/resource.do?r=lacm_verts.
643851	GBIF	1966	http://portal.vertnet.org/o/lacm/fish?id=891ebff6-67de-41e9-9c18-7f12be2a113b	891EBFF6-67DE-41E9-9C18-7F12BE2A113B. http://ipt.vertnet.org:8080/ipt/resource.do?r=lacm_verts.
644381	GBIF	1963	http://portal.vertnet.org/o/lacm/fish?id=8a0de0f5-d026-41a0-af33-c81b975899df	8A0DE0F5-D026-41A0-AF33-C81B975899DF. http://ipt.vertnet.org:8080/ipt/resource.do?r=lacm_verts.
644521	GBIF	1962	http://portal.vertnet.org/o/lacm/fish?id=8a1d7823-f014-4f6c-91ac-952086de45c5	8A1D7823-F014-4F6C-91AC-952086DE45C5. http://ipt.vertnet.org:8080/ipt/resource.do?r=lacm_verts.
661131	GBIF	1962	http://portal.vertnet.org/o/lacm/fish?id=a438bd7b-7f4f-4485-855c-21cbfec64810	A438BD7B-7F4F-4485-855C-21CBFEC64810. http://ipt.vertnet.org:8080/ipt/resource.do?r=lacm_verts.
661171	GBIF	1963	http://portal.vertnet.org/o/lacm/fish?id=a4431f44-cf6d-4d64-8eb5-4de859e1360e	A4431F44-CF6D-4D64-8EB5-4DE859E1360E. http://ipt.vertnet.org:8080/ipt/resource.do?r=lacm_verts.
674771	GBIF	1963	http://portal.vertnet.org/o/lacm/fish?id=b8f999c4-11cf-45bc-b4e0-f11f43c7084a	B8F999C4-11CF-45BC-B4E0-F11F43C7084A. http://ipt.vertnet.org:8080/ipt/resource.do?r=lacm_verts.
680581	GBIF	1962	http://portal.vertnet.org/o/lacm/fish?id=c230ecb6-97ca-47d8-b855-d8625de11450	C230ECB6-97CA-47D8-B855-D8625DE11450. http://ipt.vertnet.org:8080/ipt/resource.do?r=lacm_verts.
707081	GBIF	1963	http://portal.vertnet.org/o/lacm/fish?id=eb556126-3ccf-4c84-ad14-8bb08a708be8	EB556126-3CCF-4C84-AD14-8BB08A708BE8. http://ipt.vertnet.org:8080/ipt/resource.do?r=lacm_verts.
717081	GBIF	1962	http://portal.vertnet.org/o/lacm/fish?id=fa2dfb8c-b314-42c7-8b4a-2321e2e41747	FA2DFB8C-B314-42C7-8B4A-2321E2E41747. http://ipt.vertnet.org:8080/ipt/resource.do?r=lacm_verts.

Gymnoscopelus opisthopterus

We found multiple records with swapped coordinates from the Eltanin GBIF erroneous batch (blue points in map). We corrected their coordinates (purple points in map).

We also found two outliers in the Indian Ocean (red points in map), which we could not verify, therefore we removed them.

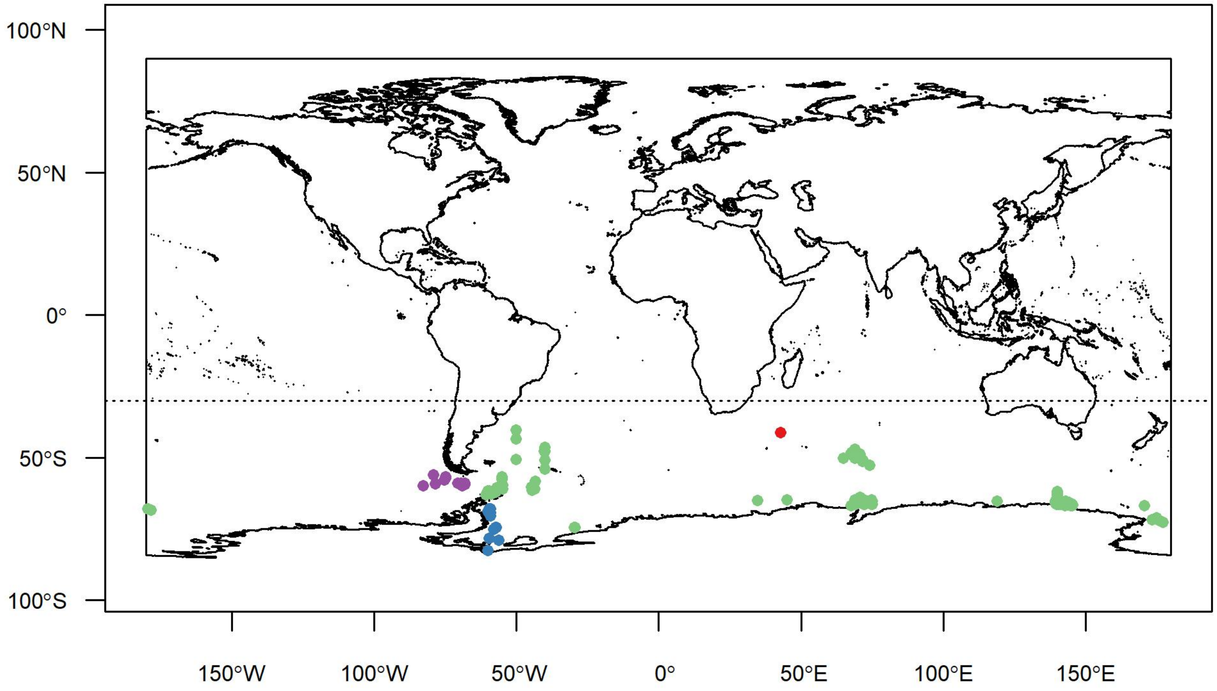

Information about Eltanin records with swapped coordinates are indicated here:

	source	year	references	bibliographicCitation
559531	GBIF	1962	http://portal.vertnet.org/o/lacm/fish?id=06190736-765e-4de2-8999-114484df6419	06190736-765E-4DE2-8999-114484DF6419. http://ipt.vertnet.org:8080/ipt/resource.do?r=lacm_verts.
561271	GBIF	1962	http://portal.vertnet.org/o/lacm/fish?id=090e9323-36b1-49b8-b6a5-2e9bb48c3956	090E9323-36B1-49B8-B6A5-2E9BB48C3956. http://ipt.vertnet.org:8080/ipt/resource.do?r=lacm_verts.
586641	GBIF	1962	http://portal.vertnet.org/o/lacm/fish?id=2ff4fc86-5d71-4c3e-90ab-f39fc9d758b4	2FF4FC86-5D71-4C3E-90AB-F39FC9D758B4. http://ipt.vertnet.org:8080/ipt/resource.do?r=lacm_verts.
595761	GBIF	1963	http://portal.vertnet.org/o/lacm/fish?id=3ead8d8a-1fd5-4df3-8dc4-16baff630fa6	3EAD8D8A-1FD5-4DF3-8DC4-16BAFF630FA6. http://ipt.vertnet.org:8080/ipt/resource.do?r=lacm_verts.
603431	GBIF	1963	http://portal.vertnet.org/o/lacm/fish?id=4a20d806-f7fa-4913-a10a-b4166b33b6d4	4A20D806-F7FA-4913-A10A-B4166B33B6D4. http://ipt.vertnet.org:8080/ipt/resource.do?r=lacm_verts.
621351	GBIF	1963	http://portal.vertnet.org/o/lacm/fish?id=65ffe5cb-8c3a-4d13-95d5-575c5a802b69	65FFE5CB-8C3A-4D13-95D5-575C5A802B69. http://ipt.vertnet.org:8080/ipt/resource.do?r=lacm_verts.
631661	GBIF	1962	http://portal.vertnet.org/o/lacm/fish?id=76006de4-4680-408b-9ed9-8705e807bbda	76006DE4-4680-408B-9ED9-8705E807BBDA. http://ipt.vertnet.org:8080/ipt/resource.do?r=lacm_verts.
635791	GBIF	1963	http://portal.vertnet.org/o/lacm/fish?id=7c85f99a-a1ef-435b-99ec-a084caf421de	7C85F99A-A1EF-435B-99EC-A084CAF421DE. http://ipt.vertnet.org:8080/ipt/resource.do?r=lacm_verts.
655821	GBIF	1963	http://portal.vertnet.org/o/lacm/fish?id=9b4c476f-2336-412f-a757-2ab5d4a8ef6b	9B4C476F-2336-412F-A757-2AB5D4A8EF6B. http://ipt.vertnet.org:8080/ipt/resource.do?r=lacm_verts.
708401	GBIF	1963	http://portal.vertnet.org/o/lacm/fish?id=ed2b80c9-2761-408c-9883-36210812502a	ED2B80C9-2761-408C-9883-36210812502A. http://ipt.vertnet.org:8080/ipt/resource.do?r=lacm_verts.
718481	GBIF	1962	http://portal.vertnet.org/o/lacm/fish?id=fc844fbb-ffb0-4f0c-a553-910329945798	FC844FBB-FFB0-4F0C-A553-910329945798. http://ipt.vertnet.org:8080/ipt/resource.do?r=lacm_verts.

Information about the outliers :

	source	decimalLongitude	decimalLatitude	year	references
892141	GBIF	42.9303	-41.4262	2009	
893012	GBIF	42.9031	-41.4193	2009	

Gymnoscopelus piabilis

We found multiple records with swapped coordinates from the Eltanin GBIF erroneous batch (blue points in map). We corrected their coordinates (purple points in map).

We also found outliers close to the Antarctic continent (red points in map). We removed all records coming from Soviet Antarctic expeditions, because, after a careful verification, we concluded the identified species could not be *Gymnoscopelus piabilis*. We also removed outlier records from the RV Tangaroa that we could not verify.

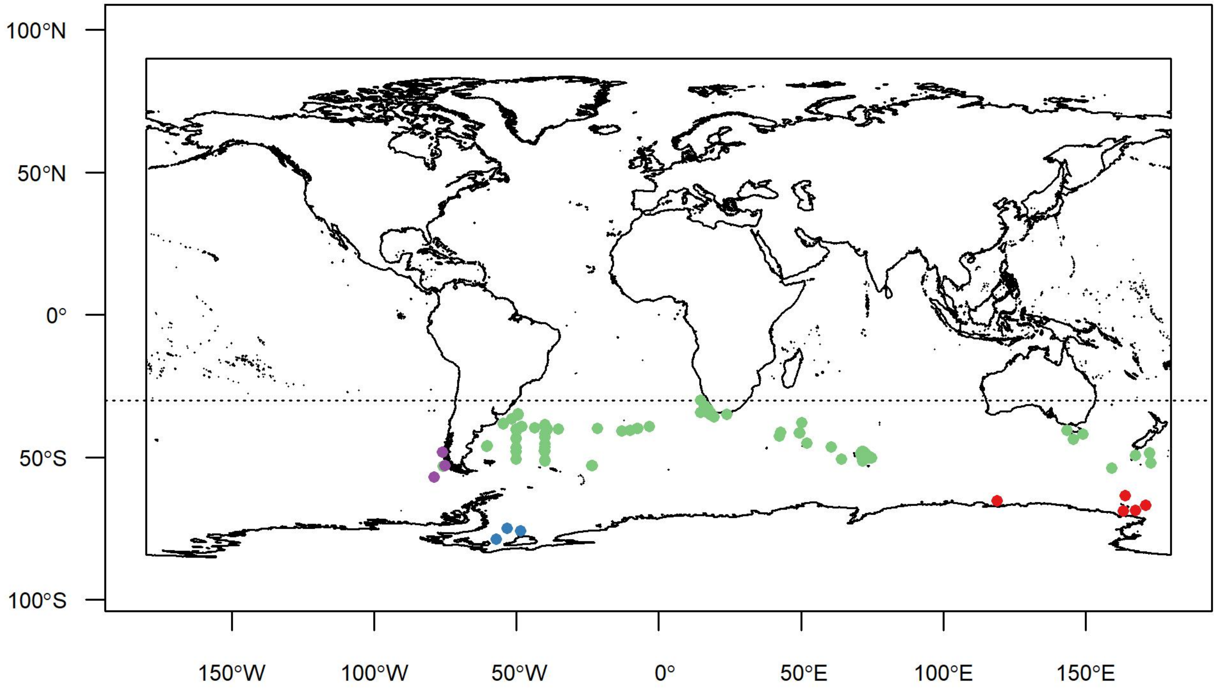

Information about Eltanin records with swapped coordinates are indicated here:

	source	year	references	bibliographicCitation
573971	GBIF	1963	http://portal.vertnet.org/o/lacm/fish?id=1c0f4eb4-6699-4b4b-b1c3-203219c612b5	1C0F4EB4-6699-4B4B-B1C3-203219C612B5. http://ipt.vertnet.org:8080/ipt/resource.do?r=lacm_verts.
592912	GBIF	1964	http://portal.vertnet.org/o/lacm/fish?id=39b19004-9e23-43c4-bd7a-ab5b97ca769e	39B19004-9E23-43C4-BD7A-AB5B97CA769E. http://ipt.vertnet.org:8080/ipt/resource.do?r=lacm_verts.
675861	GBIF	1962	http://portal.vertnet.org/o/lacm/fish?id=ba844ee7-26b4-4595-aece-7bf95ceb3d81	BA844EE7-26B4-4595-AECE-7BF95CEB3D81. http://ipt.vertnet.org:8080/ipt/resource.do?r=lacm_verts.

Information about the outliers :

	source	decimalLongitude	decimalLatitude	year	references
53439	OBIS	163.2000	-68.9167	1960	
63616	OBIS	167.5000	-68.6667	1960	
76225	OBIS	118.8333	-65.3000	1959	
442761	GBIF	171.0700	-66.9500	2008	http://www.ebi.ac.uk/ena/data/view/JN640974
809671	GBIF	163.8667	-63.5000	1960	
146569	GBIF	171.0750	-66.9503	2008	

Hintonia candens

We found two records with swapped coordinates from the Eltanin GBIF erroneous batch (blue points in map). We corrected their coordinates (purple points in map).

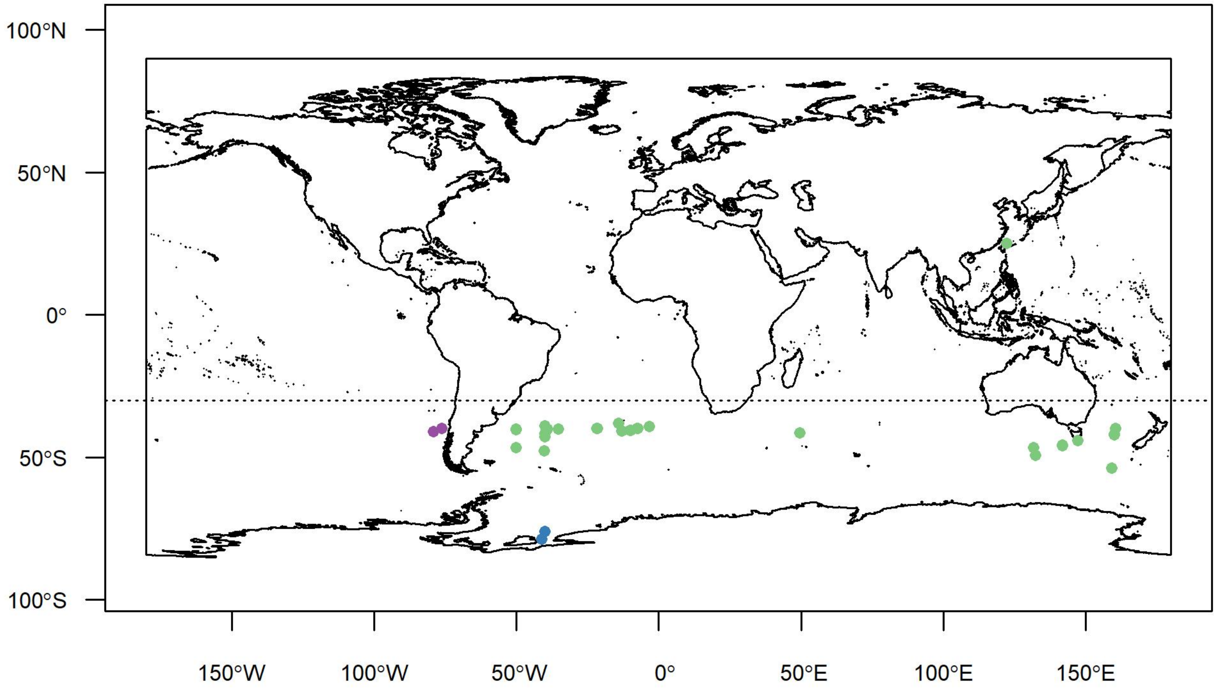

Information about Eltanin records with swapped coordinates are indicated here:

	source	year	references	bibliographicCitation
622141	GBIF	1964	http://portal.vertnet.org/o/lacm/fish?id=67901c3e-badc-4ae6-a98f-6f1ec708a04b	67901C3E-BADC-4AE6-A98F-6F1EC708A04B. http://ipt.vertnet.org:8080/ipt/resource.do?r=lacm_verts.
668741	GBIF	1966	http://portal.vertnet.org/o/lacm/fish?id=aff94ee1-b44f-4ec4-aa70-f6702bcd351f	AFF94EE1-B44F-4EC4-AA70-F6702BCD351F. http://ipt.vertnet.org:8080/ipt/resource.do?r=lacm_verts.

Hygophum bruuni

We found multiple records with swapped coordinates from the Eltanin GBIF erroneous batch (blue points in map). We corrected their coordinates (purple points in map).

We also found one potential outlier near New Zealand (red point on the map), which we removed.

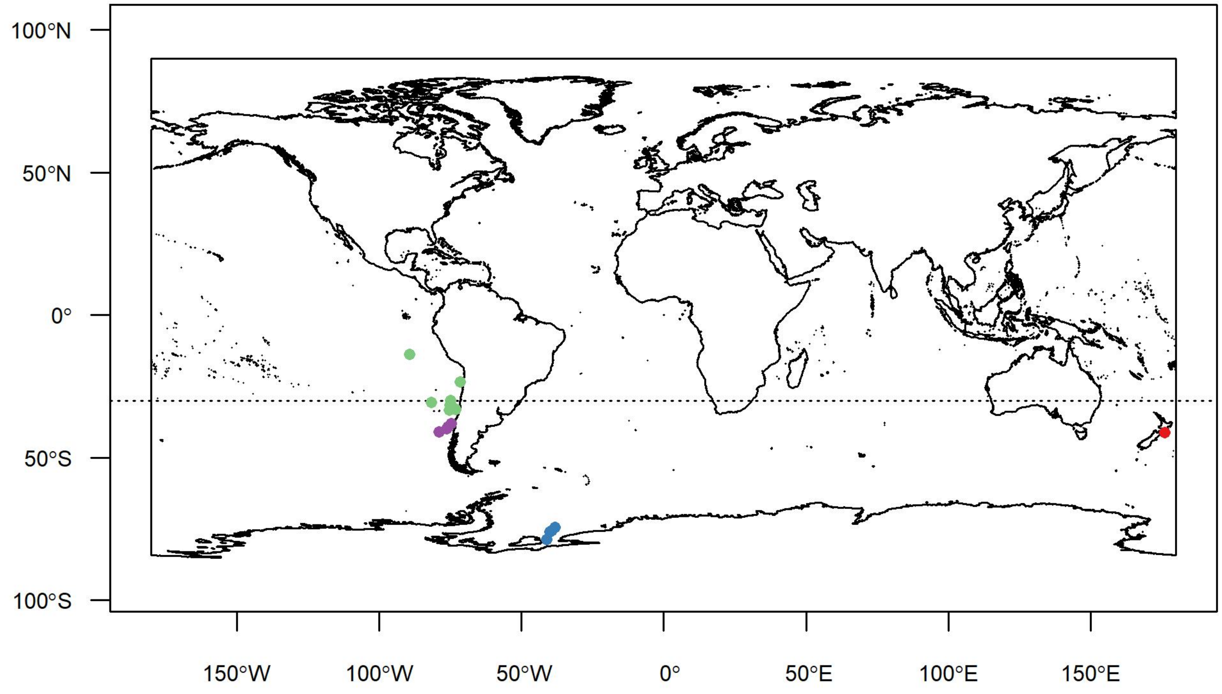

Information about Eltanin records with swapped coordinates are indicated here:

	source	year	references	bibliographicCitation
565121	GBIF	1966	http://portal.vertnet.org/o/lacm/fish?id=0f0c0124-310e-4b2f-959c-74fcbe1474e9	0F0C0124-310E-4B2F-959C-74FCBE1474E9. http://ipt.vertnet.org:8080/ipt/resource.do?r=lacm_verts.
591971	GBIF	1964	http://portal.vertnet.org/o/lacm/fish?id=383501b7-cc05-47b6-842b-d49394d0a4dc	383501B7-CC05-47B6-842B-D49394D0A4DC. http://ipt.vertnet.org:8080/ipt/resource.do?r=lacm_verts.
635561	GBIF	1964	http://portal.vertnet.org/o/lacm/fish?id=7c8fb2ff-dc9d-49ee-a642-9ce6aac4f478	7C8FB2FF-DC9D-49EE-A642-9CE6AAC4F478. http://ipt.vertnet.org:8080/ipt/resource.do?r=lacm_verts.
656312	GBIF	1962	http://portal.vertnet.org/o/lacm/fish?id=9c46a3bd-a717-448b-b8c3-8d6d0776d8ae	9C46A3BD-A717-448B-B8C3-8D6D0776D8AE. http://ipt.vertnet.org:8080/ipt/resource.do?r=lacm_verts.

Information about the outlier :

	source	decimalLongitude	decimalLatitude	year	references
287971	GBIF	176.0583	-41.43	1971	http://portal.vertnet.org/o/sio/marine-vertebrates?id=2b62bc1f-1459-4672-8855-01ef189f4ada

Hygophum hanseni

We found one record with swapped coordinates from the Eltanin GBIF erroneous batch (blue points in map), which we corrected (purple points in map).

We found one outlier in the Northern Hemisphere (red point in map) which we removed.

We also found one outlier close to the Antarctic continent (orange point in map), which we could not verify because of the lack of information, therefore we removed it.

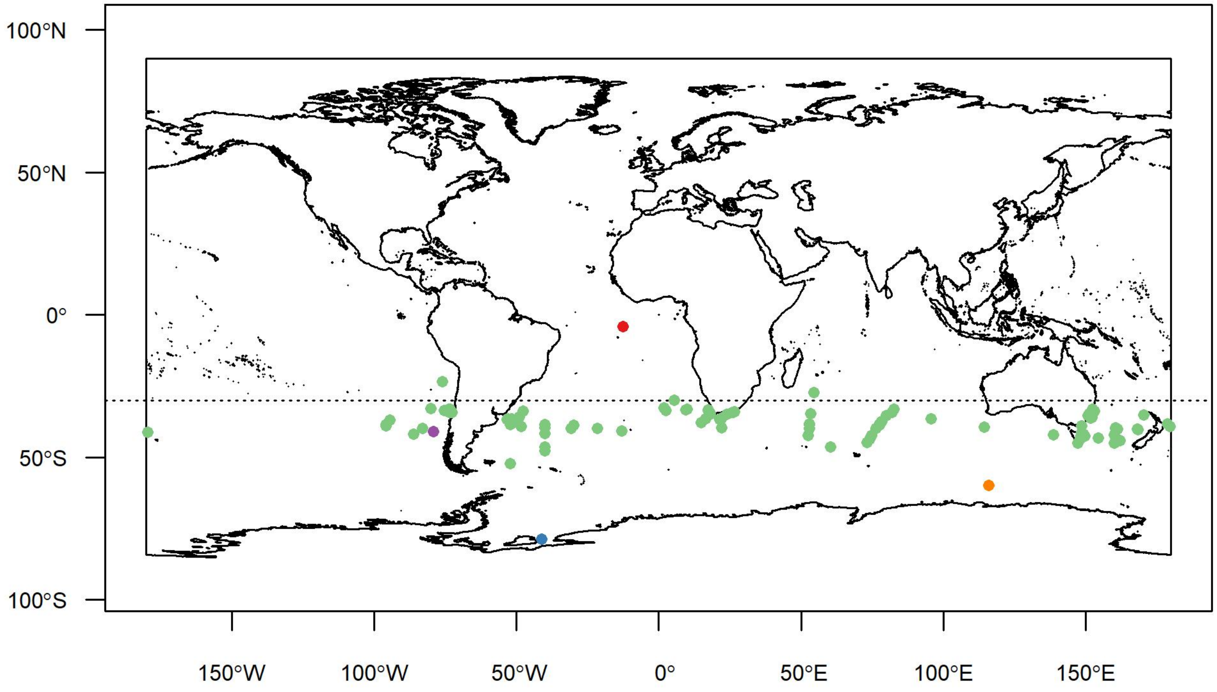

Information about Eltanin records with swapped coordinates are indicated here:

	source	year	references	bibliographicCitation
619981	GBIF	1966	http://portal.vertnet.org/o/lacm/fish?id=64166162-f329-48c4-a913-6f37222e0d11	64166162-F329-48C4-A913-6F37222E0D11. http://ipt.vertnet.org:8080/ipt/resource.do?r=lacm_verts.

Information about the Northern Hemisphere outlier :

	source	decimalLongitude	decimalLatitude	year	references
68828	OBIS	-12.405	-4.1578	2009	

Information about the Antarctic outlier :

	source	decimalLongitude	decimalLatitude	year	references
576311	GBIF	116.0003	-60.0003	1984	

Hygophum hygomii

We found no outliers for this species.

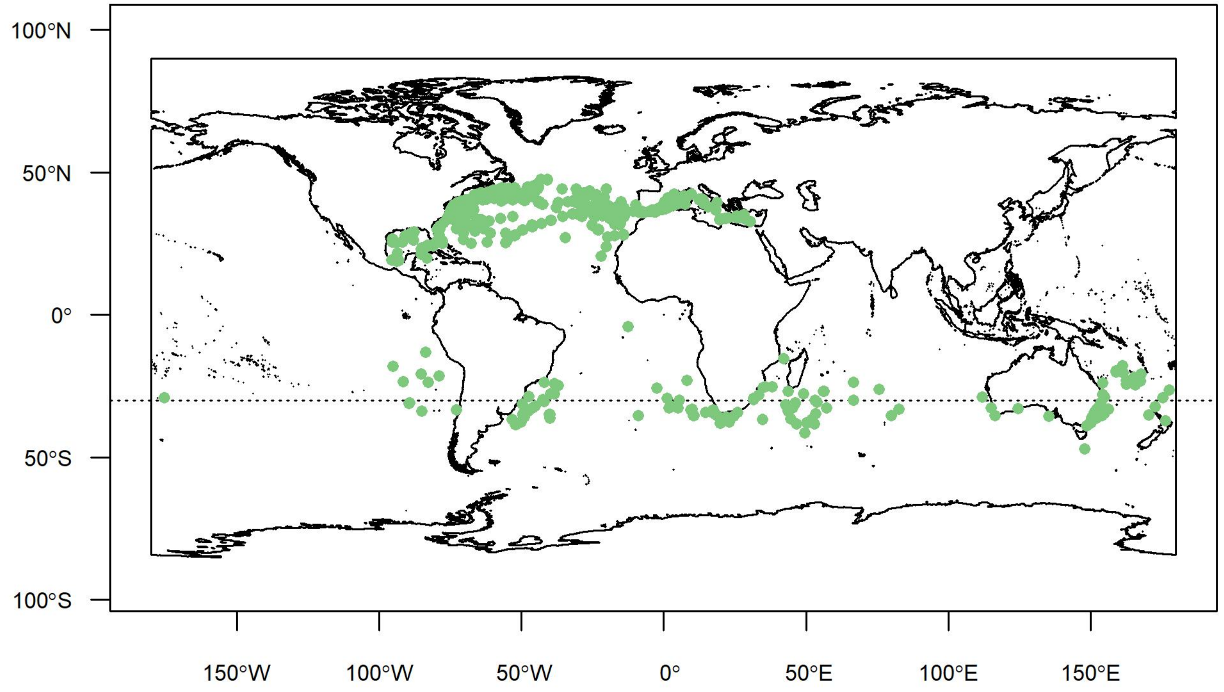

Hygophum macrochir

This species is often subject to misidentification, so we decided to remove it from our dataset.

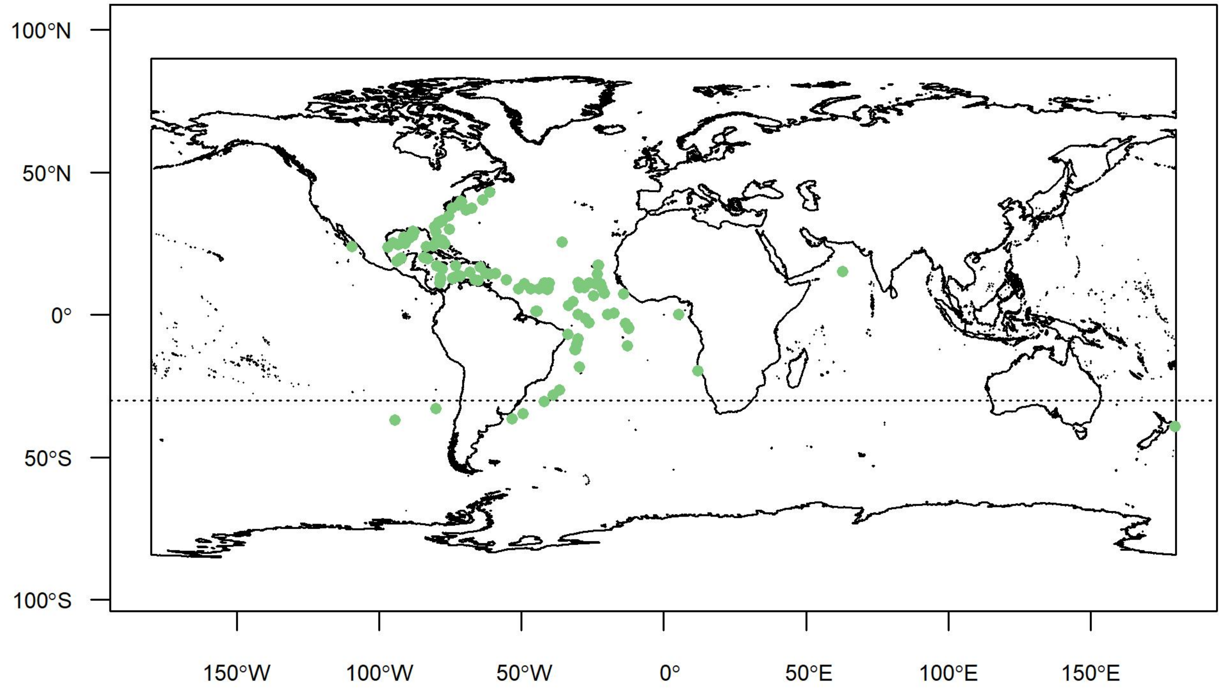

Hygophum proximum

We found one outlier in the Atlantic Ocean (red point on the map), which we removed.

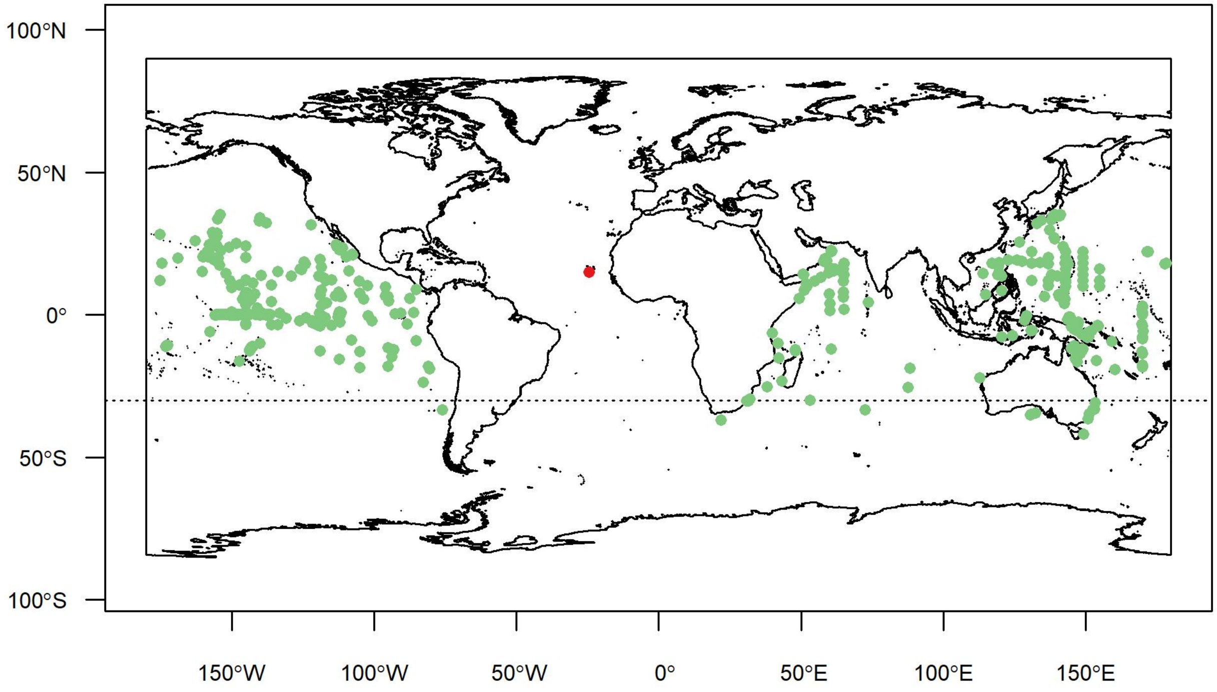

Information about the outlier :

	source	decimalLongitude	decimalLatitude	year	references
173186	GBIF	-24.36	14.7967	2011	

Hygophum reinhardtii

We found no outliers for this species.

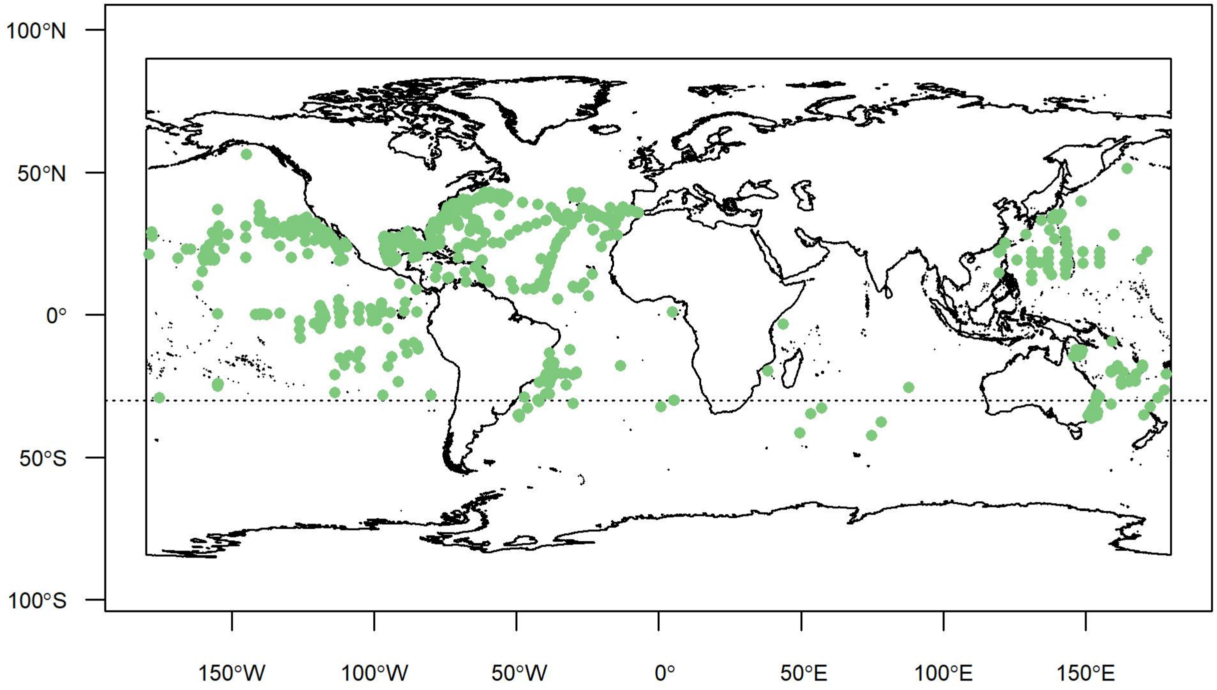

Krefftichthys anderssoni

We found multiple records with swapped coordinates from the Eltanin GBIF erroneous batch (blue points in map). We corrected their coordinates (purple points in map).

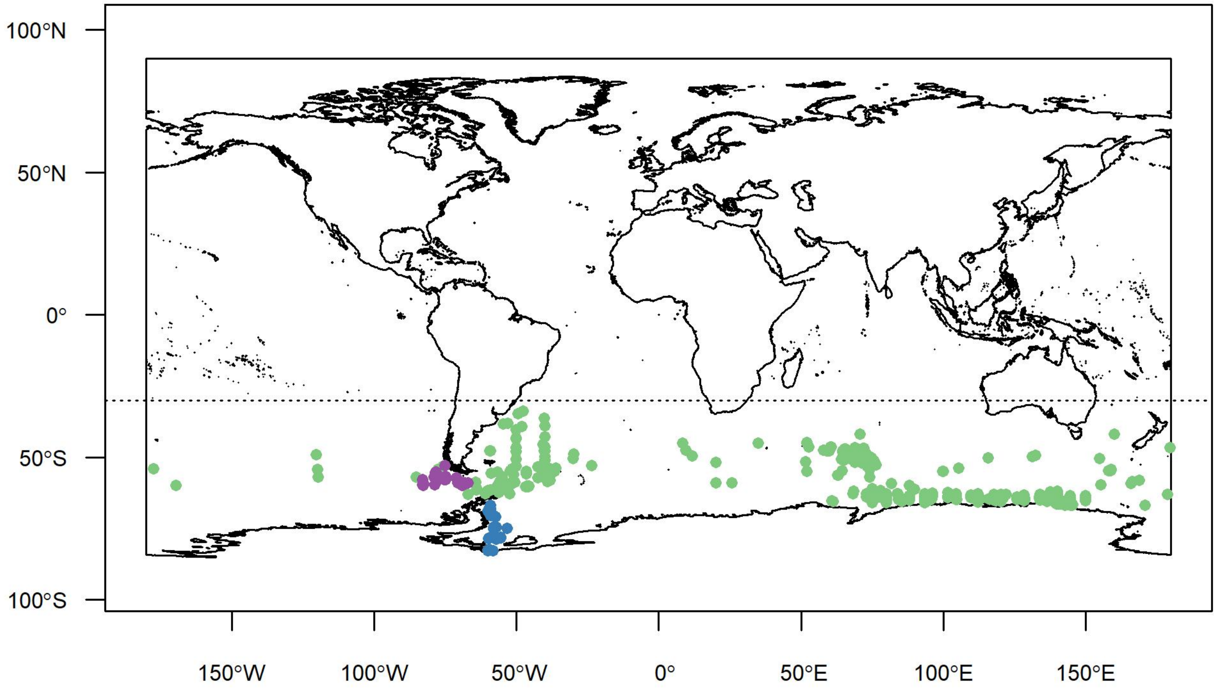

Information about Eltanin records with swapped coordinates are indicated here:

	source	year	references	bibliographicCitation
562141	GBIF	1963	http://portal.vertnet.org/o/lacm/fish?id=0a2b40fd-b543-4dcb-9760-616d5e8fdcc0	0A2B40FD-B543-4DCB-9760-616D5E8FDCC0. http://ipt.vertnet.org:8080/ipt/resource.do?r=lacm_verts.
580671	GBIF	1963	http://portal.vertnet.org/o/lacm/fish?id=26785cb2-fa91-4a82-b131-5fbfd85dcd90	26785CB2-FA91-4A82-B131-5FBFD85DCD90. http://ipt.vertnet.org:8080/ipt/resource.do?r=lacm_verts.
604412	GBIF	1962	http://portal.vertnet.org/o/lacm/fish?id=4b665514-4b5f-4b49-aac8-542cec58a472	4B665514-4B5F-4B49-AAC8-542CEC58A472. http://ipt.vertnet.org:8080/ipt/resource.do?r=lacm_verts.

	source	year	references	bibliographicCitation
604801	GBIF	1963	http://portal.vertnet.org/o/lacm/fish?id=4bdfc60a-4d63-4485-ba43-0cc31e53b0ca	4BDFC60A-4D63-4485-BA43-0CC31E53B0CA. http://ipt.vertnet.org:8080/ipt/resource.do?r=lacm_verts.
605641	GBIF	1963	http://portal.vertnet.org/o/lacm/fish?id=4d375341-5084-4198-8fd0-1926d2280c44	4D375341-5084-4198-8FD0-1926D2280C44. http://ipt.vertnet.org:8080/ipt/resource.do?r=lacm_verts.
613901	GBIF	1963	http://portal.vertnet.org/o/lacm/fish?id=59b743ad-e4bf-4609-9de7-c3e4864019ce	59B743AD-E4BF-4609-9DE7-C3E4864019CE. http://ipt.vertnet.org:8080/ipt/resource.do?r=lacm_verts.
620241	GBIF	1962	http://portal.vertnet.org/o/lacm/fish?id=645cf358-c81d-41e2-b9ae-fcf5d3fb5680	645CF358-C81D-41E2-B9AE-FCF5D3FB5680. http://ipt.vertnet.org:8080/ipt/resource.do?r=lacm_verts.
643691	GBIF	1962	http://portal.vertnet.org/o/lacm/fish?id=88e4d97c-59cc-473d-9c4b-2862cabcbf6c	88E4D97C-59CC-473D-9C4B-2862CABCBF6C. http://ipt.vertnet.org:8080/ipt/resource.do?r=lacm_verts.
645001	GBIF	1962	http://portal.vertnet.org/o/lacm/fish?id=8a4eeaa6-4b94-45d2-aeec-f1d52258af67	8A4EEAA6-4B94-45D2-AEEC-F1D52258AF67. http://ipt.vertnet.org:8080/ipt/resource.do?r=lacm_verts.
654351	GBIF	1963	http://portal.vertnet.org/o/lacm/fish?id=999ce1ae-98d8-4a11-870f-3b8a5eac2b0b	999CE1AE-98D8-4A11-870F-3B8A5EAC2B0B. http://ipt.vertnet.org:8080/ipt/resource.do?r=lacm_verts.
657691	GBIF	1963	http://portal.vertnet.org/o/lacm/fish?id=9e646c5c-fff7-4918-92aa-7de191b2d04c	9E646C5C-FFF7-4918-92AA-7DE191B2D04C. http://ipt.vertnet.org:8080/ipt/resource.do?r=lacm_verts.
662581	GBIF	1963	http://portal.vertnet.org/o/lacm/fish?id=a610547e-003f-42a0-b78d-1c6a00d64399	A610547E-003F-42A0-B78D-1C6A00D64399. http://ipt.vertnet.org:8080/ipt/resource.do?r=lacm_verts.
662901	GBIF	1962	http://portal.vertnet.org/o/lacm/fish?id=a71b8955-c864-43a3-bcb6-6efe82fe9bda	A71B8955-C864-43A3-BCB6-6EFE82FE9BDA. http://ipt.vertnet.org:8080/ipt/resource.do?r=lacm_verts.
672521	GBIF	1962	http://portal.vertnet.org/o/lacm/fish?id=b5c90552-19b0-4374-9b48-cd8681bd28a6	B5C90552-19B0-4374-9B48-CD8681BD28A6. http://ipt.vertnet.org:8080/ipt/resource.do?r=lacm_verts.
682721	GBIF	1962	http://portal.vertnet.org/o/lacm/fish?id=c549bd20-8029-49c7-a45d-edc975c5cc8d	C549BD20-8029-49C7-A45D-EDC975C5CC8D. http://ipt.vertnet.org:8080/ipt/resource.do?r=lacm_verts.
685071	GBIF	1962	http://portal.vertnet.org/o/lacm/fish?id=c93d4dc2-ade3-419d-a181-32d60ec49702	C93D4DC2-ADE3-419D-A181-32D60EC49702. http://ipt.vertnet.org:8080/ipt/resource.do?r=lacm_verts.
686301	GBIF	1963	http://portal.vertnet.org/o/lacm/fish?id=cb961192-b1ba-4c89-ac34-dd47b2aa1def	CB961192-B1BA-4C89-AC34-DD47B2AA1DEF. http://ipt.vertnet.org:8080/ipt/resource.do?r=lacm_verts.
695051	GBIF	1963	http://portal.vertnet.org/o/lacm/fish?id=d8ea6c8f-7328-4f82-82e7-10674bcdbbcc	D8EA6C8F-7328-4F82-82E7-10674BCDBBCC. http://ipt.vertnet.org:8080/ipt/resource.do?r=lacm_verts.
698051	GBIF	1963	http://portal.vertnet.org/o/lacm/fish?id=dda43d47-8c3f-4939-b014-648792ef6c44	DDA43D47-8C3F-4939-B014-648792EF6C44. http://ipt.vertnet.org:8080/ipt/resource.do?r=lacm_verts.
708801	GBIF	1964	http://portal.vertnet.org/o/lacm/fish?id=ede20c2a-7650-48d3-8027-3d671c149f9c	EDE20C2A-7650-48D3-8027-3D671C149F9C. http://ipt.vertnet.org:8080/ipt/resource.do?r=lacm_verts.
712501	GBIF	1963	http://portal.vertnet.org/o/lacm/fish?id=f365fb1d-22b2-4f91-ad7f-f9c0fe298ba0	F365FB1D-22B2-4F91-AD7F-F9C0FE298BA0. http://ipt.vertnet.org:8080/ipt/resource.do?r=lacm_verts.
716721	GBIF	1963	http://portal.vertnet.org/o/lacm/fish?id=f9c7d30f-77a0-4766-90ed-ec6bdcdaa8c6	F9C7D30F-77A0-4766-90ED-EC6BDCDAA8C6. http://ipt.vertnet.org:8080/ipt/resource.do?r=lacm_verts.

719691	GBIF	1962	http://portal.vertnet.org/o/lacm/fish?id=fe65fa5a-eae2-49d4-9c69-99cb6a1643af	FE65FA5A-EAE2-49D4-9C69-99CB6A1643AF. http://ipt.vertnet.org:8080/ipt/resource.do?r=lacm_verts.

Lampadena chavesi

We found no outliers for this species.

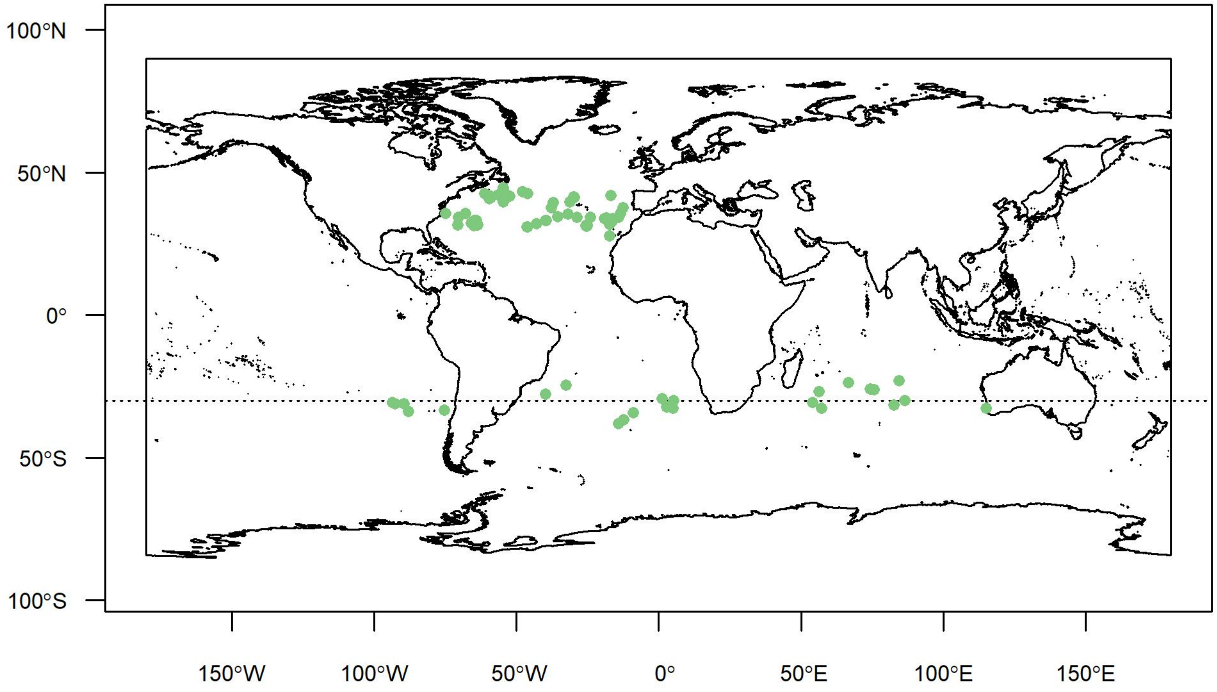

Lampadena dea

We found no outliers for this species.

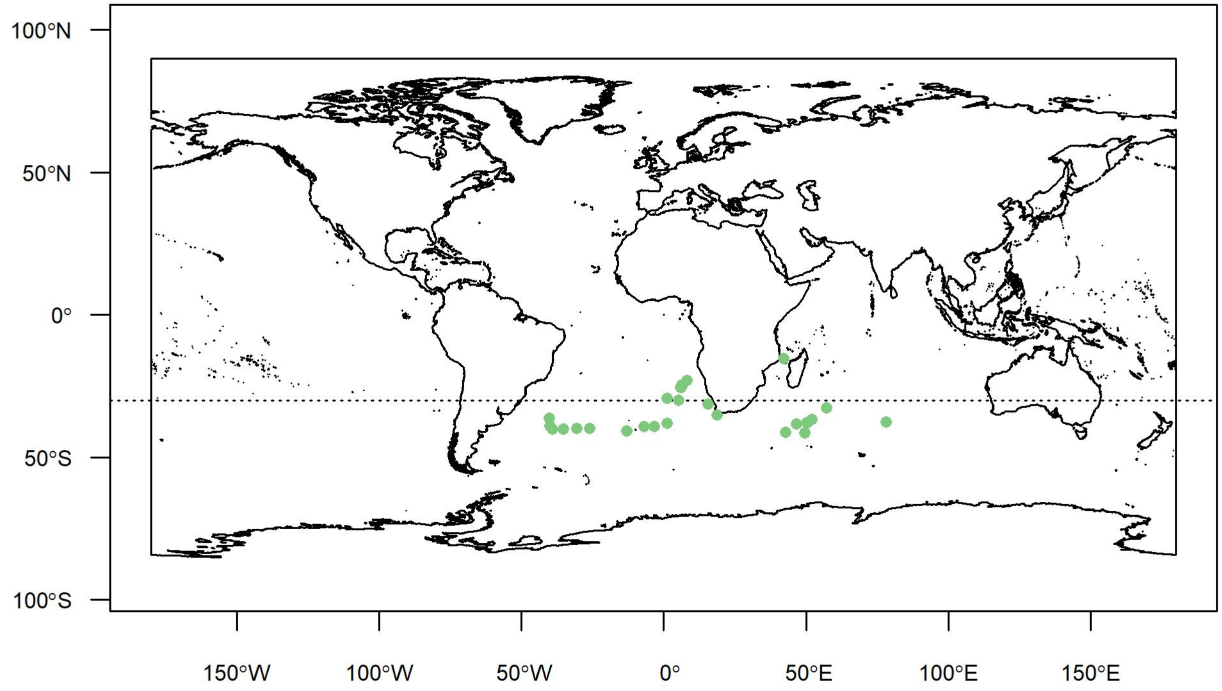

Lampadena luminosa

We found no outliers for this species.

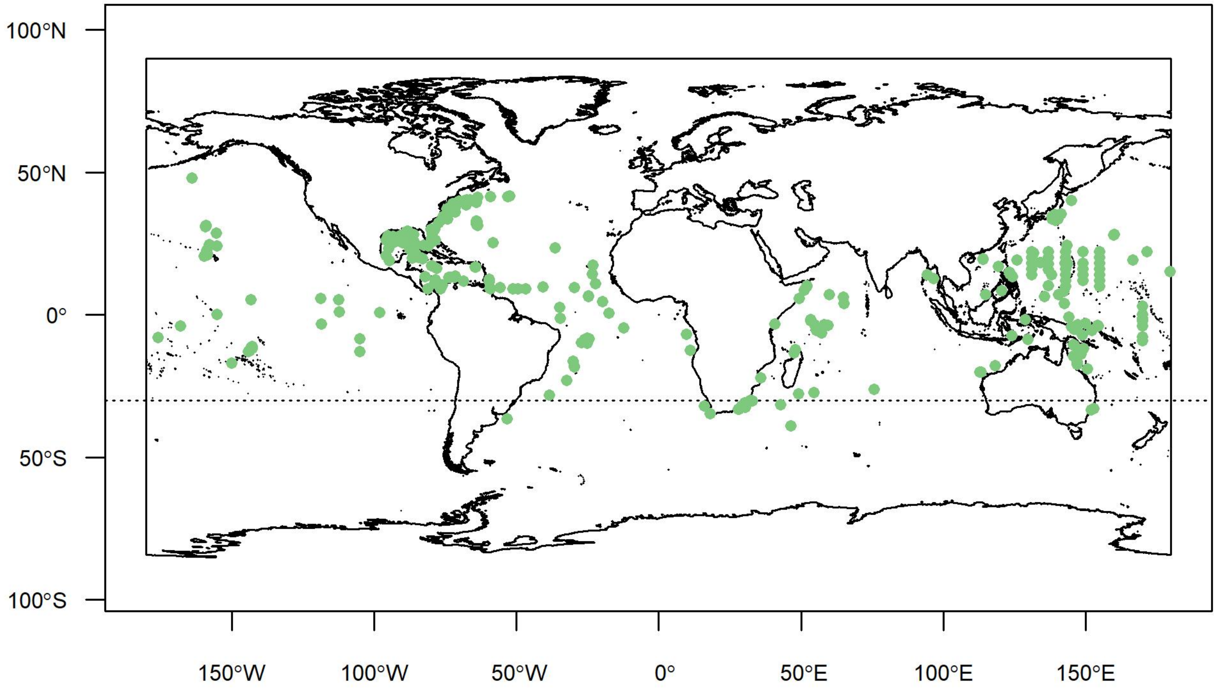

Lampadena notialis

We found no outliers for this species.

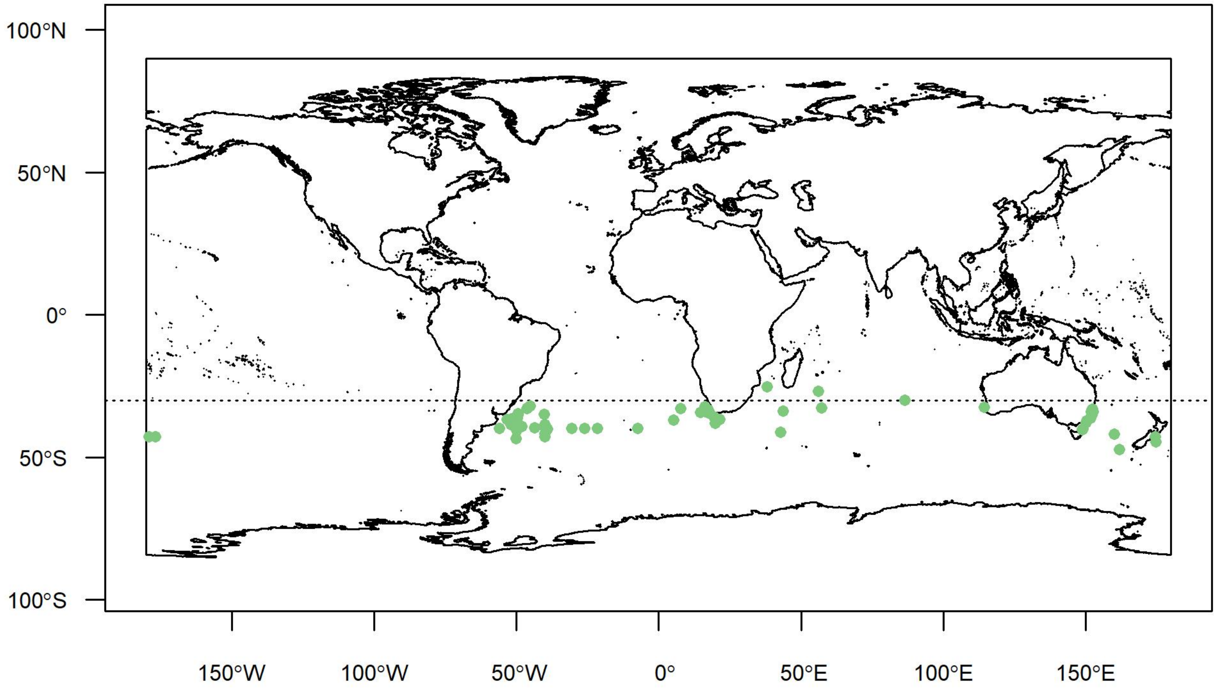

Lampadena speculigera

We found no outliers for this species.

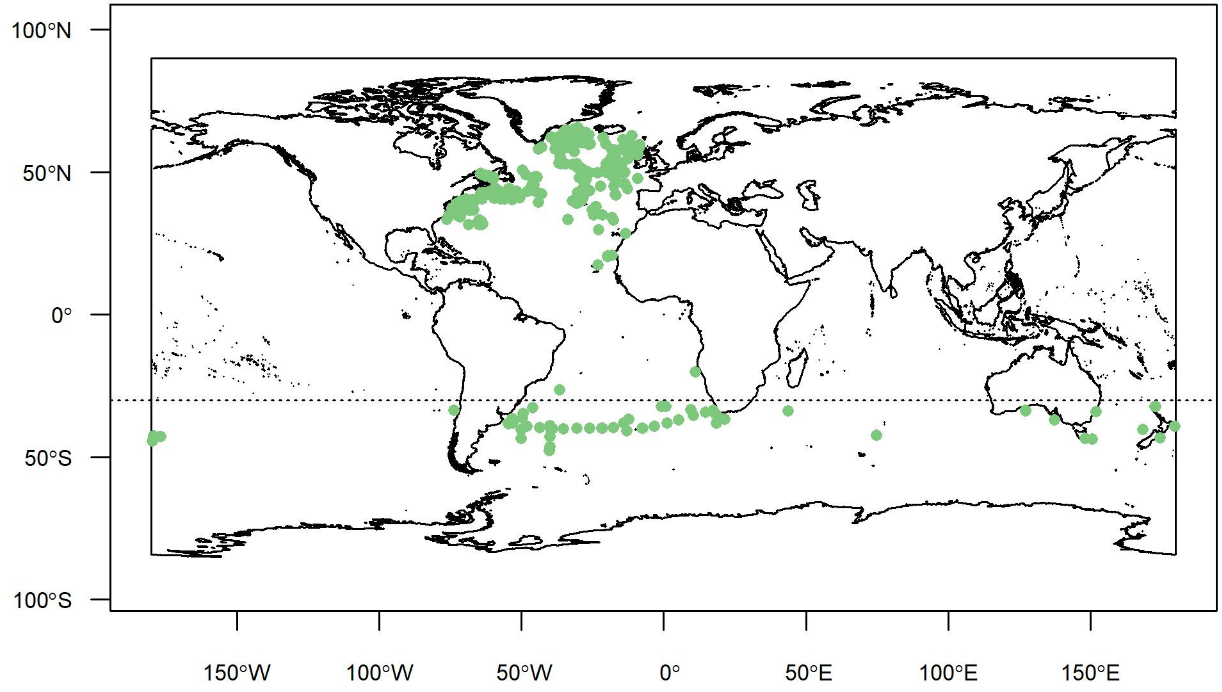

Lampanyctodes hectoris

We found two records with swapped coordinates from the Eltanin GBIF erroneous batch (blue points in map). We corrected their coordinates (purple points in map).

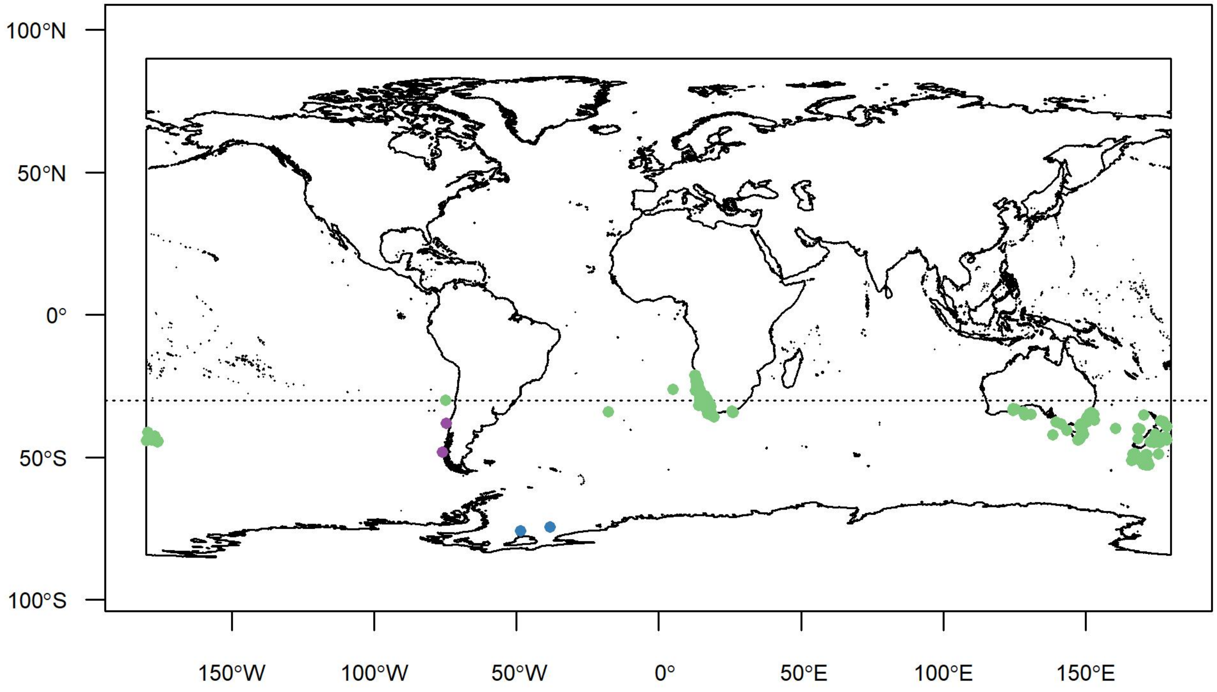

Information about Eltanin records with swapped coordinates are indicated here:

	source	year	references	bibliographicCitation
568501	GBIF	1962	http://portal.vertnet.org/o/lacm/fish?id=14183670-714d-4119-8d07-a4ed530f9555	14183670-714D-4119-8D07-A4ED530F9555. http://ipt.vertnet.org:8080/ipt/resource.do?r=lacm_verts.
689801	GBIF	1962	http://portal.vertnet.org/o/lacm/fish?id=d0aaf1cb-7406-4095-a5fd-6e3aa7db646c	D0AAF1CB-7406-4095-A5FD-6E3AA7DB646C. http://ipt.vertnet.org:8080/ipt/resource.do?r=lacm_verts.

Lampanyctus achirus

We found multiple records with swapped coordinates from the Eltanin GBIF erroneous batch (blue points in map). We corrected their coordinates (purple points in map).

We found many outliers in tropical areas and the Northern Hemisphere (red points in map), which we all removed.

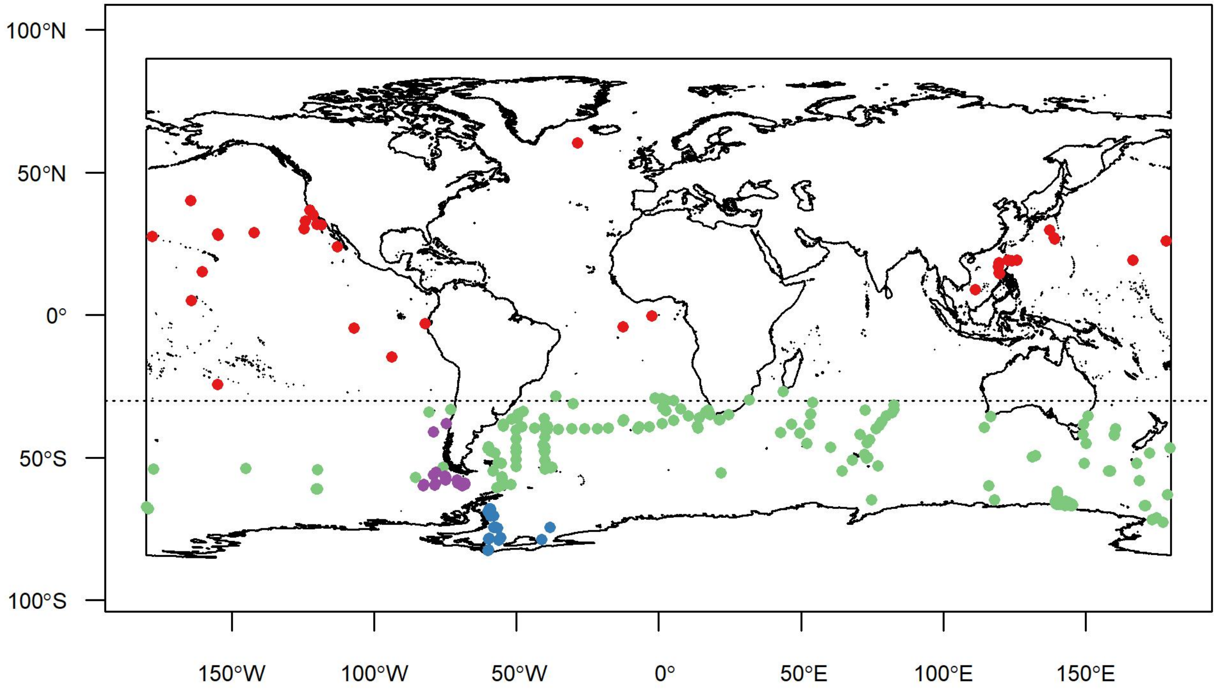

Information about Eltanin records with swapped coordinates are indicated here:

	source	year	references	bibliographicCitation
568841	GBIF	1963	http://portal.vertnet.org/o/lacm/fish?id=149f932a-a859-46fc-86c7-eb632161ed40	149F932A-A859-46FC-86C7-EB632161ED40. http://ipt.vertnet.org:8080/ipt/resource.do?r=lacm_verts.
573631	GBIF	1962	http://portal.vertnet.org/o/lacm/fish?id=1b8b0521-d09c-47ac-b51a-4284c82b3bc0	1B8B0521-D09C-47AC-B51A-4284C82B3BC0. http://ipt.vertnet.org:8080/ipt/resource.do?r=lacm_verts.
574451	GBIF	1962	http://portal.vertnet.org/o/lacm/fish?id=1ccd5638-9b9f-4349-99fe-4fc48b3a71c3	1CCD5638-9B9F-4349-99FE-4FC48B3A71C3. http://ipt.vertnet.org:8080/ipt/resource.do?r=lacm_verts.
575181	GBIF	1963	http://portal.vertnet.org/o/lacm/fish?id=1e234aaf-8dda-4230-a481-7d0526da0887	1E234AAF-8DDA-4230-A481-7D0526DA0887. http://ipt.vertnet.org:8080/ipt/resource.do?r=lacm_verts.
582021	GBIF	1963	http://portal.vertnet.org/o/lacm/fish?id=2880b112-cf0e-4c09-bbd1-63efb28b9dd7	2880B112-CF0E-4C09-BBD1-63EFB28B9DD7. http://ipt.vertnet.org:8080/ipt/resource.do?r=lacm_verts.
591281	GBIF	1962	http://portal.vertnet.org/o/lacm/fish?id=370087f0-152b-4869-891c-03e68d9b070d	370087F0-152B-4869-891C-03E68D9B070D. http://ipt.vertnet.org:8080/ipt/resource.do?r=lacm_verts.
612351	GBIF	1962	http://portal.vertnet.org/o/lacm/fish?id=57a04ac4-be41-46d4-a5ae-ee3bc37dd523	57A04AC4-BE41-46D4-A5AE-EE3BC37DD523. http://ipt.vertnet.org:8080/ipt/resource.do?r=lacm_verts.
613241	GBIF	1963	http://portal.vertnet.org/o/lacm/fish?id=58a9ddc2-d253-45e3-acad-9e44319a0b1b	58A9DDC2-D253-45E3-ACAD-9E44319A0B1B. http://ipt.vertnet.org:8080/ipt/resource.do?r=lacm_verts.
618531	GBIF	1963	http://portal.vertnet.org/o/lacm/fish?id=6170dbb7-a346-4a93-93cc-08e81a5578ab	6170DBB7-A346-4A93-93CC-08E81A5578AB. http://ipt.vertnet.org:8080/ipt/resource.do?r=lacm_verts.
622471	GBIF	1962	http://portal.vertnet.org/o/lacm/fish?id=67e24430-66f0-4dc6-95ca-02c08a6514e6	67E24430-66F0-4DC6-95CA-02C08A6514E6. http://ipt.vertnet.org:8080/ipt/resource.do?r=lacm_verts.
623251	GBIF	1962	http://portal.vertnet.org/o/lacm/fish?id=68b40f08-95cc-453a-a29d-e8aaea37a6b0	68B40F08-95CC-453A-A29D-E8AAEA37A6B0. http://ipt.vertnet.org:8080/ipt/resource.do?r=lacm_verts.
625271	GBIF	1963	http://portal.vertnet.org/o/lacm/fish?id=6b9cf304-ef82-4c53-a27f-7b2865f13fd0	6B9CF304-EF82-4C53-A27F-7B2865F13FD0. http://ipt.vertnet.org:8080/ipt/resource.do?r=lacm_verts.
668341	GBIF	1966	http://portal.vertnet.org/o/lacm/fish?id=af729bd6-ac4a-451f-9162-c38936a458a6	AF729BD6-AC4A-451F-9162-C38936A458A6. http://ipt.vertnet.org:8080/ipt/resource.do?r=lacm_verts.
674921	GBIF	1963	http://portal.vertnet.org/o/lacm/fish?id=b8f0b261-13e3-4514-8854-8b2814d8a654	B8F0B261-13E3-4514-8854-8B2814D8A654. http://ipt.vertnet.org:8080/ipt/resource.do?r=lacm_verts.
685131	GBIF	1963	http://portal.vertnet.org/o/lacm/fish?id=c95ad7b7-9166-44b1-8081-838e89bafb78	C95AD7B7-9166-44B1-8081-838E89BAFB78. http://ipt.vertnet.org:8080/ipt/resource.do?r=lacm_verts.
692561	GBIF	1962	http://portal.vertnet.org/o/lacm/fish?id=d507c2a0-12c3-4d3f-8a5f-8a366db31669	D507C2A0-12C3-4D3F-8A5F-8A366DB31669. http://ipt.vertnet.org:8080/ipt/resource.do?r=lacm_verts.
704791	GBIF	1962	http://portal.vertnet.org/o/lacm/fish?id=e7e0dd57-0346-44b3-8867-46796e5fa101	E7E0DD57-0346-44B3-8867-46796E5FA101. http://ipt.vertnet.org:8080/ipt/resource.do?r=lacm_verts.
706831	GBIF	1963	http://portal.vertnet.org/o/lacm/fish?id=eaddfd0a-1e63-4509-bdb9-a5ce642bae3e	EADDFD0A-1E63-4509-BDB9-A5CE642BAE3E. http://ipt.vertnet.org:8080/ipt/resource.do?r=lacm_verts.
708151	GBIF	1963	http://portal.vertnet.org/o/lacm/fish?id=ece1587d-0e66-46fa-bd8b-2d371386537d	ECE1587D-0E66-46FA-BD8B-2D371386537D. http://ipt.vertnet.org:8080/ipt/resource.do?r=lacm_verts.

Information about the outliers :

	source	decimalLongitude	decimalLatitude	year	references
1293100	OBIS	-28.4150	60.2780	2004	
53014	OBIS	-12.4050	-4.1578	2009	
583411	GBIF	-164.3900	40.0400	1992	
213371	GBIF	111.3250	8.7667	1960	http://portal.vertnet.org/o/sio/marine-vertebrates?id=a2159cc4-78e4-47f1-b57c-77c3d6f1bbab
241061	GBIF	166.5617	19.2383	1968	http://portal.vertnet.org/o/sio/marine-vertebrates?id=a16f5aa8-bb15-4f1f-b0ac-517528d540a8
241541	GBIF	178.3267	25.7817	1968	http://portal.vertnet.org/o/sio/marine-vertebrates?id=b72011c4-ee72-4045-9b7e-d74d86ae988f
241691	GBIF	-177.7717	27.5400	1968	http://portal.vertnet.org/o/sio/marine-vertebrates?id=f80e2f95-2070-4e58-af87-0e32657885a5
242771	GBIF	-160.3267	14.9950	1968	http://portal.vertnet.org/o/sio/marine-vertebrates?id=1fb14940-2097-49e5-bf2a-11064d6ffb01
243661	GBIF	-164.2283	4.9833	1968	http://portal.vertnet.org/o/sio/marine-vertebrates?id=8714da9a-de53-4f95-8efa-7cb77cfcb1e0
245712	GBIF	-93.6250	-14.7667	1969	http://portal.vertnet.org/o/sio/marine-vertebrates?id=b28b1351-dac9-4334-a9b8-591eaa719ec7
250001	GBIF	139.0217	26.9733	1970	http://portal.vertnet.org/o/sio/marine-vertebrates?id=65e45711-e249-4451-a97b-88f9aa558e14
250461	GBIF	137.2650	29.7900	1970	http://portal.vertnet.org/o/sio/marine-vertebrates?id=e6efa485-8154-4303-8172-f92fc8b4715f
250871	GBIF	139.1750	26.5617	1970	http://portal.vertnet.org/o/sio/marine-vertebrates?id=f5a60ed1-9491-41bf-bf27-258367b3dae0
252102	GBIF	124.1750	18.8250	1970	http://portal.vertnet.org/o/sio/marine-vertebrates?id=0ff8d52c-8789-4a3a-a809-3c7447f78331
252191	GBIF	125.9717	19.1317	1970	http://portal.vertnet.org/o/sio/marine-vertebrates?id=2f22dfb6-ce34-4cfc-a714-03fb3ffa08fa
252461	GBIF	122.6950	19.1433	1970	http://portal.vertnet.org/o/sio/marine-vertebrates?id=fc14ba93-0134-40e4-96ef-d88507407bdc
253201	GBIF	119.4000	16.8667	1970	http://portal.vertnet.org/o/sio/marine-vertebrates?id=109af3c3-c0e8-4f32-9b4c-b246232c4f5e
253512	GBIF	119.5600	14.5767	1970	http://portal.vertnet.org/o/sio/marine-vertebrates?id=82994d48-7a83-4c6b-8abe-0a8f48c49a4f
253661	GBIF	119.8000	14.3283	1970	http://portal.vertnet.org/o/sio/marine-vertebrates?id=a2ca5615-b78e-4b46-bf2b-a2f7f46fea5e
253891	GBIF	119.5833	18.1650	1970	http://portal.vertnet.org/o/sio/marine-vertebrates?id=973edd3b-d9c1-4f19-a9b2-4bed4353cc95

	source	decimalLongitude	decimalLatitude	year	references
268721	GBIF	-154.6933	27.9933	1973	http://portal.vertnet.org/o/sio/marine-vertebrates?id=98b2e481-4c92-47c2-ab09-25717fab4c32
268901	GBIF	-154.9667	28.3317	1973	http://portal.vertnet.org/o/sio/marine-vertebrates?id=f8a3b6ed-3a14-4c8f-b46b-394daa45228c
270491	GBIF	-124.4500	30.1833	1970	http://portal.vertnet.org/o/sio/marine-vertebrates?id=a16fc7fd-0147-46af-b80f-ddd6279e554d
286231	GBIF	-112.9167	23.7667	1974	http://portal.vertnet.org/o/sio/marine-vertebrates?id=b311c836-33ae-404b-80d3-1f82a58ff910
288851	GBIF	-124.1167	32.8333	1986	http://portal.vertnet.org/o/sio/marine-vertebrates?id=7025d648-5e76-430d-88d1-4bc5186f737d
295961	GBIF	-122.4017	36.5950	1972	http://portal.vertnet.org/o/sio/marine-vertebrates?id=4e91763c-91b5-40a3-a4cd-aec98dab56e1
296312	GBIF	-121.4883	35.1200	1972	http://portal.vertnet.org/o/sio/marine-vertebrates?id=a15f861a-a89d-43af-a469-46a2e713fa24
303341	GBIF	-154.8333	-24.5667	1969	http://portal.vertnet.org/o/sio/marine-vertebrates?id=84e61b3b-65fd-4040-9670-1a566468feb2
309331	GBIF	-107.0333	-4.7500	1976	http://portal.vertnet.org/o/sio/marine-vertebrates?id=07c6ed76-3a06-40d9-9a88-e93168a2a558
353801	GBIF	-81.9167	-3.1333	1967	http://portal.vertnet.org/o/sio/marine-vertebrates?id=21ebda1c-4aa3-4d17-8c1f-131f75736a0d
424481	GBIF	-2.3603	-0.4011	2005	
555301	GBIF	-120.1250	31.7458	1966	http://portal.vertnet.org/o/lacm/fish?id=0034c09d-cd76-42fb-a4ea-4bdca44bd54c
580081	GBIF	-118.5025	31.5975	1967	http://portal.vertnet.org/o/lacm/fish?id=25964e5c-3b4c-4c8c-b838-269a984d33bc
628961	GBIF	-141.9833	28.8000	1966	http://portal.vertnet.org/o/lacm/fish?id=71adc7c5-3939-4972-a9bd-9ca296c088ef

Lampanyctus alatus

We found one outlier close to the Antarctic continent (red point on the map), which we removed.

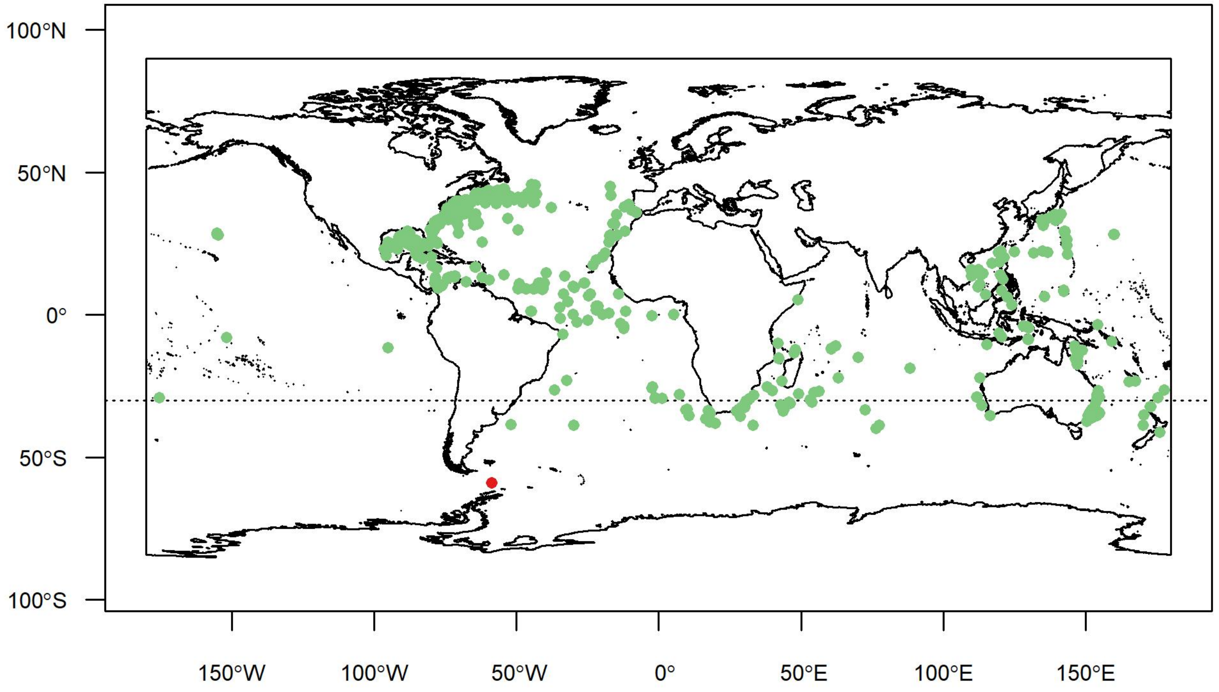

Information about the outlier :

	source	decimalLongitude	decimalLatitude	year	references
39033	OBIS	-58.5367	-59.155	1996	

Lampanyctus ater

We found multiple records in Mid-Pacific and Indonesia, which may be a confusion with *Lampanyctus niger* (ex - *Nannobrachium nigrum*) according to species distribution from Zahuranec, 2000. Hence, we removed them.

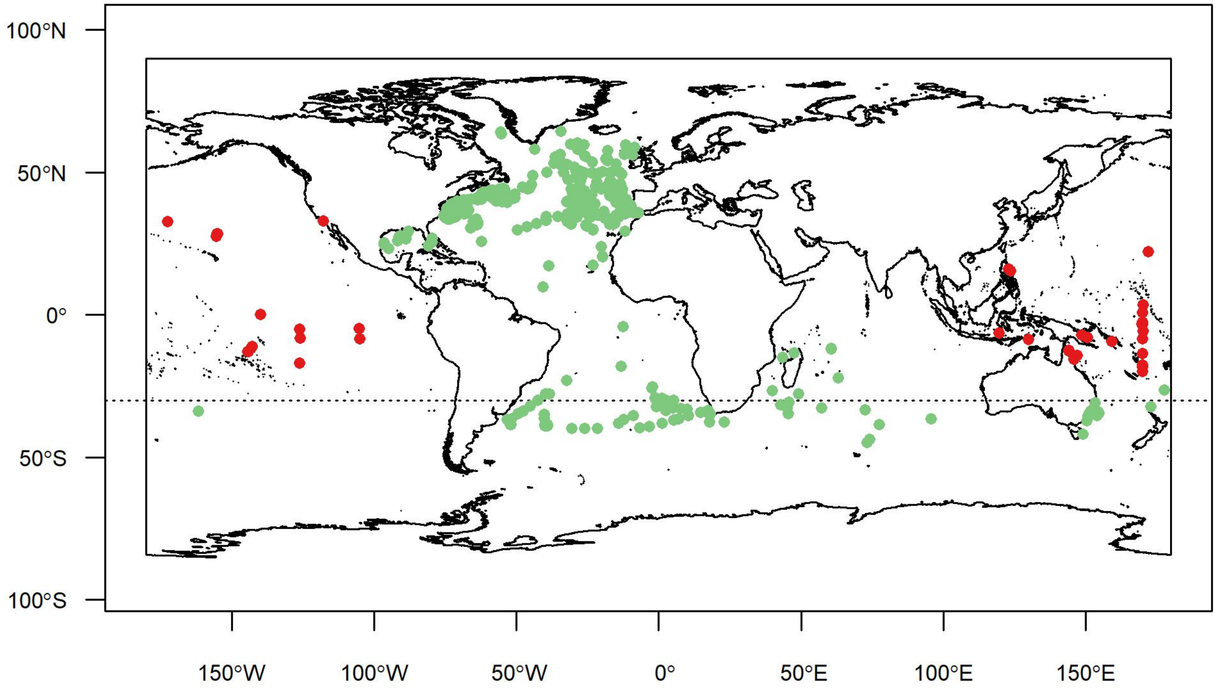

Information about the outliers :

	source	decimalLongitude	decimalLatitude	year	references
1191510	OBIS	170.0500	-18.4330	1966	
1265510	OBIS	-144.2170	-13.0670	1969	
1474610	OBIS	169.9170	-2.6670	1965	
25466	OBIS	-144.2500	-12.9500	1968	
31493	OBIS	-142.7670	-11.2670	1969	
33164	OBIS	169.9170	-3.3330	1965	
49434	OBIS	170.0500	-20.0000	1966	

	source	decimalLongitude	decimalLatitude	year	references
55819	OBIS	170.0170	-8.6000	1966	
67435	OBIS	-139.9330	0.0330	1969	
82349	OBIS	169.8830	-13.7670	1966	
87904	OBIS	170.2830	-5.7500	1966	
100437	OBIS	169.8830	0.6500	1966	
102445	OBIS	-143.1670	-11.9670	1968	
115489	OBIS	170.0000	-17.8170	1966	
118896	OBIS	169.7500	-3.3000	1966	
122448	OBIS	170.2170	3.5000	1966	
618811	GBIF	119.5700	-6.4003	1985	
206771	GBIF	-172.3550	32.6267	1956	http://portal.vertnet.org/o/sio/marine-vertebrates?id=fea38787-3f6d-44a5-b9a5-2946cc43f296
241431	GBIF	171.9517	22.1667	1968	http://portal.vertnet.org/o/sio/marine-vertebrates?id=01299c7b-cb90-450b-85a0-b7a8e2c3162c
258261	GBIF	-155.4067	27.3600	1971	http://portal.vertnet.org/o/sio/marine-vertebrates?id=51892a16-4d59-45b9-afe9-b9f84a0fb565
268701	GBIF	-154.9667	28.3317	1973	http://portal.vertnet.org/o/sio/marine-vertebrates?id=041a43bb-70a8-4588-9c27-8bcc211667af
269361	GBIF	-155.2350	28.3467	1973	http://portal.vertnet.org/o/sio/marine-vertebrates?id=3f665407-c33e-439c-b196-b6f77d4119bd
333491	GBIF	159.1000	-9.4167	1975	http://portal.vertnet.org/o/sio/marine-vertebrates?id=9f33f419-f2d9-4493-96c2-446ff5a0397c
347761	GBIF	-126.0000	-5.1500	1967	http://portal.vertnet.org/o/sio/marine-vertebrates?id=17de2943-e6c2-4237-b2f0-4249b45ba2f4
347991	GBIF	-125.9833	-8.2500	1967	http://portal.vertnet.org/o/sio/marine-vertebrates?id=23c128ce-aa21-468b-922a-eafb4f18fc1e
348261	GBIF	-126.0300	-17.0883	1967	http://portal.vertnet.org/o/sio/marine-vertebrates?id=78c65a94-c8c3-469d-92ae-4f8296474ce3
348751	GBIF	-104.9833	-8.5333	1967	http://portal.vertnet.org/o/sio/marine-vertebrates?id=7a010272-2679-4d0a-9202-db290401049c
349851	GBIF	-105.1167	-4.9100	1967	http://portal.vertnet.org/o/sio/marine-vertebrates?id=d40b7a31-0fb1-43f4-85cd-e6d829314d46
956691	GBIF	129.9500	-8.8330	1975	
959691	GBIF	148.6660	-7.0660	1969	
960081	GBIF	150.7330	-8.0500	1969	
960121	GBIF	150.5330	-8.0500	1969	
964191	GBIF	144.0160	-12.6660	1979	
964271	GBIF	144.0500	-12.7500	1979	
974201	GBIF	123.5547	15.3134	1995	
974291	GBIF	123.0016	16.0630	1995	

	source	decimalLongitude	decimalLatitude	year	references
977221	GBIF	147.0830	-14.3830	1981	
1064071	GBIF	145.9760	-15.7361	2009	
1128712	GBIF	147.0917	-14.3917	1981	http://mczbase.mcz.harvard.edu/guid/MCZ:Ich:86689
147669	GBIF	123.5547	15.3135	1995	http://collections.peabody.yale.edu/search/Record/YPM-ICH-010075
148577	GBIF	-117.7835	32.8309	2016	

Lampanyctus australis

We found multiple records with swapped coordinates from the Eltanin GBIF erroneous batch (blue points in map). We corrected their coordinates (purple points in map).

We found several outliers in the Southern Ocean (red points in map), which we removed.

By comparing with our own records, we found that many records were erroneous and were in fact *Lampanyctus achirus* records, so we removed them (orange points in map).

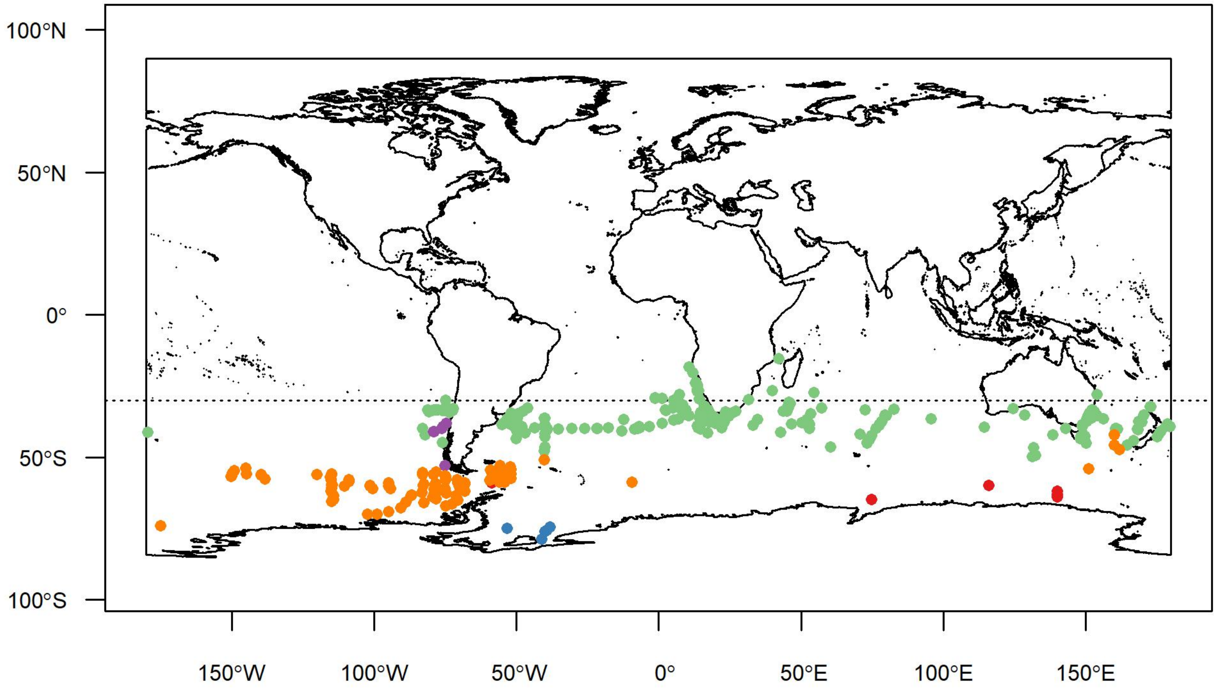

Information about Eltanin records with swapped coordinates are indicated here:

	source	year	references	bibliographicCitation
571881	GBIF	1964	http://portal.vertnet.org/o/lacm/fish?id=18bd4c9d-5f69-4324-b11c-bc4dd988ac87	18BD4C9D-5F69-4324-B11C-BC4DD988AC87. http://ipt.vertnet.org:8080/ipt/resource.do?r=lacm_verts.
577721	GBIF	1964	http://portal.vertnet.org/o/lacm/fish?id=21c92c50-c406-44e7-bb50-3d3ec0037bfc	21C92C50-C406-44E7-BB50-3D3EC0037BFC. http://ipt.vertnet.org:8080/ipt/resource.do?r=lacm_verts.
581561	GBIF	1966	http://portal.vertnet.org/o/lacm/fish?id=27dbcce5-9d35-4cc7-b87a-9e74d2e463ae	27DBCCE5-9D35-4CC7-B87A-9E74D2E463AE. http://ipt.vertnet.org:8080/ipt/resource.do?r=lacm_verts.

	source	year	references	bibliographicCitation
626701	GBIF	1966	http://portal.vertnet.org/o/lacm/fish?id=6dca7baa-ef09-4133-bff2-f8263d58233d	6DCA7BAA-EF09-4133-BFF2-F8263D58233D. http://ipt.vertnet.org:8080/ipt/resource.do?r=lacm_verts.
715421	GBIF	1964	http://portal.vertnet.org/o/lacm/fish?id=f7b8fd78-d6f1-4b00-be42-133fd29ae0ff	F7B8FD78-D6F1-4B00-BE42-133FD29AE0FF. http://ipt.vertnet.org:8080/ipt/resource.do?r=lacm_verts.
719491	GBIF	1962	http://portal.vertnet.org/o/lacm/fish?id=fe463c7e-a455-4bd1-b83e-9fb2aac6125e	FE463C7E-A455-4BD1-B83E-9FB2AAC6125E. http://ipt.vertnet.org:8080/ipt/resource.do?r=lacm_verts.

Information about the outliers :

	source	decimalLongitude	decimalLatitude	year	references
849	MyctoDB	74.8817	-64.9933	1991	AAMBER_2
1263	MyctoDB	139.9842	-63.9776	2008	Ceamarc_Umitaka_Maru
1572	MyctoDB	140.0105	-63.0319	2008	Ceamarc_Umitaka_Maru
2367	MyctoDB	139.9854	-61.9748	2008	Ceamarc_Umitaka_Maru
4439	MyctoDB	-9.3000	-58.9000	1964	Eltanin_Cruises
582411	GBIF	116.0003	-60.0003	1984	
4302	MyctoDB	-58.5400	-59.1600	1996	PolarStern_1996

Information about the *L. achirus* errors :

	source	decimalLongitude	decimalLatitude	year	references
15	MyctoDB	-174.8867	-74.1483	1968	Eltanin_Cruises
38	MyctoDB	-98.9500	-70.2167	1964	Eltanin_Cruises
47	MyctoDB	-102.2167	-70.1333	1964	Eltanin_Cruises
64	MyctoDB	-94.8333	-69.3000	1964	Eltanin_Cruises
137	MyctoDB	-90.4667	-67.7833	1964	Eltanin_Cruises
154	MyctoDB	-74.9000	-67.2833	1962	Eltanin_Cruises
287	MyctoDB	-72.6167	-66.4667	1962	Eltanin_Cruises
362	MyctoDB	-82.5333	-66.1333	1963	Eltanin_Cruises
543	MyctoDB	-88.8000	-65.7833	1964	Eltanin_Cruises
580	MyctoDB	-114.9000	-65.6833	1964	Eltanin_Cruises
729	MyctoDB	-70.5833	-65.1333	1962	Eltanin_Cruises
903	MyctoDB	-114.2167	-64.9667	1964	Eltanin_Cruises
952	MyctoDB	-78.1167	-64.7500	1963	Eltanin_Cruises
1289	MyctoDB	-79.2500	-63.9500	1963	Eltanin_Cruises
1338	MyctoDB	-114.2333	-63.7000	1964	Eltanin_Cruises
1347	MyctoDB	-71.2667	-63.6833	1962	Eltanin_Cruises

	source	decimalLongitude	decimalLatitude	year	references
1367	MyctoDB	-86.9000	-63.6500	1964	Eltanin_Cruises
1418	MyctoDB	-86.8333	-63.4500	1964	Eltanin_Cruises
1786	MyctoDB	-78.9333	-62.8167	1963	Eltanin_Cruises
1813	MyctoDB	-74.6833	-62.7333	1963	Eltanin_Cruises
1836	MyctoDB	-78.5667	-62.7000	1963	Eltanin_Cruises
1952	MyctoDB	-83.1333	-62.4833	1963	Eltanin_Cruises
2167	MyctoDB	-75.3000	-62.0667	1963	Eltanin_Cruises
2179	MyctoDB	-115.1167	-62.0500	1964	Eltanin_Cruises
2318	MyctoDB	-68.0167	-62.0000	1962	Eltanin_Cruises
2411	MyctoDB	-75.2333	-61.8667	1963	Eltanin_Cruises
2901	MyctoDB	-78.9167	-61.3333	1963	Eltanin_Cruises
3067	MyctoDB	-74.9667	-61.0833	1963	Eltanin_Cruises
3076	MyctoDB	-94.0833	-61.0667	1964	Eltanin_Cruises
3194	MyctoDB	-100.3500	-61.0167	1964	Eltanin_Cruises
3343	MyctoDB	-114.7833	-60.9167	1964	Eltanin_Cruises
3454	MyctoDB	-114.8500	-60.8000	1964	Eltanin_Cruises
3669	MyctoDB	-110.2500	-60.2833	1964	Eltanin_Cruises
3704	MyctoDB	-94.8167	-60.2333	1964	Eltanin_Cruises
3735	MyctoDB	-101.2667	-60.2000	1964	Eltanin_Cruises
3789	MyctoDB	-70.1333	-60.1167	1962	Eltanin_Cruises
3945	MyctoDB	-82.6667	-60.0000	1963	Eltanin_Cruises
3966	MyctoDB	-101.3000	-59.9833	1964	Eltanin_Cruises
4000	MyctoDB	-69.0000	-59.9333	1962	Eltanin_Cruises
4039	MyctoDB	-114.8833	-59.8333	1964	Eltanin_Cruises
4058	MyctoDB	-114.6667	-59.7500	1964	Eltanin_Cruises
4059	MyctoDB	-78.5833	-59.7500	1963	Eltanin_Cruises
4133	MyctoDB	-82.4500	-59.6333	1963	Eltanin_Cruises
4207	MyctoDB	-68.0167	-59.4833	1962	Eltanin_Cruises
4226	MyctoDB	-78.4500	-59.4167	1963	Eltanin_Cruises
4296	MyctoDB	-69.0167	-59.1667	1962	Eltanin_Cruises
4332	MyctoDB	-67.9833	-59.1000	1962	Eltanin_Cruises
4344	MyctoDB	-69.4667	-59.0833	1962	Eltanin_Cruises
4373	MyctoDB	-94.7833	-59.0167	1964	Eltanin_Cruises
4401	MyctoDB	-70.6167	-58.9833	1962	Eltanin_Cruises
4417	MyctoDB	-56.0000	-58.9333	1962	Eltanin_Cruises
4418	MyctoDB	-54.0833	-58.9333	1966	Eltanin_Cruises
4439	MyctoDB	-9.3000	-58.9000	1964	Eltanin_Cruises
4490	MyctoDB	-108.8333	-58.6167	1964	Eltanin_Cruises
4612	MyctoDB	-59.2167	-58.1667	1962	Eltanin_Cruises
4752	MyctoDB	-70.6667	-58.0000	1962	Eltanin_Cruises

	source	decimalLongitude	decimalLatitude	year	references
4778	MyctoDB	-115.0500	-57.9500	1964	Eltanin_Cruises
4809	MyctoDB	-56.8500	-57.9000	1966	Eltanin_Cruises
4826	MyctoDB	-108.7500	-57.8667	1964	Eltanin_Cruises
4827	MyctoDB	-74.7167	-57.8667	1963	Eltanin_Cruises
4841	MyctoDB	-115.0333	-57.8333	1964	Eltanin_Cruises
4929	MyctoDB	-138.3167	-57.6667	1964	Eltanin_Cruises
4945	MyctoDB	-55.9667	-57.6333	1962	Eltanin_Cruises
4960	MyctoDB	-51.8833	-57.5833	1966	Eltanin_Cruises
5025	MyctoDB	-74.7000	-57.3333	1963	Eltanin_Cruises
5075	MyctoDB	-115.2667	-57.1833	1963	Eltanin_Cruises
5157	MyctoDB	-56.0833	-57.0833	1962	Eltanin_Cruises
5265	MyctoDB	-74.7167	-56.9333	1963	Eltanin_Cruises
5355	MyctoDB	-150.0833	-56.7667	1964	Eltanin_Cruises
5421	MyctoDB	-149.9500	-56.6000	1964	Eltanin_Cruises
5428	MyctoDB	-74.9333	-56.5167	1963	Eltanin_Cruises
5441	MyctoDB	-58.4167	-56.4833	1962	Eltanin_Cruises
5460	MyctoDB	-139.5500	-56.4000	1964	Eltanin_Cruises
5474	MyctoDB	-58.1667	-56.3167	1962	Eltanin_Cruises
5503	MyctoDB	-114.9667	-56.2500	1963	Eltanin_Cruises
5528	MyctoDB	-120.1167	-56.2000	1964	Eltanin_Cruises
5557	MyctoDB	-139.5833	-56.1500	1964	Eltanin_Cruises
5575	MyctoDB	-79.0667	-56.1000	1963	Eltanin_Cruises
5594	MyctoDB	-82.8333	-56.0667	1963	Eltanin_Cruises
5605	MyctoDB	-114.8667	-56.0500	1963	Eltanin_Cruises
5658	MyctoDB	-51.7667	-55.9333	1966	Eltanin_Cruises
5659	MyctoDB	-144.7333	-55.9333	1964	Eltanin_Cruises
5695	MyctoDB	-56.0500	-55.8667	1962	Eltanin_Cruises
5724	MyctoDB	-149.7500	-55.6500	1964	Eltanin_Cruises
5800	MyctoDB	-82.9000	-55.4167	1963	Eltanin_Cruises
5826	MyctoDB	-55.7500	-55.3833	1962	Eltanin_Cruises
5838	MyctoDB	-78.1333	-55.3667	1963	Eltanin_Cruises
5894	MyctoDB	-53.5000	-55.2500	1966	Eltanin_Cruises
6146	MyctoDB	-149.1167	-54.8667	1964	Eltanin_Cruises
6265	MyctoDB	-58.9667	-54.6667	1962	Eltanin_Cruises
6453	MyctoDB	-51.8667	-54.4667	1966	Eltanin_Cruises
6671	MyctoDB	150.9833	-54.2167	1967	Eltanin_Cruises
6860	MyctoDB	-145.0333	-54.0167	1964	Eltanin_Cruises
7641	MyctoDB	-52.1333	-53.4833	1966	Eltanin_Cruises
7991	MyctoDB	-55.7000	-53.0167	1962	Eltanin_Cruises
8939	MyctoDB	-40.2500	-50.9000	1966	Eltanin_Cruises

	source	decimalLongitude	decimalLatitude	year	references
15821	MyctoDB	161.8667	-47.4667	1966	Eltanin_Cruises
16415	MyctoDB	160.1333	-45.7500	1966	Eltanin_Cruises
16984	MyctoDB	160.0833	-42.1333	1966	Eltanin_Cruises

Lampanyctus festivus

We found no outliers for this species.

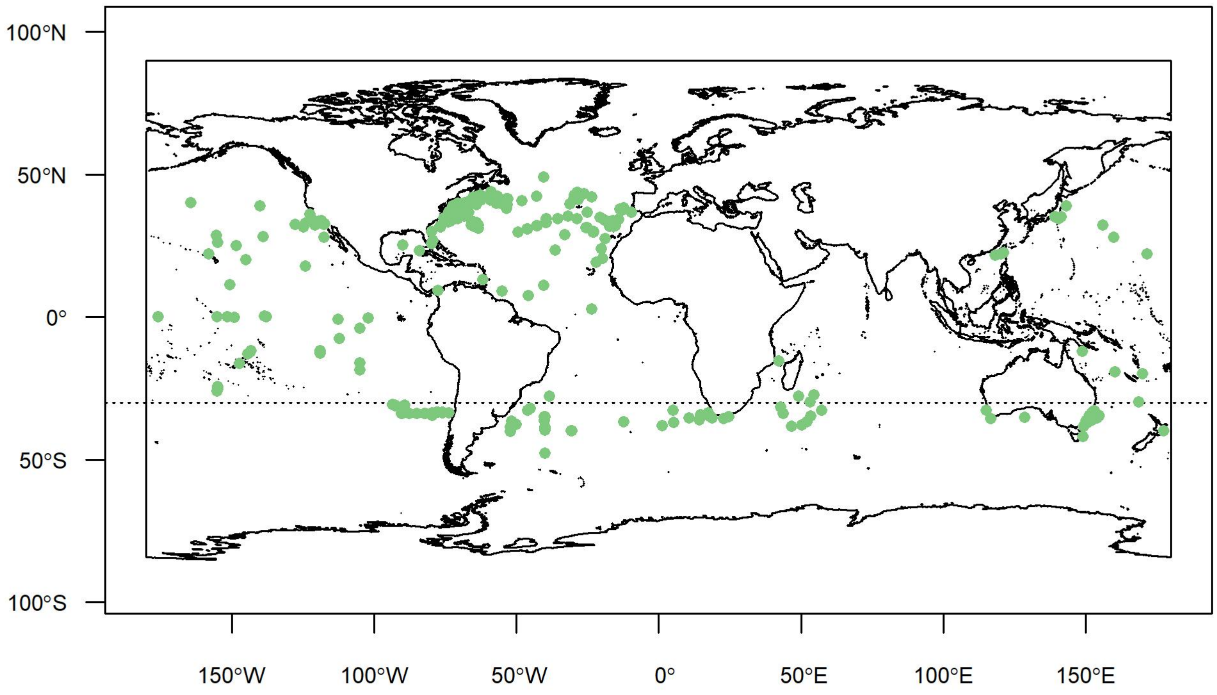

Lampanyctus intricarius

We found no outliers for this species.

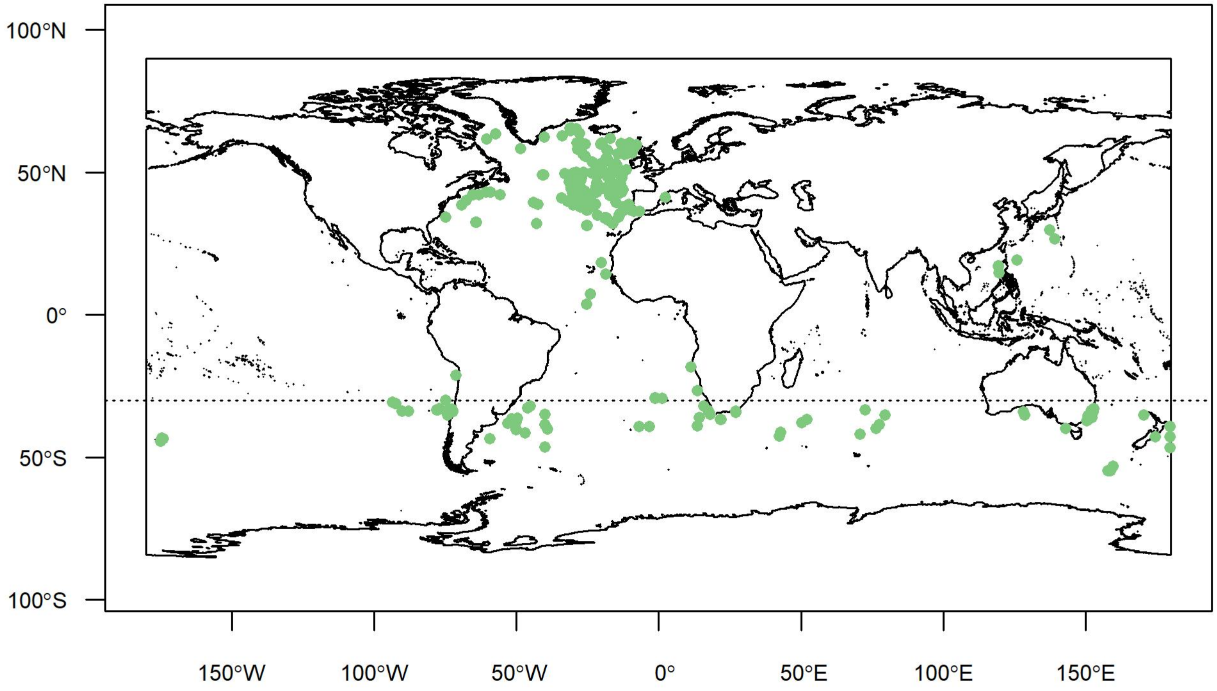

Lampanyctus iselinoides

We found four records with swapped coordinates from the Eltanin GBIF erroneous batch (blue points in map). We corrected their coordinates (purple points in map).

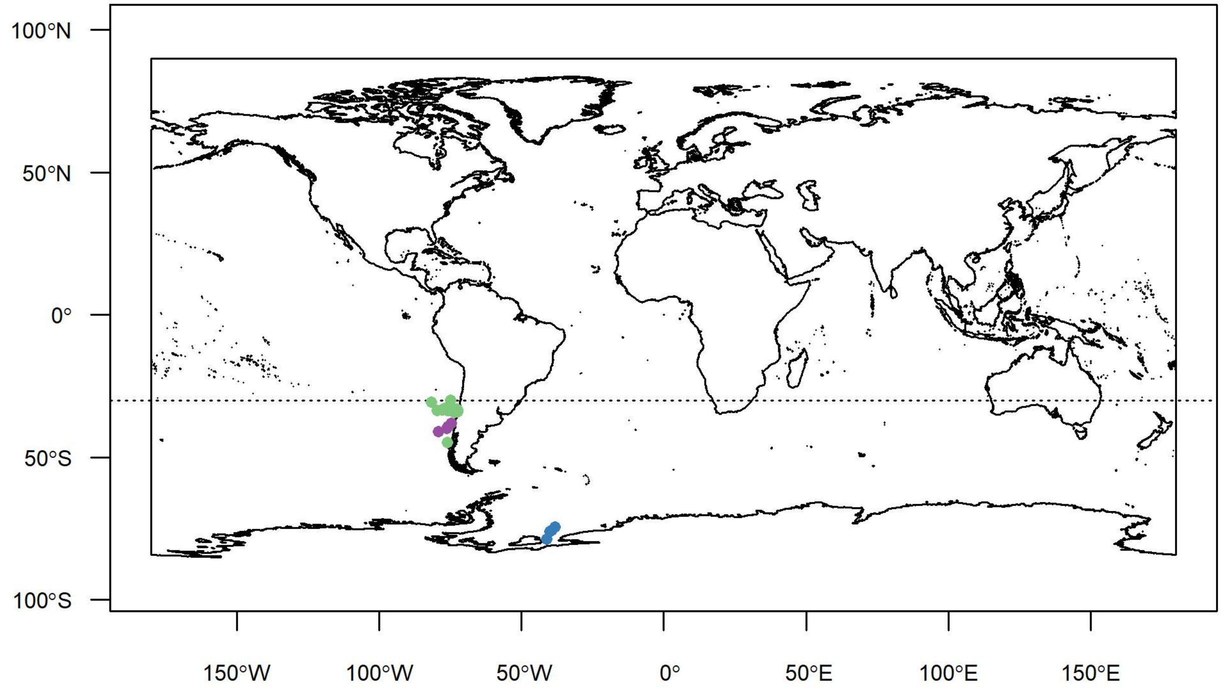

Information about Eltanin records with swapped coordinates are indicated here:

	source	year	references	bibliographicCitation
597801	GBIF	1964	http://portal.vertnet.org/o/lacm/fish?id=41dd39d5-9bc5-40c6-afe0-18901fc18dd4	41DD39D5-9BC5-40C6-AFE0-18901FC18DD4. http://ipt.vertnet.org:8080/ipt/resource.do?r=lacm_verts.
658312	GBIF	1964	http://portal.vertnet.org/o/lacm/fish?id=9f479e2a-5e65-4347-912b-ddcfde0dbf49	9F479E2A-5E65-4347-912B-DDCFDE0DBF49. http://ipt.vertnet.org:8080/ipt/resource.do?r=lacm_verts.
660031	GBIF	1966	http://portal.vertnet.org/o/lacm/fish?id=a22c0c16-1310-408c-a5d4-03a169ab908c	A22C0C16-1310-408C-A5D4-03A169AB908C. http://ipt.vertnet.org:8080/ipt/resource.do?r=lacm_verts.

	source	year	references	bibliographicCitation
690641	GBIF	1962	http://portal.vertnet.org/o/lacm/fish?id=d214cd36-505b-4d01-a96f-bc12fe72a36f	D214CD36-505B-4D01-A96F-BC12FE72A36F. http://ipt.vertnet.org:8080/ipt/resource.do?r=lacm_verts.

Lampanyctus lepidolychnus

We found several outliers in the Northern Hemisphere (red points on the map), which we removed.

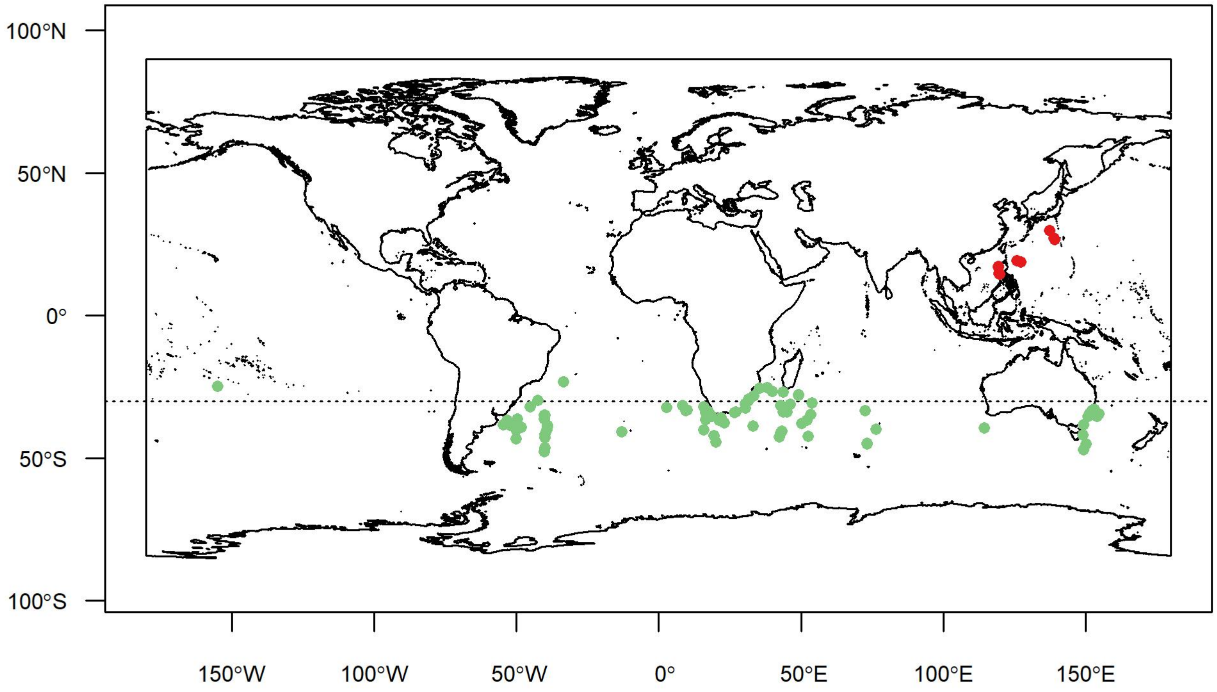

Information about the outliers :

	source	decimalLongitude	decimalLatitude	year	references
249912	GBIF	139.0217	26.9733	1970	http://portal.vertnet.org/o/sio/marine-vertebrates?id=6aa28b02-30b7-4b5a-96cc-b441b3c77b83
250441	GBIF	137.2650	29.7900	1970	http://portal.vertnet.org/o/sio/marine-vertebrates?id=d6d8b72c-6cf2-4b2a-8091-66134fd7639b
250912	GBIF	139.1750	26.5617	1970	http://portal.vertnet.org/o/sio/marine-vertebrates?id=922b62d5-288b-48fd-81fb-666344338b6d
251831	GBIF	127.2800	18.6867	1970	http://portal.vertnet.org/o/sio/marine-vertebrates?id=94754b38-5a1e-4cbb-9cd9-ca853516797e
252221	GBIF	119.5600	14.5767	1970	http://portal.vertnet.org/o/sio/marine-vertebrates?id=95496a33-fe33-4e33-824d-2b98c40d0cef
252401	GBIF	125.9717	19.1317	1970	http://portal.vertnet.org/o/sio/marine-vertebrates?id=1f3c23d3-ab0d-4fd8-8cfc-22c4df0649c4
253141	GBIF	119.4600	17.1867	1970	http://portal.vertnet.org/o/sio/marine-vertebrates?id=0f2dd414-741c-4bf8-9e07-b1cd7c729e34
253321	GBIF	119.4000	16.8667	1970	http://portal.vertnet.org/o/sio/marine-vertebrates?id=ffcbbdba-0ba0-4c93-bc65-e590bfb3b5fb
253612	GBIF	119.8000	14.3283	1970	http://portal.vertnet.org/o/sio/marine-vertebrates?id=127a32c1-fc82-4f08-92f2-10e5b827bb2e

Lampanyctus macdonaldi

We found multiple records with swapped coordinates from the Eltanin GBIF erroneous batch (blue points in map). We corrected their coordinates (purple points in map).

We found several outliers in the Pacific Ocean (red points in map), which we removed.

Likewise, we found several outliers in the Atlantic Ocean (orange points in map), which we removed.

Finally, we also found a few dubious records in the Carribeans (brown points in map), which we also removed.

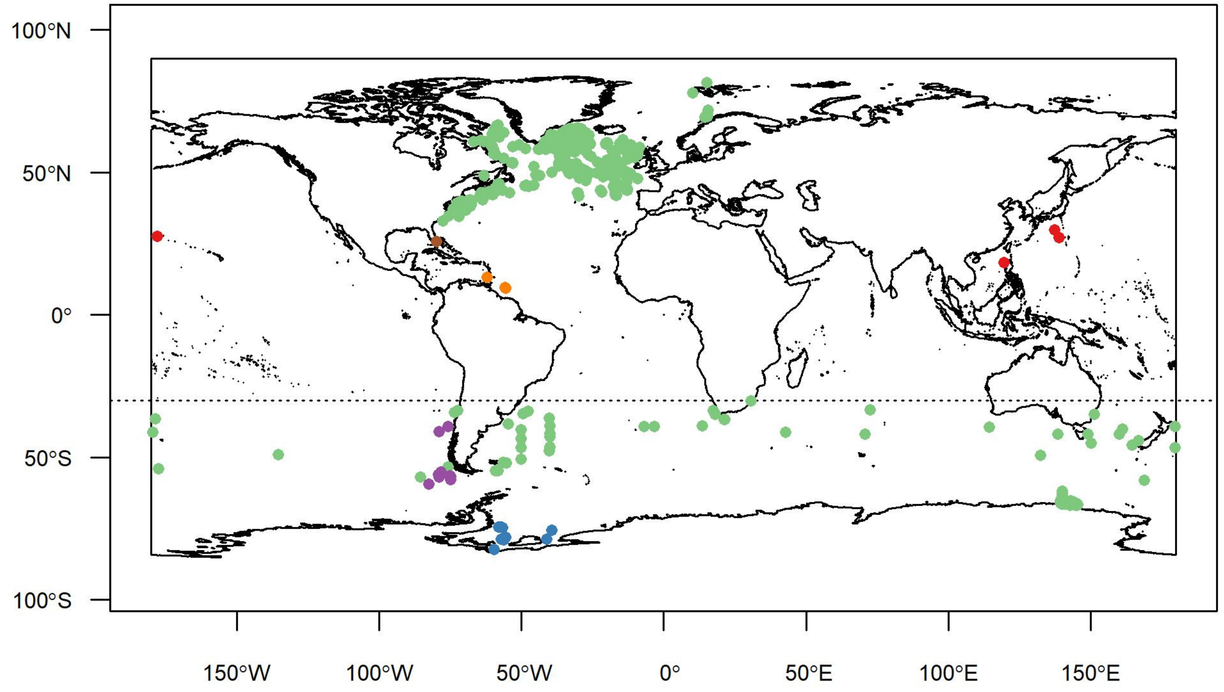

Information about Eltanin records with swapped coordinates are indicated here:

	source	year	references	bibliographicCitation
590571	GBIF	1963	http://portal.vertnet.org/o/lacm/fish?id=361f324d-4a34-4958-8210-04ab72535a24	361F324D-4A34-4958-8210-04AB72535A24. http://ipt.vertnet.org:8080/ipt/resource.do?r=lacm_verts.
608112	GBIF	1963	http://portal.vertnet.org/o/lacm/fish?id=50e0b9f6-b815-4c94-8c56-1e6a2d8730a4	50E0B9F6-B815-4C94-8C56-1E6A2D8730A4. http://ipt.vertnet.org:8080/ipt/resource.do?r=lacm_verts.
611231	GBIF	1964	http://portal.vertnet.org/o/lacm/fish?id=556760ff-804e-4c87-9341-7a6dab4c8f0d	556760FF-804E-4C87-9341-7A6DAB4C8F0D. http://ipt.vertnet.org:8080/ipt/resource.do?r=lacm_verts.

	source	year	references	bibliographicCitation
616851	GBIF	1963	http://portal.vertnet.org/o/lacm/fish?id=5ef97d47-6e3d-40fa-96d0-33539720c0af	5EF97D47-6E3D-40FA-96D0-33539720C0AF. http://ipt.vertnet.org:8080/ipt/resource.do?r=lacm_verts.
639212	GBIF	1963	http://portal.vertnet.org/o/lacm/fish?id=817439b0-c7c4-4a2c-9918-9f31b181d2a0	817439B0-C7C4-4A2C-9918-9F31B181D2A0. http://ipt.vertnet.org:8080/ipt/resource.do?r=lacm_verts.
644841	GBIF	1963	http://portal.vertnet.org/o/lacm/fish?id=8a786283-fd3c-4584-9589-ec87a1cb71d9	8A786283-FD3C-4584-9589-EC87A1CB71D9. http://ipt.vertnet.org:8080/ipt/resource.do?r=lacm_verts.
658912	GBIF	1966	http://portal.vertnet.org/o/lacm/fish?id=a02b5248-c4ed-47eb-89b8-b1c16d5e394d	A02B5248-C4ED-47EB-89B8-B1C16D5E394D. http://ipt.vertnet.org:8080/ipt/resource.do?r=lacm_verts.
661612	GBIF	1963	http://portal.vertnet.org/o/lacm/fish?id=a4e2f45f-8a09-4ee8-83ca-8b757b354bcf	A4E2F45F-8A09-4EE8-83CA-8B757B354BCF. http://ipt.vertnet.org:8080/ipt/resource.do?r=lacm_verts.
678721	GBIF	1963	http://portal.vertnet.org/o/lacm/fish?id=bf1c8d61-ec59-48ed-8657-0335ca320f5b	BF1C8D61-EC59-48ED-8657-0335CA320F5B. http://ipt.vertnet.org:8080/ipt/resource.do?r=lacm_verts.

Information about the Pacific Ocean outliers :

	source	decimalLongitude	decimalLatitude	year	references
96639	OBIS	137.2600	29.7900	1970	
241741	GBIF	-177.7717	27.5400	1968	http://portal.vertnet.org/o/sio/marine-vertebrates?id=2af01fd8-7ebf-4250-9abe-46b302265792
249931	GBIF	139.0217	26.9733	1970	http://portal.vertnet.org/o/sio/marine-vertebrates?id=10a049ac-1466-4e34-a846-4f8663eb7951
250451	GBIF	137.2650	29.7900	1970	http://portal.vertnet.org/o/sio/marine-vertebrates?id=4c643289-047a-4e37-b623-7d4f69fa99e6
252621	GBIF	119.5833	18.1650	1970	http://portal.vertnet.org/o/sio/marine-vertebrates?id=e146db78-0c54-41b6-84d7-94f6f25440ba

Information about the Atlantic Ocean outliers :

	source	decimalLongitude	decimalLatitude	year	references
542231	GBIF	-55.5833	9.4667	1968	
148198	GBIF	-55.4333	9.2500	1968	
148373	GBIF	-61.9200	13.1350	1966	

Information about the Carribeans outliers :

	source	decimalLongitude	decimalLatitude	year	references
536881	GBIF	-79.7667	25.5750	1962	
539191	GBIF	-79.7000	25.7167	1963	
539431	GBIF	-79.7833	25.6667	1963	
539921	GBIF	-79.8667	25.5833	1963	

Lampanyctus nobilis

We found no outliers for this species.

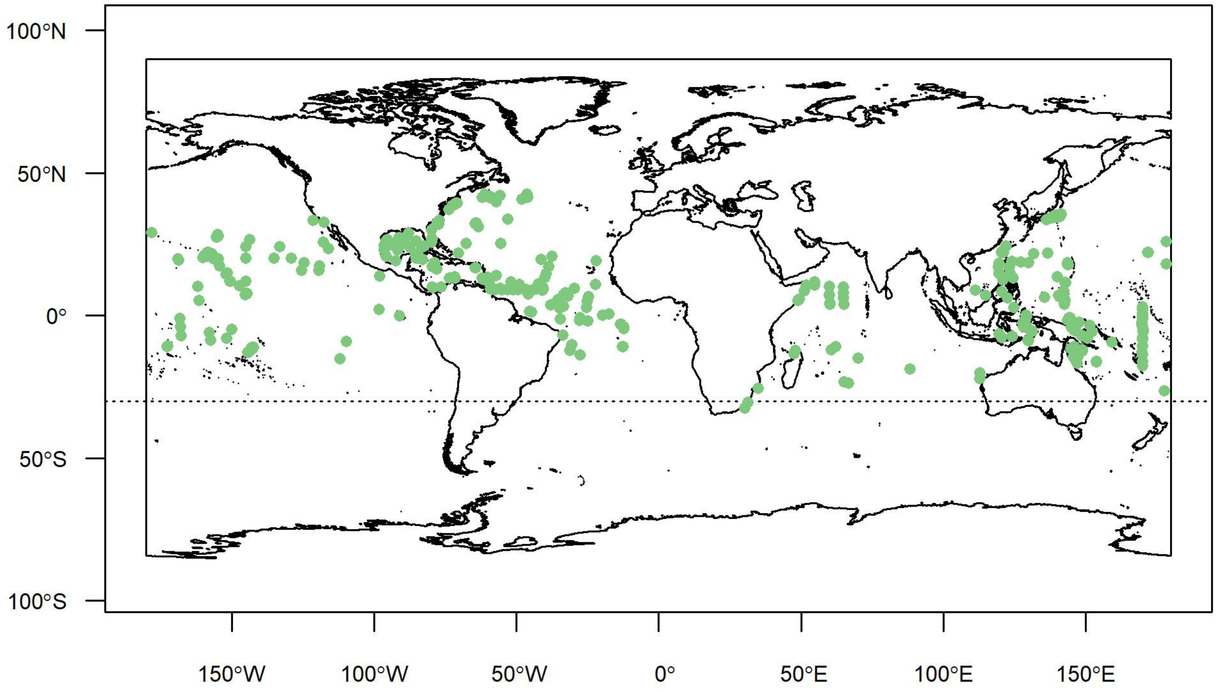

Lampanyctus phyllisae

We found no outliers for this species.

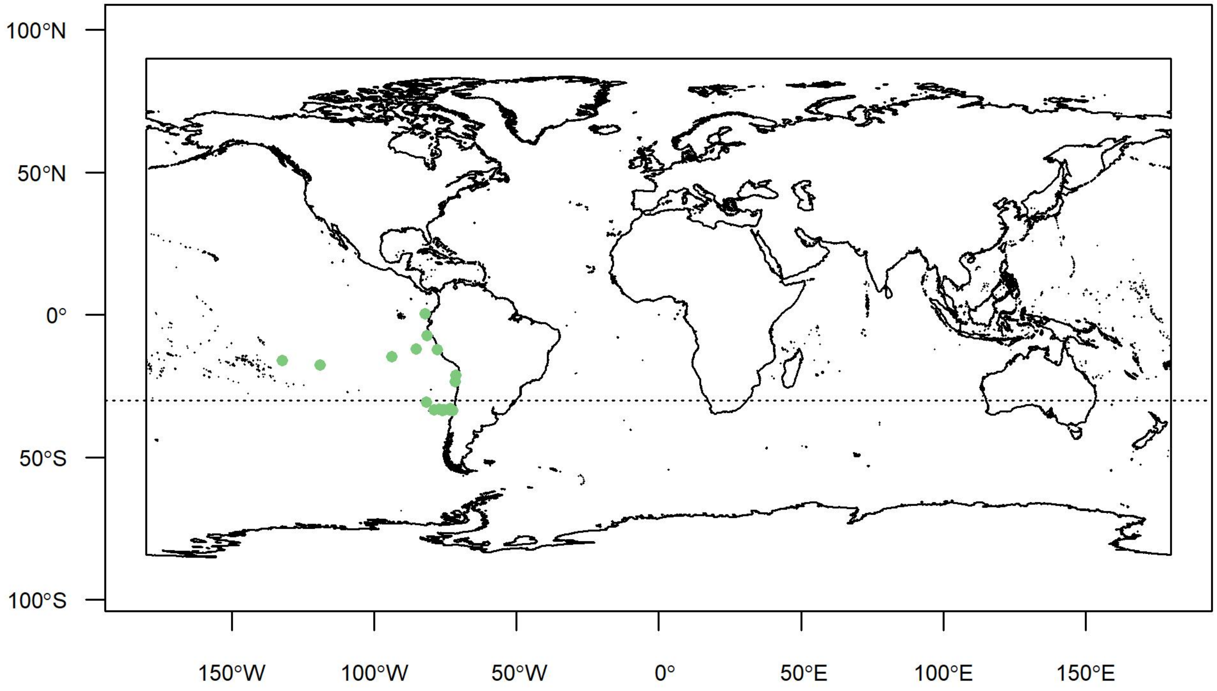

Lampanyctus pusillus

We found one record with swapped coordinates from the Eltanin GBIF erroneous batch (blue points in map). We corrected its coordinates (purple points in map).

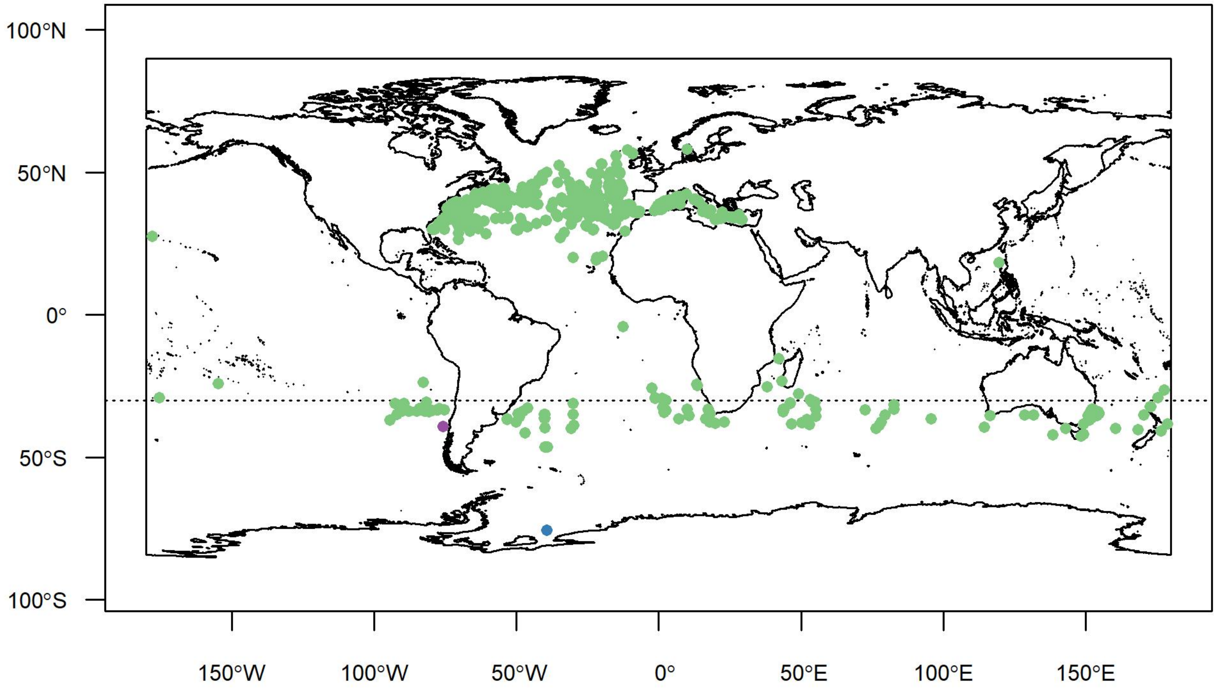

Information about Eltanin records with swapped coordinates are indicated here:

	source	year	references	bibliographicCitation
569281	GBIF	1964	http://portal.vertnet.org/o/lacm/fish?id=155da5e3-f43e-43e9-9959-652e4728ba66	155DA5E3-F43E-43E9-9959-652E4728BA66. http://ipt.vertnet.org:8080/ipt/resource.do?r=lacm_verts.

Lampanyctus tenuiformis

We found no outliers for this species.

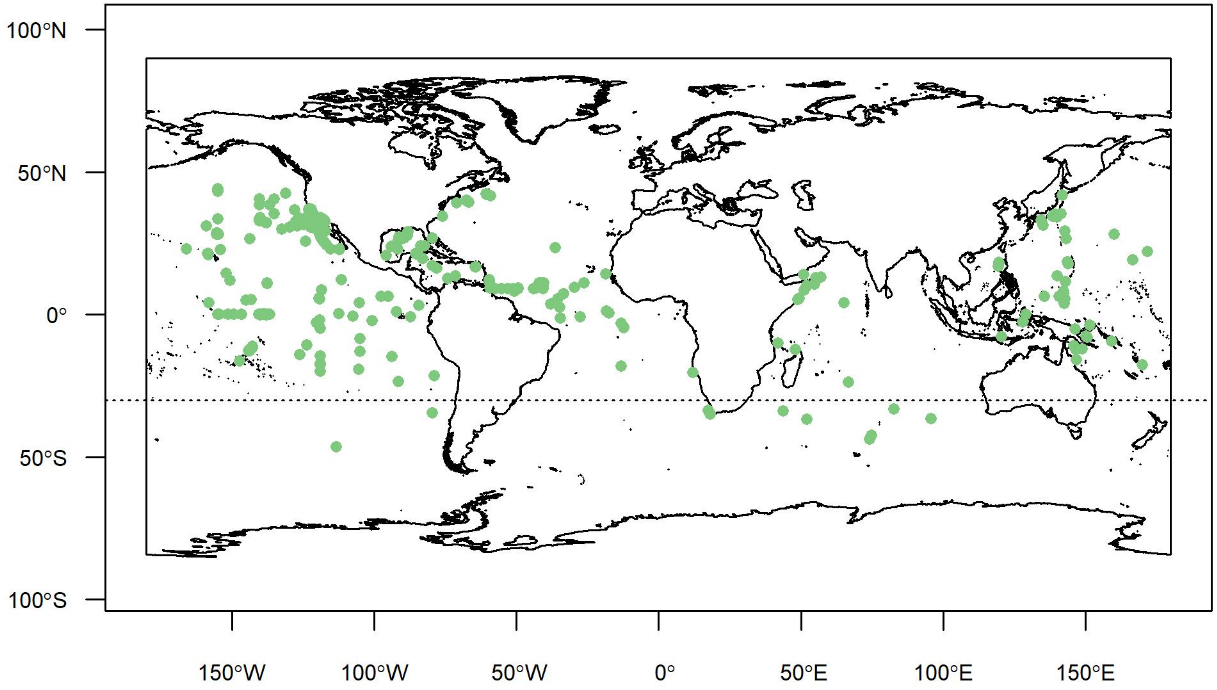

Lampanyctus turneri

We found two outliers in Central Pacific (red points on the map), which we removed.

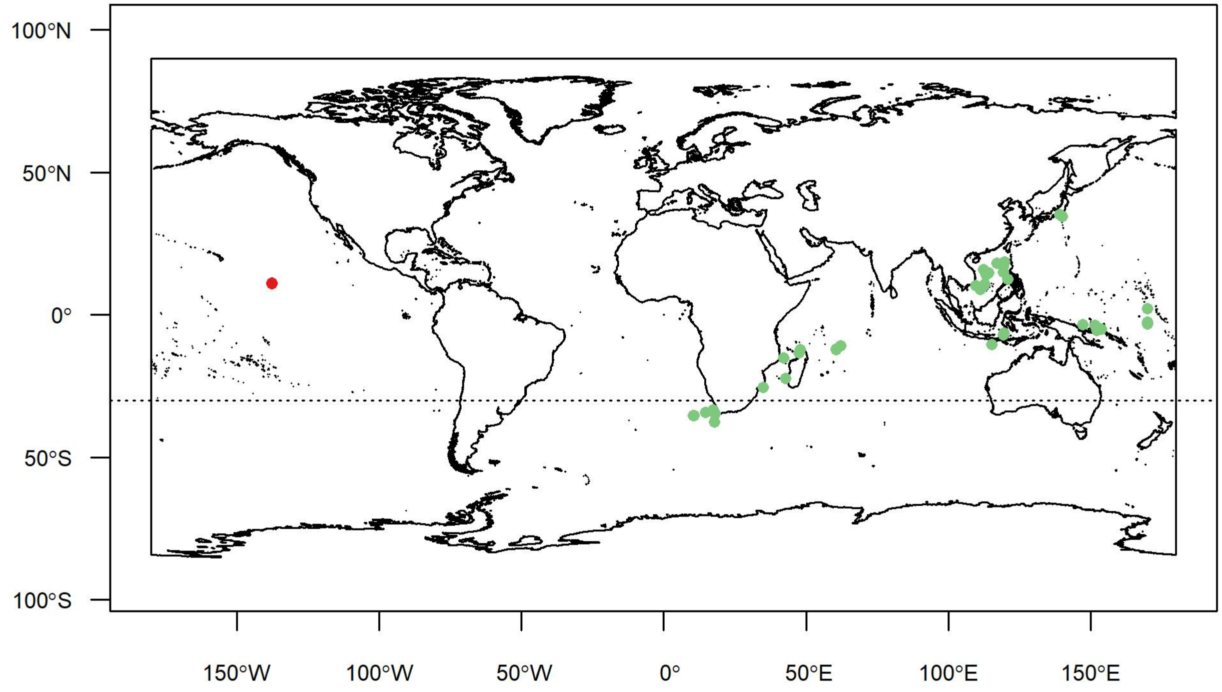

Information about the outlier :

	source	decimalLongitude	decimalLatitude	year	references
117498	OBIS	-137.60	10.90	1995	
229511	GBIF	-137.58	10.95	1995	

Lampanyctus wisneri

We found no outliers for this species.

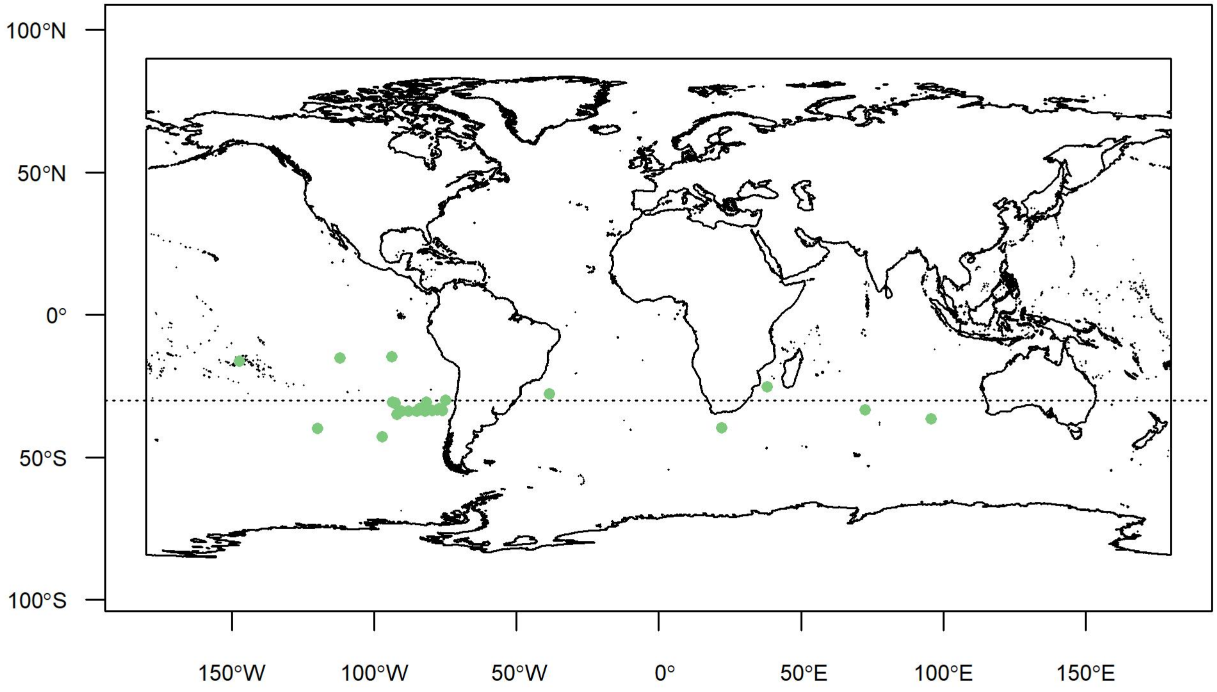

Lampichthys procerus

We found two records with swapped coordinates from the Eltanin GBIF erroneous batch (blue points in map). We corrected their coordinates (purple points in map).

We found several potential outliers in the Pacific Ocean (red points in map) which should be accurate given the expertise of their identifier (Bob Wisner), hence we kept them.

We found one outlier in the Atlantic Ocean (orange points in map), which we removed.

Finally, we also found one dubious record Northwest to Madagascar (brown point in map), which we also removed.

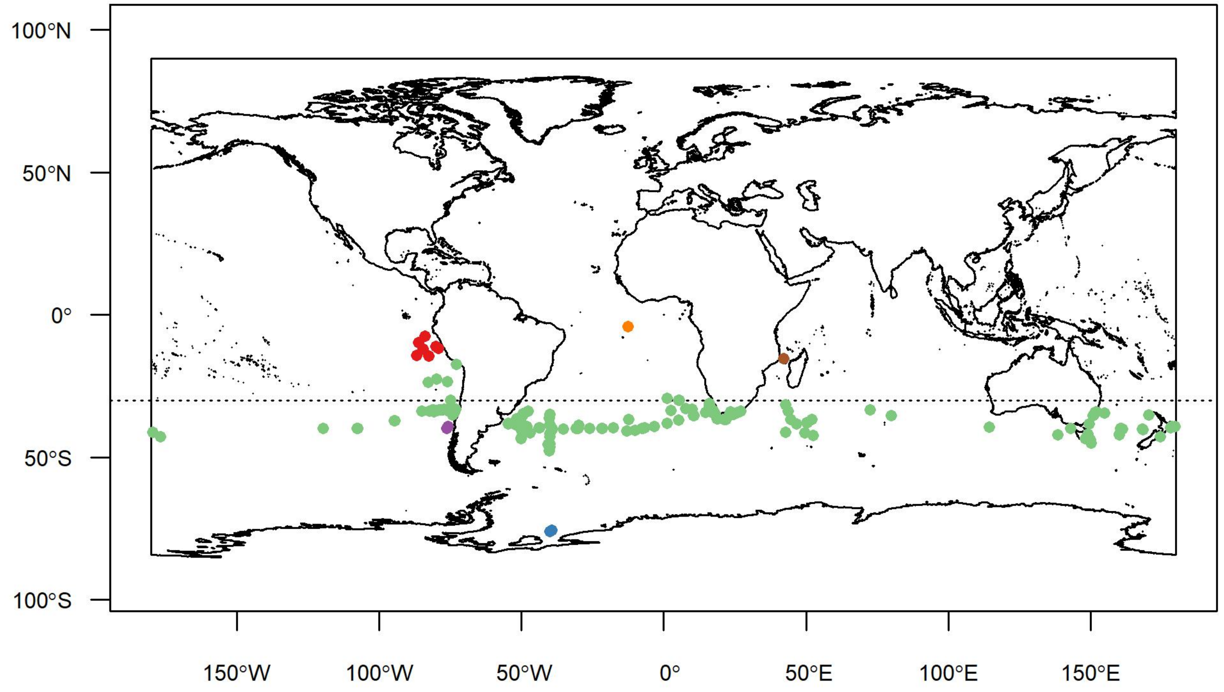

Information about Eltanin records with swapped coordinates are indicated here:

	source	year	references	bibliographicCitation
661461	GBIF	1964	http://portal.vertnet.org/o/lacm/fish?id=a4a0b37c-d22b-4b71-a250-6136eb82a6bd	A4A0B37C-D22B-4B71-A250-6136EB82A6BD. http://ipt.vertnet.org:8080/ipt/resource.do?r=lacm_verts.
691081	GBIF	1964	http://portal.vertnet.org/o/lacm/fish?id=d2ba8e1e-f263-4378-9743-f7355b1173c0	D2BA8E1E-F263-4378-9743-F7355B1173C0. http://ipt.vertnet.org:8080/ipt/resource.do?r=lacm_verts.

Information about the Pacific Ocean outliers :

	source	decimalLongitude	decimalLatitude	year	references
215871	GBIF	-83.8000	-7.5333	1960	http://portal.vertnet.org/o/sio/marine-vertebrates?id=4b8c1cfb-985e-4b01-aefc-144f1cd1c428
217001	GBIF	-80.0167	-11.1667	1960	http://portal.vertnet.org/o/sio/marine-vertebrates?id=277a5bc5-c549-4b10-b219-b030e0ab96e2
228721	GBIF	-79.0217	-11.9833	1965	http://portal.vertnet.org/o/sio/marine-vertebrates?id=b04b2512-45ef-4127-b485-b47b794a8bdc
352821	GBIF	-86.1000	-9.8667	1967	http://portal.vertnet.org/o/sio/marine-vertebrates?id=4058b138-f418-4a99-b982-28ab57373892
353091	GBIF	-84.5333	-12.1000	1967	http://portal.vertnet.org/o/sio/marine-vertebrates?id=73eef481-ec08-4610-b7ac-6f1b73027cd6
353112	GBIF	-86.8333	-14.4500	1967	http://portal.vertnet.org/o/sio/marine-vertebrates?id=8d457bc1-c980-4a7f-bc31-a38745631b89
354431	GBIF	-82.3667	-14.6667	1967	http://portal.vertnet.org/o/sio/marine-vertebrates?id=65f7ab74-e253-4a1b-9ac9-f496705fdd75

Information about the Atlantic Ocean outliers :

	source	decimalLongitude	decimalLatitude	year	references
738510	OBIS	-12.405	-4.1578	2009	

Information about the Madagascar outliers :

	source	decimalLongitude	decimalLatitude	year	references
140542	GBIF	42.25	-15.45	1988	

Lepidophanes gaussi

We found one outlier close to Uruguay (red point on the map), which we removed.

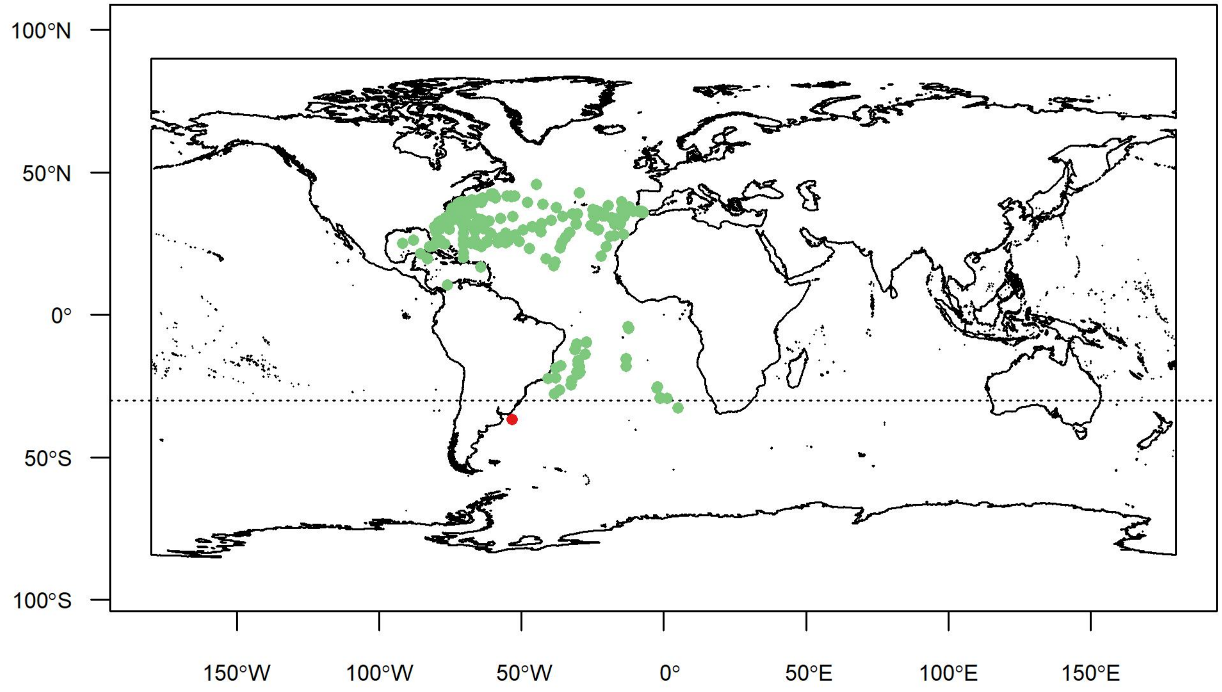

Information about the outlier :

	source	decimalLongitude	decimalLatitude	year	references
1107271	GBIF	-53.1083	-36.7583	1967	http://mczbase.mcz.harvard.edu/guid/MCZ:Ich:109421

Lepidophanes guentheri

We found one outlier in the Indian Ocean (red point on the map), which we removed because this species is endemic to the Atlantic Ocean.

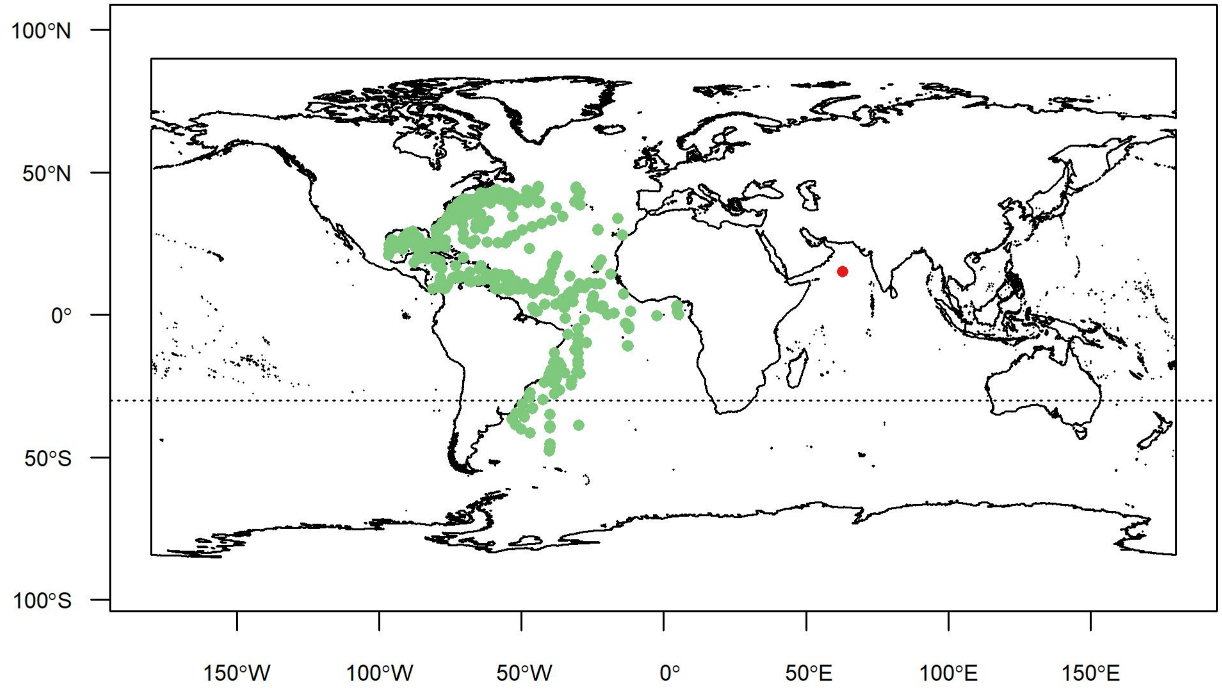

Information about the outlier :

	source	decimalLongitude	decimalLatitude	year	references
138839	GBIF	63	15	1974	

Lobianchia dofleini

We found one outlier in the Pacific Ocean (red point in map) which we removed.

We found one outlier in the Indian Ocean (orange point in map), which we removed.

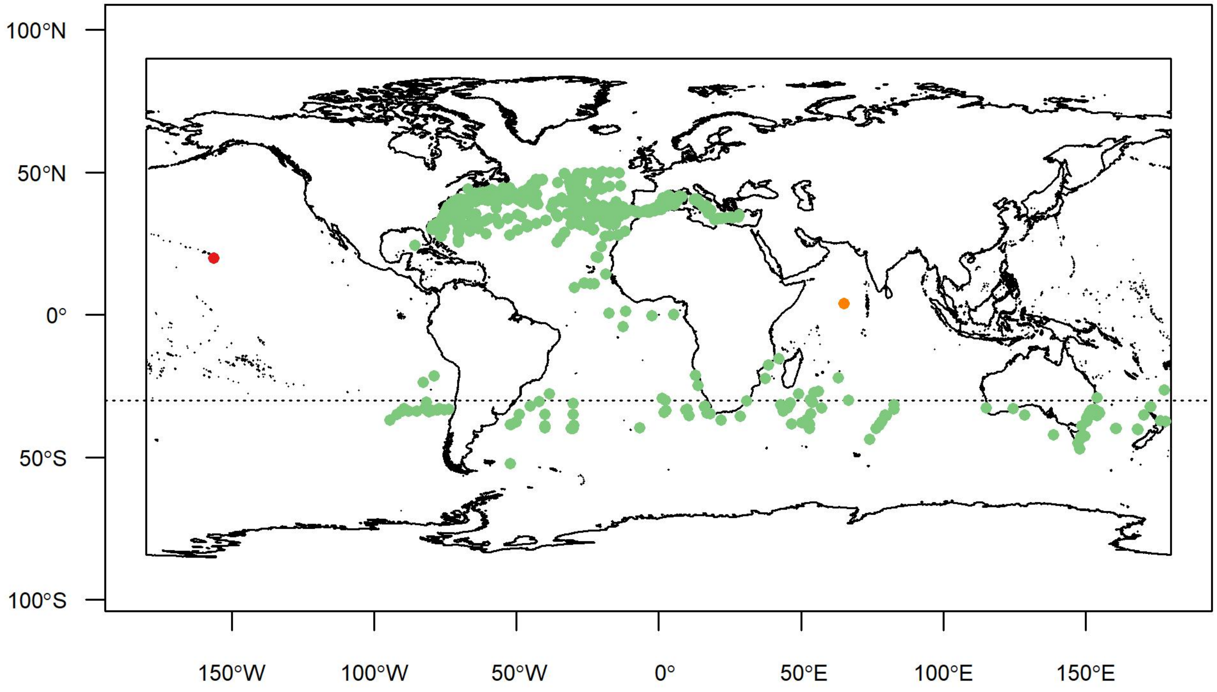

Information about the Pacific Ocean outliers :

	source	decimalLongitude	decimalLatitude	year	references
204281	GBIF	-156.255	19.83	1960	http://portal.vertnet.org/o/sio/marine-vertebrates?id=f385dcb8-2db7-48b9-9f34-865739bfda41

Information about the Indian Ocean outliers :

	source	decimalLongitude	decimalLatitude	year	references
573891	GBIF	65.0833	3.7667	1964	http://portal.vertnet.org/o/lacm/fish?id=1bddda8f-9001-434e-8916-35f15e920529

Lobianchia gemellarii

We found no outliers for this species.

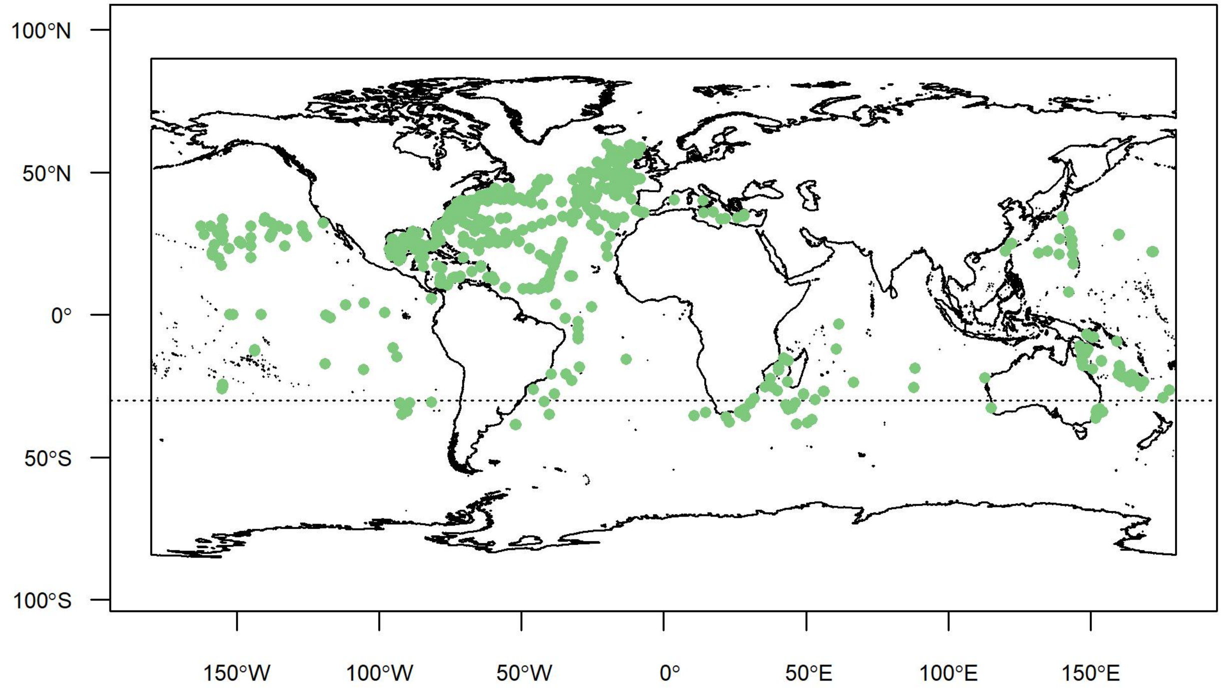

Loweina interrupta

We found no outliers for this species.

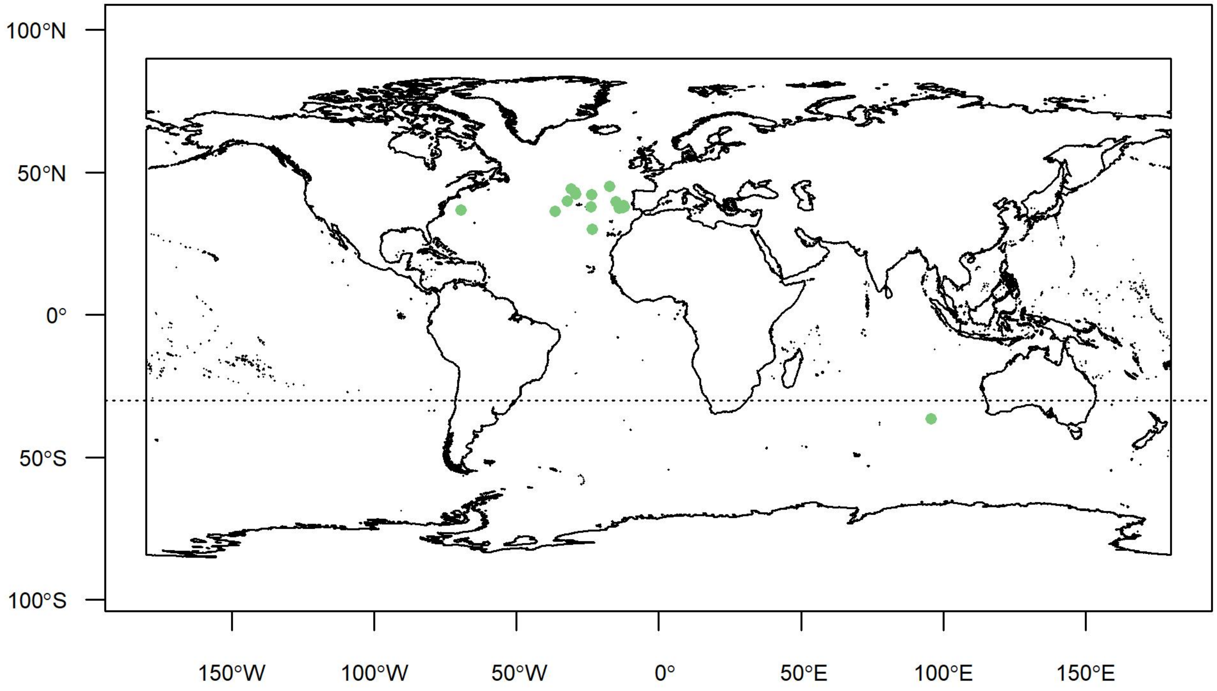

Loweina rara

We found no outliers for this species.

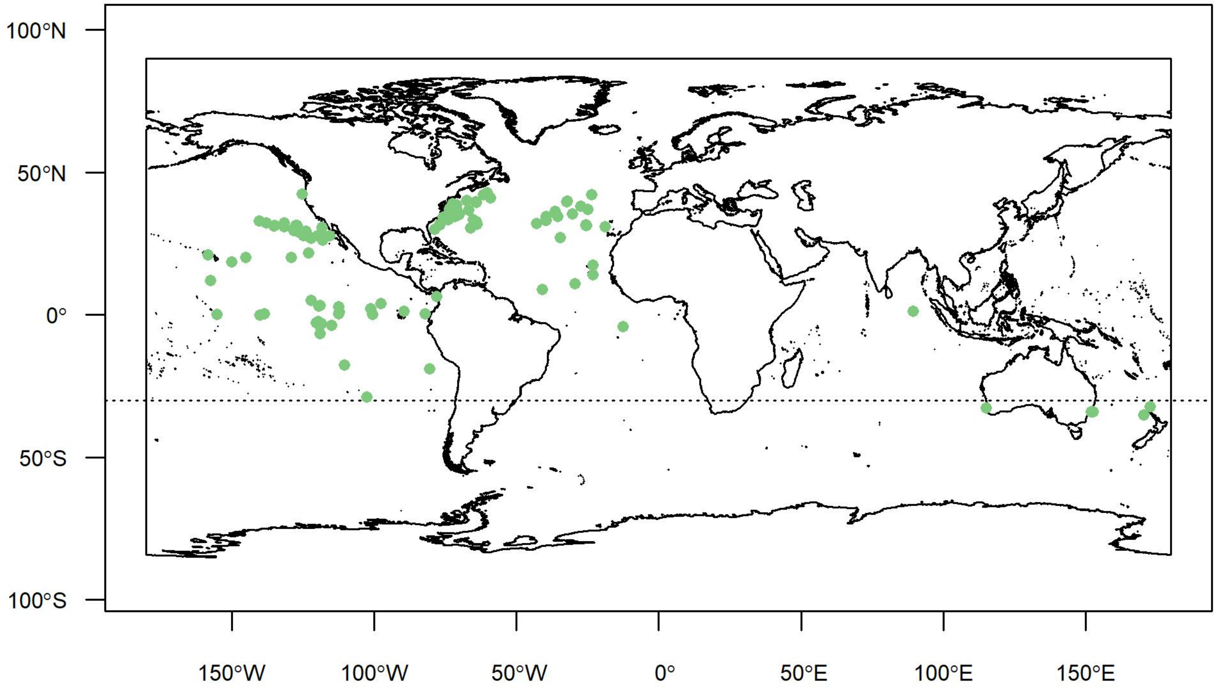

Metelectrona ahlstromi

We found two outliers in the Atlantic Ocean (red points on the map), which we removed.

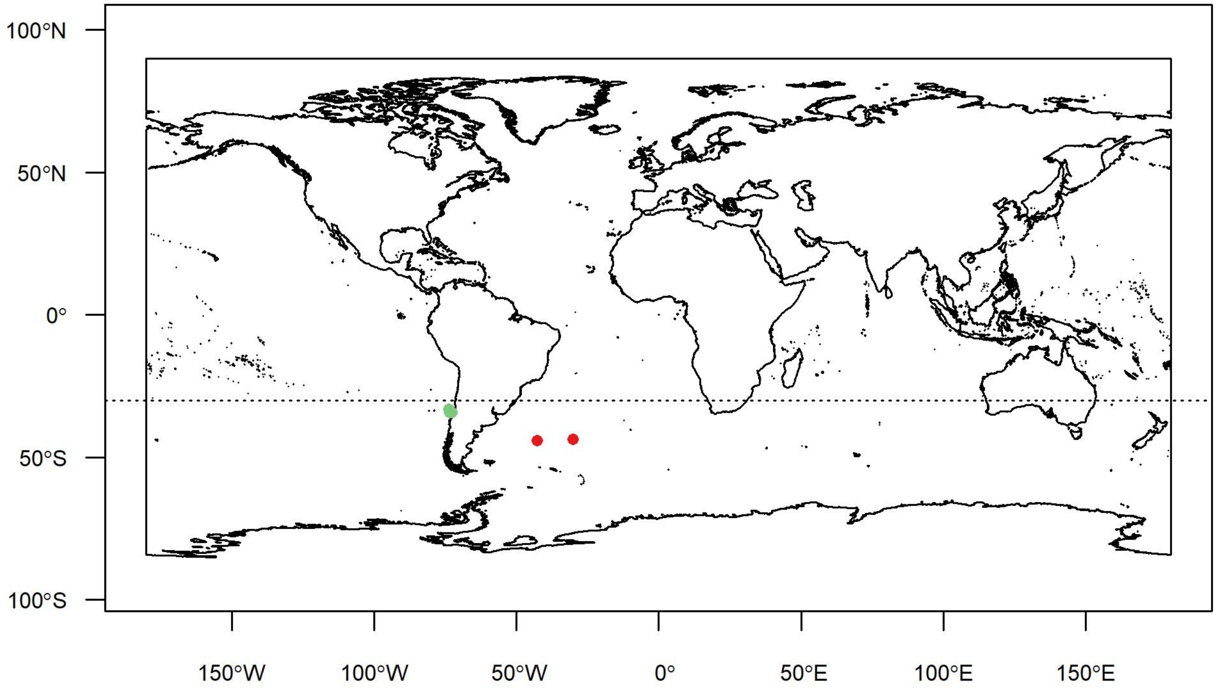

Information about the outliers :

	source	decimalLongitude	decimalLatitude	year	references
576210	OBIS	-30.0000	-43.7167	1969	
1721810	OBIS	-42.7167	-44.2333	1970	

Metelectrona herwigi

We found no outliers for this species.

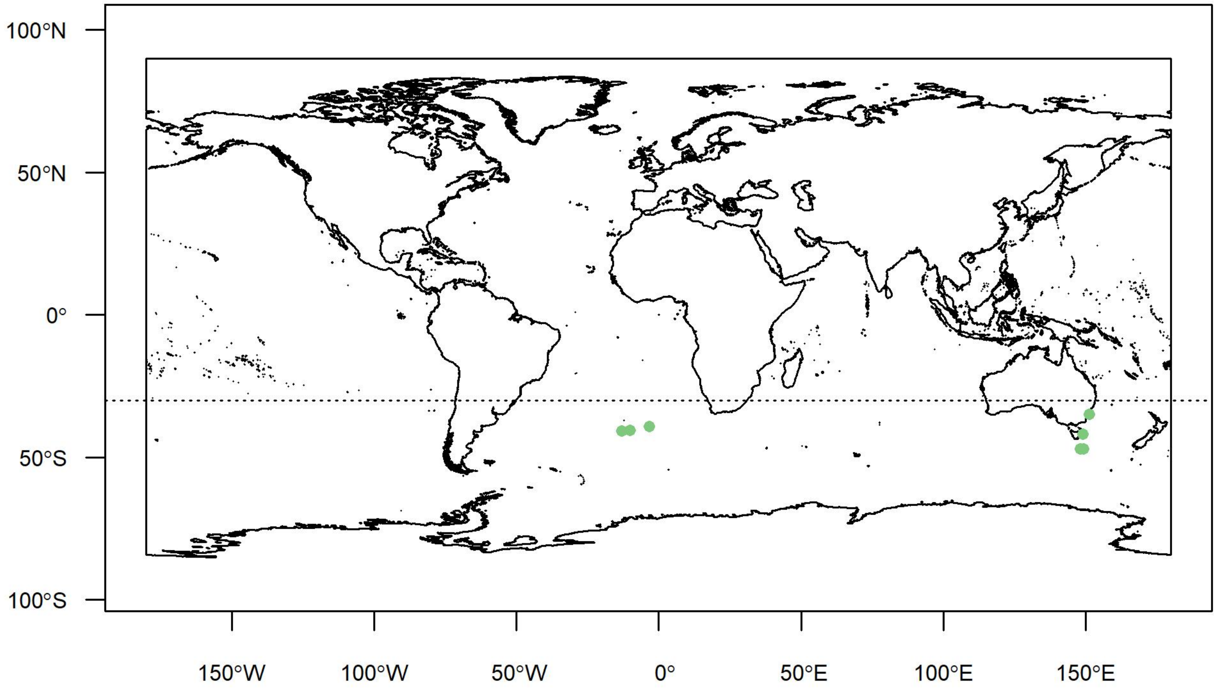

Metelectrona ventralis

We found two records with swapped coordinates from the Eltanin GBIF erroneous batch (blue points in map). We corrected their coordinates (purple points in map).

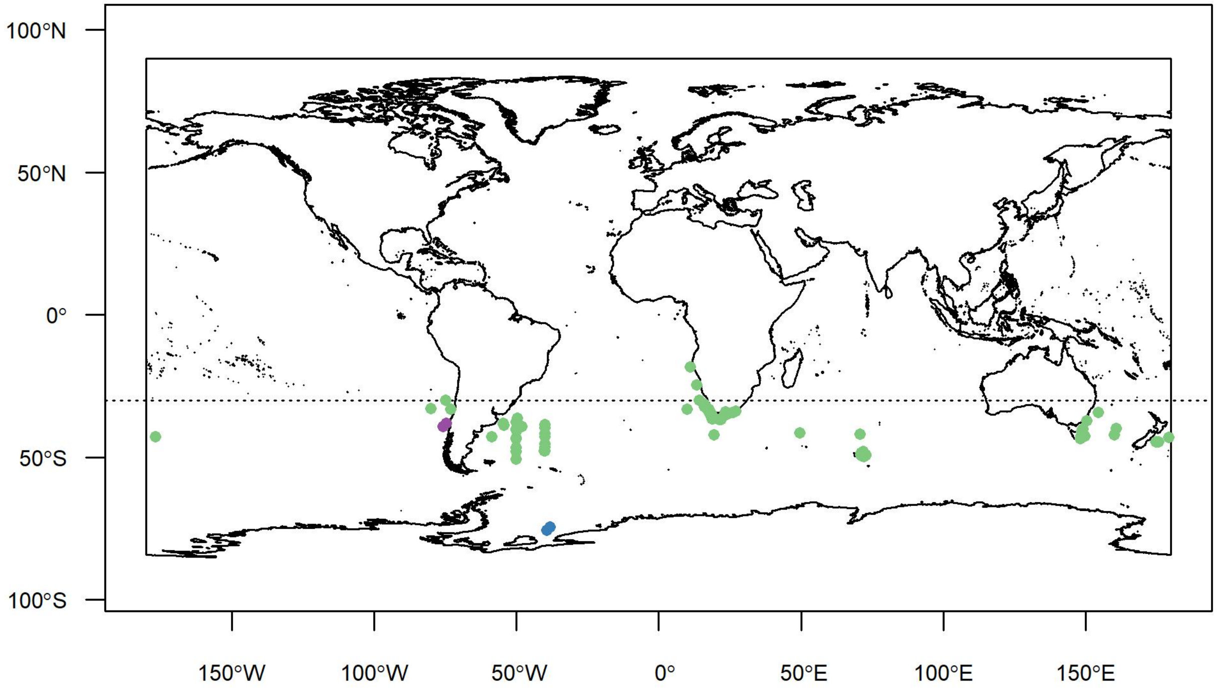

Information about Eltanin records with swapped coordinates are indicated here:

	source	year	references	bibliographicCitation
605701	GBIF	1962	http://portal.vertnet.org/o/lacm/fish?id=4d61faab-4a1a-4cba-8477-0f7ed4fd27a9	4D61FAAB-4A1A-4CBA-8477-0F7ED4FD27A9. http://ipt.vertnet.org:8080/ipt/resource.do?r=lacm_verts.
648681	GBIF	1964	http://portal.vertnet.org/o/lacm/fish?id=90233547-00f9-43d5-903c-26287e38a91f	90233547-00F9-43D5-903C-26287E38A91F. http://ipt.vertnet.org:8080/ipt/resource.do?r=lacm_verts.

Myctophum affine

According to (Namiki et al, 2015), this species is endemic to the Atlantic Ocean, so we removed the outliers in the other oceans (red points on the map).

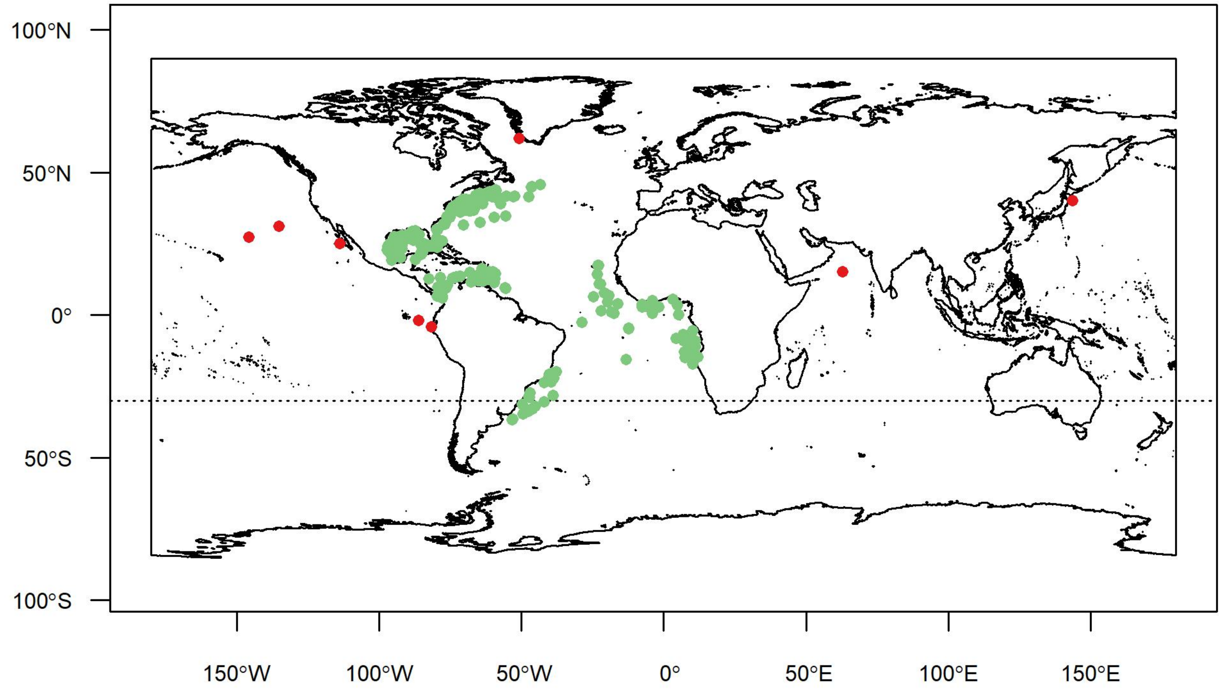

Information about the outliers :

	source	decimalLongitude	decimalLatitude	year	references
1826611	GBIF	-86.0000	-1.9167	1956	
193671	GBIF	143.5767	40.0717	1953	http://portal.vertnet.org/o/sio/marine-vertebrates?id=1fda0783-cce0-42d1-8eda-948816368336
576851	GBIF	-135.2167	31.1000	1966	http://portal.vertnet.org/o/lacm/fish?id=205f10be-63c6-4456-98e7-b683b00a3257

	source	decimalLongitude	decimalLatitude	year	references
608781	GBIF	-145.6333	27.2667	1966	http://portal.vertnet.org/o/lacm/fish?id=51e36d1f-b9db-45c8-ba09-dc92bb40f745
877521	GBIF	-113.6689	24.9172	1953	
138862	GBIF	63.0000	15.0000	1974	
147727	GBIF	-81.5943	-4.2480	1953	http://collections.peabody.yale.edu/search/Record/YPM-ICH-001840
169980	GBIF	-50.6333	61.7667	1955	

Myctophum asperum

We found no outliers for this species.

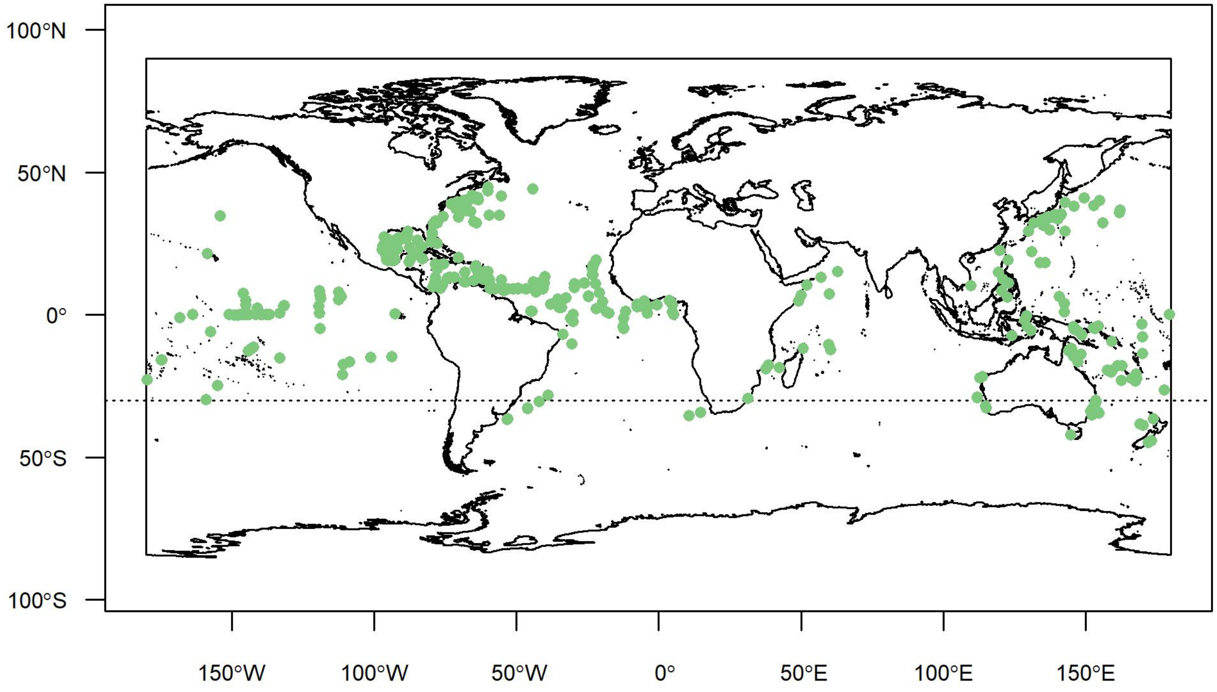

Myctophum nitidulum

We found no outliers for this species.

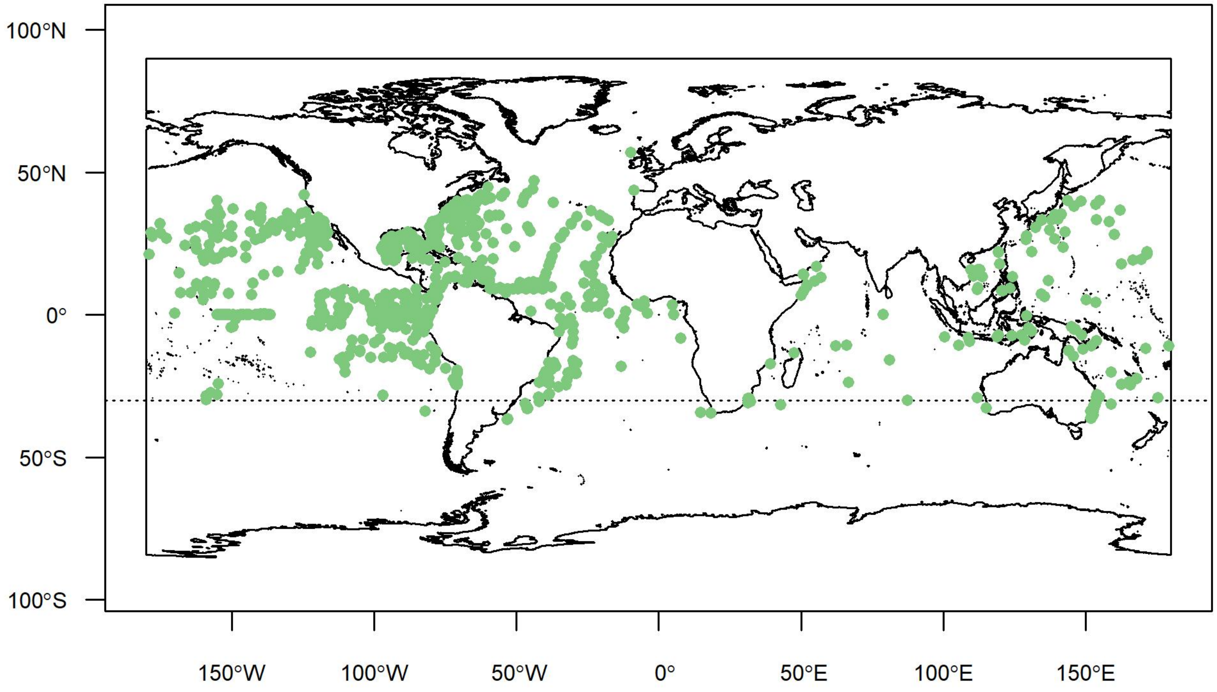

Myctophum obtusirostre

We found no outliers for this species.

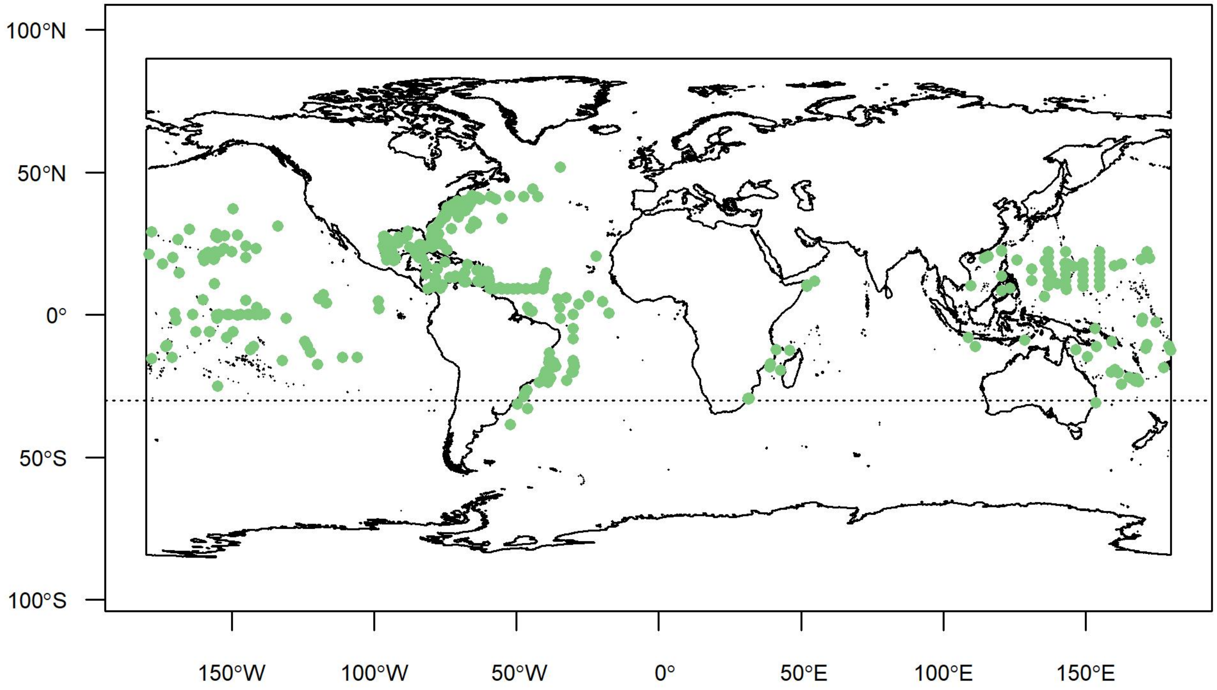

Myctophum phengodes

We found one record with swapped coordinates from the Eltanin GBIF erroneous batch (blue points in map). We corrected its coordinates (purple points in map).

We found one outlier close to Greenland (red point on the map), which we removed.

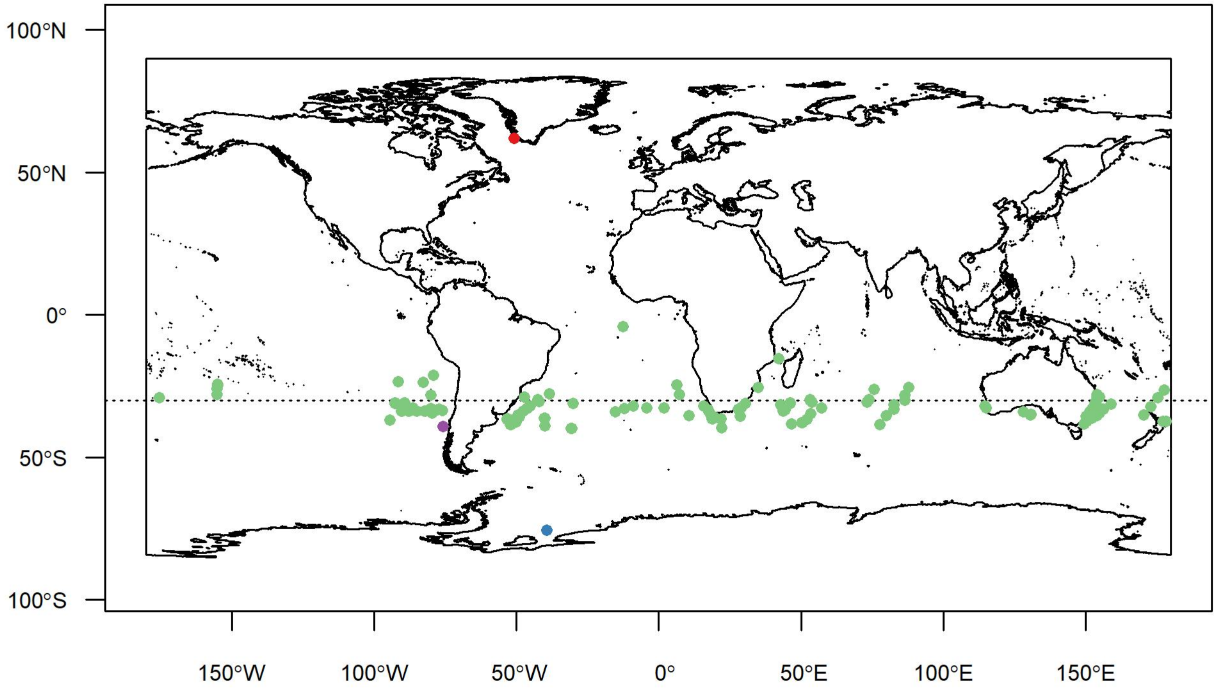

Information about Eltanin records with swapped coordinates are indicated here:

	source	year	references	bibliographicCitation
582681	GBIF	1964	http://portal.vertnet.org/o/lacm/fish?id=29a369d4-e685-4fda-817f-0cc3639f72e0	29A369D4-E685-4FDA-817F-0CC3639F72E0. http://ipt.vertnet.org:8080/ipt/resource.do?r=lacm_verts.

Information about the outlier :

	source	decimalLongitude	decimalLatitude	year	references
169992	GBIF	-50.6333	61.7667	1955	

Myctophum punctatum

We found multiple outliers in the Pacific and Atlantic Oceans (red points on the map), however this species is restricted to the North Atlantic, so we removed it from our dataset.

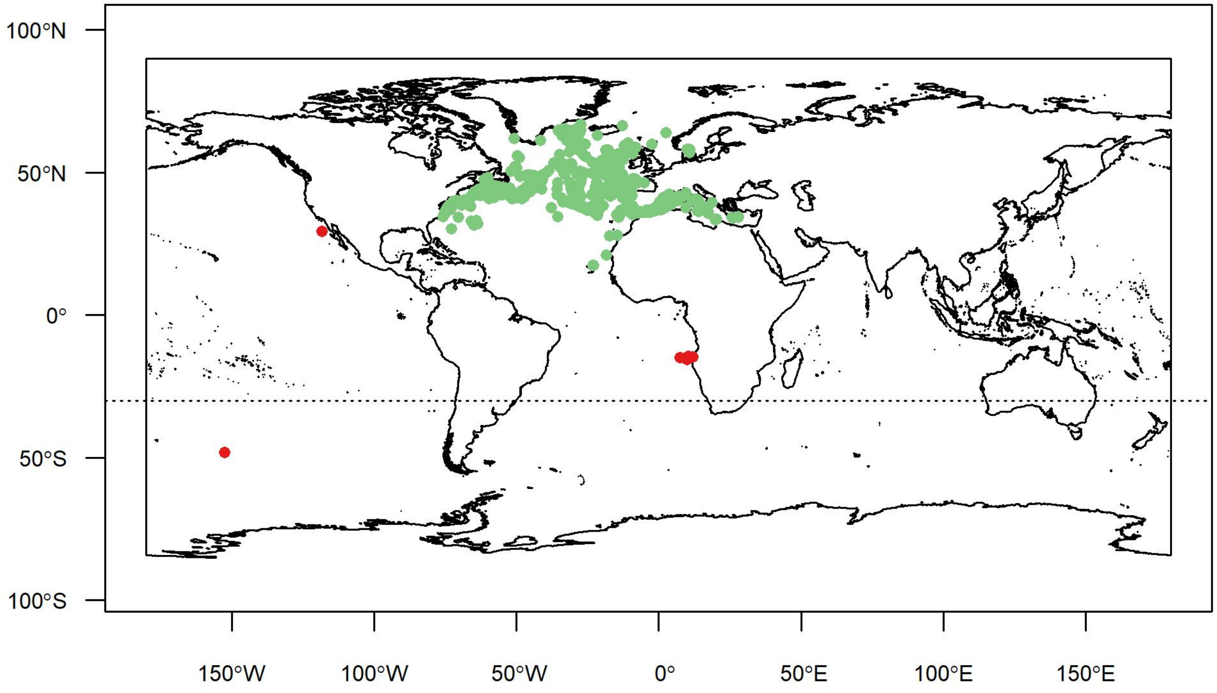

Information about the outliers :

	source	decimalLongitude	decimalLatitude	year	references
874010	OBIS	12.0000	-14.9170	1961	
31457	OBIS	10.5500	-14.5830	1961	
78169	OBIS	10.1170	-15.7830	1961	
104867	OBIS	7.6330	-15.0830	1961	
632141	GBIF	-118.2000	29.3403	1966	http://portal.vertnet.org/o/lacm/fish?id=76976517-946a-4672-b162-7e7dfbed8aff
724131	GBIF	-152.3667	-48.2667	1950	

Myctophum selenops

We found no outliers for this species.

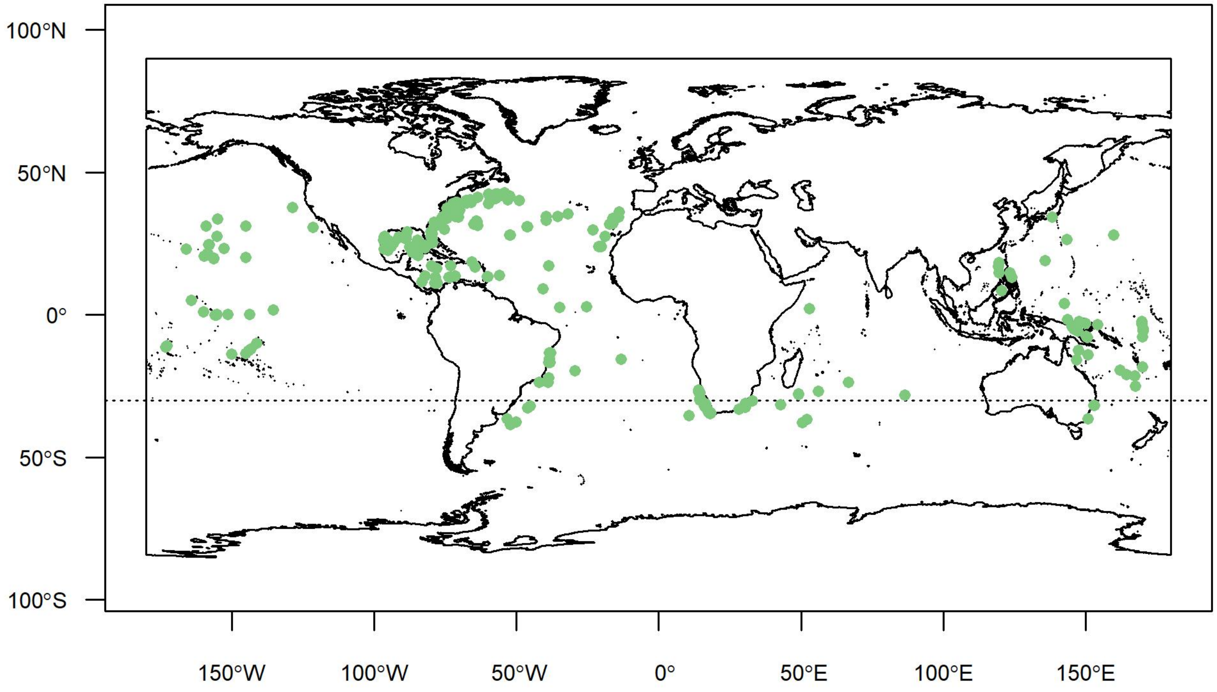

Myctophum spinosum

We found one outlier close to the Antarctic continent (red point on the map), which we removed.

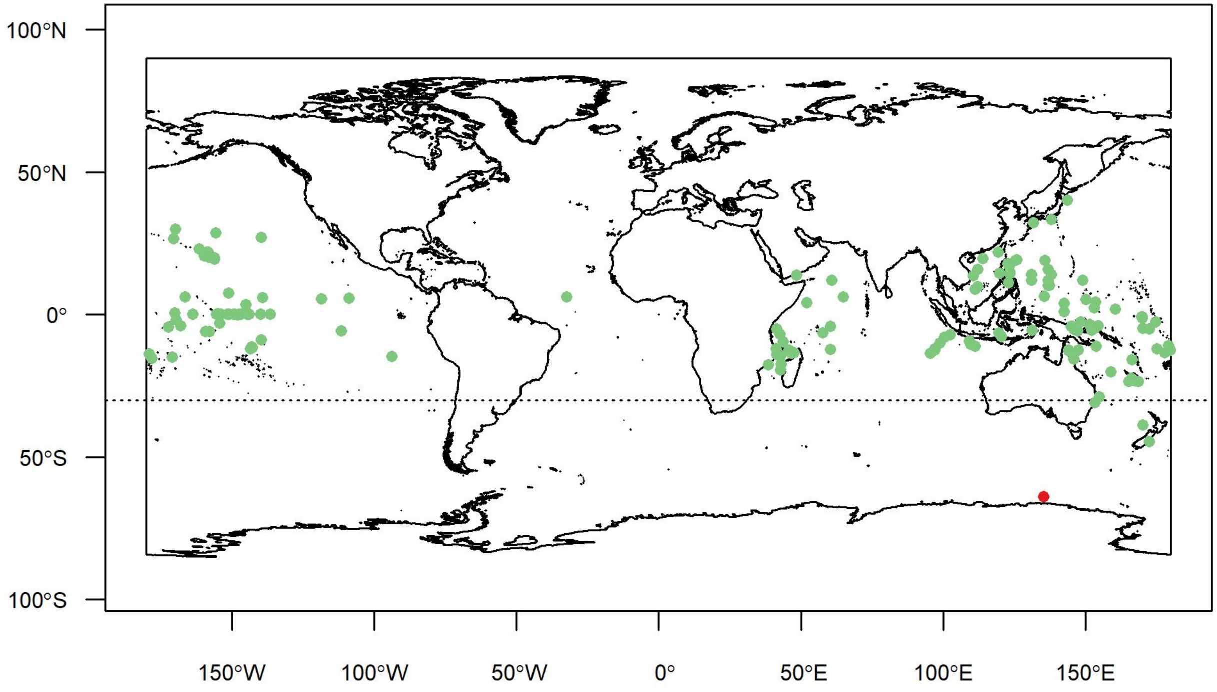

Information about the outlier :

	source	decimalLongitude	decimalLatitude	year	references
631811	GBIF	135.4301	-64.1305	1985	

Notolychnus valdiviae

We found no outliers for this species.

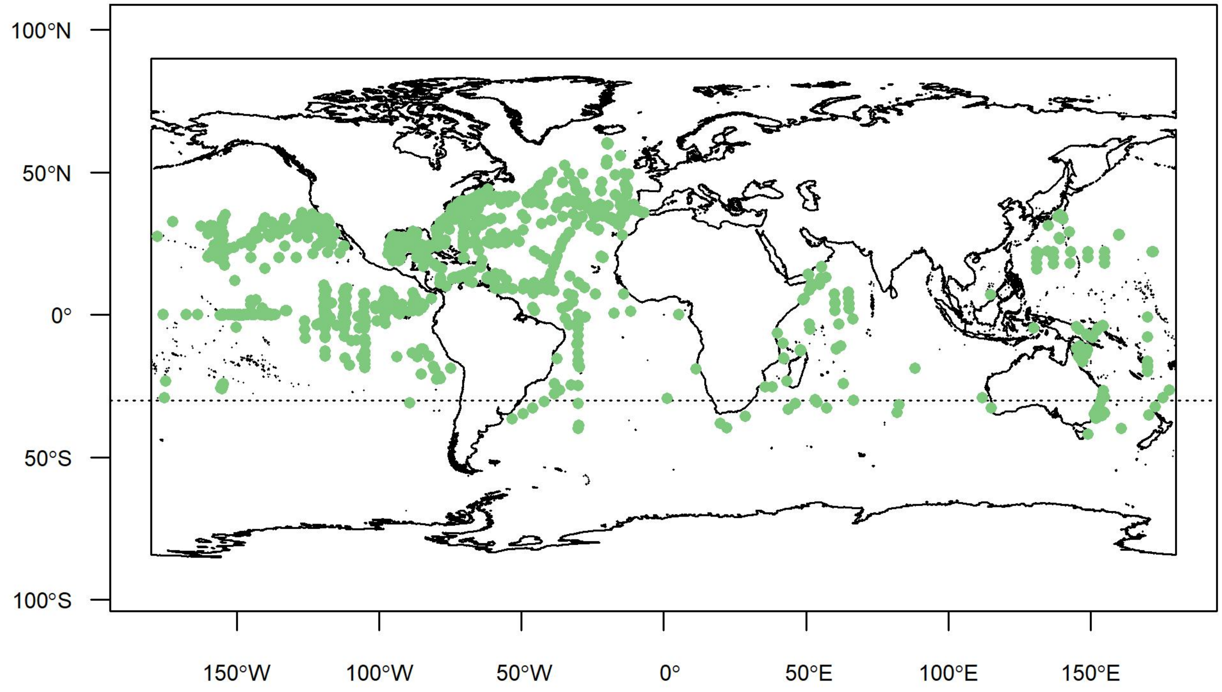

Notoscopelus caudispinosus

We found one potential outlier close to the Chilian coast (red point on the map), which we removed.

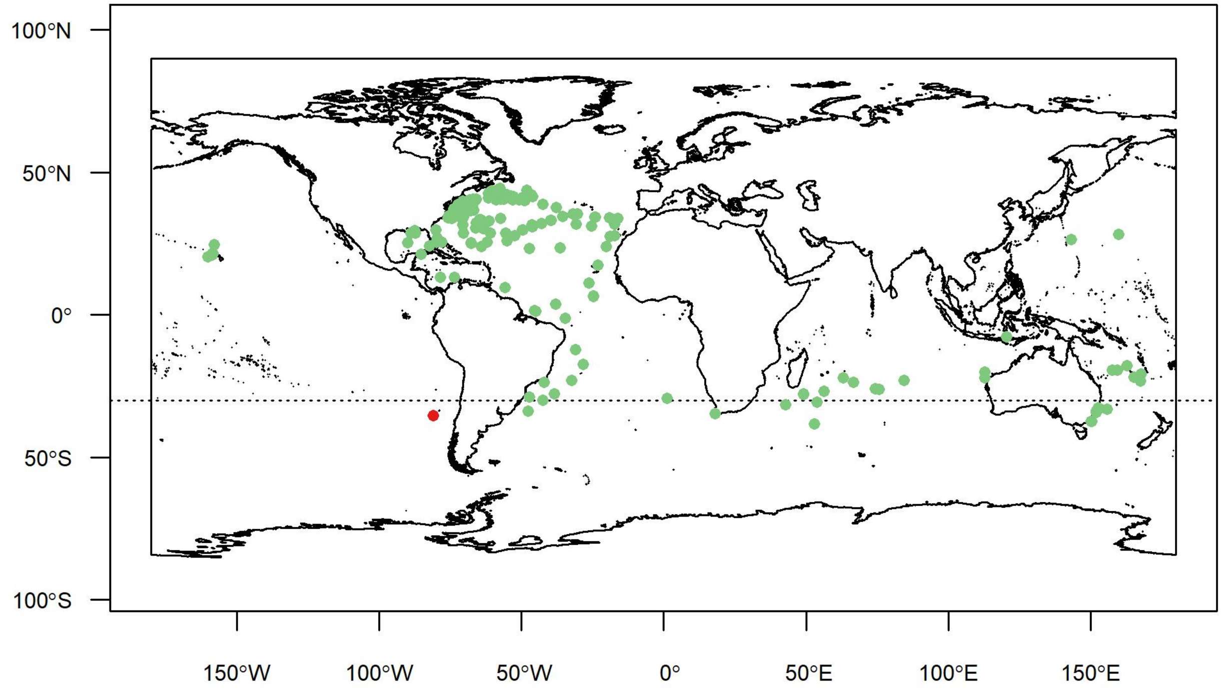

Information about the outlier :

	source	decimalLongitude	decimalLatitude	year	references
71998	OBIS	-80.9053	-35.4	1990	

Notoscopelus resplendens

We found one outlier in the Pacific Ocean (red point in map), which we removed.

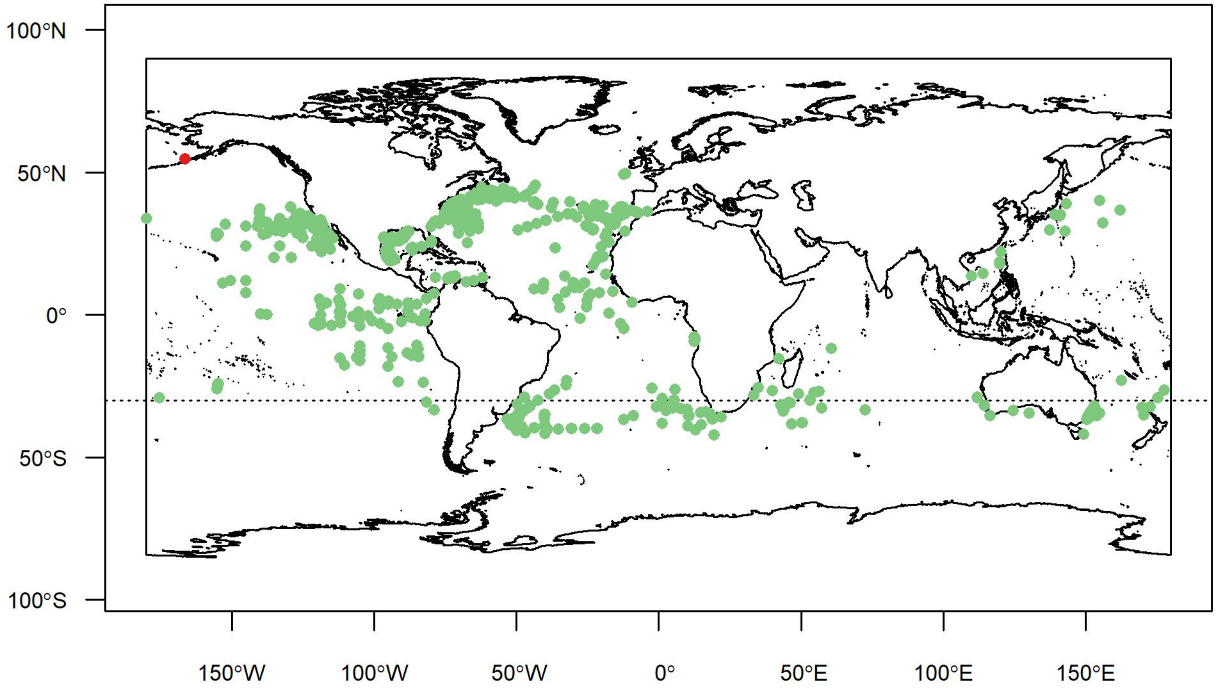

Information about the outliers :

	source	decimalLongitude	decimalLatitude	year	references
63205	OBIS	-166.4167	54.5667	1979	http://portal.vertnet.org/o/uwfc/adult-collection?id=uw-115417

Protomyctophum andriashevi

We found multiple records with swapped coordinates from the Eltanin GBIF erroneous batch (blue points in map). We corrected their coordinates (purple points in map).

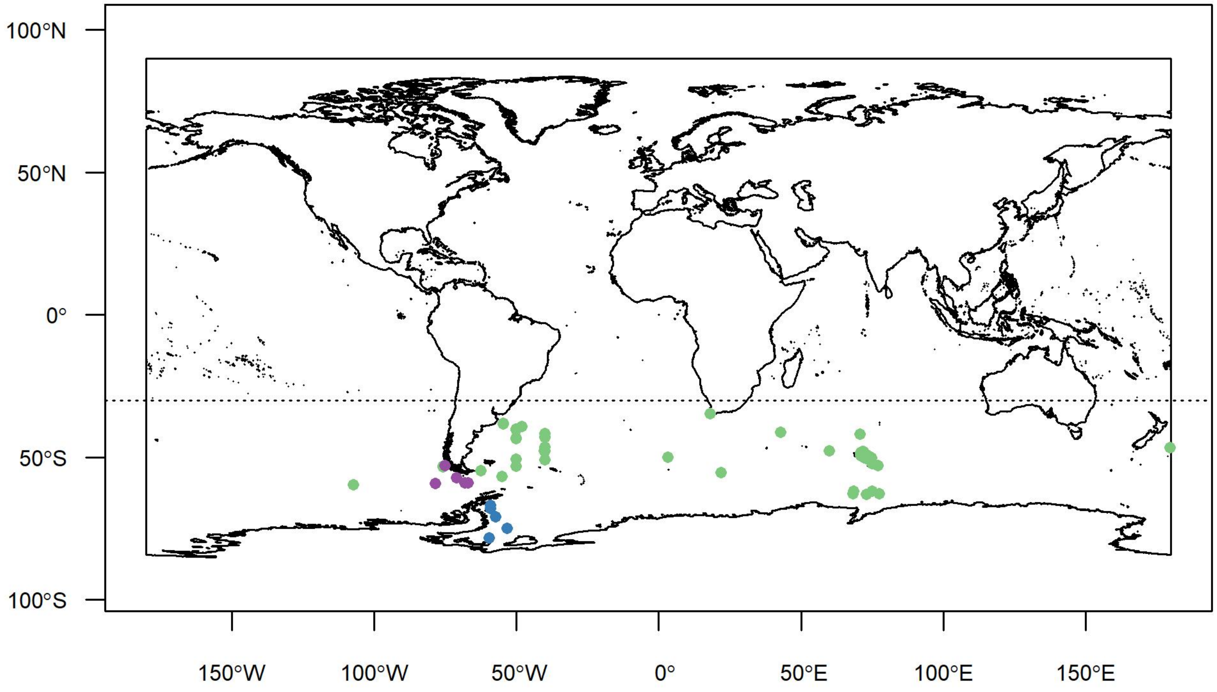

Information about Eltanin records with swapped coordinates are indicated here:

	source	year	references	bibliographicCitation
209111	GBIF	1964	http://portal.vertnet.org/o/lacm/fish?id=b4536fea-5f9c-4560-9a11-a47ac2053bf2	B4536FEA-5F9C-4560-9A11-A47AC2053BF2. http://ipt.vertnet.org:8080/ipt/resource.do?r=lacm_verts.
625071	GBIF	1962	http://portal.vertnet.org/o/lacm/fish?id=6b4de713-debf-40cb-8c30-16d3a8d60824	6B4DE713-DEBF-40CB-8C30-16D3A8D60824. http://ipt.vertnet.org:8080/ipt/resource.do?r=lacm_verts.
662612	GBIF	1963	http://portal.vertnet.org/o/lacm/fish?id=a66663d4-8fc7-4908-af12-f9788156e399	A66663D4-8FC7-4908-AF12-F9788156E399. http://ipt.vertnet.org:8080/ipt/resource.do?r=lacm_verts.

	source	year	references	bibliographicCitation
675771	GBIF	1962	http://portal.vertnet.org/o/lacm/fish?id=ba6c0672-1e75-4cd6-90d4-d8bf968b5f27	BA6C0672-1E75-4CD6-90D4-D8BF968B5F27. http://ipt.vertnet.org:8080/ipt/resource.do?r=lacm_verts.
687821	GBIF	1962	http://portal.vertnet.org/o/lacm/fish?id=cda2381a-b292-442e-a746-acacf4df5578	CDA2381A-B292-442E-A746-ACACF4DF5578. http://ipt.vertnet.org:8080/ipt/resource.do?r=lacm_verts.

Protomyctophum bolini

We found multiple records with swapped coordinates from the Eltanin GBIF erroneous batch (blue points in map). We corrected their coordinates (purple points in map).

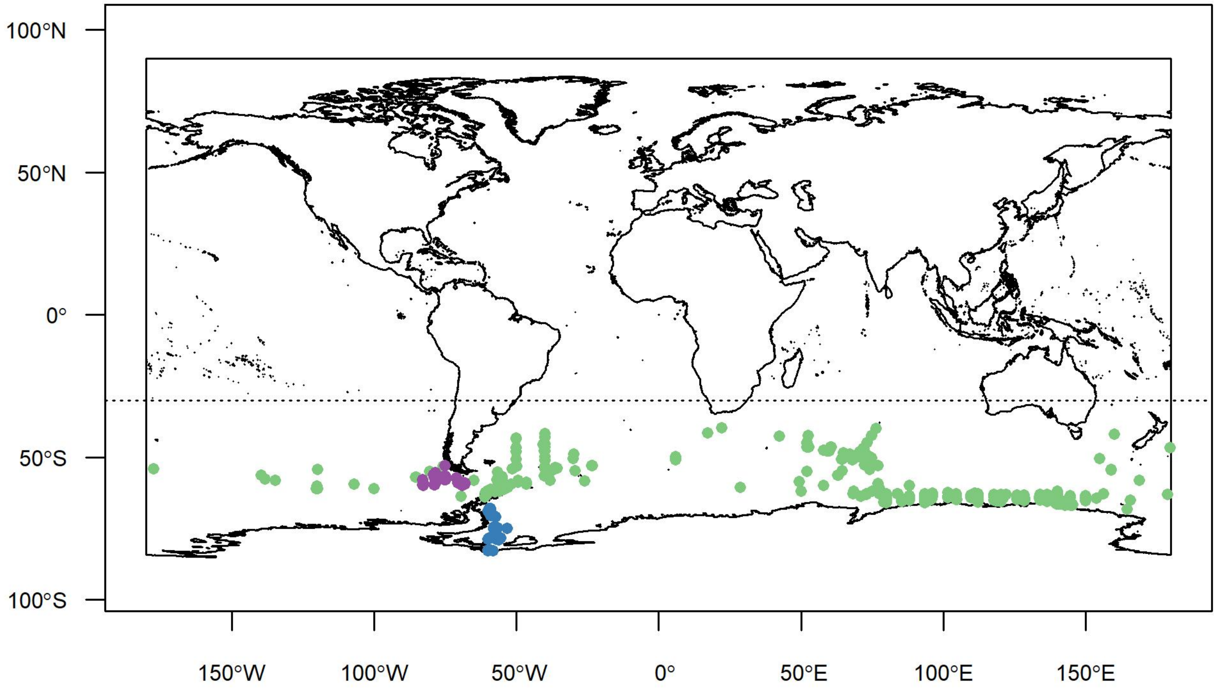

Information about Eltanin records with swapped coordinates are indicated here:

	source	year	references	bibliographicCitation
558361	GBIF	1963	http://portal.vertnet.org/o/lacm/fish?id=04786782-b988-45b5-945f-214903a66874	04786782-B988-45B5-945F-214903A66874. http://ipt.vertnet.org:8080/ipt/resource.do?r=lacm_verts.
558571	GBIF	1963	http://portal.vertnet.org/o/lacm/fish?id=04c02827-771a-4f1b-962a-48c3a0266998	04C02827-771A-4F1B-962A-48C3A0266998. http://ipt.vertnet.org:8080/ipt/resource.do?r=lacm_verts.
561551	GBIF	1963	http://portal.vertnet.org/o/lacm/fish?id=096b7c55-e058-41ef-8c49-9cf3b342ae3c	096B7C55-E058-41EF-8C49-9CF3B342AE3C. http://ipt.vertnet.org:8080/ipt/resource.do?r=lacm_verts.

	source	year	references	bibliographicCitation
575531	GBIF	1962	http://portal.vertnet.org/o/lacm/fish?id=1e939457-720e-4033-b890-13c2916c97da	1E939457-720E-4033-B890-13C2916C97DA. http://ipt.vertnet.org:8080/ipt/resource.do?r=lacm_verts.
586351	GBIF	1963	http://portal.vertnet.org/o/lacm/fish?id=2f80106e-8469-4492-b074-6076974c03f9	2F80106E-8469-4492-B074-6076974C03F9. http://ipt.vertnet.org:8080/ipt/resource.do?r=lacm_verts.
611212	GBIF	1962	http://portal.vertnet.org/o/lacm/fish?id=5578eb8e-0da7-477a-8d79-60d61891cd8c	5578EB8E-0DA7-477A-8D79-60D61891CD8C. http://ipt.vertnet.org:8080/ipt/resource.do?r=lacm_verts.
614581	GBIF	1963	http://portal.vertnet.org/o/lacm/fish?id=5ac33ea7-54ae-4d0f-920e-8f542be8628f	5AC33EA7-54AE-4D0F-920E-8F542BE8628F. http://ipt.vertnet.org:8080/ipt/resource.do?r=lacm_verts.
626191	GBIF	1964	http://portal.vertnet.org/o/lacm/fish?id=6d70ed2e-769c-438a-8911-dc95f794de2a	6D70ED2E-769C-438A-8911-DC95F794DE2A. http://ipt.vertnet.org:8080/ipt/resource.do?r=lacm_verts.
628651	GBIF	1962	http://portal.vertnet.org/o/lacm/fish?id=7176ec18-dcea-45b0-9be5-8fe1b3d91677	7176EC18-DCEA-45B0-9BE5-8FE1B3D91677. http://ipt.vertnet.org:8080/ipt/resource.do?r=lacm_verts.
631261	GBIF	1963	http://portal.vertnet.org/o/lacm/fish?id=754280aa-61fa-4ca1-aa00-90f939f7a51d	754280AA-61FA-4CA1-AA00-90F939F7A51D. http://ipt.vertnet.org:8080/ipt/resource.do?r=lacm_verts.
657081	GBIF	1963	http://portal.vertnet.org/o/lacm/fish?id=9d8967aa-8b48-467e-ba84-4e6e3c3d64c8	9D8967AA-8B48-467E-BA84-4E6E3C3D64C8. http://ipt.vertnet.org:8080/ipt/resource.do?r=lacm_verts.
663921	GBIF	1962	http://portal.vertnet.org/o/lacm/fish?id=a8a56e55-1363-46c6-95b8-43b31d89943c	A8A56E55-1363-46C6-95B8-43B31D89943C. http://ipt.vertnet.org:8080/ipt/resource.do?r=lacm_verts.
664641	GBIF	1963	http://portal.vertnet.org/o/lacm/fish?id=a9b08c2c-f4dc-4426-910c-612337d8dd1c	A9B08C2C-F4DC-4426-910C-612337D8DD1C. http://ipt.vertnet.org:8080/ipt/resource.do?r=lacm_verts.
678561	GBIF	1963	http://portal.vertnet.org/o/lacm/fish?id=bee826b1-a64d-410a-b715-46625d9d3f82	BEE826B1-A64D-410A-B715-46625D9D3F82. http://ipt.vertnet.org:8080/ipt/resource.do?r=lacm_verts.
682481	GBIF	1963	http://portal.vertnet.org/o/lacm/fish?id=c51931d4-60fb-491b-a2ea-fad639d83fbd	C51931D4-60FB-491B-A2EA-FAD639D83FBD. http://ipt.vertnet.org:8080/ipt/resource.do?r=lacm_verts.
682881	GBIF	1962	http://portal.vertnet.org/o/lacm/fish?id=c5c09229-0276-482e-9052-8c6ae4dc2c1d	C5C09229-0276-482E-9052-8C6AE4DC2C1D. http://ipt.vertnet.org:8080/ipt/resource.do?r=lacm_verts.
683191	GBIF	1963	http://portal.vertnet.org/o/lacm/fish?id=c5db000a-5a78-4797-b5b3-0f6442e46df8	C5DB000A-5A78-4797-B5B3-0F6442E46DF8. http://ipt.vertnet.org:8080/ipt/resource.do?r=lacm_verts.
684951	GBIF	1963	http://portal.vertnet.org/o/lacm/fish?id=c916d15c-95e3-4d0f-a89d-4de2f0596e12	C916D15C-95E3-4D0F-A89D-4DE2F0596E12. http://ipt.vertnet.org:8080/ipt/resource.do?r=lacm_verts.
688712	GBIF	1963	http://portal.vertnet.org/o/lacm/fish?id=ced5d998-326f-4440-b293-b4311f876fa5	CED5D998-326F-4440-B293-B4311F876FA5. http://ipt.vertnet.org:8080/ipt/resource.do?r=lacm_verts.
691561	GBIF	1962	http://portal.vertnet.org/o/lacm/fish?id=d3795b24-65f2-40c1-bc3f-dd9317898986	D3795B24-65F2-40C1-BC3F-DD9317898986. http://ipt.vertnet.org:8080/ipt/resource.do?r=lacm_verts.
704112	GBIF	1962	http://portal.vertnet.org/o/lacm/fish?id=e6dbe545-3ae3-4ad1-be12-8d387890aef5	E6DBE545-3AE3-4AD1-BE12-8D387890AEF5. http://ipt.vertnet.org:8080/ipt/resource.do?r=lacm_verts.
704291	GBIF	1963	http://portal.vertnet.org/o/lacm/fish?id=e72342da-c9bb-46d4-bb24-cdf2d7525d4b	E72342DA-C9BB-46D4-BB24-CDF2D7525D4B. http://ipt.vertnet.org:8080/ipt/resource.do?r=lacm_verts.

709931	GBIF	1963	http://portal.vertnet.org/o/lacm/fish?id=efacb3b1-641d-476d-99c1-097ef6fb4550	EFACB3B1-641D-476D-99C1-097EF6FB4550. http://ipt.vertnet.org:8080/ipt/resource.do?r=lacm_verts.

Protomyctophum chilense

We found no outliers for this species.

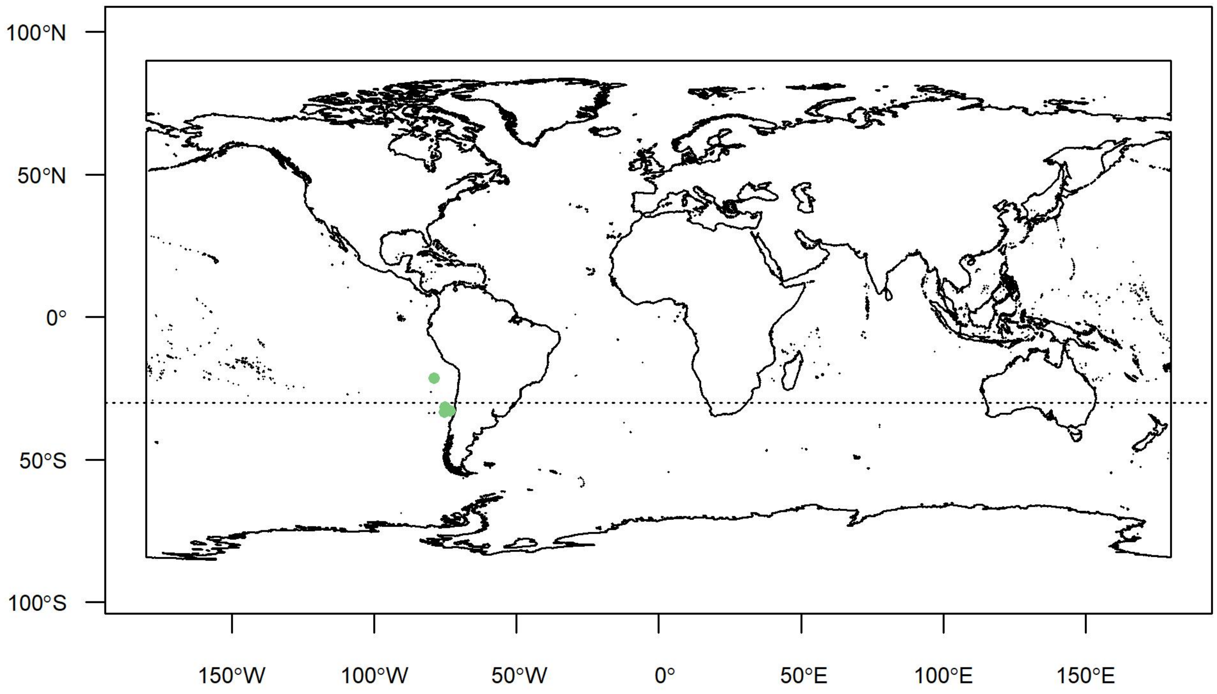

Protomyctophum choriodon

We found no outliers for this species.

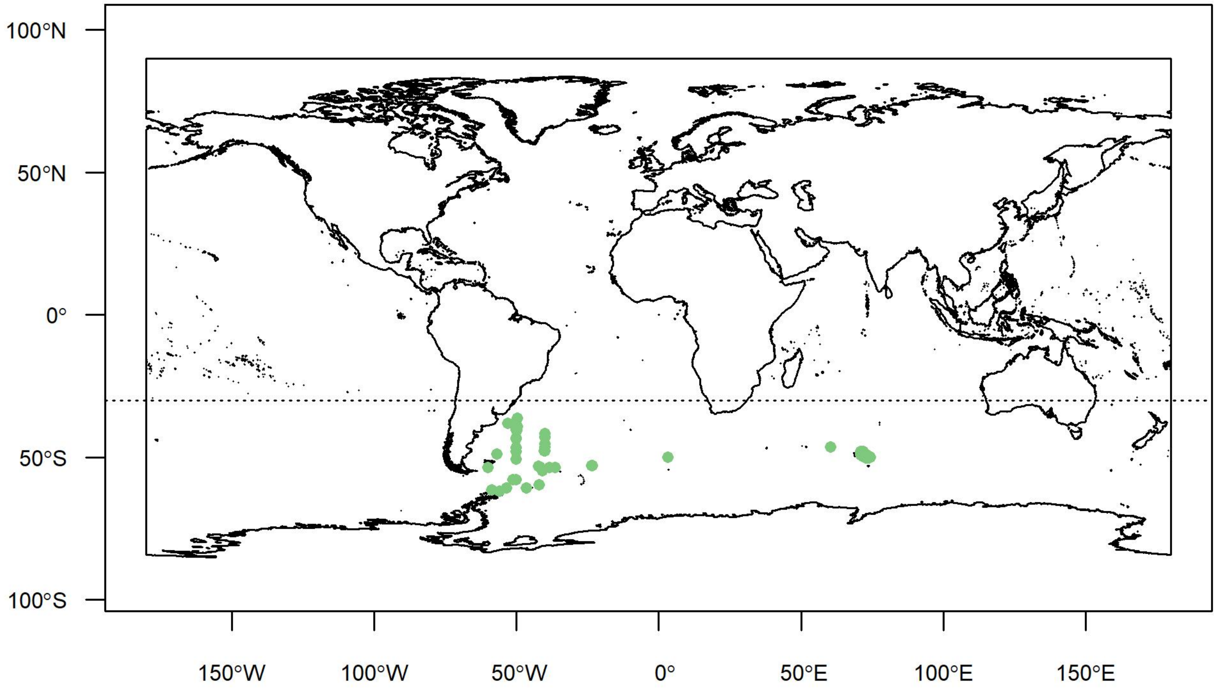

Protomyctophum gemmatum

We found no outliers for this species.

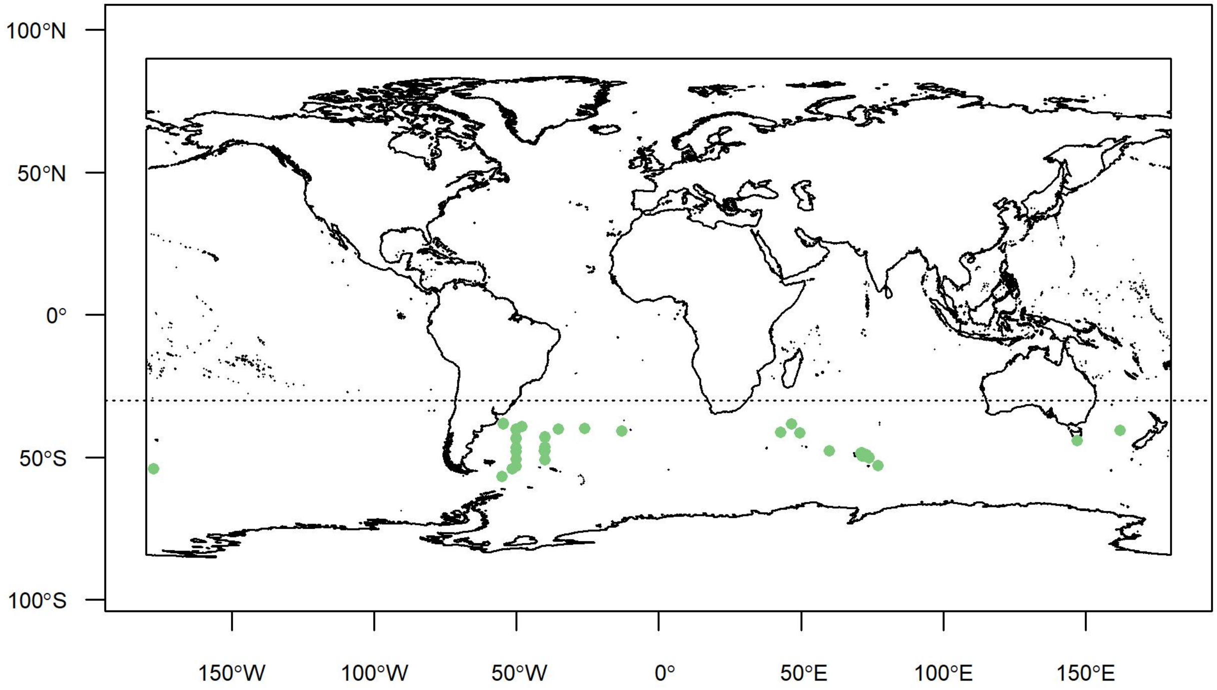

Protomyctophum luciferum

We found no outliers for this species.

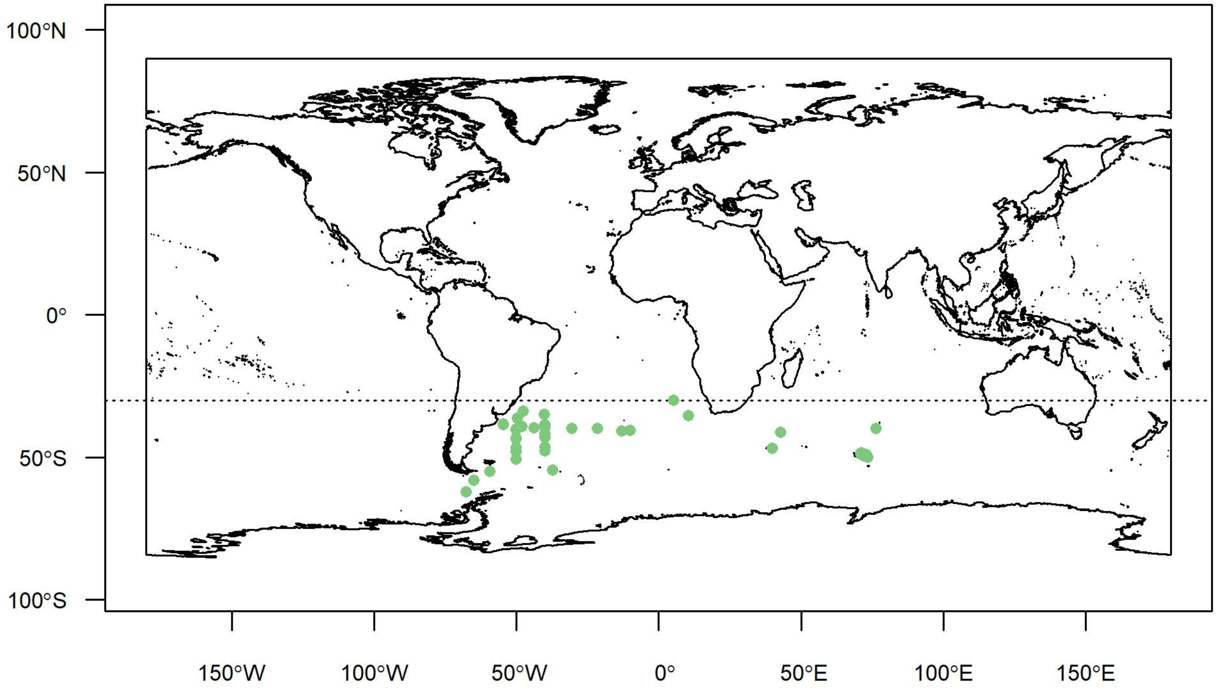

Protomyctophum normani

We found two outliers in the Northern Hemisphere (red points on the map), which we removed.

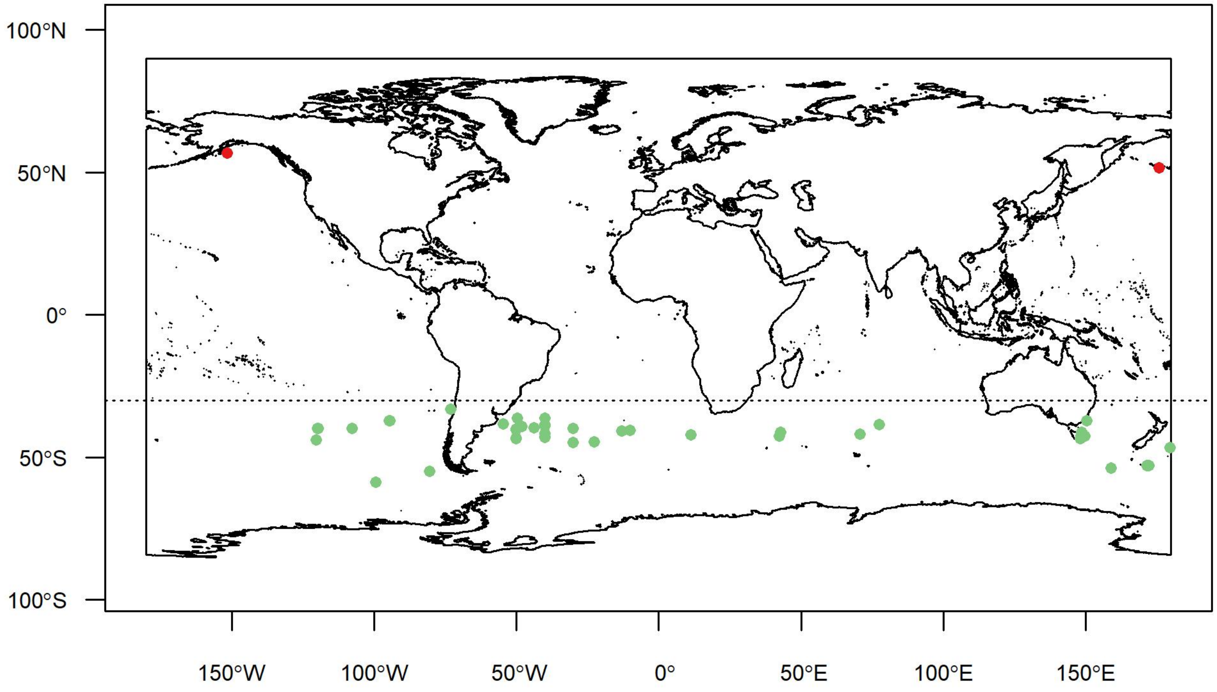

Information about the outliers :

	source	decimalLongitude	decimalLatitude	year	references
294712	GBIF	175.7667	51.45	1957	http://portal.vertnet.org/o/sio/marine-vertebrates?id=54e14fdc-e9cc-49fa-8b12-d481bd7c7a4f
294741	GBIF	-151.5333	56.75	1957	http://portal.vertnet.org/o/sio/marine-vertebrates?id=efd680f6-f735-47b6-9a31-51a549d97993

Protomyctophum parallelum

We found multiple records with swapped coordinates from the Eltanin GBIF erroneous batch (blue points in map). We corrected their coordinates (purple points in map).

There was one outlier in the Atlantic Ocean (red point in map), which we removed.

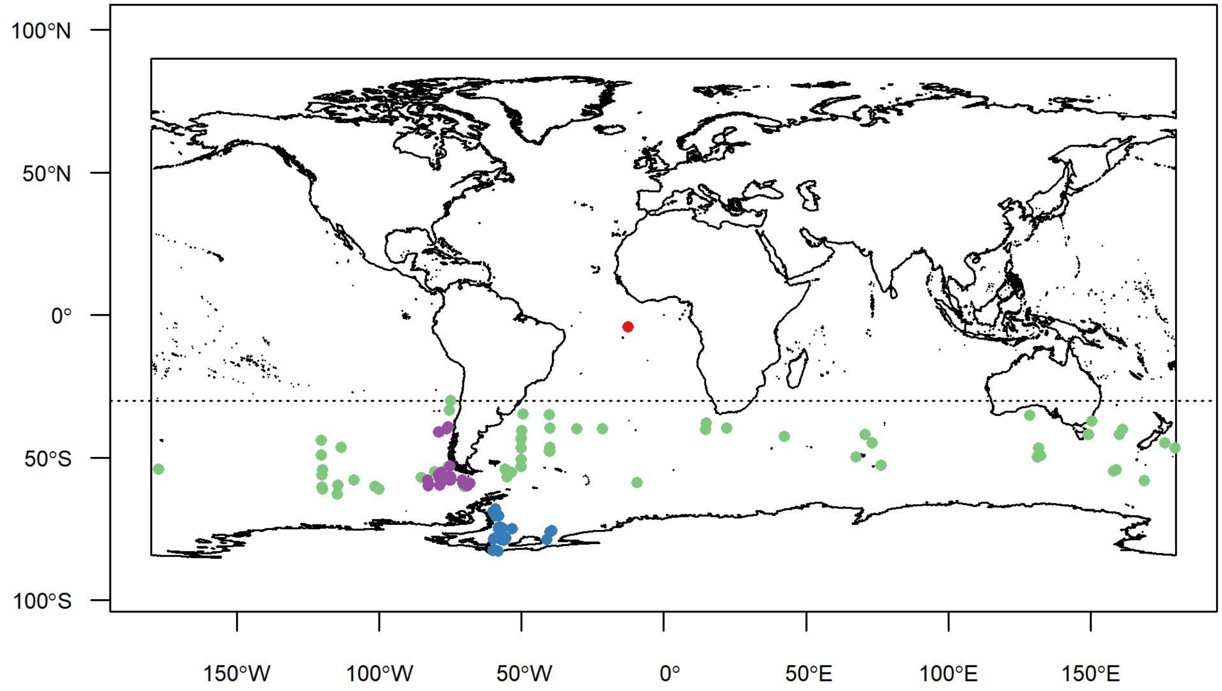

Information about Eltanin records with swapped coordinates are indicated here:

	source	year	references	bibliographicCitation
561681	GBIF	1966	http://portal.vertnet.org/o/lacm/fish?id=098969c9-a90a-40cf-aa20-743c698bc1c2	098969C9-A90A-40CF-AA20-743C698BC1C2. http://ipt.vertnet.org:8080/ipt/resource.do?r=lacm_verts.
571631	GBIF	1962	http://portal.vertnet.org/o/lacm/fish?id=184a960a-5ee0-4617-90b5-b8508f5f389a	184A960A-5EE0-4617-90B5-B8508F5F389A. http://ipt.vertnet.org:8080/ipt/resource.do?r=lacm_verts.
574912	GBIF	1963	http://portal.vertnet.org/o/lacm/fish?id=1d97df5f-b990-4a14-9f79-780e648828c0	1D97DF5F-B990-4A14-9F79-780E648828C0. http://ipt.vertnet.org:8080/ipt/resource.do?r=lacm_verts.
579412	GBIF	1962	http://portal.vertnet.org/o/lacm/fish?id=245df05c-468b-439f-aa4b-3bbdb4b508fb	245DF05C-468B-439F-AA4B-3BBDB4B508FB. http://ipt.vertnet.org:8080/ipt/resource.do?r=lacm_verts.
588151	GBIF	1963	http://portal.vertnet.org/o/lacm/fish?id=32554e9b-82b2-4a40-9ebd-6dd86c7145a4	32554E9B-82B2-4A40-9EBD-6DD86C7145A4. http://ipt.vertnet.org:8080/ipt/resource.do?r=lacm_verts.
596681	GBIF	1964	http://portal.vertnet.org/o/lacm/fish?id=4029a5b2-b673-4b30-97db-227599984ae3	4029A5B2-B673-4B30-97DB-227599984AE3. http://ipt.vertnet.org:8080/ipt/resource.do?r=lacm_verts.
602561	GBIF	1964	http://portal.vertnet.org/o/lacm/fish?id=48beb805-8208-4910-a5f1-14ff2e2353bf	48BEB805-8208-4910-A5F1-14FF2E2353BF. http://ipt.vertnet.org:8080/ipt/resource.do?r=lacm_verts.
611331	GBIF	1963	http://portal.vertnet.org/o/lacm/fish?id=55bff805-dda2-4fa0-9627-ae9a53d0bb57	55BFF805-DDA2-4FA0-9627-AE9A53D0BB57. http://ipt.vertnet.org:8080/ipt/resource.do?r=lacm_verts.
612412	GBIF	1963	http://portal.vertnet.org/o/lacm/fish?id=57969e1f-bcf6-4963-bed9-cc173851160c	57969E1F-BCF6-4963-BED9-CC173851160C. http://ipt.vertnet.org:8080/ipt/resource.do?r=lacm_verts.
615491	GBIF	1962	http://portal.vertnet.org/o/lacm/fish?id=5c43c5d7-58de-4b92-b964-0e761470a1bd	5C43C5D7-58DE-4B92-B964-0E761470A1BD. http://ipt.vertnet.org:8080/ipt/resource.do?r=lacm_verts.
616401	GBIF	1963	http://portal.vertnet.org/o/lacm/fish?id=5df330b8-9d3a-4b93-b2da-0c9def55a2f9	5DF330B8-9D3A-4B93-B2DA-0C9DEF55A2F9. http://ipt.vertnet.org:8080/ipt/resource.do?r=lacm_verts.
617571	GBIF	1963	http://portal.vertnet.org/o/lacm/fish?id=5fe59ed9-f7e1-404e-a41a-7ff0980d731c	5FE59ED9-F7E1-404E-A41A-7FF0980D731C. http://ipt.vertnet.org:8080/ipt/resource.do?r=lacm_verts.
619291	GBIF	1964	http://portal.vertnet.org/o/lacm/fish?id=62b01f57-2ce1-4c36-a65c-882b25a75d6f	62B01F57-2CE1-4C36-A65C-882B25A75D6F. http://ipt.vertnet.org:8080/ipt/resource.do?r=lacm_verts.
621331	GBIF	1962	http://portal.vertnet.org/o/lacm/fish?id=661fe53b-cad4-4c15-9502-391e969ca7ef	661FE53B-CAD4-4C15-9502-391E969CA7EF. http://ipt.vertnet.org:8080/ipt/resource.do?r=lacm_verts.
629341	GBIF	1963	http://portal.vertnet.org/o/lacm/fish?id=720a4fc3-5996-4345-a47a-326c271c9e72	720A4FC3-5996-4345-A47A-326C271C9E72. http://ipt.vertnet.org:8080/ipt/resource.do?r=lacm_verts.
633901	GBIF	1963	http://portal.vertnet.org/o/lacm/fish?id=79a8a5f1-407f-4684-bf4a-632187059c07	79A8A5F1-407F-4684-BF4A-632187059C07. http://ipt.vertnet.org:8080/ipt/resource.do?r=lacm_verts.
649131	GBIF	1963	http://portal.vertnet.org/o/lacm/fish?id=90efef7c-dbfc-421e-a0dd-580e15dbafe3	90EFEF7C-DBFC-421E-A0DD-580E15DBAFE3. http://ipt.vertnet.org:8080/ipt/resource.do?r=lacm_verts.
649891	GBIF	1963	http://portal.vertnet.org/o/lacm/fish?id=91b0fe3d-c337-45eb-82b7-00aaf599763c	91B0FE3D-C337-45EB-82B7-00AAF599763C. http://ipt.vertnet.org:8080/ipt/resource.do?r=lacm_verts.
669612	GBIF	1963	http://portal.vertnet.org/o/lacm/fish?id=b19d4969-9d21-4819-ad7d-8ea4318c43de	B19D4969-9D21-4819-AD7D-8EA4318C43DE. http://ipt.vertnet.org:8080/ipt/resource.do?r=lacm_verts.
676791	GBIF	1962	http://portal.vertnet.org/o/lacm/fish?id=bbf58d70-e2aa-4644-89f4-d0b122baabbf	BBF58D70-E2AA-4644-89F4-D0B122BAABBF. http://ipt.vertnet.org:8080/ipt/resource.do?r=lacm_verts.

	source	year	references	bibliographicCitation
688131	GBIF	1966	http://portal.vertnet.org/o/lacm/fish?id=ce6af893-ffd5-47bb-b89a-732f806d896a	CE6AF893-FFD5-47BB-B89A-732F806D896A. http://ipt.vertnet.org:8080/ipt/resource.do?r=lacm_verts.
696381	GBIF	1962	http://portal.vertnet.org/o/lacm/fish?id=dae42d86-5a91-441b-81d5-e4a2b1edcf0a	DAE42D86-5A91-441B-81D5-E4A2B1EDCF0A. http://ipt.vertnet.org:8080/ipt/resource.do?r=lacm_verts.
700191	GBIF	1963	http://portal.vertnet.org/o/lacm/fish?id=e09eca82-8d23-4fb6-9039-a371107c713d	E09ECA82-8D23-4FB6-9039-A371107C713D. http://ipt.vertnet.org:8080/ipt/resource.do?r=lacm_verts.
705021	GBIF	1963	http://portal.vertnet.org/o/lacm/fish?id=e84bed4f-170d-4404-9893-96f3b00c1ded	E84BED4F-170D-4404-9893-96F3B00C1DED. http://ipt.vertnet.org:8080/ipt/resource.do?r=lacm_verts.
706061	GBIF	1963	http://portal.vertnet.org/o/lacm/fish?id=e9b66646-356f-4c3a-a654-dc0cf453ed08	E9B66646-356F-4C3A-A654-DC0CF453ED08. http://ipt.vertnet.org:8080/ipt/resource.do?r=lacm_verts.

Information about the outlier :

	source	decimalLongitude	decimalLatitude	year	references
845110	OBIS	-12.405	-4.1578	2009	

Protomyctophum subparallelum

We found multiple records with swapped coordinates from the Eltanin GBIF erroneous batch (blue points in map). We corrected their coordinates (purple points in map).

We found one outlier in the Northern Hemisphere (red point on the map), which we removed.

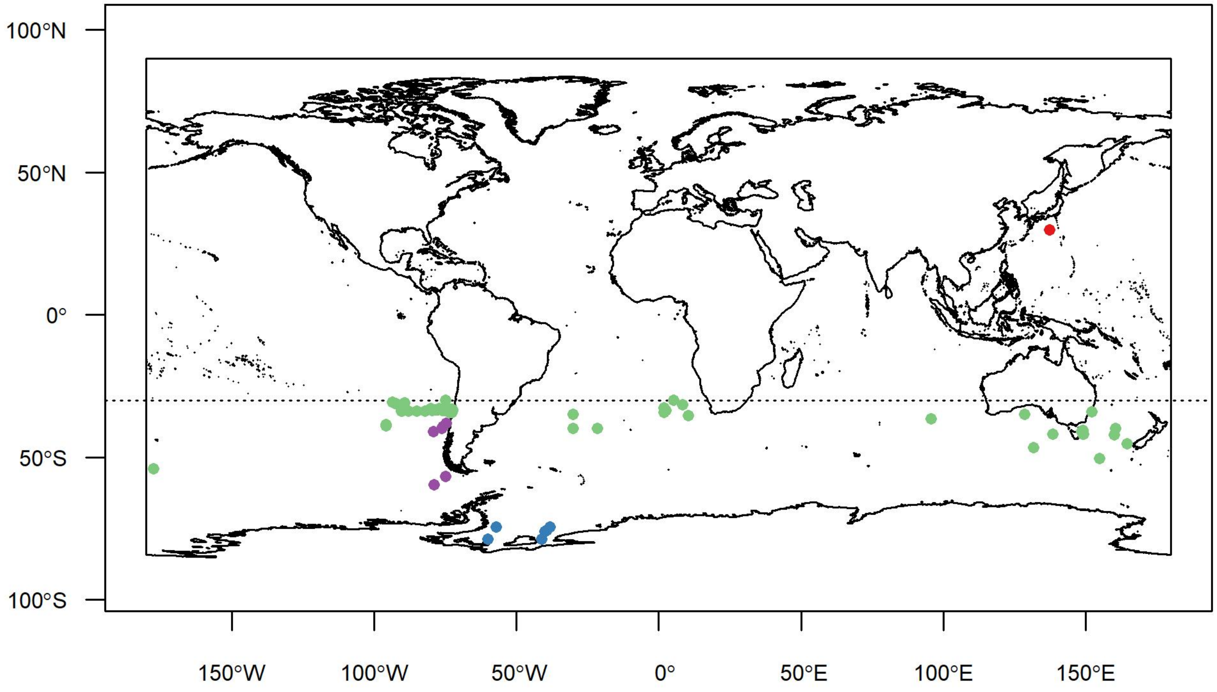

Information about Eltanin records with swapped coordinates are indicated here:

	source	year	references	bibliographicCitation
568721	GBIF	1963	http://portal.vertnet.org/o/lacm/fish?id=147acb0d-6370-4a8e-9b2e-73f32eb98383	147ACB0D-6370-4A8E-9B2E-73F32EB98383. http://ipt.vertnet.org:8080/ipt/resource.do?r=lacm_verts.
589681	GBIF	1962	http://portal.vertnet.org/o/lacm/fish?id=34a24bb4-bddd-4fc3-a24a-86ab21e098c4	34A24BB4-BDDD-4FC3-A24A-86AB21E098C4. http://ipt.vertnet.org:8080/ipt/resource.do?r=lacm_verts.
603031	GBIF	1966	http://portal.vertnet.org/o/lacm/fish?id=49985720-78bd-481b-86aa-fbf8f6d8a4e0	49985720-78BD-481B-86AA-FBF8F6D8A4E0. http://ipt.vertnet.org:8080/ipt/resource.do?r=lacm_verts.

	source	year	references	bibliographicCitation
629131	GBIF	1964	http://portal.vertnet.org/o/lacm/fish?id=71d6972a-ede9-49bb-9972-260ca43024c0	71D6972A-EDE9-49BB-9972-260CA43024C0. http://ipt.vertnet.org:8080/ipt/resource.do?r=lacm_verts.
670281	GBIF	1964	http://portal.vertnet.org/o/lacm/fish?id=b2676956-14c8-4067-a7fe-e859093f5ff8	B2676956-14C8-4067-A7FE-E859093F5FF8. http://ipt.vertnet.org:8080/ipt/resource.do?r=lacm_verts.
695351	GBIF	1963	http://portal.vertnet.org/o/lacm/fish?id=d964e4db-d2fc-4a0e-82d3-f8eb93ec818f	D964E4DB-D2FC-4A0E-82D3-F8EB93EC818F. http://ipt.vertnet.org:8080/ipt/resource.do?r=lacm_verts.

Information about the outlier :

	source	decimalLongitude	decimalLatitude	year	references
250591	GBIF	137.265	29.79	1970	http://portal.vertnet.org/o/sio/marine-vertebrates?id=b2353fae-6f29-4945-94ba-ed77af1aa3a9

Protomyctophum tenisoni

We found multiple records with swapped coordinates from the Eltanin GBIF erroneous batch (blue points in map). We corrected their coordinates (purple points in map).

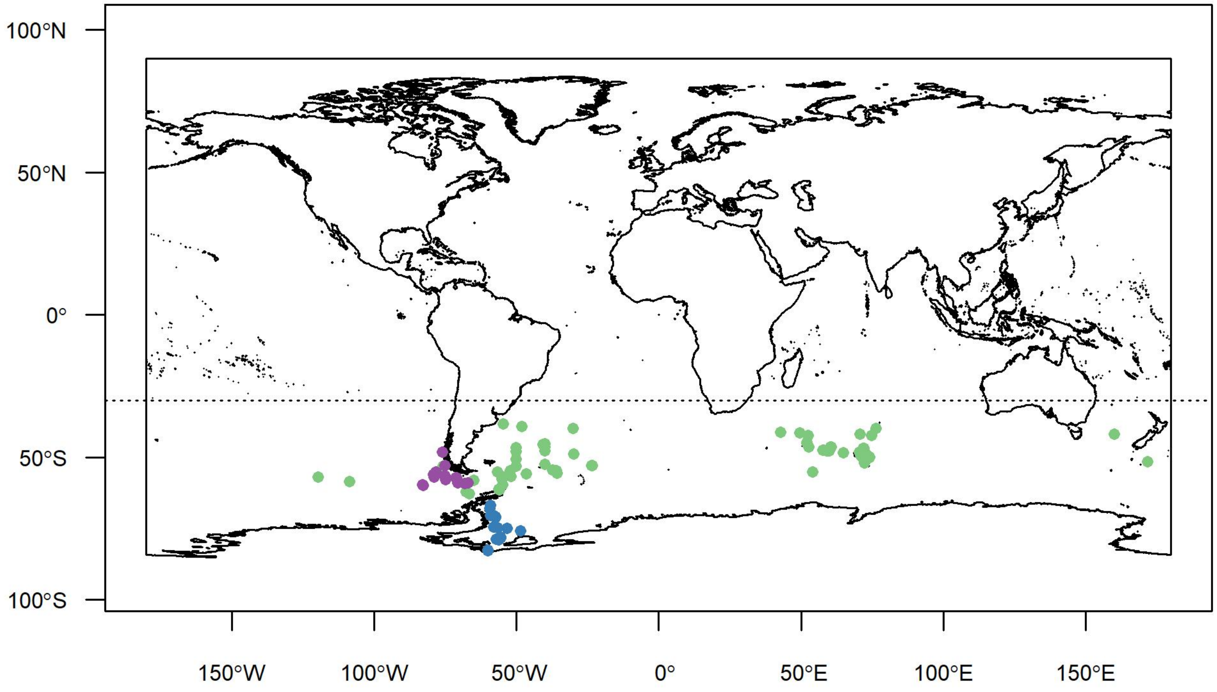

Information about Eltanin records with swapped coordinates are indicated here:

	source	year	references	bibliographicCitation
557361	GBIF	1962	http://portal.vertnet.org/o/lacm/fish?id=02dfd266-c795-4611-a530-10d62214bbaf	02DFD266-C795-4611-A530-10D62214BBAF. http://ipt.vertnet.org:8080/ipt/resource.do?r=lacm_verts.
567551	GBIF	1962	http://portal.vertnet.org/o/lacm/fish?id=128cefe9-f6a3-4595-bcf7-fa54072ff94b	128CEFE9-F6A3-4595-BCF7-FA54072FF94B. http://ipt.vertnet.org:8080/ipt/resource.do?r=lacm_verts.
574031	GBIF	1962	http://portal.vertnet.org/o/lacm/fish?id=1c1aa3a5-7827-4124-9e29-1f4e1174e129	1C1AA3A5-7827-4124-9E29-1F4E1174E129. http://ipt.vertnet.org:8080/ipt/resource.do?r=lacm_verts.

	source	year	references	bibliographicCitation
575391	GBIF	1964	http://portal.vertnet.org/o/lacm/fish?id=1e66206b-0499-4c0b-9ac8-a662849fa1da	1E66206B-0499-4C0B-9AC8-A662849FA1DA. http://ipt.vertnet.org:8080/ipt/resource.do?r=lacm_verts.
576701	GBIF	1963	http://portal.vertnet.org/o/lacm/fish?id=204eeee6-1bf8-49e9-a7b1-960fca0dd379	204EEEE6-1BF8-49E9-A7B1-960FCA0DD379. http://ipt.vertnet.org:8080/ipt/resource.do?r=lacm_verts.
583761	GBIF	1962	http://portal.vertnet.org/o/lacm/fish?id=2b3a84eb-1a31-4346-b8b6-f5c5c4891d49	2B3A84EB-1A31-4346-B8B6-F5C5C4891D49. http://ipt.vertnet.org:8080/ipt/resource.do?r=lacm_verts.
598671	GBIF	1963	http://portal.vertnet.org/o/lacm/fish?id=431b5364-18ba-4320-adca-3580cfd16c6f	431B5364-18BA-4320-ADCA-3580CFD16C6F. http://ipt.vertnet.org:8080/ipt/resource.do?r=lacm_verts.
601541	GBIF	1962	http://portal.vertnet.org/o/lacm/fish?id=471ca1fe-b91a-4e3d-8bd5-7162207b47c4	471CA1FE-B91A-4E3D-8BD5-7162207B47C4. http://ipt.vertnet.org:8080/ipt/resource.do?r=lacm_verts.
608512	GBIF	1962	http://portal.vertnet.org/o/lacm/fish?id=519c5db2-676e-4a95-9dc1-49f241eab07c	519C5DB2-676E-4A95-9DC1-49F241EAB07C. http://ipt.vertnet.org:8080/ipt/resource.do?r=lacm_verts.
614051	GBIF	1962	http://portal.vertnet.org/o/lacm/fish?id=59eceaec-fae3-4575-a78f-421016a065f5	59ECEAEC-FAE3-4575-A78F-421016A065F5. http://ipt.vertnet.org:8080/ipt/resource.do?r=lacm_verts.
632721	GBIF	1963	http://portal.vertnet.org/o/lacm/fish?id=77c1ce4e-ba54-4f10-8796-8ca00e48fdaf	77C1CE4E-BA54-4F10-8796-8CA00E48FDAF. http://ipt.vertnet.org:8080/ipt/resource.do?r=lacm_verts.
636461	GBIF	1963	http://portal.vertnet.org/o/lacm/fish?id=7da4babf-e05d-4260-badf-b9376dcbdbf7	7DA4BABF-E05D-4260-BADF-B9376DCBDBF7. http://ipt.vertnet.org:8080/ipt/resource.do?r=lacm_verts.
640351	GBIF	1963	http://portal.vertnet.org/o/lacm/fish?id=83bfee36-e589-4ebb-8cce-32acc1d68696	83BFEE36-E589-4EBB-8CCE-32ACC1D68696. http://ipt.vertnet.org:8080/ipt/resource.do?r=lacm_verts.
644151	GBIF	1963	http://portal.vertnet.org/o/lacm/fish?id=89ddac15-c06a-4a6f-8c4a-c844fb343f09	89DDAC15-C06A-4A6F-8C4A-C844FB343F09. http://ipt.vertnet.org:8080/ipt/resource.do?r=lacm_verts.
688501	GBIF	1963	http://portal.vertnet.org/o/lacm/fish?id=ce86fe45-9780-4767-be1b-e52467f055cb	CE86FE45-9780-4767-BE1B-E52467F055CB. http://ipt.vertnet.org:8080/ipt/resource.do?r=lacm_verts.
689401	GBIF	1962	http://portal.vertnet.org/o/lacm/fish?id=cfa3b2da-7f29-412a-b0aa-1cb3924de06e	CFA3B2DA-7F29-412A-B0AA-1CB3924DE06E. http://ipt.vertnet.org:8080/ipt/resource.do?r=lacm_verts.
704671	GBIF	1963	http://portal.vertnet.org/o/lacm/fish?id=e7cb05da-d2f1-4552-831d-119c4e6d1be1	E7CB05DA-D2F1-4552-831D-119C4E6D1BE1. http://ipt.vertnet.org:8080/ipt/resource.do?r=lacm_verts.

Scopelopsis multipunctatus

We found outliers in the Atlantic Ocean and in the Indian Ocean (red points on the map), which we removed.

There were several potential outliers in the Pacific Ocean (brown points in map) which we decided to keep after verification.

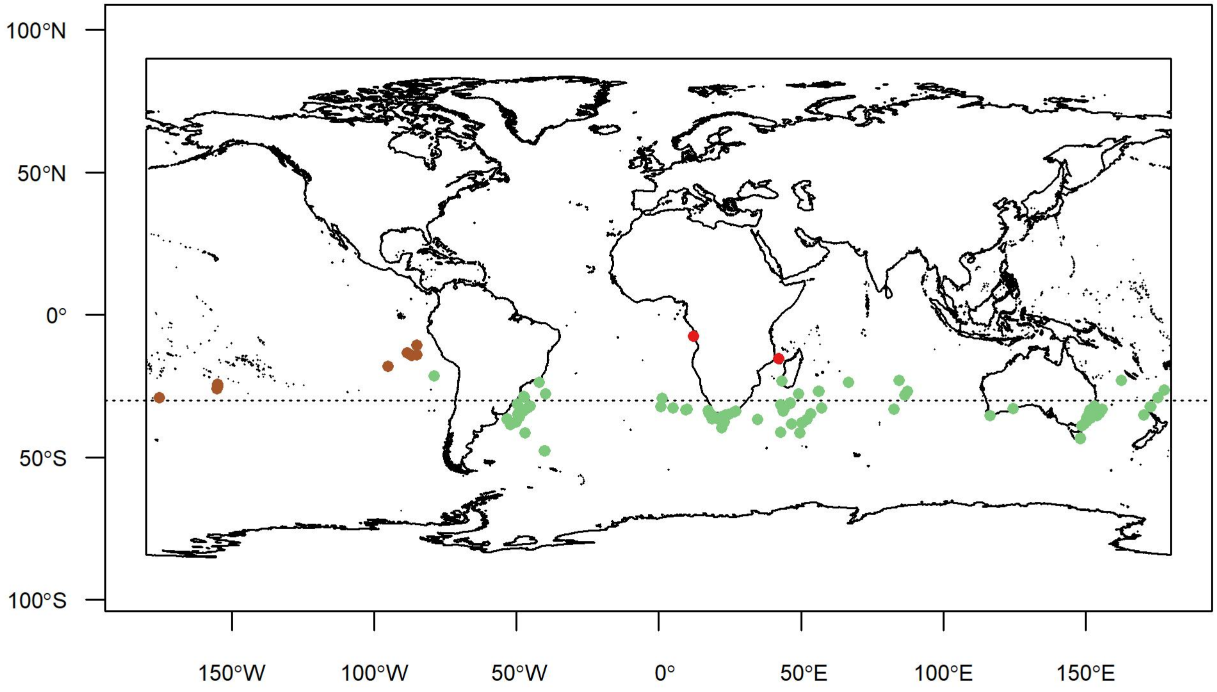

Information about the Atlantic & Indian outliers :

	source	decimalLongitude	decimalLatitude	year	references
107419	OBIS	12.2333	-7.5667	2002	
140573	GBIF	42.2500	-15.4500	1988	

Information about the Pacific outliers :

	source	decimalLongitude	decimalLatitude	year	references
217151	GBIF	-95.0833	-18.2667	1960	http://portal.vertnet.org/o/sio/marine-vertebrates?id=5a59a617-f25e-42b6-a13d-f1433d0e5d4e
248801	GBIF	-155.0317	-26.0100	1969	http://portal.vertnet.org/o/sio/marine-vertebrates?id=320dc829-7d68-4389-8140-f8d57ca7675a
248861	GBIF	-154.8200	-25.4117	1969	http://portal.vertnet.org/o/sio/marine-vertebrates?id=fa4aadcf-91c2-4974-87ce-efe129e0f244
249012	GBIF	-154.8167	-24.6333	1969	http://portal.vertnet.org/o/sio/marine-vertebrates?id=a2766844-e473-42ce-a77f-f5e9eaccc4fc
249131	GBIF	-154.8167	-24.9667	1969	http://portal.vertnet.org/o/sio/marine-vertebrates?id=82a07b38-50d7-4cdc-8aed-d8f59cda5691
249461	GBIF	-155.0050	-24.6450	1969	http://portal.vertnet.org/o/sio/marine-vertebrates?id=7813b950-f086-4472-9178-bf4cba49b8f8
249581	GBIF	-154.9167	-24.5283	1969	http://portal.vertnet.org/o/sio/marine-vertebrates?id=e1cb9bee-2a78-4d3e-bc07-81778fc9570b
249821	GBIF	-154.8333	-24.5667	1969	http://portal.vertnet.org/o/sio/marine-vertebrates?id=51f9681d-48d5-45cf-b2d4-441ffb223d11
323931	GBIF	-84.9667	-14.1500	1968	http://portal.vertnet.org/o/sio/marine-vertebrates?id=5095e2f1-aed1-4e4b-bcc9-8e0b46d460c9
324371	GBIF	-85.0333	-10.8333	1968	http://portal.vertnet.org/o/sio/marine-vertebrates?id=805dec57-70ae-458d-a60a-00c42a35ccf8
353081	GBIF	-88.3000	-13.5483	1967	http://portal.vertnet.org/o/sio/marine-vertebrates?id=d06a9c93-9873-47bd-acb9-8d7a180b05b5
353241	GBIF	-86.8333	-14.4500	1967	http://portal.vertnet.org/o/sio/marine-vertebrates?id=184a901e-58f5-4268-986a-5d885320cd5a
180111	GBIF	-175.4420	-29.1830	1967	

Symbolophorus barnardi

We found one outlier in the Atlantic Ocean (red point on the map), which we removed.

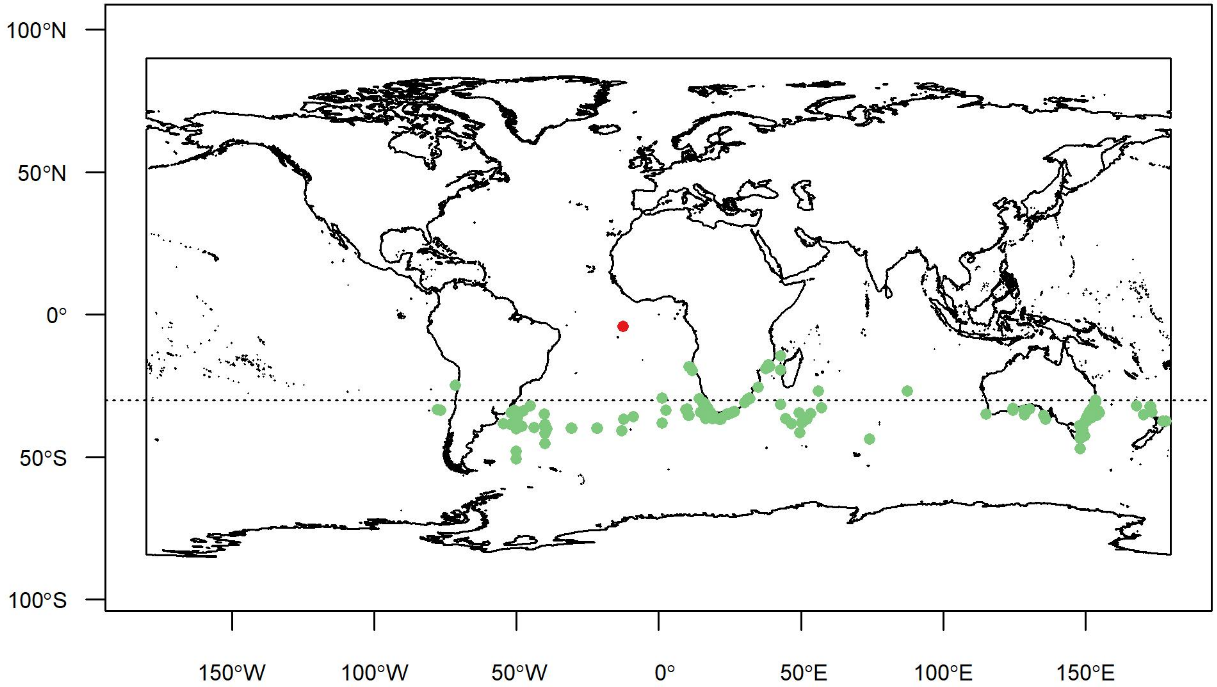

Information about the Atlantic outlier :

	source	decimalLongitude	decimalLatitude	year	references
79701	OBIS	-12.405	-4.1578	2009	

Symbolophorus boops

We found outliers to the North of this species' distribution (red points in map), which we removed.

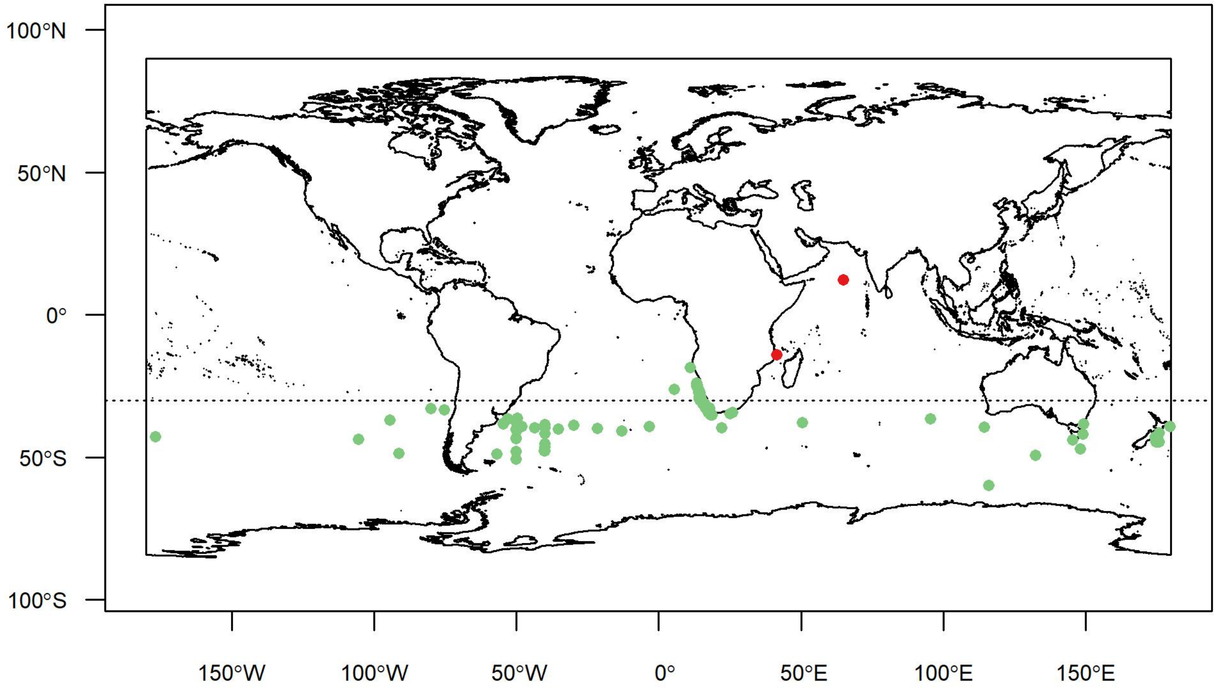

Information about the outliers :

	source	decimalLongitude	decimalLatitude	year	references
893291	GBIF	41.4440	-14.1707	2007	
1216521	GBIF	65.0083	12.1250	1964	http://mczbase.mcz.harvard.edu/guid/MCZ:Ich:108898

Symbolophorus evermanni

We found two outliers in the Atlantic Ocean (red points on the map), which we removed.

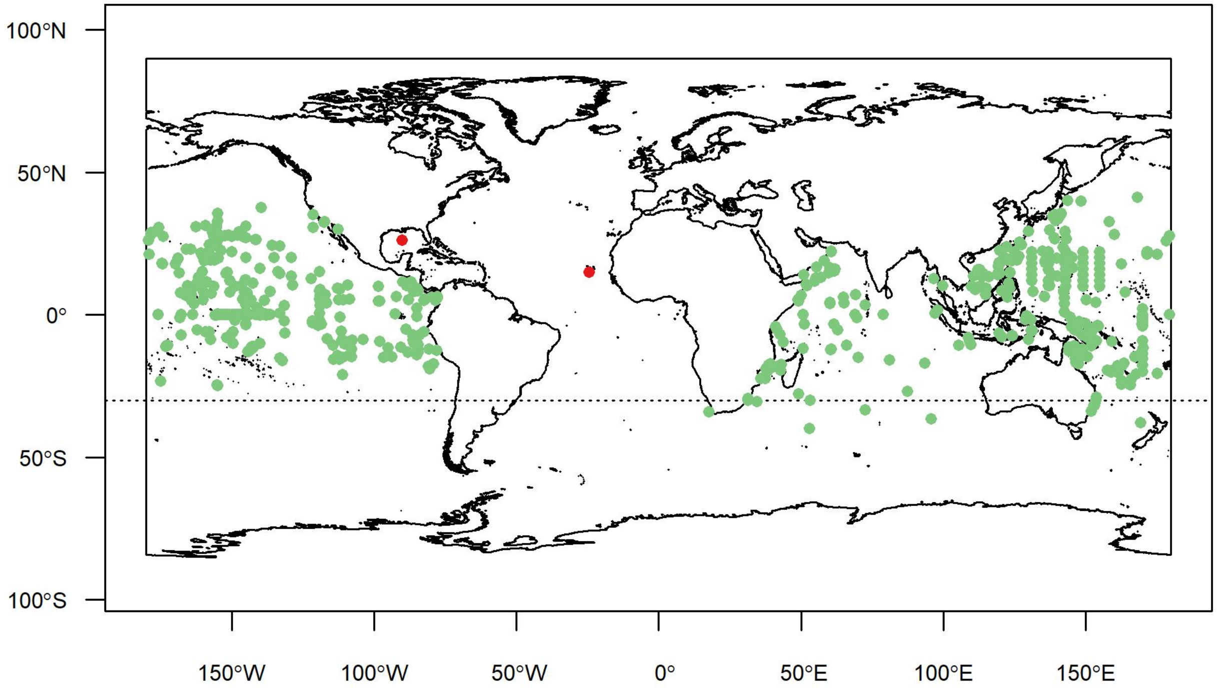

Information about the outliers :

	source	decimalLongitude	decimalLatitude	year	references
392631	GBIF	-90.00	26.0000	1991	http://portal.vertnet.org/o/sio/marine-vertebrates?id=9351f6ff-8e74-4dfb-9788-c14bef4dfc2f
547001	GBIF	-24.36	14.7967	2011	

Taaningichthys bathyphilus

We found no outliers for this species. Several records in the Southern Ocean could be considered as outliers, but their identification has been verified and they are considered as valid records.

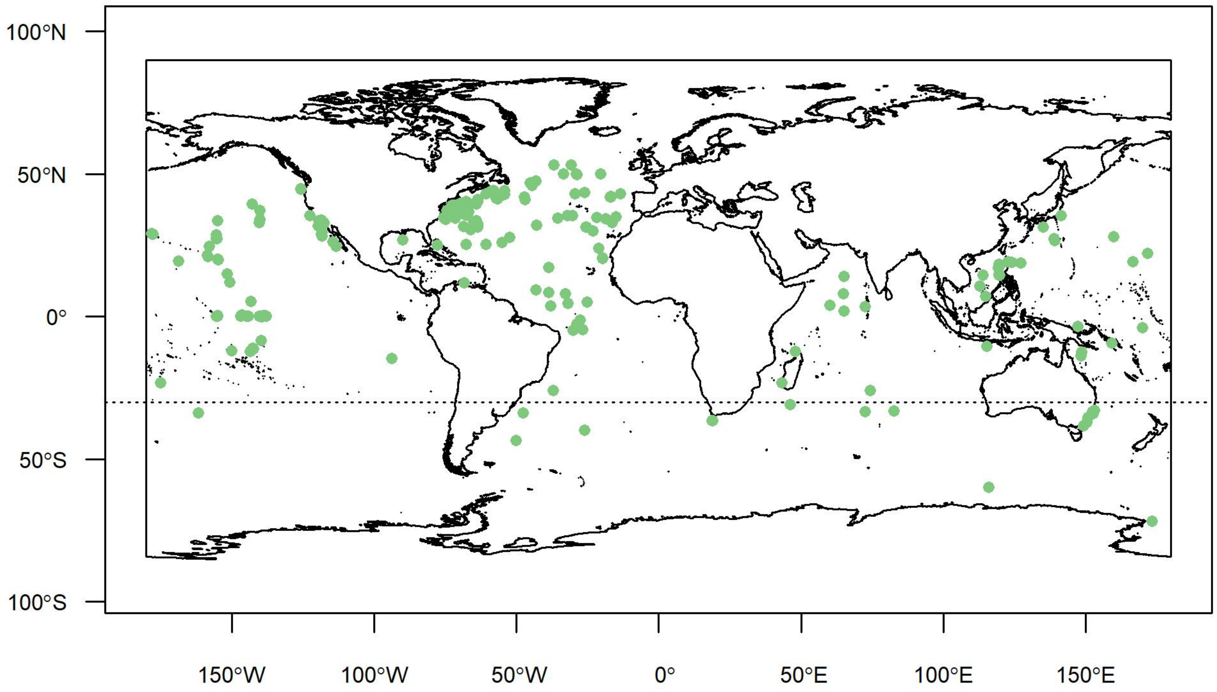

Taaningichthys minimus

We found one outlier in the Black Sea (blue point on the map), which after verification appeared to be a sign error of the coordinates, so we corrected it (purple point on the map)

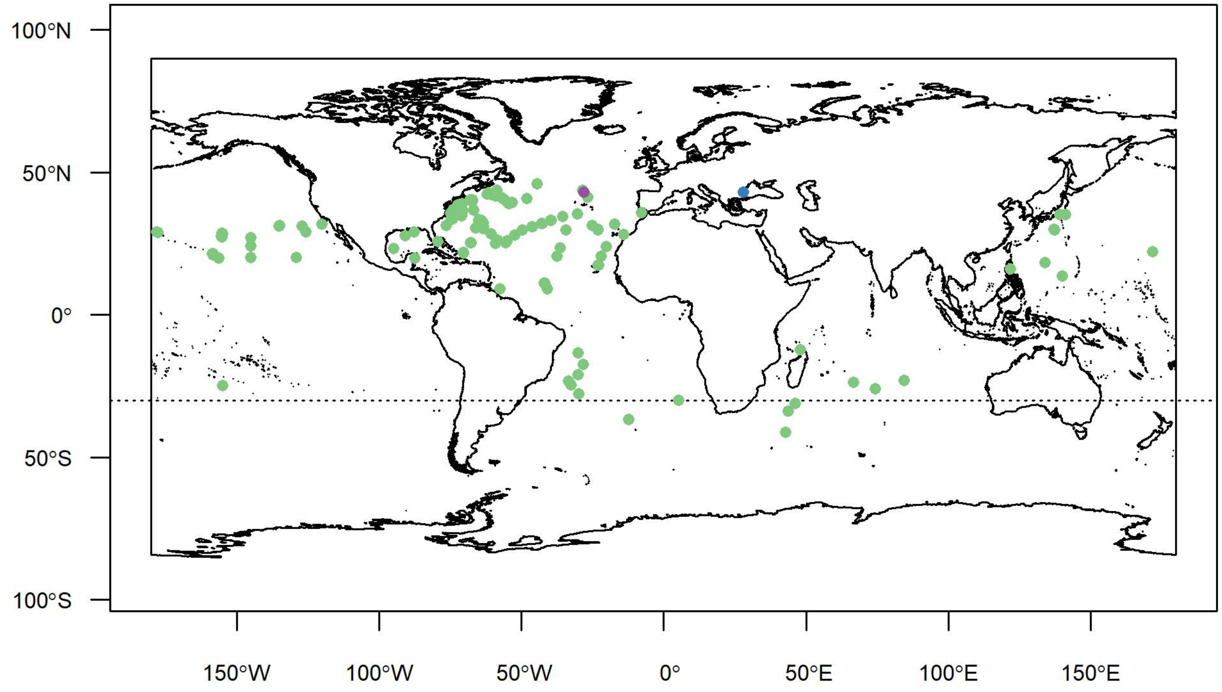

Information about the outlier :

	source	decimalLongitude	decimalLatitude	year	references
143367	GBIF	28	43	1982	

Triphoturus mexicanus

We found two outliers in the Western Pacific Ocean (red points on the map), which we removed.

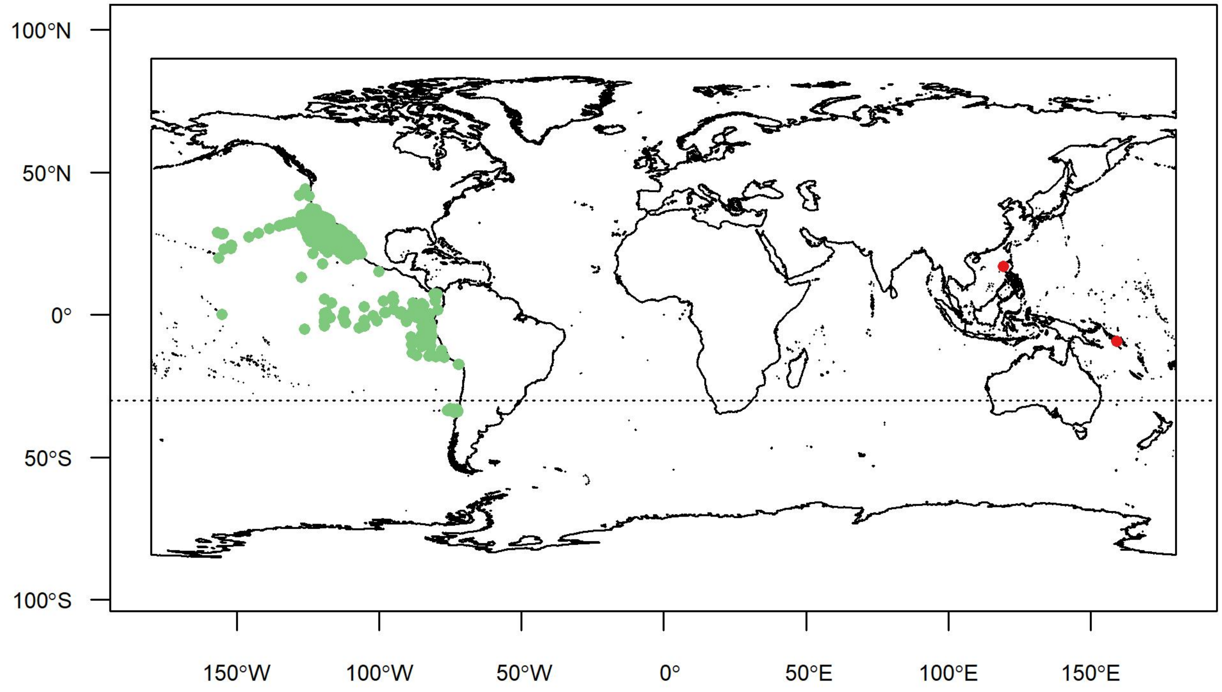

Information about the outliers :

	source	decimalLongitude	decimalLatitude	year	references
253031	GBIF	119.4	16.8667	1970	http://portal.vertnet.org/o/sio/marine-vertebrates?id=b53dbede-0db6-4c78-b7ed-320c7193c910
277981	GBIF	159.1	-9.4167	1975	http://portal.vertnet.org/o/sio/marine-vertebrates?id=eb21ac60-9230-4e56-bcbf-9ba8fa20442b

Triphoturus nigrescens

We found no outliers for this species.

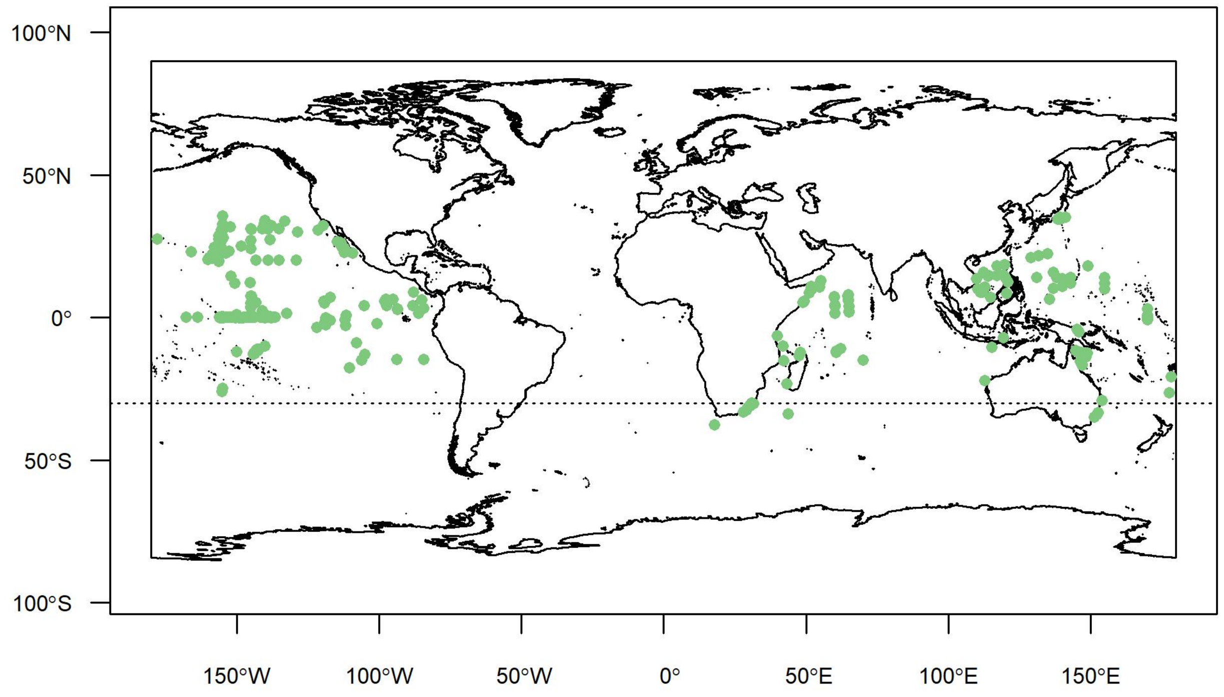

References

Cavallaro, M., Ammendolia, G., Andaloro, F., & Battaglia, P. (2017). First record of the mesopelagic fish Diaphus dumerilii (Bleeker, 1856) in the Mediterranean Sea. Marine Biodiversity, 47(2), 585-588. https://doi.org/10.1007/s12526-016-0492-3

Duhamel, G., Hulley, P.-A., Causse, R., Koubbi, P., Vacchi, M., Pruvost, P., Vigetta, S., Irisson, J.-O., Mormede, S., Belchier, M., Dettai, A., Detrich, H.

W., Gutt, J., Jones, C. D., Kock, K.-H., Lopez Abellan, L. J., & Van de Putte, A. (2014). Biogeographic patterns of fish (C. De Broyer, P. Koubbi, H. Griffiths, B. Raymond, C. d'Udekem d'Acoz, A. Van de Putte, B. Danis, B. David, S. Grant, J. Gutt, C. Held, G. Hosie, F. Huettmann, A. Post, & Y. Ropert-Coudert, Éds.; p. 328-362). Scientific Committee on Antarctic Research. https://nora.nerc.ac.uk/id/eprint/508228/

Flynn, A. J., & Paxton, J. R. (2012). Spawning aggregation of the lanternfish Diaphus danae (family Myctophidae) in the north-western Coral Sea and associations with tuna aggregations. Marine and Freshwater Research, 63(12), 1255-1271. https://doi.org/10.1071/MF12185

Hulley, P., & Duhamel, G. (2009). A review of the lanternfish genus Bolinichthys Paxton, 1972 (Myctophidae). Cybium, 33, 259-304.

Koubbi, P., Djian, V., Vacchi, M., Rintz, C. L., Leroy, B., Walters, A., Serandour, B., & Tavernier, E. (2023, juin 15). Atlas of mesopelagic fish in the sub-Antarctic Indian and in the South Indian Ocean | Meetings. https://meetings.ccamlr.org/en/wg-emm-2023/20

Meera, K. M. (2018). Myctophids of Western Indian Ocean with special reference to Eastern Arabian Sea. University. https://shodhganga.inflibnet.ac.in:8443/jspui/handle/10603/273878

Namiki, C., Katsuragawa, M., & Zani-Teixeira, M. L. (2015). Growth and mortality of larval Myctophum affine (Myctophidae, Teleostei). Journal of Fish Biology, 86(4), 1335-1347. https://doi.org/10.1111/jfb.12643

Sutton, T. T., Hulley, P. A., Wienerroither, R., Zaera-Perez, D., & Paxton, J. R. (2020). Identification guide to the mesopelagic fishes of the central and south east Atlantic Ocean. https://policycommons.net/artifacts/1422305/identification-guide-to-the-mesopelagic-fishes-of-the-central-and-south-east-atlantic-ocean/2036388/

Zahuranec, B. J. (2000). Zoogeography and systematics of the lanternfishes of the genus Nannobrachium (Myctophidae:Lampanyctini). http://repository.si.edu/xmlui/handle/10088/5099

Zizka, A., Silvestro, D., Andermann, T., Azevedo, J., Duarte Ritter, C., Edler, D., Farooq, H., Herdean, A., Ariza, M., Scharn, R., Svantesson, S., Wengström, N., Zizka, V., & Antonelli, A. (2019). CoordinateCleaner : Standardized cleaning of occurrence records from biological collection databases. Methods in Ecology and Evolution, 10(5), 744-751. https://doi.org/10.1111/2041-210X.13152
