# Supplementary material for: Biogeographical Regions and Climate Change: Lanternfishes Shed Light on the Role of Climatic Barriers in the Southern Ocean
Source: Glob Chang Biol. 2025 Jun 16;31(6):e70256. doi: 10.1111/gcb.70256 (PMC12168107; doi:10.1111/gcb.70256)
Supplement: Supplementary file 3 — Appendix S2. Appendix S3. Appendix S5. Appendix S6. Appendix S7. Appendix S8. Appendix S9. Appendix S10. Appendix S11. Appendix S12. Appendix S13. Appendix S14. [file GCB-31-e70256-s002.pdf]

Supporting Information

Appendix S2: Influence of the occurrence threshold on species selection. This graph displays the number of selected species depending on the value of the occurrence threshold, which is the minimum number of records below 30°S required for a species to be included in the analysis. The highlighted bar corresponds to the final chosen threshold.

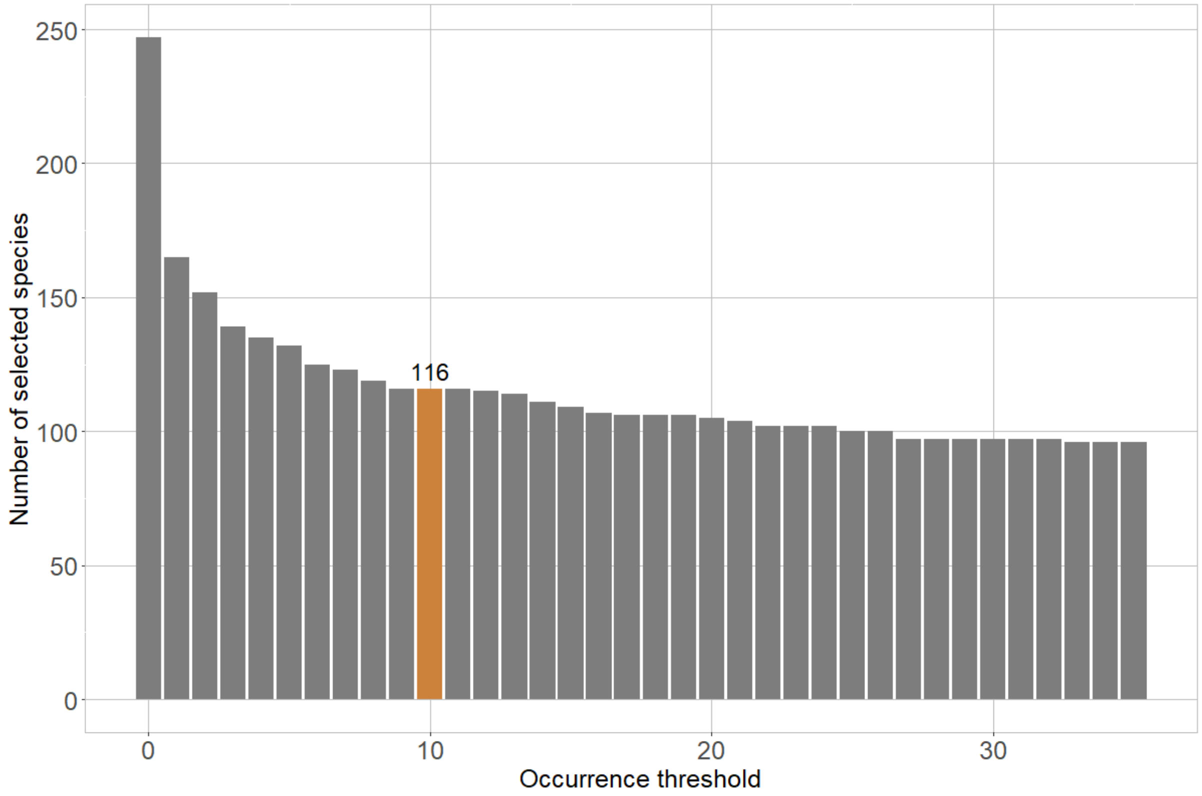

Supporting Information

Appendix S3: List of species with the occurrence corrections. The displayed number of records corresponds to the final count resulting from the correction procedure and temporal filtering (spring-summer, 1950-2019).

Species	Corrections	Source	Total number of records	Number of records below 30°S	% records below 30°S	Cluster
Benthosema fibulatum			215	10	5	1
Benthosema suborbitale			1158	121	10	1
Bolinichthys indicus	3 outliers removed	Expert assessment<br>Hulley & Duhamel, 2009<br>FishBase	688	30	4	1
Bolinichthys longipes	7 outliers removed	Expert assessment<br>Hulley & Duhamel, 2009<br>FishBase	689	7	1	1
Bolinichthys nikolayi	2 outliers removed	Expert assessment<br>Hulley & Duhamel, 2009<br>FishBase	27	14	52	1
Bolinichthys photothorax			325	4	1	1
Bolinichthys supralateralis	1 outlier removed	Expert assessment<br>Hulley & Duhamel, 2009<br>Meera, 2018<br>FishBase	393	76	19	1
Centrobranchus nigroocellatus			649	23	4	1
Ceratoscopelus townsendi	Species removed because only distributed in the North-Eastern Pacific	Expert assessment<br>FishBase	-	-		-
Ceratoscopelus warmingii			2020	297	15	1
Diaphus anderseni			165	19	12	1
Diaphus bertelseni			55	9	16	1
Diaphus brachycephalus	1 outlier removed	Expert assessment<br>FishBase	490	24	5	1
Diaphus coeruleus			39	24	62	8
Diaphus danae			126	114	90	4
Diaphus diadematus	11 outliers removed	Expert assessment<br>FishBase	49	17	35	1
Diaphus dumerilii			857	19	2	1
Diaphus effulgens			423	44	10	1
Diaphus fragilis			349	32	9	1

Diaphus garmani			417	18	4	1
Diaphus holti	Species removed because only distributed in the Northern Atlantic	Expert assessment<br>Sutton et al., 2020<br>FishBase	-	-	-	-
Diaphus hudsoni	1 outlier removed<br>2 corrections of swapped coordinates	Expert assessment<br>VertNet<br>FishBase	295	282	96	1
Diaphus kapalae	1 outlier removed	Expert assessment<br>FishBase	19	14	74	1
Diaphus lucidus			390	35	9	1
Diaphus luetkeni	1 outlier removed	Expert assessment<br>FishBase	470	7	1	1
Diaphus mascarensis			8	6	75	1
Diaphus meadi	1 outlier removed	Expert assessment<br>FishBase	211	199	94	1
Diaphus metopoclampus			339	70	21	1
Diaphus mollis			1139	101	9	1
Diaphus ostenfeldi	2 outliers removed	Expert assessment<br>FishBase	128	125	98	1
Diaphus parri			301	25	8	1
Diaphus perspicillatus			358	29	8	1
Diaphus splendidus	Species removed because no records left below 30°S after temporal filtering		558	0	0	-
Diaphus taaningi	Species removed because only distributed above 30°S in the Atlantic	Expert assessment<br>FishBase	-	-	-	-
Diaphus termophilus	2 outliers removed	Expert assessment<br>FishBase	281	59	21	1
Diaphus theta	Species removed because often subject to misidentification	Expert assessment<br>FishBase	-	-	-	-
Diaphus watasei	1 outlier removed	Expert assessment<br>FishBase	76	13	17	8
Diogenichthys atlanticus			1575	88	6	1
Diogenichthys panurgus			111	5	5	1
Electrona antarctica	6 outliers removed	Expert assessment<br>FishBase	1207	1207	100	2
Electrona carlsbergi	3 corrections of swapped coordinates	VertNet	636	636	100	2
Electrona paucirastra	1 outlier removed<br>4 corrections of swapped coordinates	Expert assessment<br>VertNet<br>FishBase	38	38	100	10
Electrona risso		Expert assessment<br>FishBase	721	220	31	1

Electrona subaspera	4 corrections of swapped coordinates	VertNet	264	264	100	2
Gonichthys barnesi	7 outliers removed	Expert assessment<br>FishBase	81	78	96	1
Gonichthys cocco	2 outliers removed	Expert assessment<br>FishBase	700	4	1	1
Gymnoscopelus basili	Removed because wrong species name	Expert assessment<br>WoRMS	-		-	-
Gymnoscopelus bolini	4 corrections of swapped coordinates	VertNet	351	351	100	2
Gymnoscopelus braueri	19 corrections of swapped coordinates	VertNet	904	904	100	2
Gymnoscopelus fraseri	12 corrections of swapped coordinates	VertNet	320	320	100	2
Gymnoscopelus fraseroides	Removed because wrong species name	Expert assessment<br>WoRMS	-		-	-
Gymnoscopelus hintonoides			53	53	100	2
Gymnoscopelus knudseni	Removed because wrong species name	Expert assessment<br>WoRMS	-	-	-	-
Gymnoscopelus microlampas	1 outlier removed	Expert assessment<br>FishBase	135	135	100	2
Gymnoscopelus nicholsi	22 corrections of swapped coordinates	VertNet	1327	1327	100	2
Gymnoscopelus opisthopterus	2 outliers removed<br>11 corrections of swapped coordinates	Expert assessment<br>VertNet	230	230	100	2
Gymnoscopelus piabilis	5 outliers removed<br>3 corrections of swapped coordinates	Expert assessment<br>VertNet<br>FishBase	265	265	100	3
Hintonia candens	2 corrections of swapped coordinates	VertNet	43	42	98	3
Hygophum bruuni	1 outlier removed<br>4 corrections of swapped coordinates	Expert assessment<br>VertNet<br>FishBase	13	11	85	5
Hygophum hanseni	2 outliers removed<br>1 correction of swapped coordinates	Expert assessment<br>VertNet<br>FishBase	129	127	98	1
Hygophum hygomii			1297	205	16	1
Hygophum macrochir	Species removed because often subject to misidentification	Expert assessment<br>FishBase	-	-	-	-
Hygophum proximum	1 outlier removed	Expert assessment<br>FishBase	612	18	3	1
Hygophum reinhardtii			930	47	5	1
Krefftichthys anderssoni	23 corrections of swapped coordinates	VertNet	888	888	100	2

Lampadena chavesi			163	18	11	1
Lampadena dea			48	39	81	1
Lampadena luminosa			365	23	6	1
Lampadena notialis			74	72	97	1
Lampadena speculigera			538	98	18	1
Lampanyctodes hectoris	2 corrections of swapped coordinates	VertNet	1128	999	89	4
Lampanyctus achirus	34 outliers removed<br>19 corrections of swapped coordinates	Expert assessment<br>VertNet<br>FishBase	318	311	98	2
Lampanyctus alatus	1 outlier removed	Expert assessment<br>FishBase	821	128	16	1
Lampanyctus ater	41 outliers removed	Expert assessment<br>Zahuranec, 2000<br>FishBase	741	135	18	1
Lampanyctus australis	106 outliers removed<br>6 corrections of swapped coordinates	Expert assessment<br>VertNet<br>FishBase	384	359	93	1
Lampanyctus festivus			473	102	22	1
Lampanyctus intricarius		Expert assessment<br>FishBase	471	130	28	1
Lampanyctus iselinoides	4 corrections of swapped coordinates	VertNet	31	31	100	5
Lampanyctus lepidolychnus	9 outliers removed	Expert assessment<br>FishBase	127	114	90	1
Lampanyctus macdonaldi	12 outliers removed<br>9 corrections of swapped coordinates	Expert assessment<br>VertNet<br>FishBase	758	212	28	2
Lampanyctus nobilis			509	4	1	9
Lampanyctus patagonica	Removed because wrong species name	Expert assessment<br>WoRMS	-	-	-	-
Lampanyctus phyllisae			22	13	59	5
Lampanyctus pusillus	1 outlier removed	Expert assessment<br>FishBase	1221	155	13	1
Lampanyctus tenuiformis			378	13	3	1
Lampanyctus turneri	2 outliers removed	Expert assessment<br>FishBase	47	9	19	1
Lampanyctus wisneri			30	25	83	1
Lampichthys procerus	2 outliers removed<br>2 corrections of swapped coordinates	Expert assessment<br>Kobylianski et al., 2010<br>VertNet<br>FishBase	275	263	96	1

Lepidophanes gaussi	1 outlier removed	Expert assessment<br>FishBase	483	1	0	1
Lepidophanes guentheri	1 outlier removed	Expert assessment<br>FishBase	940	23	2	1
Lobianchia dofleini	2 outliers removed	Expert assessment<br>FishBase	1462	170	12	1
Lobianchia gemellarii			1089	47	4	1
Loweina interrupta			22	1	5	9
Loweina rara			134	8	6	1
Metelectrona ahlstromi	2 outliers removed	Expert assessment<br>FishBase	7	7	100	5
Metelectrona herwigi			7	7	100	3
Metelectrona ventralis	2 corrections of swapped coordinates	VertNet	145	143	99	6
Myctophum affine	8 outliers removed	Expert assessment<br>Namiki et al., 2015<br>FishBase	554	10	2	1
Myctophum asperum			515	36	7	1
Myctophum nitidulum			1276	37	3	1
Myctophum obtusirostre			487	4	1	1
Myctophum phengodes	1 outlier removed<br>1 correction of swapped coordinates	Expert assessment<br>VertNet<br>FishBase	218	187	86	1
Myctophum punctatum	Species removed because only distributed in the Northern Atlantic	Expert assessment<br>FishBase	-	-	-	-
Myctophum selenops			383	53	14	1
Myctophum spinosum	1 outlier removed	Expert assessment<br>FishBase	195	3	2	11
Notolychnus valdiviae			1835	64	3	1
Notoscopelus caudispinosus	1 outlier removed	Expert assessment<br>FishBase	308	21	7	1
Notoscopelus resplendens	1 outlier removed	Expert assessment<br>FishBase	1149	189	16	1
Protomyctophum andriashevi	5 corrections of swapped coordinates	VertNet	235	235	100	2
Protomyctophum bolini	23 corrections of swapped coordinates	VertNet	796	796	100	2
Protomyctophum chilense			5	4	80	5
Protomyctophum choriodon			145	145	100	2
Protomyctophum gemmatum			67	67	100	2
Protomyctophum luciferum			63	63	100	3

Protomyctophum normani	2 outliers removed	Expert assessment<br>FishBase	80	80	100	7
Protomyctophum parallelum	1 outlier removed<br>25 corrections of swapped coordinates	Expert assessment<br>VertNet<br>FishBase	108	108	100	2
Protomyctophum subparallelum	1 outlier removed<br>6 corrections of swapped coordinates	Expert assessment<br>VertNet<br>FishBase	107	107	100	5
Protomyctophum tenisoni	17 corrections of swapped coordinates	VertNet	316	316	100	2
Scopelopsis multipunctatus	2 outliers removed	Expert assessment<br>C. Klepadlo (pers. comm., 2020)<br>FishBase	201	167	83	1
Symbolophorus barnardi	1 outlier removed	Expert assessment<br>FishBase	287	267	93	1
Symbolophorus boops	2 outliers removed	Expert assessment<br>FishBase	358	328	92	3
Symbolophorus evermanni	2 outliers removed	Expert assessment<br>FishBase	829	14	2	9
Taaningichthys bathyphilus			354	27	8	1
Taaningichthys minimus	1 correction of sign of coordinates	Expert assessment<br>FishBase	230	7	3	1
Triphoturus mexicanus	2 outliers removed	Expert assessment<br>FishBase	1550	18	1	5
Triphoturus nigrescens			436	19	4	1

References

Froese, R. and D. Pauly. Editors. (2022). FishBase. World Wide Web electronic publication. www.fishbase.org, version (2022-02).

Hulley, P., & Duhamel, G. (2009). A review of the lanternfish genus Bolinichthys Paxton, 1972 (Myctophidae). *Cybium*, *33*, 259-304.

Kobyliansky, S. G., Orlov, A. M., & Gordeeva, N. V. (2010). Composition of deepsea pelagic ichthyocenes of the Southern Atlantic, from waters of the range of the Mid-Atlantic and Walvis Ridges. Journal of Ichthyology, 50(10), 932-949. https://doi.org/10.1134/S0032945210100036

Meera, K. M. (2018). Myctophids of Western Indian Ocean with special reference to Eastern Arabian Sea. University. https://shodhganga.inflibnet.ac.in:8443/jspui/handle/10603/273878

Namiki, C., Katsuragawa, M., & Zani-Teixeira, M. L. (2015). Growth and mortality of larval Myctophum affine (Myctophidae, Teleostei). Journal of Fish Biology, 86(4), 1335-1347. https://doi.org/10.1111/jfb.12643

Sutton, T. T., Hulley, P. A., Wienerroither, R., Zaera-Perez, D., & Paxton, J. R. (2020). Identification guide to the mesopelagic fishes of the central and south east Atlantic Ocean. https://policycommons.net/artifacts/1422305/identification-guide-to-the-mesopelagic-fishes-of-the-central-and-south-east-atlantic-ocean/2036388/

VertNet. www.vertnet.org, version (2016-09-29)

WoRMS Editorial Board (2022). World Register of Marine Species. Available from https://www.marinespecies.org at VLIZ. Accessed 2022-02. doi:10.14284/170

Zahuranec, B. J. (2000). Zoogeography and systematics of the lanternfishes of the genus Nannobrachium (Myctophidae:Lampanyctini). http://repository.si.edu/xmlui/handle/10088/5099

Supporting Information

Appendix S5: List of environmental variables and download process. Following the variable selection process, we removed correlated variables from the analysis: sea surface temperature, sea surface salinity and sea surface oxygen.

Variable	Depth	Time span	Spatial resolution	Source	Downloaded products
Temperature (°C)	Sea surface (0 m), 100 m, 200 m & 500 m	1955-2017 October-March	1°	World Ocean Atlas 2018 (Boyer et al., 2018; Locarnini et al., 2019)	Average of decadal means: 1955-1964, 1965-1974, 1975-1984, 1985-1994, 1995-2004, 2005-2017<br>Seasons: October-November-December & January-February-March
Salinity (psu)	Sea surface (0 m), 100 m, 200 m & 500 m	1955-2017 October-March	1°	World Ocean Atlas 2018 (Boyer et al., 2018; Zweng et al., 2019)	Average of decadal means: 1955-1964, 1965-1974, 1975-1984, 1985-1994, 1995-2004, 2005-2017<br>Seasons: October-November-December & January-February-March
Bathymetry (m)	-	-	30" converted to 1°	SRTM30_PLUS (Becker et al., 2009)	SRTM30_PLUS bathymetry grid (data compilation + satellite model), topo30 format
Chlorophyll a (kg.m-3)	Sea surface (2.5 m), 100 m, 200 m & 500 m	1955-2017 October-March	1°	CMIP6 (Eyring et al., 2016)	GCMs: GFDL-CM4, GFDL-ESM4<br>Periods: historical (1955-2014) and SSP2.4-5 projection (2014-2017)<br>Months: October to March
Dissolved Oxygen (µmol.kg-1)	Sea surface (0 m), 100 m, 200 m & 500 m	1960-2017 October-March	1°	World Ocean Atlas 2018 (Boyer et al., 2018; García et al., 2019)	Average of all available data over 1960-2017<br>Seasons: October-November-December & January-February-March

References

Becker, J. J., Sandwell, D. T., Smith, W. H. F., Braud, J., Binder, B., Depner, J., Fabre, D., Factor, J., Ingalls, S., Kim, S.-H., Ladner, R., Marks, K., Nelson, S., Pharaoh, A., Trimmer, R., Von Rosenberg, J., Wallace, G., & Weatherall, P. (2009). Global Bathymetry and Elevation Data at 30 Arc Seconds Resolution : SRTM30_PLUS. Marine Geodesy, 32(4), 355-371. https://doi.org/10.1080/01490410903297766

Boyer, T., García, H., Locarnini, R., Zweng, M. M., Mishonov, A., Reagan, J., Weathers, K., Baranova, O., Paver, C., Seidov, D., Smolyar, I. (2018). World Ocean Atlas 2018. NOAA National Centers for Environmental Information. Dataset. https://www.ncei.noaa.gov/archive/accession/NCEI-WOA18. Accessed [04/2022].

Eyring, V., Bony, S., Meehl, G. A., Senior, C. A., Stevens, B., Stouffer, R. J., & Taylor, K. E. (2016). Overview of the Coupled Model Intercomparison Project Phase 6 (CMIP6) experimental design and organization. Geoscientific Model Development, 9(5), 1937-1958. https://doi.org/10.5194/gmd-9-1937-2016

García, H., Weathers, K., Paver, C., Smolyar, I., Boyer, T., Locarnini, R., Zweng, M., Mishonov, A., Baranova, O., Seidov, D., & Reagan, J. (2019). World Ocean Atlas 2018, Volume 3 : Dissolved Oxygen, Apparent Oxygen Utilization, and Dissolved Oxygen Saturation. A. Mishonov Technical Ed.; NOAA Atlas NESDIS 83, 38 pp.

Locarnini, R., Mishonov, A., Baranova, O., Boyer, T., Zweng, M., García, H., Reagan, J., Seidov, D., Weathers, K., Paver, C., & Smolyar, I. (2019). World Ocean Atlas 2018, Volume 1 : Temperature. A. Mishonov Technical Ed.; NOAA Atlas NESDIS 81, 52 pp.

Zweng, M. M., Reagan, J., Seidov, D., Boyer, T., Locarnini, R., García, H., Mishonov, A., Baranova, O., Weathers, K., Paver, C., & Smolyar, I. (2019). World Ocean Atlas 2018, Volume 2 : Salinity. A. Mishonov Technical Ed.; NOAA Atlas NESDIS 82, 50 pp.

Supporting Information

Appendix S6: List of CMIP6 simulations used

Global climate model	Institution	Variant	Citation
ACCESS-CM2	CSIRO-ARCCSS	r1i1p1f1	Dix et al., 2019a, 2019b
AWI-CM-1-1-MR	AWI	r1i1p1f1	Semmler et al., 2018, 2019
CanESM5	CCCma	r1i1p1f1	Swart et al., 2019a, 2019b
CIESM	THU	r1i1p1f1	Huang, 2019a, 2019b
CMCC-ESM2	CMCC	r1i1p1f1	Lovato et al., 2021a, 2021b
FGOALS-g3	CAS	r1i1p1f1	Li, 2019a, 2019b
FIO-ESM-2-0	FIO-QLNM	r1i1p1f1	Song et al., 2019a, 2019b
IPSL-CM6A-LR	IPSL	r1i1p1f1	Boucher et al., 2018, 2019
MPI-ESM1-2-LR	MPI-M AWI DKRZ DWD	r1i1p1f1	Wieners et al., 2019a, 2019b
MRI-ESM2-0	MRI	r1i1p1f1	Yukimoto et al., 2019a, 2019b

References

Boucher, O., Denvil, S., Levavasseur, G., Cozic, A., Caubel, A., Foujols, M., Meurdesoif, Y., Cadule, P., Devilliers, M., Ghattas, J., Lebas, N., Lurton, T., Mellul, L., Musat, I., Mignot, J., & Cheruy, F. (2018). IPSL IPSL-CM6A-LR model output prepared for CMIP6 CMIP. Version 20220715.Earth System Grid Federation. https://doi.org/10.22033/ESGF/CMIP6.1534

Boucher, O., Denvil, S., Levavasseur, G., Cozic, A., Caubel, A., Foujols, M., Meurdesoif, Y., Cadule, P., Devilliers, M., Dupont, E., & Lurton, T. (2019). IPSL IPSL-CM6A-LR model output prepared for CMIP6 ScenarioMIP. Version 20220715.Earth System Grid Federation. https://doi.org/10.22033/ESGF/CMIP6.1532

Dix, M., Bi, D., Dobrohotoff, P., Fiedler, R., Harman, I., Law, R., Mackallah, C., Marsland, S., O'Farrell, S., Rashid, H., Srbinovsky, J., Sullivan, A., Trenham, C., Vohralik, P., Watterson, I., Williams, G., Woodhouse, M., Bodman, R., Dias, F. B., . . . Yang, R. (2019a). CSIRO-ARCCSS ACCESS-CM2 model output prepared for CMIP6 CMIP. Version 20220715.Earth System Grid Federation. https://doi.org/10.22033/ESGF/CMIP6.2281

Dix, M., Bi, D., Dobrohotoff, P., Fiedler, R., Harman, I., Law, R., Mackallah, C., Marsland, S., O'Farrell, S., Rashid, H., Srbinovsky, J., Sullivan, A., Trenham, C., Vohralik, P., Watterson, I., Williams, G., Woodhouse, M., Bodman, R., Dias, F. B., . . . Yang, R. (2019b). CSIRO-ARCCSS ACCESS-CM2 model output prepared for CMIP6 ScenarioMIP. Version 20220715.Earth System Grid Federation. https://doi.org/10.22033/ESGF/CMIP6.2285

Huang, W. (2019a). THU CIESM model output prepared for CMIP6 CMIP. Version 20220715.Earth System Grid Federation. https://doi.org/10.22033/ESGF/CMIP6.1352

Huang, W. (2019b). THU CIESM model output prepared for CMIP6 ScenarioMIP. Version 20220715.Earth System Grid Federation. https://doi.org/10.22033/ESGF/CMIP6.1357

Li, L. (2019a). CAS FGOALS-g3 model output prepared for CMIP6 CMIP. Version 20220715.Earth System Grid Federation. https://doi.org/10.22033/ESGF/CMIP6.1783

Li, L. (2019b). CAS FGOALS-g3 model output prepared for CMIP6 ScenarioMIP. Version 20220715.Earth System Grid Federation. https://doi.org/10.22033/ESGF/CMIP6.2056

Lovato, T., Peano, D., Butenschön, M. (2021a). CMCC CMCC-ESM2 model output prepared for CMIP6 CMIP. Version 20220715.Earth System Grid Federation. https://doi.org/10.22033/ESGF/CMIP6.13164

Lovato, T., Peano, D., Butenschön, M. (2021b). CMCC CMCC-ESM2 model output prepared for CMIP6 ScenarioMIP. Version 20220715.Earth System Grid Federation. https://doi.org/10.22033/ESGF/CMIP6.13168

Semmler, T., Danilov, S., Rackow, T., Sidorenko, D., Barbi, D., Hegewald, J., Sein, D., Wang, Q., & Jung, T. (2018). AWI AWI-CM1.1MR model output prepared for CMIP6 CMIP. Version 20220715.Earth System Grid Federation. https://doi.org/10.22033/ESGF/CMIP6.359

Semmler, T., Danilov, S., Rackow, T., Sidorenko, D., Barbi, D., Hegewald, J., Pradhan, H. K., Sein, D., Wang, Q., & Jung, T. (2019). AWI AWI-CM1.1MR model output prepared for CMIP6 ScenarioMIP. Version 20220715.Earth System Grid Federation. https://doi.org/10.22033/ESGF/CMIP6.376

Song, Z., Qiao, F., Bao, Y., Shu, Q., Song, Y., Yang, X. (2019a). FIO-QLNM FIO-ESM2.0 model output prepared for CMIP6 CMIP. Version 20220715.Earth System Grid Federation. https://doi.org/10.22033/ESGF/CMIP6.9047

Song, Z., Qiao, F., Bao, Y., Shu, Q., Song, Y., Yang, X. (2019b). FIO-QLNM FIO-ESM2.0 model output prepared for CMIP6 ScenarioMIP. Version 20220715.Earth System Grid Federation. https://doi.org/10.22033/ESGF/CMIP6.9051

Swart, N. C., Cole, J. N., Kharin, V. V., Lazare, M., Scinocca, J. F., Gillett, N. P., Anstey, J., Arora, V., Christian, J. R., Jiao, Y., Lee, W. G., Majaess, F., Saenko, O. A., Seiler, C., Seinen, C., Shao, A., Solheim, L., Von Salzen, K., Yang, D., . . . Sigmond, M. (2019a). CCCma CanESM5 model output prepared for CMIP6 CMIP. Version 20220715.Earth System Grid Federation. https://doi.org/10.22033/ESGF/CMIP6.1303

Swart, N. C., Cole, J. N., Kharin, V. V., Lazare, M., Scinocca, J. F., Gillett, N. P., Anstey, J., Arora, V., Christian, J. R., Jiao, Y., Lee, W. G., Majaess, F., Saenko, O. A., Seiler, C., Seinen, C., Shao, A., Solheim, L., Von Salzen, K., Yang, D., . . . Sigmond, M. (2019b). CCCma CanESM5 model output prepared for CMIP6 ScenarioMIP. Version 20220715. Earth System Grid Federation. https://doi.org/10.22033/ESGF/CMIP6.1317

Wieners, K., Giorgetta, M., Jungclaus, J., Reick, C., Esch, M., Bittner, M., Legutke, S., Schupfner, M., Wachsmann, F., Gayler, V., Haak, H., De Vrese, P., Raddatz, T., Mauritsen, T., Von Storch, J., Behrens, J., Brovkin, V., Claussen, M., Crueger, T., . . . Roeckner, E. (2019a). MPI-M MPIESM1.2-LR model output prepared for CMIP6 CMIP. Version 20220715.Earth System Grid Federation. https://doi.org/10.22033/ESGF/CMIP6.742

Wieners, K., Giorgetta, M., Jungclaus, J., Reick, C., Esch, M., Bittner, M., Gayler, V., Haak, H., De Vrese, P., Raddatz, T., Mauritsen, T., Von Storch, J., Behrens, J., Brovkin, V., Claussen, M., Crueger, T., Fast, I., Fiedler, S., Hagemann, S., . . . Roeckner, E. (2019b). MPI-M MPIESM1.2-LR model output prepared for CMIP6 ScenarioMIP. Version 20220715.Earth System Grid Federation. https://doi.org/10.22033/ESGF/CMIP6.793

Yukimoto, S., Koshiro, T., Kawai, H., Oshima, N., Yoshida, K., Urakawa, S., Tsujino, H., Deushi, M., Tanaka, T., Hosaka, M., Yoshimura, H., Shindo, E., Mizuta, R., Ishii, M., Obata, A., & Adachi, Y. (2019a). MRI MRI-ESM2.0 model output prepared for CMIP6 CMIP. Version 20220715.Earth System Grid Federation. https://doi.org/10.22033/ESGF/CMIP6.621

Yukimoto, S., Koshiro, T., Kawai, H., Oshima, N., Yoshida, K., Urakawa, S., Tsujino, H., Deushi, M., Tanaka, T., Hosaka, M., Yoshimura, H., Shindo, E., Mizuta, R., Ishii, M., Obata, A., & Adachi, Y. (2019b). MRI MRI-ESM2.0 model output prepared for CMIP6 ScenarioMIP. Version 20220715.Earth System Grid Federation. https://doi.org/10.22033/ESGF/CMIP6.638

Supporting Information

Appendix S7: Taylor diagram comparing baseline of observed temperature data (1955-2017) and CMIP6 historical data (1950-2014). This diagram allows us to compare datasets by simultaneously displaying the correlation coefficient, the standard deviation and the Root Mean Square Deviation (RMSD). Here, the reference dataset is the baseline dataset (black dot on the plot). The ten coloured points correspond to the outputs of the ten CMIP6 models.

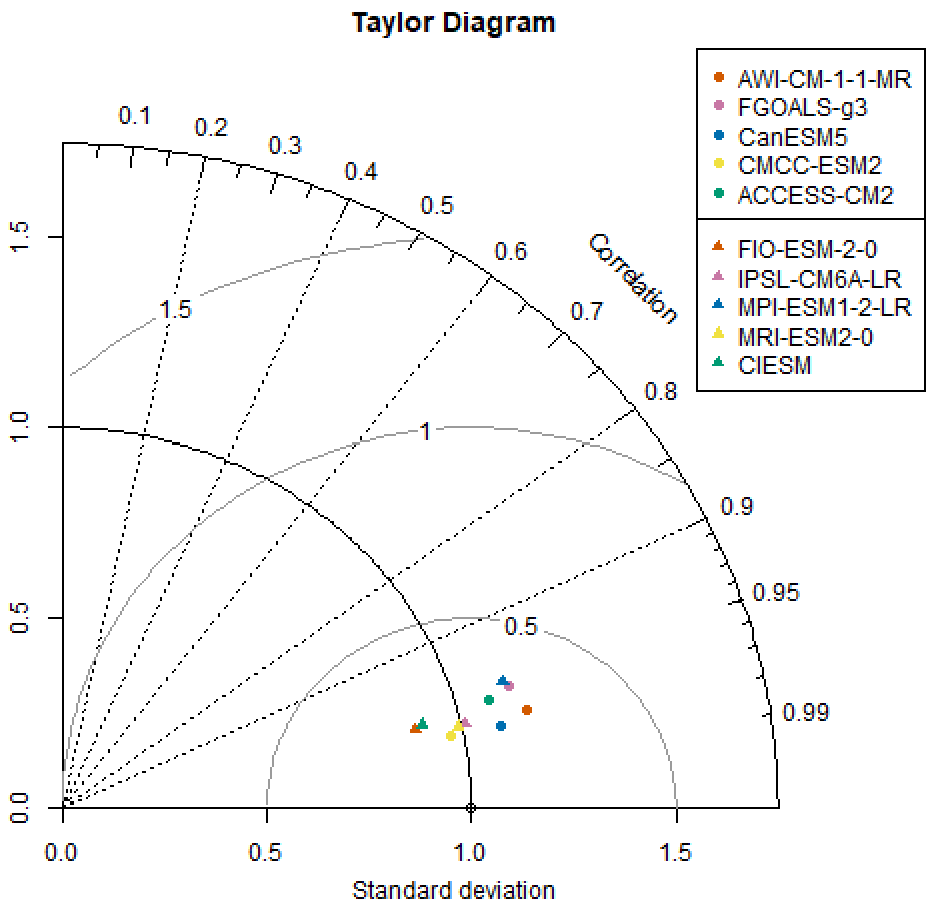

Supporting Information

Appendix S8: Summary of SDM outputs and performance. For each biogreographical region, we provide the number of presence and absence points, the importance scores of each environmental variable and the values of Jaccard index and AUC. The median, mean and standard deviation values are calculated over the five SDMs and four cross-validation runs for the variable importance scores. They are calculated over the four cross-validation runs for the model evaluation metrics.

Biogeographical regions	Nb presence points	Nb absence points	Variable importance																										
			temperature200			salinity200			oxygen200			chla2 5			bathymetry			chla200			oxygen500			salinity500			chla100		
			Median	Mean	SD	Median	Mean	SD	Median	Mean	SD	Median	Mean	SD	Median	Mean	SD	Median	Mean	SD	Median	Mean	SD	Median	Mean	SD	Median	Mean	SD
Region 1 (subtropical)	283	769	1	0.87	0.25	0,06	0,05	0,03	0,02	0,04	0,05	0	0,01	0,02	0,02	0,03	0,03	0	0,01	0,02	0	0,01	0,02	0,01	0,02	0,02	0	0,01	0,01
Region 2 (southern)	625	427	0,97	0.9	0.17	0	0,01	0,02	0	0,01	0,02	0	0,01	0,01	0,01	0,02	0,02	0	0	0,01	0,01	0,01	0,02	0,01	0,01	0,01	0	0	0,01
Region 3	32	1020	0,2	0,3	0,3	0,19	0,3	0,3	0,12	0,22	0,21	0	0,07	0,16	0,04	0,16	0,28	0	0,05	0,13	0,19	0,27	0,27	0,27	0,4	0,33	0,01	0,12	0,19
Region 4	51	1001	0,51	0,5	0,27	0,03	0,13	0,2	0,02	0,13	0,2	0,04	0,16	0,23	0,68	0,63	0,26	0,01	0,11	0,19	0,04	0,07	0,11	0,02	0,08	0,12	0,02	0,08	0,11

Biogeographical regions	Nb presence points	Nb absence points	Model evaluation metrics																													
			GLM						GAM						ANN						MARS						FDA					
			Jaccard index			AUC			Jaccard index			AUC			Jaccard index			AUC			Jaccard index			AUC			Jaccard index			AUC		
			Median	Mean	SD	Median	Mean	SD	Median	Mean	SD	Median	Mean	SD	Median	Mean	SD	Median	Mean	SD	Median	Mean	SD	Median	Mean	SD	Median	Mean	SD	Median	Mean	SD
Region 1 (subtropical)	283	769	0,7	0,71	0,06	0,95	0,96	0,01	0,7	0,71	0,06	0,95	0,96	0,01	0,7	0,71	0,06	0,95	0,96	0,01	0,69	0,7	0,06	0,95	0,94	0,03	0,69	0,7	0,06	0,95	0,94	0,03
Region 2 (southern)	625	427	0,95	0,93	0,04	0,99	0,98	0,02	0,95	0,93	0,04	0,98	0,98	0,02	0,95	0,93	0,04	0,99	0,98	0,02	0,95	0,93	0,04	0,98	0,98	0,02	0,95	0,93	0,04	0,98	0,97	0,02
Region 3	32	1020	0,12	0,15	0,09	0,82	0,84	0,06	0,11	0,15	0,08	0,86	0,85	0,05	0,06	0,09	0,08	0,74	0,69	0,12	0,18	0,18	0,08	0,83	0,83	0,06	0,14	0,16	0,08	0,87	0,85	0,08
Region 4	51	1001	0,34	0,32	0,19	0,92	0,88	0,12	0,28	0,29	0,22	0,94	0,92	0,05	0,31	0,3	0,24	0,92	0,9	0,07	0,23	0,26	0,21	0,86	0,87	0,04	0,2	0,25	0,27	0,68	0,69	0,16

Supporting Information

Appendix S9: Species fidelity and focus on region 3 species.
(a) Species fidelity ranges to their regions. Species fidelity is defined as the proportion of occurrence in the assigned region out of total occurrences. Well defined biogeographical regions are expected to have species with high fidelity values. **(b) Distribution of region 3 species and occurrence in sites of regions 1 and 2**. The colored points correspond to sites belonging to region 1 or 2 where there is at least one occurrence of a region 3 species. **(c) Temperature ranges of species of region 3 based on their distribution.** These ranges correspond to the temperature values encountered by the species in their occurrence sites.

(a)

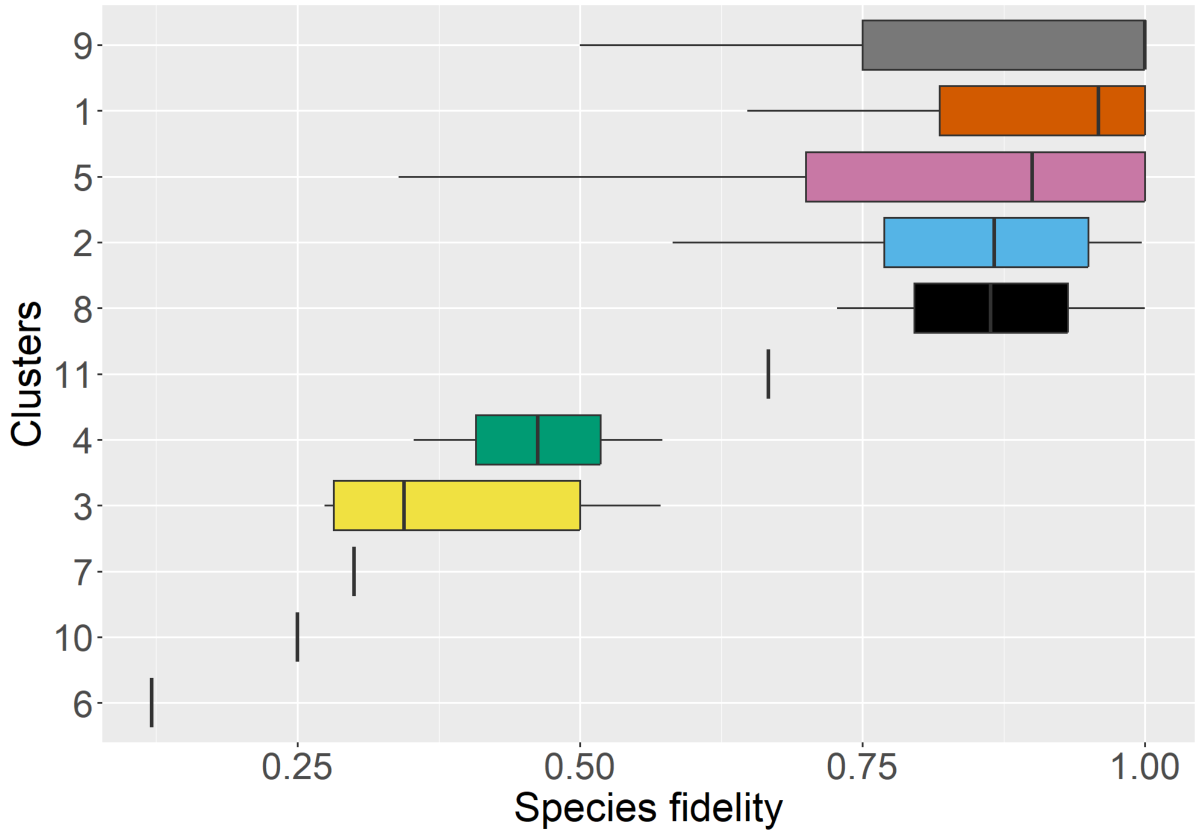

(b)

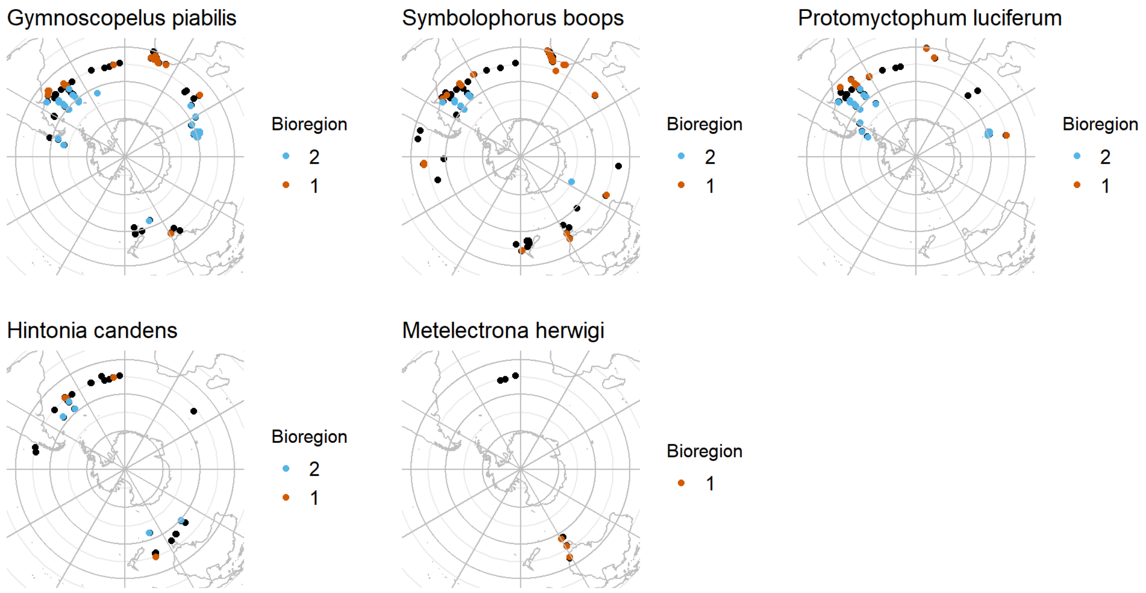
Gymnoscopelus piabilis
Symbolophorus boops
Protomyctophum luciferum
Hintonia candens
Metelectrona herwigi
Bioregion
2
1

(c)

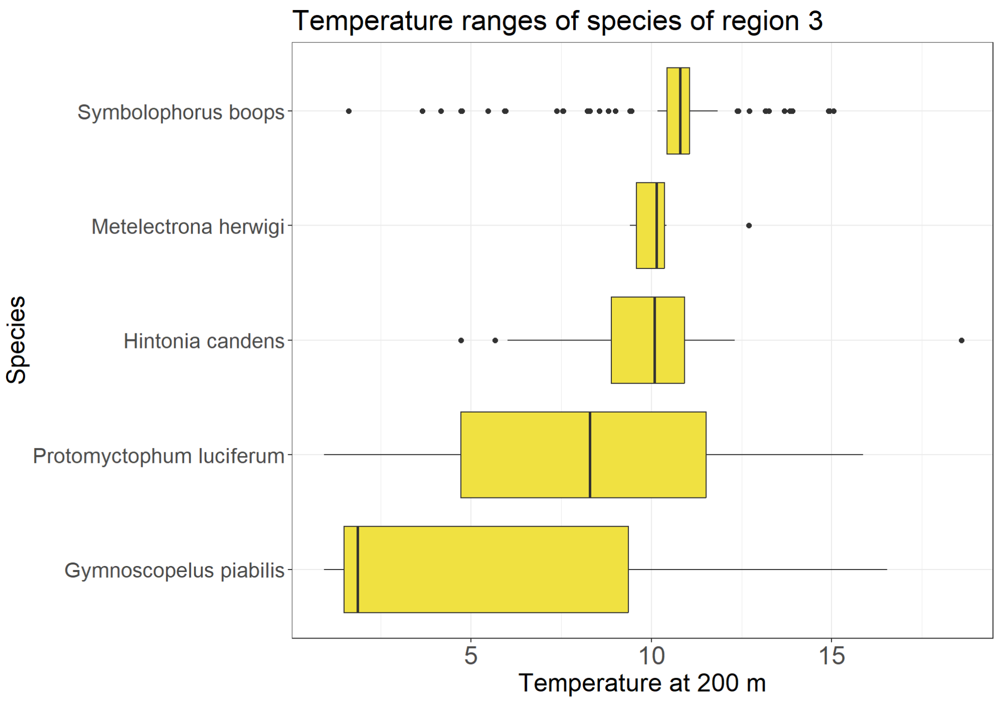
Temperature ranges of species of region 3
Symbolophorus boops
Metelectrona herwigi
Hintonia candens
Protomyctophum luciferum
Gymnoscopelus piabilis
Species
5
10
15
Temperature at 200 m

Supporting Information

Appendix S10: Observed response of myctophid species from all regions along the temperature gradient. Each curve represents the density of occurrence for one myctophid species across the temperature gradient at 200 m depth. Visual adjustments were applied to ensure a balanced representation of distributions. Therefore, the appearance of curves may differ slightly compared to Figure 4a, while maintaining the integrity of the underlying data distribution.

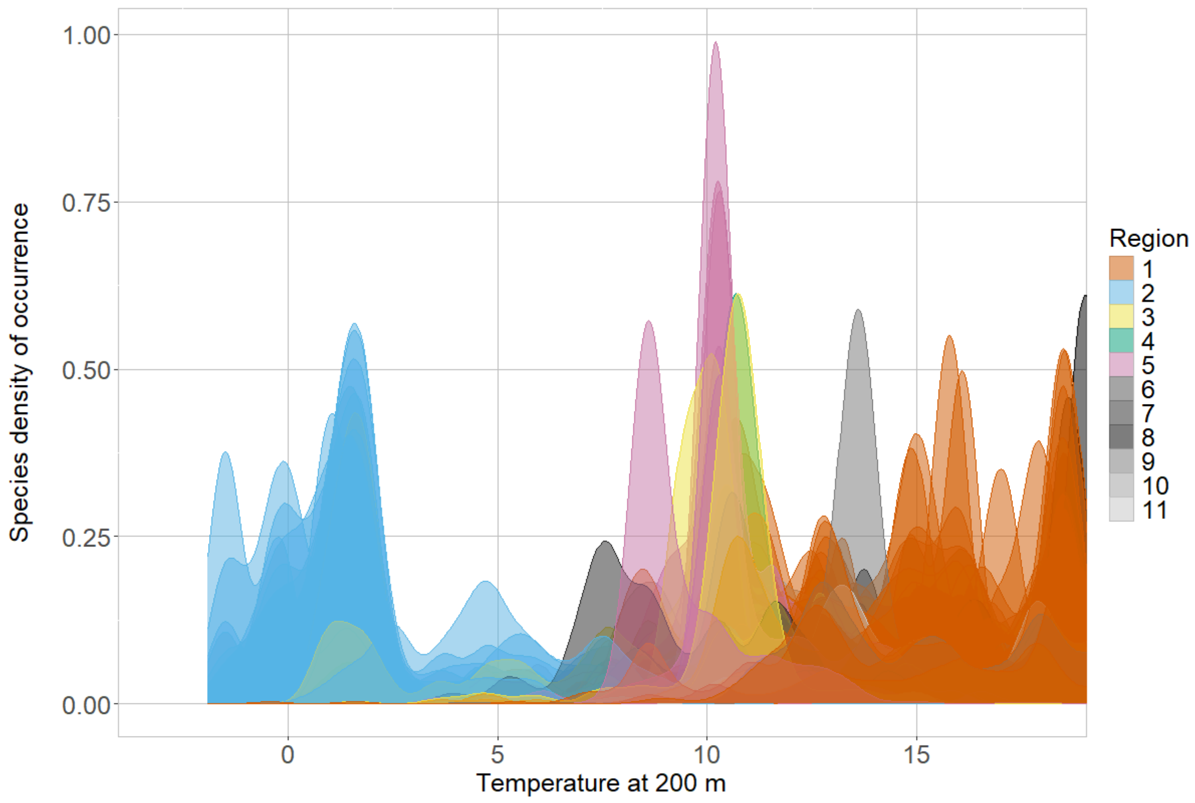

Supporting Information

Appendix S11: Observed distribution of species and sites from the subtropical and southern regions along gradients of other variables. Graphs were generated for each of the non-collinear variables that were selected by our variable selection approach: **(a)** salinity at 500 m, **(b)** oxygen at 200 m, **(c)** bathymetry. The first panel depicts the density of species occurrence along each variable gradient. Each curve represents the density distribution of one myctophid species. The second panel illustrates the distribution of sampling sites from the subtropical and southern regions along each variable gradient. Violin plots show the density of sites at each value: thicker violins indicate higher site densities.

(a)

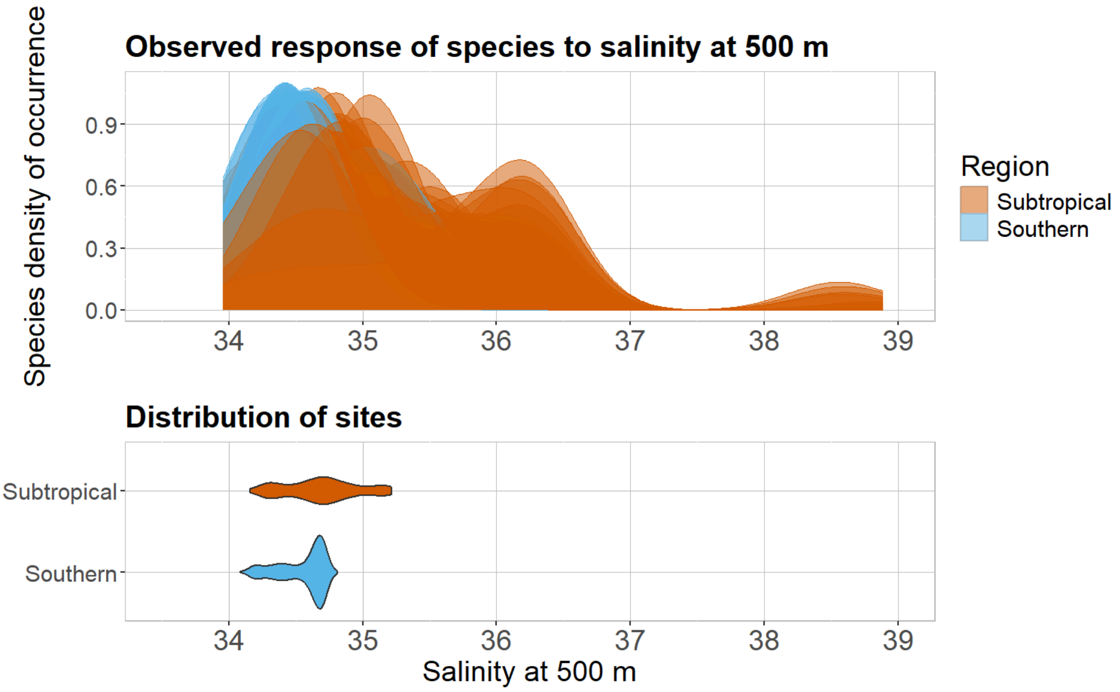

(b)

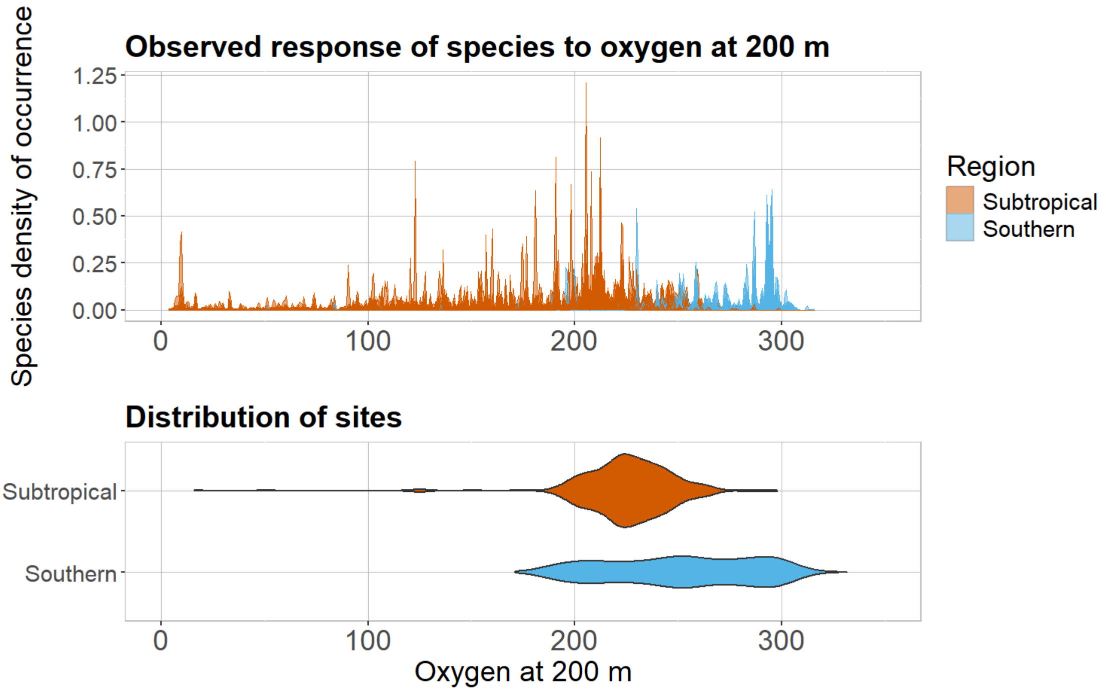

Observed response of species to oxygen at 200 m
Species density of occurrence
1.25
1.00
0.75
0.50
0.25
0.00
0
100
200
300
Region
Subtropical
Southern
Distribution of sites
Subtropical
Southern
0
100
200
300
Oxygen at 200 m

(c)

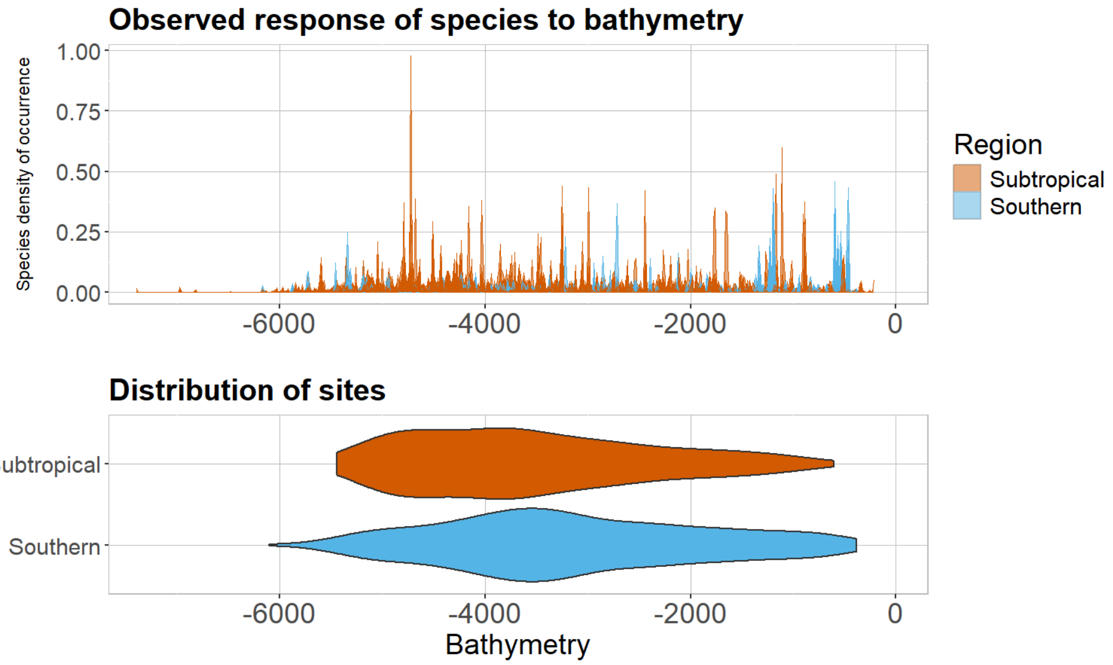

Observed response of species to bathymetry
Species density of occurrence
1.00
0.75
0.50
0.25
0.00
-6000
-4000
-2000
0
Region
Subtropical
Southern
Distribution of sites
Subtropical
Southern
-6000
-4000
-2000
0
Bathymetry

Supporting Information

Appendix S12: Detailed results of the variable selection protocol.

1) Selection of non-collinear variables

By calculating Spearman's correlation coefficient among all variables and applying a 0.7 threshold, we found three groups of correlated variables, one of them comprising all temperature variables, and multiple individual variables not showing collinearity patterns.

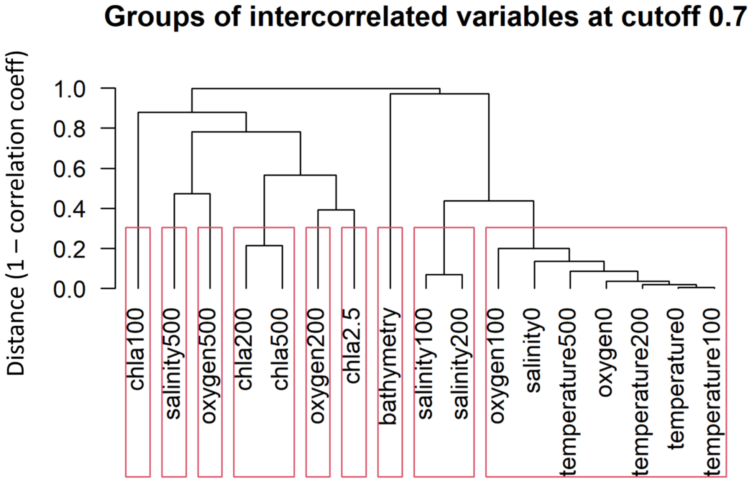

Figure S12a. Correlation between variables based on Spearman coefficient.

These results suggest that at our resolution of analysis (1° cells), for each of the three groups of collinear variables, the variables convey approximately the same meaning for the models. Therefore, the consequences of selecting one of the variables for each of these groups will be very limited for the models. Nevertheless, we opted to select one variable per group on the basis of a biological rationale. Among multiple depths for the same variable, we decided to keep the one at 200 m depth for the following reasons:

- water masses and fronts are well identified at this depth. For example, one of the definitions of the Polar Front is that its northern limit corresponds to the northern limit of the 2°C isotherm at 200 m (Orsi et al., 1995)
- most of the myctophid species that do diel vertical migration pass through the 200 m zone
- variables at 200 m were proven to be determinants of myctophid distribution in previous studies (Koubbi et al., 2011; Duhamel et al., 2014; Freer et al., 2019)

The variables selected at this first stage were:

- bathymetry
- chla2.5, chla100, chla200
- oxygen200, oxygen500
- salinity200, salinity500
- temperature200

2) Selection of important variables

We calculated the importance of each variable and for each region. The more a variable changes the predicted probabilities when it is resampled, the higher its importance.

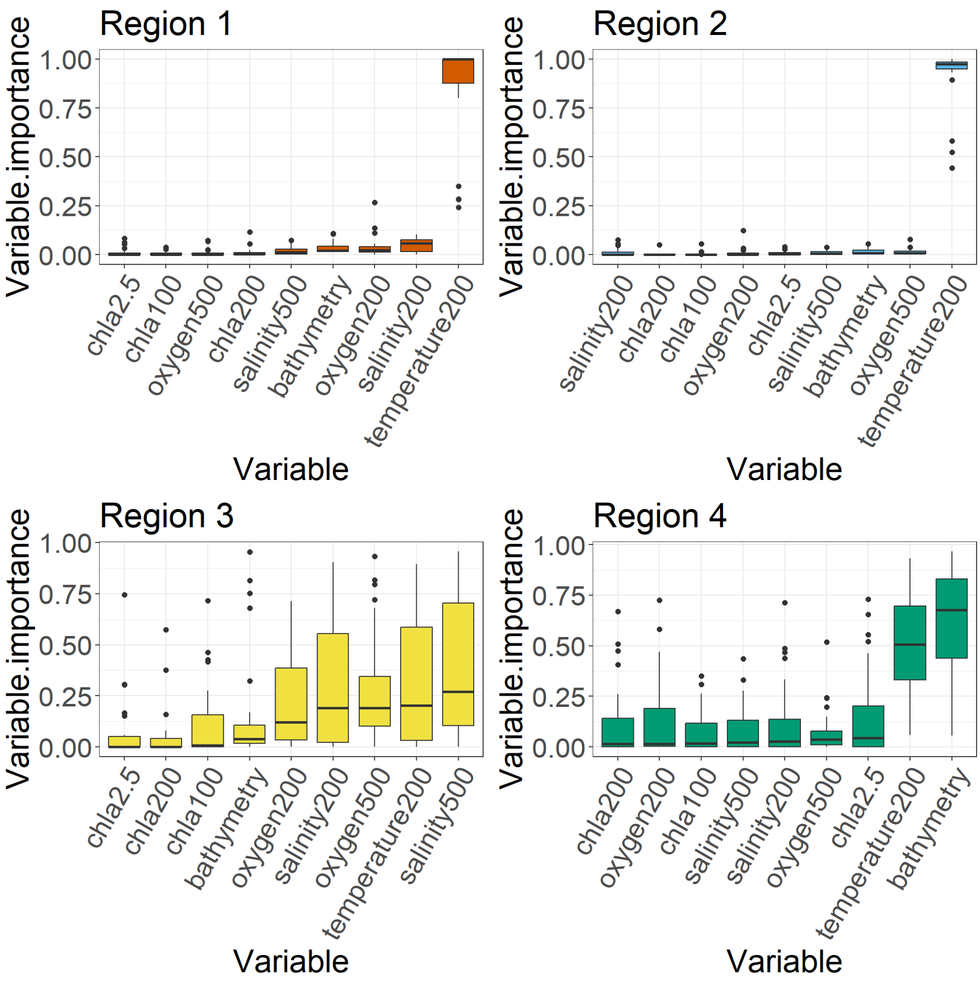

Figure S12b. Variable importance for the four main regions.

We selected variables with a median importance greater than 10%. Note that temperature at 200 m particularly stands out for region 1 (subtropical region) and region 2 (southern region).

The variables selected at this second stage were:

- cluster 1: temperature200
- cluster 2: temperature200
- cluster 3: oxygen200, oxygen500, salinity200, salinity500, temperature500
- cluster 4: temperature200, bathymetry

3) Selection of not mutually exclusive variables

We analysed the correlation among variable importances to select variables that were not negatively correlated. Between two negatively correlated variables, we kept the most important variable.

The variables selected at this final stage were:

- cluster 1: temperature200
- cluster 2: temperature200
- cluster 3: oxygen200, salinity500, temperature200
- cluster 4: temperature200, bathymetry

References:

Duhamel, G., Hulley, P.-A., Causse, R., Koubbi, P., Vacchi, M., Pruvost, P., Vigetta, S., Irisson, J.-O., Mormede, S., Belchier, M., Dettai, A., Detrich, H. W., Gutt, J., Jones, C. D., Kock, K.-H., Lopez Abellan, L. J., & Van de Putte, A. (2014). Biogeographic patterns of fish (C. De Broyer, P. Koubbi, H. Griffiths, B. Raymond, C. d'Udekem d'Acoz, A. Van de Putte, B. Danis, B. David, S. Grant, J. Gutt, C. Held, G. Hosie, F. Huettmann, A. Post, & Y. Ropert-Coudert, Éds.; p. 328‑362). Scientific Committee on Antarctic Research. https://nora.nerc.ac.uk/id/eprint/508228/

Freer, J. J., Tarling, G. A., Collins, M. A., Partridge, J. C., & Genner, M. J. (2019). Predicting future distributions of lanternfish, a significant ecological resource within the Southern Ocean. Diversity and Distributions, 25(8), 1259‑1272. https://doi.org/10.1111/ddi.12934

Koubbi, P., Moteki, M., Duhamel, G., Goarant, A., Hulley, P.-A., O'Driscoll, R., Ishimaru, T., Pruvost, P., Tavernier, E., & Hosie, G. (2011). Ecoregionalization of myctophid fish in the Indian sector of the Southern Ocean : Results from generalized dissimilarity models. Deep Sea Research Part II: Topical Studies in Oceanography, 58(1), 170‑180. https://doi.org/10.1016/j.dsr2.2010.09.007

Orsi, A. H., Whitworth, T., & Nowlin, W. D. (1995). On the meridional extent and fronts of the Antarctic Circumpolar Current. Deep Sea Research Part I: Oceanographic Research Papers, 42(5), 641‑673. https://doi.org/10.1016/0967-0637(95)00021-W

Supporting Information

Appendix S13: Individual projections and standard deviation maps. Individual projections of region occurrence probability of the five species distribution models and the four cross-validation runs are shown for **(a)** the subtropical region **(c)** the southern region. The standard deviation values are calculated across these five species distribution models and four cross-validation runs for **(b)** the subtropical region **(d)** the southern region.

(a) Individual projections of the Subtropical region

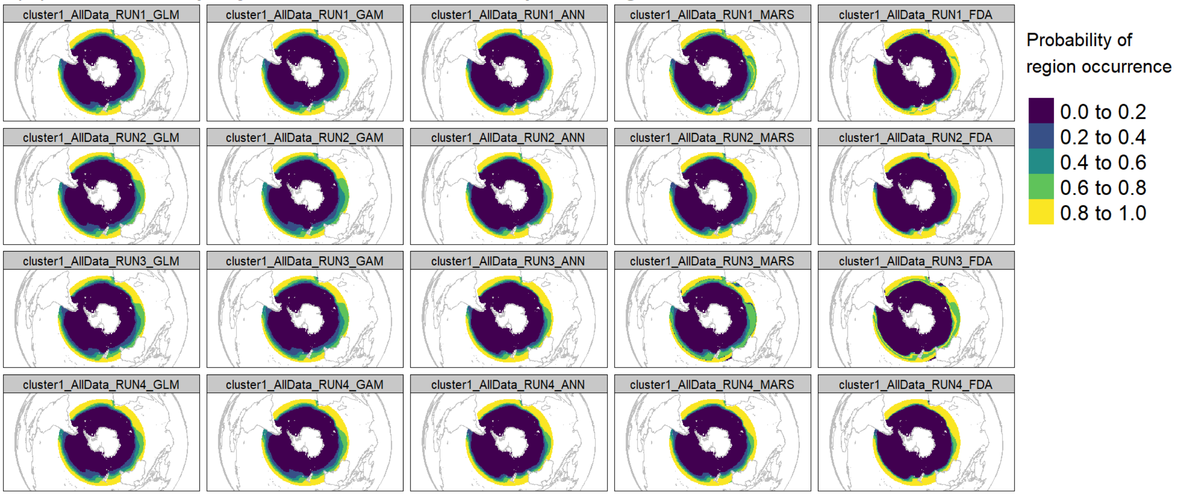

(b) Standard deviation for the Subtropical region

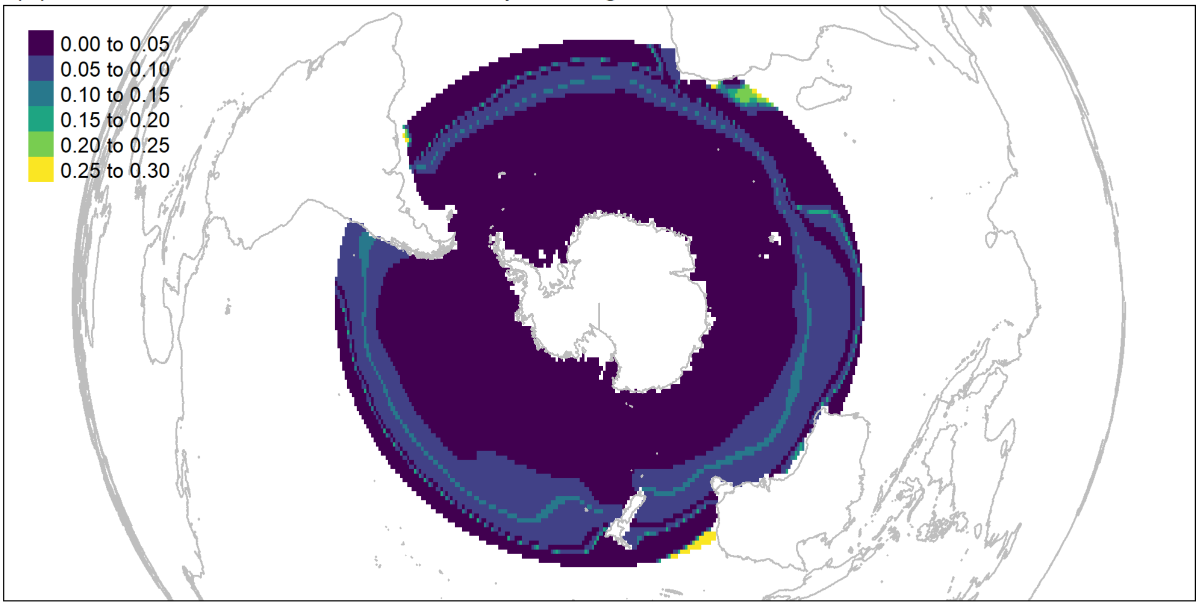

(c) Individual projections of the Southern region

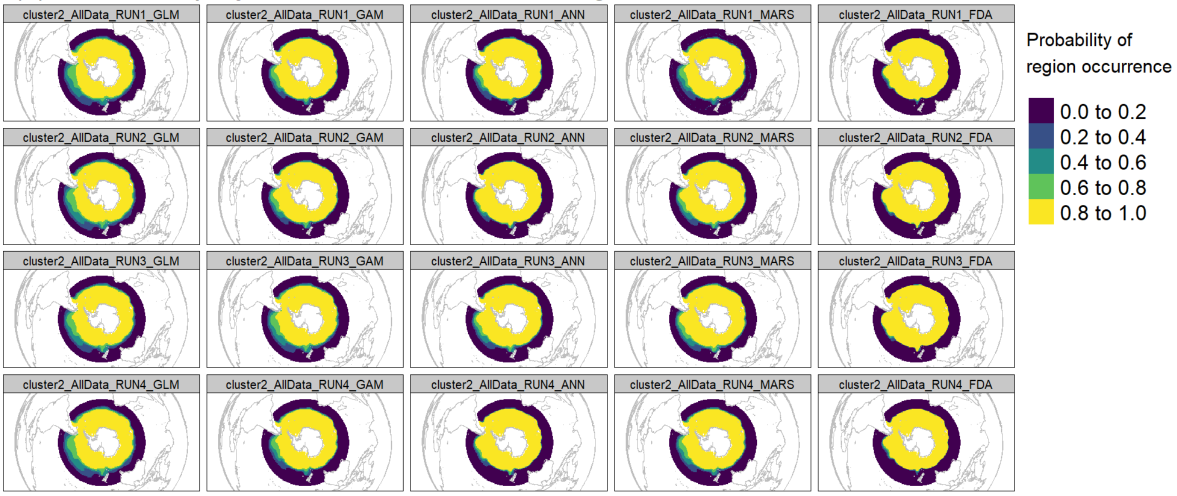

(d) Standard deviation for the Southern region

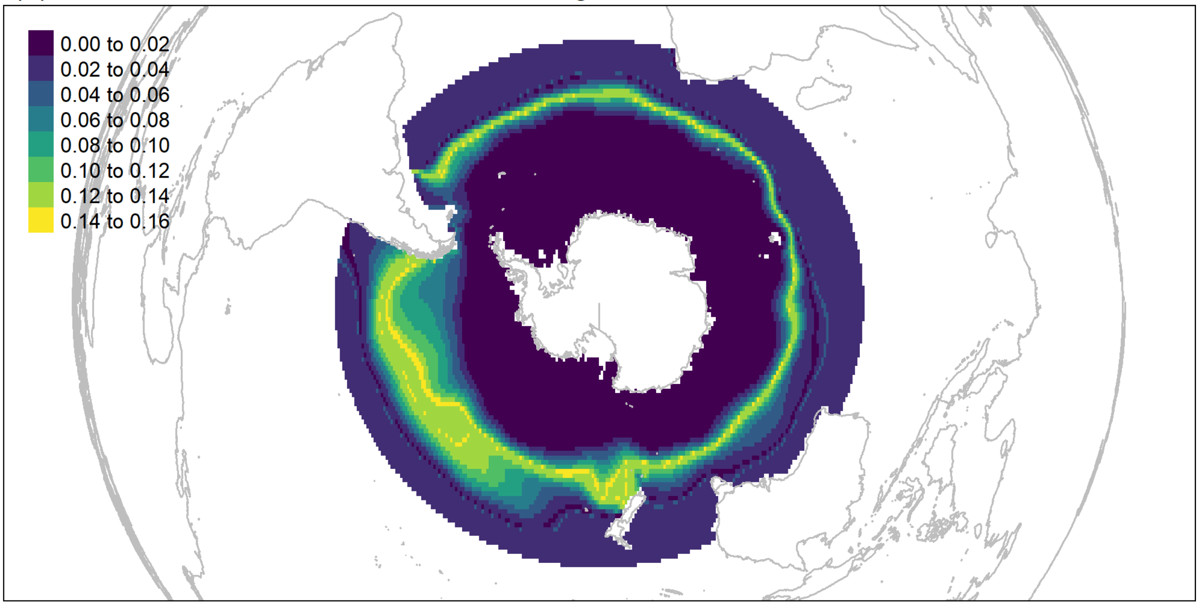

Supporting Information

Appendix S14: Future projections of regions and transition zone, and standard deviation, under three climate change scenarios and two time horizons. Projections are shown for **(a)** the Subtropical region, **(b)** the Southern region and **(c)** the transition zone, for the mid-term (2041-2060) and long-term (2081-2100) periods under the SSP1-2.6, SSP2-4.5, and SSP5-8.5 emission scenarios from CMIP6.
The standard deviation values are calculated across the five species distribution models, the four cross-validation runs and the ten global climate models.

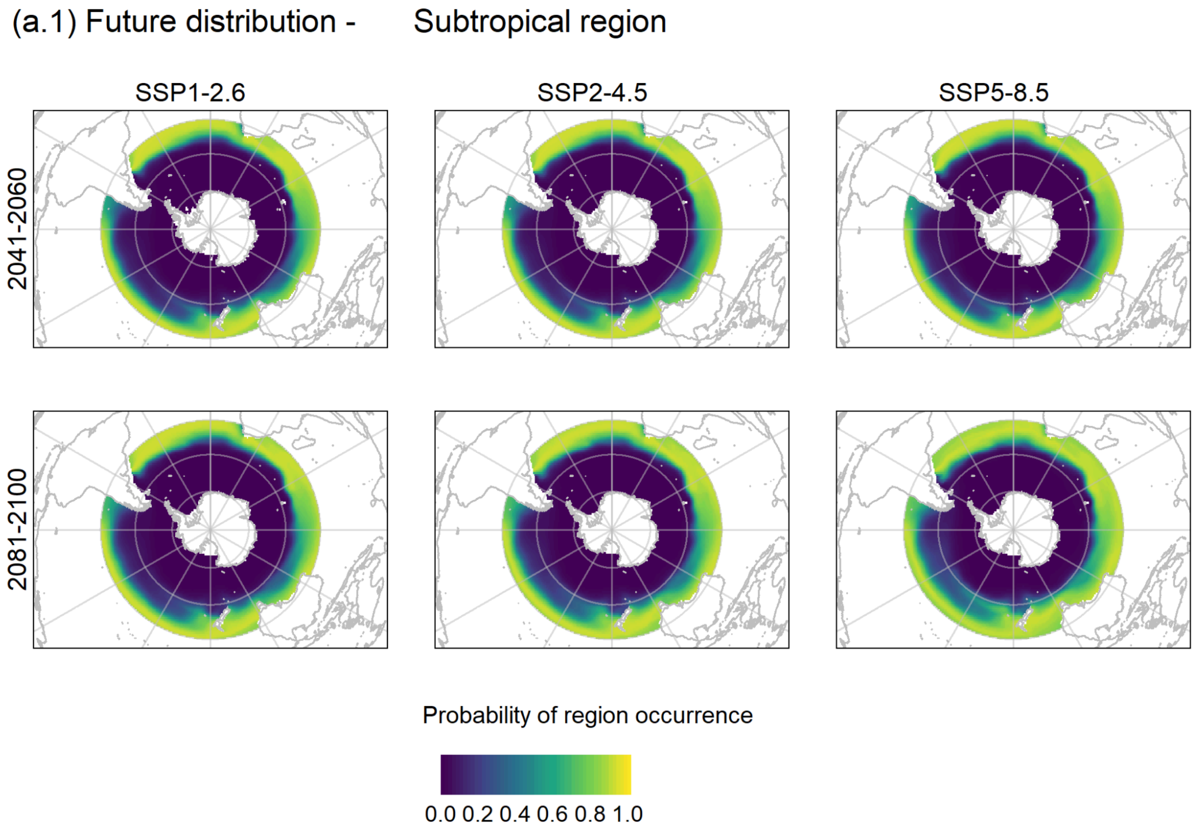

(a.2) Standard deviation - Subtropical region

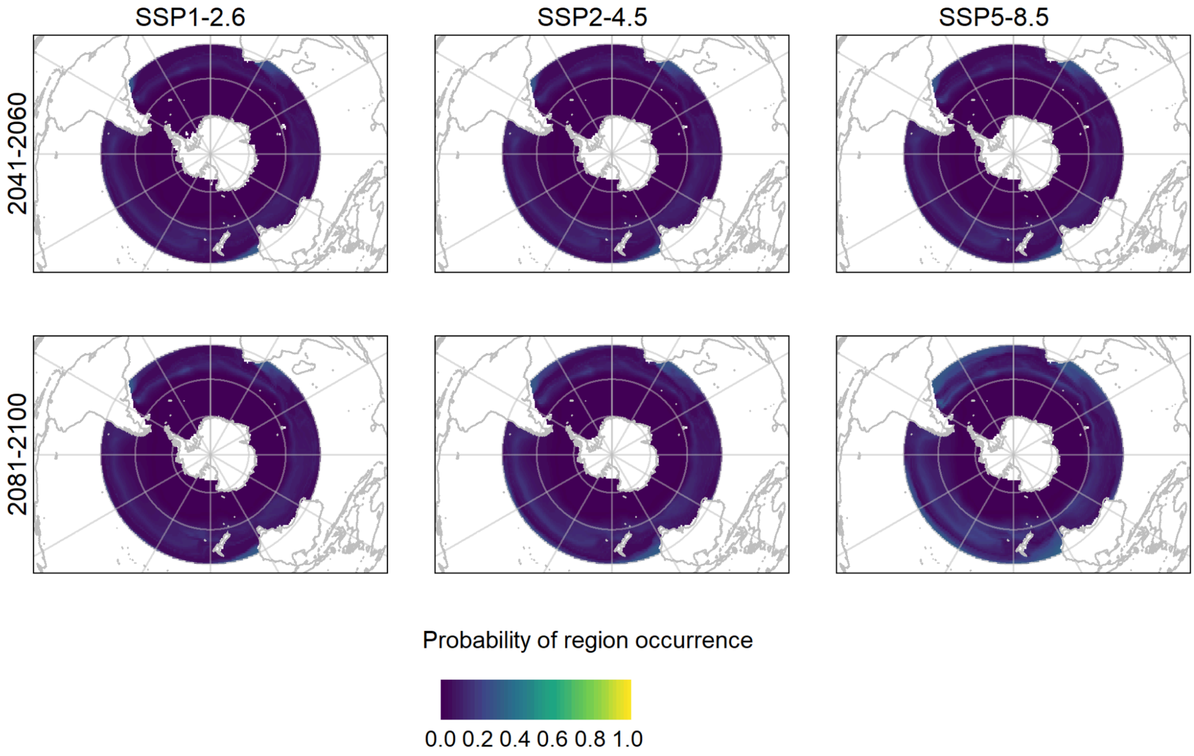

(b.1) Future distribution - Southern region

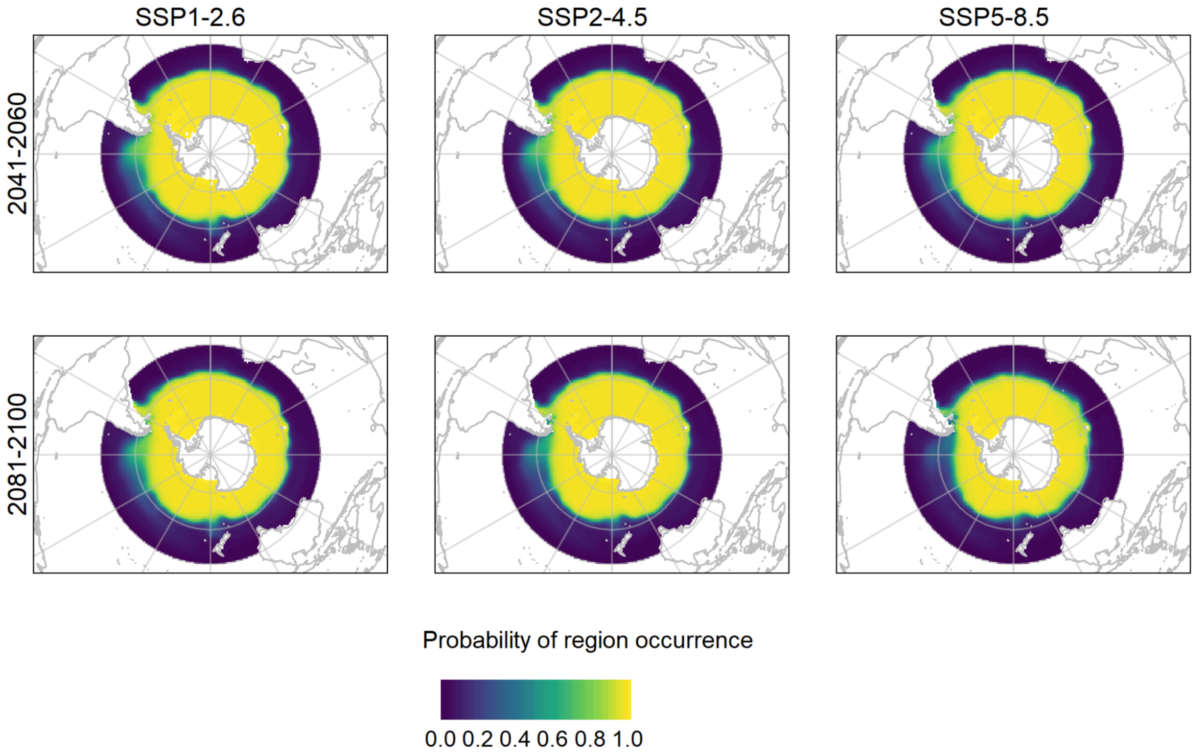

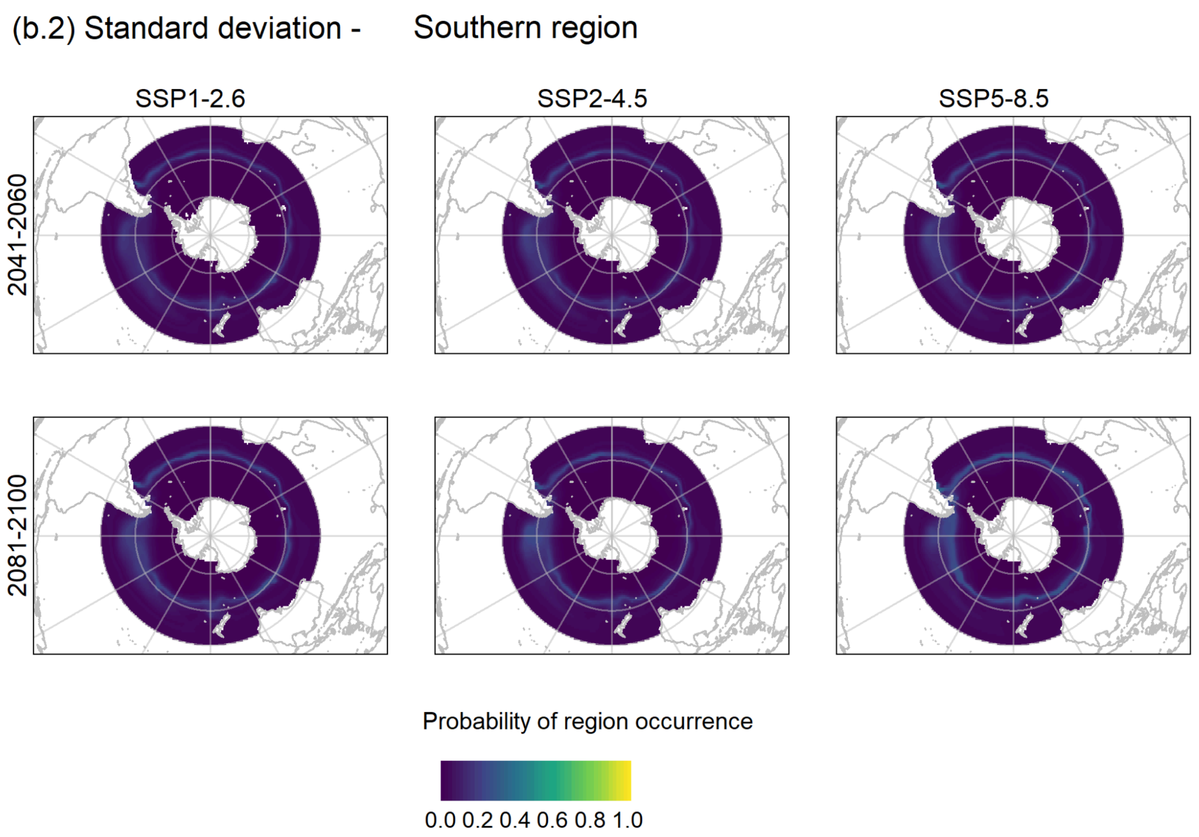
(b.2) Standard deviation - Southern region
SSP1-2.6
SSP2-4.5
SSP5-8.5
2041-2060
2081-2100
Probability of region occurrence
0.0 0.2 0.4 0.6 0.8 1.0

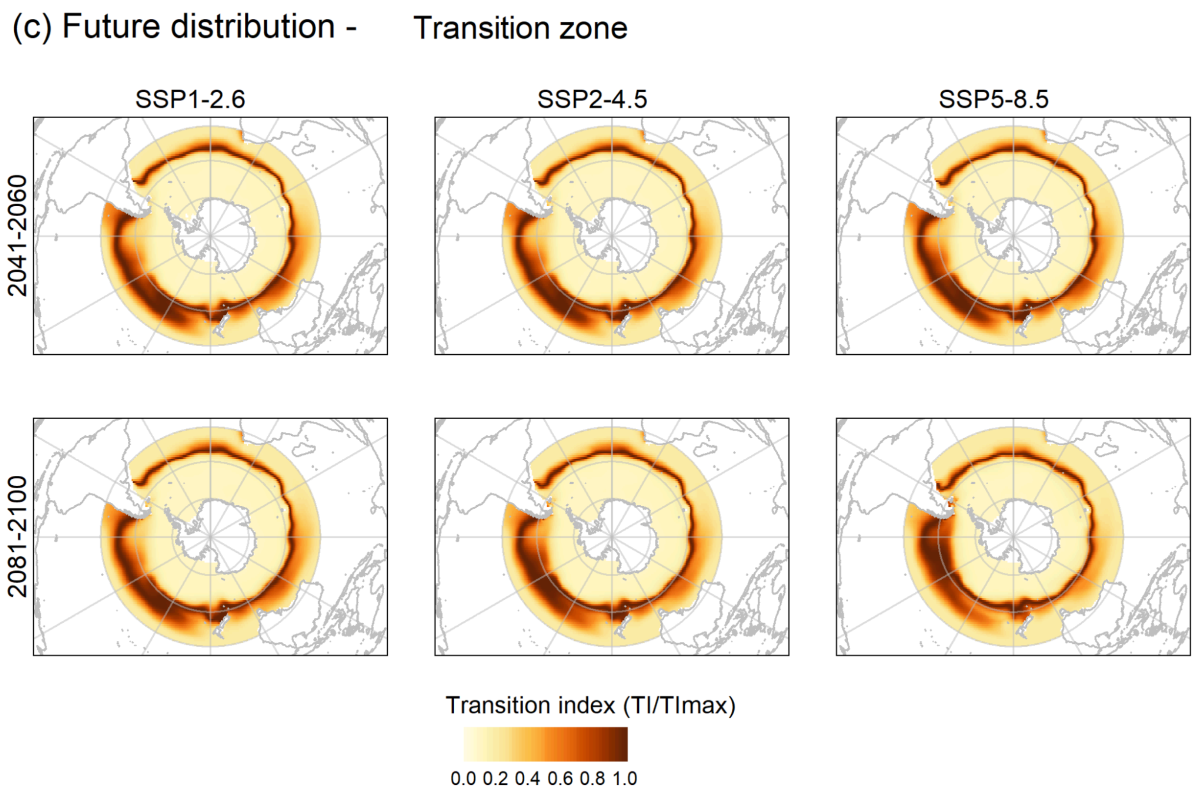
(c) Future distribution - Transition zone
SSP1-2.6
SSP2-4.5
SSP5-8.5
2041-2060
2081-2100
Transition index (TI/TImax)
0.0 0.2 0.4 0.6 0.8 1.0
